# Single-cell transcriptomics reveals that human milk feeding shapes neonatal immune cell interleukin signaling pathways in a nonrandomized clinical trial

**DOI:** 10.1016/j.ajcnut.2025.04.024

**Published:** 2025-04-26

**Authors:** Michael L Salinas, Bharath Kumar Mulakala, Laurie A Davidson, James J Cai, Sharon M Donovan, Robert S Chapkin, Laxmi Yeruva

**Affiliations:** 1 Department of Nutrition, Texas A&M University, College Station, TX, United States; 2 CPRIT Single Cell Data Science Core, Texas A&M University, College Station, TX, United States; 3 Texas A&M AgriLife Institute for Advancing Health Through Agriculture, College Station, TX, United States; 4 Microbiome and Metabolism Research Unit, USDA-ARS, SEA, Little Rock, AR, United States; 5 Arkansas Children's Nutrition Center, Little Rock, AR, United States; 6 Department of Veterinary Integrative Biosciences, Texas A&M University, College Station, TX, United States; 7 Department of Electrical and Computer Engineering, Texas A&M University, College Station, TX, United States; 8 Department of Food Science and Human Nutrition, Personalized Nutrition Initiative, University of Illinois, Urbana, IL, United States

**Keywords:** human milk, infant formula, peripheral blood mononuclear cells, inflammation, allergy

## Abstract

**Background:**

Several studies have indicated the benefits of human milk feeding to infants however, mechanisms behind positive health outcomes have not been investigated.

**Objectives:**

The study aimed to characterize circulating immune cell subpopulation gene expression in human milk-fed (HMF) compared with cow milk formula-fed (FF) infants using single-cell transcriptomics.

**Methods:**

Peripheral blood mononuclear cells (PBMCs) were isolated from healthy HMF (*n =* 6), and FF (*n =* 3) infants who were 3–3.5 mo old and enrolled in a nonrandomized clinical trial. Single-cell RNA sequencing was used to generate a PBMC atlas and evaluate gene expression in immune cell subsets. Differential expression analysis was performed on each cell type independently after clustering the cells by similar marker gene expression using the scGEAToolbox. Differentially expressed genes were subjected to pathway analyses using an online functional enrichment analysis program.

**Results:**

The relative abundance (%) of T and B lymphocytes, natural killer (NK) cells, and plasmacytoid dendritic cells were similar, whereas monocytes were higher in FF infants than in HMF infants (22.6 ± 10.7 compared with 8.3 ± 5.6; *P =* 0.0314). In addition, innate and adaptive immune cells from FF infants exhibited a higher activation state compared with HMF infants. We identified 16 distinct cell subsets from the major immune cell types: 3 monocyte subsets, 4 NK subsets, 2 B cell subsets, and 7 T cell subsets. Transcriptional profiles of each peripheral innate and adaptive immune cell subtype varied between HMF and FF infants. Pathway enrichment analysis of cell-specific transcriptional changes within subsets of major cell types revealed that the interleukin (IL)-4/IL-13 signaling pathways were upregulated in FF infants relative to HMF infants.

**Conclusions:**

These findings suggest that human milk downregulates peripheral immune cell cytokine transcriptional signatures linked to allergic inflammation and infection relative to formula feeding.

## Introduction

Clinical and epidemiological studies support the health benefits of human milk feeding (HMF) compared with formula-fed (FF), including reduced upper respiratory tract and gastrointestinal tract infections during infancy [1], lower rates of necrotizing enterocolitis [2], reduced risk of allergy [3], and decreased incidence of obesity in childhood [1]. Therefore, the WHO recommends exclusive HMF for the first 6 mo of life and continued breastfeeding for 2 y and beyond [4]. Although previous studies have documented the benefits of exclusive HMF, the mechanisms by which human milk contributes to the development of the infant’s innate and adaptive immune function are still unknown.

Human milk has a unique nutritional composition that helps shape the infant immune system [5]. Various clinical studies have demonstrated that circulatory immune cell composition was different in HMF infants compared with FF infants aged at 6 mo [6]. Carver et al. [7] showed a higher percentage of blood cluster differentiation (CD)4^+^ T cells in HMF compared with FF infants aged at 6 mo. In contrast, Andersson et al. [8] showed a lower percentage of blood CD4^+^ T cells in HMF infants compared with the same age FF infants. This heterogeneity may be attributed to: *1*) the fact that immune cells, particularly T lymphocytes, are highly dynamic during the first year of life, and differences vary based on the cohort, length of HMF, and time of sample collection [6,9]; *2*) analytical limitations precluding the ability to detect subsets of immune cell populations; *3*) age of the infants; and *4*) human milk compositional differences based on maternal diet and environment. Although the existing literature provides a snapshot of the influence of HMF on immune cell composition, subpopulations associated with different immune responses to the same immune stimuli have not been investigated. For example, in adults, classical monocytes have a higher expression of genes involved in immune responses, inflammasome signaling, and low-density lipoprotein uptake, whereas nonclassical monocytes exhibit a higher expression of genes involved in cytoskeletal dynamics, tissue invasion and inflammation to the same immune stimuli [10]. This heterogeneity in the transcriptional responsiveness of cell subsets highlights the complexity of the immune response.

We hypothesized that exclusive HMF shapes gene expression in peripheral immune cell subsets resulting in a lower inflammatory immune response. The primary goal was to assess the transcriptomic profile of peripheral immune cell subsets in HMF and FF infants at single-cell resolution, and the secondary outcome included identifying differentially expressed genes (DEGs) within each immune cell subset between the HMF and FF infants.

## Methods

### Blood collection

Blood samples were collected from (*n =* 9) 3- to 3.5-mo-old exclusively HMF or cow milk-based FF infants enrolled in the ongoing nonrandomized Happy Tummies Study (clinical trial# NCT03493711) at the Arkansas Children’s Nutrition Center between 24 October, 2020 and 26 May, 2021. The study was approved by the University of Arkansas for Medical Sciences Institutional Review Board (IRB #217562#) and the participants’ parent signed informed consent. FF infants received Similac Advance with prebiotics galacto-oligosaccharides (GOS) (Abbott Nutrition). Inclusion criteria included infants being exclusively fed with human milk since birth or exclusively fed with cow milk-based formula ≥2 mo before sample collection. Exclusion criteria included preterm delivery, infants with medical diagnosis, antibiotics/probiotics and prebiotics within 10–14 d of sample collection, or infants born to mothers taking medication during pregnancy (such as thyroid hormones, glucocorticoids, insulin, and oral hypoglycemic agents) or having pre-existing medical conditions (diabetes mellitus, gestational diabetes mellitus, hypertension, pre-eclampsia or eclampsia, malignancies, seizure disorder, lupus, severe psychiatric disorders) or smoking, alcohol drinking or drug use during pregnancy or lactation. The participant’s parent was asked over the phone to provide information regarding the infant race and ethnicity. The options provided for race included African American, American Indian or Alaskan Native, Asia, >1 race, Native Hawaiian or Other Pacific Islander, unknown, or not reported, and White; for ethnicity, the options included Hispanic and non-Hispanic. During the visit, infant anthropometric measurements were recorded using standardized techniques. For example, weight was recorded using a scale, and length was recorded using a length board with a fixed headpiece and sliding foot piece, followed by measuring head circumference using a tape measure.

### Peripheral blood mononuclear cell isolation

Mothers were instructed to not feed the infants 1 h before the blood draw, a trained phlebotomist collected blood from a peripheral vein. Peripheral blood mononuclear cell (PBMCs) were isolated from the blood samples (*n =* 6 HMF; *n =* 3 FF) using FICO/Lite-LymphoH density gradient media (R&D Systems, cat#I40150). Briefly, blood was collected in a evacuated tube containing heparin and centrifuged to remove the plasma, followed by the addition of an equal volume of phosphate-buffered saline (PBS) + 1 mM EDTA to the remaining blood sample. After thoroughly mixing, the sample was transferred into a 50 mL conical tube containing FICO-Lite and centrifuged at 470 × g for 25 min at 18° C. Centrifugation formed 3 layers; the middle layer containing PBMCs was transferred into a collection tube of Dulbecco's Modified Eagle's Medium (DMEM) and again centrifugated at 900 × g for 10 min at 18 °C. After discarding the supernatant, the cell pellet was counted and suspended in freezing media [1% 4-(2-hydroxyethyl)piperazine-1-ethanesulfonic acid, 20% fetal bovine serum (FBS), and 5% DMSO in DMEM], stored at −80 °C overnight and subsequently transferred to liquid nitrogen for long-term storage. The sample size was based on previous literature for single-cell omics where 3 samples/groups yielded meaningful results. Thus, we chose to use 3–6 samples/group.

### Single-cell RNA sequencing sample processing

PBMC samples were thawed according to the 10× Genomics PBMC Dropwise Thawing Protocol for Single Cell Assays (CG000447, Rev A) handbook. Briefly, frozen single-cell suspensions were thawed in a 37 °C water bath and gently transferred to a 50 mL conical tube. Warm complete growth media [10% FBS (VWR, 97068-085) in Roswell Park Memorial Institute Medium (Corning, 10-040-CV) media] was added dropwise to the cell suspension, followed by a sequential dilution in complete growth media as detailed in the PBMC Dropwise Thawing Protocol. Samples were then centrifuged and rinsed in complete growth media before resuspending cells in 0.04% bovine serum albumin (BSA) (Miltenyi Biotec, 130-091-376) in 1× PBS (Corning, 21-040-CV). Cell concentration was then determined using an automated cell counter (Nexcelom Cellometer K2) and ViaStain AOPI Staining Solution (Nexcelom, CS2-0106-5 mL).

Samples were fixed according to 10× Genomics Fixation of Cells and Nuclei for Chromium Fixed RNA Profiling (CG000478, Rev C) protocol. All samples were fixed for 20 h and kept on ice whereas cell concentration was determined with the Nexcelom Cellometer K2. Samples were then prepared for long-term storage according to the 10× protocol and stored at −80° C. Frozen samples were subsequently processed for the fixed RNA profiling (Flex) assay according to the 10× Genomics Chromium Fixed RNA Profiling Reagent Kits for Multiplexed Samples (CG000527, Rev E) user guide. Three to 4 samples were combined per pool (3 pools total). A 1:80 dilution of the sample pools was tested for quality control utilizing an Agilent Bioanalyzer High Sensitivity chip (Agilent, 5067-4626). Library quantification was performed with the KAPA Library Quantification Kit for Illumina Platforms (Roche, KK4824). Libraries were pooled based on the Chromium Connect Pooling Worksheet (CG000156) and prepared for Illumina sequencing platforms as recommended by 10× Genomics. Single-cell libraries were sequenced by Admera Health using a NovaSeq X Plus sequencer (Illumina). For the mapping of reads to transcripts and cells, sample demultiplexing, barcode processing, and unique molecular identifier (UMI) counts were performed using the 10× Genomics pipeline CellRanger v.8.0.0 [11]. Sequences were aligned to the human reference genome (GRCh38-2024-A) to generate the gene-barcode matrix.

Single-cell RNA sequencing (scRNA-seq) generates a vast number of observations per biological replicate, capturing cellular heterogeneity and enabling robust statistical analyses and highly granular data by profiling thousands of individual cells from each sample. Consequently, scRNA-seq provides exceptional statistical power, even with a modest number of biological replicates, as it offers insights at both the individual cell and population levels, enhancing the ability to identify meaningful biological patterns and differences [12]. Although our study included 6 infants fed human milk and 3 infants fed formula, each sample yielded an average of 4856 high-quality single-cell profiles, resulting in 43,709 cellular observations in total. This high-dimensional approach enabled robust detection of cell populations and transcriptional patterns, as each cell served as an independent measurement of the biological state.

### scRNA-seq sequencing data quality control and cell annotation

A gene-by-cell matrix was generated from the filtered feature gene-barcode matrix utilizing the scGEAToolbox (v24.4.6) from GitHub: (https://github.com/jamesjcai/scGEAToolbox) [13]. Specifically, to filter out low-quality cells, cells expressing: *1*) <500 or >50,000 UMIs; *2*) >20% mitochondrial reads; and/or, *3*) <200 unique genes, were excluded utilizing the quality control (QC) filtering feature of the scGEAToolbox. Genes expressed in <10 cells were also removed. Sample matrices were merged, doublets (via scrublet v0.2.3), and ambient RNA contamination (via decontX v1.2.0) were removed, and batch correction was performed (via harmonypy v0.0.10). Dimensionality reduction of expression matrices was performed using principal component analysis to identify the most informative components (*n* = 50) before generating a 2D uniform manifold approximation and projection (UMAP) embedding. Cell clustering was then conducted using the Leiden clustering algorithm, a graph-based community detection method that partitions cells into clusters by optimizing modularity and ensuring well-connected groups in the *k*-nearest neighbor graph (*k* = 15, res = 1) [14]. Following cell clustering, cell type annotation was performed with each cluster independently, after which clusters of cells with the same annotation were subsequently merged. These analyses were carried out utilizing the scGEAToolbox’s embedding and cell clustering features in an automatic manner. PanglaoDB, containing a community-curated cell-type marker database, along with well-known marker genes, was utilized for identifying cell types [15]. Discrete cell clusters were identified for B cells (including plasma B cells), hematopoietic stem cells, monocytes, natural killer (NK) cells, plasmacytoid dendritic cells, and T cells. Additionally, subsets of broad cell type clusters were re-embedded using a 2D UMAP projection, and Leiden-based cell clustering was performed to identify cell subtypes.

### Gene expression and normalization

Gene expression data were normalized by library size which scales single-cell gene expression counts by dividing each gene count by the total expression of its respective cell and multiplying by the mean library size across all cells, ensuring comparability across samples while accounting for differences in sequencing depth. Library size normalization was then followed by a log1p or log(*x*+1) transformation to minimize the effects of expression outliers whereas preventing high-abundance genes from dominating downstream analysis.

### Cell function and activation status scores

The UCell package in R was used for evaluating the gene signature enrichment scores for a predefined gene set in single-cell datasets [16]. The Molecular Signatures Database v2022.1 was used as the source of annotated gene sets for analysis. Briefly, the UCell package allows the estimation of the gene signature enrichment scores called UCell scores (Mann–Whitney U statistic) for a given predefined gene set based on the expression of mRNA in a respective cell. Details related to the gene sets are described in Supplemental Table 1.

### Differential gene expression and pathway enrichment analysis

Differential gene expression profiles in subsets of each immune cell type between the FF and HMF infants were examined. To identify DEGs, we utilized the differential expression Analysis function in scGEAToolbox, which is based on the Wilcoxon rank sum (Mann–Whitney U) test in MATLAB R2023b (v23.2) and false discovery rate estimation by the Benjamini and Hochberg method. Furthermore, to examine the potential function of DEGs, upregulated genes (log2FC > 1) were ranked based on adjusted *P* values. Enrichr along with the top 250 upregulated genes was utilized to perform pathway enrichment analysis focusing on the Reactome 2024 pathways [[17], [18], [19]]. The R package, ComplexUpset (v1.3.3), was utilized to visualize and compare the top 25 upregulated Reactome pathways from each major cell type and the top 10 upregulated Reactome pathways from each cell subtype [20].

## Results

### Participant characteristics

The exclusively HMF infants (*n =* 6) or FF (*n =* 3) infants were drawn from an ongoing nonrandomized clinical trial. The average birth weight and length were significantly different between the groups; the HMF group had higher birth weights and lengths (Table 1). Within the HMF group, 50% of infants were delivered vaginally, whereas 67% of FF infants were vaginally-delivered. Furthermore, the percentage of females and males in the HMF group was 67% and 33%, respectively, whereas in the FF group, the rate was 33% and 67%, respectively. The predominant ethnic group in the HMF and FF groups was White (83% and 67%, respectively).

**TABLE 1:** Characteristics of participants enrolled in the study.

Characteristics	HMF	FF	*P* value
Sample size (*n*)	6	3	—
Delivery method			
Vaginal	3 (50%)	2 (67%)	—
C-section	3 (50%)	1 (33%)	—
Sex			
Female	4 (67%)	1 (33%)	—
Male	2 (33%)	2 (67%)	—
Average birth weight (kg)	3.39 ± 0.24	2.87 ± 0.26	0.01
Average birth length (cm)	52.2 ± 0.014	47.8 ± 0.036	0.01
Race			
White	5 (83%)	2 (67%)	—
>1 race	1 (17%)		—
African American	—	1 (33%)	—

Mean±SD or percentage (%). One-way ANOVA was used to generate the *P* values.

Abbreviations: ANOVA, analysis of variance; HMF, human milk fed; FF, formula fed.

### Single-cell transcriptome profiles of PBMCs in HMF and FF infants

There was an average of 4856 PBMCs per sample from the HMF and FF groups. Cluster analysis identified 6 major populations in HMF and FF infants, which were annotated using cell-type-specific markers that included T cells, B cells (including plasma B cells), monocytes, NK cells, plasmacytoid dendritic cells, and hematopoietic stem cells (Figure 1A). Cell type and donor variation are depicted in Figure 1B and Supplemental Figure 1. Although marker genes utilized for cell type annotation are shown in Figure 1C. The relative proportion (%) of identified major cell types between HMF and FF infants is shown in Figure 1D and was not significantly different between the groups, except for circulating monocytes, which were higher in FF infants than HMF infants.

**FIGURE 1:**
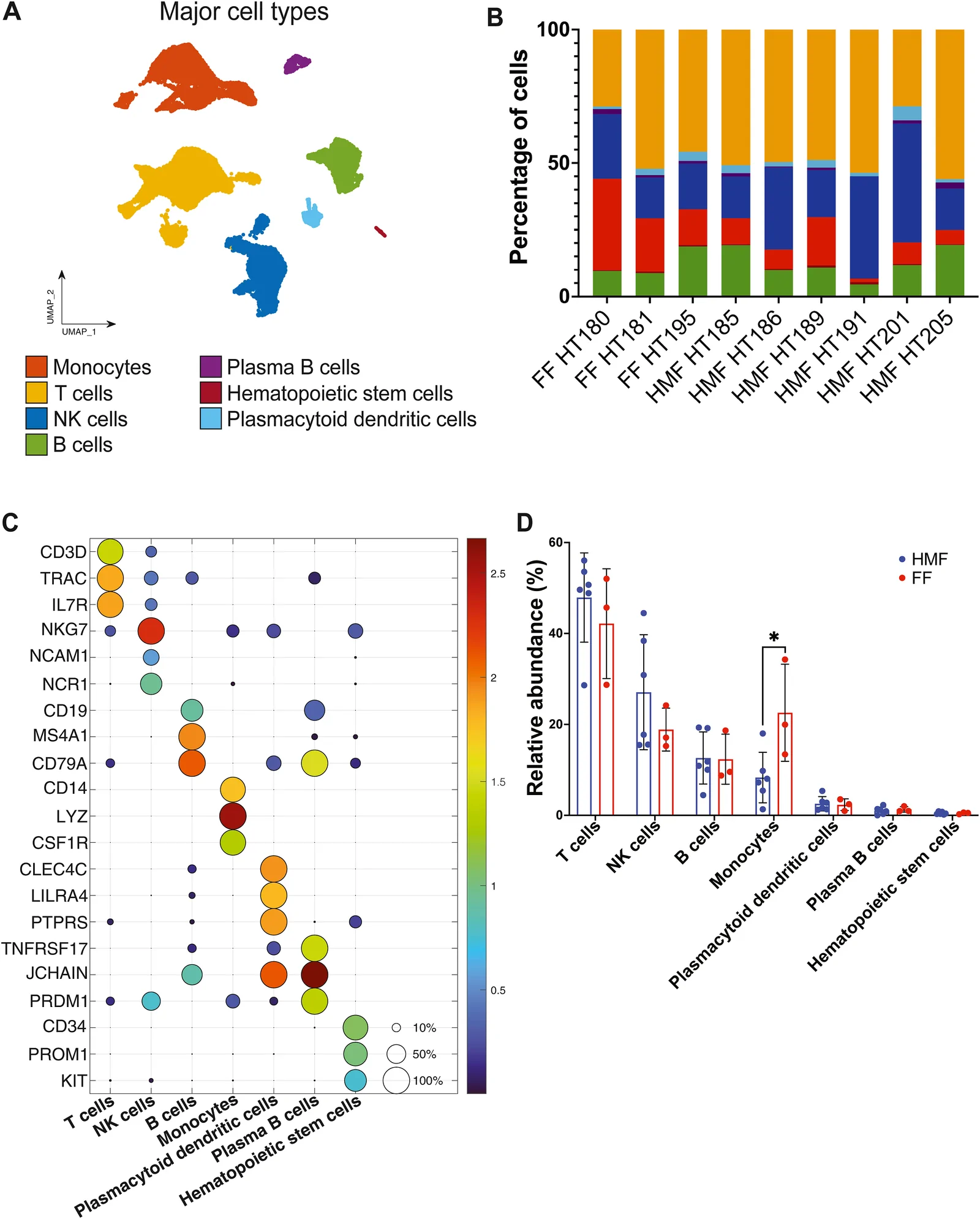
Clustering and major cell type identification. (A) UMAP showing cell clusters identified from scRNA-seq data derived from HMF (*n =* 6) and FF (*n =* 3) infants. (B) Stacked bar plot depicting cell type distribution by the donor. (C) A dot plot showing expression levels of specific markers used to identify each cell type; the color of the dot indicates the normalized expression level, and the size of the dot indicates the proportion of cells expressing the gene. Gene expression is normalized by library size and is log1p transformed. (D) Percentage of immune cell types in PBMCs of HMF and FF infants generated by scRNA-seq. The Sidak multiple comparisons test was conducted to generate *P* values. Mean ± SD, ∗*P* <0.05. HMF, human milk-fed; FF, formula-fed; scRNA-seq, single-cell RNA sequencing; PBMCs, peripheral blood mononuclear cells; UMAP, uniform manifold approximation and projection.

### HMF resulted in cell-specific transcriptional changes in peripheral immune cell types and distinct immune cell functions

Initially, we performed pathway enrichment scoring using the UCell R package to examine the functional differences in innate (monocytes and NK cells) and adaptive immune cells (B and T cells) between HMF and FF infants. Enrichment scores of biological processes for innate and adaptive immune cells were higher in FF infants in comparison to HMF infants (Table 2). In monocytes, enrichment scores for activation, aggregation, chemotaxis, and extravasation pathways were higher in FF infants, whereas enrichment scores for monocyte chemotactic 1 protein production and monocyte differentiation were higher in HMF infants. In contrast, enrichment scores for cytokine production and degranulation pathways were higher in NK cells from HMF infants. We observed that pathway enrichment scores were higher for biological processes, such as activation, proliferation, and differentiation in B, T, and NK cells, and chemotaxis in B and NK cells from FF infants compared with HMF infants. Additionally, the enrichment scores for receptor signaling and mediated immunity processes were higher in B cells from FF infants compared with HMF infants. Furthermore, we observed higher pathway enrichment scores for extravasation and exhaustion in the T cells of FF infants compared with HMF infants. Finally, enrichment scores for IL production were higher in T, NK, and B cells from FF infants; however, IL-13 production was higher in T cells from HMF infants. Overall, the study identified differences in immune cell activation and function associated with infant feeding mode.

**TABLE 2:** Enrichment scores of various biological processes in human milk (*n =* 6) and formula fed (*n =* 3) infant monocytes, NK cells, T cells, and B cells.

Biological process	HMF	FF	*P* value
Monocytes			
Activation	0.0596 ± 0.0462	0.0726 ± 0.0476	<0.0001
Aggregation	0.5878 ± 0.2173	0.6342 ± 0.2088	<0.0001
Chemotaxis	0.1362 ± 0.0286	0.1474 ± 0.0285	<0.0001
MCP1 production^3^	0.2556 ± 0.0700	0.2375 ± 0.0635	<0.0001
Differentiation	0.1732 ± 0.0350	0.1653 ± 0.0365	<0.0001
Extravasation	0.2236 ± 0.0737	0.2549 ± 0.0747	<0.0001
Natural killer cells			
Activation	0.1834 ± 0.0314	0.1891 ± 0.0326	<0.0001
Chemotaxis	0.3093 ± 0.1107	0.3251 ± 0.1117	<0.0001
Proliferation	0.0945 ± 0.0537	0.0969 ± 0.0522	0.0226
Cytokine production	0.1475 ± 0.1122	0.1401 ± 0.1133	0.0013
Degranulation	0.3944 ± 0.0977	0.3900 ± 0.0993	0.0441
Differentiation	0.1223 ± 0.0439	0.1277 ± 0.0466	<0.0001
IL-4 production	0.1137 ± 0.0364	0.1253 ± 0.0401	<0.0001
IL-13 production	0.0295 ± 0.0308	0.0304 ± 0.0303	0.1131
T cells			
Proliferation	0.1512 ± 0.0161	0.1563 ± 0.0176	<0.0001
Differentiation	0.2174 ± 0.0161	0.2244 ± 0.0169	<0.0001
Chemotaxis	0.1030 ± 0.0406	0.1059 ± 0.0410	<0.0001
Activation	0.2786 ± 0.0141	0.2839 ± 0.0149	<0.0001
Extravasation	0.0816 ± 0.0530	0.0858 ± 0.0545	<0.0001
Exhaustion markers	0.0088 ± 0.0292	0.0157 ± 0.0453	<0.0001
IL-4 production	0.1409 ± 0.0395	0.1433 ± 0.0390	0.0003
IL-13 production	0.0885 ± 0.0385	0.0870 ± 0.0385	0.0052
B cells			
Proliferation	0.1787 ± 0.0304	0.1855 ± 0.0316	<0.0001
Differentiation	0.2068 ± 0.0301	0.2139 ± 0.0313	<0.0001
Chemotaxis	0.1152 ± 0.0681	0.1173 ± 0.0676	0.3551
Activation	0.2295 ± 0.0238	0.2355 ± 0.0249	<0.0001
Receptor signaling	0.2790 ± 0.0480	0.2875 ± 0.0531	<0.0001
Mediated immunity	0.1321 ± 0.0175	0.1337 ± 0.0175	0.0004
IL-4 production	0.0868 ± 0.0352	0.0925 ± 0.0345	<0.0001
IL-13 production	0.0426 ± 0.0331	0.0426 ± 0.0324	0.9142

Mean ± SD. The Mann–Whitney U test was used to generate *P* values.

Abbreviations: HMF, human milk-fed; FF, formula-fed; MCP1, monocyte chemotactic protein 1; NK, natural killer.

### Monocyte subset analysis

To further explore potential subsets within each cell type, we utilized unique cell-specific markers (Supplemental Table 2). Functional heterogeneity of immune cell subtypes using pathway enrichment analysis of DEGs was performed in FF infants relative to the HMF infants. We identified 3 monocyte subsets (Figure 2A): classical (CD14^++^ CD16^−^), nonclassical (CD14^+^ CD16^++^), and FCER1A^+^ (CD14^+^ CD16^−^). UMAPs of monocyte marker genes are shown in Figure 2B. The top upregulated DEGs across the monocyte subsets are listed in Supplemental Table 3. In classical monocytes, 1835 genes were differentially expressed, of which 1178 and 657 were up- and downregulated, respectively, in FF infants compared with HMF infants. The upregulated DEGs in the classical monocytes were significantly enriched in 3 immune-related pathways (signaling by ILs, cytokine signaling in the immune system, and innate immune system). In the nonclassical subtype, we detected 440 DEGs (167 up- and 273 downregulated) in FF infants compared with HMF infants. In the FCER1A^+^ subtype, 184 DEGs were identified (152 up- and 32 downregulated) in FF compared with HMF infants. The upregulated DEGs in all monocytes were significantly enriched in 6 immune-related pathways, including the innate immune system, signaling by ILs, cytokine signaling in the immune system, IL-4 and IL-13 signaling, IL-10 signaling, and immunoregulatory interactions between a lymphoid and a nonlymphoid cell (Figure 2C).

**FIGURE 2:**
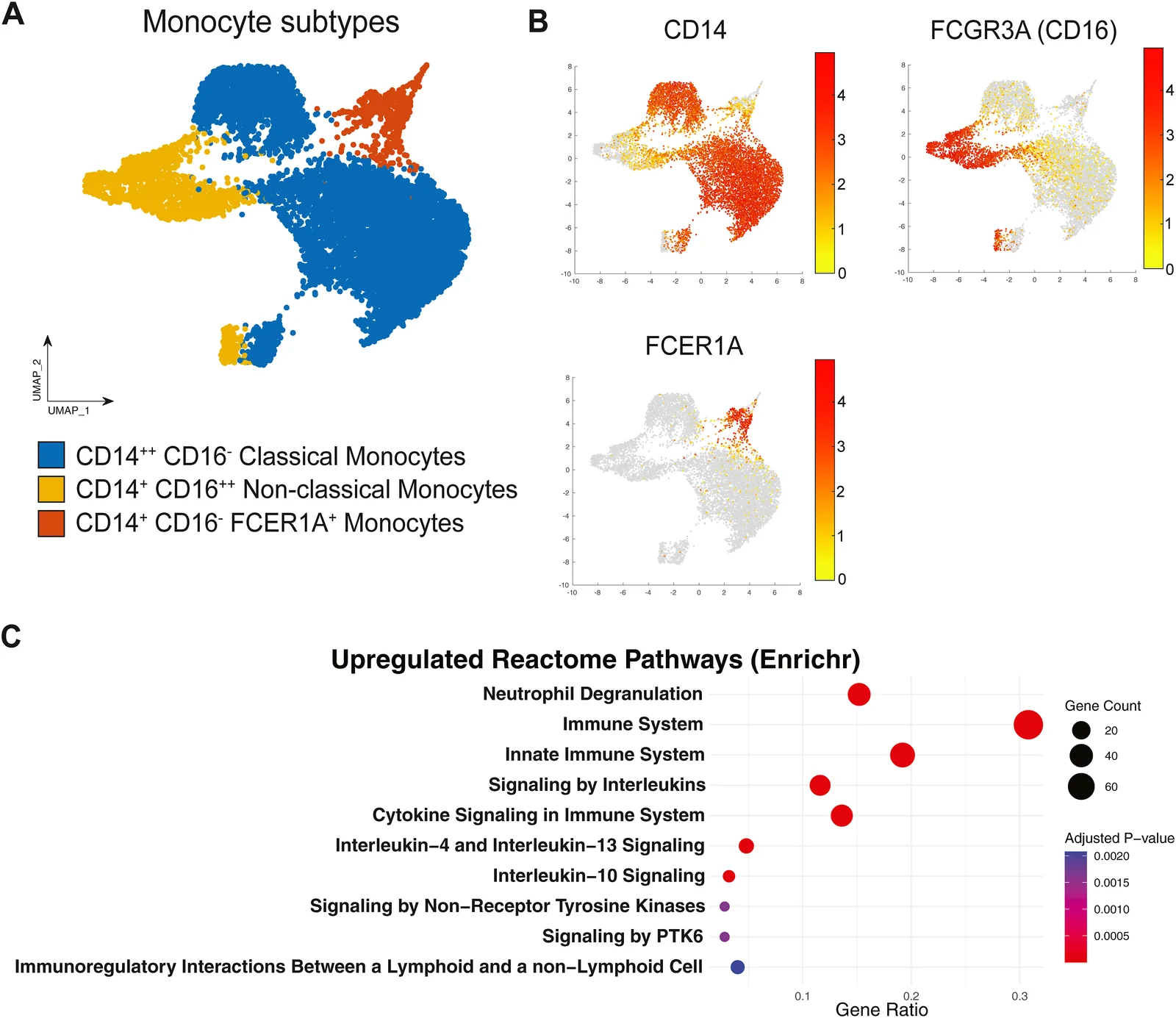
Human milk (*n =* 6) and formula-fed (*n =* 3) infant monocyte subset analysis. (A) UMAP plot showing the subsets of monocytes [classical (CD14^++^ CD16^−^), nonclassical (CD14^+^ CD16^++^), and FCER1A^+^ (CD14^+^ CD16^−^)]. (B) Monocyte marker gene UMAPs. (C) Enriched pathways of upregulated differentially expressed genes in all monocytes of formula-fed infants as identified by Reactome pathway enrichment analysis using the online program Enrichr (ranked by adjusted *P* value). The circle color indicates the adjusted *P* value, and the size indicates the number of genes detected in the pathway. CD, cluster differentiation; UMAP, uniform manifold approximation and projection.

### NK cell subset analysis

NK cells comprised 4 subsets (Figure 3A): CD56^+^ CD16^−^ NK cells, CD56^−^ CD16^+^ NK cells, proliferating (CD56^−^ CD16^+^) NK cells, and NK T-like cells. A small population of innate lymphoid cells was identified among the NK cell populations. UMAPs of NK cell marker genes are shown in Figure 3B. The top upregulated DEGs across the NK cell subsets are listed in Supplemental Table 4. We identified 301 DEGs (207 up- and 94 downregulated) in CD56^+^ CD16^−^ NK cells, 494 DEGs (391 up- and 103 downregulated) in CD56^−^ CD16^+^ NK cells, 25 DEGs (15 up- and 10 downregulated) in proliferating (CD56^−^ CD16^+^) NK cells, and 98 DEGs (73 up- and 25 downregulated) in NK T-like cells in FF infants relative to the HMF infants. The upregulated DEGs in CD56^+^ CD16^−^ NK cells were significantly enriched in 3 immune-related pathways (translocation of ZAP-70 to immunological synapse, immunoregulatory interactions between a lymphoid and a nonlymphoid cell, and PD-1 signaling), whereas upregulated DEGs in CD56^−^ CD16^+^ NK cells were significantly enriched in 5 immune-related pathways (immunoregulatory interactions between a lymphoid and a nonlymphoid cell, signaling in the immune system, IL-4 and IL-13 signaling, innate immune system, and IL-10 signaling). Overall, 6 pathways (immunoregulatory interactions between a lymphoid and a nonlymphoid cell, innate immune system, IL-4, and IL-13 signaling, cytokine signaling in the immune system, IL-7 signaling, and signaling by ILs) were upregulated in all NK cell subsets from FF infants compared with HMF infants (Figure 3C).

**FIGURE 3:**
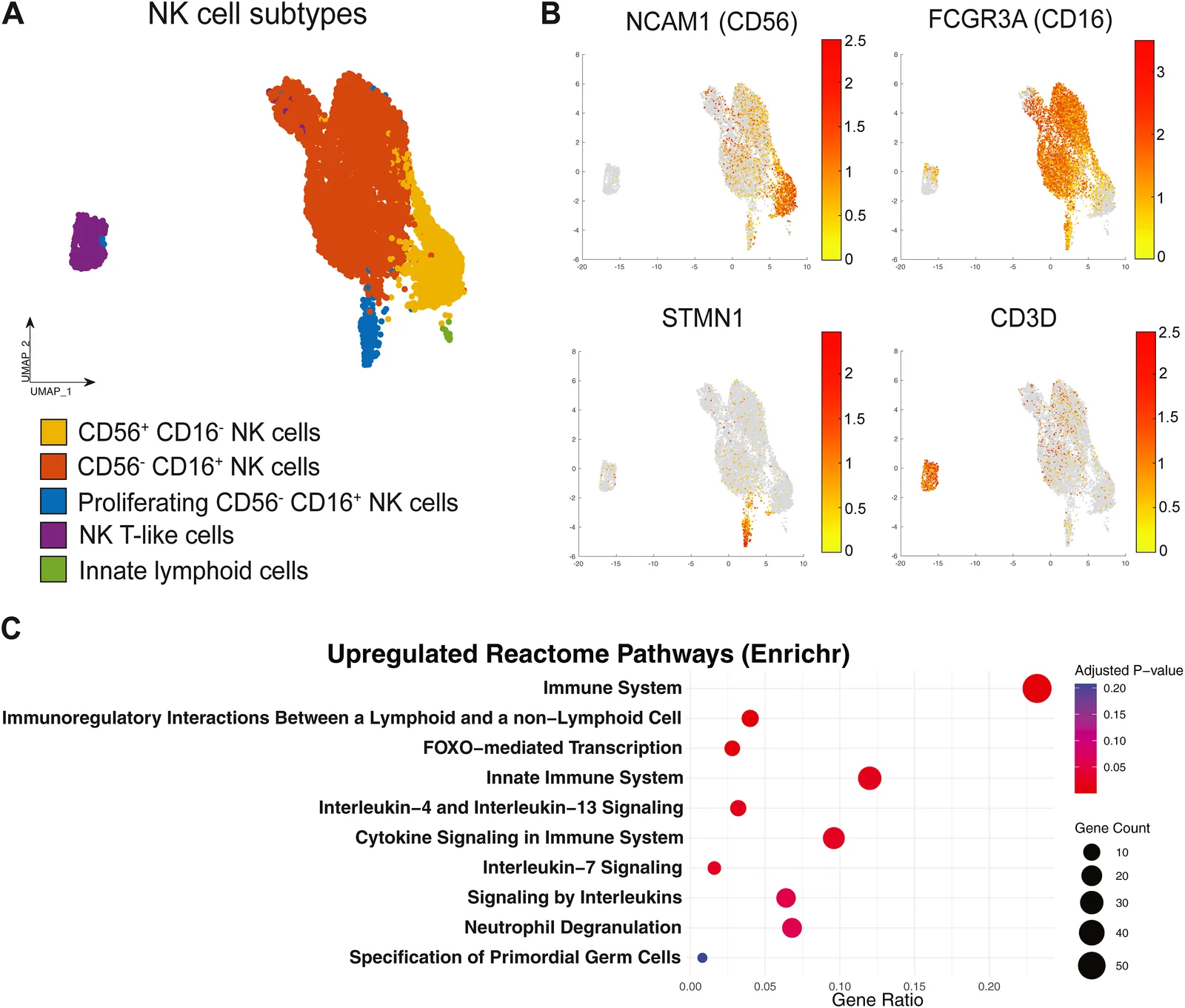
Human milk (*n =* 6) and formula-fed (*n =* 3) infant natural killer (NK) cell subset analysis. (A) UMAP plot showing the subsets of NK cells [CD56^+^ CD16^−^ NK cells, CD56^−^ CD16^+^ NK cells, proliferating (CD56^−^ CD16^+^) NK cells, NK T-like cells, and innate lymphoid cells]. (B) NK cell marker gene UMAPs. (C) Enriched pathways of upregulated DEGs in all NK cells of formula-fed infants as identified by Reactome pathway enrichment analysis using the online program Enrichr (ranked by adjusted *P* value). The color of the circle indicates the adjusted *P* value, and the size indicates the number of genes detected in the pathway. CD, cluster differentiation; DEGs, differentially expressed genes; UMAP, uniform manifold approximation and projection.

### B cell subset analysis

We identified 2 B cell subsets (Figure 4A): B cells and plasma B cells. UMAPs of B cell marker genes are shown in Figure 4B. The top upregulated DEGs across the B cell subsets are listed in Supplemental Table 5. We observed 131 DEGs (107 up- and 24 downregulated) in the plasma B cells, and 1812 DEGs (1744 up- and 68 downregulated) in the B cell subset in FF infants relative to the HMF infants. The upregulated DEGs in B cells were significantly enriched in 5 immune-related pathways [IL-4 and IL-13 signaling, innate immune system, cytokine signaling in the immune system, signaling by ILs, and regulation of interferon gamma (IFN-γ) signaling], whereas upregulated DEGs in plasma B cells were significantly enriched in 5 immune-related pathways (IL-4 and IL-13 signaling, IL-10 signaling, signaling by ILs, IL-20 family signaling, and IL-9 signaling). Overall, we observed that 5 common pathways in all B cell subsets (IL-4 and IL-13 signaling, cytokine signaling in the immune system, signaling by ILs, innate immune system, and regulation of IFNG signaling) were upregulated in FF infants compared with HMF infants (Figure 4D).

**FIGURE 4:**
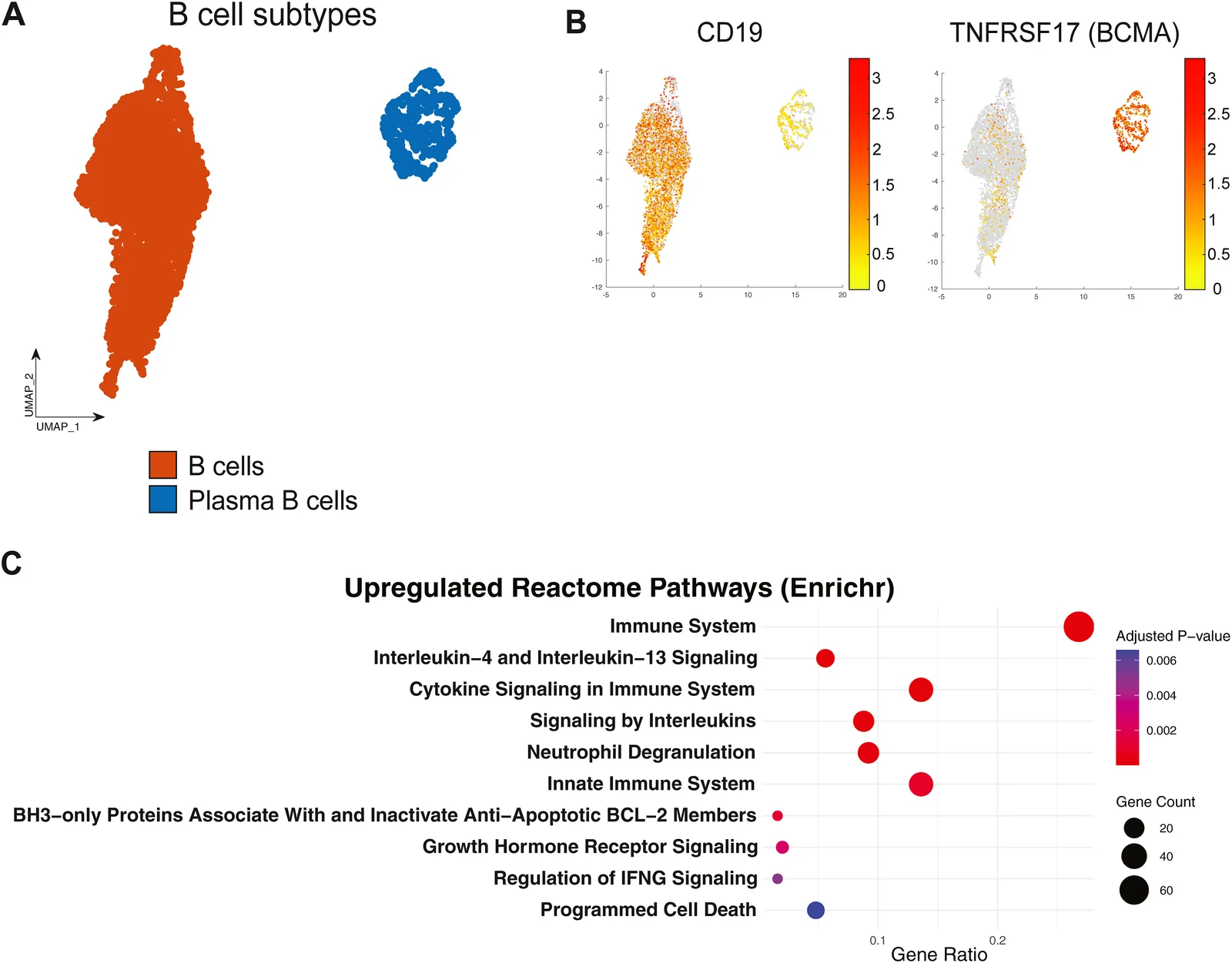
Human milk (*n =* 6) and formula-fed (*n =* 3) infant B cell subset analysis. (A) UMAP plot showing the subsets of B cells (B cells and plasma B cells). (B) B cell marker gene UMAPs. (C) The list of enriched pathways of upregulated differentially expressed genes in all B cell subsets of formula-fed infants as identified by Reactome pathway enrichment analysis using the online program Enrichr (ranked by adjusted *P* value). The circle color indicates the adjusted *P* value, and the size indicates the number of genes detected in the pathway. UMAP, uniform manifold approximation and projection.

### T cell subset analysis

T cells consisted of 7 subsets (Figure 5A): CD4^+^ T cells, regulatory CD4^+^ T cells, Th1 CD4^+^ T cells, T helper type 2 cell (Th2) CD4^+^ T cells, CD8^+^ T cells, gamma-delta (γδ) T cells, and double negative (CD4^−^ CD8^−^) T cells. UMAPs of T cell marker genes are shown in Figure 5B. The top upregulated DEGs across the T cell subsets are listed in Supplemental Table 6. We identified 1606 DEGs (1529 up- and 77 downregulated) in the CD4^+^ T cell subset, 8 upregulated DEGs in CD4^+^ regulatory T cells, 44 DEGs (16 up- and 28 downregulated) in Th1 CD4^+^ T cells, and 21 DEGs (20 up- and 1 downregulated DEGs in Th2 CD4^+^ T cells, 318 DEGs (284 up- and 34 downregulated) in CD8^+^ T cells, and 36 DEGs (29 up- and 7 downregulated) in γδ T cells in FF infants relative to the HMF infants. Upregulated DEGs in all CD4^+^ T cells were significantly enriched in 4 immune-related pathways (IL-4 and IL-13 signaling, signaling by ILs, cytokine signaling in the immune system, and interferon-gamma signaling), whereas upregulated DEGs in CD8^+^ T cells were significantly enriched in 5 immune-related pathways (interferon-gamma signaling, IL-4, and IL-13 signaling, interferon alpha beta signaling, cytokine signaling, and interferon signaling). As shown in Figure 5C, upregulated DEGs in all T cells from FF infants were significantly enriched in 4 immune-related pathways including IL-4 and IL-13 signaling, signaling by ILs, cytokine signaling in the immune system, and interferon-gamma signaling.

**FIGURE 5:**
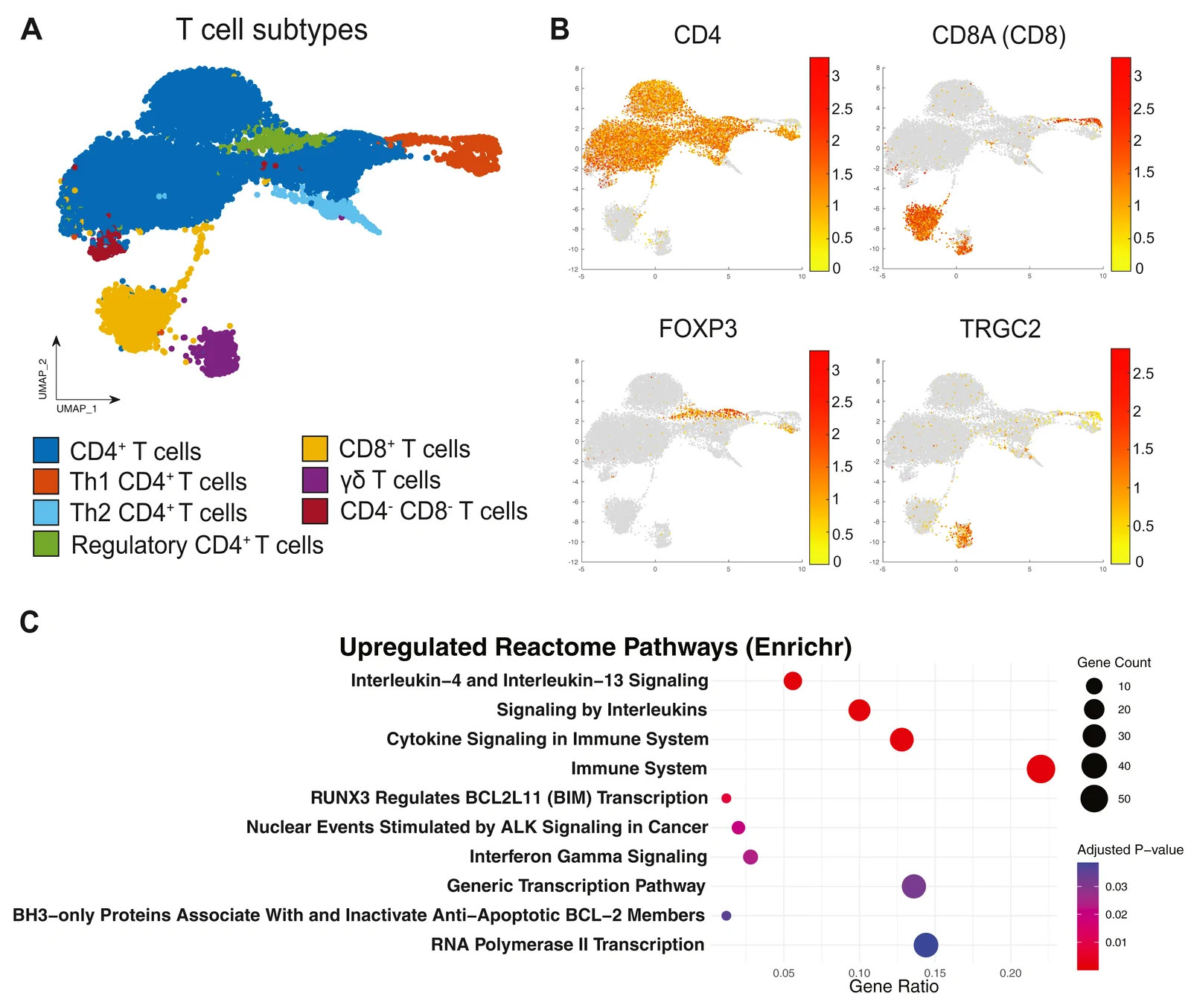
Human milk (*n =* 6) and formula-fed (*n =* 3) infant T cell subset analysis. (A) UMAP plot showing the subsets of T cells (CD4^+^ T cells, regulatory CD4^+^ T cells, Th1 CD4^+^ T cells, Th2 CD4^+^ T cells, CD8^+^ T cells, and gamma-delta (γδ) T cells). (B) T cell marker gene UMAPs. (C) The list of enriched pathways of upregulated differentially expressed genes in all T subsets of formula-fed infants as identified by Reactome pathway enrichment analysis using the online program Enrichr (ranked by adjusted *P* value). The circle color indicates the adjusted *P* value, and the size indicates the number of genes detected in the pathway. CD, cluster differentiation; Th2, T helper type 2 cells; UMAP, uniform manifold approximation and projection.

Overall, we found that the transcriptional profile of each peripheral immune cell subtype varied between the HMF and FF infants. Furthermore, transcriptional changes impacted across the monocyte, NK cell, B cell, and T cell subsets converged on common signaling and immunomodulatory pathways (Figure 6A, Supplemental Figure 2), including IL-4 and IL-13 signaling, cytokine signaling in the immune system, and signaling by ILs, all of which were upregulated in the FF infants (Figure 6B).

**FIGURE 6:**
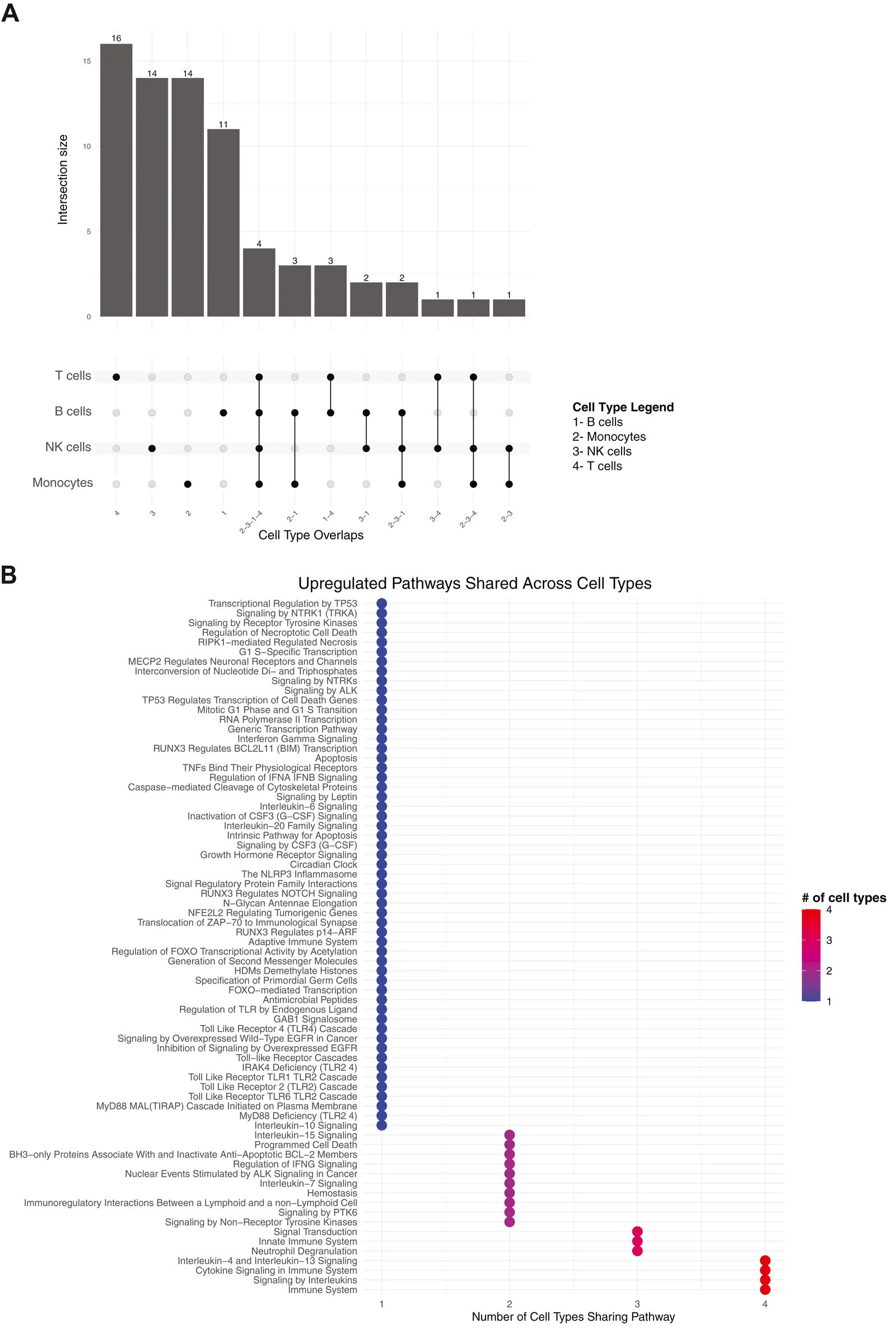
An overview of the top 25 upregulated pathways in peripheral immune cell subsets of formula-fed (*n =* 3) compared with human milk-fed infants (*n =* 6). (A) A ComplexUpSet plot depicting the pathways that monocytes, NK cells, B cells, and T cells have in common. (B) A dot plot showing the pathways identified by the ComplexUpSet plot. The circle color indicates the number of pathways shared among the immune cell subsets. NK, natural killer.

## Discussion

To our knowledge, this is the first study to comprehensively document the PBMC transcriptional phenotype of healthy HMF and FF infants at the single-cell level. Overall, PBMCs in infants were clustered into 6 major immune cell types. Monocytes were higher in FF than HMF infants and were enriched in several biological processes, such as activation, aggregation, chemotaxis, and extravasation. Also, the highest number of DEGs was associated with classical monocytes in FF infants. Previously, different monocyte subtypes were observed in children (aged 1–12 y) and adults (aged 20–40 y) with heterogeneous transcriptional changes in monocyte subtypes, whereas feeding mode was not considered in this study [21]. As neonates heavily depend on their innate immune responses for protection against infection, further characterizing the individual monocyte subset functions based on the type of infant feeding and age helps in elucidating the regulatory mechanisms of responses of distinct neonatal immune cell types. Interestingly, NK cells in FF infants exhibited higher levels of activation, chemotaxis, proliferation, and differentiation. Furthermore, FF infant NK cell subsets were enriched in almost the same immune-related pathways, suggesting transcriptomic responses with regard to infant feeding mode (human milk compared with formula) were similar across NK cell subsets. We speculate that FF infants’ high proportion of innate immune and elevated biological processes may reflect an immature/delayed immune response to exogenous stimuli. In contrast, this was not observed in HMF infants, suggesting human milk components optimize immune stimulation [7].

Studies have shown that total T cell numbers were different in human milk-fed compared with formula groups [7,8], whereas no difference was observed between HMF compared with FF groups in our study. It was previously shown that FF infants exhibited higher T cell activation than HMF infants [22]. For example, the expression of activation markers (alpha and beta integrins) on the surface of CD4^+^ and CD8^+^ T cells was higher in FF infants compared with HMF infants aged at 6 mo. Similarly, in this study, the enrichment of various T cell biological processes, such as activation, chemotaxis, proliferation, differentiation, exhaustion, and extravasation, was observed in FF infants. The variation in T cell functional status between the HMF and FF infants may be attributed to human milk components. For example, human milk IgA can limit gut bacteria translocation and, hence, decrease immune stimulation [23]. Human milk contains anti-inflammatory cytokines, such as transforming growth factor-β and IL-10, that downregulate lymphocyte proliferation [24]. Furthermore, lactoferrin in human milk blocks the production of the proinflammatory cytokines IL-1β, TNF-α, IL-6, and IL-5 [25], which can enhance the antigen-presenting capacity of immune cells [26].

Most previous studies have focused on the differential composition of PBMCs in HMF compared with FF infants. For example, it was reported that formula feeding resulted in a significant increase in naïve CD4^+^ T cells compared with HMF [8]. This is the first study to explore the gene expression profiles of the CD4^+^ and CD8^+^ T cell subsets in HMF and FF infants. Our data indicate that FF induces an upregulation of inflammatory pathways (IL-4 and IL-13 signaling, cytokine signaling in the immune system, and interferon signaling) in CD4^+^ and CD8^+^ T cell subsets. This suggests that human milk consumption may influence immune response by modifying the transcriptional profile of CD4^+^ and CD8^+^ T cells. Additional studies are needed to further probe the functional relevance of these gene-enriched pathways in terms of neonatal nutrition.

In this study, the functional status of B cells varied between HMF and FF infants. Biological processes such as activation, B cell receptor signaling, proliferation, and differentiation were enriched in B cells of FF infants. Specifically, upregulated DEGs in plasma B cells from FF infants were enriched in cytokine signaling pathways, for example, IL-4 and IL-13 signaling, IL-10 signaling, signaling by ILs, IL-20 family signaling, and IL-9 signaling. In contrast, DEGs in B cells of FF infants were enriched in inflammatory pathways, for example, IL-4 and IL-13 signaling, innate immune system, cytokine signaling in the immune system, signaling by ILs, and regulation of IFN-γ signaling. Taken together, B cell subsets exhibit distinct immunomodulatory characteristics associated with diet.

Interestingly, the upregulated DEGs in subsets within the innate and adaptive immune cell types of HMF infants were commonly enriched in signaling pathways, including IL-4 and IL-13, cytokine, and ILs. In addition, it has been reported that specific plasma cytokine concentrations (IL-1rα, IL-1α, IL-1β, IL-6, and TNF-α) are elevated in FF (containing only GOS) compared with breastfed infants [27]. In another study, the serum concentrations of TNF-α and IL-2 were higher in FF infants compared to the breastfed infants, throughout the first year of life [28]. IL-4 and 13 are key regulatory cytokines capable of modulating immune responsiveness triggered by either invading parasites or allergens [29]. Previous studies have also shown that FF infants have a distinct microbiota composition relative to breastfed infants, which can lead to an immune environment that favors the production of IL-4 and IL-13 [[30], [31], [32]], Additionally, higher levels of IL-4 and IL-13 (Th2 immune responses) in stimulated PBMCs to allergen house dust mite have been linked to an increased risk of developing allergic inflammation [33]. Therefore, the enrichment of the IL-4 and IL-13 signaling pathways in FF infants is consistent with an increased allergic sensitivity status in FF infants. Further elucidation of these signaling mechanisms will aid in the development of early dietary interventions.

Overall, the results of this study indicate that FF infants have a higher baseline innate and adaptive immune cell activation than HMF infants. Previously, in-vitro PBMC proliferation responsiveness of FF and human-milk-fed infants (aged at 3–9 mo) to various stimuli [phytohemagglutinin, pokeweed mitogen, *Staphylococcus aureus* (strain Cowan I), mixed lymphocyte reaction (allogeneic lymphocytes), and tetanus toxoid] was assessed [34]. The PBMC proliferative responsiveness to all stimuli was higher in the FF compared with the HMF group. The authors hypothesized that human milk components, such as IgA, were responsible for lower proliferative responses of PBMCs in HMF infants, protecting their immune systems from activation by external and endogenous stimuli. The current study’s findings add further support to previous reports that FF infants may have a heightened response to immune stimuli compared with HMF infants, consistent with the higher immune cell subset activation status in FF infants.

A strength of our study involves the collection of blood before complementary food introduction. Study limitations include a small sample size, a lack of human milk composition, and an insufficient number of cells for the detection of less-abundant immune cell types. Finally, cross-sectional studies have limitations, such as recall bias, as the study depends on the parent's recall of the feeding mode and confounding factors, such as socioeconomic status. Additionally, our differential gene expression analysis did not explicitly model subject-to-subject variability, which can influence the detection of consistently regulated genes across individuals. Although our use of stringent thresholds aimed to reduce potential false positives, future studies with broader scope may benefit from incorporating approaches such as pseudobulk aggregation or linear mixed models to more robustly account for intersubject variation.

In conclusion, transcriptional changes were cell-type specific; these changes converged on common immune-related pathways such as IL-4 and IL-13 signaling. Our results indicate that FF infants have a higher systemic baseline immune cell activation status, which may have short- and long-term consequences for infant health.

## Author contributions

The authors’ responsibilities were as follows – LY, SMD, RSC: conceived the overall study and supervised the research; BKM, MLS, LAD: performed experiments, and completed the data analysis; BKM, LY, MLS, RSC, SMD: contributed to literature search, data analyses and drafted the manuscript; and all authors: read and approved the final manuscript.

## Data availability

https://github.com/chapkinlab Single-cell RNA sequencing data described in the manuscript are publicly available in the Gene Expression Omnibus (GEO) repository accession GSE296678. The tool used for data analysis is available at https://github.com/jamesjcai/scGEAToolbox.

## Funding

This work was supported by USDA-ARS (6026-51000-012-000D) (LY), and USDA-ARS (55-3091-1-015) (LY, SMD, and RSC), Texas A&M Institute for Advancing Health Through Agriculture (RSC), at Texas A&M University, Texas A&M Hagler Institute for Advanced Study (SMD), Allen Endowed Chair in Nutrition & Chronic Disease Prevention (RSC) and the Cancer Prevention & Research Institute of Texas (RP230204) (MLS, JJC, and RSC).

## Conflict of interest

The authors report no conflicts of interest.
