# Supplementary material for: Single-cell transcriptomics reveals that human milk feeding shapes neonatal immune cell interleukin signaling pathways in a nonrandomized clinical trial
Source: Am J Clin Nutr. 2025 Apr 26;122(1):196–207. doi: 10.1016/j.ajcnut.2025.04.024 (PMC13639470; doi:10.1016/j.ajcnut.2025.04.024)
Supplement: Supplementary file 1 [file mmc1.docx]

**Single-cell transcriptomics reveals that human milk feeding shapes neonatal immune cell interleukin signaling pathways in a non-randomized clinical trial.**

Michael, Salinas and Bharath-Kumar, Mulakala et al.

**Supplementa1 Table 1.** Gene sets used for calculating UCell scores (Human milk (n=6) and formula fed (n=3) infants). The source for annotated gene sets for analysis is from Molecular Signatures Database (MSigDB) v2022.1.

| **Pathways** | | **Genes** |
| --- | --- | --- |
| Gobp_B_Cell_Activation | ABL1,ADA,ADAM17,ADGRG3,AHR,AICDA,AKAP17A,AKIRIN2,APLF,AQP8,ATAD5,ATM,BAD,BAK1,BANK1,BATF,BAX,BCL2,BCL3,BCL6,BLNK,BMI1,BST1,BST2,BTK,C17orf99,CARD11,CASP3,CASP8,CCR6,CD180,CD19,CD22,CD27,CD28,CD300A,CD320,CD38,CD40,CD40LG,CD70,CD74,CD79A,CD79B,CD81,CD86,CDH17,CDKN1A,CEBPG,CHRNA4,CHRNA7,CHRNB2,CLCF1,CMTM7,CR1,CR2,CTLA4,CTPS1,CXCR5,DCAF1,DCLRE1C,DLL1,DNAJB9,DOCK10,DOCK11,EP300,EPHB2,ERCC1,EXO1,EXOSC3,EXOSC6,EZH2,FCGR2B,FCRL1,FCRL3,FLT3,FNIP1,FOSL2,FOXJ1,FOXP3,FZD9,GAPT,GPR183,GPS2,HDAC4,HDAC5,HDAC9,HHEX,HMCES,HSPD1,ICOSLG,ID2,IFNA1,IFNA10,IFNA13,IFNA14,IFNA16,IFNA17,IFNA2,IFNA21,IFNA4,IFNA5,IFNA6,IFNA7,IFNA8,IFNB1,IFNE,IFNK,IFNW1,IGBP1,IKZF3,IL10,IL11,IL13,IL2,IL21,IL27RA,IL2RG,IL4,IL4I1,IL5,IL6,IL7,IL7R,IL9,INHA,INHBA,INPP5D,IRF2BP2,IRF8,IRS2,ITFG2,ITGA4,ITGB1,ITM2A,JAK3,KIT,KLF6,KMT5B,KMT5C,LAPTM5,LAT2,LAX1,LEF1,LFNG,LGALS1,LIG4,LRRC8A,LYL1,LYN,MAD2L2,MALT1,MEF2C,MFNG,MIF,MIR17HG,MIR185,MIR19A,MLH1,MMP14,MNDA,MS4A1,MSH2,MSH6,MZB1,NBN,NCKAP1L,NDFIP1,NFAM1,NFATC2,NFKBIZ,NHEJ1,NKX23,NOD2,NOTCH2,NSD2,NTRK1,ONECUT1,PARP3,PAWR,PAX5,PAXIP1,PCID2,PELI1,PHB1,PHB2,PHF14,PIK3CD,PIK3R1,PIK3R2,PIK3R3,PKN1,PLCG2,PLCL2,PMS2,POU2AF1,PPP2R3C,PRKCB,PRKCD,PRKDC,PRLR,PTK2B,PTPN2,PTPN6,PTPRC,PTPRJ,RABL3,RAG1,RAG2,RASGRP1,RBPJ,RC3H1,RIF1,RNF168,RNF8,SAMSN1,SANBR,SASH3,SFRP1,SH3KBP1,SHB,SHLD1,SHLD2,SHLD3,SKAP2,SLAMF8,SLC15A4,SLC25A5,SLC39A10,SLC39A7,SP3,SPI1,ST3GAL1,STAT5B,STAT6,SUPT6H,SWAP70,SYK,SYVN1,TBC1D10C,TBX21,TCF3,TCIRG1,TFRC,TGFB1,THEMIS2,THOC1,TICAM1,TIRAP,TLR4,TLR9,TNFAIP3,TNFRSF13B,TNFRSF13C,TNFRSF21,TNFRSF4,TNFSF13,TNFSF13B,TNFSF4,TNIP2,TOP2B,TP53,TP53BP1,TPD52,TRAF3IP2,TXLNA,TYROBP,UNG,VAV3,VCAM1,WNT3A,XBP1,YY1,ZAP70,ZBTB1,ZBTB7A,ZFP36L1,ZFP36L2 | |
| Gobp_B_Cell_Chemotaxis | CH25H,CXCL13,CYP7B1,GAS6,HSD3B7,PIK3CD,PTK2B,XCL1 | |
| Gobp_B_Cell_Differentiation | ABL1,ADA,ADAM17,ADGRG3,AICDA,AQP8,ATM,BAD,BAK1,BAX,BCL2,BCL3,BCL6,BLNK,BTK,C17orf99,CARD11,CD19,CD27,CD40LG,CD79A,CD79B,CDH17,CEBPG,CLCF1,CMTM7,CR1,CR2,DCAF1,DCLRE1C,DLL1,DNAJB9,DOCK10,DOCK11,EP300,EZH2,FCGR2B,FCRL3,FLT3,FNIP1,FOSL2,FZD9,GPR183,GPS2,HDAC4,HDAC5,HDAC9,HHEX,ID2,IFNA1,IFNA10,IFNA13,IFNA14,IFNA16,IFNA17,IFNA2,IFNA21,IFNA4,IFNA5,IFNA6,IFNA7,IFNA8,IFNB1,IFNE,IFNK,IFNW1,IKZF3,IL10,IL11,IL2,IL21,IL2RG,IL4,IL4I1,IL6,IL7,IL9,INHA,INHBA,INPP5D,IRF2BP2,IRF8,ITFG2,ITGA4,ITGB1,ITM2A,JAK3,KIT,KLF6,LFNG,LGALS1,LRRC8A,LYL1,MALT1,MFNG,MIR17HG,MMP14,MS4A1,MSH2,NCKAP1L,NFAM1,NFKBIZ,NHEJ1,NKX2-3,NOTCH2,NTRK1,ONECUT1,PAX5,PCID2,PHF14,PIK3CD,PIK3R1,PIK3R2,PIK3R3,PLCG2,PLCL2,POU2AF1,PPP2R3C,PRKDC,PTK2B,PTPN2,PTPN6,PTPRC,PTPRJ,RABL3,RAG1,RAG2,RBPJ,SFRP1,SLAMF8,SLC25A5,SLC39A7,SP3,SPI1,ST3GAL1,STAT5B,SYK,SYVN1,TCF3,TCIRG1,TLR9,TNFSF13B,TOP2B,TP53,TPD52,TRAF3IP2,VCAM1,XBP1,YY1,ZBTB1,ZBTB7A,ZFP36L1,ZFP36L2 | |
| Gobp_B_Cell_Mediated_Immunity | AICDA,APLF,ATAD5,BATF,BCL10,BCL3,BCL6,BTK,C17orf99,C1QA,C1QB,C1QBP,C1QC,C1R,C1RL,C1S,C2,C3,C4A,C4B,C4BPA,C4BPB,C5,C6,C7,C8A,C8B,C8G,C9,CARD9,CCR6,CD19,CD226,CD27,CD28,CD40,CD40LG,CD46,CD55,CD70,CD74,CD81,CFI,CLCF1,CLU,CR1,CR1L,CR2,CSF2RB,ERCC1,EXO1,EXOSC3,EXOSC6,FCER1A,FCER1G,FCER2,FCGR1A,FCGR2B,FCGR3A,FOXJ1,FOXP3,GAPT,HLA-E,HLA-G,HMCES,HPX,HSPD1,IGHA1,IGHA2,IGHD,IGHE,IGHG1,IGHG2,IGHG3,IGHG4,IGHM,IGHV1-18,IGHV1-24,IGHV1-3,IGHV1-45,IGHV1-58,IGHV1-69,IGHV1-69-2,IGHV1-69D,IGHV2-26,IGHV2-5,IGHV2-70,IGHV2-70D,IGHV3-11,IGHV3-13,IGHV3-15,IGHV3-16,IGHV3-20,IGHV3-21,IGHV3-23,IGHV3-30,IGHV3-33,IGHV3-35,IGHV3-38,IGHV3-43,IGHV3-48,IGHV3-49,IGHV3-53,IGHV3-64,IGHV3-64D,IGHV3-66,IGHV3-7,IGHV3-72,IGHV3-73,IGHV3-74,IGHV4-28,IGHV4-31,IGHV4-34,IGHV4-39,IGHV4-4,IGHV4-59,IGHV4-61,IGHV5-10-1,IGHV5-51,IGHV6-1,IGHV7-4-1,IGHV7-81,IGHV8511,IGKC,IGLC1,IGLC2,IGLC3,IGLC6,IGLC7,IGLL1,IGLL5,IL10,IL13RA2,IL2,IL21R,IL27RA,IL2RB,IL4,IL4R,IL9,IL9R,INPP5D,IRF7,KMT5B,KMT5C,LIG4,LTA,MAD2L2,MASP2,MBL2,MLH1,MPL,MSH2,MSH6,MYD88,NBN,NCR3LG1,NDFIP1,NECTIN2,NFKBIZ,NOD2,NSD2,PARP3,PAXIP1,PMS2,PRKCD,PTPN6,PTPRC,RIF1,RNF168,RNF8,SANBR,SERPING1,SHLD1,SHLD2,SHLD3,SLA2,SLC15A4,STAT6,SUPT6H,SUSD4,SVEP1,SWAP70,TBX21,TCIRG1,TFRC,TGFB1,THOC1,TLR8,TNF,TNFSF13,TNFSF4,TP53BP1,TRAF3IP2,TREM2,TREX1,UNG,XCL1,ZP3 | |
| Gobp_B_Cell_Proliferation | ABL1,ADA,AHR,ATAD5,ATM,BAX,BCL2,BCL6,BMI1,BST1,BTK,CARD11,CASP3,CD180,CD19,CD22,CD300A,CD320,CD38,CD40,CD40LG,CD70,CD74,CD79A,CD81,CDKN1A,CHRNB2,CLCF1,CR2,CTLA4,CTPS1,EPHB2,FCGR2B,FCRL3,FOSL2,GAPT,GPR183,HSPD1,IFNA1,IFNA10,IFNA13,IFNA14,IFNA16,IFNA17,IFNA2,IFNA21,IFNA4,IFNA5,IFNA6,IFNA7,IFNA8,IFNB1,IFNE,IFNK,IFNW1,IKZF3,IL10,IL13,IL2,IL21,IL4,IL5,IL7,IL7R,IL9,INPP5D,IRS2,LEF1,LYN,MEF2C,MIF,MIR185,MNDA,MS4A1,MZB1,NCKAP1L,NFATC2,NFKBIZ,PAWR,PELI1,PKN1,PLCL2,PRKCD,PRLR,PTPRC,RAG2,RASGRP1,RC3H1,SASH3,SHB,SLC39A10,TFRC,TICAM1,TIRAP,TLR4,TLR9,TNFRSF13B,TNFRSF13C,TNFRSF21,TNFRSF4,TNFSF13B,TYROBP,VAV3,WNT3A | |
| Gobp_B_Cell_Receptor_Signaling_Pathway | ABL1,BANK1,BAX,BCAR1,BCL2,BLK,BLNK,BMX,BTK,CD19,CD22,CD300A,CD38,CD79A,CD79B,CD81,CMTM3,CTLA4,FCGR2B,FCRL3,FOXP1,GCSAM,GCSAML,GPS2,GRB2,IGHA1,IGHA2,IGHD,IGHE,IGHG1,IGHG2,IGHG3,IGHG4,IGHM,IGKC,IGLC1,IGLC3,IGLC6,IGLC7,ITK,KLHL6,LAT2,LCK,LIME1,LPXN,LYN,MAPK1,MEF2C,MIR18A,MIR19A,MIR34A,MNDA,MS4A1,NCKAP1L,NFAM1,NFATC2,NFKB1,NFKBIA,NFKBIZ,PIK3CD,PLCG2,PLCL2,PLEKHA1,PRKCB,PRKCH,PTPN22,PTPN6,PTPRC,RFTN1,SH2B2,SLC39A10,SOS1,STAP1,SYK,TEC,TMIGD2,VAV3 | |
| Gobp_Interleukin_13_Production | ARG2,GATA3,HLAE,IFNA2,IFNL1,IL17RA,IL18,IL1RAP,IL33,IL4,IRF4,LEF1,LGALS9,LILRA5,PRKCZ,RARA,SCGB1A1,SPHK2,TNFRSF21,TNFSF4,TSLP | |
| Gobp_Interleukin_4_Production | CD28,CD3E,CD40LG,CD83,CD86,CEBPB,CLECL1P,DDIT3,EPX,FCER1G,FOXP3,GATA3,HAVCR2,HLA-DRB1,HLAE,IL1RAP,IL20RB,IL33,IRF4,LEF1,LGALS9,MIR320A,NDFIP1,NLRP3,PRG2,PRKCQ,PRKCZ,RARA,SASH3,SCGB1A1,SLC7A5,SYK,TNFSF4,ZFPM1,ZP3 | |
| Gobp_Monocyte_Activation | ADAM10,ADAM9,AZU1,CD33,CSF1,DYSF,FN1,FOXP1,HYAL2,LILRB4,MT1G,SPACA3 | |
| Gobp_Monocyte_Aggregation | BMP7,CD44,HAS2,IL1B,NR4A3 | |
| Gobp_Monocyte_Chemotactic_Protein_1_Production | ADIPOQ,AGER,APOD,C1QTNF3,CD84,CLEC7A,ERBIN,GSTP1,IL1B,LGALS9,MCOLN2,MIR92A1,NOD2,NR1H4,OAS1,OAS3,SELENOK,SOCS5,SYK,TRPV4,TWIST1 | |
| Gobp_Monocyte_Chemotaxis | AIF1,ANO6,ANXA1,CALCA,CCL1,CCL11,CCL13,CCL14,CCL15,CCL16,CCL17,CCL18,CCL19,CCL2,CCL20,CCL21,CCL22,CCL23,CCL24,CCL25,CCL26,CCL3,CCL3L1,CCL3L3,CCL4,CCL5,CCL7,CCL8,CCN3,CCR1,CCR2,CREB3,CTSG,CX3CL1,CX3CR1,CXCL10,CXCL12,CXCL17,DEFB104A,DEFB104B,DEFB124,DUSP1,FLT1,FOLR2,FPR2,GREM1,HMGB1,IL6,IL6R,LGALS3,LGMN,LYN,MICOS10-NBL1,MOSPD2,MSMP,NBL1,PDGFB,PLA2G7,PTPRO,RPS19,S100A12,S100A14,S100A7,SERPINE1,SLAMF8,SLIT2,TNFRSF11A,TNFSF11,TNFSF18,XCL1,XCL2 | |
| Gobp_Monocyte_Differentiation | ACIN1,APCS,BMP4,CD4,CD74,CDK6,CSF1,CSF1R,CSF2,CTNNBIP1,DCSTAMP,FASN,FES,FOXP1,GPR68,HLADRB1,HOXA7,IFI16,IL31RA,IL34,INPP5D,IRF7,MED1,MIR125B1,MT1G,MYC,MYH9,PDE1B,PIR,PPARG,SP3,THOC5,VEGFA,ZBTB46,ZFP36L1 | |
| Gobp_Monocyte_Extravasation | AGER,CCR2,CD47,JAM3,JAML,MIR146A,PDGFD,PECAM1,PLCB1,SIRPA | |
| Gobp_Natural_Killer_Cell_Activation | AP1G1,AXL,BAG6,BLOC1S3,BLOC1S6,CASP8,CD2,CD244,CLNK,CORO1A,DCAF15,ELF4,EMP2,FCGR3A,FGR,GAS6,GATA3,HAVCR2,HECTD1,HLA-E,HLAF,IFNA1,IFNA10,IFNA13,IFNA14,IFNA16,IFNA17,IFNA2,IFNA21,IFNA4,IFNA5,IFNA6,IFNA7,IFNA8,IFNB1,IFNE,IFNK,IFNW1,IL12A,IL12B,IL15,IL15RA,IL18,IL18R1,IL2,IL21R,IL23A,IL23R,JAK2,KAT7,KIR3DS1,KLRC1,KLRC2,KLRC3,KLRC4,KLRK1,KLRD1,KLRF2,KLRK1,LAMP1,LEP,MERTK,MICA,NCR1,NCR3,NFIL3,NKG7,PBX1,PGLYRP1,PGLYRP2,PGLYRP3,PIBF1,PIK3CD,PRDM1,PRDX1,PTPN22,PTPRC,RAB27A,RABL3,RASGRP1,RHBDD3,SLAMF1,SLAMF7,SNX27,SP3,STAT5B,TICAM1,TICAM2,TOX,TUSC2,TYK2,TYRO3,TYROBP,ULBP1,ULBP2,ULBP3,UNC13D,VAMP2,VAMP7,ZBTB1,ZNF683 | |
| Gobp_Natural_Killer_Cell_Chemotaxis | CCL2,CCL3,CCL4,CCL5,CCL7,KLRC4-KLRK1,KLRK1,PIK3CD,PIK3CG,XCL1 | |
| Gobp_Natural_Killer_Cell_Cytokine_Production | CD160,CD226,CD96,CLNK,HLA-E,HLA-F,HLA-G,KIR2DL4,RAET1G | |
| Gobp_Natural_Killer_Cell_Degranulation | AP1G1,CORO1A,FCGR3A,HLA-F,KLRC2,KLRF2,LAMP1,NKG7,RAB27A,UNC13D,VAMP2,VAMP7 | |
| Gobp_Natural_Killer_Cell_Differentiation | AXL,GAS6,HECTD1,IL15,IL15RA,KAT7,MERTK,NFIL3,PBX1,PGLYRP1,PGLYRP2,PGLYRP3,PIK3CD,PRDM1,PTPRC,RABL3,RASGRP1,SLAMF1,SP3,STAT5B,TOX,TUSC2,TYRO3,ZBTB1,ZNF683 | |
| Gobp_Natural_Killer_Cell_Proliferation | ELF4,EMP2,FCGR3A,HLA-E,IL12B,IL15,IL18,IL23A,IL23R,JAK2,LEP,PTPN22,SLAMF1,STAT5B,TYK2 | |
| Gobp_T_Cell_Activation | ABL1,ABL2,ACTB,ACTL6A,ACTL6B,ADA,ADAM17,ADAM8,ADORA2A,AGER,AIF1,AIRE,AKT1,AMBRA1,ANXA1,AP3B1,AP3D1,APBB1IP,ARG1,ARG2,ARID1A,ARID1B,ARID2,ARMC5,ASCL2,ATF2,ATG5,ATP7A,AZI2,B2M,BAD,BATF,BAX,BCL10,BCL11B,BCL2,BCL3,BCL6,BID,BMI1,BMP4,BRAF,BRD7,BTN2A2,BTN3A1,CADM1,CAMK4,CARD11,CASP3,CASP8,CAV1,CBFB,CBLB,CCDC88B,CCL19,CCL2,CCL21,CCL5,CCND3,CCR2,CCR6,CCR7,CCR9,CD151,CD160,CD1C,CD1D,CD2,CD209,CD24,CD247,CD27,CD274,CD276,CD28,CD300A,CD3D,CD3E,CD3G,CD4,CD40LG,CD44,CD46,CD47,CD5,CD55,CD6,CD7,CD70,CD74,CD80,CD81,CD83,CD84,CD86,CD8A,CD8B,CDH26,CDK6,CEACAM1,CEACAM21,CEBPB,CGAS,CHD7,CHRNA7,CLC,CLEC4A,CLEC4D,CLEC4E,CLEC4G,CLEC7A,CLECL1P,CLPTM1,CORO1A,CR1,CRTAM,CSK,CTLA4,CTNNB1,CTPS1,CTSG,CTSL,CUL4A,CXADR,CYP26B1,CYRIB,DAPL1,DCAF12,DDOST,DHPS,DLG1,DLG5,DLL4,DNAJA3,DOCK2,DOCK8,DPP4,DROSHA,DTX1,DUSP10,DUSP22,DUSP3,EBI3,EFNB1,EFNB2,EFNB3,EGR1,EGR3,EIF2AK4,ELF4,ENTPD7,EOMES,EPO,ERBB2,F2RL1,FADD,FANCA,FANCD2,FCER1G,FCGR2B,FCHO1,FGL1,FGL2,FKBP1A,FKBP1B,FLOT2,FOSL2,FOXJ1,FOXN1,FOXO3,FOXP1,FOXP3,FUT7,FYN,FZD5,FZD7,FZD8,GATA3,GBA1,GLI2,GLI3,GLMN,GPAM,GPNMB,GPR18,GPR183,GPR89A,GPR89B,GSN,HAVCR2,HES1,HFE,HHLA2,HLA-A,HLA-DMA,HLADMB,HLA-DOA,HLA-DOB,HLA-DPA1,HLA-DPB1,HLA-DQA1,HLA-DQA2,HLA-DQB1,HLADQB2,HLADRA,HLADRB1,HLADRB3,HLDRB4,HLADRB5,HLAE,HLAG,HLX,HMGB1,HSH2D,HSPD1,HSPH1,ICAM1,ICOS,ICOSLG,IDO1,IFNA1,IFNA10,IFNA13,IFNA14,IFNA16,IFNA17,IFNA2,IFNA21,IFNA4,IFNA5,IFNA6,IFNA7,IFNA8,IFNB1,IFNE,IFNG,IFNK,IFNL1,IFNW1,IGF1,IGF2,IGFBP2,IHH,IKZF3,IL10,IL12A,IL12B,IL12RB1,IL15,IL18,IL18R1,IL1A,IL1B,IL1RL2,IL2,IL20RB,IL21,IL23A,IL23R,IL27,IL2RA,IL2RG,IL36B,IL4,IL4I1,IL4R,IL6,IL6R,IL6ST,IL7,IL7R,ILDR2,INS,IRF1,IRF4,ITCH,ITGAL,ITK,ITPKB,JAG2,JAK1,JAK2,JAK3,JAML,JMJD6,JUNB,KAT2A,KAT5,KDELR1,KIF13B,KIT,KITLG,KLHL25,KLRC1,KLRC4-KLRK1,KLRK1,KMT2A,LAG3,LAPTM5,LAT,LAX1,LCK,LCP1,LEF1,LEP,LEPR,LFNG,LGALS1,LGALS3,LGALS9,LGALS9B,LGALS9C,LIG4,LILRB1,LILRB2,LILRB4,LIPA,LMBR1L,LMO1,LOXL3,LRRC32,LY9,LYN,MAD1L1,MAFB,MALT1,MAP3K8,MAPK8IP1,MARCHF7,MDK,MEN1,METTL3,MFAP3,MICA,MICB,MIR181C,MIR21,MIR27A,MIR30B,MR1,MSN,MTOR,MYB,MYH9,NCAPH2,NCK1,NCK2,NCKAP1L,NCSTN,NDFIP1,NFKBID,NFKBIZ,NHEJ1,NKAP,NKG7,NKX23,NLRC3,NLRP3,NOD2,NRARP,P2RX7,PAG1,PATZ1,PAWR,PBRM1,PCK1,PDCD1LG2,PDE5A,PDPK1,PELI1,PHF10,PIK3CA,PIK3CD,PIK3CG,PIK3R1,PIK3R2,PIK3R3,PIK3R6,PKNOX1,PLA2G2A,PLA2G2D,PLA2G2E,PLA2G2F,PLA2G5,PNP,PPP3CA,PPP3CB,PRDM1,PRDX2,PRELID1,PREX1,PRKAR1A,PRKCQ,PRKCZ,PRKDC,PRNP,PRR7,PSEN1,PSG9,PSMB10,PSMB11,PTGER4,PTPN11,PTPN2,PTPN22,PTPN6,PTPRC,PYCARD,RAB27A,RAB29,RABL3,RAC2,RAG1,RAG2,RARA,RASAL3,RASGRP1,RBX1,RC3H1,RC3H2,RELB,RHOA,RHOH,RIPK2,RIPK3,RIPOR2,RORA,RORC,RPL22,RPS3,RPS6,RSAD2,RUNX1,RUNX2,RUNX3,SART1,SASH3,SCGB1A1,SCRIB,SDC4,SELENOK,SEMA4A,SFTPD,SH2D2A,SH3RF1,SHB,SHH,SIRPA,SIRPB1,SIRPG,SIT1,SLA2,SLAMF1,SLAMF6,SLAMF7,SLAMF9,SLC11A1,SLC46A2,SLC4A2,SLC7A1,SMAD3,SMAD7,SMARCA2,SMARCA4,SMARCB1,SMARCC1,SMARCC2,SMARCD1,SMARCD2,SMARCD3,SMARCE1,SOCS1,SOCS3,SOCS5,SOCS6,SOD1,SOS1,SOS2,SOX12,SOX13,SOX4,SP3,SPI1,SPINK5,SPN,SPTA1,SRC,SRF,STAT3,STAT4,STAT5B,STAT6,STK11,STOML2,SYK,TARM1,TBX21,TCF7,TCIRG1,TESPA1,TFRC,TGFB1,TGFBR2,THEMIS,THY1,TIGIT,TMEM131L,TMEM98,TMIGD2,TNFAIP8L2,TNFRSF13C,TNFRSF14,TNFRSF1B,TNFRSF21,TNFRSF4,TNFRSF9,TNFSF11,TNFSF13B,TNFSF14,TNFSF18,TNFSF4,TNFSF8,TNFSF9,TOX,TP53,TRAC,TRAF3IP2,TRAF6,TRBC1,TRBC2,TRDC,TREML2,TREX1,TRGC1,TSC1,TSPAN32,TWSG1,TYK2,USP44,VAV1,VCAM1,VNN1,VSIG4,VSIR,VTCN1,WAS,WDFY4,WNT1,WNT10B,WNT4,XBP1,XCL1,YES1,ZAP70,ZBTB1,ZBTB16,ZBTB7B,ZC3H12A,ZC3H8,ZEB1,ZFP36L1,ZFP36L2,ZFPM1,ZMIZ1,ZNF683,ZP3,ZP4 | |
| Gobp_T_Cell_Chemotaxis | ADAM10,ADAM17,CCL21,CCL26,CCL3,CCL5,CCR2,CXCL10,CXCL11,CXCL13,CXCL16,DEFA1,DEFA1B,GPR183,OXSR1,PIK3CD,PIK3CG,PLEC,S100A7,SLC12A2,STK39,TMEM102,TNFSF14,WNK1,WNT5A,XCL1,XCL2 | |
| Gobp_T_Cell_Differentiation | ABL1,ACTB,ACTL6A,ACTL6B,ADA,ADAM17,ADAM8,AIRE,AMBRA1,ANXA1,AP3B1,AP3D1,ARID1A,ARID1B,ARID2,ARMC5,ASCL2,ATF2,ATG5,ATP7A,B2M,BAD,BATF,BCL11B,BCL2,BCL3,BCL6,BMI1,BMP4,BRAF,BRD7,BTN2A2,CAMK4,CARD11,CBFB,CCL19,CCR2,CCR6,CCR7,CCR9,CD1D,CD2,CD27,CD28,CD3D,CD3E,CD3G,CD4,CD46,CD74,CD80,CD83,CD86,CD8A,CDK6,CHD7,CLEC4D,CLEC4E,CLEC4G,CLPTM1,CR1,CRTAM,CTLA4,CTNNB1,CTSL,CYP26B1,DLL4,DNAJA3,DOCK2,DROSHA,DTX1,DUSP10,EGR1,EGR3,ENTPD7,EOMES,ERBB2,FADD,FANCA,FANCD2,FCER1G,FGL2,FOSL2,FOXJ1,FOXN1,FOXO3,FOXP1,FOXP3,FUT7,FZD5,FZD7,FZD8,GATA3,GBA1,GLI2,GLI3,GPR18,GPR183,GPR89A,GPR89B,HLADOA,HLADRA,HLADRB1,HLAG,HLX,HMGB1,IFNA2,IFNB1,IFNG,IFNL1,IHH,IKZF3,IL12B,IL12RB1,IL15,IL18,IL18R1,IL1A,IL1B,IL1RL2,IL2,IL21,IL23A,IL23R,IL27,IL2RA,IL2RG,IL36B,IL4,IL4I1,IL4R,IL6,IL6R,IL7,IL7R,IRF1,IRF4,ITK,ITPKB,JAG2,JAK1,JAK3,JMJD6,JUNB,KAT2A,KAT5,KDELR1,KIT,KLHL25,KLRC1,KMT2A,LAG3,LCK,LEF1,LEP,LEPR,LFNG,LGALS9,LIG4,LILRB2,LILRB4,LIPA,LMBR1L,LOXL3,LY9,MAFB,MALT1,MDK,MEN1,METTL3,MIR21,MIR30B,MR1,MTOR,MYB,NCAPH2,NCKAP1L,NFKBID,NFKBIZ,NHEJ1,NKAP,NKX23,NLRP3,NRARP,PATZ1,PBRM1,PCK1,PHF10,PIK3CD,PIK3R1,PIK3R2,PIK3R3,PIK3R6,PKNOX1,PLA2G2D,PNP,PPP3CB,PRDM1,PRDX2,PRELID1,PREX1,PRKCZ,PRKDC,PRR7,PSG9,PSMB11,PTGER4,PTPN2,PTPN22,PTPRC,RABL3,RAG1,RAG2,RARA,RASGRP1,RC3H1,RC3H2,RELB,RHOA,RHOH,RIPK2,RIPK3,RORA,RORC,RPL22,RPS6,RSAD2,RUNX1,RUNX2,RUNX3,SART1,SASH3,SEMA4A,SH3RF1,SHB,SHH,SLAMF6,SLC46A2,SLC4A2,SMAD7,SMARCA2,SMARCA4,SMARCB1,SMARCC1,SMARCC2,SMARCD1,SMARCD2,SMARCD3,SMARCE1,SOCS1,SOCS3,SOCS5,SOD1,SOS1,SOS2,SOX12,SOX13,SOX4,SP3,SPI1,SPINK5,SPN,SRF,STAT3,STAT4,STAT5B,STAT6,STK11,SYK,TBX21,TCF7,TCIRG1,TESPA1,TGFB1,TGFBR2,THEMIS,TMEM131L,TMEM98,TNFRSF9,TNFSF18,TNFSF4,TNFSF8,TNFSF9,TOX,TP53,TRAF3IP2,TSC1,USP44,VAV1,VNN1,VSIR,WNT1,WNT10B,WNT4,XBP1,ZAP70,ZBTB1,ZBTB16,ZBTB7B,ZC3H12A,ZC3H8,ZEB1,ZFP36L1,ZFP36L2,ZFPM1,ZMIZ1,ZNF683 | |
| Gobp_T_Cell_Extravasation | CCL2,CCR2,CD99,CD99L2,CRK,CRKL,F11R,FADD,ICAM1,IL27RA,ITGAL,RIPK3,XG | |
| Gobp_T_Cell_Proliferation | ABL1,AGER,AIF1,ANXA1,ARG1,ARG2,ARMC5,BAX,BCL6,BID,BMI1,BMP4,BTN2A2,BTN3A1,CADM1,CARD11,CASP3,CBLB,CCDC88B,CCL19,CCL5,CCND3,CCR2,CD151,CD1D,CD209,CD24,CD274,CD276,CD28,CD3E,CD40LG,CD46,CD55,CD6,CD70,CD80,CD81,CD86,CEBPB,CLC,CLEC4G,CLECL1P,CORO1A,CR1,CRTAM,CTLA4,CTNNB1,CTPS1,DHPS,DLG1,DLG5,DNAJA3,DOCK2,DOCK8,EBI3,EFNB1,ELF4,EPO,ERBB2,FADD,FKBP1B,FOXJ1,FOXP3,FYN,GLMN,GPAM,GPNMB,HAVCR2,HES1,HHLA2,HLAA,HLADMB,HLADPA1,HLADPB1,HLADRB1,HLAE,HLAG,HMGB1,ICOSLG,IDO1,IGF1,IGF2,IGFBP2,IHH,IL10,IL12B,IL12RB1,IL15,IL18,IL1A,IL1B,IL2,IL20RB,IL21,IL23A,IL23R,IL27,IL2RA,IL4,IL4I1,IL6,IL6ST,IRF1,ITCH,JAK2,KITLG,LEP,LGALS3,LGALS9,LGALS9B,LGALS9C,LILRB1,LILRB2,LILRB4,LIPA,LMBR1L,LMO1,LRRC32,MAD1L1,MALT1,MAPK8IP1,MARCHF7,MIR181C,MIR21,MIR30B,MSN,NCK1,NCK2,NCKAP1L,NCSTN,NDFIP1,P2RX7,PAWR,PDCD1LG2,PDE5A,PELI1,PIK3CG,PLA2G2A,PLA2G2D,PLA2G2E,PLA2G2F,PLA2G5,PNP,PPP3CA,PPP3CB,PRDX2,PRKAR1A,PRKCQ,PRNP,PSMB10,PTPN22,PTPN6,PTPRC,PYCARD,RAC2,RASAL3,RASGRP1,RC3H1,RC3H2,RIPK2,RIPK3,RPS3,RPS6,SASH3,SCGB1A1,SCRIB,SDC4,SELENOK,SFTPD,SH2D2A,SH3RF1,SHH,SLAMF1,SLC11A1,SLC4A2,SLC7A1,SOS1,SOS2,SPN,SPTA1,STAT5B,SYK,TFRC,TGFBR2,TMEM131L,TMIGD2,TNFRSF13C,TNFRSF14,TNFRSF1B,TNFRSF21,TNFRSF4,TNFRSF9,TNFSF13B,TNFSF14,TNFSF18,TNFSF4,TNFSF8,TNFSF9,TP53,TRAF6,TSPAN32,TWSG1,TYK2,VCAM1,VSIG4,VSIR,VTCN1,WNT4,XCL1,ZAP70,ZBTB7B,ZP3,ZP4 | |
| Custom_T_Cell_Exhaustion | CD160,CD244,CTLA4,HAVCR2,LAG3,PDCD1,TIGIT | |

**Supplemental Table 2.** Marker genes used to identify the subsets of innate immune cells and adaptive immune cells in the PBMCs of human milk fed and formula fed infants by scRNAseq.

| **Cell type** | **Marker gene(s)** |
| --- | --- |
| Monocytes | CD14, LYZ, CSF1R |
| Classical monocytes | CD14 |
| Non-classical monocytes | FCGR3A, FCGR3B |
| Natural killer (NK) cells | NCAM1, NKG7, NCR1 |
| CD56^+^ CD16^-^ NK cells | NCAM1 |
| CD56^-^ CD16^+^ NK cells | FCGR3A, FCGR3B |
| Proliferating CD56^-^ CD16^+^ NK cells | MKI67, STMN1 |
| NKT-like cells | CD3D, NKG7 |
| B cells | CD19, MS4A1, CD79A, CD79B |
| B cells | CD19, MS4A1, |
| Plasma B cells | TNFRSF17, JCHAIN |
| T cells | CD3D, CD3E, CD3G, TRAC, IL7R |
| CD4^+^ T cells | CD4 |
| Th1 T cells | TBX21, IFNG |
| Th2 T cells | GATA3, CCR4 |
| Regulatory T cells | FOXP3, IL2RA |
| CD8^+^ T cells | CD8A, CD8B2 |
| γδ T cells | TRDC, TRGC1, TRGC2 |

**Supplemental Table 3.** The list of differentially expressed genes in monocyte subsets of FF (n=3) and HMF (n=6) infants.

| **Gene** | **log2FC^1^** | **avg_FF^2^** | **avg_HMF^3^** | **pct_1_FF^4^** | **pct_2_HMF^5^** | **p_val_adj^6^** |
| --- | --- | --- | --- | --- | --- | --- |
| **Classical monocytes** | |  |  |  |  |  |
| SIGLEC14 | 3.227618 | 1.175407 | 0.125481 | 0.85775 | 0.104803 | 0 |
| MT-ND2 | 2.094633 | 1.963731 | 0.459764 | 0.971726 | 0.283843 | 0 |
| DDX3Y | 2.936011 | 1.22674 | 0.160297 | 0.785819 | 0.104803 | 1.5E-306 |
| SLC11A1 | 0.443677 | 2.418437 | 1.778176 | 0.990771 | 0.937991 | 1.5E-202 |
| SOCS3 | 0.599422 | 2.7206 | 1.795646 | 0.980076 | 0.855022 | 2.7E-186 |
| SLC2A3 | 0.761387 | 2.016177 | 1.189402 | 0.939643 | 0.748472 | 1E-177 |
| AQP9 | 1.456141 | 1.261968 | 0.459945 | 0.78157 | 0.374672 | 3.9E-174 |
| LILRB1 | 0.921264 | 1.515533 | 0.800272 | 0.895253 | 0.659389 | 1.2E-163 |
| ACSL1 | 0.696973 | 1.841232 | 1.135792 | 0.940229 | 0.772926 | 1.1E-150 |
| CEBPB | 0.398245 | 2.350952 | 1.783858 | 0.987548 | 0.930131 | 1.7E-147 |
| GNG10 | 0.816315 | 1.36549 | 0.775449 | 0.884413 | 0.660262 | 2.7E-146 |
| MCTP2 | 3.411063 | 0.54924 | 0.051633 | 0.474363 | 0.055022 | 1E-144 |
| FKBP5 | 0.929476 | 1.178157 | 0.61859 | 0.852183 | 0.575546 | 5E-143 |
| THBS1 | 0.897745 | 2.625735 | 1.409298 | 0.883241 | 0.623581 | 1.5E-137 |
| PHC2 | 0.680031 | 1.465997 | 0.915005 | 0.914591 | 0.738865 | 2E-132 |
| GCA | 0.468485 | 2.111001 | 1.525669 | 0.967184 | 0.892576 | 2E-130 |
| PROK2 | 1.674649 | 1.09209 | 0.342089 | 0.646499 | 0.265502 | 2.4E-125 |
| PLSCR1 | 0.708339 | 1.346314 | 0.823977 | 0.899355 | 0.689956 | 2.6E-123 |
| FPR1 | 0.365534 | 2.378065 | 1.84581 | 0.972605 | 0.905677 | 6.3E-121 |
| UTY | 2.722685 | 0.410538 | 0.062193 | 0.448725 | 0.063755 | 5.1E-120 |
| SBNO2 | 0.59346 | 1.448839 | 0.960221 | 0.929827 | 0.769432 | 6.7E-119 |
| S100A12 | 0.522211 | 2.550578 | 1.775978 | 0.929974 | 0.857642 | 4.1E-110 |
| JAK3 | 0.859209 | 1.09357 | 0.602836 | 0.819514 | 0.558952 | 3.9E-109 |
| CR1 | 1.00958 | 1.054359 | 0.52369 | 0.763112 | 0.480349 | 4.6E-107 |
| PER1 | 0.392756 | 2.111811 | 1.608509 | 0.976414 | 0.88559 | 1.9E-104 |
| MCEMP1 | 0.885221 | 1.313292 | 0.711023 | 0.790653 | 0.569432 | 5.4E-103 |
| CD300E | 0.428198 | 2.140387 | 1.590713 | 0.967624 | 0.866376 | 2.3E-101 |
| AGFG1 | 0.632568 | 1.316768 | 0.849352 | 0.881629 | 0.716157 | 2.8E-101 |
| IRS2 | 0.376659 | 2.346313 | 1.807175 | 0.979783 | 0.913537 | 3.6E-101 |
| HRH2 | 0.66852 | 1.269786 | 0.798888 | 0.872693 | 0.684716 | 1.8E-99 |
| NIBAN1 | 0.957049 | 0.894754 | 0.460896 | 0.757838 | 0.439301 | 4.18E-96 |
| CYP1B1 | 0.637276 | 2.112027 | 1.357876 | 0.89833 | 0.721397 | 9.83E-96 |
| PPP1CB | 0.428131 | 1.536027 | 1.141612 | 0.948139 | 0.846288 | 3.44E-95 |
| PIM1 | 0.811123 | 1.300021 | 0.740931 | 0.818781 | 0.569432 | 4.64E-93 |
| NFIL3 | 0.445994 | 1.805972 | 1.325725 | 0.936713 | 0.827074 | 4.44E-82 |
| LIPA | 0.631051 | 1.385416 | 0.894571 | 0.844125 | 0.689083 | 2.36E-81 |
| FPR2 | 0.844113 | 1.117198 | 0.622339 | 0.7615 | 0.51179 | 1.28E-80 |
| EIF1AY | 2.604443 | 0.286839 | 0.047165 | 0.332113 | 0.049782 | 1.5E-77 |
| LIMK2 | 1.258381 | 0.647396 | 0.27062 | 0.581746 | 0.28559 | 2.78E-77 |
| MXD1 | 0.427165 | 1.692934 | 1.259072 | 0.939936 | 0.829694 | 4.64E-77 |
| MBOAT7 | 0.385708 | 1.711827 | 1.310238 | 0.950923 | 0.855895 | 1.06E-76 |
| RNF144B | 0.812554 | 0.974345 | 0.554766 | 0.762819 | 0.50131 | 5.58E-76 |
| RNF149 | 0.354028 | 1.854979 | 1.451329 | 0.972458 | 0.891703 | 1.16E-75 |
| CCND3 | 0.431906 | 1.55398 | 1.151937 | 0.921477 | 0.816594 | 1.37E-75 |
| KDM5D | 2.666112 | 0.272551 | 0.042941 | 0.321858 | 0.046288 | 1.98E-75 |
| STAT3 | 0.470823 | 1.327094 | 0.957568 | 0.901113 | 0.765939 | 3.34E-75 |
| RUNX1 | 0.378489 | 1.786517 | 1.374265 | 0.949897 | 0.855022 | 1.69E-74 |
| SAMSN1 | 0.608124 | 1.461967 | 0.959122 | 0.858482 | 0.675109 | 1.93E-74 |
| GASK1B | 0.683901 | 1.110278 | 0.691125 | 0.802666 | 0.595633 | 1.72E-73 |
| FOSL2 | 0.321501 | 1.991211 | 1.59344 | 0.967184 | 0.899563 | 2.57E-72 |
| BCL3 | 0.413369 | 1.659262 | 1.245887 | 0.939789 | 0.814847 | 9.79E-70 |
| FLOT1 | 0.544089 | 1.173496 | 0.804812 | 0.860094 | 0.692576 | 6.16E-68 |
| ZBTB16 | 1.663833 | 0.437277 | 0.138004 | 0.432757 | 0.153712 | 8.18E-68 |
| LILRA6 | 1.022347 | 0.582062 | 0.286558 | 0.613097 | 0.321397 | 1.03E-67 |
| LMNB1 | 0.666405 | 1.155789 | 0.728233 | 0.791532 | 0.59738 | 3.33E-67 |
| HBEGF | 0.669862 | 1.43857 | 0.904237 | 0.807354 | 0.598253 | 9.63E-67 |
| ETS2 | 0.533835 | 1.223276 | 0.844937 | 0.868884 | 0.710044 | 5.66E-66 |
| RAB20 | 1.013721 | 0.687155 | 0.340325 | 0.617345 | 0.353712 | 1.19E-65 |
| DSE | 0.70198 | 0.992468 | 0.610098 | 0.755787 | 0.558079 | 2.63E-65 |
| PFKFB3 | 0.552479 | 1.473823 | 1.004923 | 0.872546 | 0.70917 | 3.6E-65 |
| FLVCR2 | 1.14444 | 0.536454 | 0.242673 | 0.552007 | 0.273362 | 9.28E-65 |
| TIMP1 | 0.383289 | 1.775389 | 1.361169 | 0.938617 | 0.840175 | 1.3E-64 |
| SLA | 0.591493 | 1.134766 | 0.753094 | 0.835482 | 0.647162 | 1.51E-64 |
| PTPN1 | 0.599082 | 1.012624 | 0.668508 | 0.812335 | 0.625328 | 1.12E-63 |
| CD63 | 0.474344 | 1.249598 | 0.899453 | 0.8724 | 0.732751 | 1.67E-62 |
| OTUD1 | 0.476775 | 1.553509 | 1.116324 | 0.904629 | 0.769432 | 1.91E-61 |
| SEMA6B | 1.740434 | 0.395948 | 0.118499 | 0.397158 | 0.142358 | 5.5E-61 |
| FCGR2A | 0.542366 | 1.126444 | 0.773466 | 0.859508 | 0.673362 | 2.25E-60 |
| NIBAN2 | 0.59214 | 1.036711 | 0.687711 | 0.806182 | 0.613974 | 3.82E-60 |
| FAM20A | 7.238687 | 0.17018 | 0.001127 | 0.204659 | 0.001747 | 6.47E-60 |
| GLUL | 0.279733 | 2.295178 | 1.890638 | 0.978611 | 0.924017 | 3.94E-59 |
| C5AR1 | 0.338064 | 2.518573 | 1.992449 | 0.956197 | 0.871616 | 8.51E-58 |
| SMAP2 | 0.226286 | 2.227652 | 1.90427 | 0.984032 | 0.933624 | 8.67E-58 |
| THBD | 1.119561 | 0.881656 | 0.405768 | 0.573249 | 0.322271 | 1.84E-57 |
| CCR2 | 0.675607 | 1.04561 | 0.654624 | 0.758424 | 0.539738 | 4.15E-57 |
| ATP5PO | 2.394642 | 0.200033 | 0.03804 | 0.27322 | 0.047162 | 1.8E-56 |
| HLX | 0.922119 | 0.68088 | 0.359323 | 0.601084 | 0.348472 | 5.91E-56 |
| GPR27 | 1.602024 | 0.363564 | 0.119763 | 0.385438 | 0.136245 | 9.45E-56 |
| HP | 2.019144 | 0.36234 | 0.089391 | 0.334896 | 0.10655 | 2.82E-55 |
| BIN1 | 1.560546 | 0.335591 | 0.113773 | 0.38368 | 0.138865 | 4.62E-55 |
| DYSF | 0.559322 | 1.383478 | 0.938857 | 0.794462 | 0.644541 | 2.98E-54 |
| CD163 | 0.458234 | 1.350971 | 0.983341 | 0.849253 | 0.724017 | 3.98E-54 |
| FLOT2 | 0.366944 | 1.47279 | 1.142035 | 0.925139 | 0.823581 | 5.25E-53 |
| GAS6 | 1.585815 | 0.320704 | 0.106838 | 0.360973 | 0.124891 | 8.46E-53 |
| ADK | 1.078042 | 0.479399 | 0.227078 | 0.51465 | 0.262009 | 2.39E-52 |
| SLC7A5 | 0.813317 | 0.876558 | 0.498824 | 0.65104 | 0.434061 | 5.89E-52 |
| GRK6 | 0.404261 | 1.29742 | 0.980361 | 0.89496 | 0.777293 | 1.35E-51 |
| PLIN3 | 0.459942 | 1.110159 | 0.807102 | 0.850718 | 0.707424 | 4.41E-51 |
| NRG1 | 1.441534 | 0.427193 | 0.157282 | 0.389979 | 0.156332 | 1.41E-50 |
| TSPAN14 | 0.349768 | 1.422763 | 1.116457 | 0.924114 | 0.820087 | 5.64E-50 |
| NANS | 0.767685 | 0.678711 | 0.398647 | 0.652945 | 0.424454 | 1.09E-49 |
| ADCY3 | 2.13605 | 0.24942 | 0.056743 | 0.278494 | 0.068996 | 1.12E-49 |
| BLOC1S1 | 0.542577 | 0.931961 | 0.639832 | 0.789188 | 0.596507 | 2.43E-48 |
| LILRA5 | 0.276754 | 1.909689 | 1.576345 | 0.955757 | 0.892576 | 2.96E-48 |
| UBALD2 | 0.576928 | 0.893932 | 0.599283 | 0.772781 | 0.580786 | 3.19E-48 |
| NECTIN2 | 1.26852 | 0.393287 | 0.163248 | 0.422209 | 0.18952 | 3.65E-48 |
| CEMIP2 | 0.735589 | 0.718588 | 0.431564 | 0.667301 | 0.436681 | 4.41E-48 |
| EOLA1 | 0.832854 | 0.735084 | 0.412689 | 0.626575 | 0.406987 | 7.68E-48 |
| VSIR | 0.253984 | 1.854238 | 1.554922 | 0.964108 | 0.905677 | 9.61E-48 |
| DDIT4 | 0.562666 | 1.322173 | 0.895177 | 0.790653 | 0.604367 | 3.9E-47 |
| XBP1 | 0.452104 | 1.206331 | 0.881799 | 0.840902 | 0.702183 | 1.74E-46 |
| SH3BP5 | 0.506785 | 1.073058 | 0.755206 | 0.810431 | 0.646288 | 6.58E-46 |
| PADI2 | 0.582316 | 1.175897 | 0.785371 | 0.744653 | 0.587773 | 2.68E-45 |
| PITPNA | 0.428253 | 1.13104 | 0.840545 | 0.85482 | 0.712664 | 7.14E-45 |
| NFE2 | 0.444687 | 1.283031 | 0.942699 | 0.850132 | 0.712664 | 1.48E-44 |
| FMN1 | 1.116527 | 0.440898 | 0.203343 | 0.443745 | 0.218341 | 2.8E-44 |
| ELL2 | 0.634616 | 0.865501 | 0.55748 | 0.702754 | 0.515284 | 1.22E-42 |
| PXN | 0.288966 | 1.492602 | 1.221678 | 0.935394 | 0.856769 | 2.01E-42 |
| SERPINB9 | 0.365998 | 1.649952 | 1.280251 | 0.926165 | 0.78952 | 1.52E-41 |
| TLE3 | 0.598783 | 0.774951 | 0.511708 | 0.717551 | 0.51441 | 3.66E-41 |
| YWHAB | 0.470618 | 0.911753 | 0.657972 | 0.796513 | 0.632314 | 1.02E-39 |
| AREG | 1.954322 | 0.340531 | 0.087871 | 0.259156 | 0.078603 | 1.97E-39 |
| CPEB4 | 0.586293 | 0.779631 | 0.519275 | 0.702461 | 0.527511 | 2.11E-39 |
| PLCXD1 | 1.121958 | 0.411442 | 0.189045 | 0.410782 | 0.206987 | 2.23E-38 |
| CNN2 | 0.318841 | 1.488551 | 1.193392 | 0.911222 | 0.811354 | 3.39E-38 |
| PDE4D | 0.642347 | 0.839925 | 0.538115 | 0.658219 | 0.481223 | 7.96E-38 |
| PHF21A | 0.483269 | 0.886316 | 0.634031 | 0.771023 | 0.608734 | 1.23E-37 |
| TBC1D14 | 0.487101 | 0.902964 | 0.644226 | 0.771023 | 0.608734 | 1.44E-37 |
| CLEC12A | 0.367846 | 1.225418 | 0.949624 | 0.873132 | 0.746725 | 3.88E-37 |
| TRIM25 | 0.342397 | 1.366558 | 1.077845 | 0.901406 | 0.8 | 3.89E-37 |
| VNN2 | 0.609002 | 0.958484 | 0.62843 | 0.686639 | 0.497817 | 6.53E-37 |
| SGMS1 | 0.61494 | 0.769308 | 0.502325 | 0.67741 | 0.49083 | 6.63E-37 |
| B4GALT5 | 0.631816 | 0.705688 | 0.455426 | 0.659977 | 0.467249 | 7.85E-37 |
| CTSA | 0.241504 | 1.941047 | 1.641861 | 0.956929 | 0.892576 | 1.69E-36 |
| PHACTR1 | 0.496467 | 1.035179 | 0.733777 | 0.7678 | 0.60786 | 2.67E-36 |
| FURIN | 0.377106 | 1.14238 | 0.87961 | 0.859361 | 0.729258 | 3.1E-36 |
| TRIB1 | 0.309417 | 1.732791 | 1.398307 | 0.940668 | 0.844541 | 1.3E-35 |
| HSPA8 | 0.343089 | 1.329389 | 1.048025 | 0.885878 | 0.772926 | 1.54E-35 |
| PELI1 | 0.439631 | 1.209256 | 0.891613 | 0.843686 | 0.677729 | 5.22E-35 |
| MAFB | 0.284368 | 2.286156 | 1.877166 | 0.94184 | 0.868996 | 1.88E-34 |
| LAP3 | 0.393277 | 1.115335 | 0.849214 | 0.83138 | 0.682969 | 3.92E-34 |
| SIRPB1 | 0.366159 | 1.162159 | 0.901655 | 0.858482 | 0.737118 | 5.31E-34 |
| IFITM2 | 0.321026 | 1.429993 | 1.14471 | 0.915763 | 0.806987 | 5.44E-34 |
| LILRA2 | 0.355098 | 1.233357 | 0.964259 | 0.876648 | 0.751092 | 1.17E-33 |
| EHD1 | 0.379169 | 1.229807 | 0.945574 | 0.840609 | 0.720524 | 1.65E-33 |
| HS3ST3B1 | 1.388105 | 0.293458 | 0.112121 | 0.305889 | 0.130131 | 4.56E-33 |
| IL1RAP | 0.828565 | 0.492232 | 0.27717 | 0.499268 | 0.30393 | 4.88E-33 |
| USB1 | 0.53331 | 0.790041 | 0.545893 | 0.711251 | 0.537118 | 5.17E-33 |
| IDI1 | 0.619809 | 0.670972 | 0.43664 | 0.627454 | 0.439301 | 6.1E-33 |
| SERPINB2 | 0.803804 | 1.160738 | 0.664913 | 0.54058 | 0.358952 | 8.83E-33 |
| NDRG1 | 0.410949 | 1.170057 | 0.880033 | 0.823762 | 0.70393 | 9.9E-33 |
| RABGEF1 | 0.56681 | 0.777775 | 0.525082 | 0.679461 | 0.505677 | 1.05E-32 |
| CYP27A1 | 0.921542 | 0.468181 | 0.247173 | 0.437885 | 0.241048 | 1.49E-32 |
| NCF4 | 0.311856 | 1.292169 | 1.040977 | 0.887636 | 0.79738 | 1.58E-32 |
| ABCA1 | 0.918068 | 0.486399 | 0.257411 | 0.440522 | 0.247162 | 2.07E-32 |
| VAPA | 0.369054 | 1.068074 | 0.826999 | 0.843246 | 0.718777 | 2.65E-32 |
| F5 | 0.79979 | 0.544993 | 0.313062 | 0.506006 | 0.31441 | 3.25E-32 |
| MS4A4A | 1.481356 | 0.242499 | 0.086851 | 0.277029 | 0.108297 | 3.46E-32 |
| ENGASE | 0.826013 | 0.435237 | 0.245511 | 0.475974 | 0.282969 | 5.48E-32 |
| GPR183 | 0.497877 | 1.586667 | 1.123595 | 0.745239 | 0.598253 | 7.41E-32 |
| PPP1R3B | 1.577146 | 0.209218 | 0.070118 | 0.245971 | 0.084716 | 7.58E-32 |
| ADM | 0.950768 | 0.487743 | 0.252337 | 0.419426 | 0.228821 | 8.37E-32 |
| STOM | 0.853991 | 0.403436 | 0.223202 | 0.441401 | 0.247162 | 1.51E-31 |
| MAP3K8 | 0.237797 | 2.302719 | 1.952795 | 0.960738 | 0.891703 | 2.64E-31 |
| ACTN4 | 0.352742 | 1.077996 | 0.844173 | 0.849399 | 0.723144 | 3.36E-31 |
| CTDSP1 | 0.211541 | 1.771133 | 1.529575 | 0.961764 | 0.905677 | 5.4E-31 |
| SULT1A1 | 0.914626 | 0.370714 | 0.196657 | 0.421477 | 0.231441 | 5.82E-31 |
| PRKCA | 1.008276 | 0.348892 | 0.173448 | 0.383534 | 0.20262 | 6.16E-31 |
| PYGL | 0.377264 | 1.068811 | 0.822874 | 0.828157 | 0.70393 | 1.23E-30 |
| ZFY | 3.425304 | 0.098213 | 0.009142 | 0.132142 | 0.011354 | 1.48E-30 |
| BCL6 | 0.458315 | 0.921268 | 0.670532 | 0.764284 | 0.59214 | 1.85E-30 |
| APBB3 | 0.365448 | 1.623194 | 1.259968 | 0.852036 | 0.754585 | 1.93E-30 |
| ASGR2 | 0.542152 | 0.914919 | 0.628317 | 0.700264 | 0.543231 | 2.26E-30 |
| PAG1 | 0.632382 | 0.587652 | 0.3791 | 0.57823 | 0.395633 | 1.64E-29 |
| SLC25A37 | 0.458225 | 0.90456 | 0.658412 | 0.756373 | 0.600873 | 2.11E-29 |
| GNAI3 | 0.277892 | 1.346469 | 1.110561 | 0.91547 | 0.824454 | 2.24E-29 |
| TFRC | 0.627059 | 0.748276 | 0.484505 | 0.616027 | 0.445415 | 2.42E-29 |
| MAN1A1 | 0.617936 | 0.600199 | 0.391091 | 0.601963 | 0.408734 | 2.5E-29 |
| CCNL1 | 0.229499 | 1.604629 | 1.368638 | 0.951802 | 0.89083 | 2.66E-29 |
| ECE1 | 0.377556 | 1.215438 | 0.935572 | 0.834603 | 0.686463 | 2.9E-29 |
| KYNU | 0.363099 | 1.169897 | 0.909586 | 0.845444 | 0.726638 | 5.71E-29 |
| LILRA1 | 0.373329 | 1.140181 | 0.880219 | 0.835628 | 0.708297 | 6.12E-29 |
| PLK3 | 0.330899 | 1.252843 | 0.996061 | 0.875623 | 0.757205 | 6.38E-29 |
| LRG1 | 0.530432 | 0.936407 | 0.648319 | 0.659684 | 0.460262 | 1E-28 |
| MTHFS | 0.639851 | 0.595877 | 0.382422 | 0.571784 | 0.386026 | 1.31E-28 |
| DSC2 | 0.858788 | 0.407292 | 0.224587 | 0.437299 | 0.254148 | 1.57E-28 |
| ARHGAP26 | 0.247029 | 1.478855 | 1.246127 | 0.944623 | 0.870742 | 1.64E-28 |
| CD82 | 0.736343 | 0.540555 | 0.324473 | 0.512599 | 0.337991 | 1.86E-28 |
| BCL2A1 | 0.278482 | 1.816677 | 1.497773 | 0.926018 | 0.839301 | 4.61E-28 |
| ABCA7 | 0.804598 | 0.394623 | 0.22593 | 0.437445 | 0.249782 | 1.49E-27 |
| CDKN2D | 0.36156 | 1.136668 | 0.884694 | 0.84559 | 0.69345 | 2.19E-27 |
| YBX3 | 0.297903 | 1.510599 | 1.228772 | 0.887929 | 0.787773 | 5.27E-27 |
| TP53INP2 | 0.499422 | 0.79859 | 0.564915 | 0.687811 | 0.529258 | 5.71E-27 |
| MAPK7 | 0.450636 | 0.898001 | 0.657085 | 0.741283 | 0.583406 | 6.43E-27 |
| CSF2RB | 0.293303 | 1.345395 | 1.097885 | 0.900234 | 0.796507 | 7.09E-27 |
| SLC66A2 | 0.462884 | 0.750954 | 0.544843 | 0.711837 | 0.539738 | 8.68E-27 |
| BASP1 | 0.611573 | 0.699922 | 0.458087 | 0.596982 | 0.423581 | 1.17E-26 |
| SH3PXD2B | 1.9489 | 0.153551 | 0.039772 | 0.175652 | 0.048035 | 2.2E-26 |
| KLHL2 | 0.621974 | 0.517003 | 0.335939 | 0.556695 | 0.372926 | 3.67E-26 |
| ASPH | 0.852933 | 0.356268 | 0.197251 | 0.406241 | 0.236681 | 5.73E-26 |
| ACSL3 | 0.709984 | 0.462135 | 0.282516 | 0.502051 | 0.333624 | 7.66E-26 |
| SIPA1L2 | 1.296638 | 0.200477 | 0.081609 | 0.251538 | 0.102183 | 8.69E-26 |
| EIF4E3 | 0.312468 | 1.22449 | 0.986036 | 0.855845 | 0.745852 | 1.68E-25 |
| PTPN2 | 0.629325 | 0.497009 | 0.321306 | 0.548931 | 0.371179 | 1.77E-25 |
| HSPA1B | 0.767825 | 0.351422 | 0.206391 | 0.402871 | 0.226201 | 3.93E-25 |
| FNDC3B | 0.318987 | 1.107982 | 0.888194 | 0.854966 | 0.740611 | 4.57E-25 |
| NQO2 | 0.623894 | 0.515488 | 0.334509 | 0.539994 | 0.361572 | 8.36E-25 |
| FFAR2 | 0.791624 | 0.405898 | 0.234485 | 0.396425 | 0.224454 | 8.58E-25 |
| IL10 | 1.735218 | 0.161726 | 0.048577 | 0.179314 | 0.054148 | 9.81E-25 |
| PGS1 | 0.469991 | 0.730003 | 0.527039 | 0.697627 | 0.540611 | 1.83E-24 |
| CTSG | 5.089205 | 0.076956 | 0.002261 | 0.094931 | 0.00262 | 1.93E-24 |
| LDLRAD3 | 0.872502 | 0.322312 | 0.176047 | 0.374158 | 0.209607 | 2.61E-24 |
| XRRA1 | 2.347631 | 0.098571 | 0.019366 | 0.132581 | 0.024454 | 5.75E-24 |
| WWC3 | 0.744764 | 0.417712 | 0.249276 | 0.445063 | 0.282096 | 9.56E-24 |
| LDLR | 0.389898 | 1.190744 | 0.908757 | 0.763551 | 0.615721 | 1.23E-23 |
| TNFRSF10D | 0.889459 | 0.318906 | 0.172151 | 0.359508 | 0.200873 | 1.58E-23 |
| SRA1 | 0.254532 | 1.917241 | 1.607145 | 0.920891 | 0.849782 | 1.81E-23 |
| LONRF3 | 0.833111 | 0.376514 | 0.211344 | 0.394667 | 0.234061 | 2.18E-23 |
| TRMT6 | 0.675801 | 0.473503 | 0.296405 | 0.493701 | 0.336245 | 2.69E-23 |
| KCNN4 | 0.9522 | 0.266485 | 0.137731 | 0.310577 | 0.154585 | 2.7E-23 |
| PRR7 | 0.659395 | 0.454724 | 0.287905 | 0.474509 | 0.295197 | 3E-23 |
| POLR2L | 0.2373 | 1.322584 | 1.121989 | 0.895693 | 0.817467 | 4.21E-23 |
| DNTTIP2 | 0.327109 | 1.131118 | 0.90165 | 0.820393 | 0.697817 | 4.25E-23 |
| HFE | 1.324993 | 0.17674 | 0.070546 | 0.212716 | 0.081223 | 5.89E-23 |
| FGD4 | 0.440469 | 0.750914 | 0.553345 | 0.685321 | 0.525764 | 8.94E-23 |
| TNFRSF1A | 0.253407 | 1.436276 | 1.204911 | 0.92426 | 0.827948 | 1.22E-22 |
| LAPTM4A | 0.407989 | 0.744884 | 0.561399 | 0.72385 | 0.585153 | 1.24E-22 |
| CH25H | 1.199185 | 0.319513 | 0.139155 | 0.240844 | 0.104803 | 1.32E-22 |
| DOK3 | 0.316067 | 1.095223 | 0.879745 | 0.846176 | 0.715284 | 1.58E-22 |
| FCGR1A | 0.765284 | 0.349829 | 0.205818 | 0.410929 | 0.246288 | 1.58E-22 |
| SLC16A3 | 0.177605 | 1.811615 | 1.601775 | 0.964108 | 0.912664 | 2.42E-22 |
| WDFY3 | 0.503047 | 0.593124 | 0.418517 | 0.610021 | 0.460262 | 3.19E-22 |
| HSPA1A | 0.430277 | 0.706676 | 0.524438 | 0.673454 | 0.510044 | 7.01E-22 |
| IRAK3 | 0.208056 | 1.600874 | 1.385882 | 0.951069 | 0.881223 | 7.42E-22 |
| NR1H2 | 0.309213 | 1.074177 | 0.866949 | 0.844125 | 0.720524 | 9.75E-22 |
| ATP13A3 | 0.586202 | 0.503168 | 0.335157 | 0.544536 | 0.380786 | 1.16E-21 |
| CXCL16 | 0.379011 | 1.018963 | 0.783547 | 0.769118 | 0.622707 | 1.22E-21 |
| STEAP4 | 0.705093 | 0.468858 | 0.287599 | 0.422502 | 0.263755 | 2.51E-21 |
| NR4A3 | 0.401304 | 1.092879 | 0.827499 | 0.727219 | 0.587773 | 3.44E-21 |
| SH3TC1 | 0.38696 | 0.802036 | 0.613348 | 0.724875 | 0.593013 | 3.68E-21 |
| SELPLG | 0.17802 | 1.812761 | 1.602328 | 0.948579 | 0.897817 | 6.56E-21 |
| NR4A1 | 0.290975 | 1.636682 | 1.337741 | 0.833431 | 0.755459 | 1.04E-20 |
| CD7 | 1.008461 | 0.220885 | 0.109797 | 0.275271 | 0.137991 | 1.2E-20 |
| CHSY1 | 0.403102 | 0.785004 | 0.593644 | 0.70671 | 0.571179 | 1.23E-20 |
| ARID5B | 1.309507 | 0.153662 | 0.061996 | 0.186493 | 0.068996 | 1.39E-20 |
| CSNK1D | 0.271266 | 1.151155 | 0.953838 | 0.866686 | 0.761572 | 1.76E-20 |
| GZF1 | 0.443352 | 0.809471 | 0.595304 | 0.659537 | 0.528384 | 1.93E-20 |
| NR4A2 | 0.178712 | 2.653681 | 2.344504 | 0.974949 | 0.90917 | 2.59E-20 |
| EREG | 0.565443 | 1.007355 | 0.680718 | 0.51172 | 0.350218 | 3.37E-20 |
| PIK3AP1 | 0.209629 | 1.517131 | 1.311954 | 0.918986 | 0.858515 | 4.7E-20 |
| SECTM1 | 0.320312 | 1.109178 | 0.888337 | 0.806036 | 0.682096 | 4.78E-20 |
| MERTK | 1.604845 | 0.148811 | 0.048925 | 0.171257 | 0.061135 | 5.21E-20 |
| DUSP3 | 0.420191 | 0.714202 | 0.533741 | 0.676384 | 0.537118 | 6.21E-20 |
| H3-3B | 0.310093 | 0.955673 | 0.770836 | 0.816584 | 0.684716 | 7.23E-20 |
| PDGFC | 0.933255 | 0.234418 | 0.122759 | 0.27908 | 0.141485 | 7.31E-20 |
| IVNS1ABP | 0.246725 | 1.523639 | 1.284134 | 0.904922 | 0.825328 | 7.45E-20 |
| TACSTD2 | 2.429726 | 0.08666 | 0.016084 | 0.104307 | 0.017467 | 1.93E-19 |
| ENO1 | 0.192041 | 1.437277 | 1.258143 | 0.913419 | 0.859389 | 2.46E-19 |
| IDS | 0.210632 | 1.484346 | 1.28271 | 0.921037 | 0.855895 | 2.81E-19 |
| HIF1A | 0.600676 | 0.432012 | 0.284888 | 0.490331 | 0.333624 | 3.3E-19 |
| GOLPH3 | 0.264866 | 1.1347 | 0.944383 | 0.843979 | 0.754585 | 6.14E-19 |
| PNPLA8 | 0.407696 | 0.731001 | 0.551048 | 0.693232 | 0.550218 | 6.29E-19 |
| ATF3 | 0.211601 | 1.965206 | 1.697109 | 0.899502 | 0.837555 | 7.87E-19 |
| FSTL3 | 0.823165 | 0.270754 | 0.15303 | 0.316877 | 0.177293 | 8.33E-19 |
| CCR1 | 0.317739 | 1.067332 | 0.856348 | 0.800762 | 0.689956 | 1.09E-18 |
| MAD1L1 | 0.533392 | 0.523179 | 0.361479 | 0.545268 | 0.401747 | 1.82E-18 |
| NKG7 | 0.944307 | 0.225705 | 0.117294 | 0.283914 | 0.154585 | 2.14E-18 |
| VPS9D1 | 0.626652 | 0.353653 | 0.229053 | 0.410782 | 0.261135 | 2.65E-18 |
| AMPD2 | 0.277724 | 1.125827 | 0.928685 | 0.84852 | 0.742358 | 3.05E-18 |
| TLR4 | 0.28989 | 1.069481 | 0.874798 | 0.833724 | 0.716157 | 3.65E-18 |
| MAP2K1 | 0.256327 | 1.23651 | 1.035227 | 0.871814 | 0.768559 | 5.3E-18 |
| TDRD9 | 1.081966 | 0.18188 | 0.085917 | 0.211544 | 0.095197 | 5.63E-18 |
| SESN2 | 0.636495 | 0.463417 | 0.298104 | 0.468796 | 0.336245 | 6.02E-18 |
| DICER1 | 0.349283 | 0.775129 | 0.608456 | 0.724143 | 0.584279 | 8.02E-18 |
| SPATA13 | 0.410049 | 0.736664 | 0.554411 | 0.670085 | 0.539738 | 8.23E-18 |
| EGR3 | 0.633714 | 0.451886 | 0.291248 | 0.4184 | 0.278603 | 1.14E-17 |
| NSFL1C | 0.280202 | 1.038182 | 0.854918 | 0.829915 | 0.726638 | 1.15E-17 |
| NABP1 | 0.468938 | 0.600414 | 0.433797 | 0.575593 | 0.424454 | 1.29E-17 |
| GK5 | 1.07448 | 0.168865 | 0.080184 | 0.214914 | 0.100437 | 1.96E-17 |
| CLMN | 0.62526 | 0.373481 | 0.242129 | 0.43012 | 0.286463 | 2.71E-17 |
| FAM110B | 1.578493 | 0.125978 | 0.042182 | 0.144008 | 0.048908 | 3E-17 |
| ENPP2 | 2.570704 | 0.074753 | 0.012583 | 0.094345 | 0.016594 | 3.11E-17 |
| SLC25A19 | 0.671467 | 0.407166 | 0.255646 | 0.413273 | 0.277729 | 3.39E-17 |
| ITPKC | 0.507316 | 0.407947 | 0.287003 | 0.42719 | 0.273362 | 3.9E-17 |
| HMOX1 | 0.255887 | 1.426556 | 1.194701 | 0.864928 | 0.779913 | 4.46E-17 |
| CTSD | 0.195799 | 1.520667 | 1.327678 | 0.91884 | 0.856769 | 8.13E-17 |
| MLLT1 | 0.339409 | 0.790459 | 0.624751 | 0.738793 | 0.59476 | 9.67E-17 |
| SLC1A3 | 2.37196 | 0.07014 | 0.01355 | 0.094052 | 0.017467 | 1.1E-16 |
| LIPN | 0.629583 | 0.391716 | 0.253191 | 0.411368 | 0.270742 | 1.1E-16 |
| UBAP1 | 0.306212 | 0.882228 | 0.713513 | 0.773074 | 0.654148 | 1.29E-16 |
| IFI27 | 4.204689 | 0.114511 | 0.00621 | 0.073103 | 0.006114 | 1.43E-16 |
| ADARB1 | 1.992315 | 0.07783 | 0.019561 | 0.108556 | 0.027074 | 1.77E-16 |
| ATP6V0C | 0.206386 | 1.267139 | 1.098236 | 0.907852 | 0.827948 | 1.89E-16 |
| TOM1 | 0.319586 | 0.856669 | 0.686449 | 0.765309 | 0.635808 | 1.94E-16 |
| CDKN1B | 0.235691 | 1.383869 | 1.175289 | 0.885438 | 0.788646 | 2.11E-16 |
| CHCHD7 | 0.486669 | 0.516403 | 0.368542 | 0.531058 | 0.386026 | 2.14E-16 |
| UTF1 | 1.409646 | 0.111514 | 0.041974 | 0.143276 | 0.049782 | 2.38E-16 |
| CLIC1 | 0.215534 | 1.204385 | 1.037249 | 0.887343 | 0.806987 | 3.51E-16 |
| CTSL | 0.52526 | 0.53833 | 0.37405 | 0.503662 | 0.366812 | 4.82E-16 |
| DACH1 | 1.718931 | 0.096 | 0.029162 | 0.118224 | 0.034061 | 5.16E-16 |
| PLEKHG2 | 0.802053 | 0.234298 | 0.134377 | 0.286844 | 0.162445 | 5.16E-16 |
| UPP1 | 0.26705 | 1.128913 | 0.938146 | 0.828304 | 0.732751 | 5.22E-16 |
| IFNAR2 | 0.306181 | 0.895218 | 0.724034 | 0.7615 | 0.659389 | 5.76E-16 |
| CD300C | 0.637208 | 0.305777 | 0.196601 | 0.374597 | 0.234934 | 5.83E-16 |
| MKNK2 | 0.261626 | 1.044168 | 0.87099 | 0.830941 | 0.728384 | 6.02E-16 |
| SLC49A4 | 0.408303 | 0.654812 | 0.493407 | 0.623205 | 0.472489 | 7.05E-16 |
| TLR2 | 0.191268 | 1.5182 | 1.329694 | 0.931292 | 0.865502 | 8.25E-16 |
| ARFGAP3 | 0.431453 | 0.556261 | 0.412476 | 0.577644 | 0.440175 | 1.2E-15 |
| RNASET2 | 0.233182 | 1.144233 | 0.973463 | 0.864635 | 0.787773 | 1.44E-15 |
| R3HDM4 | 0.223735 | 1.216123 | 1.041422 | 0.880164 | 0.798253 | 1.5E-15 |
| RGMA | 2.9907 | 0.065785 | 0.008276 | 0.073982 | 0.008734 | 2.31E-15 |
| GPRIN3 | 1.614718 | 0.089574 | 0.029248 | 0.125256 | 0.041048 | 2.71E-15 |
| EMP1 | 0.773568 | 0.43572 | 0.254883 | 0.316144 | 0.196507 | 2.73E-15 |
| XRN2 | 0.24902 | 1.019934 | 0.858242 | 0.824934 | 0.723144 | 4.3E-15 |
| ADAM9 | 0.498275 | 0.383552 | 0.271537 | 0.443891 | 0.299563 | 4.36E-15 |
| XRCC6 | 0.54085 | 0.367867 | 0.252859 | 0.446235 | 0.30393 | 4.72E-15 |
| ARPIN | 0.501811 | 0.458477 | 0.323786 | 0.484471 | 0.339738 | 5.09E-15 |
| CST7 | 2.601377 | 0.066697 | 0.01099 | 0.080135 | 0.0131 | 5.82E-15 |
| SFT2D3 | 0.671074 | 0.322591 | 0.202599 | 0.348081 | 0.221834 | 6.95E-15 |
| CTDP1 | 0.337508 | 0.741893 | 0.587139 | 0.693964 | 0.560699 | 8.69E-15 |
| SHB | 1.409419 | 0.109271 | 0.041136 | 0.137855 | 0.051528 | 8.95E-15 |
| MARCHF2 | 0.500884 | 0.407723 | 0.288127 | 0.461324 | 0.318777 | 1.26E-14 |
| P4HB | 0.35311 | 0.63657 | 0.498368 | 0.651187 | 0.517904 | 1.26E-14 |
| SLC16A7 | 0.772104 | 0.216083 | 0.12653 | 0.273953 | 0.156332 | 1.5E-14 |
| CSNK1A1 | 0.340524 | 0.657157 | 0.518993 | 0.675066 | 0.555459 | 1.51E-14 |
| FBXO33 | 0.566682 | 0.342885 | 0.231505 | 0.414005 | 0.280349 | 1.73E-14 |
| ZDHHC20 | 0.290918 | 0.834694 | 0.682263 | 0.751392 | 0.642795 | 1.85E-14 |
| CENPU | 1.668745 | 0.104979 | 0.033019 | 0.115587 | 0.037555 | 2.47E-14 |
| PCNX4 | 0.651318 | 0.263312 | 0.16765 | 0.302666 | 0.180786 | 2.85E-14 |
| SELENOK | 0.264263 | 0.994248 | 0.827834 | 0.789628 | 0.692576 | 3.3E-14 |
| SMOX | 0.897547 | 0.190021 | 0.102003 | 0.222531 | 0.117031 | 3.31E-14 |
| SAP30 | 0.376922 | 0.724336 | 0.557796 | 0.631116 | 0.524017 | 3.77E-14 |
| RBPJ | 0.362501 | 0.596522 | 0.463984 | 0.621008 | 0.486463 | 4.29E-14 |
| CHI3L2 | 2.356188 | 0.071177 | 0.013901 | 0.085116 | 0.018341 | 4.43E-14 |
| PPARG | 1.723059 | 0.084675 | 0.025649 | 0.104893 | 0.030568 | 4.51E-14 |
| CYBC1 | 0.237697 | 1.088419 | 0.923086 | 0.843686 | 0.739738 | 5.99E-14 |
| POR | 0.291999 | 0.835267 | 0.682221 | 0.743041 | 0.630568 | 8.24E-14 |
| RETN | 0.584424 | 0.485762 | 0.323962 | 0.439789 | 0.325764 | 9.43E-14 |
| MIF4GD | 0.444731 | 0.499408 | 0.366926 | 0.526663 | 0.395633 | 1.09E-13 |
| DNAJB9 | 0.418626 | 0.589243 | 0.440835 | 0.563873 | 0.434061 | 1.11E-13 |
| ERLIN1 | 0.532974 | 0.356633 | 0.246479 | 0.405655 | 0.275983 | 1.15E-13 |
| KSR1 | 0.290864 | 0.864492 | 0.706647 | 0.737328 | 0.644541 | 1.3E-13 |
| ELL | 0.328796 | 0.661266 | 0.526501 | 0.656168 | 0.525764 | 1.84E-13 |
| HPR | 1.77203 | 0.07443 | 0.021793 | 0.098154 | 0.027948 | 2.48E-13 |
| EVI5L | 0.664554 | 0.250977 | 0.158337 | 0.307061 | 0.187773 | 2.59E-13 |
| TSHZ3 | 0.568157 | 0.3236 | 0.218261 | 0.36903 | 0.244541 | 3.42E-13 |
| PACSIN2 | 0.286803 | 0.806699 | 0.661264 | 0.72927 | 0.616594 | 3.63E-13 |
| AAK1 | 0.371633 | 0.534532 | 0.413143 | 0.56944 | 0.435808 | 3.72E-13 |
| COX5B | 0.60626 | 0.268806 | 0.176578 | 0.348813 | 0.226201 | 3.84E-13 |
| PLB1 | 0.563264 | 0.326663 | 0.221075 | 0.378406 | 0.254148 | 4.02E-13 |
| EIF4G3 | 0.432862 | 0.470242 | 0.348351 | 0.514064 | 0.375546 | 4.26E-13 |
| TPM4 | 0.291795 | 0.79836 | 0.652168 | 0.727659 | 0.621834 | 4.64E-13 |
| ACAP1 | 0.352556 | 0.673483 | 0.527469 | 0.63639 | 0.519651 | 4.9E-13 |
| PNKD | 0.275563 | 0.967887 | 0.799598 | 0.760621 | 0.672489 | 4.98E-13 |
| RRP7A | 0.591208 | 0.284449 | 0.188813 | 0.352622 | 0.229694 | 5.18E-13 |
| OXSR1 | 0.303748 | 0.780764 | 0.632532 | 0.707442 | 0.603493 | 5.55E-13 |
| ATP2B4 | 0.353695 | 0.645604 | 0.505235 | 0.634046 | 0.500437 | 6.19E-13 |
| CCDC71L | 0.848255 | 0.15361 | 0.085324 | 0.189276 | 0.095197 | 6.57E-13 |
| OSM | 0.287938 | 1.659706 | 1.359418 | 0.771462 | 0.677729 | 8.2E-13 |
| TOMM40L | 0.616496 | 0.297669 | 0.194156 | 0.351157 | 0.234061 | 8.49E-13 |
| RHBDD2 | 0.434183 | 0.441953 | 0.327095 | 0.48535 | 0.346725 | 1.06E-12 |
| BCL2 | 1.232938 | 0.099461 | 0.042316 | 0.135365 | 0.055022 | 1.22E-12 |
| FCER2 | 1.95198 | 0.090049 | 0.023274 | 0.096396 | 0.029694 | 1.42E-12 |
| BEST1 | 0.392405 | 0.50014 | 0.381036 | 0.501904 | 0.359825 | 1.44E-12 |
| MGAT4A | 0.546296 | 0.342567 | 0.234582 | 0.401553 | 0.280349 | 1.57E-12 |
| CIC | 0.225234 | 1.04442 | 0.893455 | 0.832699 | 0.746725 | 1.6E-12 |
| DENND3 | 0.190075 | 1.298635 | 1.138332 | 0.893642 | 0.824454 | 1.78E-12 |
| DCXR | 0.471516 | 0.406971 | 0.29351 | 0.459273 | 0.331878 | 1.94E-12 |
| ADGRE2 | 0.270356 | 0.971933 | 0.805844 | 0.759449 | 0.657642 | 2.35E-12 |
| RALGDS | 0.384662 | 0.555895 | 0.425793 | 0.566364 | 0.447162 | 2.48E-12 |
| GRAMD1A | 0.316018 | 0.781038 | 0.627396 | 0.693818 | 0.584279 | 2.51E-12 |
| DNAJA4 | 0.629695 | 0.259066 | 0.167438 | 0.295195 | 0.186026 | 2.64E-12 |
| LILRB4 | 0.363878 | 0.563405 | 0.437807 | 0.585702 | 0.448908 | 2.7E-12 |
| TSEN34 | 0.383363 | 0.53621 | 0.411085 | 0.569733 | 0.443668 | 2.72E-12 |
| EAF1 | 0.259067 | 1.10107 | 0.920085 | 0.77864 | 0.674236 | 2.75E-12 |
| LRRC37A2 | 3.095109 | 0.039349 | 0.004605 | 0.057427 | 0.006114 | 3.36E-12 |
| TMED8 | 0.549104 | 0.304 | 0.207767 | 0.356138 | 0.236681 | 3.68E-12 |
| ANKRD28 | 0.918584 | 0.156334 | 0.082705 | 0.193818 | 0.102183 | 3.8E-12 |
| MAD2L2 | 0.450709 | 0.418483 | 0.306197 | 0.47993 | 0.349345 | 3.83E-12 |
| DDIT3 | 0.446854 | 0.457524 | 0.335658 | 0.467038 | 0.343231 | 4.08E-12 |
| GBGT1 | 0.504822 | 0.324142 | 0.228439 | 0.376795 | 0.253275 | 4.38E-12 |
| TOP1 | 0.27449 | 0.801336 | 0.662499 | 0.742162 | 0.631441 | 4.71E-12 |
| CYFIP1 | 0.274266 | 0.823824 | 0.681196 | 0.741723 | 0.633188 | 5.15E-12 |
| INKA2 | 0.519569 | 0.305516 | 0.213122 | 0.333431 | 0.218341 | 7.1E-12 |
| MAPK14 | 0.21574 | 1.123498 | 0.967449 | 0.844711 | 0.769432 | 7.29E-12 |
| BMP2K | 0.34883 | 0.624108 | 0.490063 | 0.631702 | 0.50917 | 7.88E-12 |
| PNP | 0.205892 | 1.457074 | 1.263286 | 0.868444 | 0.79476 | 9.76E-12 |
| FOSL1 | 0.482625 | 0.421433 | 0.301608 | 0.419719 | 0.30131 | 1.15E-11 |
| SIRPB2 | 0.218356 | 1.014475 | 0.871986 | 0.820539 | 0.728384 | 1.24E-11 |
| USP9Y | 2.721285 | 0.040796 | 0.006186 | 0.056109 | 0.006987 | 1.73E-11 |
| NTPCR | 0.858301 | 0.141851 | 0.078245 | 0.189716 | 0.100437 | 1.81E-11 |
| PXK | 0.314875 | 0.64634 | 0.519606 | 0.648257 | 0.517904 | 1.88E-11 |
| UBE2J1 | 0.210446 | 1.124864 | 0.972187 | 0.849692 | 0.759825 | 2.37E-11 |
| ATP2B1 | 0.224608 | 1.137665 | 0.973644 | 0.832845 | 0.737118 | 2.5E-11 |
| RNF44 | 0.182526 | 1.314994 | 1.158718 | 0.90375 | 0.831441 | 2.96E-11 |
| SLC12A9 | 0.187184 | 1.209052 | 1.061933 | 0.881629 | 0.806987 | 3.09E-11 |
| MMP25 | 0.882584 | 0.152035 | 0.082463 | 0.175359 | 0.09083 | 3.46E-11 |
| PRMT9 | 0.472913 | 0.446885 | 0.321984 | 0.445942 | 0.332751 | 3.49E-11 |
| TIPARP | 0.140407 | 2.353211 | 2.134983 | 0.965573 | 0.908297 | 3.74E-11 |
| TNNI2 | 0.936962 | 0.136597 | 0.071349 | 0.170085 | 0.086463 | 3.8E-11 |
| SGMS2 | 0.377996 | 0.527677 | 0.406051 | 0.539555 | 0.417467 | 3.8E-11 |
| CCDC69 | 0.17089 | 1.241051 | 1.102419 | 0.882362 | 0.820087 | 3.89E-11 |
| JOSD1 | 0.317313 | 0.656944 | 0.527239 | 0.635511 | 0.510917 | 4.85E-11 |
| SLC39A1 | 0.431295 | 0.390666 | 0.289716 | 0.446088 | 0.327511 | 5.76E-11 |
| NDST2 | 0.599007 | 0.195534 | 0.129093 | 0.263991 | 0.159825 | 5.95E-11 |
| STYXL1 | 0.499029 | 0.317322 | 0.224531 | 0.388222 | 0.275983 | 6.8E-11 |
| CREM | 0.557482 | 0.306687 | 0.20839 | 0.340463 | 0.231441 | 6.86E-11 |
| B3GALNT2 | 0.889546 | 0.128748 | 0.069496 | 0.172429 | 0.089956 | 8.74E-11 |
| CDKN2B | 0.916839 | 0.137805 | 0.072991 | 0.152066 | 0.075109 | 1.2E-10 |
| KLHDC7B | 0.668028 | 0.208011 | 0.130915 | 0.241576 | 0.145852 | 1.2E-10 |
| TRABD | 0.203262 | 1.108249 | 0.962608 | 0.859947 | 0.767686 | 1.33E-10 |
| CCNH | 0.342679 | 0.609534 | 0.480663 | 0.590536 | 0.475983 | 1.53E-10 |
| TMEM259 | 0.240243 | 0.855568 | 0.724326 | 0.768532 | 0.650655 | 1.57E-10 |
| SYAP1 | 0.260908 | 0.951449 | 0.794044 | 0.764284 | 0.660262 | 1.8E-10 |
| RBM39 | 0.191638 | 1.092991 | 0.957035 | 0.865807 | 0.769432 | 1.8E-10 |
| CARM1 | 0.411757 | 0.426945 | 0.320938 | 0.478904 | 0.361572 | 1.83E-10 |
| FYN | 0.204744 | 1.036739 | 0.899571 | 0.823469 | 0.739738 | 2.16E-10 |
| DDX60L | 0.354417 | 0.509971 | 0.398892 | 0.545415 | 0.416594 | 2.85E-10 |
| ADAMTSL4 | 0.234967 | 1.016871 | 0.86404 | 0.783914 | 0.678603 | 3.69E-10 |
| ABHD5 | 0.330053 | 0.598604 | 0.476194 | 0.574714 | 0.447162 | 3.8E-10 |
| CDA | 0.238739 | 1.00631 | 0.852834 | 0.79329 | 0.663755 | 3.82E-10 |
| SNIP1 | 0.427765 | 0.375097 | 0.278852 | 0.441547 | 0.321397 | 3.95E-10 |
| WASF1 | 1.769883 | 0.0573 | 0.016802 | 0.076619 | 0.022707 | 4.14E-10 |
| KAT6A | 0.325465 | 0.531974 | 0.424537 | 0.572077 | 0.453275 | 4.79E-10 |
| ADIPOR1 | 0.200655 | 1.085892 | 0.944894 | 0.847495 | 0.762445 | 6.05E-10 |
| SEL1L3 | 1.129868 | 0.09102 | 0.041592 | 0.122326 | 0.055022 | 6.13E-10 |
| NLRC5 | 0.263022 | 0.743303 | 0.619425 | 0.691034 | 0.581659 | 6.65E-10 |
| QPCT | 0.274385 | 0.848066 | 0.701184 | 0.669206 | 0.564192 | 6.84E-10 |
| GUK1 | 0.293535 | 0.658565 | 0.537323 | 0.658072 | 0.551965 | 7.1E-10 |
| DMWD | 0.660744 | 0.152973 | 0.096763 | 0.193818 | 0.10917 | 7.8E-10 |
| CTNNA1 | 0.188675 | 0.995408 | 0.873382 | 0.814679 | 0.747598 | 9.69E-10 |
| RAP2B | 0.189976 | 1.070997 | 0.938857 | 0.823616 | 0.746725 | 1E-09 |
| SNX9 | 0.572694 | 0.297134 | 0.199781 | 0.344565 | 0.248908 | 1.05E-09 |
| GNA15 | 0.225389 | 0.952726 | 0.814928 | 0.769851 | 0.677729 | 1.08E-09 |
| PJA2 | 0.186689 | 0.985983 | 0.866304 | 0.821565 | 0.745852 | 1.08E-09 |
| APOBEC3A | 0.217975 | 1.28075 | 1.101152 | 0.819367 | 0.726638 | 1.1E-09 |
| YTHDF3 | 0.237035 | 0.85592 | 0.726238 | 0.750073 | 0.646288 | 1.18E-09 |
| SEC24D | 0.434984 | 0.311042 | 0.230078 | 0.379139 | 0.270742 | 1.19E-09 |
| HIPK2 | 0.300042 | 0.616918 | 0.501079 | 0.613976 | 0.50655 | 1.34E-09 |
| MTCH1 | 0.220143 | 0.877175 | 0.753037 | 0.764723 | 0.673362 | 1.39E-09 |
| PLIN5 | 0.745355 | 0.265791 | 0.15855 | 0.220481 | 0.136245 | 1.41E-09 |
| FBXO9 | 0.317916 | 0.511432 | 0.410285 | 0.556548 | 0.436681 | 1.42E-09 |
| SDCBP | 0.150805 | 1.546318 | 1.392843 | 0.943745 | 0.879476 | 1.42E-09 |
| ATOH8 | 0.911329 | 0.118974 | 0.063258 | 0.135951 | 0.066376 | 1.57E-09 |
| CXCR4 | 0.182691 | 1.810798 | 1.595418 | 0.888222 | 0.828821 | 1.59E-09 |
| RBM47 | 0.169912 | 1.217783 | 1.082484 | 0.86947 | 0.806114 | 1.61E-09 |
| CDK2AP2 | 0.566455 | 0.207069 | 0.139828 | 0.266774 | 0.170306 | 1.61E-09 |
| CMKLR1 | 0.804582 | 0.153613 | 0.087948 | 0.17448 | 0.096943 | 1.62E-09 |
| TREML2 | 1.130952 | 0.093233 | 0.042572 | 0.116027 | 0.052402 | 1.78E-09 |
| EPAS1 | 1.875724 | 0.054486 | 0.014847 | 0.068561 | 0.019214 | 1.78E-09 |
| IRF2BPL | 0.248236 | 1.000893 | 0.842677 | 0.732786 | 0.632314 | 2.19E-09 |
| TAMALIN | 0.303332 | 0.811268 | 0.657434 | 0.618517 | 0.517904 | 2.22E-09 |
| EGR2 | 0.435278 | 0.475618 | 0.351744 | 0.403457 | 0.302183 | 2.36E-09 |
| CLEC10A | 0.691254 | 0.31342 | 0.194106 | 0.25857 | 0.171179 | 2.38E-09 |
| STX2 | 0.451456 | 0.292723 | 0.21407 | 0.348667 | 0.239301 | 2.51E-09 |
| CHST15 | 0.212811 | 0.948531 | 0.818445 | 0.789628 | 0.692576 | 2.83E-09 |
| SSH2 | 0.266836 | 0.666789 | 0.554195 | 0.669645 | 0.548472 | 2.89E-09 |
| VAMP1 | 0.742657 | 0.160636 | 0.096002 | 0.194257 | 0.11441 | 2.92E-09 |
| LPCAT1 | 0.210318 | 0.982053 | 0.848834 | 0.808819 | 0.70917 | 2.99E-09 |
| MSC | 1.757325 | 0.056081 | 0.016589 | 0.070319 | 0.020961 | 3.04E-09 |
| TRIM8 | 0.129925 | 1.536215 | 1.403914 | 0.944916 | 0.892576 | 3.31E-09 |
| ARIH1 | 0.224123 | 0.802999 | 0.68746 | 0.736156 | 0.641048 | 3.37E-09 |
| NEDD9 | 0.374156 | 0.51672 | 0.398679 | 0.512013 | 0.406114 | 3.53E-09 |
| SH2B2 | 0.365833 | 0.393532 | 0.305389 | 0.431292 | 0.315284 | 3.68E-09 |
| RNF24 | 0.355232 | 0.501218 | 0.391824 | 0.535892 | 0.424454 | 3.7E-09 |
| MTHFR | 0.326243 | 0.478807 | 0.381902 | 0.520363 | 0.40262 | 3.7E-09 |
| JPT1 | 0.429564 | 0.318479 | 0.236467 | 0.388222 | 0.283843 | 3.77E-09 |
| PRR5 | 1.69042 | 0.046224 | 0.014322 | 0.064459 | 0.017467 | 4.21E-09 |
| SPAG5 | 0.546064 | 0.243733 | 0.166929 | 0.285672 | 0.191266 | 4.74E-09 |
| LOXHD1 | 1.013109 | 0.118564 | 0.058746 | 0.125696 | 0.061135 | 4.87E-09 |
| IL1R2 | 1.443349 | 0.073087 | 0.026875 | 0.080135 | 0.027948 | 4.98E-09 |
| NLRP6 | 2.963664 | 0.035448 | 0.004544 | 0.044829 | 0.006114 | 5.38E-09 |
| ADAM19 | 0.37888 | 0.451679 | 0.347356 | 0.454878 | 0.343231 | 5.44E-09 |
| NME1 | 0.602115 | 0.199932 | 0.131713 | 0.253882 | 0.165939 | 5.58E-09 |
| RHBDF2 | 0.278304 | 0.606722 | 0.500279 | 0.6109 | 0.504803 | 6.03E-09 |
| SLC15A4 | 0.325061 | 0.516386 | 0.412213 | 0.550103 | 0.434934 | 6.11E-09 |
| ACTN1 | 0.19572 | 1.035773 | 0.904371 | 0.818635 | 0.731878 | 6.75E-09 |
| LPCAT3 | 0.341458 | 0.426764 | 0.33682 | 0.49165 | 0.374672 | 7.49E-09 |
| PCGF3 | 0.337447 | 0.441471 | 0.349398 | 0.494433 | 0.376419 | 7.52E-09 |
| JDP2 | 0.213801 | 1.015955 | 0.87602 | 0.781131 | 0.708297 | 7.53E-09 |
| ELF2 | 0.372523 | 0.399934 | 0.308921 | 0.455757 | 0.346725 | 7.6E-09 |
| RAB1B | 0.179192 | 1.044616 | 0.922603 | 0.846176 | 0.772052 | 7.77E-09 |
| AFAP1 | 0.973379 | 0.091218 | 0.046459 | 0.109728 | 0.048908 | 8.43E-09 |
| CDK2 | 0.75757 | 0.112233 | 0.066385 | 0.147964 | 0.077729 | 9.72E-09 |
| TSSC4 | 0.459675 | 0.302317 | 0.21983 | 0.370495 | 0.265502 | 1.04E-08 |
| HAUS4 | 0.306841 | 0.554324 | 0.448121 | 0.566217 | 0.469869 | 1.14E-08 |
| SORL1 | 0.171048 | 1.274452 | 1.131965 | 0.884559 | 0.806987 | 1.19E-08 |
| C1RL | 0.369737 | 0.355221 | 0.274914 | 0.407266 | 0.30131 | 1.26E-08 |
| DEFB1 | 2.025632 | 0.0473 | 0.011617 | 0.056695 | 0.013974 | 1.27E-08 |
| PHF20L1 | 0.204519 | 0.944337 | 0.819523 | 0.792411 | 0.696943 | 1.29E-08 |
| RIT1 | 0.227212 | 0.825318 | 0.705056 | 0.733812 | 0.636681 | 1.3E-08 |
| SMARCB1 | 0.32471 | 0.472843 | 0.377546 | 0.529007 | 0.418341 | 1.31E-08 |
| ATP5PF | 0.537719 | 0.203389 | 0.140107 | 0.279226 | 0.185153 | 1.48E-08 |
| XKR8 | 0.375834 | 0.41979 | 0.323516 | 0.445502 | 0.344105 | 1.58E-08 |
| SLC39A8 | 0.727286 | 0.133158 | 0.080433 | 0.134193 | 0.068122 | 1.59E-08 |
| HIVEP2 | 0.817153 | 0.105568 | 0.059916 | 0.141078 | 0.074236 | 1.63E-08 |
| ASGR1 | 0.272793 | 0.603021 | 0.49913 | 0.60167 | 0.49345 | 1.72E-08 |
| NTNG2 | 0.908952 | 0.121414 | 0.064662 | 0.135218 | 0.070742 | 1.74E-08 |
| ACVR1B | 0.347751 | 0.41835 | 0.328743 | 0.464987 | 0.356332 | 1.89E-08 |
| USP37 | 0.485962 | 0.302269 | 0.215827 | 0.352183 | 0.259389 | 1.89E-08 |
| ATP6V0D1 | 0.13097 | 1.521053 | 1.389052 | 0.932611 | 0.875109 | 1.89E-08 |
| TMEM39A | 0.513766 | 0.206968 | 0.144958 | 0.269704 | 0.179913 | 2.15E-08 |
| CLTC | 0.499908 | 0.200366 | 0.141689 | 0.274832 | 0.181659 | 2.34E-08 |
| ENTPD7 | 0.622862 | 0.177218 | 0.115082 | 0.22092 | 0.138865 | 2.34E-08 |
| RASA2 | 0.422487 | 0.312041 | 0.232826 | 0.38075 | 0.281223 | 2.44E-08 |
| H1-0 | 0.337331 | 0.524102 | 0.414829 | 0.450923 | 0.339738 | 2.51E-08 |
| ZNF394 | 0.170597 | 1.308832 | 1.162865 | 0.882655 | 0.806987 | 2.61E-08 |
| KRT10 | 0.383897 | 0.347162 | 0.266053 | 0.43349 | 0.324017 | 3.09E-08 |
| CNOT1 | 0.609813 | 0.154001 | 0.100914 | 0.214328 | 0.131878 | 3.15E-08 |
| E2F2 | 0.717349 | 0.1398 | 0.085028 | 0.170964 | 0.100437 | 3.43E-08 |
| SEPTIN4 | 1.112574 | 0.159935 | 0.073965 | 0.127307 | 0.067249 | 3.84E-08 |
| CBX6 | 0.253293 | 0.654646 | 0.549234 | 0.641078 | 0.555459 | 3.91E-08 |
| CNEP1R1 | 0.404018 | 0.323657 | 0.244604 | 0.374304 | 0.274236 | 3.94E-08 |
| DHRS13 | 0.793034 | 0.121526 | 0.070136 | 0.146499 | 0.080349 | 3.98E-08 |
| ATP1B3 | 0.310937 | 0.569774 | 0.459305 | 0.591269 | 0.492576 | 3.98E-08 |
| NUMB | 0.128055 | 1.497483 | 1.370293 | 0.939643 | 0.883843 | 4.08E-08 |
| ZNF593 | 0.643862 | 0.162201 | 0.103808 | 0.209493 | 0.131878 | 4.59E-08 |
| SCAND1 | 0.232699 | 0.678051 | 0.577049 | 0.664958 | 0.567686 | 4.82E-08 |
| SSH1 | 0.323903 | 0.522652 | 0.417549 | 0.546587 | 0.446288 | 4.85E-08 |
| PFKFB4 | 0.300839 | 0.519987 | 0.422115 | 0.546147 | 0.441048 | 5.28E-08 |
| SLC38A2 | 0.182837 | 1.179478 | 1.039083 | 0.856578 | 0.782533 | 5.29E-08 |
| SMPDL3A | 0.514992 | 0.216244 | 0.151327 | 0.269851 | 0.180786 | 5.72E-08 |
| NDEL1 | 0.210703 | 0.931568 | 0.804983 | 0.780838 | 0.687336 | 5.86E-08 |
| ELMOD3 | 0.770373 | 0.105248 | 0.061703 | 0.142836 | 0.076856 | 6.23E-08 |
| MED16 | 0.365929 | 0.364802 | 0.283075 | 0.417961 | 0.312664 | 6.31E-08 |
| RBM5 | 0.354192 | 0.38292 | 0.299561 | 0.45356 | 0.351092 | 6.64E-08 |
| SHMT1 | 0.708421 | 0.15135 | 0.092624 | 0.178142 | 0.10655 | 6.73E-08 |
| FKBP8 | 0.234599 | 0.617665 | 0.524967 | 0.639027 | 0.538865 | 6.99E-08 |
| SERINC1 | 0.155598 | 1.156086 | 1.037888 | 0.872546 | 0.809607 | 7.65E-08 |
| CD81 | 0.508876 | 0.223205 | 0.156862 | 0.292997 | 0.20262 | 7.75E-08 |
| HERPUD1 | 0.147291 | 1.430797 | 1.29193 | 0.901553 | 0.841048 | 7.93E-08 |
| GALK1 | 0.295461 | 0.526835 | 0.429272 | 0.54395 | 0.444541 | 8.17E-08 |
| TRA2B | 0.132235 | 1.126101 | 1.027474 | 0.877088 | 0.798253 | 8.17E-08 |
| BLVRB | 0.155228 | 1.272667 | 1.142843 | 0.876209 | 0.81048 | 8.67E-08 |
| PRSS21 | 1.255529 | 0.063653 | 0.02666 | 0.081746 | 0.033188 | 8.75E-08 |
| DAB2 | 0.601519 | 0.152993 | 0.100832 | 0.193525 | 0.118777 | 8.86E-08 |
| SEC14L1 | 0.21572 | 0.830485 | 0.715144 | 0.742748 | 0.655895 | 9.35E-08 |
| KCNC3 | 0.380563 | 0.409091 | 0.314238 | 0.4247 | 0.337991 | 9.5E-08 |
| EZR | 0.178001 | 1.222647 | 1.080731 | 0.840609 | 0.763319 | 9.68E-08 |
| COPA | 0.180417 | 1.04475 | 0.921938 | 0.838265 | 0.749345 | 1.01E-07 |
| ORAI3 | 0.559373 | 0.16574 | 0.11247 | 0.208175 | 0.131878 | 1.12E-07 |
| MUC1 | 1.204751 | 0.067957 | 0.029483 | 0.08746 | 0.037555 | 1.14E-07 |
| VENTX | 0.382181 | 0.331717 | 0.254518 | 0.3891 | 0.289956 | 1.14E-07 |
| CSMD1 | 2.059113 | 0.037468 | 0.008991 | 0.04981 | 0.012227 | 1.17E-07 |
| NOP58 | 0.434918 | 0.250693 | 0.185447 | 0.315558 | 0.223581 | 1.19E-07 |
| CHD1 | 0.184744 | 0.936976 | 0.824357 | 0.794316 | 0.704803 | 1.22E-07 |
| C5AR2 | 0.366548 | 0.491527 | 0.381246 | 0.456929 | 0.360699 | 1.29E-07 |
| SPHK1 | 0.504838 | 0.221266 | 0.155935 | 0.25857 | 0.176419 | 1.29E-07 |
| SPINT2 | 0.208919 | 0.891095 | 0.770963 | 0.757691 | 0.652402 | 1.39E-07 |
| BTAF1 | 0.190231 | 0.906032 | 0.794105 | 0.771902 | 0.689956 | 1.43E-07 |
| G0S2 | 0.273635 | 1.474082 | 1.219409 | 0.586581 | 0.489083 | 1.45E-07 |
| PRDX2 | 0.498404 | 0.225988 | 0.159974 | 0.259303 | 0.177293 | 1.56E-07 |
| SERPINB10 | 1.298639 | 0.060316 | 0.024519 | 0.068415 | 0.024454 | 1.57E-07 |
| IL1RN | 0.236169 | 1.080689 | 0.917501 | 0.769997 | 0.686463 | 1.57E-07 |
| HNRNPF | 0.140226 | 1.348606 | 1.223694 | 0.900527 | 0.836681 | 1.59E-07 |
| ROMO1 | 0.256915 | 0.52936 | 0.443009 | 0.577058 | 0.469869 | 1.59E-07 |
| FFAR3 | 1.540221 | 0.061128 | 0.021018 | 0.069294 | 0.025328 | 1.62E-07 |
| ATF6 | 0.231139 | 0.662694 | 0.56459 | 0.658805 | 0.544105 | 1.63E-07 |
| CRKL | 0.293198 | 0.516521 | 0.421528 | 0.558453 | 0.450655 | 1.64E-07 |
| CLASRP | 0.208868 | 0.765906 | 0.662674 | 0.724143 | 0.620087 | 1.69E-07 |
| DHX38 | 0.30441 | 0.42804 | 0.346615 | 0.488134 | 0.383406 | 1.7E-07 |
| ZNF410 | 0.256947 | 0.633493 | 0.530142 | 0.626575 | 0.531004 | 1.73E-07 |
| PREX1 | 0.148307 | 1.242167 | 1.120818 | 0.89247 | 0.828821 | 1.75E-07 |
| ZNF141 | 0.439719 | 0.315206 | 0.232395 | 0.326253 | 0.239301 | 1.77E-07 |
| CDC42EP2 | 0.345994 | 0.441496 | 0.347354 | 0.422795 | 0.323144 | 1.78E-07 |
| PIM3 | 0.151389 | 1.562961 | 1.407264 | 0.912833 | 0.843668 | 1.95E-07 |
| C4orf48 | 0.3515 | 0.394104 | 0.308887 | 0.454585 | 0.351092 | 1.95E-07 |
| KCNJ15 | 0.957062 | 0.087496 | 0.045069 | 0.107823 | 0.053275 | 1.99E-07 |
| RDH5 | 2.230946 | 0.031366 | 0.006682 | 0.044536 | 0.009607 | 2.07E-07 |
| MSRB1 | 0.188247 | 1.036233 | 0.909472 | 0.812189 | 0.726638 | 2.08E-07 |
| C17orf107 | 0.370776 | 0.309726 | 0.239532 | 0.327132 | 0.237555 | 2.08E-07 |
| SUPT5H | 0.193861 | 0.868861 | 0.759613 | 0.772341 | 0.692576 | 2.12E-07 |
| BST1 | 0.155149 | 1.316601 | 1.18236 | 0.862145 | 0.801747 | 2.21E-07 |
| PAPSS2 | 1.001583 | 0.091989 | 0.045944 | 0.111632 | 0.056769 | 2.22E-07 |
| DCUN1D3 | 0.512578 | 0.221947 | 0.155578 | 0.259303 | 0.178166 | 2.42E-07 |
| TEX14 | 0.535519 | 0.295422 | 0.203815 | 0.283768 | 0.20524 | 2.47E-07 |
| TRAF1 | 0.961658 | 0.071096 | 0.036505 | 0.094931 | 0.043668 | 2.5E-07 |
| MVP | 0.190943 | 0.901684 | 0.789905 | 0.79373 | 0.70131 | 2.56E-07 |
| CAB39 | 0.222626 | 0.720562 | 0.617525 | 0.696015 | 0.59476 | 2.61E-07 |
| EIF2AK3 | 0.416474 | 0.331138 | 0.248106 | 0.380604 | 0.287336 | 2.83E-07 |
| FAM131A | 0.481765 | 0.191796 | 0.137345 | 0.245385 | 0.164192 | 2.89E-07 |
| STAT5B | 0.223996 | 0.714695 | 0.611916 | 0.699531 | 0.598253 | 2.99E-07 |
| HYCC2 | 0.371023 | 0.338148 | 0.261467 | 0.390272 | 0.292576 | 3E-07 |
| ULK3 | 0.985189 | 0.073394 | 0.037076 | 0.102989 | 0.050655 | 3.1E-07 |
| GLB1L | 1.556587 | 0.044356 | 0.015079 | 0.061529 | 0.020961 | 3.2E-07 |
| HOXA5 | 1.95198 | 0.043602 | 0.011269 | 0.049224 | 0.0131 | 3.33E-07 |
| ZNF708 | 0.688743 | 0.115405 | 0.071597 | 0.153824 | 0.089083 | 3.34E-07 |
| CDKN1A | 0.169562 | 2.429001 | 2.159656 | 0.911661 | 0.815721 | 3.4E-07 |
| MAFF | 0.19933 | 1.235689 | 1.076229 | 0.776443 | 0.694323 | 3.77E-07 |
| ANXA6 | 0.178929 | 0.998258 | 0.881819 | 0.792265 | 0.725764 | 3.77E-07 |
| CYCS | 0.36793 | 0.409715 | 0.317485 | 0.456929 | 0.362445 | 3.8E-07 |
| RAB5C | 0.117213 | 1.726421 | 1.591704 | 0.95649 | 0.89869 | 3.81E-07 |
| MVD | 0.497311 | 0.203171 | 0.143931 | 0.254322 | 0.174672 | 4.29E-07 |
| PPP2R1B | 0.452892 | 0.219317 | 0.160228 | 0.274246 | 0.190393 | 4.34E-07 |
| RFX2 | 0.513327 | 0.219384 | 0.153701 | 0.250659 | 0.172052 | 4.53E-07 |
| SNX18 | 0.351339 | 0.359147 | 0.28152 | 0.41298 | 0.30917 | 4.7E-07 |
| RBMS1 | 0.190016 | 0.914947 | 0.802039 | 0.776443 | 0.692576 | 4.74E-07 |
| MTRR | 0.499395 | 0.176143 | 0.124604 | 0.22678 | 0.149345 | 4.99E-07 |
| TMEM121B | 0.579975 | 0.167539 | 0.11208 | 0.187372 | 0.119651 | 5.38E-07 |
| PLIN4 | 0.923569 | 0.16475 | 0.086857 | 0.108263 | 0.055895 | 5.38E-07 |
| CLK1 | 0.138673 | 1.285732 | 1.1679 | 0.893349 | 0.834934 | 5.45E-07 |
| MANF | 0.349016 | 0.405797 | 0.3186 | 0.441547 | 0.347598 | 5.52E-07 |
| WDR1 | 0.454019 | 0.180963 | 0.132104 | 0.25022 | 0.170306 | 5.61E-07 |
| SPTLC2 | 0.135257 | 1.293078 | 1.177357 | 0.90082 | 0.841048 | 5.89E-07 |
| TNFRSF8 | 0.56198 | 0.150335 | 0.101833 | 0.180926 | 0.113537 | 6.01E-07 |
| ST3GAL2 | 0.224741 | 0.665538 | 0.569534 | 0.64562 | 0.541485 | 6.04E-07 |
| MROH6 | 0.43531 | 0.231881 | 0.171484 | 0.271609 | 0.190393 | 6.32E-07 |
| DGAT2 | 0.422578 | 0.304918 | 0.227496 | 0.334017 | 0.250655 | 6.33E-07 |
| TXLNA | 0.257588 | 0.592537 | 0.495648 | 0.596689 | 0.494323 | 6.44E-07 |
| SDC2 | 4.263251 | 0.022413 | 0.001167 | 0.026663 | 0.000873 | 6.52E-07 |
| SEC16A | 0.261976 | 0.551052 | 0.459547 | 0.576179 | 0.470742 | 6.63E-07 |
| CHCHD10 | 0.306764 | 0.393592 | 0.3182 | 0.464987 | 0.360699 | 6.71E-07 |
| CHRFAM7A | 2.480169 | 0.024575 | 0.004404 | 0.036332 | 0.006114 | 6.99E-07 |
| TBC1D8 | 0.277446 | 0.489452 | 0.403823 | 0.512599 | 0.409607 | 7E-07 |
| ZNF668 | 0.289428 | 0.544789 | 0.445761 | 0.538383 | 0.439301 | 7.55E-07 |
| H2BC21 | 0.198784 | 0.740013 | 0.644762 | 0.630384 | 0.524891 | 7.92E-07 |
| NUAK2 | 0.34051 | 0.444691 | 0.3512 | 0.441254 | 0.347598 | 7.99E-07 |
| PLEKHG3 | 0.272806 | 0.495873 | 0.410438 | 0.497656 | 0.39214 | 8.02E-07 |
| ING1 | 0.451225 | 0.221102 | 0.161718 | 0.27615 | 0.19738 | 8.74E-07 |
| TTLL5 | 0.335472 | 0.333866 | 0.264597 | 0.3724 | 0.280349 | 8.93E-07 |
| FBXL19 | 0.376242 | 0.27246 | 0.209914 | 0.333431 | 0.243668 | 8.95E-07 |
| SLC24A4 | 0.321368 | 0.46799 | 0.374538 | 0.48579 | 0.39476 | 9.47E-07 |
| RB1 | 0.205482 | 0.715492 | 0.62051 | 0.696308 | 0.600873 | 9.58E-07 |
| ZNF414 | 0.311648 | 0.364628 | 0.293788 | 0.422209 | 0.324891 | 1.08E-06 |
| SELENOT | 0.287218 | 0.414865 | 0.339974 | 0.488134 | 0.386026 | 1.13E-06 |
| IBA57 | 0.523568 | 0.191529 | 0.133237 | 0.241137 | 0.166812 | 1.18E-06 |
| GYG1 | 0.329692 | 0.359402 | 0.285978 | 0.430999 | 0.334498 | 1.2E-06 |
| PRPS1 | 0.430644 | 0.171436 | 0.127193 | 0.228684 | 0.152838 | 1.26E-06 |
| CCDC47 | 0.263442 | 0.495141 | 0.412501 | 0.545415 | 0.439301 | 1.29E-06 |
| HCST | 0.134395 | 1.42162 | 1.295169 | 0.90126 | 0.841048 | 1.29E-06 |
| TRPV4 | 1.659682 | 0.034375 | 0.01088 | 0.044243 | 0.011354 | 1.32E-06 |
| DDAH2 | 0.298918 | 0.440633 | 0.358174 | 0.463522 | 0.368559 | 1.35E-06 |
| CXXC5 | 0.397258 | 0.281896 | 0.214044 | 0.322444 | 0.237555 | 1.41E-06 |
| DNAJC25 | 0.481598 | 0.190592 | 0.136499 | 0.230589 | 0.156332 | 1.59E-06 |
| RTN4 | 0.122918 | 1.309852 | 1.202875 | 0.910929 | 0.857642 | 1.6E-06 |
| NFKBIB | 0.343102 | 0.325457 | 0.256572 | 0.388954 | 0.296943 | 1.66E-06 |
| SH3RF3 | 1.222452 | 0.052828 | 0.02264 | 0.069587 | 0.028821 | 1.68E-06 |
| HEATR3 | 0.289852 | 0.425964 | 0.348433 | 0.474363 | 0.377293 | 1.72E-06 |
| HNRNPH2 | 0.155941 | 0.985611 | 0.884631 | 0.814826 | 0.751092 | 1.73E-06 |
| AGO2 | 0.216834 | 0.701785 | 0.603852 | 0.675212 | 0.587773 | 1.73E-06 |
| TMEM35B | 0.362002 | 0.317561 | 0.247089 | 0.376355 | 0.287336 | 1.88E-06 |
| MLXIP | 0.245139 | 0.5799 | 0.489282 | 0.613536 | 0.524891 | 1.92E-06 |
| RNPEP | 0.138471 | 1.13447 | 1.030645 | 0.848667 | 0.795633 | 1.92E-06 |
| SLC2A14 | 1.099327 | 0.050653 | 0.023642 | 0.073689 | 0.031441 | 1.93E-06 |
| DGCR2 | 0.22409 | 0.643315 | 0.550764 | 0.647964 | 0.545852 | 1.94E-06 |
| MON2 | 0.318229 | 0.331044 | 0.265515 | 0.401113 | 0.303057 | 1.96E-06 |
| CWC25 | 0.303226 | 0.334974 | 0.271476 | 0.399355 | 0.303057 | 1.98E-06 |
| TINF2 | 0.202383 | 0.749065 | 0.651023 | 0.701436 | 0.606114 | 2.01E-06 |
| ATXN7 | 0.230668 | 0.584175 | 0.497858 | 0.606065 | 0.505677 | 2.16E-06 |
| ARMCX3 | 0.413734 | 0.216353 | 0.162411 | 0.268239 | 0.188646 | 2.17E-06 |
| IMPA2 | 0.150325 | 1.059156 | 0.95435 | 0.82552 | 0.758952 | 2.2E-06 |
| NDUFB9 | 0.168361 | 1.020186 | 0.907816 | 0.803985 | 0.729258 | 2.35E-06 |
| KLHL15 | 0.308191 | 0.373458 | 0.301625 | 0.411368 | 0.318777 | 2.37E-06 |
| HAPSTR1 | 0.139501 | 1.161323 | 1.054288 | 0.882215 | 0.820087 | 2.43E-06 |
| CDK20 | 0.541244 | 0.134888 | 0.092692 | 0.17111 | 0.108297 | 2.57E-06 |
| SOCS1 | 0.466495 | 0.154267 | 0.111646 | 0.1799 | 0.11441 | 2.59E-06 |
| SERTAD1 | 0.211722 | 0.789687 | 0.681899 | 0.684002 | 0.587773 | 2.62E-06 |
| FOXO3 | 0.211826 | 0.717463 | 0.619489 | 0.679754 | 0.596507 | 2.62E-06 |
| ATP8B4 | 0.411402 | 0.200585 | 0.150818 | 0.244506 | 0.166812 | 2.69E-06 |
| PLAC8 | 0.282701 | 0.723098 | 0.594423 | 0.591708 | 0.518777 | 2.71E-06 |
| CANT1 | 0.267341 | 0.492875 | 0.409505 | 0.543803 | 0.445415 | 2.71E-06 |
| ZNF438 | 0.470585 | 0.196471 | 0.141788 | 0.239525 | 0.165939 | 2.78E-06 |
| SEPHS2 | 0.363135 | 0.329558 | 0.256223 | 0.393495 | 0.30655 | 3.19E-06 |
| NUP98 | 0.216191 | 0.580267 | 0.499515 | 0.611778 | 0.517031 | 3.2E-06 |
| SUSD3 | 0.526502 | 0.165756 | 0.115074 | 0.204512 | 0.137118 | 3.32E-06 |
| DYNC1LI2 | 0.25043 | 0.502638 | 0.422541 | 0.544829 | 0.454148 | 3.39E-06 |
| PLEKHB2 | 0.146284 | 1.204516 | 1.08837 | 0.869323 | 0.798253 | 3.41E-06 |
| TUBA1A | 0.248093 | 0.546697 | 0.460324 | 0.567829 | 0.468122 | 3.44E-06 |
| LENG9 | 0.689584 | 0.095881 | 0.059449 | 0.119689 | 0.067249 | 3.47E-06 |
| IRS1 | 1.567409 | 0.035104 | 0.011845 | 0.047759 | 0.014847 | 3.5E-06 |
| RAB24 | 0.17898 | 0.674964 | 0.596214 | 0.658951 | 0.567686 | 3.64E-06 |
| SURF4 | 0.224197 | 0.669417 | 0.573069 | 0.654996 | 0.572052 | 3.64E-06 |
| PTTG1IP | 0.127993 | 1.220508 | 1.116891 | 0.879871 | 0.827948 | 3.72E-06 |
| FDPS | 0.408927 | 0.207458 | 0.156254 | 0.276443 | 0.199127 | 4.03E-06 |
| USP9X | 0.196033 | 0.715812 | 0.624866 | 0.695429 | 0.598253 | 4.2E-06 |
| STING1 | 0.146413 | 1.254217 | 1.133177 | 0.860826 | 0.790393 | 4.33E-06 |
| TOLLIP | 0.31065 | 0.390631 | 0.314958 | 0.448872 | 0.365066 | 4.48E-06 |
| ABCG1 | 0.647794 | 0.105405 | 0.067275 | 0.12804 | 0.074236 | 4.49E-06 |
| GNA13 | 0.140676 | 1.231155 | 1.116773 | 0.886171 | 0.823581 | 4.55E-06 |
| LRRC4 | 1.322908 | 0.044289 | 0.017704 | 0.057574 | 0.021834 | 4.78E-06 |
| PTX3 | 0.379118 | 0.403407 | 0.310183 | 0.304571 | 0.227074 | 5E-06 |
| TP53I11 | 0.417779 | 0.230916 | 0.172858 | 0.280838 | 0.20524 | 5.1E-06 |
| SWAP70 | 0.270312 | 0.464144 | 0.38484 | 0.497363 | 0.40786 | 5.2E-06 |
| RTN4RL2 | 0.752506 | 0.120366 | 0.071446 | 0.12467 | 0.073362 | 5.5E-06 |
| UBXN4 | 0.198225 | 0.742696 | 0.64735 | 0.703487 | 0.600873 | 5.52E-06 |
| SLC6A6 | 0.128293 | 1.222482 | 1.118464 | 0.888368 | 0.827948 | 5.64E-06 |
| CPT1A | 0.266394 | 0.4442 | 0.369306 | 0.498242 | 0.40524 | 5.69E-06 |
| TRIM11 | 0.423678 | 0.186268 | 0.138867 | 0.235863 | 0.164192 | 6.02E-06 |
| ETF1 | 0.194519 | 0.719307 | 0.628577 | 0.697773 | 0.613974 | 6.18E-06 |
| USP32 | 0.167175 | 0.939054 | 0.836307 | 0.797539 | 0.70393 | 6.25E-06 |
| CSNK1E | 0.438004 | 0.185094 | 0.136628 | 0.242162 | 0.168559 | 6.35E-06 |
| CALHM6 | 0.260179 | 0.504562 | 0.421301 | 0.502637 | 0.41048 | 6.55E-06 |
| VPS37A | 0.376362 | 0.218456 | 0.168294 | 0.278787 | 0.199127 | 6.65E-06 |
| SMDT1 | 0.173571 | 0.894887 | 0.793447 | 0.768093 | 0.68559 | 6.69E-06 |
| INAFM1 | 0.420183 | 0.226218 | 0.169059 | 0.278054 | 0.203493 | 6.7E-06 |
| BATF | 0.359185 | 0.200915 | 0.156634 | 0.241137 | 0.167686 | 6.79E-06 |
| CRTC3 | 0.220904 | 0.569764 | 0.488874 | 0.594052 | 0.489083 | 7.12E-06 |
| ANPEP | 0.165027 | 1.212106 | 1.081091 | 0.804571 | 0.736245 | 7.13E-06 |
| HIP1 | 0.402936 | 0.287272 | 0.217269 | 0.323616 | 0.245415 | 7.15E-06 |
| COMMD4 | 0.443346 | 0.191635 | 0.140933 | 0.246264 | 0.174672 | 7.36E-06 |
| SRSF2 | 0.139282 | 1.019864 | 0.926007 | 0.824788 | 0.748472 | 7.71E-06 |
| SCYL1 | 0.224293 | 0.638247 | 0.546349 | 0.640199 | 0.542358 | 7.73E-06 |
| RAB1A | 0.171639 | 0.816503 | 0.724919 | 0.762819 | 0.684716 | 7.84E-06 |
| RGCC | 0.227264 | 1.132926 | 0.967805 | 0.62467 | 0.562445 | 7.93E-06 |
| SCML1 | 0.510342 | 0.132079 | 0.092727 | 0.173747 | 0.11179 | 7.94E-06 |
| F11R | 0.263514 | 0.493975 | 0.411509 | 0.508936 | 0.412227 | 8.03E-06 |
| KCNK13 | 0.671385 | 0.110492 | 0.069378 | 0.144594 | 0.089956 | 8.17E-06 |
| TNKS | 0.293321 | 0.318672 | 0.260043 | 0.37533 | 0.284716 | 8.34E-06 |
| FKBP11 | 0.588951 | 0.141213 | 0.093882 | 0.184735 | 0.122271 | 8.44E-06 |
| PSMB3 | 0.275219 | 0.422787 | 0.34936 | 0.504395 | 0.4131 | 8.46E-06 |
| RNF167 | 0.21252 | 0.671588 | 0.5796 | 0.660856 | 0.578166 | 8.72E-06 |
| PIM2 | 0.381307 | 0.331648 | 0.25462 | 0.363903 | 0.286463 | 8.82E-06 |
| MAFG | 0.19503 | 0.737356 | 0.644121 | 0.6862 | 0.606114 | 8.88E-06 |
| SNCA | 0.318398 | 0.329251 | 0.264046 | 0.361119 | 0.275109 | 8.99E-06 |
| NUP58 | 0.20828 | 0.673606 | 0.583052 | 0.657779 | 0.563319 | 9.32E-06 |
| FRAT1 | 0.255163 | 0.42807 | 0.358677 | 0.459859 | 0.366812 | 9.41E-06 |
| DHCR7 | 0.692112 | 0.098464 | 0.060944 | 0.121447 | 0.070742 | 9.43E-06 |
| SPATA2L | 0.326296 | 0.204071 | 0.162763 | 0.246411 | 0.173799 | 9.92E-06 |
| BRCA2 | 1.240335 | 0.051001 | 0.021587 | 0.065485 | 0.028821 | 1.09E-05 |
| ATP11B | 0.160849 | 0.942624 | 0.843176 | 0.798564 | 0.727511 | 1.1E-05 |
| CREB5 | 0.231115 | 0.659666 | 0.56202 | 0.608702 | 0.532751 | 1.12E-05 |
| PPP1R11 | 0.201928 | 0.691465 | 0.601152 | 0.686053 | 0.599127 | 1.12E-05 |
| EBPL | 0.480295 | 0.14987 | 0.107431 | 0.20041 | 0.137118 | 1.13E-05 |
| RELA | 0.237752 | 0.504932 | 0.428215 | 0.554351 | 0.456769 | 1.15E-05 |
| MPHOSPH6 | 0.398658 | 0.186023 | 0.141111 | 0.249634 | 0.177293 | 1.15E-05 |
| DUSP28 | 0.327967 | 0.269719 | 0.214874 | 0.32303 | 0.244541 | 1.18E-05 |
| TMTC1 | 2.117677 | 0.023012 | 0.005302 | 0.031351 | 0.006114 | 1.18E-05 |
| MCM8 | 1.015124 | 0.056616 | 0.028013 | 0.074861 | 0.035808 | 1.25E-05 |
| DNAJB11 | 0.196736 | 0.690112 | 0.602138 | 0.657779 | 0.584279 | 1.26E-05 |
| CITED2 | 0.208742 | 0.86436 | 0.747923 | 0.659537 | 0.579913 | 1.28E-05 |
| ABL2 | 0.506551 | 0.146737 | 0.103288 | 0.190741 | 0.128384 | 1.3E-05 |
| PHYKPL | 0.189034 | 0.626188 | 0.549288 | 0.632874 | 0.540611 | 1.32E-05 |
| FAM89B | 0.1596 | 0.970104 | 0.868508 | 0.780105 | 0.712664 | 1.39E-05 |
| BLOC1S4 | 0.412586 | 0.179797 | 0.135077 | 0.222824 | 0.155459 | 1.41E-05 |
| TXNDC11 | 0.137785 | 1.199641 | 1.09037 | 0.862877 | 0.796507 | 1.42E-05 |
| ATP5F1B | 0.194539 | 0.710323 | 0.620718 | 0.693671 | 0.606114 | 1.51E-05 |
| HSD3B7 | 1.260652 | 0.038745 | 0.01617 | 0.05274 | 0.020087 | 1.51E-05 |
| DENND4B | 0.145005 | 0.963034 | 0.870945 | 0.817902 | 0.742358 | 1.54E-05 |
| DENND2D | 0.416774 | 0.209582 | 0.156997 | 0.263698 | 0.19214 | 1.54E-05 |
| GM2A | 0.181791 | 0.810583 | 0.714616 | 0.707296 | 0.629694 | 1.58E-05 |
| RYBP | 0.132099 | 1.290913 | 1.177962 | 0.88954 | 0.820087 | 1.63E-05 |
| ZBTB43 | 0.498289 | 0.148119 | 0.10486 | 0.201143 | 0.137118 | 1.63E-05 |
| REXO1 | 0.411377 | 0.220876 | 0.166078 | 0.286844 | 0.212227 | 1.66E-05 |
| TGIF1 | 0.214972 | 0.694959 | 0.598751 | 0.614122 | 0.532751 | 1.77E-05 |
| TMBIM6 | 0.212582 | 0.642361 | 0.554352 | 0.664518 | 0.567686 | 1.78E-05 |
| SLC25A20 | 0.288487 | 0.322587 | 0.264122 | 0.381776 | 0.29869 | 1.8E-05 |
| HNRNPLL | 0.345005 | 0.282105 | 0.222103 | 0.336068 | 0.257642 | 1.93E-05 |
| CRACR2B | 1.255184 | 0.037163 | 0.015569 | 0.048345 | 0.017467 | 1.93E-05 |
| TMEM51 | 1.371258 | 0.033916 | 0.01311 | 0.048345 | 0.017467 | 1.98E-05 |
| TPK1 | 0.367486 | 0.269999 | 0.209285 | 0.322737 | 0.244541 | 1.99E-05 |
| SLC46A2 | 0.328452 | 0.336316 | 0.267839 | 0.364928 | 0.28559 | 2.01E-05 |
| BCL2L11 | 0.238805 | 0.513677 | 0.435314 | 0.526956 | 0.442795 | 2.06E-05 |
| CDC25B | 0.582982 | 0.12229 | 0.081638 | 0.169206 | 0.11179 | 2.09E-05 |
| CRK | 0.183974 | 0.699934 | 0.616135 | 0.682244 | 0.587773 | 2.16E-05 |
| XPO6 | 0.222256 | 0.594478 | 0.509601 | 0.621154 | 0.536245 | 2.19E-05 |
| CYSTM1 | 0.285777 | 0.384245 | 0.315196 | 0.416203 | 0.328384 | 2.22E-05 |
| DDX21 | 0.141628 | 1.08688 | 0.985252 | 0.824788 | 0.767686 | 2.32E-05 |
| KLF9 | 0.156747 | 0.933708 | 0.837579 | 0.746411 | 0.660262 | 2.33E-05 |
| IL4R | 0.341781 | 0.217234 | 0.171412 | 0.278347 | 0.203493 | 2.37E-05 |
| METTL7B | 5.731376 | 0.014293 | 0.000269 | 0.020363 | 0.000873 | 2.37E-05 |
| USP36 | 0.272186 | 0.470337 | 0.389469 | 0.505713 | 0.421834 | 2.41E-05 |
| MYO1C | 0.602472 | 0.101357 | 0.066757 | 0.135072 | 0.083843 | 2.52E-05 |
| ERI1 | 0.486242 | 0.144438 | 0.103112 | 0.189276 | 0.128384 | 2.54E-05 |
| SAMD4B | 0.238211 | 0.435881 | 0.369538 | 0.495166 | 0.4 | 2.56E-05 |
| ARFGEF2 | 0.274989 | 0.38682 | 0.31969 | 0.454292 | 0.366812 | 2.63E-05 |
| POFUT2 | 0.382503 | 0.201589 | 0.15464 | 0.258863 | 0.187773 | 2.65E-05 |
| RAB7A | 0.159185 | 0.883314 | 0.791035 | 0.800176 | 0.717031 | 2.68E-05 |
| NXF1 | 0.123185 | 1.115373 | 1.024089 | 0.86361 | 0.80262 | 2.7E-05 |
| TEDC1 | 0.200925 | 0.824511 | 0.717318 | 0.643569 | 0.572052 | 2.83E-05 |
| ANXA3 | 3.442739 | 0.021206 | 0.00195 | 0.02344 | 0.00262 | 2.84E-05 |
| GTPBP1 | 0.21302 | 0.610121 | 0.526369 | 0.611925 | 0.540611 | 2.89E-05 |
| PSMC4 | 0.268422 | 0.408532 | 0.339174 | 0.474949 | 0.384279 | 2.91E-05 |
| INAFM2 | 0.314433 | 0.304459 | 0.244836 | 0.362731 | 0.28559 | 2.94E-05 |
| BAZ1A | 0.218903 | 0.541144 | 0.464961 | 0.566217 | 0.477729 | 2.94E-05 |
| TMEM63B | 0.390278 | 0.178884 | 0.136486 | 0.2322 | 0.164192 | 3.02E-05 |
| AKAP17A | 0.144706 | 1.033445 | 0.934817 | 0.830501 | 0.747598 | 3.07E-05 |
| TP53I3 | 0.318204 | 0.310769 | 0.249258 | 0.346176 | 0.268122 | 3.11E-05 |
| TPST2 | 0.23906 | 0.474841 | 0.402332 | 0.523586 | 0.433188 | 3.16E-05 |
| KRAS | 0.231013 | 0.514035 | 0.437977 | 0.556109 | 0.455895 | 3.2E-05 |
| MAN2B2 | 0.234732 | 0.468994 | 0.398571 | 0.514943 | 0.422707 | 3.35E-05 |
| B3GALT6 | 0.449731 | 0.15116 | 0.110677 | 0.199678 | 0.137991 | 3.41E-05 |
| CIAO3 | 0.482998 | 0.132057 | 0.094485 | 0.173161 | 0.115284 | 3.62E-05 |
| IFITM10 | 0.598676 | 0.115205 | 0.076077 | 0.144741 | 0.09345 | 3.84E-05 |
| CLSTN3 | 0.407944 | 0.114131 | 0.08602 | 0.147085 | 0.09345 | 3.92E-05 |
| PEAK3 | 0.465191 | 0.147858 | 0.107105 | 0.192939 | 0.133624 | 4.4E-05 |
| SLC16A6 | 0.295658 | 0.405607 | 0.330448 | 0.415031 | 0.341485 | 4.45E-05 |
| UBN1 | 0.28183 | 0.389279 | 0.320201 | 0.448286 | 0.364192 | 4.48E-05 |
| LAX1 | 1.300251 | 0.041192 | 0.016726 | 0.053912 | 0.022707 | 4.56E-05 |
| CFAP410 | 0.445832 | 0.146552 | 0.107593 | 0.19499 | 0.134498 | 4.61E-05 |
| UBA6 | 0.270157 | 0.375379 | 0.311275 | 0.440961 | 0.357205 | 4.7E-05 |
| DNAJB14 | 0.373808 | 0.189758 | 0.146445 | 0.245239 | 0.178166 | 4.89E-05 |
| ZBTB47 | 0.303382 | 0.301878 | 0.244627 | 0.349253 | 0.272489 | 5.18E-05 |
| ANXA2R | 0.330258 | 0.197607 | 0.157175 | 0.233665 | 0.167686 | 5.2E-05 |
| SDF2L1 | 0.318677 | 0.308472 | 0.247334 | 0.359947 | 0.284716 | 5.4E-05 |
| RAB5IF | 0.138322 | 0.995394 | 0.904391 | 0.829915 | 0.758952 | 5.42E-05 |
| HIC1 | 0.572323 | 0.163763 | 0.110136 | 0.175798 | 0.123144 | 5.51E-05 |
| CDC40 | 0.246114 | 0.462838 | 0.390248 | 0.513185 | 0.433188 | 5.58E-05 |
| DMPK | 1.104593 | 0.047994 | 0.022319 | 0.064313 | 0.030568 | 5.67E-05 |
| TIMM10B | 0.260265 | 0.360726 | 0.301183 | 0.40419 | 0.322271 | 5.68E-05 |
| NARF | 0.231473 | 0.483758 | 0.412048 | 0.525051 | 0.436681 | 5.84E-05 |
| PSMD12 | 0.207156 | 0.576095 | 0.499039 | 0.601084 | 0.517904 | 5.91E-05 |
| PPP2CB | 0.448612 | 0.110413 | 0.080905 | 0.157486 | 0.103057 | 5.92E-05 |
| SNX21 | 0.30072 | 0.187643 | 0.152338 | 0.229563 | 0.162445 | 6.12E-05 |
| SIGLEC9 | 0.151958 | 0.927653 | 0.834914 | 0.784207 | 0.69869 | 6.39E-05 |
| ARPC5L | 0.285778 | 0.335326 | 0.275068 | 0.391737 | 0.30917 | 6.44E-05 |
| USP53 | 3.205968 | 0.015585 | 0.001689 | 0.021975 | 0.00262 | 6.63E-05 |
| TET2 | 0.155002 | 0.791967 | 0.71129 | 0.73806 | 0.661135 | 6.75E-05 |
| MAP1S | 0.206452 | 0.618518 | 0.536048 | 0.611485 | 0.530131 | 6.83E-05 |
| APRT | 0.154196 | 0.87707 | 0.788164 | 0.757252 | 0.683843 | 7.02E-05 |
| TUSC2 | 0.308477 | 0.27096 | 0.218799 | 0.331087 | 0.256769 | 7.06E-05 |
| RNASEH2C | 0.317942 | 0.284484 | 0.228217 | 0.351304 | 0.273362 | 7.06E-05 |
| ETFDH | 0.464036 | 0.118825 | 0.086143 | 0.163786 | 0.108297 | 7.25E-05 |
| TUFM | 0.128814 | 1.043683 | 0.954534 | 0.826399 | 0.765066 | 7.25E-05 |
| ARL3 | 0.355001 | 0.196462 | 0.153608 | 0.253296 | 0.185153 | 7.39E-05 |
| TAGLN2 | 0.155168 | 1.062527 | 0.954179 | 0.79373 | 0.725764 | 7.39E-05 |
| VPS28 | 0.161923 | 0.782469 | 0.699396 | 0.728538 | 0.653275 | 7.51E-05 |
| SOCS4 | 0.292869 | 0.18822 | 0.15364 | 0.242162 | 0.173799 | 7.63E-05 |
| SLC22A13 | 1.59295 | 0.026093 | 0.00865 | 0.033695 | 0.009607 | 7.68E-05 |
| MORF4L2 | 0.40239 | 0.213954 | 0.161879 | 0.285672 | 0.217467 | 7.86E-05 |
| RIPOR2 | 0.109063 | 1.632947 | 1.514053 | 0.94228 | 0.89083 | 7.9E-05 |
| KMT2B | 0.205844 | 0.489775 | 0.42465 | 0.530472 | 0.446288 | 8.02E-05 |
| THAP2 | 0.241274 | 0.50157 | 0.424327 | 0.49121 | 0.411354 | 8.08E-05 |
| MAP3K7CL | 0.471479 | 0.179693 | 0.129599 | 0.196455 | 0.141485 | 8.67E-05 |
| SHISA5 | 0.114919 | 1.351533 | 1.248052 | 0.91547 | 0.859389 | 8.68E-05 |
| GAPVD1 | 0.24608 | 0.329417 | 0.27776 | 0.402285 | 0.319651 | 8.9E-05 |
| CRTC2 | 0.227239 | 0.51702 | 0.441674 | 0.566657 | 0.482969 | 8.96E-05 |
| BCKDK | 0.162202 | 0.734282 | 0.656198 | 0.689423 | 0.616594 | 9.32E-05 |
| GDI1 | 0.136538 | 0.907936 | 0.825949 | 0.791679 | 0.71441 | 9.32E-05 |
| ARFIP2 | 0.349457 | 0.191474 | 0.150284 | 0.242602 | 0.179039 | 9.5E-05 |
| MAP1LC3B | 0.156573 | 1.005045 | 0.90168 | 0.793437 | 0.71441 | 9.55E-05 |
| KNDC1 | 2.357755 | 0.019041 | 0.003715 | 0.024465 | 0.004367 | 9.65E-05 |
| MYC | 0.486579 | 0.132747 | 0.094744 | 0.165837 | 0.11441 | 9.72E-05 |
| RNF146 | 0.266237 | 0.378818 | 0.314982 | 0.426311 | 0.348472 | 9.96E-05 |
| HTT | 0.194918 | 0.60755 | 0.530769 | 0.622473 | 0.532751 | 0.000114 |
| AGPAT4 | 0.221987 | 0.519244 | 0.445191 | 0.506739 | 0.423581 | 0.000115 |
| GAB2 | 0.157526 | 0.80221 | 0.71923 | 0.734398 | 0.648908 | 0.00014 |
| ETFB | 0.196028 | 0.602056 | 0.525565 | 0.603575 | 0.521397 | 0.000141 |
| SERTAD2 | 0.146525 | 0.980623 | 0.885918 | 0.770144 | 0.694323 | 0.000143 |
| ECD | 0.273588 | 0.279026 | 0.230827 | 0.350278 | 0.275983 | 0.00016 |
| SCN1B | 1.99565 | 0.021191 | 0.005314 | 0.0293 | 0.00786 | 0.000164 |
| ZBTB48 | 0.234601 | 0.310534 | 0.263929 | 0.368005 | 0.287336 | 0.000168 |
| AURKAIP1 | 0.176698 | 0.580784 | 0.513834 | 0.603282 | 0.513537 | 0.000171 |
| LTBR | 0.134795 | 1.205419 | 1.097894 | 0.876648 | 0.793013 | 0.000174 |
| RFX1 | 0.241487 | 0.419964 | 0.355236 | 0.451509 | 0.372052 | 0.000175 |
| SNRPB | 0.143315 | 0.722267 | 0.653966 | 0.700264 | 0.630568 | 0.000175 |
| PTPN18 | 0.25564 | 0.381183 | 0.319285 | 0.427044 | 0.346725 | 0.000176 |
| ABI1 | 0.16615 | 0.661347 | 0.589404 | 0.665837 | 0.577293 | 0.000177 |
| CXCR1 | 1.660352 | 0.029552 | 0.009349 | 0.031937 | 0.009607 | 0.000181 |
| ARL16 | 0.364996 | 0.157255 | 0.122104 | 0.208175 | 0.149345 | 0.000181 |
| VEGFA | 0.183817 | 0.938161 | 0.82593 | 0.690888 | 0.630568 | 0.000183 |
| CARS1 | 0.43027 | 0.106504 | 0.079039 | 0.149575 | 0.099563 | 0.000187 |
| EGLN2 | 0.182369 | 0.655584 | 0.577737 | 0.649429 | 0.572052 | 0.000192 |
| DNM2 | 0.094252 | 1.404318 | 1.315506 | 0.926897 | 0.875983 | 0.000194 |
| CPSF1 | 0.315964 | 0.200437 | 0.161014 | 0.26487 | 0.198253 | 0.000197 |
| MAU2 | 0.371522 | 0.143186 | 0.110678 | 0.194404 | 0.136245 | 0.000197 |
| SH3GL1 | 0.224629 | 0.459523 | 0.393267 | 0.512745 | 0.430568 | 0.000206 |
| MUL1 | 0.375632 | 0.156924 | 0.120952 | 0.207735 | 0.149345 | 0.000212 |
| MIF | 0.186679 | 0.617804 | 0.542818 | 0.643862 | 0.566812 | 0.000217 |
| SLC19A2 | 0.296297 | 0.324206 | 0.264014 | 0.355845 | 0.286463 | 0.000225 |
| TXNRD2 | 0.166117 | 0.659438 | 0.587716 | 0.637709 | 0.544978 | 0.000226 |
| SLC9A9 | 0.31856 | 0.20412 | 0.163678 | 0.262819 | 0.198253 | 0.000227 |
| CRLS1 | 0.232669 | 0.444087 | 0.377944 | 0.483299 | 0.406114 | 0.000234 |
| IFI27L2 | 0.198274 | 0.586363 | 0.511069 | 0.589364 | 0.51179 | 0.000236 |
| NDUFB3 | 0.240138 | 0.37187 | 0.314849 | 0.456636 | 0.372052 | 0.000239 |
| P2RY2 | 0.295874 | 0.276752 | 0.225436 | 0.300029 | 0.234061 | 0.000245 |
| MTMR6 | 0.182237 | 0.629832 | 0.555093 | 0.623205 | 0.549345 | 0.000245 |
| DHX34 | 0.24762 | 0.358994 | 0.302375 | 0.412394 | 0.334498 | 0.000253 |
| PER2 | 0.297303 | 0.272903 | 0.22208 | 0.323323 | 0.254148 | 0.000255 |
| C12orf60 | 2.319797 | 0.017304 | 0.003466 | 0.024172 | 0.00524 | 0.000255 |
| TMC6 | 0.215513 | 0.462578 | 0.39839 | 0.50586 | 0.425328 | 0.000264 |
| ZNF503 | 0.337575 | 0.198228 | 0.156872 | 0.216379 | 0.160699 | 0.000264 |
| ITPRIP | 0.136247 | 0.965881 | 0.878839 | 0.77659 | 0.71441 | 0.00027 |
| H2AZ1 | 0.223558 | 0.471173 | 0.403536 | 0.53179 | 0.453275 | 0.000274 |
| AFF4 | 0.194917 | 0.514733 | 0.449682 | 0.559771 | 0.476856 | 0.000274 |
| SIGLEC7 | 0.227395 | 0.376165 | 0.321311 | 0.428216 | 0.350218 | 0.000288 |
| SLC8B1 | 0.276187 | 0.284227 | 0.234706 | 0.344272 | 0.272489 | 0.000289 |
| PGAM1 | 0.335359 | 0.186572 | 0.147875 | 0.254322 | 0.190393 | 0.000291 |
| CTNNB1 | 0.173573 | 0.681009 | 0.603812 | 0.663786 | 0.58952 | 0.000292 |
| TMEM87B | 0.315199 | 0.248353 | 0.199611 | 0.308526 | 0.241048 | 0.000298 |
| KIFC3 | 2.056868 | 0.021925 | 0.005269 | 0.028128 | 0.00786 | 0.000301 |
| MFSD10 | 0.207768 | 0.540229 | 0.467772 | 0.561822 | 0.482969 | 0.000308 |
| GHITM | 0.128314 | 0.98274 | 0.899109 | 0.815705 | 0.749345 | 0.000309 |
| RCBTB1 | 0.314158 | 0.272605 | 0.219262 | 0.32552 | 0.259389 | 0.000316 |
| PCSK5 | 0.313688 | 0.265485 | 0.213605 | 0.310284 | 0.247162 | 0.000317 |
| SELENOO | 0.207565 | 0.417207 | 0.361301 | 0.470993 | 0.390393 | 0.000322 |
| GGA1 | 0.236016 | 0.405812 | 0.344569 | 0.449165 | 0.374672 | 0.000323 |
| NOTCH1 | 0.126444 | 0.977362 | 0.895348 | 0.813507 | 0.731004 | 0.000326 |
| ENDOG | 0.33062 | 0.239723 | 0.190626 | 0.289921 | 0.227948 | 0.000331 |
| SLC5A6 | 0.270481 | 0.34436 | 0.28549 | 0.384559 | 0.31179 | 0.000336 |
| EFCAB2 | 0.23807 | 0.373829 | 0.316962 | 0.40375 | 0.325764 | 0.000337 |
| KRT10-AS1 | 1.50071 | 0.023433 | 0.008281 | 0.03472 | 0.012227 | 0.000338 |
| ARPP19 | 0.239237 | 0.287262 | 0.243366 | 0.361559 | 0.284716 | 0.000343 |
| HRK | 4.53678 | 0.012482 | 0.000538 | 0.015675 | 0.000873 | 0.000349 |
| PEF1 | 0.268261 | 0.308812 | 0.256413 | 0.382069 | 0.308297 | 0.000353 |
| SLC31A2 | 0.116423 | 1.282278 | 1.182866 | 0.881483 | 0.824454 | 0.000365 |
| HSPA5 | 0.163361 | 0.74343 | 0.663841 | 0.684002 | 0.613974 | 0.000371 |
| TECPR2 | 0.285637 | 0.221452 | 0.181675 | 0.268679 | 0.204367 | 0.000371 |
| TERF2IP | 0.163493 | 0.535438 | 0.478071 | 0.567829 | 0.487336 | 0.000373 |
| SYNE1 | 0.317137 | 0.233102 | 0.187102 | 0.237914 | 0.179913 | 0.000374 |
| XRCC5 | 0.251974 | 0.279021 | 0.234307 | 0.364342 | 0.287336 | 0.000375 |
| MAN2A2 | 0.147739 | 0.824079 | 0.743867 | 0.754908 | 0.654148 | 0.000379 |
| PPARD | 0.237947 | 0.251442 | 0.21321 | 0.306329 | 0.236681 | 0.000383 |
| ROCK1 | 0.11715 | 1.001143 | 0.923061 | 0.826253 | 0.764192 | 0.000388 |
| FRMD8 | 0.29713 | 0.125916 | 0.10248 | 0.170817 | 0.118777 | 0.00039 |
| WBP2 | 0.179859 | 0.621517 | 0.548669 | 0.650747 | 0.561572 | 0.000394 |
| YIPF1 | 0.301876 | 0.261173 | 0.211863 | 0.328011 | 0.261135 | 0.000399 |
| TMTC2 | 0.227467 | 0.300223 | 0.256431 | 0.353062 | 0.281223 | 0.000404 |
| U2AF2 | 0.25245 | 0.302321 | 0.253789 | 0.386903 | 0.310044 | 0.000415 |
| SERINC2 | 0.327742 | 0.182775 | 0.145632 | 0.222824 | 0.163319 | 0.000415 |
| ZNF467 | 0.136198 | 0.866442 | 0.788387 | 0.748315 | 0.672489 | 0.000419 |
| SH3GLB2 | 0.225255 | 0.324097 | 0.277246 | 0.383973 | 0.308297 | 0.000435 |
| RNF10 | 0.135358 | 0.974307 | 0.887052 | 0.81087 | 0.738865 | 0.000437 |
| NSMAF | 0.242908 | 0.36095 | 0.305017 | 0.423821 | 0.350218 | 0.000439 |
| SMG1 | 0.330052 | 0.188888 | 0.150262 | 0.252417 | 0.18952 | 0.000439 |
| COLGALT1 | 0.110858 | 1.16744 | 1.081093 | 0.870495 | 0.808734 | 0.000441 |
| SCAPER | 0.264894 | 0.172876 | 0.143878 | 0.223117 | 0.163319 | 0.000442 |
| BLOC1S6 | 0.215181 | 0.435829 | 0.37544 | 0.503223 | 0.417467 | 0.000442 |
| SNAPC1 | 0.364632 | 0.166652 | 0.129433 | 0.20671 | 0.152838 | 0.000456 |
| BECN1 | 0.20975 | 0.412339 | 0.356544 | 0.481981 | 0.39738 | 0.00047 |
| MTHFD2 | 0.181228 | 0.568134 | 0.501067 | 0.569733 | 0.49083 | 0.000473 |
| DGKA | 0.185315 | 0.439856 | 0.386834 | 0.488427 | 0.412227 | 0.000474 |
| RPRD1B | 0.340131 | 0.174753 | 0.13805 | 0.233519 | 0.174672 | 0.000481 |
| APMAP | 0.20962 | 0.46811 | 0.404805 | 0.524465 | 0.442795 | 0.000494 |
| SLC39A7 | 0.239479 | 0.398129 | 0.337236 | 0.451362 | 0.378166 | 0.000506 |
| RRAGA | 0.216533 | 0.391384 | 0.336837 | 0.448286 | 0.369432 | 0.000514 |
| NDUFS7 | 0.142052 | 0.858114 | 0.777648 | 0.755787 | 0.687336 | 0.000514 |
| GGH | 1.012316 | 0.02963 | 0.014689 | 0.042924 | 0.018341 | 0.000517 |
| CLHC1 | 1.755176 | 0.020361 | 0.006032 | 0.028421 | 0.008734 | 0.00054 |
| GOLGB1 | 0.214712 | 0.339594 | 0.292635 | 0.40961 | 0.329258 | 0.000547 |
| CPNE8 | 0.263441 | 0.261416 | 0.217785 | 0.326839 | 0.257642 | 0.000555 |
| GBA1 | 0.18997 | 0.536576 | 0.470375 | 0.571784 | 0.486463 | 0.000558 |
| ATP1B2 | 2.599977 | 0.013044 | 0.002152 | 0.018166 | 0.00262 | 0.000565 |
| LTB4R | 0.139914 | 0.902229 | 0.818839 | 0.736595 | 0.662009 | 0.000566 |
| VCPKMT | 0.28057 | 0.196607 | 0.16186 | 0.247583 | 0.1869 | 0.00058 |
| ACOX1 | 0.190834 | 0.475716 | 0.416775 | 0.517873 | 0.437555 | 0.000581 |
| CXCL8 | 0.110814 | 3.111923 | 2.881844 | 0.884706 | 0.80786 | 0.000587 |
| BTBD19 | 0.246132 | 0.391809 | 0.330355 | 0.441401 | 0.367686 | 0.000588 |
| NDUFC1 | 0.257097 | 0.333454 | 0.279024 | 0.397451 | 0.324891 | 0.000595 |
| MTX1 | 0.187948 | 0.478847 | 0.420358 | 0.521975 | 0.439301 | 0.000599 |
| PPP2CA | 0.175509 | 0.584496 | 0.517545 | 0.631556 | 0.551092 | 0.000605 |
| SLCO4A1 | 1.72045 | 0.01725 | 0.005235 | 0.025491 | 0.006987 | 0.000609 |
| SEH1L | 0.271888 | 0.292251 | 0.242052 | 0.351304 | 0.279476 | 0.00061 |
| PURB | 0.122727 | 1.075117 | 0.987441 | 0.819953 | 0.737991 | 0.000612 |
| DENND4A | 0.160849 | 0.722422 | 0.646206 | 0.677263 | 0.615721 | 0.000612 |
| HSD17B12 | 0.290397 | 0.154543 | 0.126367 | 0.219748 | 0.159825 | 0.000637 |
| PTPN12 | 0.126725 | 0.908286 | 0.831907 | 0.786844 | 0.719651 | 0.000638 |
| SLC39A3 | 0.301203 | 0.217736 | 0.176709 | 0.27615 | 0.214847 | 0.000638 |
| HPRT1 | 0.293077 | 0.242454 | 0.197881 | 0.313947 | 0.246288 | 0.000646 |
| ABCF2 | 0.256499 | 0.235038 | 0.196755 | 0.30545 | 0.238428 | 0.000664 |
| KDM6B | 0.104439 | 1.373785 | 1.277849 | 0.913566 | 0.856769 | 0.000671 |
| GOLGA2 | 0.264892 | 0.231747 | 0.192874 | 0.303106 | 0.235808 | 0.000672 |
| TSPAN4 | 0.39265 | 0.154068 | 0.117358 | 0.197334 | 0.146725 | 0.000676 |
| HSPA9 | 0.27724 | 0.239905 | 0.197962 | 0.316144 | 0.247162 | 0.000689 |
| RAB4B | 0.169257 | 0.657751 | 0.584938 | 0.643862 | 0.581659 | 0.000702 |
| MED22 | 0.24579 | 0.286626 | 0.241727 | 0.344711 | 0.275983 | 0.000724 |
| SCAF1 | 0.206988 | 0.440295 | 0.381447 | 0.505713 | 0.425328 | 0.000733 |
| IQCB1 | 0.328624 | 0.15201 | 0.121045 | 0.198945 | 0.145852 | 0.000735 |
| HSDL2 | 0.248499 | 0.244452 | 0.205773 | 0.302666 | 0.235808 | 0.00074 |
| CD69 | 0.271812 | 0.234908 | 0.194569 | 0.268825 | 0.20786 | 0.000743 |
| C1orf162 | 0.107027 | 1.293716 | 1.201214 | 0.891298 | 0.831441 | 0.000751 |
| TNFRSF10C | 0.365016 | 0.184716 | 0.143425 | 0.214035 | 0.162445 | 0.000753 |
| ARAP2 | 0.300759 | 0.188033 | 0.15265 | 0.239965 | 0.182533 | 0.000753 |
| DPF2 | 0.188617 | 0.392304 | 0.344226 | 0.448432 | 0.372052 | 0.000753 |
| ST14 | 0.186236 | 0.42464 | 0.373215 | 0.442866 | 0.363319 | 0.000762 |
| TFE3 | 0.142911 | 0.826284 | 0.748357 | 0.74436 | 0.674236 | 0.000776 |
| ASXL1 | 0.181247 | 0.548792 | 0.484002 | 0.576765 | 0.50393 | 0.000798 |
| FAM53C | 0.174143 | 0.633392 | 0.561371 | 0.619836 | 0.540611 | 0.000816 |
| B3GAT3 | 0.251229 | 0.243062 | 0.204216 | 0.312482 | 0.246288 | 0.000818 |
| ACVRL1 | 1.19998 | 0.027206 | 0.011842 | 0.040434 | 0.017467 | 0.00082 |
| LONRF1 | 0.353242 | 0.173697 | 0.135974 | 0.20671 | 0.155459 | 0.000828 |
| TSPAN17 | 0.245029 | 0.221594 | 0.186981 | 0.285526 | 0.221834 | 0.000836 |
| SNX20 | 0.146752 | 0.78841 | 0.712156 | 0.706124 | 0.618341 | 0.000836 |
| DSTYK | 0.327392 | 0.181791 | 0.144883 | 0.230296 | 0.174672 | 0.000847 |
| POP4 | 0.285097 | 0.233254 | 0.191428 | 0.299297 | 0.233188 | 0.000855 |
| ATP6V1C1 | 0.205158 | 0.391728 | 0.339802 | 0.456197 | 0.374672 | 0.000856 |
| UBE2H | 0.25343 | 0.334904 | 0.280951 | 0.408145 | 0.337991 | 0.000859 |
| DLC1 | 1.98577 | 0.017354 | 0.004382 | 0.023293 | 0.006114 | 0.000868 |
| HNRNPL | 0.120067 | 0.962439 | 0.885583 | 0.822004 | 0.754585 | 0.000872 |
| BCAP31 | 0.145174 | 0.778237 | 0.703737 | 0.723557 | 0.641921 | 0.000876 |
| TRAPPC5 | 0.106277 | 1.352036 | 1.256018 | 0.895253 | 0.840175 | 0.000879 |
| STAB1 | 0.099016 | 1.425974 | 1.331389 | 0.884999 | 0.827074 | 0.000917 |
| MVB12B | 0.217496 | 0.235813 | 0.202813 | 0.289481 | 0.225328 | 0.000918 |
| SDE2 | 0.190873 | 0.518752 | 0.454466 | 0.543071 | 0.469869 | 0.00092 |
| DUSP8 | 1.080253 | 0.023762 | 0.011238 | 0.032669 | 0.012227 | 0.000925 |
| ALCAM | 0.352011 | 0.168915 | 0.132343 | 0.217258 | 0.163319 | 0.000936 |
| LAMTOR5 | 0.224272 | 0.331181 | 0.2835 | 0.416203 | 0.335371 | 0.000952 |
| ATP6V1F | 0.120031 | 1.036819 | 0.954048 | 0.82596 | 0.762445 | 0.000953 |
| RNF11 | 0.16315 | 0.649822 | 0.580339 | 0.653677 | 0.565066 | 0.000977 |
| MRE11 | 0.323334 | 0.149525 | 0.119504 | 0.20378 | 0.151092 | 0.000999 |
| PICK1 | 0.340365 | 0.152964 | 0.120817 | 0.198066 | 0.147598 | 0.001017 |
| HNRNPK | 0.127758 | 0.947913 | 0.86758 | 0.818635 | 0.733624 | 0.001037 |
| VDAC2 | 0.185978 | 0.485688 | 0.426945 | 0.556988 | 0.468996 | 0.001038 |
| MED26 | 0.237021 | 0.276071 | 0.234245 | 0.333724 | 0.264629 | 0.001053 |
| IFT20 | 0.177191 | 0.576095 | 0.509512 | 0.566657 | 0.495197 | 0.001057 |
| ICAM5 | 1.039995 | 0.034197 | 0.016631 | 0.045708 | 0.021834 | 0.001078 |
| EVA1B | 0.315473 | 0.180179 | 0.14479 | 0.218137 | 0.165066 | 0.001091 |
| EXOC2 | 0.199155 | 0.268103 | 0.233534 | 0.336947 | 0.266376 | 0.001095 |
| WSB1 | 0.097192 | 1.223788 | 1.144059 | 0.886024 | 0.832314 | 0.001111 |
| DERL2 | 0.236739 | 0.348776 | 0.295992 | 0.40419 | 0.331878 | 0.001113 |
| HEATR5B | 0.214087 | 0.357229 | 0.307964 | 0.401992 | 0.332751 | 0.001116 |
| RBCK1 | 0.196054 | 0.400692 | 0.349778 | 0.458687 | 0.383406 | 0.001122 |
| NT5C2 | 0.149948 | 0.700623 | 0.63146 | 0.68576 | 0.59476 | 0.001158 |
| SNRNP27 | 0.276939 | 0.204111 | 0.168461 | 0.26194 | 0.203493 | 0.001164 |
| TMEM184B | 0.230903 | 0.359163 | 0.306043 | 0.419426 | 0.348472 | 0.001165 |
| PPP2R1A | 0.249266 | 0.15756 | 0.132559 | 0.22136 | 0.164192 | 0.001263 |
| NDUFA13 | 0.184543 | 0.511447 | 0.450036 | 0.577498 | 0.50655 | 0.001274 |
| OTULINL | 0.139868 | 0.812841 | 0.737736 | 0.721213 | 0.646288 | 0.001277 |
| RAD23A | 0.139267 | 0.741929 | 0.673657 | 0.692206 | 0.633188 | 0.001299 |
| ZNF207 | 0.213462 | 0.346294 | 0.298667 | 0.425139 | 0.351965 | 0.001311 |
| TUBB6 | 0.218876 | 0.432688 | 0.37178 | 0.442426 | 0.374672 | 0.001318 |
| RCOR1 | 0.127233 | 0.806066 | 0.738023 | 0.727366 | 0.668996 | 0.001366 |
| TMEM30A | 0.237662 | 0.272566 | 0.231168 | 0.360094 | 0.28821 | 0.001396 |
| ECI1 | 0.216774 | 0.392064 | 0.337366 | 0.442866 | 0.372052 | 0.001444 |
| GNB1 | 0.10384 | 1.373272 | 1.277902 | 0.916496 | 0.855895 | 0.001453 |
| SZT2 | 0.185049 | 0.147137 | 0.129424 | 0.197041 | 0.144105 | 0.001473 |
| PLPPR2 | 0.124513 | 1.082216 | 0.992732 | 0.830501 | 0.749345 | 0.001473 |
| SREBF2 | 0.128355 | 0.766467 | 0.701221 | 0.681365 | 0.603493 | 0.0015 |
| AKT2 | 0.185657 | 0.469354 | 0.412679 | 0.533695 | 0.455895 | 0.001507 |
| SPG7 | 0.168825 | 0.442571 | 0.393696 | 0.492236 | 0.420087 | 0.001558 |
| DNAJB1 | 0.130855 | 0.834512 | 0.762152 | 0.741576 | 0.68559 | 0.001568 |
| UBTD1 | 0.281852 | 0.209687 | 0.172474 | 0.268239 | 0.208734 | 0.00157 |
| AGPAT2 | 0.127304 | 0.880233 | 0.805889 | 0.746411 | 0.679476 | 0.001585 |
| STK17A | 0.279317 | 0.20235 | 0.166732 | 0.262086 | 0.203493 | 0.001654 |
| WDR45 | 0.20372 | 0.301972 | 0.262205 | 0.383534 | 0.308297 | 0.001656 |
| PLK2 | 0.283265 | 0.214436 | 0.176208 | 0.222531 | 0.171179 | 0.001659 |
| AP1G1 | 0.191663 | 0.406179 | 0.355649 | 0.468063 | 0.39476 | 0.001667 |
| ARID5A | 0.165895 | 0.544963 | 0.485767 | 0.576765 | 0.496943 | 0.001709 |
| ANAPC11 | 0.156321 | 0.592594 | 0.531741 | 0.611339 | 0.538865 | 0.001719 |
| CPSF4 | 0.306216 | 0.155762 | 0.125974 | 0.210812 | 0.158952 | 0.001753 |
| OAT | 0.240055 | 0.249686 | 0.211412 | 0.318342 | 0.254148 | 0.001854 |
| UBE2F | 0.226967 | 0.283656 | 0.242364 | 0.365954 | 0.295197 | 0.001872 |
| CARD19 | 0.126745 | 0.85034 | 0.778823 | 0.741137 | 0.678603 | 0.001874 |
| MCOLN1 | 0.166173 | 0.58053 | 0.51737 | 0.578963 | 0.49869 | 0.00188 |
| PREB | 0.195037 | 0.342332 | 0.299044 | 0.413126 | 0.338865 | 0.00188 |
| TTC19 | 0.189832 | 0.473508 | 0.415128 | 0.497803 | 0.431441 | 0.001898 |
| HNRNPM | 0.157043 | 0.648763 | 0.58185 | 0.66862 | 0.578166 | 0.001927 |
| FCHO2 | 0.211128 | 0.313265 | 0.270618 | 0.371814 | 0.30393 | 0.001942 |
| TMEM59 | 0.18359 | 0.524843 | 0.462129 | 0.587606 | 0.50393 | 0.001943 |
| SLC11A2 | 0.232293 | 0.26766 | 0.227854 | 0.325227 | 0.262009 | 0.002016 |
| TMEM160 | 0.220887 | 0.318156 | 0.27299 | 0.389833 | 0.323144 | 0.002019 |
| SF3B4 | 0.164242 | 0.497186 | 0.443687 | 0.553619 | 0.477729 | 0.002095 |
| UAP1 | 0.271203 | 0.249057 | 0.206375 | 0.29666 | 0.238428 | 0.002117 |
| SMURF1 | 0.30751 | 0.151813 | 0.12267 | 0.202608 | 0.151092 | 0.002135 |
| UROD | 0.190468 | 0.34584 | 0.303067 | 0.402432 | 0.330131 | 0.002136 |
| SEPTIN2 | 0.179771 | 0.502862 | 0.443948 | 0.563287 | 0.486463 | 0.002136 |
| ZNF787 | 0.244239 | 0.177783 | 0.150095 | 0.239818 | 0.183406 | 0.002167 |
| SLC20A1 | 0.130946 | 0.780683 | 0.712945 | 0.676531 | 0.604367 | 0.002191 |
| TBC1D15 | 0.183712 | 0.398304 | 0.35068 | 0.465866 | 0.393013 | 0.002284 |
| BTBD6 | 0.287237 | 0.169617 | 0.138996 | 0.222824 | 0.170306 | 0.002294 |
| MEF2D | 0.122505 | 0.880684 | 0.808989 | 0.774978 | 0.712664 | 0.002294 |
| MON1B | 0.143093 | 0.619623 | 0.561116 | 0.616906 | 0.524891 | 0.002331 |
| NCSTN | 0.110148 | 1.155977 | 1.071005 | 0.855699 | 0.788646 | 0.002334 |
| ADGRG3 | 1.59639 | 0.021155 | 0.006996 | 0.027981 | 0.01048 | 0.002338 |
| SPRY2 | 1.129127 | 0.024823 | 0.011349 | 0.033109 | 0.013974 | 0.002412 |
| DOCK5 | 0.089894 | 1.20611 | 1.133251 | 0.878113 | 0.815721 | 0.002431 |
| ZSWIM6 | 0.138462 | 0.711386 | 0.646285 | 0.684442 | 0.59476 | 0.002458 |
| CMBL | 65535 | 0.007258 | 0 | 0.010401 | 0 | 0.002462 |
| BRWD1 | 0.243606 | 0.171138 | 0.144549 | 0.226926 | 0.173799 | 0.002486 |
| RNF141 | 0.147375 | 0.72116 | 0.65113 | 0.665251 | 0.578166 | 0.002495 |
| TULP4 | 0.194262 | 0.276122 | 0.241336 | 0.334456 | 0.267249 | 0.00253 |
| LGALS8 | 0.121399 | 0.907051 | 0.833849 | 0.772927 | 0.704803 | 0.002611 |
| BCR | 0.225852 | 0.329031 | 0.281351 | 0.390565 | 0.324891 | 0.002642 |
| ATP13A2 | 0.244849 | 0.200352 | 0.169078 | 0.256959 | 0.2 | 0.002715 |
| ELMO2 | 0.180026 | 0.410875 | 0.362674 | 0.463961 | 0.39476 | 0.002854 |
| IL7 | 1.177873 | 0.019484 | 0.008612 | 0.02637 | 0.009607 | 0.002896 |
| ALOX5AP | 0.155686 | 0.470963 | 0.422786 | 0.424553 | 0.349345 | 0.002956 |
| TFG | 0.188127 | 0.338648 | 0.297246 | 0.409024 | 0.339738 | 0.002982 |
| EMD | 0.12555 | 0.713383 | 0.653926 | 0.680193 | 0.59738 | 0.002996 |
| SLC39A11 | 0.189771 | 0.377157 | 0.330671 | 0.431292 | 0.365066 | 0.003177 |
| GPX3 | 1.137475 | 0.023386 | 0.01063 | 0.031204 | 0.0131 | 0.003215 |
| HNRNPA3 | 0.129456 | 0.672169 | 0.614481 | 0.680633 | 0.608734 | 0.003283 |
| DNAJC8 | 0.145254 | 0.626852 | 0.566812 | 0.640639 | 0.576419 | 0.003361 |
| CKLF | 0.124204 | 0.897565 | 0.823525 | 0.771609 | 0.69607 | 0.003602 |
| ALG2 | 0.265891 | 0.238268 | 0.198164 | 0.297539 | 0.245415 | 0.003612 |
| PTPRJ | 0.118523 | 0.76939 | 0.708708 | 0.711984 | 0.659389 | 0.003656 |
| MGST2 | 0.160749 | 0.506574 | 0.453161 | 0.547905 | 0.470742 | 0.003722 |
| UQCR11 | 0.137851 | 0.676169 | 0.614551 | 0.686639 | 0.619214 | 0.003722 |
| ZRANB1 | 0.209742 | 0.314632 | 0.272059 | 0.382508 | 0.321397 | 0.003765 |
| CHUK | 0.24277 | 0.232456 | 0.196453 | 0.299883 | 0.241921 | 0.003859 |
| GPRC5A | 1.294166 | 0.034106 | 0.013907 | 0.035453 | 0.016594 | 0.00388 |
| BIK | 1.673943 | 0.01738 | 0.005447 | 0.023 | 0.00786 | 0.003977 |
| NISCH | 0.147089 | 0.593891 | 0.536326 | 0.605626 | 0.520524 | 0.003988 |
| ETNK1 | 0.129981 | 0.574036 | 0.524579 | 0.590243 | 0.51179 | 0.003988 |
| ANKHD1 | 0.09462 | 0.906624 | 0.849071 | 0.795927 | 0.730131 | 0.003988 |
| SPCS1 | 0.125304 | 0.788089 | 0.722528 | 0.724729 | 0.658515 | 0.004004 |
| CRLF2 | 1.217312 | 0.023916 | 0.010286 | 0.035453 | 0.016594 | 0.004023 |
| TP53BP2 | 0.137245 | 0.691246 | 0.628518 | 0.669206 | 0.6 | 0.004027 |
| HMGB2 | 0.122884 | 0.837095 | 0.768746 | 0.731761 | 0.666376 | 0.004044 |
| MAFK | 0.153481 | 0.521092 | 0.468503 | 0.545268 | 0.474236 | 0.004046 |
| RAB17 | 65535 | 0.006666 | 0 | 0.009522 | 0 | 0.004046 |
| HSP90AA1 | 0.126803 | 0.842486 | 0.771598 | 0.750952 | 0.680349 | 0.004079 |
| SEPTIN6 | 0.201748 | 0.359549 | 0.312627 | 0.431878 | 0.359825 | 0.004087 |
| TOR4A | 0.174054 | 0.287238 | 0.254593 | 0.358922 | 0.289083 | 0.004136 |
| TMEM185B | 0.198226 | 0.416247 | 0.36281 | 0.453267 | 0.38952 | 0.004185 |
| PLPP3 | 3.199297 | 0.011058 | 0.001204 | 0.012892 | 0.001747 | 0.004186 |
| SDF4 | 0.116533 | 0.897849 | 0.828178 | 0.771609 | 0.71441 | 0.004203 |
| HMGXB4 | 0.267148 | 0.206688 | 0.171749 | 0.261061 | 0.21048 | 0.004253 |
| SLC25A44 | 0.19457 | 0.33064 | 0.288925 | 0.391591 | 0.324017 | 0.004269 |
| SMARCD3 | 0.139293 | 0.67909 | 0.61659 | 0.651333 | 0.575546 | 0.004312 |
| KCTD7 | 0.204538 | 0.200573 | 0.17406 | 0.251538 | 0.199127 | 0.004346 |
| RHOBTB3 | 1.507734 | 0.01698 | 0.005971 | 0.024172 | 0.008734 | 0.004371 |
| KCNMA1 | 2.353681 | 0.011063 | 0.002164 | 0.014357 | 0.00262 | 0.004497 |
| CRELD2 | 0.156967 | 0.348202 | 0.312305 | 0.394667 | 0.327511 | 0.004568 |
| PSMD13 | 0.126953 | 0.733929 | 0.672105 | 0.703926 | 0.637555 | 0.004641 |
| H6PD | 0.177065 | 0.273861 | 0.24223 | 0.339584 | 0.273362 | 0.004761 |
| KDSR | 0.248268 | 0.264696 | 0.222849 | 0.314972 | 0.259389 | 0.004768 |
| FOXP4 | 0.206205 | 0.316865 | 0.274663 | 0.377088 | 0.31441 | 0.004807 |
| ATP2A2 | 0.177892 | 0.390027 | 0.344781 | 0.463668 | 0.391266 | 0.004807 |
| CYRIA | 0.112094 | 0.997065 | 0.922528 | 0.802959 | 0.740611 | 0.004834 |
| MAEA | 0.15294 | 0.464985 | 0.418215 | 0.515236 | 0.443668 | 0.004855 |
| TMSB4Y | 2.377864 | 0.011447 | 0.002202 | 0.015675 | 0.003493 | 0.004871 |
| GMPPB | 0.222533 | 0.272998 | 0.233975 | 0.319953 | 0.264629 | 0.004936 |
| ID1 | 0.207253 | 0.472656 | 0.409408 | 0.378992 | 0.322271 | 0.004971 |
| SEC61G | 0.208347 | 0.251574 | 0.217744 | 0.33138 | 0.266376 | 0.005131 |
| COX7A2 | 0.14712 | 0.43568 | 0.393442 | 0.506739 | 0.429694 | 0.005258 |
| MOSPD3 | 0.171317 | 0.268241 | 0.238206 | 0.318928 | 0.256769 | 0.005294 |
| ID2 | 0.105396 | 1.520576 | 1.413451 | 0.814679 | 0.761572 | 0.005434 |
| ASPSCR1 | 0.176627 | 0.327592 | 0.289843 | 0.379139 | 0.315284 | 0.005477 |
| MDM2 | 0.134571 | 0.736778 | 0.671161 | 0.684442 | 0.61048 | 0.005485 |
| CTTNBP2 | 1.409628 | 0.012686 | 0.004775 | 0.015529 | 0.003493 | 0.005489 |
| S1PR3 | 0.202466 | 0.420745 | 0.365654 | 0.403018 | 0.351965 | 0.005497 |
| C1QA | 1.96509 | 0.022804 | 0.005841 | 0.018312 | 0.00524 | 0.005561 |
| IDUA | 0.145837 | 0.238438 | 0.215513 | 0.300176 | 0.241921 | 0.005609 |
| OTOF | 1.899126 | 0.018037 | 0.004836 | 0.016847 | 0.004367 | 0.005666 |
| INTS10 | 0.187775 | 0.318118 | 0.279294 | 0.38119 | 0.313537 | 0.005758 |
| LBR | 0.127373 | 0.69156 | 0.633121 | 0.675505 | 0.59738 | 0.005811 |
| PSMB2 | 0.171375 | 0.459061 | 0.407645 | 0.526223 | 0.460262 | 0.005878 |
| SUGT1 | 0.177995 | 0.394704 | 0.348891 | 0.459713 | 0.38952 | 0.005896 |
| YWHAG | 0.140514 | 0.614358 | 0.557343 | 0.639906 | 0.574672 | 0.005926 |
| DHDDS | 0.210123 | 0.220267 | 0.190413 | 0.270876 | 0.216594 | 0.006013 |
| YIF1B | 0.143922 | 0.625224 | 0.565863 | 0.620568 | 0.549345 | 0.006028 |
| PSMB6 | 0.178495 | 0.42698 | 0.37729 | 0.516701 | 0.448035 | 0.006029 |
| BEX4 | 0.242739 | 0.211356 | 0.178626 | 0.257691 | 0.206114 | 0.006037 |
| TLK2 | 0.233978 | 0.261466 | 0.222321 | 0.332113 | 0.273362 | 0.006084 |
| INPPL1 | 0.098851 | 0.967537 | 0.903463 | 0.812628 | 0.754585 | 0.006178 |
| LACC1 | 0.210014 | 0.23725 | 0.205109 | 0.285819 | 0.231441 | 0.006201 |
| RAB3IL1 | 3.402965 | 0.007437 | 0.000703 | 0.010548 | 0.000873 | 0.006214 |
| NDUFAF8 | 0.165936 | 0.447384 | 0.398776 | 0.484325 | 0.414847 | 0.006346 |
| WDR26 | 0.108124 | 0.869798 | 0.806993 | 0.773367 | 0.70393 | 0.006383 |
| ABHD16A | 0.140131 | 0.391425 | 0.355193 | 0.460592 | 0.385153 | 0.006465 |
| SDF2 | 0.160059 | 0.33245 | 0.297539 | 0.396425 | 0.329258 | 0.006471 |
| SRSF1 | 0.177129 | 0.364815 | 0.322664 | 0.437299 | 0.370306 | 0.006522 |
| ANAPC15 | 0.137964 | 0.573911 | 0.521571 | 0.572077 | 0.491703 | 0.006557 |
| CXXC1 | 0.185075 | 0.43916 | 0.386287 | 0.487548 | 0.423581 | 0.006579 |
| BCL7B | 0.11637 | 0.78891 | 0.727774 | 0.724729 | 0.661135 | 0.006893 |
| SLC22A4 | 0.1808 | 0.383853 | 0.338641 | 0.420744 | 0.358079 | 0.006931 |
| PSMD1 | 0.171908 | 0.410601 | 0.364477 | 0.480809 | 0.41048 | 0.00698 |
| CCDC122 | 1.989177 | 0.011235 | 0.00283 | 0.016408 | 0.004367 | 0.007047 |
| RTL8C | 0.170031 | 0.418287 | 0.371783 | 0.487108 | 0.417467 | 0.007226 |
| TOX4 | 0.153993 | 0.492237 | 0.442403 | 0.541899 | 0.466376 | 0.007253 |
| HSPA13 | 0.211502 | 0.230807 | 0.199334 | 0.2908 | 0.235808 | 0.007265 |
| LAIR1 | 0.115152 | 0.77601 | 0.716479 | 0.677996 | 0.620961 | 0.007291 |
| GADD45GIP1 | 0.183299 | 0.393947 | 0.346944 | 0.441108 | 0.377293 | 0.007312 |
| PNMA1 | 0.218167 | 0.285107 | 0.245095 | 0.310431 | 0.256769 | 0.007497 |
| ULBP1 | 1.719532 | 0.011928 | 0.003622 | 0.017726 | 0.00524 | 0.007543 |
| ADAP1 | 0.109909 | 0.949762 | 0.880093 | 0.800615 | 0.731004 | 0.00763 |
| RALBP1 | 0.160235 | 0.497793 | 0.445465 | 0.542778 | 0.478603 | 0.007718 |
| STK3 | 0.230973 | 0.223713 | 0.190617 | 0.286551 | 0.231441 | 0.00773 |
| UBE2K | 0.210107 | 0.18213 | 0.157446 | 0.249341 | 0.198253 | 0.007827 |
| CTR9 | 0.195584 | 0.286626 | 0.250287 | 0.355406 | 0.29345 | 0.007985 |
| AHR | 0.093935 | 1.721255 | 1.612754 | 0.930853 | 0.874236 | 0.007988 |
| AP2A2 | 0.147882 | 0.501296 | 0.452457 | 0.537211 | 0.468996 | 0.008097 |
| C12orf57 | 0.078418 | 1.182889 | 1.120309 | 0.741283 | 0.660262 | 0.008226 |
| KCTD17 | 0.164909 | 0.299803 | 0.26742 | 0.35189 | 0.29345 | 0.00834 |
| ZFAND2A | 0.141414 | 0.539768 | 0.48937 | 0.56695 | 0.49345 | 0.00834 |
| HTRA4 | 1.236193 | 0.013736 | 0.005831 | 0.018898 | 0.006114 | 0.008425 |
| LPAR2 | 0.142433 | 0.426379 | 0.386295 | 0.469675 | 0.399127 | 0.00846 |
| CYP51A1 | 0.245441 | 0.212694 | 0.179419 | 0.266921 | 0.215721 | 0.008473 |
| HAX1 | 0.225348 | 0.19016 | 0.162661 | 0.256373 | 0.20524 | 0.008486 |
| RRM2 | 1.898396 | 0.010098 | 0.002709 | 0.01465 | 0.003493 | 0.008516 |
| SLC36A4 | 0.183395 | 0.298782 | 0.263116 | 0.358775 | 0.29869 | 0.008585 |
| PIP4P1 | 0.155667 | 0.534714 | 0.480022 | 0.553472 | 0.48821 | 0.00863 |
| IGF1R | 0.186265 | 0.251286 | 0.220849 | 0.309259 | 0.252402 | 0.008729 |
| MBD1 | 0.184223 | 0.21432 | 0.188628 | 0.279226 | 0.224454 | 0.008768 |
| SLC26A8 | 1.561783 | 0.015541 | 0.005264 | 0.02007 | 0.006987 | 0.008796 |
| EIF4E | 0.230095 | 0.181503 | 0.154746 | 0.240551 | 0.18952 | 0.008825 |
| NDUFA7 | 0.18149 | 0.381606 | 0.336497 | 0.43935 | 0.378166 | 0.00888 |
| DLG4 | 0.158394 | 0.433592 | 0.388508 | 0.472898 | 0.406987 | 0.009128 |
| MFSD11 | 0.217404 | 0.213294 | 0.183457 | 0.272488 | 0.220087 | 0.009133 |
| SRSF11 | 0.188598 | 0.236039 | 0.207115 | 0.309405 | 0.252402 | 0.009145 |
| AREL1 | 0.188385 | 0.246177 | 0.216042 | 0.313068 | 0.254148 | 0.009145 |
| JADE3 | 1.277247 | 0.0194 | 0.008004 | 0.027542 | 0.012227 | 0.009149 |
| SLC44A1 | 0.147829 | 0.386682 | 0.349022 | 0.440082 | 0.377293 | 0.009388 |
| DNMT3A | 0.179815 | 0.284468 | 0.251133 | 0.329476 | 0.274236 | 0.009447 |
| GK3 | 1.21717 | 0.017336 | 0.007457 | 0.026223 | 0.011354 | 0.009651 |
| CD58 | 0.12431 | 0.663391 | 0.608623 | 0.655142 | 0.579913 | 0.009775 |
| LYAR | 0.193724 | 0.286082 | 0.250135 | 0.348081 | 0.291703 | 0.009915 |
| HDAC3 | 0.198552 | 0.259322 | 0.22598 | 0.328157 | 0.269869 | 0.00997 |
| **Non-classical monocytes** | | |  |  |  |  |
| SIGLEC14 | 2.328336 | 0.771939 | 0.153704 | 0.666093 | 0.133641 | 3.85E-90 |
| CLEC12A | 0.873482 | 1.513019 | 0.825848 | 0.922547 | 0.723502 | 4.35E-78 |
| DDX3Y | 2.712402 | 0.678837 | 0.103574 | 0.54475 | 0.095238 | 8.29E-74 |
| MT-ND2 | 1.157646 | 1.534971 | 0.688041 | 0.806368 | 0.376344 | 2.58E-54 |
| UTY | 2.506025 | 0.370696 | 0.065257 | 0.384682 | 0.069124 | 1.88E-42 |
| KDM5D | 3.094514 | 0.281071 | 0.032906 | 0.314114 | 0.046083 | 2.3E-37 |
| EIF1AY | 2.685865 | 0.267768 | 0.041613 | 0.297762 | 0.050691 | 3.14E-32 |
| ETS2 | 0.61508 | 1.198824 | 0.782705 | 0.825301 | 0.705069 | 2.51E-31 |
| ZBTB16 | 2.068364 | 0.329156 | 0.078481 | 0.345955 | 0.101382 | 1.1E-28 |
| PLCXD1 | 1.306241 | 0.608106 | 0.245902 | 0.502582 | 0.256528 | 6.93E-27 |
| PHC2 | 0.526537 | 1.128357 | 0.783327 | 0.805508 | 0.701997 | 8.79E-24 |
| JUP | 0.79014 | 0.881757 | 0.50991 | 0.660929 | 0.477727 | 1.81E-19 |
| ADK | 0.755823 | 0.65961 | 0.390627 | 0.63253 | 0.442396 | 2.17E-19 |
| KCNN4 | 1.436935 | 0.268507 | 0.099173 | 0.309811 | 0.124424 | 9.18E-17 |
| SOCS3 | 0.58785 | 1.458787 | 0.97058 | 0.713425 | 0.614439 | 1.06E-16 |
| RNF144B | 0.441631 | 1.162327 | 0.855823 | 0.791738 | 0.721966 | 8.88E-16 |
| SLA | 0.395265 | 1.035425 | 0.787285 | 0.789157 | 0.725038 | 7.46E-15 |
| OSBPL8 | 0.348769 | 1.017674 | 0.799133 | 0.819277 | 0.740399 | 1.04E-14 |
| TBC1D8 | 0.373648 | 1.099779 | 0.848841 | 0.820138 | 0.734255 | 1.97E-13 |
| KNDC1 | 0.789594 | 0.476811 | 0.275839 | 0.458692 | 0.261137 | 2.52E-13 |
| SULT1A1 | 1.336027 | 0.251833 | 0.099754 | 0.290878 | 0.130568 | 3.43E-13 |
| IFI27 | 4.43118 | 0.187398 | 0.008687 | 0.107573 | 0.00768 | 3.67E-13 |
| PAG1 | 0.323151 | 1.124808 | 0.899084 | 0.847676 | 0.78341 | 1.59E-12 |
| ATP5PO | 1.420833 | 0.137334 | 0.051294 | 0.20568 | 0.064516 | 3.37E-12 |
| PALM | 2.809892 | 0.105454 | 0.015038 | 0.113597 | 0.013825 | 5.01E-12 |
| ABCA7 | 0.770819 | 0.413615 | 0.242413 | 0.464716 | 0.290323 | 5.19E-12 |
| CNN2 | 0.315991 | 1.181376 | 0.948998 | 0.850258 | 0.784946 | 1.09E-11 |
| LILRA2 | 0.328996 | 1.159791 | 0.923298 | 0.835628 | 0.749616 | 1.94E-11 |
| GPR27 | 2.842539 | 0.087571 | 0.012209 | 0.111015 | 0.015361 | 2.72E-11 |
| LYPD2 | 1.560235 | 0.379778 | 0.128781 | 0.209983 | 0.078341 | 2.83E-11 |
| BLOC1S1 | 0.389814 | 0.903286 | 0.689414 | 0.76506 | 0.65745 | 7.64E-11 |
| KCNC3 | 0.651655 | 0.600386 | 0.382176 | 0.561102 | 0.419355 | 1.03E-10 |
| SLC2A3 | 1.163931 | 0.432994 | 0.193243 | 0.349398 | 0.211982 | 1.3E-10 |
| CMKLR1 | 0.942868 | 0.332505 | 0.172968 | 0.350258 | 0.195084 | 1.55E-10 |
| PDGFC | 0.926794 | 0.253153 | 0.133165 | 0.301205 | 0.15361 | 5.81E-10 |
| EGR3 | 0.903986 | 0.353317 | 0.188815 | 0.351119 | 0.205837 | 6.99E-10 |
| FCER2 | 2.652195 | 0.089161 | 0.014183 | 0.10327 | 0.016897 | 9.13E-10 |
| SIRPB1 | 0.396391 | 0.857481 | 0.651476 | 0.718589 | 0.645161 | 9.18E-10 |
| SNX18 | 0.432961 | 0.799302 | 0.592075 | 0.685026 | 0.592934 | 1.14E-09 |
| ZFY | 2.208802 | 0.079477 | 0.017192 | 0.110155 | 0.021505 | 1.97E-09 |
| NLRP6 | 1.236277 | 0.191861 | 0.081439 | 0.233219 | 0.107527 | 4.35E-09 |
| ENGASE | 0.832119 | 0.328849 | 0.184716 | 0.376936 | 0.230415 | 6.68E-09 |
| LILRA6 | 0.781599 | 0.353313 | 0.20553 | 0.416523 | 0.27957 | 8.24E-09 |
| MAN2A2 | 0.439899 | 0.704468 | 0.519324 | 0.669535 | 0.560676 | 8.61E-09 |
| ADM | 1.051049 | 0.280496 | 0.135372 | 0.274527 | 0.144393 | 9.92E-09 |
| P2RY2 | 0.946319 | 0.347095 | 0.180127 | 0.348537 | 0.225806 | 1.7E-08 |
| CH25H | 1.345406 | 0.303835 | 0.119572 | 0.211704 | 0.099846 | 3.09E-08 |
| HES4 | 0.473361 | 0.673152 | 0.484861 | 0.590361 | 0.462366 | 3.27E-08 |
| APBB3 | 0.721422 | 0.472518 | 0.286582 | 0.422547 | 0.288786 | 3.87E-08 |
| PELI1 | 0.376372 | 0.987857 | 0.761018 | 0.72031 | 0.637481 | 4.37E-08 |
| KCNMA1 | 1.407426 | 0.120826 | 0.045549 | 0.144578 | 0.049155 | 7.16E-08 |
| YBX3 | 0.322852 | 1.001459 | 0.800654 | 0.767642 | 0.668203 | 9.31E-08 |
| FKBP5 | 0.629856 | 0.464888 | 0.30043 | 0.466437 | 0.348694 | 1.01E-07 |
| SH3TC1 | 0.361739 | 0.81234 | 0.632184 | 0.703098 | 0.628264 | 1.74E-07 |
| CDC42EP2 | 0.614876 | 0.448369 | 0.292779 | 0.451807 | 0.3149 | 1.75E-07 |
| RAP1GAP2 | 0.259279 | 1.032456 | 0.862623 | 0.832186 | 0.764977 | 2.23E-07 |
| EGR1 | 1.032199 | 0.358905 | 0.175492 | 0.292599 | 0.178187 | 3.48E-07 |
| HS3ST3B1 | 0.844021 | 0.324201 | 0.180609 | 0.339071 | 0.22427 | 3.55E-07 |
| P2RY1 | 1.447243 | 0.0884 | 0.032418 | 0.119621 | 0.038402 | 6.98E-07 |
| PROK2 | 2.371632 | 0.070472 | 0.013617 | 0.08778 | 0.019969 | 7.26E-07 |
| TNNI2 | 0.86056 | 0.248107 | 0.136642 | 0.288296 | 0.175115 | 9.61E-07 |
| PLEKHG3 | 0.786762 | 0.228246 | 0.132302 | 0.281411 | 0.165899 | 9.96E-07 |
| CLEC12B | 0.987457 | 0.163396 | 0.082411 | 0.201377 | 0.099846 | 1.89E-06 |
| PTP4A3 | 0.236447 | 1.2252 | 1.03999 | 0.851119 | 0.784946 | 2.06E-06 |
| ACSL1 | 0.328679 | 0.923875 | 0.73565 | 0.710843 | 0.651306 | 3.47E-06 |
| ARID5B | 1.621488 | 0.106214 | 0.03452 | 0.13167 | 0.052227 | 3.65E-06 |
| RRP7A | 0.617039 | 0.291927 | 0.190339 | 0.357143 | 0.238095 | 1.03E-05 |
| TMTC1 | 1.417259 | 0.082377 | 0.030844 | 0.097246 | 0.030722 | 1.14E-05 |
| DDIT4 | 0.460069 | 0.827235 | 0.60136 | 0.564544 | 0.491551 | 1.43E-05 |
| FAM20A | 1.180212 | 0.134755 | 0.059465 | 0.154905 | 0.072197 | 1.49E-05 |
| C1QA | 0.948908 | 0.422861 | 0.219053 | 0.240103 | 0.144393 | 1.55E-05 |
| HSH2D | 0.307267 | 0.7496 | 0.605806 | 0.703959 | 0.643625 | 1.73E-05 |
| THBS1 | 0.987307 | 0.307283 | 0.154999 | 0.22031 | 0.12596 | 1.81E-05 |
| GIMAP6 | 0.458155 | 0.47452 | 0.345411 | 0.528399 | 0.420891 | 1.86E-05 |
| MERTK | 0.63656 | 0.375941 | 0.241822 | 0.364888 | 0.258065 | 1.87E-05 |
| GIMAP1 | 0.359773 | 0.601085 | 0.468418 | 0.6179 | 0.52381 | 4.11E-05 |
| NUAK2 | 0.529142 | 0.43845 | 0.303831 | 0.44062 | 0.341014 | 4.69E-05 |
| ZNF141 | 0.817247 | 0.227845 | 0.129307 | 0.262478 | 0.173579 | 4.98E-05 |
| FCAR | 0.330571 | 0.957465 | 0.761397 | 0.643718 | 0.526882 | 5.24E-05 |
| PIM2 | 0.706032 | 0.195541 | 0.119867 | 0.261618 | 0.162826 | 5.32E-05 |
| ZNF593 | 0.643055 | 0.267821 | 0.171501 | 0.331325 | 0.228879 | 5.94E-05 |
| XRRA1 | 1.544826 | 0.075414 | 0.025847 | 0.100688 | 0.038402 | 7.84E-05 |
| SIRPB2 | 0.368383 | 0.587303 | 0.454955 | 0.586059 | 0.482335 | 8.35E-05 |
| S100A12 | 1.167641 | 0.115972 | 0.051625 | 0.135972 | 0.064516 | 9.26E-05 |
| MDM2 | 0.320477 | 0.721754 | 0.577984 | 0.666954 | 0.589862 | 9.32E-05 |
| WWC3 | 0.549905 | 0.269881 | 0.184346 | 0.336489 | 0.22427 | 0.0001 |
| PSD4 | 0.272824 | 0.645963 | 0.534662 | 0.694492 | 0.628264 | 0.000122 |
| ABCA1 | 0.574715 | 0.432967 | 0.290702 | 0.389845 | 0.294931 | 0.000122 |
| ELL2 | 0.500473 | 0.457578 | 0.32345 | 0.462134 | 0.365591 | 0.000123 |
| PRR5 | 1.150108 | 0.113593 | 0.051184 | 0.154045 | 0.079877 | 0.000131 |
| MAD1L1 | 0.384077 | 0.567508 | 0.434863 | 0.575731 | 0.517665 | 0.000131 |
| PHACTR1 | 0.524489 | 0.463627 | 0.322316 | 0.443201 | 0.356375 | 0.000144 |
| SPATA13 | 0.286213 | 0.660813 | 0.541901 | 0.649742 | 0.589862 | 0.000154 |
| NANS | 0.363869 | 0.534802 | 0.415583 | 0.551635 | 0.466974 | 0.000154 |
| IL10 | 2.743036 | 0.045918 | 0.006859 | 0.050775 | 0.009217 | 0.000169 |
| GPBAR1 | 0.309545 | 0.66929 | 0.540048 | 0.638554 | 0.562212 | 0.000189 |
| SUSD3 | 2.043645 | 0.039736 | 0.009638 | 0.063683 | 0.016897 | 0.000199 |
| MEPCE | 0.26118 | 0.778695 | 0.649747 | 0.719449 | 0.645161 | 0.000213 |
| BASP1 | 0.702182 | 0.273249 | 0.16795 | 0.282272 | 0.196621 | 0.000272 |
| CEMIP2 | 0.338714 | 0.534145 | 0.422373 | 0.557659 | 0.463902 | 0.000272 |
| GRAMD1A | 0.332178 | 0.648313 | 0.514978 | 0.615318 | 0.551459 | 0.00029 |
| LYNX1 | 0.563912 | 0.251225 | 0.169945 | 0.306368 | 0.208909 | 0.000319 |
| TAMALIN | 0.302914 | 0.845509 | 0.685381 | 0.655766 | 0.602151 | 0.000319 |
| BIN1 | 1.153534 | 0.078784 | 0.035415 | 0.11704 | 0.053763 | 0.000336 |
| ELMOD3 | 1.024441 | 0.105854 | 0.052038 | 0.148881 | 0.079877 | 0.000504 |
| SLC49A4 | 0.320848 | 0.579046 | 0.463584 | 0.570568 | 0.499232 | 0.00051 |
| TLE4 | 0.306951 | 0.609466 | 0.492661 | 0.604991 | 0.552995 | 0.000535 |
| STX7 | 0.213159 | 0.968569 | 0.835533 | 0.805508 | 0.743472 | 0.000558 |
| STX2 | 0.650258 | 0.222275 | 0.141626 | 0.271945 | 0.184332 | 0.000597 |
| PHYKPL | 0.298909 | 0.601133 | 0.488642 | 0.611015 | 0.556068 | 0.000752 |
| TMEM259 | 0.229117 | 0.747525 | 0.637756 | 0.702238 | 0.643625 | 0.000773 |
| NOCT | 0.479193 | 0.428168 | 0.307159 | 0.434596 | 0.364055 | 0.000843 |
| EVA1B | 1.39512 | 0.057584 | 0.021894 | 0.077453 | 0.029186 | 0.000843 |
| SELENOO | 0.463292 | 0.309984 | 0.22484 | 0.36747 | 0.284178 | 0.000894 |
| TTC39B | 1.727857 | 0.049667 | 0.014994 | 0.070568 | 0.024578 | 0.000902 |
| GPRIN3 | 1.69211 | 0.042038 | 0.01301 | 0.066265 | 0.021505 | 0.000912 |
| H2BC21 | 0.382703 | 0.569866 | 0.437087 | 0.510327 | 0.422427 | 0.000963 |
| MAP3K7CL | 1.629545 | 0.088741 | 0.02868 | 0.094664 | 0.043011 | 0.001041 |
| LPAR2 | 0.297098 | 0.65955 | 0.5368 | 0.626506 | 0.533026 | 0.001045 |
| USP9Y | 4.862892 | 0.021032 | 0.000723 | 0.02926 | 0.001536 | 0.001051 |
| FBXW8 | 0.803542 | 0.138641 | 0.079433 | 0.177281 | 0.105991 | 0.001116 |
| MAN1A1 | 0.333685 | 0.482728 | 0.383048 | 0.538726 | 0.460829 | 0.001218 |
| B3GALNT2 | 0.910847 | 0.12771 | 0.067925 | 0.163511 | 0.096774 | 0.001263 |
| KLHDC8B | 2.710858 | 0.034489 | 0.005268 | 0.04389 | 0.009217 | 0.001265 |
| FYN | 0.269915 | 0.600844 | 0.498321 | 0.636833 | 0.542243 | 0.001272 |
| SFTPD | 0.700589 | 0.152456 | 0.093809 | 0.179002 | 0.107527 | 0.001692 |
| CXCL8 | 0.327901 | 0.859943 | 0.685112 | 0.45525 | 0.347158 | 0.001713 |
| CD244 | 0.249355 | 0.724627 | 0.609608 | 0.646299 | 0.589862 | 0.001746 |
| MGLL | 0.362657 | 0.509292 | 0.396092 | 0.490534 | 0.402458 | 0.001828 |
| EOLA1 | 0.474338 | 0.314889 | 0.226656 | 0.364028 | 0.281106 | 0.001862 |
| NEURL1 | 0.216252 | 1.097561 | 0.944779 | 0.784854 | 0.734255 | 0.001875 |
| FFAR2 | 0.548335 | 0.304439 | 0.208178 | 0.320998 | 0.241167 | 0.001877 |
| PDK4 | 0.35368 | 0.553335 | 0.433032 | 0.500861 | 0.417819 | 0.001923 |
| IGFBP6 | 1.236503 | 0.098688 | 0.041883 | 0.099828 | 0.047619 | 0.002017 |
| ITPKB | 0.305039 | 0.525983 | 0.425741 | 0.58864 | 0.513057 | 0.002206 |
| MTHFR | 0.332446 | 0.465542 | 0.369728 | 0.494836 | 0.417819 | 0.002314 |
| HRK | 4.006796 | 0.024302 | 0.001512 | 0.026678 | 0.001536 | 0.002326 |
| LAP3 | 0.235582 | 0.908259 | 0.771423 | 0.730637 | 0.680492 | 0.002326 |
| ATP8B4 | 1.040968 | 0.086478 | 0.042029 | 0.111876 | 0.058372 | 0.002516 |
| NFKBIB | 0.543019 | 0.263612 | 0.180926 | 0.322719 | 0.248848 | 0.002523 |
| ISG20 | 1.000644 | 0.119265 | 0.059606 | 0.148021 | 0.086022 | 0.002674 |
| C1QB | 1.165407 | 0.13609 | 0.060674 | 0.08864 | 0.039939 | 0.002675 |
| S1PR4 | 0.222729 | 0.724297 | 0.620682 | 0.708262 | 0.654378 | 0.003034 |
| RUNX1 | 0.257571 | 0.707973 | 0.592216 | 0.640275 | 0.583717 | 0.003097 |
| GFI1B | 2.045528 | 0.039119 | 0.009476 | 0.045611 | 0.012289 | 0.003405 |
| SCARB1 | 0.940073 | 0.109876 | 0.057268 | 0.140275 | 0.081413 | 0.003415 |
| NQO2 | 0.466487 | 0.31034 | 0.224601 | 0.354561 | 0.27957 | 0.003847 |
| SNED1 | 0.976636 | 0.137183 | 0.069711 | 0.153184 | 0.093702 | 0.00408 |
| C19orf25 | 0.502375 | 0.255326 | 0.180246 | 0.304647 | 0.225806 | 0.004156 |
| CNIH2 | 2.788612 | 0.021088 | 0.003052 | 0.030981 | 0.004608 | 0.004414 |
| HSPA1B | 0.461095 | 0.303436 | 0.220426 | 0.330465 | 0.245776 | 0.004487 |
| MCTP2 | 0.841941 | 0.121591 | 0.067835 | 0.142857 | 0.084485 | 0.004662 |
| NTPCR | 0.871796 | 0.111461 | 0.06091 | 0.152324 | 0.093702 | 0.005386 |
| CLCF1 | 0.569128 | 0.258355 | 0.174137 | 0.254733 | 0.182796 | 0.006043 |
| ELFN1 | 4.670116 | 0.018876 | 0.000741 | 0.023236 | 0.001536 | 0.006398 |
| PRMT9 | 0.593101 | 0.190532 | 0.126307 | 0.228055 | 0.16129 | 0.006592 |
| CLCN6 | 0.497045 | 0.233862 | 0.165705 | 0.297762 | 0.225806 | 0.006714 |
| ACSL3 | 0.391085 | 0.359762 | 0.274339 | 0.422547 | 0.347158 | 0.007046 |
| FLOT1 | 0.347987 | 0.380422 | 0.29889 | 0.436317 | 0.359447 | 0.00744 |
| ST3GAL2 | 0.389664 | 0.384492 | 0.293486 | 0.430293 | 0.362519 | 0.007501 |
| MAFF | 0.488931 | 0.208042 | 0.148241 | 0.225473 | 0.155146 | 0.008112 |
| TMEM51 | 1.121998 | 0.055819 | 0.025646 | 0.074871 | 0.033794 | 0.008226 |
| SNAP23 | 0.244262 | 0.634046 | 0.535292 | 0.643718 | 0.583717 | 0.008229 |
| CLN6 | 0.318428 | 0.415042 | 0.33284 | 0.468158 | 0.394777 | 0.008667 |
| CDKN1A | 0.175376 | 1.530289 | 1.355127 | 0.787435 | 0.725038 | 0.009357 |
| SPCS1 | 0.200568 | 0.619459 | 0.539058 | 0.67642 | 0.625192 | 0.009638 |
| TNFAIP8L2 | 0.429783 | 0.296726 | 0.220282 | 0.359725 | 0.281106 | 0.009728 |
| **FCER1A+ monocytes** | | |  |  |  |  |
| MT-ND2 | 1.739568 | 1.83564 | 0.5497 | 0.960396 | 0.351351 | 1.05E-33 |
| SIGLEC14 | 1.987662 | 1.059052 | 0.267037 | 0.839109 | 0.209459 | 1.85E-27 |
| DDX3Y | 2.118363 | 1.113995 | 0.256562 | 0.717822 | 0.168919 | 1.6E-21 |
| SLC2A3 | 0.995744 | 1.57155 | 0.788097 | 0.868812 | 0.614865 | 9.44E-18 |
| BCL3 | 0.649092 | 1.793715 | 1.143819 | 0.955446 | 0.804054 | 5.54E-17 |
| SOCS3 | 0.440916 | 2.273271 | 1.674644 | 0.950495 | 0.871622 | 4.65E-11 |
| CEBPB | 0.619785 | 1.727748 | 1.124362 | 0.881188 | 0.72973 | 2.02E-10 |
| DDIT4 | 0.504435 | 2.072818 | 1.461204 | 0.962871 | 0.858108 | 3.66E-10 |
| IRS2 | 0.889878 | 1.184541 | 0.639249 | 0.787129 | 0.554054 | 1.26E-09 |
| SMAP2 | 0.308634 | 1.977131 | 1.596347 | 0.982673 | 0.925676 | 1.51E-09 |
| UTY | 1.970156 | 0.371513 | 0.09482 | 0.445545 | 0.114865 | 2.66E-09 |
| LIPA | 0.488437 | 1.549187 | 1.104256 | 0.933168 | 0.790541 | 4.7E-09 |
| FKBP5 | 0.585935 | 1.172073 | 0.780855 | 0.881188 | 0.709459 | 5.47E-09 |
| CLEC12A | 0.800717 | 0.998493 | 0.573198 | 0.806931 | 0.560811 | 8.32E-09 |
| SBNO2 | 0.560405 | 1.273684 | 0.8637 | 0.925743 | 0.722973 | 1.21E-08 |
| GCA | 0.493118 | 1.392782 | 0.989555 | 0.910891 | 0.797297 | 2.9E-08 |
| EIF1AY | 2.32059 | 0.271862 | 0.054423 | 0.35396 | 0.074324 | 6.39E-08 |
| SERPINB9 | 0.825473 | 0.989702 | 0.558486 | 0.742574 | 0.466216 | 2.68E-07 |
| LILRA6 | 2.765079 | 0.228653 | 0.033636 | 0.289604 | 0.040541 | 2.83E-07 |
| JAK3 | 0.660855 | 0.919328 | 0.581478 | 0.821782 | 0.614865 | 5.21E-07 |
| MXD1 | 0.537098 | 1.430133 | 0.985584 | 0.846535 | 0.736486 | 5.29E-07 |
| LAP3 | 0.396745 | 1.466004 | 1.113533 | 0.950495 | 0.837838 | 1.33E-06 |
| ATP5PO | 1.46758 | 0.326696 | 0.118129 | 0.435644 | 0.148649 | 1.67E-06 |
| PLSCR1 | 0.483224 | 1.273273 | 0.91087 | 0.90099 | 0.756757 | 1.67E-06 |
| PTPN1 | 0.403977 | 1.260247 | 0.95246 | 0.938119 | 0.831081 | 1.67E-06 |
| RNF144B | 0.825989 | 0.860414 | 0.485355 | 0.707921 | 0.452703 | 1.93E-06 |
| CSNK1D | 0.474997 | 1.146673 | 0.824995 | 0.89604 | 0.722973 | 3.7E-06 |
| PFKFB3 | 0.500344 | 1.514047 | 1.070338 | 0.861386 | 0.75 | 4.24E-06 |
| KDM6B | 0.409935 | 1.498589 | 1.127925 | 0.945545 | 0.831081 | 5.17E-06 |
| DUSP3 | 0.540009 | 1.170592 | 0.805094 | 0.866337 | 0.668919 | 5.67E-06 |
| STAT3 | 0.415926 | 1.199962 | 0.899418 | 0.881188 | 0.790541 | 5.67E-06 |
| SLC7A5 | 0.500795 | 1.33379 | 0.942612 | 0.826733 | 0.702703 | 1.1E-05 |
| EOLA1 | 0.705445 | 0.97173 | 0.595917 | 0.75495 | 0.533784 | 1.16E-05 |
| HS3ST3B1 | 1.471421 | 0.334391 | 0.12059 | 0.388614 | 0.141892 | 1.77E-05 |
| KDM5D | 1.695592 | 0.243918 | 0.075304 | 0.334158 | 0.101351 | 2.08E-05 |
| RNF149 | 0.416834 | 1.398705 | 1.047723 | 0.933168 | 0.790541 | 2.22E-05 |
| KCNC3 | 0.690897 | 0.856405 | 0.530516 | 0.725248 | 0.47973 | 2.61E-05 |
| AREG | 0.774505 | 1.400777 | 0.818879 | 0.695545 | 0.466216 | 3.19E-05 |
| SGK1 | 0.493552 | 1.759322 | 1.249601 | 0.866337 | 0.736486 | 3.66E-05 |
| MAT2A | 0.320755 | 1.666782 | 1.33451 | 0.957921 | 0.905405 | 3.66E-05 |
| ABCA7 | 0.892422 | 0.522992 | 0.28174 | 0.611386 | 0.331081 | 3.82E-05 |
| PELI1 | 1.040043 | 0.528105 | 0.256824 | 0.522277 | 0.27027 | 5.53E-05 |
| SIRPB1 | 0.981441 | 0.488645 | 0.247486 | 0.492574 | 0.25 | 8.05E-05 |
| TMEM259 | 0.420223 | 0.945655 | 0.706697 | 0.863861 | 0.736486 | 8.05E-05 |
| ETS2 | 0.39113 | 1.373654 | 1.047455 | 0.873762 | 0.797297 | 8.18E-05 |
| MIF4GD | 0.613385 | 0.761425 | 0.497714 | 0.740099 | 0.5 | 0.000134 |
| FCER1A | 0.361298 | 2.607289 | 2.02968 | 0.90099 | 0.777027 | 0.000139 |
| CD300E | 0.985811 | 0.719458 | 0.363285 | 0.534653 | 0.324324 | 0.000158 |
| DSE | 0.437597 | 1.253862 | 0.925806 | 0.873762 | 0.722973 | 0.000162 |
| ACSL1 | 0.810623 | 0.704711 | 0.401781 | 0.60396 | 0.412162 | 0.00021 |
| TNFRSF1B | 0.251813 | 1.894625 | 1.591182 | 0.980198 | 0.905405 | 0.000214 |
| ABCA1 | 2.855145 | 0.175718 | 0.024285 | 0.180693 | 0.02027 | 0.000216 |
| APBB3 | 0.366738 | 1.724279 | 1.337237 | 0.886139 | 0.797297 | 0.000216 |
| HNRNPK | 0.313593 | 1.07682 | 0.866448 | 0.905941 | 0.817568 | 0.00023 |
| INAFM2 | 0.988283 | 0.430422 | 0.216966 | 0.470297 | 0.236486 | 0.000267 |
| GUK1 | 0.509895 | 0.769877 | 0.540664 | 0.789604 | 0.614865 | 0.000296 |
| FLVCR2 | 1.689878 | 0.243753 | 0.075552 | 0.289604 | 0.101351 | 0.00031 |
| SCO2 | 0.294805 | 1.807596 | 1.473521 | 0.965347 | 0.871622 | 0.00031 |
| FLOT1 | 0.467722 | 0.978414 | 0.707497 | 0.806931 | 0.662162 | 0.000342 |
| JOSD1 | 0.368113 | 1.003035 | 0.777146 | 0.856436 | 0.763514 | 0.000349 |
| PPIF | 0.313425 | 1.845122 | 1.484823 | 0.928218 | 0.864865 | 0.000382 |
| PITPNA | 0.313841 | 1.26053 | 1.014092 | 0.903465 | 0.810811 | 0.000382 |
| IQSEC1 | 0.283036 | 1.433356 | 1.178017 | 0.960396 | 0.885135 | 0.000382 |
| PIM1 | 0.582724 | 0.91031 | 0.607816 | 0.727723 | 0.513514 | 0.000411 |
| THBD | 0.935178 | 0.781078 | 0.408487 | 0.537129 | 0.324324 | 0.00043 |
| FPR1 | 0.75011 | 0.866657 | 0.515278 | 0.621287 | 0.445946 | 0.00043 |
| CXCL16 | 0.489556 | 1.194236 | 0.850588 | 0.826733 | 0.668919 | 0.000465 |
| SLC66A2 | 0.458262 | 0.887782 | 0.646183 | 0.792079 | 0.641892 | 0.000465 |
| CTDP1 | 0.463491 | 0.936798 | 0.679393 | 0.834158 | 0.662162 | 0.000522 |
| SLC25A19 | 0.641294 | 0.764924 | 0.490422 | 0.668317 | 0.459459 | 0.00053 |
| SLC20A1 | 0.543507 | 0.972215 | 0.667037 | 0.829208 | 0.594595 | 0.000575 |
| CH25H | 1.504221 | 0.496602 | 0.175062 | 0.309406 | 0.121622 | 0.0006 |
| NANS | 0.485713 | 0.885329 | 0.632252 | 0.782178 | 0.581081 | 0.0006 |
| FCAR | 1.22477 | 0.445873 | 0.190774 | 0.339109 | 0.141892 | 0.000861 |
| SEMA6B | 2.267301 | 0.161849 | 0.033619 | 0.205446 | 0.047297 | 0.000906 |
| FMN1 | 0.559255 | 0.817019 | 0.554472 | 0.712871 | 0.527027 | 0.000961 |
| HBEGF | 0.622328 | 1.19093 | 0.773654 | 0.688119 | 0.547297 | 0.000988 |
| TRMT6 | 0.533978 | 0.81701 | 0.564266 | 0.707921 | 0.560811 | 0.001114 |
| RAB20 | 1.242604 | 0.283018 | 0.119606 | 0.341584 | 0.148649 | 0.001248 |
| PIM2 | 1.422597 | 0.199461 | 0.074407 | 0.284653 | 0.101351 | 0.001394 |
| CD14 | 0.622259 | 1.269403 | 0.824671 | 0.688119 | 0.493243 | 0.001552 |
| SKI | 0.30033 | 1.231721 | 1.00024 | 0.893564 | 0.837838 | 0.001581 |
| ARID5A | 0.548318 | 0.668105 | 0.456861 | 0.740099 | 0.52027 | 0.001945 |
| TDG | 1.561141 | 0.180289 | 0.061097 | 0.25495 | 0.087838 | 0.002205 |
| PARP9 | 0.622562 | 0.598264 | 0.388582 | 0.638614 | 0.418919 | 0.002205 |
| SECTM1 | 0.620074 | 0.721305 | 0.469308 | 0.616337 | 0.452703 | 0.002276 |
| THBS1 | 1.350276 | 0.395657 | 0.155183 | 0.292079 | 0.121622 | 0.002379 |
| FOSL1 | 0.713772 | 0.580366 | 0.353863 | 0.549505 | 0.337838 | 0.002383 |
| UBALD2 | 0.596386 | 0.61846 | 0.409055 | 0.675743 | 0.472973 | 0.0027 |
| OXSR1 | 0.330879 | 1.189515 | 0.945726 | 0.893564 | 0.777027 | 0.002852 |
| TNFRSF21 | 0.767261 | 0.376779 | 0.22137 | 0.455446 | 0.236486 | 0.002858 |
| CD300C | 0.830165 | 0.402274 | 0.226265 | 0.472772 | 0.256757 | 0.002937 |
| IER3 | 0.651079 | 1.231962 | 0.784519 | 0.626238 | 0.472973 | 0.003035 |
| GNG10 | 0.309031 | 1.285351 | 1.037514 | 0.89604 | 0.804054 | 0.003035 |
| IL10RA | 0.237641 | 1.74681 | 1.481524 | 0.980198 | 0.912162 | 0.003035 |
| DEF6 | 0.238838 | 1.221462 | 1.035101 | 0.933168 | 0.878378 | 0.003361 |
| PHC2 | 0.378781 | 1.07478 | 0.826599 | 0.816832 | 0.702703 | 0.003633 |
| TSEN34 | 0.622122 | 0.608829 | 0.395565 | 0.618812 | 0.412162 | 0.003662 |
| IFITM2 | 0.271854 | 1.372672 | 1.136922 | 0.935644 | 0.885135 | 0.003668 |
| B3GALNT2 | 0.845525 | 0.358564 | 0.199544 | 0.443069 | 0.236486 | 0.003716 |
| SESN2 | 0.7274 | 0.563514 | 0.340357 | 0.537129 | 0.351351 | 0.00373 |
| OTUD1 | 0.356043 | 1.362445 | 1.064485 | 0.876238 | 0.722973 | 0.003914 |
| MAN1A1 | 0.58768 | 0.555011 | 0.369311 | 0.636139 | 0.445946 | 0.004345 |
| NFIL3 | 0.30383 | 1.591406 | 1.289196 | 0.89604 | 0.804054 | 0.004529 |
| ZC3HAV1 | 0.327544 | 1.371919 | 1.093271 | 0.910891 | 0.858108 | 0.004565 |
| GPR27 | 4.759192 | 0.076009 | 0.002807 | 0.111386 | 0.006757 | 0.004609 |
| TRIB1 | 0.440718 | 1.216745 | 0.896458 | 0.814356 | 0.641892 | 0.004641 |
| ZFY | 2.02407 | 0.126969 | 0.031217 | 0.173267 | 0.040541 | 0.004807 |
| CCNH | 0.4329 | 0.814063 | 0.603035 | 0.727723 | 0.601351 | 0.004951 |
| SAMSN1 | 0.4749 | 1.028512 | 0.740032 | 0.730198 | 0.581081 | 0.005171 |
| HLX | 0.846696 | 0.436879 | 0.242929 | 0.443069 | 0.256757 | 0.005182 |
| UBE2J1 | 0.336994 | 1.076972 | 0.852627 | 0.883663 | 0.763514 | 0.005405 |
| WRN | 2.25777 | 0.080361 | 0.016803 | 0.138614 | 0.02027 | 0.005525 |
| EHD1 | 0.692382 | 0.536332 | 0.331899 | 0.54703 | 0.385135 | 0.005525 |
| FCGR2A | 0.669898 | 0.5506 | 0.34608 | 0.529703 | 0.351351 | 0.005525 |
| CHST11 | 0.359099 | 0.995965 | 0.776506 | 0.834158 | 0.722973 | 0.00595 |
| MAFB | 0.757309 | 0.963658 | 0.570099 | 0.529703 | 0.371622 | 0.005989 |
| NSMF | 0.337357 | 1.041545 | 0.824373 | 0.834158 | 0.722973 | 0.006026 |
| CHSY1 | 0.561784 | 0.609349 | 0.412812 | 0.611386 | 0.418919 | 0.006093 |
| TTLL3 | 0.564224 | 0.54935 | 0.371536 | 0.584158 | 0.432432 | 0.006266 |
| NECTIN2 | 1.056338 | 0.325173 | 0.15636 | 0.351485 | 0.175676 | 0.006523 |
| TBC1D10C | 0.720327 | 0.346482 | 0.210301 | 0.457921 | 0.27027 | 0.007029 |
| ADGRE2 | 0.502136 | 0.760574 | 0.537011 | 0.678218 | 0.486486 | 0.007029 |
| GPCPD1 | 0.248 | 1.368266 | 1.152166 | 0.893564 | 0.837838 | 0.007116 |
| RAB24 | 0.562387 | 0.663346 | 0.449205 | 0.648515 | 0.472973 | 0.007124 |
| PPP1R3B | 1.34354 | 0.193003 | 0.076053 | 0.25 | 0.094595 | 0.007126 |
| BLOC1S1 | 0.306336 | 1.079143 | 0.872695 | 0.89604 | 0.790541 | 0.007126 |
| PLAUR | 0.252526 | 2.156574 | 1.810283 | 0.935644 | 0.864865 | 0.007134 |
| SELENOK | 0.356732 | 1.066887 | 0.833166 | 0.84901 | 0.72973 | 0.007135 |
| PROK2 | 2.714293 | 0.1113 | 0.01696 | 0.131188 | 0.02027 | 0.007166 |
| C5AR1 | 1.532765 | 0.268224 | 0.092702 | 0.230198 | 0.087838 | 0.007349 |
| AFF3 | 1.192055 | 0.176156 | 0.0771 | 0.269802 | 0.108108 | 0.007733 |
| CREM | 0.591032 | 0.660069 | 0.438198 | 0.633663 | 0.445946 | 0.007733 |
| NRG1 | 2.418788 | 0.098172 | 0.01836 | 0.131188 | 0.02027 | 0.007741 |
| EIF4E3 | 0.834232 | 0.414404 | 0.232432 | 0.433168 | 0.256757 | 0.007741 |
| GASK1B | 0.741891 | 0.532505 | 0.318414 | 0.50495 | 0.331081 | 0.007741 |
| PLK3 | 0.308356 | 1.214295 | 0.980618 | 0.851485 | 0.790541 | 0.007741 |
| HSPA6 | 0.575153 | 0.789495 | 0.52992 | 0.638614 | 0.445946 | 0.007826 |
| ZNF703 | 0.683249 | 0.765146 | 0.476503 | 0.539604 | 0.364865 | 0.007883 |
| VSIR | 0.237025 | 1.562098 | 1.325429 | 0.940594 | 0.885135 | 0.008087 |
| PLCG2 | 0.426931 | 0.772116 | 0.574333 | 0.787129 | 0.628378 | 0.00842 |
| QRICH1 | 0.340151 | 0.717579 | 0.566858 | 0.762376 | 0.655405 | 0.00842 |
| NABP1 | 0.738173 | 0.392515 | 0.235312 | 0.435644 | 0.236486 | 0.008599 |
| LILRA5 | 0.531534 | 0.948612 | 0.656267 | 0.680693 | 0.540541 | 0.008599 |
| HERPUD1 | 0.243928 | 1.649529 | 1.392933 | 0.95297 | 0.891892 | 0.008778 |
| MTHFR | 0.579055 | 0.557638 | 0.373284 | 0.616337 | 0.405405 | 0.008943 |
| SLC2A1 | 1.490664 | 0.152668 | 0.054327 | 0.225248 | 0.087838 | 0.008963 |
| TTC19 | 0.413632 | 0.833946 | 0.626069 | 0.779703 | 0.608108 | 0.008963 |
| SOCS1 | 2.808925 | 0.089677 | 0.012797 | 0.126238 | 0.02027 | 0.009165 |
| TSPAN17 | 1.09529 | 0.231329 | 0.108271 | 0.306931 | 0.148649 | 0.009254 |
| MBOAT7 | 0.258096 | 1.583972 | 1.324503 | 0.940594 | 0.844595 | 0.009254 |
| ZNF628 | 1.044483 | 0.21598 | 0.104711 | 0.30198 | 0.141892 | 0.009645 |

^1^log2FC - log fold-change of the average expression between the two groups (FF compared to HMF infants).

^2^FF – formula-fed.

^3^HMF - human milk fed.

^4^pct_FF, percent of cells with detected expression of the gene in FF infants.

^5^pct_HMF, percent of cells with detected expression the gene in HMF infants.

^6^p_val_adj, adjusted p-value based on Benjamini and Hochberg method using all genes in the dataset.

**Supplemental Table 4.** The list of differentially expressed genes in natural killer subsets of FF (n=3) and HMF (n=6) infants.

| **Gene** | **log2FC^1^** | **avg_FF^2^** | **avg_HMF^3^** | **pct_FF^4^** | **pct_HMF^5^** | **p_val_adj^6^** |
| --- | --- | --- | --- | --- | --- | --- |
| **CD56+ CD16- NK cells** | | |  |  |  |  |
| CYTIP | 0.505098 | 1.447498 | 1.019926 | 0.941993 | 0.801187 | 1.11E-23 |
| THEMIS2 | 1.694979 | 0.487054 | 0.150431 | 0.497549 | 0.175074 | 6.3E-23 |
| DDX3Y | 1.14604 | 0.73164 | 0.330602 | 0.658497 | 0.317507 | 1.02E-22 |
| ZFP36 | 0.363073 | 2.33564 | 1.815975 | 0.976307 | 0.902077 | 9.03E-22 |
| CXCR4 | 0.437727 | 2.129945 | 1.572531 | 0.951797 | 0.833828 | 4.44E-20 |
| AREG | 1.028289 | 0.9164 | 0.449303 | 0.669118 | 0.41543 | 7.47E-20 |
| FOSL2 | 0.613981 | 1.250308 | 0.81694 | 0.848039 | 0.667656 | 5.92E-19 |
| DUSP2 | 0.454442 | 1.750287 | 1.277346 | 0.906863 | 0.804154 | 6.69E-18 |
| METRNL | 0.52424 | 1.312745 | 0.912784 | 0.875 | 0.724036 | 1.77E-16 |
| BTG2 | 0.36416 | 1.65656 | 1.287017 | 0.940359 | 0.839763 | 4.5E-15 |
| S1PR5 | 1.888517 | 0.399371 | 0.107864 | 0.338235 | 0.109792 | 2.83E-14 |
| ARL4C | 0.342658 | 1.616366 | 1.274645 | 0.929739 | 0.866469 | 2.83E-14 |
| NR4A2 | 0.436258 | 1.61216 | 1.191465 | 0.89134 | 0.774481 | 8.34E-14 |
| CD3E | 0.379867 | 1.461302 | 1.123023 | 0.909314 | 0.792285 | 4.8E-13 |
| FCGR3A | 1.199413 | 0.649116 | 0.282659 | 0.484477 | 0.264095 | 6.98E-13 |
| GZMB | 0.856393 | 0.901243 | 0.497785 | 0.621732 | 0.385757 | 1.01E-12 |
| MAP3K8 | 0.3727 | 1.507001 | 1.163911 | 0.935458 | 0.821958 | 1.6E-12 |
| ADGRG1 | 0.872167 | 1.000973 | 0.546857 | 0.620098 | 0.421365 | 1.86E-12 |
| PLXNA4 | 1.068115 | 0.516983 | 0.246571 | 0.495098 | 0.267062 | 2.54E-12 |
| DTHD1 | 1.630697 | 0.334743 | 0.108099 | 0.326797 | 0.11276 | 4.14E-12 |
| SLA | 0.544581 | 0.998761 | 0.684741 | 0.805556 | 0.611276 | 5.21E-12 |
| RNF125 | 0.392026 | 1.224829 | 0.933391 | 0.897059 | 0.771513 | 6.54E-12 |
| TNFRSF1B | 1.172553 | 0.403973 | 0.179217 | 0.435458 | 0.225519 | 1.43E-11 |
| FKBP5 | 1.49428 | 0.278865 | 0.098985 | 0.337418 | 0.139466 | 4.8E-11 |
| PDE4D | 0.948142 | 0.532389 | 0.275937 | 0.52451 | 0.311573 | 5.25E-11 |
| CEMIP2 | 1.407949 | 0.293647 | 0.11066 | 0.331699 | 0.127596 | 1.14E-10 |
| ISG20 | 0.529008 | 0.92491 | 0.640992 | 0.786765 | 0.587537 | 1.29E-10 |
| RUNX3 | 0.27192 | 1.611577 | 1.334734 | 0.945261 | 0.890208 | 4.92E-10 |
| PIK3IP1 | 0.918482 | 0.464318 | 0.245655 | 0.49183 | 0.284866 | 5.24E-10 |
| FGL2 | 2.242488 | 0.158141 | 0.033419 | 0.206699 | 0.04451 | 6.79E-10 |
| RBM38 | 0.383529 | 1.232671 | 0.944917 | 0.888072 | 0.756677 | 7.85E-10 |
| DYSF | 1.071924 | 0.372641 | 0.17726 | 0.406863 | 0.20178 | 1.14E-09 |
| CD38 | 0.59123 | 0.803331 | 0.533232 | 0.696078 | 0.507418 | 1.68E-09 |
| SMAP2 | 0.300511 | 1.279705 | 1.039076 | 0.89951 | 0.818991 | 2.1E-09 |
| CCL4 | 1.895736 | 0.454805 | 0.122223 | 0.278595 | 0.109792 | 5.11E-09 |
| RGS2 | 0.942269 | 0.488616 | 0.254283 | 0.460784 | 0.27003 | 5.51E-09 |
| KLF3 | 0.533559 | 0.795469 | 0.549549 | 0.713235 | 0.531157 | 5.94E-09 |
| ZBTB16 | 0.70535 | 0.682256 | 0.418423 | 0.578431 | 0.379822 | 8.57E-09 |
| REL | 0.355634 | 1.189353 | 0.92951 | 0.873366 | 0.771513 | 9.63E-09 |
| SOCS1 | 0.849254 | 0.457229 | 0.253795 | 0.482026 | 0.296736 | 1.39E-08 |
| PSMB9 | 0.397143 | 1.013063 | 0.76928 | 0.829248 | 0.68546 | 2.84E-08 |
| H1-3 | 0.816635 | 0.515532 | 0.292701 | 0.488562 | 0.290801 | 3.33E-08 |
| ZEB2 | 0.555899 | 0.91388 | 0.621651 | 0.666667 | 0.501484 | 2.29E-07 |
| CEBPB | 1.827628 | 0.156421 | 0.044068 | 0.19281 | 0.05638 | 2.95E-07 |
| KLF13 | 0.348471 | 1.029027 | 0.808215 | 0.848856 | 0.732938 | 3.38E-07 |
| IRS2 | 0.510165 | 0.815672 | 0.572718 | 0.696078 | 0.534125 | 3.81E-07 |
| CST7 | 0.235121 | 1.690472 | 1.436249 | 0.945261 | 0.881306 | 5.2E-07 |
| CDKN1B | 0.5244 | 0.704628 | 0.489891 | 0.660131 | 0.486647 | 6.35E-07 |
| GZMA | 0.551798 | 1.032851 | 0.704579 | 0.634804 | 0.445104 | 8.25E-07 |
| GZMM | 0.491736 | 0.887594 | 0.631229 | 0.685458 | 0.525223 | 9.03E-07 |
| CD55 | 0.409466 | 0.870484 | 0.655389 | 0.772876 | 0.637982 | 9.47E-07 |
| B3GNT7 | 0.558085 | 0.634317 | 0.43083 | 0.627451 | 0.451039 | 9.6E-07 |
| TRBC1 | 0.59411 | 0.777556 | 0.515094 | 0.595588 | 0.454006 | 1.31E-06 |
| PRDM1 | 2.718806 | 0.146242 | 0.022214 | 0.148693 | 0.035608 | 1.32E-06 |
| RASA2 | 0.564427 | 0.496569 | 0.335792 | 0.558824 | 0.391691 | 1.57E-06 |
| GFOD1 | 0.499468 | 0.757306 | 0.535694 | 0.688725 | 0.507418 | 1.62E-06 |
| CLK1 | 0.316563 | 1.129108 | 0.906652 | 0.879902 | 0.774481 | 1.69E-06 |
| CEBPD | 0.693507 | 0.468455 | 0.289668 | 0.473856 | 0.287834 | 1.83E-06 |
| TP53INP2 | 1.178688 | 0.221811 | 0.097986 | 0.267157 | 0.118694 | 1.93E-06 |
| KDM5D | 1.222114 | 0.191227 | 0.08197 | 0.25 | 0.106825 | 2.39E-06 |
| FGFBP2 | 0.957448 | 0.635912 | 0.327473 | 0.434641 | 0.296736 | 3.05E-06 |
| NR4A3 | 0.577817 | 0.620322 | 0.415601 | 0.585784 | 0.41543 | 3.67E-06 |
| SPON2 | 1.409944 | 0.331645 | 0.124807 | 0.25 | 0.115727 | 4.58E-06 |
| EIF1AY | 1.324336 | 0.16368 | 0.065363 | 0.211601 | 0.083086 | 7.99E-06 |
| DCXR | 0.812879 | 0.327159 | 0.186234 | 0.387255 | 0.234421 | 8.19E-06 |
| CEP78 | 0.450332 | 0.780697 | 0.571372 | 0.699346 | 0.548961 | 9.3E-06 |
| ZBTB1 | 0.402319 | 0.810948 | 0.613596 | 0.754085 | 0.593472 | 9.9E-06 |
| PDZD8 | 0.517818 | 0.564869 | 0.39452 | 0.594771 | 0.436202 | 1.07E-05 |
| SH2D2A | 0.584032 | 0.516889 | 0.344815 | 0.533497 | 0.382789 | 1.13E-05 |
| ERBB2 | 1.398119 | 0.203188 | 0.077094 | 0.212418 | 0.086053 | 1.2E-05 |
| LGALS9C | 1.296528 | 0.162058 | 0.065974 | 0.214052 | 0.089021 | 1.67E-05 |
| NSMF | 1.118431 | 0.221961 | 0.102234 | 0.270425 | 0.139466 | 1.97E-05 |
| ENC1 | 1.042587 | 0.296617 | 0.143995 | 0.305556 | 0.172107 | 2.19E-05 |
| SH2D1B | 0.304449 | 1.049886 | 0.850147 | 0.822712 | 0.724036 | 2.21E-05 |
| TENT5C | 0.561002 | 0.571235 | 0.387201 | 0.536765 | 0.385757 | 2.94E-05 |
| UTY | 0.753214 | 0.291522 | 0.172954 | 0.353758 | 0.198813 | 3.32E-05 |
| NEIL1 | 0.747981 | 0.356391 | 0.212208 | 0.38317 | 0.234421 | 3.86E-05 |
| BRD1 | 0.312345 | 0.970068 | 0.781227 | 0.811275 | 0.703264 | 4.1E-05 |
| TAMALIN | 0.783143 | 0.353158 | 0.20522 | 0.369281 | 0.231454 | 5.32E-05 |
| TLE1 | 0.630808 | 0.462088 | 0.298423 | 0.482026 | 0.338279 | 5.54E-05 |
| PTGDR | 0.42729 | 0.659665 | 0.490565 | 0.656046 | 0.498516 | 9.9E-05 |
| USP9Y | 1.137584 | 0.163792 | 0.074447 | 0.220588 | 0.10089 | 0.0001 |
| SLC7A5 | 0.811938 | 0.307911 | 0.175391 | 0.345588 | 0.21365 | 0.000101 |
| CHST2 | 0.393628 | 0.786121 | 0.598405 | 0.704248 | 0.554896 | 0.000102 |
| CHST11 | 0.452697 | 0.582743 | 0.425796 | 0.603758 | 0.451039 | 0.000114 |
| ATF3 | 1.305894 | 0.140887 | 0.056985 | 0.176471 | 0.068249 | 0.000114 |
| SLAMF6 | 0.485711 | 0.555364 | 0.396611 | 0.560458 | 0.412463 | 0.000121 |
| H2AC15 | 1.780258 | 0.106126 | 0.030897 | 0.142974 | 0.047478 | 0.000129 |
| PDZD4 | 1.135029 | 0.184102 | 0.083826 | 0.209967 | 0.094955 | 0.000129 |
| DDIT4 | 0.282373 | 1.297718 | 1.067033 | 0.879902 | 0.762611 | 0.000138 |
| DENND6A | 0.497037 | 0.465792 | 0.330042 | 0.522876 | 0.364985 | 0.000139 |
| TNFSF9 | 1.103181 | 0.23317 | 0.108538 | 0.246732 | 0.127596 | 0.000141 |
| PPP1CB | 0.398844 | 0.595342 | 0.451547 | 0.648693 | 0.495549 | 0.000146 |
| TIGIT | 0.753291 | 0.340414 | 0.20195 | 0.328431 | 0.189911 | 0.000182 |
| NTPCR | 1.121623 | 0.176677 | 0.081197 | 0.230392 | 0.115727 | 0.000205 |
| KMT2E | 0.272058 | 1.010034 | 0.836447 | 0.844771 | 0.72997 | 0.000222 |
| ATP9A | 1.128772 | 0.154257 | 0.070542 | 0.200163 | 0.089021 | 0.000224 |
| PPP2R5C | 0.49 | 0.530986 | 0.378075 | 0.571078 | 0.442136 | 0.000226 |
| SLC16A3 | 0.419924 | 0.631449 | 0.471985 | 0.628268 | 0.507418 | 0.000233 |
| LYAR | 0.749571 | 0.28368 | 0.168727 | 0.334967 | 0.204748 | 0.000312 |
| H2BC21 | 1.092631 | 0.190118 | 0.089147 | 0.227941 | 0.118694 | 0.000319 |
| GIMAP8 | 0.817941 | 0.230026 | 0.130482 | 0.278595 | 0.154303 | 0.000342 |
| GGNBP2 | 0.395221 | 0.591262 | 0.44958 | 0.620915 | 0.48368 | 0.000348 |
| DYNLT1 | 0.562966 | 0.403124 | 0.272878 | 0.464052 | 0.341246 | 0.000362 |
| MSL2 | 0.268181 | 0.952346 | 0.790795 | 0.803105 | 0.718101 | 0.000372 |
| LBH | 0.574652 | 0.495741 | 0.332864 | 0.47549 | 0.347181 | 0.000382 |
| GALC | 0.554929 | 0.395166 | 0.268986 | 0.45915 | 0.332344 | 0.000414 |
| HAVCR2 | 0.545482 | 0.528135 | 0.361858 | 0.500817 | 0.37092 | 0.000418 |
| NFKBIB | 0.759252 | 0.259566 | 0.153352 | 0.319444 | 0.195846 | 0.000442 |
| CMKLR1 | 65535 | 0.052908 | 0 | 0.056373 | 0 | 0.000508 |
| TAGAP | 0.219487 | 1.444086 | 1.240284 | 0.906863 | 0.836795 | 0.000518 |
| ATG9A | 0.819923 | 0.226064 | 0.128059 | 0.294118 | 0.175074 | 0.000567 |
| PYHIN1 | 0.370878 | 0.859049 | 0.664312 | 0.681373 | 0.551929 | 0.000584 |
| ACTN4 | 0.267688 | 0.948158 | 0.787587 | 0.804739 | 0.712166 | 0.0006 |
| ARID5B | 0.461915 | 0.563459 | 0.409083 | 0.552288 | 0.403561 | 0.000601 |
| CCL5 | 0.445987 | 1.169417 | 0.858448 | 0.59232 | 0.489614 | 0.00066 |
| PIK3R5 | 0.332834 | 0.81957 | 0.650719 | 0.735294 | 0.605341 | 0.00066 |
| TFEB | 0.80964 | 0.20893 | 0.1192 | 0.261438 | 0.145401 | 0.000729 |
| CNOT6L | 0.253893 | 1.086615 | 0.911269 | 0.857026 | 0.762611 | 0.000794 |
| COLGALT2 | 2.538464 | 0.058294 | 0.010034 | 0.076797 | 0.011869 | 0.000816 |
| ZBED4 | 0.60503 | 0.34361 | 0.225909 | 0.403595 | 0.281899 | 0.00088 |
| ATF7IP | 0.44922 | 0.471755 | 0.345532 | 0.526961 | 0.397626 | 0.000913 |
| IDI1 | 0.566375 | 0.373591 | 0.25229 | 0.435458 | 0.317507 | 0.000957 |
| RHOH | 0.445341 | 0.511413 | 0.375588 | 0.527778 | 0.418398 | 0.001011 |
| SLAMF7 | 0.416778 | 0.567051 | 0.424776 | 0.565359 | 0.421365 | 0.001224 |
| CCND3 | 0.209603 | 1.247862 | 1.079121 | 0.896242 | 0.813056 | 0.001236 |
| CLIC3 | 0.519468 | 0.73632 | 0.513678 | 0.534314 | 0.430267 | 0.001269 |
| CRACR2B | 1.285672 | 0.12332 | 0.050583 | 0.15768 | 0.068249 | 0.001306 |
| SMAGP | 0.819717 | 0.210293 | 0.119142 | 0.254902 | 0.142433 | 0.001334 |
| TGFBR3 | 0.39524 | 0.628259 | 0.477705 | 0.612745 | 0.501484 | 0.001559 |
| CREM | 0.559041 | 0.384313 | 0.260854 | 0.416667 | 0.296736 | 0.001564 |
| ITM2A | 0.466449 | 0.447722 | 0.324036 | 0.471405 | 0.341246 | 0.001614 |
| CD247 | 0.235703 | 1.251644 | 1.062986 | 0.871732 | 0.783383 | 0.001629 |
| TP53INP1 | 0.565807 | 0.503973 | 0.340473 | 0.464869 | 0.364985 | 0.001703 |
| KLF9 | 0.480876 | 0.510785 | 0.365999 | 0.511438 | 0.391691 | 0.001757 |
| SLC5A6 | 0.704318 | 0.280797 | 0.172335 | 0.331699 | 0.216617 | 0.001762 |
| MYBL1 | 0.511475 | 0.453889 | 0.318406 | 0.465686 | 0.347181 | 0.001799 |
| ITPRIP | 0.501387 | 0.481298 | 0.340002 | 0.489379 | 0.373887 | 0.001833 |
| RMDN3 | 0.412527 | 0.504677 | 0.379167 | 0.554739 | 0.430267 | 0.001909 |
| SYNE2 | 0.774424 | 0.429638 | 0.251176 | 0.35866 | 0.255193 | 0.001969 |
| GFI1 | 0.932654 | 0.186821 | 0.097874 | 0.231209 | 0.130564 | 0.002038 |
| NPC1 | 0.866786 | 0.224557 | 0.12314 | 0.263072 | 0.163205 | 0.002038 |
| ZAP70 | 0.167075 | 1.526574 | 1.359638 | 0.949346 | 0.890208 | 0.002038 |
| IVNS1ABP | 0.219867 | 1.046814 | 0.898841 | 0.838235 | 0.768546 | 0.00204 |
| BCL11B | 1.178713 | 0.122092 | 0.053934 | 0.146242 | 0.062315 | 0.002062 |
| EOLA1 | 0.686315 | 0.275775 | 0.171377 | 0.34232 | 0.231454 | 0.002072 |
| CYBRD1 | 0.80855 | 0.186316 | 0.106378 | 0.223856 | 0.118694 | 0.002276 |
| HIPK2 | 0.615802 | 0.318035 | 0.207539 | 0.361111 | 0.246291 | 0.002276 |
| PSTPIP1 | 0.275688 | 0.938547 | 0.775292 | 0.79902 | 0.670623 | 0.002276 |
| TMEM140 | 0.976969 | 0.159105 | 0.080833 | 0.19281 | 0.097923 | 0.002567 |
| ZNF331 | 0.733171 | 0.229186 | 0.137874 | 0.268791 | 0.163205 | 0.002629 |
| JMY | 0.489206 | 0.338768 | 0.241344 | 0.406046 | 0.290801 | 0.002638 |
| PMAIP1 | 0.642092 | 0.431888 | 0.276746 | 0.392157 | 0.284866 | 0.002639 |
| GTPBP1 | 0.445985 | 0.434186 | 0.318728 | 0.50817 | 0.37092 | 0.002711 |
| MT2A | 1.164843 | 0.112739 | 0.050283 | 0.142974 | 0.059347 | 0.002797 |
| MT-ND2 | 0.280407 | 1.278951 | 1.053036 | 0.845588 | 0.682493 | 0.002933 |
| CHD1 | 0.407897 | 0.488467 | 0.368168 | 0.549837 | 0.430267 | 0.003115 |
| ENGASE | 0.732567 | 0.246718 | 0.148483 | 0.297386 | 0.195846 | 0.003205 |
| ELL2 | 0.531609 | 0.341775 | 0.236434 | 0.397876 | 0.275964 | 0.003452 |
| LDLR | 1.230671 | 0.114313 | 0.048711 | 0.134804 | 0.05638 | 0.003528 |
| AGK | 0.351973 | 0.602512 | 0.472075 | 0.606209 | 0.477745 | 0.003528 |
| PLAAT4 | 0.244386 | 1.195276 | 1.009022 | 0.829248 | 0.735905 | 0.003785 |
| SKI | 0.256552 | 0.949772 | 0.795041 | 0.801471 | 0.697329 | 0.004455 |
| SELENOK | 0.380108 | 0.450761 | 0.346356 | 0.520425 | 0.376855 | 0.004546 |
| SYTL3 | 0.425363 | 0.50587 | 0.376697 | 0.528595 | 0.424332 | 0.004623 |
| RBM39 | 0.288209 | 0.748415 | 0.612891 | 0.739379 | 0.605341 | 0.004772 |
| MX1 | 0.612154 | 0.18993 | 0.124256 | 0.229575 | 0.130564 | 0.004983 |
| MRE11 | 0.828064 | 0.158777 | 0.089436 | 0.218137 | 0.124629 | 0.00507 |
| MTSS1 | 1.128244 | 0.134094 | 0.061344 | 0.156863 | 0.077151 | 0.005103 |
| USP36 | 0.550897 | 0.345053 | 0.235532 | 0.400327 | 0.293769 | 0.005263 |
| ZNF831 | 0.495217 | 0.353522 | 0.250808 | 0.41585 | 0.296736 | 0.005334 |
| MADD | 0.351413 | 0.627238 | 0.491639 | 0.623366 | 0.531157 | 0.005336 |
| NFE2L2 | 0.346534 | 0.615837 | 0.484338 | 0.615196 | 0.510386 | 0.005336 |
| FAM177A1 | 0.444154 | 0.400099 | 0.294079 | 0.458333 | 0.347181 | 0.005498 |
| JOSD1 | 0.344978 | 0.542539 | 0.427151 | 0.574346 | 0.456973 | 0.005634 |
| TMEM50A | 0.306698 | 0.661039 | 0.534444 | 0.679739 | 0.545994 | 0.005794 |
| LEPROTL1 | 0.29033 | 0.681957 | 0.557646 | 0.673203 | 0.543027 | 0.005794 |
| TGIF1 | 1.044518 | 0.113046 | 0.054805 | 0.148693 | 0.068249 | 0.006211 |
| TXK | 0.273894 | 0.790198 | 0.653561 | 0.728758 | 0.637982 | 0.006283 |
| FCRL6 | 0.511409 | 0.474445 | 0.332841 | 0.417484 | 0.308605 | 0.006331 |
| SSBP3 | 0.564324 | 0.304539 | 0.205951 | 0.349673 | 0.243323 | 0.0065 |
| MDM4 | 0.248796 | 0.888256 | 0.747555 | 0.786765 | 0.676558 | 0.00661 |
| MYO1F | 0.186404 | 1.295825 | 1.138763 | 0.910131 | 0.836795 | 0.006724 |
| TSPYL2 | 0.736515 | 0.285918 | 0.171604 | 0.303922 | 0.204748 | 0.00685 |
| RBM12 | 0.625924 | 0.215068 | 0.139365 | 0.287582 | 0.181009 | 0.00709 |
| INSIG1 | 0.557745 | 0.315866 | 0.214587 | 0.377451 | 0.278932 | 0.007328 |
| ZBP1 | 0.432384 | 0.447564 | 0.331661 | 0.478758 | 0.367953 | 0.00735 |
| VPS26A | 0.410738 | 0.391468 | 0.294477 | 0.45915 | 0.347181 | 0.00735 |
| CTDP1 | 0.303081 | 0.69366 | 0.562225 | 0.676471 | 0.551929 | 0.007691 |
| LRRC8B | 1.535063 | 0.077252 | 0.026657 | 0.099673 | 0.035608 | 0.0077 |
| MAP2K3 | 0.31085 | 0.667444 | 0.538071 | 0.662582 | 0.551929 | 0.007843 |
| FASLG | 0.875433 | 0.225614 | 0.12298 | 0.227941 | 0.139466 | 0.007976 |
| CD99 | 0.263161 | 0.796118 | 0.663374 | 0.727124 | 0.635015 | 0.008361 |
| SIPA1L2 | 1.942372 | 0.053384 | 0.01389 | 0.071078 | 0.017804 | 0.008379 |
| ATG2A | 0.668986 | 0.172378 | 0.108417 | 0.230392 | 0.133531 | 0.008379 |
| TMCC3 | 2.785453 | 0.043417 | 0.006297 | 0.056373 | 0.008902 | 0.008456 |
| MEF2D | 0.326118 | 0.571101 | 0.455556 | 0.617647 | 0.51632 | 0.008527 |
| CCDC88C | 0.221499 | 0.947235 | 0.812419 | 0.818627 | 0.72997 | 0.008754 |
| CENPC | 0.630515 | 0.227386 | 0.146879 | 0.290033 | 0.195846 | 0.009302 |
| LPCAT1 | 0.227962 | 1.153833 | 0.985189 | 0.844771 | 0.72997 | 0.009302 |
| ITK | 0.367288 | 0.511227 | 0.396323 | 0.544118 | 0.430267 | 0.009466 |
| WHRN | 0.717378 | 0.274491 | 0.166946 | 0.292484 | 0.207715 | 0.009692 |
| BMP2 | 1.81818 | 0.046958 | 0.013316 | 0.061275 | 0.011869 | 0.009749 |
| SQSTM1 | 0.226469 | 0.932698 | 0.7972 | 0.810458 | 0.709199 | 0.009757 |
| IL4R | 0.778532 | 0.189595 | 0.110526 | 0.240196 | 0.151335 | 0.009761 |
| ZFY | 1.312345 | 0.093708 | 0.037733 | 0.128268 | 0.059347 | 0.00989 |
| TMEM259 | 0.326834 | 0.554494 | 0.442089 | 0.60049 | 0.480712 | 0.009894 |
| **CD56- CD16+ NK cells** | | |  |  |  |  |
| THEMIS2 | 1.427862 | 0.79391 | 0.295081 | 0.716312 | 0.329394 | 2.1E-201 |
| MT-ND2 | 1.186002 | 1.189241 | 0.522694 | 0.808719 | 0.364851 | 8E-193 |
| BTG1 | 0.397863 | 2.039302 | 1.547793 | 0.966625 | 0.90853 | 3E-181 |
| DDX3Y | 2.290154 | 0.322807 | 0.065999 | 0.356279 | 0.076567 | 2.4E-114 |
| CXCR4 | 0.752816 | 1.591157 | 0.944263 | 0.803296 | 0.554985 | 8.3E-106 |
| CYTIP | 0.449235 | 1.358769 | 0.995204 | 0.910513 | 0.800617 | 2.6E-101 |
| CD3E | 0.922216 | 0.889506 | 0.46939 | 0.596996 | 0.381295 | 3.47E-79 |
| KLRG1 | 1.08427 | 0.527608 | 0.248836 | 0.492908 | 0.238952 | 2.9E-73 |
| UTY | 2.297013 | 0.170247 | 0.034643 | 0.208803 | 0.043165 | 9.27E-60 |
| TSC22D3 | 0.270001 | 1.996571 | 1.655794 | 0.940968 | 0.881295 | 9.59E-59 |
| PRDM1 | 0.736897 | 0.684367 | 0.41064 | 0.619107 | 0.423433 | 7.01E-56 |
| ZFP36 | 0.310968 | 1.934155 | 1.559123 | 0.924072 | 0.841727 | 3.07E-53 |
| CD55 | 0.833116 | 0.415192 | 0.233054 | 0.468502 | 0.284173 | 1.34E-43 |
| FGL2 | 1.160593 | 0.227361 | 0.101705 | 0.292032 | 0.121788 | 2.2E-43 |
| KDM5D | 2.286179 | 0.127813 | 0.026204 | 0.159992 | 0.033916 | 3.17E-43 |
| PIK3IP1 | 0.839734 | 0.38179 | 0.213324 | 0.425323 | 0.249229 | 7.05E-39 |
| RUNX3 | 0.269087 | 1.30719 | 1.084764 | 0.876095 | 0.809353 | 1.04E-36 |
| EIF1AY | 2.599064 | 0.090708 | 0.014971 | 0.122236 | 0.021069 | 6.25E-36 |
| FOSL2 | 0.371352 | 1.008227 | 0.779418 | 0.78932 | 0.684481 | 1.02E-35 |
| KLF13 | 0.364734 | 0.907566 | 0.704827 | 0.783688 | 0.678314 | 4.73E-35 |
| DDIT4 | 0.345019 | 1.15958 | 0.912935 | 0.829161 | 0.715313 | 1.36E-34 |
| PDE4D | 0.452643 | 0.79717 | 0.582495 | 0.698164 | 0.566804 | 1.09E-33 |
| SOCS1 | 1.128604 | 0.224611 | 0.102728 | 0.263663 | 0.122816 | 4.68E-33 |
| DUSP2 | 0.535655 | 0.763293 | 0.526554 | 0.598665 | 0.451182 | 4.94E-30 |
| USP9Y | 2.254016 | 0.087038 | 0.018247 | 0.116187 | 0.025694 | 1.28E-29 |
| CEBPB | 0.939481 | 0.237357 | 0.123763 | 0.284731 | 0.14851 | 5.73E-29 |
| METRNL | 0.275343 | 1.193544 | 0.98617 | 0.846266 | 0.768756 | 1.46E-28 |
| AREG | 1.066805 | 0.235937 | 0.11263 | 0.253025 | 0.125385 | 2.1E-28 |
| ARID5B | 0.962038 | 0.262351 | 0.134673 | 0.300584 | 0.166495 | 2.47E-28 |
| ITM2A | 0.906899 | 0.250133 | 0.133403 | 0.297664 | 0.159815 | 3.2E-28 |
| PDE4B | 1.655657 | 0.111949 | 0.035532 | 0.149771 | 0.049846 | 4.08E-28 |
| CEMIP2 | 0.56019 | 0.528514 | 0.358445 | 0.542553 | 0.403392 | 4.08E-28 |
| ACTN4 | 0.393455 | 0.759807 | 0.578445 | 0.708177 | 0.587359 | 5.63E-28 |
| TNFRSF1B | 0.471156 | 0.612304 | 0.441708 | 0.622028 | 0.47739 | 1.49E-27 |
| MAP3K8 | 0.399455 | 0.815739 | 0.618448 | 0.702128 | 0.572456 | 3.9E-25 |
| PSMB9 | 0.34264 | 0.786356 | 0.620117 | 0.720275 | 0.610997 | 1.76E-23 |
| NEIL1 | 1.460711 | 0.121319 | 0.044077 | 0.140175 | 0.049846 | 3.22E-23 |
| KLF3 | 0.38749 | 0.680075 | 0.519889 | 0.656237 | 0.540082 | 8.44E-22 |
| PLXNA4 | 1.049631 | 0.190468 | 0.092013 | 0.208594 | 0.106372 | 4.71E-21 |
| LAIR1 | 0.440597 | 0.521242 | 0.384067 | 0.554234 | 0.418294 | 2.11E-20 |
| ISG20 | 0.45629 | 0.544551 | 0.3969 | 0.554234 | 0.428571 | 2.31E-20 |
| FXYD5 | 0.288549 | 0.904397 | 0.740452 | 0.758031 | 0.657246 | 6.7E-20 |
| TMEM273 | 1.505213 | 0.093255 | 0.032852 | 0.11869 | 0.042138 | 1.49E-19 |
| DTHD1 | 0.681627 | 0.285121 | 0.177762 | 0.310805 | 0.194758 | 5.21E-19 |
| MT2A | 0.881068 | 0.166956 | 0.090652 | 0.217981 | 0.117163 | 9.14E-19 |
| MAD1L1 | 0.633756 | 0.304666 | 0.196356 | 0.36796 | 0.252826 | 2.23E-18 |
| SYTL3 | 0.534285 | 0.374025 | 0.258264 | 0.426783 | 0.302158 | 3.81E-18 |
| OSBPL5 | 0.496371 | 0.43145 | 0.30585 | 0.477055 | 0.357143 | 9.7E-18 |
| SLA | 0.317435 | 0.752094 | 0.603552 | 0.687735 | 0.59147 | 1E-17 |
| ZNF831 | 0.662672 | 0.252349 | 0.15941 | 0.309971 | 0.200925 | 8.84E-17 |
| TAGAP | 0.227646 | 1.079568 | 0.92198 | 0.798915 | 0.739979 | 1.06E-16 |
| DEF6 | 0.238678 | 0.902395 | 0.764799 | 0.771798 | 0.698869 | 4.59E-16 |
| KCNAB2 | 0.270226 | 0.871643 | 0.722757 | 0.742595 | 0.657246 | 6.01E-16 |
| PER1 | 0.551752 | 0.332757 | 0.227004 | 0.385899 | 0.279548 | 1.25E-15 |
| GRIK4 | 1.508401 | 0.064061 | 0.022518 | 0.086984 | 0.028263 | 2.76E-15 |
| CDKN1B | 0.302156 | 0.742915 | 0.602534 | 0.68106 | 0.58222 | 3.27E-15 |
| FKBP5 | 0.844545 | 0.16652 | 0.092732 | 0.211514 | 0.124872 | 4.65E-15 |
| SAMHD1 | 0.177806 | 1.241416 | 1.097469 | 0.873383 | 0.815519 | 8.5E-15 |
| TFEB | 0.679814 | 0.223583 | 0.139571 | 0.276179 | 0.18037 | 9.08E-15 |
| SH2D2A | 0.340532 | 0.595033 | 0.469927 | 0.599082 | 0.492806 | 1.56E-14 |
| YWHAB | 0.496056 | 0.293036 | 0.207775 | 0.380058 | 0.277492 | 2.53E-14 |
| TBC1D10C | 0.229787 | 0.955992 | 0.815233 | 0.790363 | 0.709147 | 3.67E-14 |
| NPC1 | 0.40018 | 0.460713 | 0.349112 | 0.510221 | 0.396711 | 4.39E-14 |
| PHF19 | 0.687967 | 0.193765 | 0.120275 | 0.230496 | 0.141316 | 5.76E-14 |
| SMAP2 | 0.35799 | 0.546667 | 0.426537 | 0.574885 | 0.478931 | 2.87E-13 |
| ELL2 | 0.738372 | 0.162441 | 0.09737 | 0.209428 | 0.127441 | 6.09E-13 |
| ENC1 | 0.459939 | 0.344238 | 0.250266 | 0.383396 | 0.276465 | 6.09E-13 |
| ZFY | 2.479339 | 0.038834 | 0.006964 | 0.052357 | 0.011305 | 7.69E-13 |
| REL | 0.270051 | 0.728309 | 0.60398 | 0.686483 | 0.597122 | 8.94E-13 |
| CD69 | 0.216766 | 1.244467 | 1.070854 | 0.802253 | 0.738952 | 1.05E-12 |
| ENGASE | 0.897985 | 0.127878 | 0.068624 | 0.166458 | 0.095067 | 2.02E-12 |
| SLC16A3 | 0.523067 | 0.306978 | 0.213623 | 0.359825 | 0.270298 | 2.56E-12 |
| RBM38 | 0.202928 | 1.023736 | 0.889407 | 0.805173 | 0.738952 | 4.98E-12 |
| ATP5PO | 1.383575 | 0.044535 | 0.017069 | 0.069879 | 0.023124 | 6.61E-12 |
| SYTL2 | 0.467145 | 0.315118 | 0.227955 | 0.36796 | 0.273381 | 7.87E-12 |
| TLE1 | 0.410465 | 0.396775 | 0.298526 | 0.443054 | 0.346865 | 1.1E-11 |
| SKAP2 | 0.551181 | 0.224004 | 0.152874 | 0.289529 | 0.202467 | 1.38E-11 |
| ITGAX | 0.289033 | 0.85101 | 0.696509 | 0.647059 | 0.559609 | 1.59E-11 |
| BRD1 | 0.320969 | 0.547139 | 0.438002 | 0.571756 | 0.482014 | 2.66E-11 |
| VIM | 0.23771 | 0.738037 | 0.625922 | 0.673133 | 0.520041 | 2.8E-11 |
| VSIR | 0.544572 | 0.233618 | 0.160167 | 0.296204 | 0.208119 | 2.9E-11 |
| CCL3 | 0.355986 | 0.686072 | 0.536053 | 0.465373 | 0.365365 | 4.93E-11 |
| RUNX1 | 0.390032 | 0.354202 | 0.270296 | 0.419483 | 0.323227 | 5.12E-11 |
| PSTPIP1 | 0.333136 | 0.509236 | 0.404236 | 0.533375 | 0.430627 | 5.62E-11 |
| BCR | 0.424867 | 0.281122 | 0.20941 | 0.35461 | 0.256423 | 5.79E-11 |
| F2R | 0.671019 | 0.135447 | 0.085069 | 0.184814 | 0.110997 | 6.26E-11 |
| ZBTB16 | 0.183327 | 1.0459 | 0.921093 | 0.806633 | 0.754368 | 6.92E-11 |
| RRP7A | 0.822642 | 0.105955 | 0.059907 | 0.147893 | 0.083248 | 1.18E-10 |
| PLEKHG3 | 0.212637 | 0.891209 | 0.769076 | 0.767835 | 0.696814 | 1.35E-10 |
| SIGLEC9 | 0.910788 | 0.086386 | 0.045948 | 0.112849 | 0.056012 | 1.52E-10 |
| ZNF394 | 0.340116 | 0.472174 | 0.373007 | 0.512098 | 0.424974 | 1.53E-10 |
| HEG1 | 0.460411 | 0.265496 | 0.192957 | 0.341469 | 0.254368 | 2.12E-10 |
| TC2N | 0.464145 | 0.254618 | 0.184573 | 0.289737 | 0.201953 | 2.18E-10 |
| PDLIM1 | 0.997481 | 0.069908 | 0.035015 | 0.10096 | 0.048304 | 2.33E-10 |
| NFIL3 | 0.330956 | 0.459409 | 0.365234 | 0.486859 | 0.388489 | 2.39E-10 |
| TMC6 | 0.295124 | 0.544085 | 0.44343 | 0.580726 | 0.48407 | 2.48E-10 |
| RNF125 | 0.199727 | 0.884249 | 0.769929 | 0.761577 | 0.696814 | 2.99E-10 |
| CEBPD | 0.277996 | 0.604576 | 0.498615 | 0.580309 | 0.485612 | 3.07E-10 |
| SSH2 | 0.457413 | 0.271926 | 0.198041 | 0.337505 | 0.249229 | 3.13E-10 |
| HS3ST3B1 | 0.590771 | 0.173708 | 0.11534 | 0.219441 | 0.143885 | 3.19E-10 |
| RAP2B | 0.326897 | 0.512463 | 0.408561 | 0.548602 | 0.460432 | 3.68E-10 |
| RNF44 | 0.234172 | 0.747488 | 0.635494 | 0.696913 | 0.627441 | 4.71E-10 |
| FRAT2 | 0.447518 | 0.234622 | 0.172049 | 0.294952 | 0.206578 | 5.14E-10 |
| FCRL3 | 0.372627 | 0.390472 | 0.301591 | 0.429287 | 0.337616 | 7.16E-10 |
| FTH1 | 0.152897 | 1.196245 | 1.075954 | 0.884439 | 0.822713 | 8.18E-10 |
| IRAK3 | 1.534955 | 0.043454 | 0.014995 | 0.061535 | 0.022097 | 1.02E-09 |
| GZMM | 0.155334 | 1.126307 | 1.011338 | 0.843346 | 0.789825 | 1.08E-09 |
| CCL5 | 0.191853 | 1.633508 | 1.430104 | 0.704839 | 0.638746 | 1.25E-09 |
| GPR65 | 0.274992 | 0.567473 | 0.468991 | 0.581977 | 0.502569 | 1.33E-09 |
| IFNGR1 | 0.645374 | 0.156699 | 0.100182 | 0.20776 | 0.138232 | 1.5E-09 |
| GPATCH8 | 0.401934 | 0.318224 | 0.240845 | 0.384439 | 0.292909 | 1.5E-09 |
| CHST11 | 0.289342 | 0.526468 | 0.430796 | 0.560909 | 0.470195 | 1.97E-09 |
| CDC25B | 0.371823 | 0.359485 | 0.277812 | 0.405507 | 0.319116 | 2.05E-09 |
| WWC3 | 0.502521 | 0.19595 | 0.138315 | 0.252607 | 0.174203 | 2.29E-09 |
| GIMAP1 | 0.386971 | 0.356524 | 0.272646 | 0.401544 | 0.313977 | 2.48E-09 |
| RNF166 | 0.380492 | 0.335982 | 0.258093 | 0.403004 | 0.31295 | 2.6E-09 |
| EOLA1 | 0.665082 | 0.132577 | 0.08361 | 0.182937 | 0.117677 | 2.96E-09 |
| PIM2 | 0.347884 | 0.456495 | 0.358684 | 0.473091 | 0.384892 | 3.83E-09 |
| TAMALIN | 0.562731 | 0.22226 | 0.150474 | 0.261786 | 0.191161 | 4.85E-09 |
| KAT2B | 0.308751 | 0.456673 | 0.368691 | 0.512307 | 0.42446 | 5.39E-09 |
| DPYD | 0.391406 | 0.34578 | 0.263618 | 0.401961 | 0.321686 | 5.76E-09 |
| JUN | 0.218513 | 1.085698 | 0.933104 | 0.702336 | 0.622302 | 5.93E-09 |
| SDSL | 4.548957 | 0.016503 | 0.000705 | 0.023363 | 0.001028 | 7.09E-09 |
| COMMD4 | 0.783383 | 0.102117 | 0.05933 | 0.137881 | 0.081192 | 7.12E-09 |
| GRK5 | 0.445681 | 0.230367 | 0.169144 | 0.285565 | 0.205036 | 7.12E-09 |
| CD8A | 0.347431 | 0.470521 | 0.369821 | 0.38277 | 0.29445 | 7.37E-09 |
| APBA2 | 0.382205 | 0.279084 | 0.214131 | 0.346266 | 0.262076 | 8.36E-09 |
| MED16 | 0.555679 | 0.196252 | 0.133517 | 0.242595 | 0.17112 | 8.79E-09 |
| SAMSN1 | 0.572027 | 0.155552 | 0.104635 | 0.201293 | 0.133607 | 9.54E-09 |
| DCXR | 0.461745 | 0.234969 | 0.170613 | 0.293283 | 0.2148 | 1E-08 |
| H1-3 | 0.370809 | 0.376846 | 0.291433 | 0.402378 | 0.31963 | 1.14E-08 |
| IL7R | 1.115453 | 0.059237 | 0.027341 | 0.075928 | 0.03443 | 1.54E-08 |
| SBF1 | 0.408022 | 0.276564 | 0.208434 | 0.344389 | 0.261562 | 1.58E-08 |
| CTU2 | 0.611146 | 0.116902 | 0.076533 | 0.158114 | 0.097122 | 1.6E-08 |
| LIPH | 65535 | 0.015041 | 0 | 0.020025 | 0 | 1.72E-08 |
| TIMP1 | 0.539272 | 0.13698 | 0.094258 | 0.170421 | 0.106372 | 1.82E-08 |
| PGGHG | 0.367307 | 0.38116 | 0.295486 | 0.420734 | 0.341727 | 1.89E-08 |
| CLEC12A | 1.104935 | 0.055432 | 0.025772 | 0.081352 | 0.039568 | 3.4E-08 |
| PTPRE | 0.314719 | 0.446768 | 0.359205 | 0.492491 | 0.4111 | 3.7E-08 |
| CNN2 | 0.25889 | 0.550207 | 0.459825 | 0.539841 | 0.443474 | 3.94E-08 |
| CLEC2D | 0.25952 | 0.569081 | 0.475391 | 0.58302 | 0.501542 | 4.31E-08 |
| FBXW7 | 0.229732 | 0.667602 | 0.569327 | 0.659783 | 0.578623 | 4.37E-08 |
| TRAF1 | 0.682672 | 0.100143 | 0.06239 | 0.129954 | 0.076053 | 4.54E-08 |
| R3HDM4 | 0.317779 | 0.386764 | 0.310302 | 0.448269 | 0.364337 | 6.03E-08 |
| UBALD2 | 0.334992 | 0.356535 | 0.282657 | 0.42136 | 0.340699 | 7.93E-08 |
| NOP58 | 0.41934 | 0.209574 | 0.156713 | 0.27743 | 0.204008 | 8.06E-08 |
| OXNAD1 | 0.654438 | 0.124305 | 0.078974 | 0.158323 | 0.101747 | 8.51E-08 |
| CNTRL | 0.324641 | 0.392346 | 0.313287 | 0.43033 | 0.35149 | 8.79E-08 |
| GIMAP5 | 0.383987 | 0.247557 | 0.189707 | 0.308719 | 0.228674 | 9.8E-08 |
| RMDN3 | 0.338311 | 0.309429 | 0.244748 | 0.375261 | 0.295478 | 1.03E-07 |
| KLRC1 | 0.348057 | 0.331821 | 0.260692 | 0.350438 | 0.270298 | 1.05E-07 |
| CREM | 0.378516 | 0.343941 | 0.264569 | 0.386942 | 0.313977 | 1.12E-07 |
| PTGDS | 0.533737 | 0.429625 | 0.296769 | 0.234251 | 0.169579 | 1.33E-07 |
| RBM5 | 0.459452 | 0.22761 | 0.165533 | 0.283062 | 0.213772 | 1.34E-07 |
| IRF2BPL | 0.368073 | 0.325864 | 0.252485 | 0.384439 | 0.309353 | 1.64E-07 |
| MGAT4A | 0.235272 | 0.589325 | 0.500646 | 0.595119 | 0.514902 | 1.75E-07 |
| ZMYND8 | 0.359602 | 0.281314 | 0.21925 | 0.346475 | 0.268756 | 2.03E-07 |
| CASZ1 | 0.525225 | 0.133024 | 0.092432 | 0.179808 | 0.119733 | 2.1E-07 |
| COL5A3 | 2.674488 | 0.023442 | 0.003672 | 0.025866 | 0.004111 | 2.3E-07 |
| ID2 | 0.181393 | 0.866415 | 0.76405 | 0.722987 | 0.667009 | 2.69E-07 |
| SLC7A5 | 0.5605 | 0.153168 | 0.103858 | 0.196496 | 0.137205 | 3.57E-07 |
| OBSCN | 0.507788 | 0.144075 | 0.101328 | 0.168127 | 0.110483 | 3.86E-07 |
| ZBTB1 | 0.225298 | 0.631911 | 0.540549 | 0.627034 | 0.559609 | 4.34E-07 |
| SPCS1 | 0.326729 | 0.321036 | 0.255976 | 0.392991 | 0.313977 | 4.35E-07 |
| CCM2 | 0.28643 | 0.385141 | 0.315788 | 0.456404 | 0.376156 | 5.1E-07 |
| ABCA7 | 0.475311 | 0.147009 | 0.105745 | 0.19587 | 0.135663 | 5.81E-07 |
| AP1G2 | 0.407015 | 0.240922 | 0.181699 | 0.296412 | 0.229188 | 6.19E-07 |
| RAB5IF | 0.343111 | 0.291248 | 0.229602 | 0.367543 | 0.298561 | 6.4E-07 |
| JAK3 | 0.280655 | 0.416749 | 0.343075 | 0.47622 | 0.399794 | 6.75E-07 |
| DHRS3 | 0.263475 | 0.502255 | 0.418418 | 0.537547 | 0.459404 | 6.81E-07 |
| TSPYL2 | 0.477143 | 0.230117 | 0.165316 | 0.255945 | 0.193731 | 7.44E-07 |
| GPCPD1 | 0.293014 | 0.376664 | 0.307432 | 0.439925 | 0.362795 | 7.98E-07 |
| FAM118A | 0.590844 | 0.160094 | 0.106295 | 0.187526 | 0.13258 | 8.28E-07 |
| TTC13 | 0.479636 | 0.17669 | 0.126714 | 0.234043 | 0.172662 | 8.4E-07 |
| GIMAP7 | 0.171268 | 0.884563 | 0.785547 | 0.737589 | 0.678314 | 1.01E-06 |
| MLKL | 0.524919 | 0.151456 | 0.105262 | 0.195661 | 0.138232 | 1.05E-06 |
| CRAMP1 | 0.372322 | 0.262297 | 0.202634 | 0.319566 | 0.248715 | 1.05E-06 |
| TYROBP | 0.214162 | 0.637108 | 0.549217 | 0.639341 | 0.568859 | 1.11E-06 |
| NAMPT | 0.491196 | 0.114351 | 0.081353 | 0.168127 | 0.112025 | 1.19E-06 |
| TSSC4 | 0.544217 | 0.138191 | 0.094766 | 0.18398 | 0.128983 | 1.33E-06 |
| USP36 | 0.414767 | 0.181631 | 0.136249 | 0.243846 | 0.179342 | 1.4E-06 |
| ACAP1 | 0.187499 | 0.715032 | 0.627889 | 0.676888 | 0.604317 | 1.57E-06 |
| IKZF2 | 0.270529 | 0.446314 | 0.370001 | 0.484147 | 0.406475 | 1.61E-06 |
| STXBP2 | 0.23045 | 0.51091 | 0.435484 | 0.545682 | 0.464543 | 1.72E-06 |
| EGLN2 | 0.323325 | 0.278911 | 0.222913 | 0.351898 | 0.278006 | 1.91E-06 |
| S1PR5 | 0.168521 | 0.868115 | 0.772409 | 0.747184 | 0.678828 | 2.13E-06 |
| SRA1 | 0.37669 | 0.218411 | 0.168221 | 0.280559 | 0.213258 | 2.36E-06 |
| ITK | 0.219054 | 0.518688 | 0.44562 | 0.557989 | 0.475848 | 2.39E-06 |
| H1-10 | 0.270597 | 0.606289 | 0.502599 | 0.489153 | 0.422405 | 2.48E-06 |
| MSL2 | 0.177184 | 0.745103 | 0.65899 | 0.692324 | 0.635149 | 2.64E-06 |
| GMIP | 0.241156 | 0.492771 | 0.416918 | 0.532123 | 0.460432 | 3.44E-06 |
| IFI16 | 0.165451 | 0.835451 | 0.74493 | 0.745307 | 0.672662 | 3.46E-06 |
| PDE7A | 0.285554 | 0.301466 | 0.24733 | 0.367126 | 0.288284 | 3.47E-06 |
| GRK6 | 0.250267 | 0.465142 | 0.391064 | 0.519399 | 0.437307 | 3.47E-06 |
| GLYCTK | 0.369173 | 0.248318 | 0.192254 | 0.30121 | 0.233299 | 3.63E-06 |
| ZRANB2 | 0.322725 | 0.273463 | 0.21865 | 0.336254 | 0.264645 | 3.67E-06 |
| MICAL1 | 0.344771 | 0.303054 | 0.238635 | 0.356279 | 0.288284 | 3.75E-06 |
| RBCK1 | 0.413031 | 0.206579 | 0.15515 | 0.26262 | 0.199383 | 3.83E-06 |
| BTN3A1 | 0.303715 | 0.335541 | 0.271843 | 0.396955 | 0.324255 | 3.83E-06 |
| TPST2 | 0.198275 | 0.656088 | 0.571841 | 0.653525 | 0.584275 | 4.05E-06 |
| PRR5L | 0.184897 | 0.698465 | 0.614448 | 0.660826 | 0.594553 | 4.24E-06 |
| KAT6A | 0.273197 | 0.379399 | 0.313947 | 0.451398 | 0.378212 | 5.2E-06 |
| PHF21A | 0.354952 | 0.262855 | 0.205526 | 0.32791 | 0.260534 | 5.54E-06 |
| SCAND1 | 0.307117 | 0.289384 | 0.233896 | 0.357739 | 0.285714 | 5.57E-06 |
| FBXO33 | 0.424981 | 0.173505 | 0.129235 | 0.236337 | 0.176259 | 5.69E-06 |
| BCL3 | 0.443917 | 0.162411 | 0.119394 | 0.205257 | 0.150051 | 5.98E-06 |
| CREBRF | 0.276361 | 0.37564 | 0.310155 | 0.443888 | 0.374101 | 5.98E-06 |
| SLA2 | 0.240181 | 0.472338 | 0.3999 | 0.510638 | 0.440391 | 6.03E-06 |
| TAGLN2 | 0.344083 | 0.256444 | 0.202029 | 0.323321 | 0.260534 | 6.33E-06 |
| JOSD1 | 0.280632 | 0.341939 | 0.281494 | 0.407176 | 0.335046 | 6.33E-06 |
| TMEM175 | 0.356657 | 0.205442 | 0.160444 | 0.260117 | 0.195786 | 6.36E-06 |
| BTN2A2 | 1.155252 | 0.035647 | 0.016005 | 0.048811 | 0.021069 | 6.81E-06 |
| LRRC37A2 | 2.952563 | 0.013238 | 0.00171 | 0.019191 | 0.002569 | 6.94E-06 |
| RBM6 | 0.278923 | 0.371081 | 0.305848 | 0.426366 | 0.353546 | 7.75E-06 |
| RHOB | 0.300973 | 0.15251 | 0.123794 | 0.186692 | 0.129496 | 8.82E-06 |
| RGS2 | 0.223559 | 0.476633 | 0.408212 | 0.458907 | 0.381809 | 9.23E-06 |
| KLF9 | 0.215709 | 0.588553 | 0.506817 | 0.583646 | 0.51593 | 9.4E-06 |
| LAT | 0.2248 | 0.465274 | 0.398141 | 0.51627 | 0.448613 | 1.02E-05 |
| GK5 | 0.269011 | 0.340124 | 0.282265 | 0.398206 | 0.327338 | 1.09E-05 |
| GNB2 | 0.242198 | 0.407826 | 0.344799 | 0.470588 | 0.405447 | 1.38E-05 |
| GFOD1 | 0.216111 | 0.534461 | 0.460109 | 0.555903 | 0.49075 | 1.38E-05 |
| GIMAP6 | 0.2177 | 0.52383 | 0.45046 | 0.542136 | 0.467626 | 1.52E-05 |
| HAVCR2 | 0.200194 | 0.640353 | 0.557385 | 0.608469 | 0.539054 | 1.92E-05 |
| ANKRD44 | 0.217611 | 0.531002 | 0.456655 | 0.572174 | 0.498972 | 1.95E-05 |
| PTPN4 | 0.143192 | 0.880688 | 0.797475 | 0.764497 | 0.711716 | 2.17E-05 |
| BNC2 | 0.392309 | 0.174392 | 0.132871 | 0.222778 | 0.165468 | 2.25E-05 |
| KLRC4 | 0.26676 | 0.298412 | 0.248035 | 0.342303 | 0.270812 | 2.3E-05 |
| GCHFR | 0.401299 | 0.166862 | 0.126344 | 0.195661 | 0.141829 | 2.42E-05 |
| MICA | 1.09537 | 0.040814 | 0.019102 | 0.055903 | 0.027749 | 2.55E-05 |
| DYNLT1 | 0.295458 | 0.284546 | 0.231852 | 0.352524 | 0.288798 | 3.14E-05 |
| CDK5R1 | 0.416434 | 0.168022 | 0.125895 | 0.216312 | 0.162384 | 3.14E-05 |
| GGNBP2 | 0.255127 | 0.399411 | 0.334672 | 0.457655 | 0.397225 | 3.14E-05 |
| TET2 | 0.399472 | 0.166647 | 0.126341 | 0.218607 | 0.16444 | 3.19E-05 |
| NFKBIZ | 0.303461 | 0.210901 | 0.170894 | 0.259908 | 0.199383 | 3.52E-05 |
| RASSF4 | 0.370242 | 0.190116 | 0.147084 | 0.228828 | 0.172662 | 3.88E-05 |
| SPSB3 | 0.276639 | 0.316959 | 0.261654 | 0.378598 | 0.315519 | 4E-05 |
| GIMAP8 | 0.398992 | 0.180289 | 0.136729 | 0.225699 | 0.174203 | 4.1E-05 |
| RNH1 | 0.257355 | 0.361548 | 0.302478 | 0.422612 | 0.356629 | 4.12E-05 |
| ARRDC3 | 0.240142 | 0.350596 | 0.296836 | 0.392365 | 0.321686 | 4.16E-05 |
| HTT | 0.25692 | 0.311246 | 0.260474 | 0.382562 | 0.313977 | 4.79E-05 |
| CH25H | 2.010157 | 0.014425 | 0.003581 | 0.020859 | 0.004625 | 4.8E-05 |
| RPTOR | 0.406703 | 0.162393 | 0.122501 | 0.217355 | 0.162898 | 4.8E-05 |
| CREBZF | 0.359201 | 0.229969 | 0.179283 | 0.279933 | 0.224049 | 5.47E-05 |
| AUTS2 | 0.239575 | 0.378405 | 0.320507 | 0.431164 | 0.363823 | 5.71E-05 |
| SLAMF6 | 0.165897 | 0.726786 | 0.647838 | 0.687317 | 0.622816 | 5.87E-05 |
| PUM1 | 0.213528 | 0.414056 | 0.357093 | 0.48519 | 0.410586 | 6.28E-05 |
| IRF4 | 1.035756 | 0.027895 | 0.013606 | 0.039841 | 0.016958 | 6.4E-05 |
| GPR141 | 0.347014 | 0.143861 | 0.113105 | 0.182728 | 0.131552 | 6.75E-05 |
| ABHD17B | 0.283784 | 0.283931 | 0.233231 | 0.347101 | 0.284687 | 7.04E-05 |
| PLTP | 1.896453 | 0.017694 | 0.004753 | 0.024614 | 0.007194 | 7.47E-05 |
| MYSM1 | 0.31507 | 0.264401 | 0.212529 | 0.315811 | 0.256423 | 7.7E-05 |
| IRS2 | 0.191542 | 0.583215 | 0.510703 | 0.553817 | 0.490236 | 8.56E-05 |
| PCNX4 | 0.361678 | 0.171135 | 0.133187 | 0.213809 | 0.160843 | 8.68E-05 |
| TESPA1 | 0.345598 | 0.172584 | 0.13582 | 0.225699 | 0.172662 | 8.69E-05 |
| PLEKHO1 | 0.343733 | 0.197331 | 0.155497 | 0.249896 | 0.1963 | 8.79E-05 |
| CD93 | 0.318765 | 0.20871 | 0.167334 | 0.267835 | 0.209661 | 8.79E-05 |
| PDZD8 | 0.324633 | 0.251922 | 0.201161 | 0.316854 | 0.259507 | 8.93E-05 |
| SH3BP2 | 0.208687 | 0.443527 | 0.383795 | 0.505215 | 0.43371 | 9.65E-05 |
| DDTL | 1.037513 | 0.025055 | 0.012206 | 0.034835 | 0.013875 | 9.96E-05 |
| CSNK1A1 | 0.379918 | 0.147369 | 0.11325 | 0.206925 | 0.155704 | 0.000109 |
| LCK | 0.16818 | 0.636636 | 0.566583 | 0.637046 | 0.569373 | 0.000113 |
| MAP2K3 | 0.242191 | 0.383797 | 0.324485 | 0.447226 | 0.383864 | 0.000126 |
| KIR2DL4 | 1.3885 | 0.026521 | 0.01013 | 0.035461 | 0.014902 | 0.000138 |
| RASSF1 | 0.182263 | 0.588953 | 0.519055 | 0.601377 | 0.543679 | 0.000158 |
| RASGEF1B | 0.271099 | 0.263509 | 0.218367 | 0.314768 | 0.256423 | 0.000161 |
| MUC20 | 1.035692 | 0.035409 | 0.017272 | 0.045474 | 0.022097 | 0.000165 |
| EPB41 | 0.21488 | 0.502792 | 0.433214 | 0.543805 | 0.482528 | 0.00018 |
| CHD2 | 0.151054 | 0.718788 | 0.647335 | 0.675845 | 0.617163 | 0.00018 |
| GTPBP6 | 0.308783 | 0.258805 | 0.208939 | 0.31748 | 0.260534 | 0.000184 |
| TTLL3 | 0.276993 | 0.242501 | 0.200139 | 0.299541 | 0.241521 | 0.000185 |
| ORAI3 | 1.168842 | 0.038408 | 0.017083 | 0.049854 | 0.025694 | 0.000187 |
| MED13L | 0.262396 | 0.251798 | 0.209924 | 0.326658 | 0.264132 | 0.000187 |
| TNIK | 0.217649 | 0.434936 | 0.37403 | 0.483521 | 0.421377 | 0.00019 |
| PDXK | 0.267187 | 0.27192 | 0.225949 | 0.327076 | 0.268756 | 0.000205 |
| ARAP1 | 0.224317 | 0.412291 | 0.352921 | 0.475177 | 0.406475 | 0.00021 |
| MT1X | 1.027918 | 0.030078 | 0.014751 | 0.044431 | 0.021583 | 0.000212 |
| GSAP | 0.196323 | 0.519946 | 0.453794 | 0.541302 | 0.475334 | 0.000216 |
| LPIN2 | 0.338359 | 0.182252 | 0.144151 | 0.236754 | 0.186023 | 0.000217 |
| DOCK2 | 0.171222 | 0.592676 | 0.52635 | 0.617438 | 0.564234 | 0.000223 |
| NR4A2 | 0.130899 | 1.019568 | 0.931133 | 0.733834 | 0.67112 | 0.000237 |
| ATF7IP | 0.202797 | 0.426121 | 0.370241 | 0.487276 | 0.42446 | 0.000255 |
| DUSP8 | 1.468857 | 0.021391 | 0.007728 | 0.028577 | 0.010791 | 0.000262 |
| TBC1D1 | 0.338418 | 0.196911 | 0.155739 | 0.254693 | 0.20298 | 0.000266 |
| KDM3A | 0.280632 | 0.272315 | 0.224178 | 0.330413 | 0.273381 | 0.000266 |
| ITCH | 0.145022 | 0.687875 | 0.622091 | 0.675428 | 0.615622 | 0.000299 |
| FKBP8 | 0.313588 | 0.209065 | 0.168222 | 0.270547 | 0.217369 | 0.000309 |
| P2RY11 | 0.24276 | 0.330541 | 0.279349 | 0.371089 | 0.31038 | 0.000318 |
| BPTF | 0.207037 | 0.455224 | 0.394367 | 0.511264 | 0.44296 | 0.000327 |
| DDX39A | 0.281108 | 0.278252 | 0.22899 | 0.340634 | 0.285714 | 0.000328 |
| ITPRIP | 0.175563 | 0.614918 | 0.544462 | 0.599708 | 0.536485 | 0.000342 |
| HSD17B11 | 0.228952 | 0.368406 | 0.314344 | 0.427201 | 0.365879 | 0.000381 |
| TNFSF8 | 1.519265 | 0.016626 | 0.0058 | 0.023363 | 0.007708 | 0.0004 |
| STX8 | 0.258128 | 0.309257 | 0.258592 | 0.375887 | 0.316033 | 0.00041 |
| UBR4 | 0.250208 | 0.317617 | 0.267044 | 0.389445 | 0.329394 | 0.000472 |
| ROBO3 | 1.035904 | 0.020673 | 0.010083 | 0.030246 | 0.012333 | 0.000481 |
| HNRNPL | 0.181898 | 0.522524 | 0.460627 | 0.586775 | 0.524666 | 0.000488 |
| LAX1 | 0.286144 | 0.202976 | 0.166459 | 0.257614 | 0.204522 | 0.000495 |
| THUMPD1 | 0.223143 | 0.344603 | 0.29522 | 0.409679 | 0.350976 | 0.000495 |
| EXOC4 | 0.319116 | 0.235403 | 0.18869 | 0.295161 | 0.244604 | 0.000498 |
| LAMTOR4 | 0.186365 | 0.515087 | 0.452668 | 0.541093 | 0.486125 | 0.0005 |
| PRKCQ | 0.215983 | 0.299385 | 0.257758 | 0.37234 | 0.31038 | 0.000503 |
| GLRX | 0.18959 | 0.419868 | 0.368163 | 0.472466 | 0.405447 | 0.000514 |
| FAM78A | 0.220652 | 0.37513 | 0.321929 | 0.440134 | 0.376156 | 0.000529 |
| ARAP2 | 0.243278 | 0.295156 | 0.249355 | 0.360868 | 0.301644 | 0.000534 |
| CYTL1 | 2.434081 | 0.008984 | 0.001662 | 0.012307 | 0.001542 | 0.000556 |
| YES1 | 0.260066 | 0.23771 | 0.198499 | 0.30121 | 0.24666 | 0.000562 |
| PPP2R5C | 0.158807 | 0.615693 | 0.551517 | 0.642261 | 0.583762 | 0.000575 |
| LSM7 | 0.248343 | 0.227464 | 0.191493 | 0.30121 | 0.246146 | 0.000637 |
| MTCH1 | 0.270239 | 0.282441 | 0.234195 | 0.346266 | 0.293422 | 0.000638 |
| NCAM1 | 0.239654 | 0.244505 | 0.207083 | 0.289111 | 0.233299 | 0.000641 |
| CSNK1D | 0.187184 | 0.49328 | 0.433257 | 0.544848 | 0.486125 | 0.000698 |
| MKLN1 | 0.286433 | 0.222707 | 0.182603 | 0.285148 | 0.233813 | 0.000738 |
| TNNT3 | 1.054656 | 0.02009 | 0.009672 | 0.028786 | 0.011819 | 0.000754 |
| LUC7L | 0.259043 | 0.283941 | 0.237273 | 0.342094 | 0.288798 | 0.000773 |
| DYM | 0.212769 | 0.327657 | 0.282728 | 0.3932 | 0.335046 | 0.000776 |
| CRKL | 0.218851 | 0.299754 | 0.257564 | 0.377764 | 0.317575 | 0.000783 |
| CCNL2 | 0.240871 | 0.31848 | 0.269509 | 0.375469 | 0.31963 | 0.00079 |
| NFE2L2 | 0.230316 | 0.322967 | 0.275313 | 0.377972 | 0.320658 | 0.000812 |
| LGALS1 | 0.198778 | 0.521971 | 0.454787 | 0.446809 | 0.387461 | 0.00082 |
| VPS53 | 0.29561 | 0.240925 | 0.196288 | 0.300584 | 0.249743 | 0.000866 |
| JMJD1C | 0.229916 | 0.322687 | 0.27515 | 0.390488 | 0.332477 | 0.000866 |
| ERRFI1 | 1.757389 | 0.015197 | 0.004495 | 0.019816 | 0.006166 | 0.000888 |
| HBP1 | 0.195694 | 0.410176 | 0.358146 | 0.475177 | 0.411614 | 0.000932 |
| FOXP1 | 0.179165 | 0.462931 | 0.408867 | 0.518773 | 0.458376 | 0.001012 |
| UTRN | 0.141974 | 0.685068 | 0.620862 | 0.676888 | 0.62333 | 0.001041 |
| NARF | 0.285266 | 0.236537 | 0.1941 | 0.298707 | 0.247688 | 0.001051 |
| S1PR4 | 0.213628 | 0.303234 | 0.261499 | 0.369837 | 0.31295 | 0.001057 |
| FXYD7 | 3.700833 | 0.007859 | 0.000604 | 0.009387 | 0.000514 | 0.001102 |
| ENO1 | 0.210165 | 0.337234 | 0.291518 | 0.419065 | 0.363309 | 0.001102 |
| TRIM38 | 0.187054 | 0.335793 | 0.29496 | 0.393617 | 0.331449 | 0.001117 |
| FBXW4 | 0.230519 | 0.276583 | 0.235739 | 0.341469 | 0.289825 | 0.001206 |
| H3-3B | 0.236445 | 0.274328 | 0.232859 | 0.349812 | 0.29702 | 0.001258 |
| NKTR | 0.274377 | 0.26887 | 0.222304 | 0.328536 | 0.27852 | 0.001295 |
| TMEM259 | 0.23646 | 0.343407 | 0.291492 | 0.406341 | 0.356115 | 0.00136 |
| BTN3A3 | 0.231609 | 0.298962 | 0.254622 | 0.368586 | 0.314491 | 0.00147 |
| YPEL3 | 0.164462 | 0.561127 | 0.500671 | 0.575511 | 0.513875 | 0.001515 |
| GALNT10 | 0.237214 | 0.268293 | 0.227615 | 0.331039 | 0.27852 | 0.001652 |
| TXK | 0.182801 | 0.458108 | 0.403589 | 0.499374 | 0.446043 | 0.001677 |
| ARRDC2 | 0.219659 | 0.303688 | 0.260798 | 0.364414 | 0.311408 | 0.00174 |
| CBLB | 0.160519 | 0.56599 | 0.506393 | 0.578223 | 0.521069 | 0.001816 |
| RAP1GAP2 | 0.145396 | 0.619453 | 0.560066 | 0.615561 | 0.557554 | 0.001818 |
| GUK1 | 0.224394 | 0.303243 | 0.259562 | 0.377555 | 0.321172 | 0.001902 |
| SLC12A8 | 2.56034 | 0.007923 | 0.001343 | 0.010847 | 0.001542 | 0.001976 |
| TOMM7 | 0.147742 | 0.637132 | 0.575115 | 0.647267 | 0.576053 | 0.002004 |
| ARHGAP26 | 0.202787 | 0.338713 | 0.294298 | 0.405924 | 0.350976 | 0.002135 |
| CASP8 | 0.139847 | 0.703499 | 0.638507 | 0.672716 | 0.621788 | 0.002195 |
| TUBGCP6 | 0.210685 | 0.338286 | 0.292322 | 0.400083 | 0.344296 | 0.002231 |
| PRSS21 | 1.692818 | 0.016194 | 0.005009 | 0.020859 | 0.007708 | 0.002425 |
| CDK5RAP3 | 0.190442 | 0.393951 | 0.345234 | 0.444097 | 0.388489 | 0.002689 |
| IKBKB | 0.167207 | 0.529742 | 0.471769 | 0.548811 | 0.494347 | 0.00271 |
| TBC1D9B | 0.152066 | 0.490622 | 0.44154 | 0.517939 | 0.45889 | 0.002715 |
| TRIM26 | 0.246034 | 0.255733 | 0.215637 | 0.321443 | 0.270298 | 0.002719 |
| DHX36 | 0.20992 | 0.315301 | 0.272605 | 0.382144 | 0.327338 | 0.00277 |
| UAP1 | 0.188616 | 0.322095 | 0.282621 | 0.391114 | 0.334532 | 0.003229 |
| STAT3 | 0.167162 | 0.423806 | 0.377439 | 0.488736 | 0.430627 | 0.003233 |
| MEF2D | 0.18482 | 0.392646 | 0.345434 | 0.458907 | 0.406989 | 0.003602 |
| OPHN1 | 2.578314 | 0.008453 | 0.001415 | 0.011055 | 0.002055 | 0.003675 |
| ARPC1B | 0.185464 | 0.414459 | 0.364461 | 0.477263 | 0.424974 | 0.003675 |
| SH2D1B | 0.111348 | 0.892175 | 0.825907 | 0.766792 | 0.70298 | 0.003773 |
| GOLGA6L6 | 2.808365 | 0.00718 | 0.001025 | 0.010013 | 0.001542 | 0.003922 |
| CHURC1 | 0.197133 | 0.394801 | 0.344378 | 0.453484 | 0.398767 | 0.003968 |
| B4GALT1 | 0.136015 | 0.662769 | 0.603139 | 0.643513 | 0.589928 | 0.003981 |
| GBP2 | 0.166723 | 0.408811 | 0.364195 | 0.463079 | 0.403392 | 0.004053 |
| ERN1 | 0.218715 | 0.328127 | 0.28197 | 0.378181 | 0.325797 | 0.004354 |
| TYK2 | 0.182018 | 0.39225 | 0.345757 | 0.452441 | 0.401336 | 0.0045 |
| CMKLR1 | 0.185751 | 0.309828 | 0.272398 | 0.34272 | 0.286742 | 0.004534 |
| SLC15A4 | 0.146249 | 0.432201 | 0.390535 | 0.497914 | 0.43628 | 0.004552 |
| RASA2 | 0.173725 | 0.399084 | 0.353808 | 0.468294 | 0.411614 | 0.004557 |
| ABI3 | 0.142219 | 0.57133 | 0.517696 | 0.599082 | 0.538027 | 0.004645 |
| DNTTIP2 | 0.177082 | 0.417677 | 0.369431 | 0.468502 | 0.416752 | 0.004973 |
| ZNF655 | 0.143248 | 0.426466 | 0.386155 | 0.482687 | 0.417266 | 0.005128 |
| AKAP17A | 0.166036 | 0.494961 | 0.441153 | 0.539424 | 0.487153 | 0.005199 |
| VPS37B | 0.179614 | 0.407717 | 0.359989 | 0.468711 | 0.416238 | 0.005333 |
| CCNL1 | 0.166368 | 0.368993 | 0.328803 | 0.437005 | 0.378726 | 0.005887 |
| TRIR | 0.137507 | 0.631859 | 0.574416 | 0.641427 | 0.581192 | 0.00623 |
| DNAJC18 | 1.051168 | 0.018437 | 0.008898 | 0.026074 | 0.012333 | 0.006654 |
| DPP9 | 0.181029 | 0.326805 | 0.288266 | 0.401126 | 0.346865 | 0.00667 |
| XAF1 | 0.165767 | 0.30916 | 0.275602 | 0.36796 | 0.313977 | 0.006677 |
| KCNK6 | 1.723957 | 0.009894 | 0.002995 | 0.015436 | 0.005139 | 0.006731 |
| STAT4 | 0.146338 | 0.586505 | 0.529931 | 0.605131 | 0.550874 | 0.006885 |
| RNF149 | 0.139704 | 0.500241 | 0.454071 | 0.545056 | 0.49332 | 0.007076 |
| TATDN2 | 0.161148 | 0.38772 | 0.346743 | 0.454735 | 0.400822 | 0.007653 |
| ITPKB | 0.12455 | 0.633379 | 0.580993 | 0.636629 | 0.582734 | 0.007894 |
| PPP4R3A | 0.156295 | 0.369095 | 0.331199 | 0.444931 | 0.390031 | 0.008322 |
| CLEC2B | 0.154997 | 0.491618 | 0.441539 | 0.526491 | 0.47482 | 0.008406 |
| CHMP4B | 0.178369 | 0.286412 | 0.253103 | 0.366708 | 0.313977 | 0.008435 |
| CHIC2 | 0.175131 | 0.342525 | 0.30337 | 0.41093 | 0.358171 | 0.008459 |
| RGS1 | 0.144076 | 0.583376 | 0.527931 | 0.51335 | 0.463001 | 0.00909 |
| CLEC12B | 1.146115 | 0.014591 | 0.006593 | 0.021902 | 0.009764 | 0.009151 |
| SRSF3 | 0.155792 | 0.401826 | 0.360695 | 0.478306 | 0.427544 | 0.009453 |
| TMSB4Y | 2.592462 | 0.005594 | 0.000927 | 0.007927 | 0.001028 | 0.009579 |
| **Proliferat CD56- CD16+ NK cells** | | | |  |  |  |
| MT-ND2 | 1.803037 | 1.800266 | 0.515904 | 0.84492 | 0.313953 | 1.48E-12 |
| DDX3Y | 2.933483 | 0.5646 | 0.073905 | 0.502674 | 0.081395 | 6.11E-08 |
| CYTIP | 0.558981 | 1.706017 | 1.158013 | 0.962567 | 0.872093 | 6.11E-08 |
| THEMIS2 | 1.84464 | 0.758878 | 0.211291 | 0.636364 | 0.290698 | 1.1E-07 |
| PRDM1 | 1.488498 | 0.697506 | 0.248579 | 0.588235 | 0.290698 | 0.000136 |
| FXYD5 | 0.380112 | 1.798974 | 1.382292 | 0.957219 | 0.906977 | 0.000296 |
| UTY | 4.247872 | 0.260726 | 0.013723 | 0.294118 | 0.034884 | 0.000501 |
| CD3E | 1.024848 | 1.232938 | 0.605942 | 0.673797 | 0.5 | 0.000584 |
| EIF1AY | 2.833298 | 0.272902 | 0.038291 | 0.31016 | 0.046512 | 0.000975 |
| CXCR4 | 0.479034 | 2.434911 | 1.746946 | 0.946524 | 0.813953 | 0.001023 |
| DUSP2 | 0.802138 | 1.231013 | 0.705984 | 0.743316 | 0.651163 | 0.001211 |
| ZFP36 | 0.411845 | 2.357767 | 1.772242 | 0.973262 | 0.860465 | 0.001743 |
| TSC22D3 | 0.348791 | 2.390149 | 1.876846 | 0.962567 | 0.860465 | 0.003857 |
| PSMB9 | 0.286345 | 1.783137 | 1.462131 | 0.967914 | 0.906977 | 0.004742 |
| ITM2A | 0.998102 | 0.660641 | 0.330756 | 0.620321 | 0.348837 | 0.005519 |
| **NK T-like cells** | |  |  |  |  |  |
| MT-ND2 | 1.952303 | 1.08115 | 0.279373 | 0.783562 | 0.198413 | 2.39E-34 |
| DDX3Y | 3.393943 | 0.4542 | 0.043208 | 0.490411 | 0.039683 | 5.25E-27 |
| THEMIS2 | 1.806475 | 0.540745 | 0.154593 | 0.578082 | 0.178571 | 8.32E-19 |
| COL5A3 | 6.082223 | 0.360685 | 0.005323 | 0.284932 | 0.007937 | 5.64E-16 |
| CXCR4 | 0.357542 | 2.875075 | 2.243977 | 0.978082 | 0.920635 | 7.79E-15 |
| IL7R | 0.903816 | 1.603249 | 0.856891 | 0.775342 | 0.535714 | 1.35E-14 |
| DUSP2 | 0.618046 | 1.612061 | 1.050343 | 0.909589 | 0.734127 | 1.98E-14 |
| TSC22D3 | 0.336422 | 2.225464 | 1.762575 | 0.989041 | 0.916667 | 1.81E-10 |
| PIK3R1 | 0.581047 | 1.147031 | 0.766765 | 0.854795 | 0.702381 | 1.43E-09 |
| FOSL2 | 0.673443 | 1.026108 | 0.643379 | 0.805479 | 0.599206 | 1.86E-09 |
| UTY | 2.736751 | 0.18332 | 0.027502 | 0.243836 | 0.031746 | 1.89E-09 |
| SLC7A5 | 1.210657 | 0.564644 | 0.243967 | 0.539726 | 0.281746 | 2.26E-09 |
| KDM5D | 2.963118 | 0.174643 | 0.022396 | 0.208219 | 0.02381 | 3.07E-08 |
| EIF1AY | 3.804227 | 0.125385 | 0.008976 | 0.158904 | 0.011905 | 9.56E-07 |
| ZFP36 | 0.295667 | 2.158516 | 1.758534 | 0.958904 | 0.900794 | 1.03E-06 |
| PER1 | 0.91954 | 0.562827 | 0.297554 | 0.569863 | 0.357143 | 2.16E-06 |
| USP9Y | 2.966892 | 0.118465 | 0.015152 | 0.167123 | 0.019841 | 3.26E-06 |
| PDE4D | 0.635803 | 0.840435 | 0.540889 | 0.712329 | 0.543651 | 4.56E-06 |
| FKBP5 | 1.267562 | 0.328853 | 0.136593 | 0.372603 | 0.162698 | 8.46E-06 |
| CNOT6L | 0.445333 | 0.989204 | 0.726486 | 0.827397 | 0.694444 | 1.89E-05 |
| PFKFB3 | 1.094703 | 0.375176 | 0.17567 | 0.410959 | 0.206349 | 3.04E-05 |
| PRDM1 | 0.791287 | 0.552221 | 0.319089 | 0.545205 | 0.31746 | 5.34E-05 |
| ACTN4 | 0.571558 | 0.752146 | 0.506112 | 0.715068 | 0.531746 | 6.13E-05 |
| CD55 | 0.872268 | 0.405777 | 0.221671 | 0.471233 | 0.261905 | 0.000113 |
| IL18RAP | 0.543985 | 0.822485 | 0.564121 | 0.712329 | 0.563492 | 0.000113 |
| MAP3K8 | 0.610539 | 0.712443 | 0.466616 | 0.682192 | 0.464286 | 0.000125 |
| AKNA | 0.212248 | 1.702922 | 1.469947 | 0.967123 | 0.90873 | 0.000223 |
| PDE4B | 0.845911 | 0.412988 | 0.22977 | 0.449315 | 0.257937 | 0.000406 |
| YWHAB | 0.853818 | 0.343456 | 0.190041 | 0.438356 | 0.25 | 0.000574 |
| KLF3 | 0.38914 | 1.002895 | 0.765795 | 0.8 | 0.702381 | 0.000574 |
| SPOCK2 | 0.334682 | 1.155219 | 0.916041 | 0.854795 | 0.765873 | 0.000574 |
| CEBPD | 0.569835 | 0.853347 | 0.574895 | 0.663014 | 0.484127 | 0.00064 |
| GZMK | 0.563676 | 1.106901 | 0.748903 | 0.638356 | 0.488095 | 0.000837 |
| LPIN2 | 0.943748 | 0.309575 | 0.160942 | 0.380822 | 0.214286 | 0.000997 |
| PIK3IP1 | 0.677833 | 0.521634 | 0.326075 | 0.550685 | 0.369048 | 0.001011 |
| SATB1 | 0.517106 | 0.737031 | 0.515017 | 0.679452 | 0.527778 | 0.001011 |
| KLF13 | 0.403803 | 0.949115 | 0.717401 | 0.810959 | 0.662698 | 0.001011 |
| CXCR6 | 0.850852 | 0.419225 | 0.232442 | 0.394521 | 0.210317 | 0.001054 |
| TENT5C | 0.491035 | 0.848097 | 0.603433 | 0.690411 | 0.543651 | 0.001124 |
| OXNAD1 | 0.778441 | 0.371342 | 0.216491 | 0.438356 | 0.257937 | 0.00162 |
| TNFRSF13C | 2.602999 | 0.104991 | 0.017281 | 0.128767 | 0.027778 | 0.001676 |
| NPDC1 | 0.996559 | 0.35172 | 0.17628 | 0.361644 | 0.206349 | 0.001686 |
| CEBPB | 1.189843 | 0.192093 | 0.084204 | 0.238356 | 0.095238 | 0.001933 |
| ITM2A | 0.754804 | 0.455205 | 0.269767 | 0.460274 | 0.289683 | 0.001933 |
| ZFY | 4.251006 | 0.053922 | 0.002832 | 0.082192 | 0.003968 | 0.002023 |
| RUNX3 | 0.325976 | 1.089795 | 0.869393 | 0.830137 | 0.765873 | 0.002048 |
| NPC1 | 0.683231 | 0.471107 | 0.293391 | 0.520548 | 0.349206 | 0.002183 |
| GNLY | 0.525315 | 1.058269 | 0.735293 | 0.59726 | 0.384921 | 0.002374 |
| KLRC3 | 1.969773 | 0.106025 | 0.027067 | 0.128767 | 0.027778 | 0.002407 |
| GOLGA8B | 1.46682 | 0.122494 | 0.044316 | 0.172603 | 0.055556 | 0.002911 |
| ATP2B1 | 0.87457 | 0.291652 | 0.159072 | 0.347945 | 0.194444 | 0.003363 |
| USP36 | 0.805919 | 0.324729 | 0.185744 | 0.405479 | 0.238095 | 0.003363 |
| MGAT4A | 0.476115 | 0.705116 | 0.506915 | 0.668493 | 0.52381 | 0.004751 |
| CYTIP | 0.263547 | 1.402094 | 1.167996 | 0.950685 | 0.837302 | 0.004773 |
| ACYP2 | 0.978891 | 0.223774 | 0.113536 | 0.293151 | 0.146825 | 0.005117 |
| GCH1 | 1.006532 | 0.22483 | 0.111907 | 0.29863 | 0.154762 | 0.005533 |
| SLC4A7 | 0.860023 | 0.250439 | 0.137978 | 0.328767 | 0.170635 | 0.005533 |
| IQSEC1 | 0.442233 | 0.710695 | 0.523068 | 0.70411 | 0.547619 | 0.006259 |
| SMAP2 | 0.463173 | 0.747553 | 0.542267 | 0.69863 | 0.587302 | 0.006694 |
| CDKN1B | 0.365226 | 1.010394 | 0.784417 | 0.810959 | 0.678571 | 0.006694 |
| ENGASE | 1.482532 | 0.11458 | 0.041004 | 0.158904 | 0.051587 | 0.006876 |
| ZBTB16 | 0.280075 | 1.335821 | 1.100113 | 0.90411 | 0.793651 | 0.007442 |
| XRRA1 | 1.752759 | 0.103454 | 0.030698 | 0.136986 | 0.039683 | 0.007602 |
| RALGDS | 0.597817 | 0.470788 | 0.311075 | 0.526027 | 0.369048 | 0.007602 |
| GPR171 | 0.726635 | 0.298627 | 0.180464 | 0.372603 | 0.214286 | 0.007924 |
| HECA | 0.341966 | 0.932199 | 0.735473 | 0.813699 | 0.68254 | 0.007924 |
| P2RY8 | 0.343106 | 0.932286 | 0.734961 | 0.780822 | 0.674603 | 0.008158 |
| YBX3 | 1.71034 | 0.090864 | 0.027767 | 0.136986 | 0.039683 | 0.00818 |
| FAM43A | 0.768921 | 0.390428 | 0.229125 | 0.40274 | 0.253968 | 0.008711 |
| HPGD | 0.682637 | 0.372695 | 0.232198 | 0.413699 | 0.253968 | 0.008781 |
| CLDND1 | 0.774624 | 0.348372 | 0.203638 | 0.383562 | 0.230159 | 0.00902 |
| DDI2 | 1.587699 | 0.111813 | 0.0372 | 0.158904 | 0.055556 | 0.009292 |
| HSP90AA1 | 0.376461 | 0.733908 | 0.565347 | 0.734247 | 0.59127 | 0.009975 |

^1^log2FC - log fold-change of the average expression between the two groups (FF compared to HMF infants).

^2^FF – formula-fed.

^3^HMF - human milk fed.

^4^pct_FF, percent of cells with detected expression of the gene in FF infants.

^5^pct_HMF, percent of cells with detected expression the gene in HMF infants.

^6^p_val_adj, adjusted p-value based on Benjamini and Hochberg method using all genes in the dataset.

**Supplemental Table 5.** The list of differentially expressed genes in B cell subsets of FF (n=3) and HMF (n=6) infants.

| **Gene** | **log2FC^1^** | | **avg_FF^2^** | **avg_HMF^3^** | **pct_FF^4^** | **pct_HMF^5^** | **p_val_adj^6^** |
| --- | --- | --- | --- | --- | --- | --- | --- |
| **B cells** | |  |  |  |  |  |  |
| MT-ND2 | | 1.141057 | 1.985965 | 0.90049 | 0.914128 | 0.476061 | 3.8E-240 |
| THEMIS2 | | 1.267613 | 0.434805 | 0.180595 | 0.437275 | 0.191513 | 9.16E-70 |
| CTSH | | 0.940384 | 0.752727 | 0.392242 | 0.557688 | 0.319913 | 6.21E-65 |
| WARS1 | | 1.151316 | 0.420024 | 0.189101 | 0.413528 | 0.196409 | 1.77E-55 |
| SOCS3 | | 1.182219 | 0.367409 | 0.161907 | 0.34013 | 0.140914 | 8.21E-49 |
| TNFRSF13B | | 0.875514 | 0.647934 | 0.353163 | 0.470137 | 0.261697 | 1.34E-46 |
| EIF1AY | | 1.246273 | 0.321707 | 0.135611 | 0.30295 | 0.128945 | 6.86E-43 |
| DDX3Y | | 1.046149 | 0.414728 | 0.200836 | 0.370113 | 0.18444 | 1.1E-42 |
| KDM5D | | 1.16518 | 0.320945 | 0.143112 | 0.304629 | 0.138738 | 8.99E-39 |
| USP53 | | 1.791504 | 0.17846 | 0.051552 | 0.192612 | 0.060392 | 1.09E-36 |
| MDFIC | | 0.879705 | 0.318827 | 0.173276 | 0.365076 | 0.190968 | 1.83E-34 |
| SIGLEC14 | | 1.560544 | 0.185349 | 0.062838 | 0.195491 | 0.064744 | 2.12E-34 |
| PIEZO1 | | 0.746903 | 0.381553 | 0.22736 | 0.419765 | 0.232862 | 5.55E-34 |
| LPCAT1 | | 0.571215 | 0.655986 | 0.441511 | 0.594627 | 0.400979 | 7.36E-34 |
| IL10RA | | 0.732766 | 0.445782 | 0.268249 | 0.427441 | 0.245919 | 3.59E-33 |
| PTPN1 | | 0.345654 | 1.18094 | 0.929342 | 0.815064 | 0.688248 | 8.58E-33 |
| FXYD5 | | 0.231171 | 1.823778 | 1.553755 | 0.934517 | 0.85691 | 4.82E-31 |
| SAMSN1 | | 1.441807 | 0.137445 | 0.050594 | 0.190453 | 0.068009 | 1.06E-30 |
| ARHGAP24 | | 0.751467 | 0.460177 | 0.273345 | 0.399616 | 0.243199 | 4.27E-28 |
| TXNDC5 | | 0.549091 | 1.100032 | 0.751817 | 0.583113 | 0.411317 | 1.1E-27 |
| GPRIN3 | | 1.660544 | 0.107887 | 0.034127 | 0.163828 | 0.057671 | 5.25E-27 |
| ANXA4 | | 0.562391 | 0.724853 | 0.490855 | 0.53634 | 0.385201 | 3.27E-26 |
| SERPINB6 | | 1.553872 | 0.126906 | 0.043224 | 0.138642 | 0.040261 | 5.84E-26 |
| WEE1 | | 0.849869 | 0.287661 | 0.159604 | 0.30415 | 0.163221 | 1.28E-25 |
| YWHAB | | 0.537286 | 0.482932 | 0.332772 | 0.519309 | 0.344396 | 1.55E-24 |
| VIM | | 0.285349 | 1.706401 | 1.400176 | 0.866155 | 0.741567 | 1.59E-23 |
| DDIT4 | | 0.658921 | 0.383188 | 0.242693 | 0.361478 | 0.214363 | 2.7E-23 |
| S100A10 | | 0.558099 | 0.562364 | 0.381955 | 0.444951 | 0.287813 | 1.56E-22 |
| PSMB9 | | 0.33476 | 0.975392 | 0.773404 | 0.745742 | 0.614255 | 5.17E-22 |
| HSPA6 | | 1.221265 | 0.171205 | 0.073431 | 0.17774 | 0.077258 | 1.97E-21 |
| CPNE5 | | 0.401131 | 0.814371 | 0.616694 | 0.651235 | 0.497824 | 1.97E-21 |
| ETV6 | | 0.503961 | 0.469138 | 0.330821 | 0.48189 | 0.323177 | 3E-21 |
| PCCA | | 0.952684 | 0.150158 | 0.077582 | 0.206764 | 0.097388 | 3.09E-21 |
| PELI1 | | 0.507544 | 0.452196 | 0.318083 | 0.455025 | 0.301415 | 1.02E-20 |
| GPM6A | | 1.480161 | 0.120137 | 0.043063 | 0.127609 | 0.042982 | 1.08E-20 |
| MYC | | 0.470512 | 0.725498 | 0.523598 | 0.549292 | 0.399891 | 1.2E-20 |
| EHD3 | | 0.637709 | 0.334869 | 0.215231 | 0.355721 | 0.218172 | 1.36E-20 |
| VOPP1 | | 0.58951 | 0.381056 | 0.253238 | 0.391701 | 0.24864 | 2.26E-20 |
| FLNB | | 0.865564 | 0.219696 | 0.120576 | 0.23243 | 0.118607 | 2.7E-20 |
| SEL1L3 | | 0.383128 | 0.913979 | 0.700815 | 0.646918 | 0.491839 | 3.01E-20 |
| MYO1F | | 0.627176 | 0.239794 | 0.155253 | 0.307988 | 0.177911 | 4.05E-20 |
| BACE2 | | 1.167592 | 0.15521 | 0.069094 | 0.176541 | 0.079978 | 5.06E-20 |
| IL7 | | 0.892118 | 0.218345 | 0.117649 | 0.2142 | 0.10555 | 8.65E-20 |
| LAX1 | | 0.88331 | 0.192437 | 0.104324 | 0.230271 | 0.121328 | 2.16E-19 |
| PYHIN1 | | 0.791785 | 0.262951 | 0.151888 | 0.248021 | 0.133297 | 2.47E-19 |
| CXCR3 | | 1.481098 | 0.094082 | 0.033702 | 0.12425 | 0.04407 | 2.73E-19 |
| BHLHE41 | | 1.134979 | 0.116224 | 0.052922 | 0.15999 | 0.068553 | 3.24E-19 |
| ADGRE5 | | 0.386977 | 0.708247 | 0.541618 | 0.593188 | 0.429271 | 4E-19 |
| CRACR2B | | 1.746886 | 0.057834 | 0.017231 | 0.090429 | 0.022307 | 4.11E-19 |
| DUS2 | | 0.519225 | 0.465911 | 0.325088 | 0.44591 | 0.311208 | 4.11E-19 |
| CKAP4 | | 0.809275 | 0.235073 | 0.134149 | 0.233149 | 0.121872 | 4.28E-19 |
| LDLRAP1 | | 0.681759 | 0.225284 | 0.140443 | 0.275126 | 0.155604 | 8.71E-19 |
| CD99 | | 0.459409 | 0.550089 | 0.400071 | 0.502998 | 0.353645 | 1.74E-18 |
| LRRK2 | | 0.489459 | 0.565185 | 0.402577 | 0.471336 | 0.331338 | 1.97E-18 |
| S100A4 | | 0.487012 | 0.399493 | 0.285039 | 0.397697 | 0.25136 | 1.97E-18 |
| NT5E | | 0.864795 | 0.22448 | 0.123267 | 0.202926 | 0.100109 | 2.05E-18 |
| EPB41L4A | | 2.530753 | 0.058399 | 0.010106 | 0.07172 | 0.013058 | 2.33E-18 |
| ATP5PO | | 0.828249 | 0.117698 | 0.066289 | 0.179899 | 0.082155 | 2.37E-18 |
| RASA3 | | 0.434917 | 0.484568 | 0.358453 | 0.491005 | 0.335691 | 2.54E-18 |
| C16orf87 | | 0.618379 | 0.227409 | 0.148135 | 0.290237 | 0.168118 | 3.09E-18 |
| ERO1A | | 0.500875 | 0.410812 | 0.290312 | 0.427441 | 0.282916 | 4.14E-18 |
| GRN | | 0.432112 | 0.542576 | 0.402144 | 0.512593 | 0.364527 | 5E-18 |
| TNFRSF1B | | 0.600875 | 0.240029 | 0.158264 | 0.289998 | 0.167029 | 5.76E-18 |
| BCL2L11 | | 0.451926 | 0.609481 | 0.445571 | 0.525066 | 0.387378 | 5.9E-18 |
| UTY | | 0.790189 | 0.238052 | 0.137658 | 0.242504 | 0.136561 | 8.48E-18 |
| SCRN1 | | 0.75364 | 0.237111 | 0.140632 | 0.243943 | 0.136561 | 1.72E-17 |
| ZBTB32 | | 1.352074 | 0.104726 | 0.041024 | 0.12569 | 0.048422 | 1.85E-17 |
| CFLAR | | 0.363194 | 0.737933 | 0.5737 | 0.626529 | 0.479325 | 2.44E-17 |
| GSN | | 0.887709 | 0.180396 | 0.097499 | 0.181338 | 0.087595 | 2.46E-17 |
| FABP5 | | 0.69829 | 0.162455 | 0.100121 | 0.221636 | 0.116431 | 2.93E-17 |
| GATM | | 2.270363 | 0.057803 | 0.011981 | 0.072919 | 0.015234 | 3E-17 |
| PIP5K1C | | 0.614877 | 0.283579 | 0.185173 | 0.318542 | 0.197497 | 3.07E-17 |
| XRRA1 | | 1.042234 | 0.114522 | 0.055609 | 0.152794 | 0.068553 | 8.56E-17 |
| IGHG1 | | 0.487675 | 0.419633 | 0.299271 | 0.223075 | 0.119695 | 9.42E-17 |
| GEN1 | | 0.691032 | 0.255166 | 0.158052 | 0.273207 | 0.163221 | 1.1E-16 |
| TAGLN2 | | 0.342212 | 0.783562 | 0.618098 | 0.648597 | 0.512514 | 1.62E-16 |
| TLE3 | | 0.565809 | 0.251633 | 0.169997 | 0.307988 | 0.189336 | 2.67E-16 |
| NLRC5 | | 0.394499 | 0.648016 | 0.49298 | 0.544495 | 0.406964 | 2.67E-16 |
| PAPSS1 | | 0.547139 | 0.240664 | 0.164705 | 0.300552 | 0.181719 | 3.83E-16 |
| KBTBD8 | | 0.477193 | 0.38676 | 0.277838 | 0.393859 | 0.26333 | 4.49E-16 |
| ASPH | | 1.420044 | 0.074338 | 0.02778 | 0.098345 | 0.032644 | 5.16E-16 |
| ENC1 | | 0.889729 | 0.161825 | 0.087339 | 0.171744 | 0.083787 | 5.47E-16 |
| GRK5 | | 0.478691 | 0.299076 | 0.214625 | 0.348045 | 0.22198 | 6.34E-16 |
| TUBA4A | | 0.386905 | 0.600082 | 0.458924 | 0.540897 | 0.399347 | 6.34E-16 |
| C12orf75 | | 0.647276 | 0.207869 | 0.132721 | 0.243943 | 0.139282 | 8.51E-16 |
| ITGAX | | 1.126158 | 0.115575 | 0.052949 | 0.119453 | 0.04679 | 9.24E-16 |
| MGAT3 | | 1.225354 | 0.087611 | 0.03747 | 0.122331 | 0.04951 | 9.75E-16 |
| FLOT1 | | 0.544271 | 0.257158 | 0.176343 | 0.316383 | 0.196953 | 1E-15 |
| RAB29 | | 0.479375 | 0.341662 | 0.24507 | 0.379467 | 0.249728 | 1.19E-15 |
| LRRC37A2 | | 2.616263 | 0.040942 | 0.006677 | 0.055889 | 0.008161 | 1.35E-15 |
| ACTN4 | | 0.418048 | 0.481511 | 0.36038 | 0.498441 | 0.357998 | 1.55E-15 |
| IFT57 | | 0.386104 | 0.677507 | 0.518423 | 0.555049 | 0.426551 | 1.83E-15 |
| PEA15 | | 0.567324 | 0.312779 | 0.211084 | 0.32118 | 0.206746 | 2.4E-15 |
| CCR9 | | 1.187095 | 0.129609 | 0.056923 | 0.122811 | 0.050598 | 2.46E-15 |
| PTPN22 | | 0.474568 | 0.353977 | 0.254751 | 0.394099 | 0.264418 | 2.54E-15 |
| ZNF827 | | 0.711843 | 0.163571 | 0.099867 | 0.207964 | 0.112622 | 2.7E-15 |
| SCO2 | | 0.632364 | 0.158657 | 0.102353 | 0.217078 | 0.119695 | 3.35E-15 |
| RRP7A | | 0.521199 | 0.254844 | 0.177573 | 0.307268 | 0.18988 | 3.39E-15 |
| RAB31 | | 0.946073 | 0.13891 | 0.0721 | 0.169825 | 0.085963 | 3.87E-15 |
| TOR3A | | 0.514125 | 0.277654 | 0.194418 | 0.328136 | 0.211643 | 4.43E-15 |
| KCNN4 | | 0.786668 | 0.147436 | 0.085466 | 0.187815 | 0.099021 | 6.06E-15 |
| ZMIZ1 | | 0.528794 | 0.230162 | 0.159533 | 0.277285 | 0.168118 | 6.9E-15 |
| KRAS | | 0.450087 | 0.351576 | 0.257353 | 0.400816 | 0.270947 | 9.3E-15 |
| ARPC1B | | 0.329794 | 0.719412 | 0.5724 | 0.635884 | 0.50272 | 1.45E-14 |
| EML6 | | 1.049728 | 0.107405 | 0.051883 | 0.132406 | 0.059304 | 1.52E-14 |
| ABHD17B | | 0.407207 | 0.460316 | 0.347116 | 0.470377 | 0.334059 | 1.52E-14 |
| NUGGC | | 0.879476 | 0.119874 | 0.065159 | 0.162869 | 0.08161 | 1.55E-14 |
| STAT3 | | 0.383633 | 0.472488 | 0.362164 | 0.49916 | 0.358542 | 1.6E-14 |
| DEF6 | | 0.276738 | 0.949286 | 0.783593 | 0.741665 | 0.61099 | 2.26E-14 |
| HPCAL1 | | 0.372955 | 0.47066 | 0.363443 | 0.473975 | 0.332971 | 2.59E-14 |
| MLKL | | 0.84827 | 0.097695 | 0.054265 | 0.150156 | 0.072361 | 2.75E-14 |
| ITGB1 | | 0.551993 | 0.248823 | 0.169716 | 0.270089 | 0.164309 | 2.87E-14 |
| MAPKAPK2 | | 0.293285 | 0.847969 | 0.691979 | 0.682418 | 0.563112 | 2.9E-14 |
| GOLGA8B | | 0.635852 | 0.194638 | 0.125262 | 0.241305 | 0.142546 | 3.43E-14 |
| ATXN1 | | 1.144094 | 0.061622 | 0.027882 | 0.108419 | 0.042982 | 4.09E-14 |
| SRA1 | | 0.43631 | 0.305469 | 0.225748 | 0.361717 | 0.241567 | 5.54E-14 |
| CD1C | | 0.523928 | 0.458986 | 0.319214 | 0.355721 | 0.249184 | 5.7E-14 |
| COBLL1 | | 0.393129 | 0.470861 | 0.35855 | 0.454306 | 0.321545 | 6.3E-14 |
| HNF1B | | 2.470872 | 0.04769 | 0.008602 | 0.052531 | 0.008705 | 6.31E-14 |
| ENTPD1 | | 0.40307 | 0.400173 | 0.30263 | 0.401775 | 0.273667 | 6.57E-14 |
| ZBED2 | | 2.906409 | 0.039407 | 0.005256 | 0.047014 | 0.005985 | 8.75E-14 |
| CTSA | | 0.404971 | 0.358431 | 0.270706 | 0.397697 | 0.26605 | 8.75E-14 |
| CDC42EP3 | | 0.597538 | 0.173359 | 0.11457 | 0.227153 | 0.132209 | 9.27E-14 |
| MAN2A2 | | 0.391113 | 0.420288 | 0.320487 | 0.44687 | 0.312296 | 9.46E-14 |
| SYNE2 | | 0.354217 | 0.625319 | 0.489184 | 0.532502 | 0.397171 | 9.63E-14 |
| ITGB7 | | 0.309184 | 0.885423 | 0.714623 | 0.638043 | 0.524483 | 1.03E-13 |
| FKBP11 | | 0.65718 | 0.388406 | 0.246295 | 0.262173 | 0.165397 | 1.21E-13 |
| DCXR | | 0.463114 | 0.289548 | 0.210044 | 0.33941 | 0.224157 | 1.21E-13 |
| YIF1B | | 0.52825 | 0.266252 | 0.184618 | 0.293116 | 0.18444 | 1.22E-13 |
| CALHM6 | | 0.361227 | 0.58287 | 0.453766 | 0.508755 | 0.37432 | 1.25E-13 |
| PXDN | | 1.515447 | 0.075686 | 0.026474 | 0.087311 | 0.030468 | 1.57E-13 |
| HMOX1 | | 1.073323 | 0.11106 | 0.052778 | 0.120413 | 0.052775 | 1.57E-13 |
| EOLA1 | | 0.542433 | 0.240084 | 0.164844 | 0.291437 | 0.186072 | 1.64E-13 |
| MDP1 | | 0.667406 | 0.13499 | 0.084995 | 0.183257 | 0.098477 | 1.68E-13 |
| PLEK | | 0.312593 | 0.780101 | 0.628132 | 0.619333 | 0.498368 | 1.69E-13 |
| IFI27L2 | | 0.540888 | 0.215168 | 0.147895 | 0.264092 | 0.161589 | 1.84E-13 |
| KNTC1 | | 0.591021 | 0.204309 | 0.135635 | 0.251139 | 0.152884 | 2.04E-13 |
| EEIG1 | | 0.416889 | 0.372688 | 0.279157 | 0.39314 | 0.26877 | 2.19E-13 |
| CCDC50 | | 0.309156 | 0.777466 | 0.627504 | 0.639962 | 0.509793 | 2.2E-13 |
| DNASE1L1 | | 0.564084 | 0.226732 | 0.153358 | 0.265052 | 0.165397 | 2.25E-13 |
| TM9SF2 | | 0.303589 | 0.76739 | 0.621765 | 0.640201 | 0.519587 | 2.95E-13 |
| GOLGB1 | | 0.452278 | 0.24171 | 0.176663 | 0.308227 | 0.196953 | 2.96E-13 |
| PNPLA6 | | 0.404704 | 0.321843 | 0.243117 | 0.368674 | 0.247008 | 3.34E-13 |
| TALDO1 | | 0.470563 | 0.256798 | 0.185327 | 0.323339 | 0.212187 | 4.27E-13 |
| LILRB1 | | 0.368648 | 0.386525 | 0.299367 | 0.408971 | 0.278564 | 4.61E-13 |
| NANS | | 0.685308 | 0.201169 | 0.125102 | 0.229791 | 0.140914 | 5.15E-13 |
| BIK | | 1.109091 | 0.081644 | 0.037849 | 0.115855 | 0.051143 | 5.36E-13 |
| NDST1 | | 0.668943 | 0.128195 | 0.08063 | 0.180619 | 0.097388 | 5.42E-13 |
| VPS28 | | 0.359681 | 0.453715 | 0.353597 | 0.480931 | 0.348205 | 7.66E-13 |
| CHST2 | | 0.604175 | 0.210188 | 0.138271 | 0.242744 | 0.149619 | 9.1E-13 |
| MNDA | | 1.498718 | 0.057913 | 0.020493 | 0.073159 | 0.022307 | 9.21E-13 |
| BATF | | 1.286751 | 0.055732 | 0.022843 | 0.08995 | 0.033732 | 9.29E-13 |
| ETFB | | 0.393361 | 0.376112 | 0.286355 | 0.405613 | 0.28074 | 1.05E-12 |
| SLC16A3 | | 0.613216 | 0.158413 | 0.10356 | 0.210362 | 0.12296 | 1.11E-12 |
| ASCL2 | | 2.054491 | 0.043536 | 0.010481 | 0.05325 | 0.010881 | 1.22E-12 |
| SLA | | 0.323501 | 0.567161 | 0.453235 | 0.540897 | 0.407508 | 1.46E-12 |
| SERPINB9 | | 0.301076 | 0.682217 | 0.553719 | 0.585032 | 0.4358 | 1.53E-12 |
| TNFRSF17 | | 0.669216 | 0.307843 | 0.193587 | 0.221156 | 0.134929 | 1.66E-12 |
| CPPED1 | | 0.68554 | 0.115195 | 0.071625 | 0.148717 | 0.074538 | 1.69E-12 |
| TSPO | | 0.579405 | 0.179981 | 0.120451 | 0.220197 | 0.130577 | 1.71E-12 |
| GOLPH3 | | 0.30989 | 0.677506 | 0.546546 | 0.604222 | 0.470076 | 1.72E-12 |
| SEPHS2 | | 0.402205 | 0.270874 | 0.204971 | 0.331734 | 0.218716 | 1.81E-12 |
| PRSS21 | | 2.038696 | 0.035763 | 0.008704 | 0.055409 | 0.012514 | 1.9E-12 |
| CCR10 | | 1.708534 | 0.05415 | 0.016568 | 0.083953 | 0.031012 | 2.01E-12 |
| SPATS2 | | 0.539576 | 0.229734 | 0.158051 | 0.264332 | 0.165941 | 2.2E-12 |
| BCL2A1 | | 0.56476 | 0.250693 | 0.169486 | 0.244183 | 0.150707 | 2.24E-12 |
| TPK1 | | 0.670934 | 0.155339 | 0.097568 | 0.190693 | 0.10827 | 2.27E-12 |
| PREX1 | | 0.34108 | 0.451086 | 0.35611 | 0.471336 | 0.337323 | 2.3E-12 |
| NFE2L1 | | 0.380481 | 0.358228 | 0.275184 | 0.406332 | 0.284004 | 2.66E-12 |
| CSRP1 | | 0.463276 | 0.253659 | 0.183989 | 0.28592 | 0.183351 | 2.66E-12 |
| BCL2 | | 0.324149 | 0.563644 | 0.450221 | 0.488127 | 0.352557 | 2.78E-12 |
| TENT5C | | 0.267484 | 1.066537 | 0.886044 | 0.692732 | 0.584331 | 2.78E-12 |
| UBALD2 | | 0.336421 | 0.612929 | 0.485442 | 0.556488 | 0.434712 | 2.89E-12 |
| PHPT1 | | 0.507142 | 0.247152 | 0.1739 | 0.289758 | 0.189336 | 2.9E-12 |
| BLMH | | 0.605148 | 0.134628 | 0.088505 | 0.19597 | 0.112078 | 3.37E-12 |
| ECPAS | | 0.435803 | 0.255086 | 0.188581 | 0.318062 | 0.210555 | 3.67E-12 |
| ILK | | 0.569417 | 0.153999 | 0.103778 | 0.212041 | 0.12568 | 3.79E-12 |
| ACAA2 | | 0.504927 | 0.193721 | 0.136515 | 0.251379 | 0.156692 | 4.69E-12 |
| MAP3K8 | | 0.264339 | 0.957194 | 0.79694 | 0.677381 | 0.553863 | 7.66E-12 |
| CD58 | | 0.81714 | 0.09325 | 0.052926 | 0.138163 | 0.070185 | 7.71E-12 |
| VSIR | | 0.493685 | 0.188568 | 0.133922 | 0.251619 | 0.15778 | 8.1E-12 |
| UCKL1 | | 0.317946 | 0.466341 | 0.374104 | 0.48189 | 0.350381 | 8.1E-12 |
| TTLL12 | | 0.571324 | 0.159952 | 0.107647 | 0.206524 | 0.12296 | 8.75E-12 |
| SLAMF6 | | 0.283396 | 0.794305 | 0.652645 | 0.656033 | 0.526115 | 8.99E-12 |
| FGR | | 0.281688 | 0.917645 | 0.754881 | 0.635404 | 0.524483 | 8.99E-12 |
| TFEB | | 0.318929 | 0.575739 | 0.461549 | 0.536819 | 0.418934 | 9.22E-12 |
| DAP | | 0.39655 | 0.345832 | 0.262719 | 0.386903 | 0.270947 | 1.04E-11 |
| CARM1 | | 0.425909 | 0.283227 | 0.210826 | 0.331974 | 0.223613 | 1.07E-11 |
| BID | | 0.620121 | 0.119127 | 0.077506 | 0.181338 | 0.102829 | 1.08E-11 |
| POU2F2 | | 0.256427 | 0.910072 | 0.761875 | 0.717438 | 0.597933 | 1.1E-11 |
| MSTO1 | | 0.43707 | 0.232679 | 0.171864 | 0.287359 | 0.186616 | 1.21E-11 |
| CYRIA | | 0.407799 | 0.346977 | 0.261542 | 0.362437 | 0.251904 | 1.21E-11 |
| ITGAL | | 0.336596 | 0.466746 | 0.369619 | 0.48141 | 0.350381 | 1.28E-11 |
| HDAC7 | | 0.319871 | 0.571627 | 0.457955 | 0.545215 | 0.412949 | 1.29E-11 |
| SMDT1 | | 0.276143 | 0.794882 | 0.656411 | 0.685776 | 0.548422 | 1.35E-11 |
| ARID3A | | 0.463917 | 0.274509 | 0.199023 | 0.295275 | 0.193689 | 1.42E-11 |
| INPP5F | | 0.511566 | 0.237383 | 0.166515 | 0.257616 | 0.165397 | 1.42E-11 |
| UNC50 | | 0.450393 | 0.199364 | 0.145903 | 0.259775 | 0.164853 | 1.7E-11 |
| PMVK | | 0.400248 | 0.309562 | 0.234564 | 0.348045 | 0.23667 | 1.7E-11 |
| NOP58 | | 0.331989 | 0.562117 | 0.446568 | 0.548093 | 0.416213 | 1.7E-11 |
| ZNF593 | | 0.521409 | 0.183945 | 0.128153 | 0.235068 | 0.146355 | 1.71E-11 |
| DCAF12 | | 0.907895 | 0.072356 | 0.038563 | 0.121612 | 0.05876 | 1.75E-11 |
| PPIF | | 0.432051 | 0.164312 | 0.121789 | 0.224274 | 0.135473 | 1.75E-11 |
| CTBS | | 0.417533 | 0.204496 | 0.153107 | 0.263852 | 0.165397 | 1.75E-11 |
| UBE2J1 | | 0.224729 | 1.312918 | 1.123538 | 0.804509 | 0.706746 | 1.89E-11 |
| XPNPEP1 | | 0.486456 | 0.191463 | 0.136661 | 0.250899 | 0.158868 | 1.92E-11 |
| FXYD7 | | 2.05518 | 0.04627 | 0.011134 | 0.048933 | 0.010337 | 1.99E-11 |
| JAK2 | | 0.350784 | 0.335953 | 0.26344 | 0.389542 | 0.270947 | 2.13E-11 |
| UGCG | | 0.282182 | 0.710712 | 0.584452 | 0.604462 | 0.491295 | 2.32E-11 |
| RHOB | | 0.316643 | 0.639291 | 0.51331 | 0.4994 | 0.374864 | 2.35E-11 |
| FBXO10 | | 0.642175 | 0.165006 | 0.105727 | 0.201007 | 0.120783 | 2.56E-11 |
| ENGASE | | 0.539427 | 0.18049 | 0.124185 | 0.218757 | 0.132753 | 2.69E-11 |
| TENT5A | | 0.692377 | 0.145914 | 0.090296 | 0.158311 | 0.084875 | 2.72E-11 |
| COMT | | 0.341233 | 0.356393 | 0.281324 | 0.396258 | 0.277476 | 2.77E-11 |
| JPT1 | | 0.587194 | 0.137633 | 0.091614 | 0.197889 | 0.116431 | 2.97E-11 |
| TCIRG1 | | 0.331874 | 0.51365 | 0.408097 | 0.515951 | 0.391186 | 3.12E-11 |
| IL15RA | | 1.093976 | 0.05766 | 0.027012 | 0.105061 | 0.047878 | 3.13E-11 |
| GLIPR2 | | 0.465888 | 0.172996 | 0.125254 | 0.227633 | 0.139826 | 3.52E-11 |
| SGK1 | | 0.898145 | 0.136706 | 0.073353 | 0.155673 | 0.086507 | 3.77E-11 |
| HSPA1B | | 0.80696 | 0.079873 | 0.045654 | 0.120413 | 0.05876 | 4.39E-11 |
| ZYX | | 0.513863 | 0.165774 | 0.116099 | 0.216359 | 0.131121 | 4.39E-11 |
| TBX21 | | 0.866569 | 0.075254 | 0.041273 | 0.100024 | 0.043526 | 4.47E-11 |
| CASP7 | | 0.381797 | 0.217438 | 0.166879 | 0.284481 | 0.183351 | 4.5E-11 |
| HDGFL2 | | 0.377908 | 0.300971 | 0.231613 | 0.353082 | 0.243199 | 4.51E-11 |
| GRAMD1B | | 1.863207 | 0.041662 | 0.011451 | 0.050852 | 0.01197 | 4.68E-11 |
| ANKDD1A | | 1.116957 | 0.051325 | 0.023664 | 0.077716 | 0.028836 | 5.45E-11 |
| TMEM184B | | 0.818116 | 0.180312 | 0.10227 | 0.190933 | 0.118063 | 5.45E-11 |
| ITPK1 | | 0.358021 | 0.343946 | 0.268359 | 0.384984 | 0.272579 | 6.16E-11 |
| ENDOG | | 0.617126 | 0.123761 | 0.080688 | 0.175342 | 0.100653 | 6.63E-11 |
| ACADVL | | 0.379408 | 0.442952 | 0.340521 | 0.441113 | 0.332971 | 6.84E-11 |
| IVD | | 0.506538 | 0.142526 | 0.100325 | 0.19621 | 0.116431 | 7.23E-11 |
| PLCG2 | | 0.153885 | 1.518633 | 1.364988 | 0.908131 | 0.836235 | 7.45E-11 |
| CERS6 | | 0.383845 | 0.288332 | 0.220975 | 0.335812 | 0.230141 | 7.58E-11 |
| PLOD3 | | 0.51698 | 0.163046 | 0.113942 | 0.217318 | 0.133841 | 7.75E-11 |
| NAMPT | | 0.406939 | 0.233261 | 0.17593 | 0.2852 | 0.186072 | 7.81E-11 |
| SOX5 | | 0.640073 | 0.242594 | 0.155668 | 0.207244 | 0.1284 | 8.3E-11 |
| IDI1 | | 0.367349 | 0.431847 | 0.33477 | 0.448309 | 0.336779 | 8.4E-11 |
| ETFA | | 0.348772 | 0.317535 | 0.249345 | 0.369873 | 0.257889 | 8.42E-11 |
| SH3BGRL3 | | 0.201529 | 1.232506 | 1.071822 | 0.83929 | 0.731774 | 8.52E-11 |
| SGPP1 | | 0.424576 | 0.305756 | 0.227806 | 0.307748 | 0.208923 | 9.19E-11 |
| RNH1 | | 0.294678 | 0.585657 | 0.477459 | 0.554569 | 0.429271 | 9.45E-11 |
| JAK3 | | 0.291003 | 0.506638 | 0.414092 | 0.508995 | 0.379217 | 1.05E-10 |
| TFRC | | 0.393539 | 0.319919 | 0.243541 | 0.355481 | 0.24864 | 1.06E-10 |
| PIK3IP1 | | 0.47979 | 0.354273 | 0.254043 | 0.311825 | 0.217084 | 1.07E-10 |
| ANKRD13A | | 0.299723 | 0.525294 | 0.426753 | 0.530343 | 0.405876 | 1.08E-10 |
| ZMAT3 | | 0.448445 | 0.253663 | 0.185892 | 0.277764 | 0.182807 | 1.18E-10 |
| CD27 | | 0.728973 | 0.213811 | 0.128999 | 0.192612 | 0.118607 | 1.19E-10 |
| ASB6 | | 0.498815 | 0.164044 | 0.116092 | 0.218038 | 0.134929 | 1.19E-10 |
| SOCS1 | | 0.427196 | 0.397071 | 0.295304 | 0.353082 | 0.254081 | 1.23E-10 |
| NFKBIZ | | 0.327308 | 0.472505 | 0.376597 | 0.456704 | 0.335691 | 1.23E-10 |
| NEDD4L | | 0.666124 | 0.120344 | 0.075841 | 0.158071 | 0.088683 | 1.36E-10 |
| C4orf48 | | 0.640461 | 0.095737 | 0.061416 | 0.146318 | 0.079434 | 1.43E-10 |
| HECTD3 | | 0.36731 | 0.247725 | 0.192043 | 0.310866 | 0.209467 | 1.53E-10 |
| CDV3 | | 0.356269 | 0.410762 | 0.32088 | 0.440393 | 0.319369 | 1.54E-10 |
| LDLR | | 1.32801 | 0.07751 | 0.030873 | 0.100024 | 0.04679 | 1.55E-10 |
| HTRA2 | | 0.465476 | 0.196284 | 0.142155 | 0.246582 | 0.15778 | 1.6E-10 |
| SMAP2 | | 0.189491 | 1.316152 | 1.154154 | 0.873351 | 0.772579 | 1.6E-10 |
| TAF6 | | 0.362001 | 0.270403 | 0.210397 | 0.324059 | 0.220892 | 1.67E-10 |
| SKAP2 | | 0.293564 | 0.539202 | 0.439927 | 0.530103 | 0.404788 | 1.67E-10 |
| FAN1 | | 0.729012 | 0.111647 | 0.067358 | 0.141761 | 0.076714 | 1.73E-10 |
| ELK3 | | 0.453779 | 0.174087 | 0.127106 | 0.21372 | 0.130033 | 1.73E-10 |
| ANXA2 | | 0.358295 | 0.574388 | 0.448072 | 0.460302 | 0.355822 | 1.77E-10 |
| YARS1 | | 0.479408 | 0.148619 | 0.1066 | 0.218997 | 0.135473 | 1.93E-10 |
| XBP1 | | 0.639939 | 0.352721 | 0.226355 | 0.216839 | 0.139282 | 1.98E-10 |
| GGA2 | | 0.17135 | 1.286172 | 1.142135 | 0.841689 | 0.765506 | 2.1E-10 |
| QPCTL | | 0.566754 | 0.140199 | 0.094653 | 0.194291 | 0.117519 | 2.17E-10 |
| MGAT2 | | 0.338876 | 0.474726 | 0.375346 | 0.456464 | 0.337867 | 2.36E-10 |
| SH3GLB2 | | 0.329206 | 0.266145 | 0.211844 | 0.32142 | 0.217084 | 2.39E-10 |
| AIM2 | | 0.928289 | 0.077317 | 0.040629 | 0.103142 | 0.048422 | 2.45E-10 |
| LAMTOR2 | | 0.340792 | 0.3954 | 0.312211 | 0.426001 | 0.311752 | 2.66E-10 |
| CTSD | | 0.519185 | 0.117615 | 0.082068 | 0.185416 | 0.110446 | 2.83E-10 |
| CHST15 | | 0.406386 | 0.249088 | 0.18794 | 0.289278 | 0.193689 | 2.86E-10 |
| MTHFD2 | | 0.506633 | 0.185759 | 0.130749 | 0.233629 | 0.150163 | 3.23E-10 |
| ARPC5L | | 0.324155 | 0.443288 | 0.354084 | 0.451427 | 0.328618 | 3.25E-10 |
| MTFR1L | | 0.473113 | 0.160697 | 0.115767 | 0.205085 | 0.125136 | 3.36E-10 |
| CALHM2 | | 1.212654 | 0.055847 | 0.024096 | 0.074118 | 0.028292 | 3.37E-10 |
| SESN1 | | 0.600049 | 0.25942 | 0.171148 | 0.242024 | 0.161589 | 3.44E-10 |
| MVP | | 0.362688 | 0.213609 | 0.166127 | 0.284721 | 0.186616 | 3.45E-10 |
| RIC3 | | 0.652031 | 0.137371 | 0.087421 | 0.168146 | 0.098477 | 3.66E-10 |
| PSMD4 | | 0.318419 | 0.26005 | 0.208547 | 0.33917 | 0.230141 | 3.67E-10 |
| WIPI1 | | 1.047677 | 0.067554 | 0.032679 | 0.112257 | 0.056583 | 3.67E-10 |
| APOL1 | | 1.035353 | 0.050273 | 0.024528 | 0.094507 | 0.042982 | 3.82E-10 |
| IFI30 | | 0.232455 | 0.936256 | 0.796927 | 0.711442 | 0.578346 | 3.82E-10 |
| YIF1A | | 0.43755 | 0.247653 | 0.182864 | 0.292636 | 0.200762 | 4.38E-10 |
| SPATA13 | | 0.779871 | 0.113019 | 0.065824 | 0.132406 | 0.070185 | 4.6E-10 |
| ACTR8 | | 0.400086 | 0.221166 | 0.167603 | 0.277045 | 0.184984 | 4.64E-10 |
| MPST | | 0.434084 | 0.191061 | 0.141416 | 0.244903 | 0.158324 | 4.87E-10 |
| NPM3 | | 0.520515 | 0.143264 | 0.099873 | 0.190933 | 0.115887 | 4.89E-10 |
| S100PBP | | 0.267553 | 0.659011 | 0.547458 | 0.603262 | 0.474429 | 4.89E-10 |
| SLC7A5 | | 0.672871 | 0.175023 | 0.109784 | 0.193332 | 0.120783 | 4.91E-10 |
| NT5DC2 | | 0.902212 | 0.157381 | 0.084209 | 0.138402 | 0.077258 | 4.92E-10 |
| FNDC3A | | 0.351834 | 0.364726 | 0.285795 | 0.401055 | 0.293798 | 4.92E-10 |
| CTSB | | 0.263145 | 0.407189 | 0.339298 | 0.442792 | 0.317193 | 4.92E-10 |
| GLDC | | 1.358557 | 0.104608 | 0.040794 | 0.1065 | 0.053863 | 5.36E-10 |
| CPT1A | | 0.610724 | 0.116931 | 0.076574 | 0.164068 | 0.095212 | 5.55E-10 |
| PML | | 0.304127 | 0.407462 | 0.330016 | 0.442072 | 0.326986 | 5.71E-10 |
| PLK3 | | 0.523308 | 0.203363 | 0.141495 | 0.218757 | 0.139826 | 5.96E-10 |
| ARMCX3 | | 0.44812 | 0.161352 | 0.11827 | 0.222116 | 0.14037 | 6.11E-10 |
| ABHD16A | | 0.408022 | 0.169001 | 0.127369 | 0.229312 | 0.144723 | 6.11E-10 |
| TMED9 | | 0.387201 | 0.330457 | 0.252671 | 0.362437 | 0.261153 | 6.11E-10 |
| ADAM15 | | 0.624585 | 0.131203 | 0.085099 | 0.166947 | 0.098477 | 6.55E-10 |
| UFD1 | | 0.43237 | 0.187893 | 0.139237 | 0.248021 | 0.162133 | 6.65E-10 |
| TP53I3 | | 1.223685 | 0.047884 | 0.020503 | 0.074598 | 0.02938 | 6.82E-10 |
| GRPEL1 | | 0.38792 | 0.194694 | 0.148791 | 0.254018 | 0.165941 | 6.83E-10 |
| ACP5 | | 0.319648 | 0.465874 | 0.373288 | 0.454066 | 0.340044 | 7.2E-10 |
| OPTN | | 0.369385 | 0.213468 | 0.165248 | 0.261454 | 0.171382 | 7.32E-10 |
| PIM2 | | 0.25665 | 1.209592 | 1.012464 | 0.748381 | 0.662677 | 7.98E-10 |
| CD44 | | 0.179551 | 1.437331 | 1.269131 | 0.834253 | 0.747552 | 7.98E-10 |
| RTN3 | | 0.414536 | 0.240311 | 0.180296 | 0.294315 | 0.202394 | 8.57E-10 |
| BMPR2 | | 0.730948 | 0.101095 | 0.060911 | 0.123771 | 0.0642 | 8.59E-10 |
| PPM1M | | 0.438639 | 0.200297 | 0.147785 | 0.242264 | 0.157236 | 8.88E-10 |
| SGMS1 | | 0.386194 | 0.275462 | 0.210769 | 0.32118 | 0.225789 | 9.1E-10 |
| HAGHL | | 0.846273 | 0.095986 | 0.053389 | 0.120173 | 0.062568 | 9.15E-10 |
| ARSA | | 0.317663 | 0.355564 | 0.285294 | 0.397937 | 0.28346 | 9.25E-10 |
| MIF4GD | | 0.378546 | 0.253618 | 0.195086 | 0.304629 | 0.210555 | 9.52E-10 |
| SYT11 | | 0.655181 | 0.142548 | 0.090518 | 0.150396 | 0.084875 | 9.81E-10 |
| PLXNC1 | | 0.417671 | 0.202237 | 0.151401 | 0.248021 | 0.163221 | 1.05E-09 |
| PKIG | | 0.284492 | 0.698852 | 0.573779 | 0.550732 | 0.437432 | 1.07E-09 |
| UGT8 | | 0.908117 | 0.088416 | 0.047115 | 0.109139 | 0.054407 | 1.08E-09 |
| YIPF6 | | 0.497532 | 0.117788 | 0.083432 | 0.171744 | 0.101197 | 1.08E-09 |
| CCDC88C | | 0.250481 | 0.715541 | 0.601496 | 0.662269 | 0.522307 | 1.09E-09 |
| SLC2A4RG | | 0.404726 | 0.148498 | 0.112173 | 0.203646 | 0.126224 | 1.18E-09 |
| DALRD3 | | 0.716872 | 0.067846 | 0.041279 | 0.102663 | 0.048966 | 1.21E-09 |
| PM20D2 | | 0.529746 | 0.160776 | 0.111366 | 0.204845 | 0.129489 | 1.22E-09 |
| NTPCR | | 0.586626 | 0.104637 | 0.069678 | 0.144879 | 0.080522 | 1.24E-09 |
| SLC1A4 | | 0.580625 | 0.180781 | 0.120883 | 0.194771 | 0.121872 | 1.28E-09 |
| ARHGEF3 | | 0.365104 | 0.353299 | 0.274306 | 0.362677 | 0.264418 | 1.34E-09 |
| UAP1 | | 0.502487 | 0.225731 | 0.159341 | 0.250899 | 0.169206 | 1.37E-09 |
| RUNX3 | | 0.294415 | 0.380487 | 0.31025 | 0.412329 | 0.299238 | 1.39E-09 |
| R3HDM4 | | 0.191938 | 1.103236 | 0.965805 | 0.801391 | 0.708379 | 1.39E-09 |
| TCTN3 | | 0.443743 | 0.162728 | 0.119641 | 0.224994 | 0.143634 | 1.42E-09 |
| RBM26 | | 0.268253 | 0.542068 | 0.450092 | 0.558647 | 0.428727 | 1.43E-09 |
| ATP6V1E1 | | 0.287105 | 0.359973 | 0.295014 | 0.413289 | 0.297606 | 1.5E-09 |
| LPCAT4 | | 0.436227 | 0.182564 | 0.134926 | 0.233629 | 0.152884 | 1.5E-09 |
| MDGA1 | | 0.832232 | 0.081229 | 0.045623 | 0.100504 | 0.047878 | 1.52E-09 |
| PLAGL1 | | 0.518877 | 0.137249 | 0.095788 | 0.166467 | 0.097388 | 1.56E-09 |
| GSTK1 | | 0.277401 | 0.628456 | 0.518524 | 0.590789 | 0.468444 | 1.62E-09 |
| PRAG1 | | 0.671738 | 0.126929 | 0.079679 | 0.14344 | 0.080522 | 1.73E-09 |
| MAD1L1 | | 0.370217 | 0.356422 | 0.275751 | 0.372272 | 0.272579 | 1.75E-09 |
| DYRK4 | | 0.428109 | 0.159715 | 0.118706 | 0.218997 | 0.139282 | 1.78E-09 |
| HINT2 | | 0.42674 | 0.16706 | 0.124283 | 0.228112 | 0.146899 | 1.78E-09 |
| SLC6A6 | | 0.377248 | 0.29996 | 0.230941 | 0.33893 | 0.239935 | 1.82E-09 |
| CHST12 | | 0.308 | 0.298266 | 0.240928 | 0.33965 | 0.234494 | 1.91E-09 |
| CCDC137 | | 0.455741 | 0.178263 | 0.129978 | 0.220916 | 0.141458 | 1.94E-09 |
| MTX1 | | 0.434703 | 0.163221 | 0.120758 | 0.227872 | 0.147443 | 1.95E-09 |
| ZNF587B | | 0.398574 | 0.177946 | 0.134991 | 0.234109 | 0.150707 | 1.95E-09 |
| LIMK1 | | 0.362052 | 0.308301 | 0.239876 | 0.347325 | 0.249728 | 2E-09 |
| PIM1 | | 0.264896 | 0.784402 | 0.652825 | 0.574478 | 0.466268 | 2.02E-09 |
| ENTR1 | | 0.321357 | 0.35628 | 0.285137 | 0.39314 | 0.286725 | 2.04E-09 |
| PISD | | 0.434461 | 0.150533 | 0.11139 | 0.199808 | 0.123504 | 2.06E-09 |
| HSD17B10 | | 0.349846 | 0.371133 | 0.291216 | 0.395059 | 0.291077 | 2.06E-09 |
| ZDHHC6 | | 0.365154 | 0.286171 | 0.222179 | 0.335572 | 0.239391 | 2.2E-09 |
| PTPN12 | | 0.340951 | 0.309068 | 0.244016 | 0.33893 | 0.237758 | 2.2E-09 |
| PNMA1 | | 0.508622 | 0.178206 | 0.12526 | 0.203406 | 0.1284 | 2.32E-09 |
| SAMM50 | | 0.376199 | 0.162815 | 0.125443 | 0.23219 | 0.148531 | 2.39E-09 |
| TIMM10 | | 0.354057 | 0.34975 | 0.273638 | 0.367954 | 0.265506 | 2.45E-09 |
| CASP10 | | 0.347451 | 0.267426 | 0.210189 | 0.317102 | 0.21926 | 2.45E-09 |
| ATP5F1B | | 0.340404 | 0.369897 | 0.292152 | 0.408491 | 0.300871 | 2.46E-09 |
| P4HB | | 0.504261 | 0.207023 | 0.145955 | 0.233149 | 0.153972 | 2.47E-09 |
| NDUFAF1 | | 0.520323 | 0.109475 | 0.076328 | 0.164548 | 0.097388 | 2.47E-09 |
| DRAM1 | | 0.77334 | 0.091102 | 0.0533 | 0.130967 | 0.072361 | 2.48E-09 |
| LZTFL1 | | 0.393389 | 0.235791 | 0.179517 | 0.274406 | 0.186072 | 2.58E-09 |
| ELL2 | | 0.500234 | 0.354368 | 0.250535 | 0.296954 | 0.217628 | 2.62E-09 |
| DSE | | 0.456377 | 0.277637 | 0.202345 | 0.277764 | 0.191513 | 2.62E-09 |
| USP40 | | 0.556878 | 0.123068 | 0.083658 | 0.168146 | 0.100653 | 2.95E-09 |
| NSUN4 | | 0.301112 | 0.280969 | 0.228042 | 0.329096 | 0.229053 | 2.98E-09 |
| FUCA2 | | 0.645698 | 0.071191 | 0.045504 | 0.119693 | 0.063112 | 3.03E-09 |
| ABCA7 | | 0.257064 | 0.581158 | 0.486307 | 0.556488 | 0.434712 | 3.06E-09 |
| ZDHHC20 | | 0.305988 | 0.28043 | 0.226837 | 0.348045 | 0.244287 | 3.09E-09 |
| ERGIC2 | | 0.355621 | 0.251831 | 0.196815 | 0.305349 | 0.211643 | 3.09E-09 |
| FAM167A | | 0.867928 | 0.092569 | 0.050722 | 0.103142 | 0.051143 | 3.1E-09 |
| EZR | | 0.151062 | 1.556178 | 1.401474 | 0.903094 | 0.834603 | 3.1E-09 |
| LIME1 | | 0.502864 | 0.180955 | 0.127701 | 0.19597 | 0.123504 | 3.2E-09 |
| SCIMP | | 0.343926 | 0.610014 | 0.480627 | 0.448069 | 0.355822 | 3.41E-09 |
| POMT1 | | 0.724956 | 0.089505 | 0.054152 | 0.129288 | 0.071273 | 3.42E-09 |
| COASY | | 0.330109 | 0.258202 | 0.205393 | 0.314464 | 0.218172 | 3.5E-09 |
| KCNK6 | | 0.636494 | 0.094593 | 0.060849 | 0.141281 | 0.080522 | 3.55E-09 |
| LRIG1 | | 0.399576 | 0.198751 | 0.150669 | 0.238666 | 0.156692 | 3.6E-09 |
| CHST14 | | 0.577754 | 0.126253 | 0.08459 | 0.154713 | 0.090316 | 3.75E-09 |
| ARFIP2 | | 0.397569 | 0.169421 | 0.128614 | 0.224754 | 0.145811 | 3.84E-09 |
| RGS2 | | 0.221606 | 0.956553 | 0.820349 | 0.549532 | 0.426007 | 3.86E-09 |
| ACP3 | | 2.323142 | 0.029121 | 0.005819 | 0.034541 | 0.005985 | 4.05E-09 |
| ZC3H7A | | 0.403291 | 0.203763 | 0.154071 | 0.258575 | 0.174102 | 4.07E-09 |
| ASAP1 | | 0.425274 | 0.192608 | 0.143435 | 0.228112 | 0.147443 | 4.1E-09 |
| SSH1 | | 0.47753 | 0.126085 | 0.090555 | 0.174622 | 0.10555 | 4.21E-09 |
| PRKD3 | | 0.299168 | 0.416892 | 0.338817 | 0.453586 | 0.340588 | 4.23E-09 |
| SQSTM1 | | 0.24107 | 0.758365 | 0.641666 | 0.652675 | 0.529924 | 4.24E-09 |
| GPCPD1 | | 0.22525 | 0.778666 | 0.666108 | 0.674023 | 0.54679 | 4.28E-09 |
| RGS10 | | 0.46573 | 0.126006 | 0.091241 | 0.185176 | 0.114255 | 4.29E-09 |
| FAM219B | | 0.394629 | 0.16703 | 0.127057 | 0.210602 | 0.132753 | 4.29E-09 |
| UBAP2 | | 0.616057 | 0.111071 | 0.072468 | 0.157112 | 0.093036 | 4.33E-09 |
| CPTP | | 0.446015 | 0.154593 | 0.113482 | 0.206284 | 0.131665 | 4.55E-09 |
| RTCA | | 0.32918 | 0.243739 | 0.194013 | 0.307748 | 0.212731 | 4.62E-09 |
| CORO7 | | 0.265456 | 0.599713 | 0.498923 | 0.552171 | 0.431447 | 4.62E-09 |
| ADPRH | | 0.845019 | 0.085324 | 0.0475 | 0.114656 | 0.060936 | 4.73E-09 |
| CHST11 | | 0.285875 | 0.520369 | 0.426829 | 0.502758 | 0.390098 | 4.77E-09 |
| MTIF2 | | 0.356294 | 0.19995 | 0.156195 | 0.256656 | 0.171382 | 4.84E-09 |
| PTTG1IP | | 0.314969 | 0.298209 | 0.239721 | 0.354282 | 0.254081 | 4.94E-09 |
| EHD1 | | 0.256245 | 0.677024 | 0.566848 | 0.615735 | 0.495647 | 5.05E-09 |
| GOLT1B | | 0.312048 | 0.349177 | 0.281261 | 0.382826 | 0.275843 | 5.38E-09 |
| B3GAT3 | | 0.308753 | 0.218769 | 0.176621 | 0.284481 | 0.190424 | 5.41E-09 |
| GTPBP6 | | 0.263136 | 0.482966 | 0.402444 | 0.50036 | 0.380849 | 5.52E-09 |
| RNPEP | | 0.322231 | 0.276135 | 0.220861 | 0.329336 | 0.232862 | 5.72E-09 |
| FES | | 0.514862 | 0.154424 | 0.108075 | 0.189494 | 0.118607 | 6.01E-09 |
| NIPA1 | | 0.556401 | 0.118131 | 0.080329 | 0.152555 | 0.089227 | 6.17E-09 |
| LILRB4 | | 1.032714 | 0.055183 | 0.026973 | 0.094747 | 0.04679 | 6.31E-09 |
| NDRG1 | | 0.413578 | 0.237139 | 0.178034 | 0.266491 | 0.183351 | 6.47E-09 |
| ATP5MF | | 0.351518 | 0.255778 | 0.200468 | 0.324778 | 0.228509 | 6.47E-09 |
| VMP1 | | 0.311759 | 0.241555 | 0.194611 | 0.304869 | 0.210011 | 6.52E-09 |
| FKBP5 | | 0.69659 | 0.091134 | 0.056232 | 0.140321 | 0.081066 | 6.56E-09 |
| TRIB1 | | 0.668656 | 0.229015 | 0.144071 | 0.183257 | 0.117519 | 6.76E-09 |
| LSM7 | | 0.25262 | 0.636945 | 0.534633 | 0.603982 | 0.483134 | 6.78E-09 |
| EBI3 | | 1.277409 | 0.049867 | 0.020572 | 0.062365 | 0.023395 | 6.94E-09 |
| MFHAS1 | | 0.691699 | 0.121125 | 0.074991 | 0.130247 | 0.072361 | 7.02E-09 |
| GPN2 | | 0.37085 | 0.155277 | 0.12008 | 0.218518 | 0.141458 | 7.58E-09 |
| DPAGT1 | | 0.429273 | 0.151729 | 0.112679 | 0.212521 | 0.137106 | 7.66E-09 |
| TNFSF10 | | 0.425241 | 0.17951 | 0.133684 | 0.227872 | 0.150163 | 7.87E-09 |
| ST7 | | 1.092104 | 0.040599 | 0.019044 | 0.083233 | 0.038085 | 7.94E-09 |
| CITED2 | | 0.273035 | 0.347944 | 0.28795 | 0.3574 | 0.252448 | 8.1E-09 |
| DERA | | 0.459849 | 0.170103 | 0.123675 | 0.225953 | 0.150163 | 9.18E-09 |
| MAPK6 | | 0.387203 | 0.21832 | 0.16693 | 0.273447 | 0.188248 | 9.31E-09 |
| CPSF1 | | 0.360613 | 0.22089 | 0.172036 | 0.277764 | 0.190968 | 9.44E-09 |
| CENPB | | 0.320117 | 0.246491 | 0.197441 | 0.307508 | 0.213275 | 9.76E-09 |
| MANEA | | 0.514862 | 0.169854 | 0.118874 | 0.187335 | 0.118607 | 1E-08 |
| ZNF296 | | 0.301611 | 0.267253 | 0.216834 | 0.311346 | 0.217628 | 1.02E-08 |
| PPA2 | | 0.598904 | 0.116821 | 0.077132 | 0.170305 | 0.10555 | 1.08E-08 |
| MED16 | | 0.282731 | 0.321033 | 0.2639 | 0.365795 | 0.260065 | 1.11E-08 |
| NME1 | | 0.428844 | 0.245387 | 0.182288 | 0.26817 | 0.18716 | 1.11E-08 |
| GPI | | 0.365554 | 0.214949 | 0.166837 | 0.281123 | 0.192057 | 1.11E-08 |
| KEAP1 | | 0.439685 | 0.129577 | 0.095537 | 0.189014 | 0.119151 | 1.11E-08 |
| DDOST | | 0.374889 | 0.35634 | 0.274797 | 0.358839 | 0.268226 | 1.13E-08 |
| PRDX4 | | 0.494747 | 0.216613 | 0.153727 | 0.225714 | 0.151251 | 1.15E-08 |
| RNF166 | | 0.413331 | 0.148996 | 0.111879 | 0.199808 | 0.127312 | 1.15E-08 |
| UBR3 | | 0.352252 | 0.243085 | 0.190423 | 0.298153 | 0.208379 | 1.16E-08 |
| YPEL3 | | 0.206996 | 0.931522 | 0.807014 | 0.722475 | 0.626224 | 1.2E-08 |
| DDI2 | | 0.342527 | 0.208154 | 0.164162 | 0.269609 | 0.183351 | 1.26E-08 |
| MAP3K5 | | 0.300221 | 0.311161 | 0.252703 | 0.35764 | 0.255169 | 1.27E-08 |
| MTCH1 | | 0.256518 | 0.470239 | 0.393639 | 0.493644 | 0.372688 | 1.28E-08 |
| TBKBP1 | | 0.698675 | 0.123892 | 0.076335 | 0.128808 | 0.072361 | 1.3E-08 |
| SRI | | 0.281744 | 0.452234 | 0.372006 | 0.474934 | 0.362894 | 1.38E-08 |
| USP9Y | | 0.847009 | 0.12789 | 0.071099 | 0.136004 | 0.081066 | 1.39E-08 |
| RREB1 | | 0.304277 | 0.273865 | 0.221789 | 0.335092 | 0.239391 | 1.39E-08 |
| GYG1 | | 0.658255 | 0.077244 | 0.048945 | 0.129527 | 0.073449 | 1.41E-08 |
| NPRL3 | | 0.289299 | 0.285134 | 0.233325 | 0.328856 | 0.233406 | 1.43E-08 |
| NFAT5 | | 0.322068 | 0.373357 | 0.298657 | 0.397937 | 0.300326 | 1.47E-08 |
| ZNF66 | | 0.729367 | 0.088909 | 0.053627 | 0.112257 | 0.059848 | 1.51E-08 |
| TWF2 | | 0.253904 | 0.646855 | 0.542469 | 0.573279 | 0.471708 | 1.51E-08 |
| STYXL1 | | 0.351131 | 0.150964 | 0.118351 | 0.21516 | 0.138738 | 1.55E-08 |
| SMARCB1 | | 0.257359 | 0.614276 | 0.513914 | 0.582634 | 0.470076 | 1.58E-08 |
| SEC24D | | 0.674333 | 0.140672 | 0.088148 | 0.163588 | 0.101741 | 1.6E-08 |
| CDC34 | | 0.324803 | 0.214957 | 0.171623 | 0.277285 | 0.190968 | 1.69E-08 |
| PLTP | | 1.169279 | 0.039336 | 0.017491 | 0.059487 | 0.022307 | 1.72E-08 |
| PKM | | 0.295118 | 0.458461 | 0.373648 | 0.470137 | 0.355822 | 1.73E-08 |
| POU2AF1 | | 0.167849 | 1.371658 | 1.221007 | 0.817222 | 0.760065 | 1.73E-08 |
| ADIPOR2 | | 0.334829 | 0.199751 | 0.158378 | 0.258335 | 0.173558 | 1.73E-08 |
| CREBZF | | 0.268906 | 0.347345 | 0.288278 | 0.383305 | 0.278564 | 1.73E-08 |
| ALG2 | | 0.335652 | 0.238157 | 0.188721 | 0.299832 | 0.208923 | 1.75E-08 |
| NEU1 | | 0.386745 | 0.249466 | 0.190805 | 0.28592 | 0.202938 | 1.77E-08 |
| ARHGAP31 | | 1.142432 | 0.044403 | 0.020115 | 0.070521 | 0.029924 | 1.77E-08 |
| TTLL5 | | 0.393185 | 0.214371 | 0.163232 | 0.254977 | 0.174646 | 1.77E-08 |
| H2AJ | | 0.366633 | 0.185043 | 0.143518 | 0.239386 | 0.159412 | 1.78E-08 |
| ATP5PD | | 0.301268 | 0.19834 | 0.16096 | 0.268889 | 0.182263 | 1.92E-08 |
| DYNLT1 | | 0.341312 | 0.199588 | 0.157539 | 0.257136 | 0.174646 | 1.93E-08 |
| CSTB | | 0.347562 | 0.199514 | 0.1568 | 0.26865 | 0.18444 | 1.93E-08 |
| HEATR3 | | 0.395073 | 0.160931 | 0.12238 | 0.215879 | 0.141458 | 1.94E-08 |
| TFDP1 | | 0.323583 | 0.277428 | 0.221688 | 0.329336 | 0.232862 | 1.99E-08 |
| GPR183 | | 0.236651 | 0.974803 | 0.827328 | 0.627968 | 0.531012 | 1.99E-08 |
| PCBP1 | | 0.192867 | 0.926192 | 0.810293 | 0.761813 | 0.658324 | 2.04E-08 |
| G6PC3 | | 0.428652 | 0.122103 | 0.090717 | 0.17774 | 0.11099 | 2.07E-08 |
| ABHD12 | | 0.494079 | 0.113139 | 0.08033 | 0.167906 | 0.104461 | 2.24E-08 |
| UFSP2 | | 0.376399 | 0.132309 | 0.101926 | 0.192372 | 0.121872 | 2.3E-08 |
| COX18 | | 0.37161 | 0.144711 | 0.11185 | 0.200528 | 0.1284 | 2.31E-08 |
| PLOD1 | | 0.729387 | 0.06432 | 0.038795 | 0.112257 | 0.060936 | 2.31E-08 |
| IFT20 | | 0.28887 | 0.276138 | 0.226031 | 0.337251 | 0.239935 | 2.38E-08 |
| POLR2L | | 0.269043 | 0.556564 | 0.461876 | 0.511633 | 0.40642 | 2.52E-08 |
| SLC39A14 | | 1.063138 | 0.038412 | 0.018384 | 0.07148 | 0.031012 | 2.59E-08 |
| ANP32E | | 0.315422 | 0.234429 | 0.188391 | 0.28568 | 0.196953 | 2.66E-08 |
| SEC14L1 | | 0.319902 | 0.480156 | 0.384664 | 0.437515 | 0.34222 | 2.72E-08 |
| SEC22A | | 0.467556 | 0.122903 | 0.088882 | 0.166947 | 0.102829 | 2.74E-08 |
| RIN3 | | 0.240557 | 0.855458 | 0.724075 | 0.592228 | 0.513602 | 2.75E-08 |
| TBC1D9B | | 0.218274 | 0.777693 | 0.6685 | 0.664908 | 0.545702 | 2.8E-08 |
| NIPAL2 | | 0.541649 | 0.147622 | 0.101414 | 0.175342 | 0.110446 | 2.81E-08 |
| PLEKHG7 | | 1.028361 | 0.061126 | 0.029968 | 0.077477 | 0.035365 | 2.85E-08 |
| FCRL3 | | 0.354369 | 0.463499 | 0.362554 | 0.341809 | 0.253536 | 2.87E-08 |
| ODF3B | | 0.543973 | 0.117011 | 0.080255 | 0.16023 | 0.098477 | 2.9E-08 |
| NIT2 | | 0.444448 | 0.129468 | 0.095142 | 0.180139 | 0.114255 | 2.91E-08 |
| ARL4C | | 0.267474 | 0.549483 | 0.456496 | 0.503238 | 0.392274 | 2.91E-08 |
| NFIL3 | | 0.56941 | 0.104403 | 0.070357 | 0.147278 | 0.088683 | 2.95E-08 |
| GFPT1 | | 0.483425 | 0.127302 | 0.091056 | 0.181099 | 0.114799 | 2.95E-08 |
| MCM6 | | 0.731321 | 0.085909 | 0.051747 | 0.110818 | 0.059848 | 2.96E-08 |
| PAQR7 | | 1.053416 | 0.056832 | 0.027383 | 0.073639 | 0.032644 | 3E-08 |
| PLEKHH3 | | 1.73891 | 0.019257 | 0.005769 | 0.044855 | 0.013602 | 3.11E-08 |
| PSME3 | | 0.508813 | 0.093493 | 0.065707 | 0.148717 | 0.088683 | 3.14E-08 |
| C2CD5 | | 0.329124 | 0.279871 | 0.222783 | 0.323819 | 0.232318 | 3.29E-08 |
| HAX1 | | 0.389849 | 0.18404 | 0.140461 | 0.244423 | 0.165397 | 3.32E-08 |
| ST3GAL5 | | 0.29629 | 0.403894 | 0.328909 | 0.41065 | 0.316649 | 3.39E-08 |
| DTX3 | | 1.116173 | 0.039048 | 0.018013 | 0.072919 | 0.032644 | 3.47E-08 |
| SERPINB1 | | 0.247056 | 0.6075 | 0.511888 | 0.542576 | 0.432535 | 3.55E-08 |
| EIF1AD | | 0.292677 | 0.319517 | 0.260849 | 0.368194 | 0.267138 | 3.67E-08 |
| MVD | | 0.385555 | 0.158308 | 0.121182 | 0.212761 | 0.139282 | 3.89E-08 |
| IDE | | 0.385579 | 0.137659 | 0.105374 | 0.198609 | 0.128945 | 3.95E-08 |
| PYCARD | | 0.271516 | 0.282581 | 0.234104 | 0.336532 | 0.242111 | 4.07E-08 |
| PYGB | | 0.280298 | 0.252107 | 0.20759 | 0.306548 | 0.215452 | 4.11E-08 |
| MAP2K1 | | 0.255482 | 0.457075 | 0.382895 | 0.477333 | 0.360174 | 4.12E-08 |
| SFXN1 | | 0.328313 | 0.226111 | 0.18009 | 0.274886 | 0.18988 | 4.15E-08 |
| ADAT1 | | 0.345285 | 0.19374 | 0.152503 | 0.238187 | 0.159956 | 4.24E-08 |
| CANT1 | | 0.259492 | 0.239987 | 0.200481 | 0.307988 | 0.214363 | 4.25E-08 |
| RPA2 | | 0.331818 | 0.212554 | 0.168882 | 0.273207 | 0.188792 | 4.37E-08 |
| KCNAB2 | | 0.246519 | 0.44239 | 0.372903 | 0.460302 | 0.347116 | 4.37E-08 |
| AVEN | | 0.48365 | 0.125558 | 0.089795 | 0.179659 | 0.114799 | 4.39E-08 |
| DNPH1 | | 0.41249 | 0.163112 | 0.12255 | 0.216599 | 0.144723 | 4.39E-08 |
| CLEC2B | | 0.274267 | 0.550045 | 0.454816 | 0.509235 | 0.400435 | 4.41E-08 |
| RNF149 | | 0.265006 | 0.366366 | 0.304888 | 0.416887 | 0.31012 | 4.59E-08 |
| RHOC | | 0.392173 | 0.290148 | 0.221087 | 0.266731 | 0.187704 | 4.63E-08 |
| YWHAH | | 0.457809 | 0.139296 | 0.101421 | 0.194291 | 0.126224 | 4.68E-08 |
| TAF2 | | 0.271701 | 0.279045 | 0.231145 | 0.335572 | 0.240479 | 4.68E-08 |
| DUSP7 | | 0.471285 | 0.094025 | 0.067822 | 0.145838 | 0.087051 | 4.91E-08 |
| CLTC | | 0.460801 | 0.093147 | 0.067679 | 0.153994 | 0.09358 | 4.93E-08 |
| VASH1 | | 1.163702 | 0.077151 | 0.034438 | 0.075318 | 0.03482 | 4.94E-08 |
| CNOT1 | | 0.491989 | 0.121408 | 0.086327 | 0.169345 | 0.106638 | 4.98E-08 |
| LRRC8C | | 0.367945 | 0.180738 | 0.140051 | 0.236508 | 0.159956 | 4.99E-08 |
| SEC61B | | 0.351712 | 0.349874 | 0.27418 | 0.348285 | 0.261153 | 5.28E-08 |
| WDR45 | | 0.356125 | 0.179468 | 0.140211 | 0.242984 | 0.164853 | 5.28E-08 |
| NABP1 | | 0.446181 | 0.12826 | 0.094141 | 0.17918 | 0.114255 | 5.3E-08 |
| SUMO3 | | 0.231068 | 0.591177 | 0.503685 | 0.558167 | 0.436888 | 5.41E-08 |
| IFI27L1 | | 0.67737 | 0.073204 | 0.045775 | 0.118734 | 0.066921 | 5.51E-08 |
| DAD1 | | 0.45722 | 0.15022 | 0.109418 | 0.205085 | 0.136561 | 5.51E-08 |
| SPRYD3 | | 0.316692 | 0.16039 | 0.128778 | 0.216359 | 0.141458 | 5.51E-08 |
| STX8 | | 0.279552 | 0.207819 | 0.171211 | 0.270329 | 0.185528 | 5.52E-08 |
| NAGPA | | 0.316344 | 0.159397 | 0.128012 | 0.207484 | 0.134929 | 5.65E-08 |
| FAM114A2 | | 0.476489 | 0.098924 | 0.071099 | 0.150636 | 0.09086 | 5.9E-08 |
| HIGD2A | | 0.255687 | 0.562204 | 0.470895 | 0.557208 | 0.439064 | 5.93E-08 |
| YIPF5 | | 0.347478 | 0.225218 | 0.177012 | 0.276325 | 0.192601 | 6E-08 |
| COX5B | | 0.419618 | 0.098615 | 0.073727 | 0.153514 | 0.093036 | 6.1E-08 |
| METTL2A | | 0.431324 | 0.123498 | 0.091584 | 0.180379 | 0.115343 | 6.13E-08 |
| CNTNAP1 | | 1.145724 | 0.030019 | 0.013568 | 0.05445 | 0.020131 | 6.14E-08 |
| BTN3A1 | | 0.284658 | 0.268132 | 0.220119 | 0.324059 | 0.232862 | 6.16E-08 |
| LAP3 | | 0.321493 | 0.3182 | 0.254637 | 0.360038 | 0.265506 | 6.27E-08 |
| CDC42SE2 | | 0.233322 | 0.754666 | 0.641975 | 0.665387 | 0.552231 | 6.47E-08 |
| EYA3 | | 0.323951 | 0.166404 | 0.132937 | 0.228832 | 0.152884 | 6.48E-08 |
| TMEM214 | | 0.424348 | 0.193563 | 0.144238 | 0.237227 | 0.163221 | 6.48E-08 |
| SCAMP2 | | 0.229302 | 0.699184 | 0.596437 | 0.62581 | 0.505985 | 6.57E-08 |
| SMAGP | | 0.517431 | 0.18569 | 0.129726 | 0.190933 | 0.12568 | 6.77E-08 |
| ITGAM | | 0.655801 | 0.111475 | 0.070756 | 0.126889 | 0.073449 | 7E-08 |
| TXNDC17 | | 0.35089 | 0.195207 | 0.153062 | 0.247062 | 0.168662 | 7E-08 |
| HSPA9 | | 0.347134 | 0.155558 | 0.122291 | 0.221636 | 0.147443 | 7E-08 |
| TOMM5 | | 0.289315 | 0.289036 | 0.236516 | 0.343967 | 0.250272 | 7.21E-08 |
| RASGRP3 | | 0.270322 | 0.317014 | 0.262847 | 0.355961 | 0.258433 | 7.27E-08 |
| AP1S1 | | 0.496651 | 0.0773 | 0.054786 | 0.121852 | 0.068553 | 7.28E-08 |
| ZDHHC12 | | 0.342864 | 0.154773 | 0.122035 | 0.216599 | 0.14309 | 7.28E-08 |
| AKT1 | | 0.255363 | 0.319981 | 0.268072 | 0.378508 | 0.276387 | 7.32E-08 |
| CASP8 | | 0.227915 | 0.55997 | 0.47814 | 0.541617 | 0.428183 | 7.39E-08 |
| LASP1 | | 0.214579 | 0.730347 | 0.629411 | 0.648837 | 0.542982 | 7.46E-08 |
| BCL2L12 | | 0.536469 | 0.10031 | 0.069159 | 0.144159 | 0.087051 | 7.47E-08 |
| SMS | | 0.379523 | 0.228631 | 0.175746 | 0.275846 | 0.196953 | 7.66E-08 |
| KDSR | | 0.419069 | 0.133356 | 0.099738 | 0.177261 | 0.113166 | 7.7E-08 |
| ARFGAP3 | | 0.365891 | 0.23853 | 0.185097 | 0.278724 | 0.198585 | 7.94E-08 |
| EIF3G | | 0.253852 | 0.29085 | 0.243923 | 0.359799 | 0.260609 | 8.04E-08 |
| COMMD4 | | 0.396463 | 0.15358 | 0.116677 | 0.210602 | 0.139826 | 8.31E-08 |
| TNRC6A | | 0.239703 | 0.369481 | 0.312921 | 0.422164 | 0.316104 | 8.33E-08 |
| TMEM185A | | 0.34647 | 0.222265 | 0.174813 | 0.24994 | 0.17247 | 8.43E-08 |
| PLBD2 | | 0.442374 | 0.111923 | 0.082367 | 0.157592 | 0.096844 | 8.51E-08 |
| CPEB4 | | 0.374854 | 0.257773 | 0.19879 | 0.289998 | 0.208923 | 8.6E-08 |
| SESN3 | | 0.297098 | 0.579549 | 0.471688 | 0.486448 | 0.395539 | 8.73E-08 |
| TMEM154 | | 0.203753 | 0.772161 | 0.670459 | 0.650756 | 0.542437 | 8.8E-08 |
| POLE4 | | 0.346198 | 0.18079 | 0.142219 | 0.233389 | 0.158324 | 8.91E-08 |
| WDFY1 | | 0.304941 | 0.273679 | 0.221537 | 0.324298 | 0.235582 | 8.95E-08 |
| ACAA1 | | 0.250028 | 0.401729 | 0.337806 | 0.434637 | 0.328618 | 8.99E-08 |
| DR1 | | 0.260114 | 0.377343 | 0.31509 | 0.430319 | 0.320457 | 9.02E-08 |
| VDAC2 | | 0.276518 | 0.255551 | 0.210978 | 0.329815 | 0.235038 | 9.21E-08 |
| PLAAT4 | | 0.284167 | 0.390026 | 0.320296 | 0.366515 | 0.274755 | 9.22E-08 |
| SERPINB8 | | 0.383366 | 0.239726 | 0.183785 | 0.248501 | 0.173014 | 9.28E-08 |
| ALKBH7 | | 0.281629 | 0.3229 | 0.265638 | 0.366035 | 0.269859 | 9.37E-08 |
| IGHG3 | | 0.398707 | 0.196009 | 0.14868 | 0.142 | 0.086507 | 9.42E-08 |
| SLC37A2 | | 1.965833 | 0.026676 | 0.006829 | 0.033581 | 0.007617 | 9.5E-08 |
| MORF4L2 | | 0.476233 | 0.119668 | 0.086023 | 0.180859 | 0.116975 | 9.5E-08 |
| PNKD | | 0.310991 | 0.195776 | 0.157812 | 0.25018 | 0.173014 | 9.66E-08 |
| LEPROTL1 | | 0.238456 | 0.280121 | 0.237445 | 0.337011 | 0.241567 | 9.72E-08 |
| IBA57 | | 0.497298 | 0.133972 | 0.09491 | 0.167906 | 0.106638 | 9.99E-08 |
| NIPAL3 | | 0.411425 | 0.174773 | 0.131408 | 0.221876 | 0.151795 | 9.99E-08 |
| GLT8D1 | | 0.390085 | 0.115028 | 0.087776 | 0.172943 | 0.109902 | 1.01E-07 |
| INTS14 | | 0.377893 | 0.143845 | 0.110697 | 0.195491 | 0.127312 | 1.01E-07 |
| UBA3 | | 0.240282 | 0.385639 | 0.326474 | 0.42936 | 0.321001 | 1.02E-07 |
| GORASP2 | | 0.320529 | 0.326127 | 0.261155 | 0.35644 | 0.264418 | 1.02E-07 |
| BMF | | 0.36365 | 0.136393 | 0.106004 | 0.171744 | 0.107726 | 1.06E-07 |
| ACADM | | 0.277112 | 0.341474 | 0.281799 | 0.386184 | 0.287813 | 1.06E-07 |
| ATG3 | | 0.279319 | 0.352263 | 0.290258 | 0.402255 | 0.299238 | 1.07E-07 |
| METTL25B | | 0.375377 | 0.174152 | 0.134255 | 0.21396 | 0.14309 | 1.07E-07 |
| NPEPPS | | 0.21821 | 0.434884 | 0.37384 | 0.478292 | 0.361806 | 1.08E-07 |
| PIGX | | 0.422092 | 0.149831 | 0.111825 | 0.193572 | 0.127312 | 1.08E-07 |
| GMFG | | 0.256358 | 0.477562 | 0.399814 | 0.501559 | 0.383569 | 1.08E-07 |
| KIF21B | | 0.268417 | 0.331769 | 0.275445 | 0.371312 | 0.274755 | 1.08E-07 |
| CASK | | 0.323972 | 0.309911 | 0.247578 | 0.317342 | 0.228509 | 1.09E-07 |
| LPXN | | 0.214104 | 0.725176 | 0.625161 | 0.630367 | 0.512514 | 1.1E-07 |
| NT5C3B | | 0.600032 | 0.099863 | 0.065883 | 0.123531 | 0.071273 | 1.13E-07 |
| CALCOCO1 | | 0.279602 | 0.309595 | 0.25505 | 0.348765 | 0.257345 | 1.14E-07 |
| RBFA | | 0.395824 | 0.166643 | 0.126658 | 0.21516 | 0.144723 | 1.15E-07 |
| SYK | | 0.142217 | 1.352461 | 1.225499 | 0.859679 | 0.795974 | 1.15E-07 |
| SLC35B2 | | 0.303972 | 0.275203 | 0.22292 | 0.319981 | 0.23395 | 1.15E-07 |
| MADD | | 0.264665 | 0.371367 | 0.309123 | 0.409451 | 0.307943 | 1.19E-07 |
| DTYMK | | 0.437975 | 0.122889 | 0.090713 | 0.165507 | 0.104461 | 1.22E-07 |
| INSIG2 | | 0.342799 | 0.154367 | 0.12172 | 0.211082 | 0.14037 | 1.23E-07 |
| CNP | | 0.292315 | 0.323853 | 0.264455 | 0.368674 | 0.272579 | 1.24E-07 |
| SLC2A11 | | 0.405085 | 0.175128 | 0.132255 | 0.211322 | 0.142002 | 1.25E-07 |
| CDKN1B | | 0.174444 | 1.181428 | 1.046874 | 0.814824 | 0.718716 | 1.25E-07 |
| ZDHHC21 | | 0.289663 | 0.290417 | 0.237588 | 0.331494 | 0.241023 | 1.27E-07 |
| DNTTIP2 | | 0.220421 | 0.546399 | 0.468983 | 0.554569 | 0.436344 | 1.27E-07 |
| SLC35B1 | | 0.38421 | 0.27784 | 0.212881 | 0.277045 | 0.199674 | 1.27E-07 |
| PGPEP1 | | 0.344367 | 0.168703 | 0.13288 | 0.2142 | 0.14309 | 1.29E-07 |
| MPC1 | | 0.363937 | 0.216312 | 0.168083 | 0.256177 | 0.180087 | 1.31E-07 |
| SCAND1 | | 0.253002 | 0.368222 | 0.308993 | 0.422883 | 0.317737 | 1.31E-07 |
| VDAC1 | | 0.326268 | 0.174394 | 0.139096 | 0.241785 | 0.164853 | 1.34E-07 |
| PATL2 | | 0.488229 | 0.134251 | 0.095708 | 0.16023 | 0.100109 | 1.35E-07 |
| HDAC3 | | 0.322502 | 0.184837 | 0.147811 | 0.247781 | 0.170838 | 1.35E-07 |
| ATP1A1 | | 0.297805 | 0.139459 | 0.113448 | 0.210362 | 0.139282 | 1.37E-07 |
| HIVEP2 | | 0.254476 | 0.302522 | 0.253602 | 0.348765 | 0.255169 | 1.39E-07 |
| ZNF207 | | 0.27771 | 0.231424 | 0.190901 | 0.299832 | 0.213275 | 1.4E-07 |
| NFKB1 | | 0.265741 | 0.35732 | 0.297208 | 0.401775 | 0.302503 | 1.42E-07 |
| SLC16A1 | | 0.352648 | 0.10387 | 0.081345 | 0.155913 | 0.095756 | 1.51E-07 |
| MGAT4B | | 0.304522 | 0.266645 | 0.215905 | 0.307268 | 0.223613 | 1.51E-07 |
| SEMA7A | | 0.394236 | 0.215723 | 0.164142 | 0.225953 | 0.153972 | 1.56E-07 |
| RNF113A | | 0.289001 | 0.241261 | 0.197465 | 0.296474 | 0.212187 | 1.56E-07 |
| TXNDC11 | | 0.290513 | 0.666389 | 0.544847 | 0.49988 | 0.406964 | 1.6E-07 |
| FASTKD1 | | 0.398565 | 0.100524 | 0.076259 | 0.147997 | 0.090316 | 1.61E-07 |
| SNX9 | | 0.384147 | 0.279726 | 0.214335 | 0.287839 | 0.208379 | 1.63E-07 |
| IER3IP1 | | 0.332677 | 0.20632 | 0.163831 | 0.26697 | 0.187704 | 1.63E-07 |
| ECH1 | | 0.21183 | 0.825218 | 0.712527 | 0.671624 | 0.559848 | 1.74E-07 |
| CHPF2 | | 0.255026 | 0.321069 | 0.269047 | 0.371072 | 0.274755 | 1.75E-07 |
| LY96 | | 0.443897 | 0.112902 | 0.082999 | 0.154473 | 0.095756 | 1.75E-07 |
| YIPF2 | | 0.524336 | 0.088215 | 0.061334 | 0.140801 | 0.085963 | 1.77E-07 |
| PCYT1A | | 0.301123 | 0.202804 | 0.1646 | 0.264572 | 0.183351 | 1.79E-07 |
| QDPR | | 0.392732 | 0.112255 | 0.085503 | 0.165747 | 0.105005 | 1.8E-07 |
| HM13 | | 0.330477 | 0.544501 | 0.433027 | 0.438235 | 0.350925 | 1.8E-07 |
| TMEM140 | | 0.286545 | 0.27675 | 0.226897 | 0.301991 | 0.217628 | 1.81E-07 |
| ANXA7 | | 0.293489 | 0.275295 | 0.224621 | 0.337251 | 0.246464 | 1.89E-07 |
| LGMN | | 0.479272 | 0.108738 | 0.078002 | 0.147517 | 0.09086 | 1.9E-07 |
| CFAP298 | | 0.307392 | 0.27336 | 0.220902 | 0.331974 | 0.242655 | 1.92E-07 |
| RNASEH2C | | 0.283797 | 0.246441 | 0.202433 | 0.300072 | 0.213819 | 1.95E-07 |
| GTF2H1 | | 0.249586 | 0.28187 | 0.237091 | 0.324778 | 0.235038 | 1.98E-07 |
| TATDN2 | | 0.2439 | 0.399134 | 0.337053 | 0.44687 | 0.340044 | 1.98E-07 |
| ARID3B | | 0.305687 | 0.187211 | 0.151465 | 0.247062 | 0.170838 | 1.98E-07 |
| FAM162A | | 0.273058 | 0.220932 | 0.182835 | 0.274406 | 0.190968 | 1.99E-07 |
| KIF3B | | 0.25891 | 0.270733 | 0.226256 | 0.318302 | 0.229053 | 2E-07 |
| ZNF622 | | 0.261881 | 0.291537 | 0.243142 | 0.351403 | 0.256257 | 2E-07 |
| TMEM238 | | 0.495056 | 0.093563 | 0.066386 | 0.144639 | 0.089227 | 2.01E-07 |
| SFXN4 | | 0.460921 | 0.066912 | 0.048613 | 0.118734 | 0.068009 | 2.01E-07 |
| ATP6V1B2 | | 0.299321 | 0.21036 | 0.170946 | 0.270329 | 0.190424 | 2.01E-07 |
| SRM | | 0.294566 | 0.507627 | 0.413877 | 0.444471 | 0.351469 | 2.03E-07 |
| FAM98A | | 0.307696 | 0.180273 | 0.145648 | 0.229072 | 0.155604 | 2.05E-07 |
| ADI1 | | 0.257799 | 0.397021 | 0.332053 | 0.403214 | 0.304679 | 2.07E-07 |
| APOL2 | | 0.31568 | 0.212892 | 0.171052 | 0.264812 | 0.187704 | 2.09E-07 |
| CTSC | | 0.339885 | 0.260917 | 0.206152 | 0.309906 | 0.227421 | 2.11E-07 |
| ECI1 | | 0.318583 | 0.230815 | 0.185081 | 0.283041 | 0.201306 | 2.15E-07 |
| COX11 | | 0.283013 | 0.223105 | 0.183364 | 0.276085 | 0.194233 | 2.16E-07 |
| DNAJB12 | | 0.245053 | 0.316414 | 0.266985 | 0.367234 | 0.269859 | 2.18E-07 |
| PPP1R11 | | 0.253875 | 0.351631 | 0.294892 | 0.406812 | 0.306311 | 2.2E-07 |
| WAPL | | 0.29234 | 0.226327 | 0.184813 | 0.287119 | 0.205114 | 2.25E-07 |
| ARF5 | | 0.252439 | 0.320659 | 0.269186 | 0.381626 | 0.284548 | 2.26E-07 |
| GIGYF2 | | 0.261402 | 0.173278 | 0.144562 | 0.236268 | 0.160501 | 2.28E-07 |
| PSMD2 | | 0.297707 | 0.166258 | 0.135258 | 0.235548 | 0.160501 | 2.35E-07 |
| PRMT1 | | 0.2461 | 0.495927 | 0.418152 | 0.491005 | 0.386834 | 2.35E-07 |
| ZNF281 | | 0.258762 | 0.200143 | 0.16728 | 0.252818 | 0.174102 | 2.43E-07 |
| TRAPPC2L | | 0.31667 | 0.163123 | 0.130975 | 0.226673 | 0.153972 | 2.43E-07 |
| LYRM1 | | 0.302713 | 0.191318 | 0.155107 | 0.238426 | 0.163765 | 2.49E-07 |
| PCED1B | | 0.385578 | 0.182304 | 0.139549 | 0.222595 | 0.153428 | 2.53E-07 |
| KMT5A | | 0.277346 | 0.256999 | 0.212052 | 0.307748 | 0.223069 | 2.54E-07 |
| RHOT1 | | 0.223247 | 0.251255 | 0.215234 | 0.304629 | 0.215996 | 2.62E-07 |
| STX7 | | 0.132799 | 1.260778 | 1.149905 | 0.848645 | 0.787269 | 2.64E-07 |
| EPM2AIP1 | | 0.279073 | 0.287603 | 0.237019 | 0.333653 | 0.245375 | 2.66E-07 |
| S100A6 | | 0.215478 | 0.3971 | 0.342007 | 0.419285 | 0.311208 | 2.66E-07 |
| SELL | | 0.162404 | 1.79857 | 1.607085 | 0.870712 | 0.762786 | 2.73E-07 |
| MOV10 | | 0.42127 | 0.191723 | 0.143172 | 0.206524 | 0.139282 | 2.76E-07 |
| TTLL3 | | 0.340987 | 0.265969 | 0.209983 | 0.284001 | 0.205114 | 2.79E-07 |
| ADCY3 | | 0.457768 | 0.14021 | 0.102088 | 0.177021 | 0.115343 | 2.8E-07 |
| DDX19B | | 0.359436 | 0.145944 | 0.113759 | 0.209163 | 0.141458 | 2.84E-07 |
| BRI3BP | | 0.277654 | 0.310239 | 0.255926 | 0.350684 | 0.260065 | 2.84E-07 |
| SNAI3 | | 0.554831 | 0.099852 | 0.067973 | 0.130967 | 0.07889 | 2.86E-07 |
| SWSAP1 | | 0.484574 | 0.093214 | 0.066621 | 0.12425 | 0.072361 | 2.92E-07 |
| PFKM | | 0.451914 | 0.126945 | 0.092806 | 0.155193 | 0.097933 | 2.92E-07 |
| ILVBL | | 0.428229 | 0.124996 | 0.092894 | 0.175342 | 0.114255 | 2.96E-07 |
| CLIC1 | | 0.254586 | 0.482431 | 0.404387 | 0.489086 | 0.386289 | 2.98E-07 |
| KLHL18 | | 0.513936 | 0.104313 | 0.073052 | 0.140561 | 0.085963 | 2.99E-07 |
| TXN2 | | 0.282651 | 0.177608 | 0.146008 | 0.241785 | 0.166485 | 2.99E-07 |
| CREM | | 0.328202 | 0.320833 | 0.255552 | 0.335092 | 0.252992 | 3.08E-07 |
| SEC61A1 | | 0.341461 | 0.472558 | 0.372962 | 0.407292 | 0.325354 | 3.2E-07 |
| RAB33A | | 1.836195 | 0.02365 | 0.006623 | 0.033821 | 0.008705 | 3.2E-07 |
| TRIO | | 0.237477 | 0.581724 | 0.493434 | 0.552411 | 0.446681 | 3.22E-07 |
| CCDC163 | | 1.942566 | 0.016921 | 0.004402 | 0.037419 | 0.010881 | 3.23E-07 |
| DUSP5 | | 0.320841 | 0.463905 | 0.371404 | 0.415687 | 0.335147 | 3.26E-07 |
| RHOH | | 0.166245 | 1.241035 | 1.10596 | 0.830655 | 0.739391 | 3.31E-07 |
| RAB3D | | 0.511785 | 0.079107 | 0.055482 | 0.122092 | 0.071817 | 3.31E-07 |
| OTUD1 | | 0.213252 | 0.846607 | 0.730275 | 0.64308 | 0.548422 | 3.33E-07 |
| LRRC8B | | 0.387607 | 0.120399 | 0.092033 | 0.173183 | 0.112078 | 3.36E-07 |
| MGST3 | | 0.347904 | 0.170058 | 0.133618 | 0.219477 | 0.149619 | 3.4E-07 |
| LSG1 | | 0.2606 | 0.28914 | 0.241357 | 0.349005 | 0.255169 | 3.41E-07 |
| TRPV2 | | 0.239283 | 0.302919 | 0.256623 | 0.359799 | 0.264418 | 3.41E-07 |
| CD1D | | 0.391776 | 0.22585 | 0.17214 | 0.234828 | 0.164309 | 3.45E-07 |
| BROX | | 0.277207 | 0.269534 | 0.222416 | 0.309427 | 0.225789 | 3.45E-07 |
| LRRC41 | | 0.25375 | 0.306302 | 0.2569 | 0.352123 | 0.257345 | 3.46E-07 |
| CERT1 | | 0.211538 | 0.561206 | 0.484666 | 0.548093 | 0.440152 | 3.48E-07 |
| GINM1 | | 0.26513 | 0.279298 | 0.232411 | 0.337251 | 0.247008 | 3.72E-07 |
| MTFP1 | | 0.400402 | 0.097025 | 0.073511 | 0.146318 | 0.09086 | 3.72E-07 |
| HCFC1R1 | | 0.309203 | 0.154758 | 0.124903 | 0.209882 | 0.141458 | 3.72E-07 |
| ZNF101 | | 0.271106 | 0.262138 | 0.21723 | 0.300312 | 0.217084 | 3.72E-07 |
| CIAO2B | | 0.298295 | 0.178396 | 0.145074 | 0.243704 | 0.168662 | 3.73E-07 |
| SCAF1 | | 0.244221 | 0.295489 | 0.249473 | 0.360278 | 0.26605 | 3.74E-07 |
| TMEM39A | | 0.304933 | 0.14347 | 0.116136 | 0.202686 | 0.134929 | 3.8E-07 |
| PIP4K2C | | 0.412471 | 0.111678 | 0.083908 | 0.164788 | 0.106094 | 3.81E-07 |
| BAG2 | | 0.310639 | 0.109185 | 0.088034 | 0.155913 | 0.097388 | 3.83E-07 |
| MBD4 | | 0.237751 | 0.564384 | 0.478635 | 0.549292 | 0.445049 | 3.87E-07 |
| RPP25L | | 0.398527 | 0.12202 | 0.092568 | 0.168865 | 0.108814 | 3.87E-07 |
| SELENOS | | 0.359356 | 0.301835 | 0.235284 | 0.287839 | 0.211099 | 3.9E-07 |
| IFIT5 | | 0.332501 | 0.16508 | 0.1311 | 0.197889 | 0.130577 | 3.92E-07 |
| GDPD5 | | 1.27746 | 0.040072 | 0.016531 | 0.051571 | 0.020131 | 3.94E-07 |
| PNPO | | 0.352426 | 0.13546 | 0.106101 | 0.190933 | 0.126768 | 3.95E-07 |
| TCHP | | 0.327509 | 0.164936 | 0.131439 | 0.216599 | 0.146899 | 3.98E-07 |
| PHKB | | 0.232365 | 0.465728 | 0.396446 | 0.491725 | 0.385745 | 4.05E-07 |
| APMAP | | 0.337764 | 0.147933 | 0.117054 | 0.210842 | 0.143634 | 4.06E-07 |
| MLLT1 | | 0.259819 | 0.271952 | 0.227132 | 0.330535 | 0.240479 | 4.09E-07 |
| RFC1 | | 0.263096 | 0.217022 | 0.180844 | 0.284241 | 0.20185 | 4.11E-07 |
| VAMP1 | | 0.36028 | 0.140462 | 0.109422 | 0.193092 | 0.128945 | 4.11E-07 |
| MCM3 | | 0.324801 | 0.252696 | 0.201755 | 0.283761 | 0.20457 | 4.15E-07 |
| BDH2 | | 0.529792 | 0.106754 | 0.073943 | 0.131207 | 0.079434 | 4.18E-07 |
| NAPSA | | 1.133953 | 0.020518 | 0.009349 | 0.039098 | 0.01197 | 4.19E-07 |
| PLEKHO2 | | 0.333115 | 0.185315 | 0.147107 | 0.224035 | 0.153428 | 4.19E-07 |
| INPP4A | | 0.293706 | 0.221376 | 0.180599 | 0.275126 | 0.195321 | 4.2E-07 |
| PSMC4 | | 0.278829 | 0.24079 | 0.198474 | 0.305829 | 0.220348 | 4.2E-07 |
| LINS1 | | 0.272796 | 0.181444 | 0.150183 | 0.230991 | 0.158324 | 4.22E-07 |
| SLC39A7 | | 0.330507 | 0.303095 | 0.241038 | 0.311346 | 0.232318 | 4.23E-07 |
| LDHB | | 0.223872 | 0.658955 | 0.564239 | 0.568961 | 0.463003 | 4.32E-07 |
| HMGCL | | 0.371599 | 0.169066 | 0.130675 | 0.208443 | 0.142002 | 4.37E-07 |
| FOXRED1 | | 0.480492 | 0.116702 | 0.083644 | 0.161909 | 0.103917 | 4.37E-07 |
| PRKCD | | 0.243226 | 0.360099 | 0.304231 | 0.399136 | 0.301959 | 4.37E-07 |
| NKIRAS2 | | 0.372839 | 0.157215 | 0.121411 | 0.21396 | 0.147443 | 4.4E-07 |
| POLDIP2 | | 0.234763 | 0.413868 | 0.351715 | 0.441353 | 0.332971 | 4.4E-07 |
| ARF4 | | 0.295152 | 0.367693 | 0.299665 | 0.391941 | 0.303047 | 4.42E-07 |
| PLXNA3 | | 0.286701 | 0.288699 | 0.236668 | 0.316863 | 0.23123 | 4.43E-07 |
| NAGA | | 0.367047 | 0.099015 | 0.076773 | 0.156632 | 0.099021 | 4.44E-07 |
| ANTKMT | | 0.30146 | 0.151908 | 0.123263 | 0.209643 | 0.142002 | 4.44E-07 |
| DNAAF10 | | 0.443716 | 0.094549 | 0.069516 | 0.133365 | 0.080522 | 4.45E-07 |
| LCMT1 | | 0.24414 | 0.157145 | 0.132681 | 0.216839 | 0.146355 | 4.46E-07 |
| EBAG9 | | 0.243903 | 0.217564 | 0.183724 | 0.274166 | 0.193689 | 4.46E-07 |
| UBXN6 | | 0.208126 | 0.505332 | 0.437446 | 0.520748 | 0.401523 | 4.51E-07 |
| ICMT | | 0.362356 | 0.12755 | 0.09922 | 0.17918 | 0.117519 | 4.56E-07 |
| B4GALT3 | | 0.261668 | 0.294764 | 0.245869 | 0.329096 | 0.238847 | 4.56E-07 |
| FDX1 | | 0.352035 | 0.165327 | 0.12953 | 0.226913 | 0.157236 | 4.6E-07 |
| VRK1 | | 0.273377 | 0.269088 | 0.222638 | 0.313744 | 0.229597 | 4.93E-07 |
| UBN1 | | 0.307883 | 0.188914 | 0.15261 | 0.251379 | 0.177367 | 4.94E-07 |
| ASCC1 | | 0.341854 | 0.17619 | 0.139018 | 0.218997 | 0.150707 | 4.98E-07 |
| TPGS2 | | 0.281723 | 0.21388 | 0.17594 | 0.261693 | 0.184984 | 5.02E-07 |
| SUPV3L1 | | 0.383731 | 0.158568 | 0.121535 | 0.205325 | 0.139282 | 5.06E-07 |
| METTL6 | | 0.412012 | 0.133899 | 0.100635 | 0.175342 | 0.115343 | 5.15E-07 |
| MDM1 | | 0.361654 | 0.180545 | 0.140513 | 0.221636 | 0.153428 | 5.15E-07 |
| TET2 | | 0.259415 | 0.191562 | 0.160036 | 0.242024 | 0.166485 | 5.17E-07 |
| TRAPPC1 | | 0.257677 | 0.363895 | 0.304374 | 0.421684 | 0.323721 | 5.17E-07 |
| RNF8 | | 0.266352 | 0.155861 | 0.129586 | 0.206524 | 0.138194 | 5.18E-07 |
| KLHL5 | | 0.209148 | 0.552045 | 0.477545 | 0.538978 | 0.423286 | 5.18E-07 |
| VPS35L | | 0.270847 | 0.294725 | 0.244278 | 0.342528 | 0.252448 | 5.2E-07 |
| UQCRC1 | | 0.24529 | 0.386227 | 0.325839 | 0.418326 | 0.317193 | 5.45E-07 |
| UBR4 | | 0.20435 | 0.521189 | 0.452355 | 0.540178 | 0.420022 | 5.49E-07 |
| CHUK | | 0.359199 | 0.126546 | 0.098655 | 0.181099 | 0.119695 | 5.51E-07 |
| INPP5B | | 0.240707 | 0.297506 | 0.251788 | 0.343728 | 0.25136 | 5.56E-07 |
| RAB5IF | | 0.285884 | 0.244599 | 0.200629 | 0.319021 | 0.23395 | 5.66E-07 |
| TRAF3IP3 | | 0.186788 | 0.890438 | 0.782303 | 0.703526 | 0.591948 | 5.67E-07 |
| REV3L | | 0.338848 | 0.163551 | 0.129316 | 0.205565 | 0.139282 | 5.71E-07 |
| FLNA | | 0.188156 | 0.861821 | 0.756443 | 0.708083 | 0.603373 | 5.87E-07 |
| PREB | | 0.401232 | 0.240408 | 0.18204 | 0.260494 | 0.191513 | 5.93E-07 |
| PITPNA | | 0.233277 | 0.375913 | 0.31979 | 0.423843 | 0.321001 | 5.95E-07 |
| SPTSSA | | 0.282652 | 0.286643 | 0.235643 | 0.32214 | 0.238847 | 5.95E-07 |
| TIMM17B | | 0.274281 | 0.244163 | 0.201889 | 0.30343 | 0.219804 | 6.06E-07 |
| ATP2B1 | | 0.19487 | 0.730791 | 0.638456 | 0.637323 | 0.530468 | 6.08E-07 |
| EED | | 0.311473 | 0.187142 | 0.150803 | 0.243943 | 0.170294 | 6.21E-07 |
| CUEDC2 | | 0.274553 | 0.261952 | 0.216557 | 0.320221 | 0.236126 | 6.23E-07 |
| YRDC | | 0.286251 | 0.244279 | 0.200316 | 0.30391 | 0.221436 | 6.26E-07 |
| TMA16 | | 0.310859 | 0.168227 | 0.135618 | 0.220676 | 0.151795 | 6.41E-07 |
| MAVS | | 0.227885 | 0.325306 | 0.277774 | 0.379467 | 0.28346 | 6.43E-07 |
| FKBP8 | | 0.257964 | 0.280375 | 0.234468 | 0.351403 | 0.258977 | 6.49E-07 |
| STAT5A | | 0.294691 | 0.235687 | 0.192143 | 0.263852 | 0.186616 | 6.65E-07 |
| SIGIRR | | 0.329712 | 0.210865 | 0.167784 | 0.238906 | 0.165941 | 6.69E-07 |
| ZPR1 | | 0.251874 | 0.215662 | 0.181114 | 0.278004 | 0.197497 | 6.76E-07 |
| GPX7 | | 0.296297 | 0.221363 | 0.180265 | 0.278964 | 0.201306 | 6.9E-07 |
| LANCL2 | | 0.480413 | 0.092763 | 0.06649 | 0.135044 | 0.083243 | 7.11E-07 |
| PACSIN2 | | 0.249963 | 0.343949 | 0.289233 | 0.37539 | 0.284548 | 7.11E-07 |
| CHCHD7 | | 0.248103 | 0.300213 | 0.252781 | 0.354042 | 0.26333 | 7.11E-07 |
| NDUFS1 | | 0.252634 | 0.32309 | 0.27119 | 0.371312 | 0.27802 | 7.15E-07 |
| RABGGTA | | 0.309703 | 0.161575 | 0.13036 | 0.215639 | 0.147987 | 7.27E-07 |
| MAPK8IP3 | | 0.262199 | 0.343243 | 0.286201 | 0.370832 | 0.280196 | 7.3E-07 |
| SPNS1 | | 0.28805 | 0.169541 | 0.138855 | 0.230511 | 0.159956 | 7.34E-07 |
| SLC25A20 | | 0.354883 | 0.108819 | 0.085089 | 0.159511 | 0.102829 | 7.34E-07 |
| SLC33A1 | | 0.28281 | 0.21863 | 0.179712 | 0.278004 | 0.199129 | 7.34E-07 |
| CEMIP2 | | 0.326531 | 0.38933 | 0.310472 | 0.343008 | 0.261697 | 7.35E-07 |
| ACAT1 | | 0.269132 | 0.190171 | 0.157807 | 0.241305 | 0.168118 | 7.36E-07 |
| ALG1 | | 0.289181 | 0.163773 | 0.134027 | 0.218757 | 0.149075 | 7.42E-07 |
| SLC4A1AP | | 0.264754 | 0.185314 | 0.154244 | 0.25066 | 0.175734 | 7.45E-07 |
| IL12RB1 | | 0.470389 | 0.0997 | 0.07196 | 0.150876 | 0.096844 | 7.49E-07 |
| EFHD2 | | 0.189589 | 0.627822 | 0.550509 | 0.600384 | 0.483678 | 7.59E-07 |
| N4BP2L1 | | 0.258501 | 0.242716 | 0.2029 | 0.284241 | 0.202938 | 7.79E-07 |
| MCM3AP | | 0.257206 | 0.305324 | 0.255467 | 0.360998 | 0.269314 | 7.89E-07 |
| SIAE | | 0.450264 | 0.093782 | 0.06864 | 0.126649 | 0.07617 | 8.19E-07 |
| GBA1 | | 0.336176 | 0.145297 | 0.115095 | 0.203886 | 0.138738 | 8.23E-07 |
| PGRMC1 | | 0.449392 | 0.087863 | 0.064347 | 0.134325 | 0.082699 | 8.26E-07 |
| HEXB | | 0.439363 | 0.0974 | 0.071829 | 0.148477 | 0.094668 | 8.26E-07 |
| NDUFB10 | | 0.235413 | 0.588261 | 0.499693 | 0.542336 | 0.439064 | 8.29E-07 |
| ST8SIA4 | | 0.194894 | 0.749876 | 0.65512 | 0.650995 | 0.54951 | 8.3E-07 |
| NDUFV2 | | 0.221485 | 0.637885 | 0.547102 | 0.584313 | 0.468988 | 8.32E-07 |
| PLPBP | | 0.261721 | 0.268462 | 0.223923 | 0.311586 | 0.230141 | 8.7E-07 |
| MAN2B2 | | 0.243592 | 0.289671 | 0.244667 | 0.334613 | 0.247008 | 8.74E-07 |
| SIRT2 | | 0.236163 | 0.249943 | 0.212201 | 0.308227 | 0.223613 | 8.82E-07 |
| SLC26A6 | | 1.006981 | 0.027054 | 0.013462 | 0.056608 | 0.024483 | 8.93E-07 |
| CAMKMT | | 0.324624 | 0.170266 | 0.135958 | 0.216599 | 0.149619 | 8.93E-07 |
| MTCH2 | | 0.331202 | 0.108777 | 0.086464 | 0.167186 | 0.10827 | 9.03E-07 |
| CREB3L2 | | 0.306057 | 0.27851 | 0.225273 | 0.319261 | 0.239391 | 9.03E-07 |
| TBK1 | | 0.252643 | 0.19968 | 0.167603 | 0.264812 | 0.187704 | 9.04E-07 |
| C15orf39 | | 0.275025 | 0.313883 | 0.259404 | 0.352603 | 0.264962 | 9.13E-07 |
| PPOX | | 0.359852 | 0.141599 | 0.11034 | 0.189974 | 0.1284 | 9.22E-07 |
| ZNF141 | | 0.271698 | 0.328238 | 0.271894 | 0.344927 | 0.257345 | 9.43E-07 |
| MAGED2 | | 0.261249 | 0.333396 | 0.278174 | 0.358359 | 0.271491 | 9.47E-07 |
| COPS3 | | 0.225812 | 0.336129 | 0.287429 | 0.378748 | 0.282916 | 9.56E-07 |
| CAPN2 | | 0.245051 | 0.32472 | 0.273995 | 0.335332 | 0.244831 | 9.57E-07 |
| IDH2 | | 0.233254 | 0.65464 | 0.556911 | 0.533941 | 0.43852 | 9.59E-07 |
| AGPAT3 | | 0.239412 | 0.206229 | 0.174695 | 0.270089 | 0.191513 | 9.6E-07 |
| HMGN3 | | 0.232734 | 0.391535 | 0.333204 | 0.430079 | 0.329706 | 9.67E-07 |
| ITM2B | | 0.227742 | 0.430939 | 0.368009 | 0.467498 | 0.358542 | 9.78E-07 |
| RIOK2 | | 0.292339 | 0.128508 | 0.104937 | 0.184697 | 0.122416 | 9.81E-07 |
| DESI1 | | 0.265573 | 0.226586 | 0.18849 | 0.280643 | 0.201306 | 9.89E-07 |
| RUBCN | | 0.219668 | 0.475283 | 0.408156 | 0.504438 | 0.399891 | 1E-06 |
| DOK3 | | 0.211079 | 0.32602 | 0.281645 | 0.355961 | 0.262242 | 1.04E-06 |
| UCK2 | | 0.427603 | 0.110542 | 0.082188 | 0.153514 | 0.099021 | 1.06E-06 |
| USP12 | | 0.208477 | 0.516242 | 0.446781 | 0.527944 | 0.413493 | 1.08E-06 |
| COX6A1 | | 0.241511 | 0.451848 | 0.3822 | 0.472775 | 0.368335 | 1.09E-06 |
| ADGRG5 | | 0.242928 | 0.42561 | 0.359652 | 0.431039 | 0.336235 | 1.09E-06 |
| LRWD1 | | 0.385279 | 0.117899 | 0.090267 | 0.163349 | 0.106094 | 1.1E-06 |
| TUBG1 | | 0.456376 | 0.09837 | 0.071694 | 0.136484 | 0.085419 | 1.1E-06 |
| TLK2 | | 0.246476 | 0.253677 | 0.213838 | 0.315183 | 0.231774 | 1.11E-06 |
| DNAJC3 | | 0.357162 | 0.254185 | 0.198442 | 0.264332 | 0.193689 | 1.11E-06 |
| TCF3 | | 0.196114 | 0.816039 | 0.712319 | 0.689134 | 0.574538 | 1.11E-06 |
| TXNRD1 | | 0.254609 | 0.271692 | 0.227736 | 0.326217 | 0.239935 | 1.14E-06 |
| RFTN1 | | 0.195881 | 0.571519 | 0.498959 | 0.532982 | 0.424374 | 1.17E-06 |
| ACIN1 | | 0.246817 | 0.338191 | 0.285012 | 0.399616 | 0.306311 | 1.18E-06 |
| ACAD11 | | 0.270868 | 0.155964 | 0.129266 | 0.206524 | 0.141458 | 1.19E-06 |
| TSTD1 | | 0.210068 | 0.484736 | 0.419052 | 0.492444 | 0.386834 | 1.21E-06 |
| PMPCA | | 0.241865 | 0.217281 | 0.183744 | 0.278724 | 0.199674 | 1.21E-06 |
| MPI | | 0.292018 | 0.154731 | 0.126378 | 0.211082 | 0.145267 | 1.22E-06 |
| PPA1 | | 0.276893 | 0.162508 | 0.134129 | 0.224035 | 0.15506 | 1.22E-06 |
| PEX11B | | 0.253412 | 0.21572 | 0.18097 | 0.269369 | 0.193145 | 1.28E-06 |
| HELLS | | 0.245964 | 0.224729 | 0.189503 | 0.236508 | 0.164309 | 1.31E-06 |
| STK39 | | 0.238842 | 0.296181 | 0.250991 | 0.347805 | 0.257889 | 1.31E-06 |
| SH3GL1 | | 0.253359 | 0.200552 | 0.168251 | 0.251139 | 0.177911 | 1.31E-06 |
| TIMD4 | | 1.603051 | 0.020333 | 0.006693 | 0.042216 | 0.015234 | 1.33E-06 |
| COMMD3 | | 0.230361 | 0.218301 | 0.186085 | 0.279923 | 0.200218 | 1.33E-06 |
| PTPN7 | | 0.324721 | 0.192253 | 0.153505 | 0.234828 | 0.167029 | 1.34E-06 |
| OSBP | | 0.244667 | 0.383118 | 0.323356 | 0.420245 | 0.325898 | 1.34E-06 |
| HAUS4 | | 0.381263 | 0.11602 | 0.089076 | 0.161669 | 0.10555 | 1.34E-06 |
| CHCHD5 | | 0.258111 | 0.163663 | 0.136852 | 0.218278 | 0.150163 | 1.34E-06 |
| LMAN2 | | 0.197642 | 0.937295 | 0.817298 | 0.684097 | 0.611534 | 1.34E-06 |
| ZNF595 | | 0.401006 | 0.160291 | 0.121393 | 0.176061 | 0.117519 | 1.37E-06 |
| TRNAU1AP | | 0.252931 | 0.20891 | 0.175315 | 0.260974 | 0.186616 | 1.37E-06 |
| GMPPA | | 0.413407 | 0.177379 | 0.133185 | 0.215639 | 0.151795 | 1.37E-06 |
| GATC | | 0.305145 | 0.168633 | 0.136485 | 0.220437 | 0.153428 | 1.38E-06 |
| ERAP1 | | 0.24646 | 0.300597 | 0.253392 | 0.358599 | 0.268226 | 1.39E-06 |
| ZFY | | 1.042752 | 0.053115 | 0.025782 | 0.066923 | 0.032644 | 1.39E-06 |
| CTU2 | | 0.353767 | 0.137116 | 0.107298 | 0.181338 | 0.121328 | 1.41E-06 |
| CRKL | | 0.22003 | 0.376192 | 0.322979 | 0.421684 | 0.321001 | 1.41E-06 |
| OGFOD2 | | 0.26214 | 0.196421 | 0.163786 | 0.250899 | 0.177911 | 1.41E-06 |
| NDUFA11 | | 0.266686 | 0.292626 | 0.243238 | 0.35716 | 0.269314 | 1.47E-06 |
| TAMM41 | | 0.478233 | 0.114315 | 0.082062 | 0.151595 | 0.097933 | 1.48E-06 |
| MCOLN2 | | 0.444786 | 0.125977 | 0.092555 | 0.143919 | 0.091404 | 1.48E-06 |
| SLC44A1 | | 0.412896 | 0.257011 | 0.193044 | 0.234589 | 0.170838 | 1.5E-06 |
| ARRB2 | | 0.212396 | 0.480089 | 0.414366 | 0.48213 | 0.380849 | 1.5E-06 |
| SELENOW | | 0.231002 | 0.260772 | 0.222189 | 0.323579 | 0.238303 | 1.5E-06 |
| MIGA2 | | 0.356829 | 0.134149 | 0.104754 | 0.172703 | 0.114255 | 1.52E-06 |
| SLC38A5 | | 0.511323 | 0.141675 | 0.099396 | 0.159751 | 0.106094 | 1.55E-06 |
| SLC10A3 | | 0.298312 | 0.188117 | 0.152977 | 0.243943 | 0.173558 | 1.56E-06 |
| HCST | | 0.248121 | 0.15509 | 0.130585 | 0.21444 | 0.148531 | 1.59E-06 |
| STX10 | | 0.22875 | 0.251358 | 0.214503 | 0.304869 | 0.22198 | 1.59E-06 |
| IBTK | | 0.233006 | 0.348434 | 0.296469 | 0.390022 | 0.29543 | 1.59E-06 |
| TECPR1 | | 0.231625 | 0.452222 | 0.385147 | 0.46414 | 0.365071 | 1.6E-06 |
| RIPK1 | | 0.254908 | 0.213302 | 0.178755 | 0.260254 | 0.186072 | 1.61E-06 |
| LIAS | | 0.318274 | 0.128409 | 0.102988 | 0.171504 | 0.113166 | 1.63E-06 |
| LRSAM1 | | 0.339208 | 0.175965 | 0.139096 | 0.217798 | 0.151795 | 1.64E-06 |
| H2AZ1 | | 0.258901 | 0.235896 | 0.197144 | 0.279683 | 0.202938 | 1.64E-06 |
| RMDN3 | | 0.28589 | 0.144007 | 0.118119 | 0.199328 | 0.135473 | 1.65E-06 |
| VPS25 | | 0.238335 | 0.166727 | 0.141338 | 0.23243 | 0.162133 | 1.65E-06 |
| SLC2A5 | | 0.388074 | 0.124046 | 0.09479 | 0.159751 | 0.104461 | 1.68E-06 |
| SCRN2 | | 0.257437 | 0.149867 | 0.125374 | 0.207244 | 0.142002 | 1.69E-06 |
| LAPTM4A | | 0.250395 | 0.224855 | 0.189028 | 0.2852 | 0.206202 | 1.7E-06 |
| INPP5K | | 0.206326 | 0.412423 | 0.357464 | 0.440873 | 0.340588 | 1.71E-06 |
| ZMYND19 | | 0.376528 | 0.113116 | 0.087132 | 0.153514 | 0.099021 | 1.71E-06 |
| FN3KRP | | 0.330925 | 0.193834 | 0.154103 | 0.241545 | 0.173014 | 1.74E-06 |
| TRAPPC4 | | 0.216597 | 0.357895 | 0.308002 | 0.399136 | 0.303591 | 1.74E-06 |
| TXNDC15 | | 0.234928 | 0.414557 | 0.35226 | 0.425522 | 0.331338 | 1.76E-06 |
| TRAM2 | | 0.385607 | 0.199443 | 0.152665 | 0.211082 | 0.148531 | 1.76E-06 |
| SLC3A2 | | 0.254442 | 0.401282 | 0.336399 | 0.411849 | 0.322089 | 1.79E-06 |
| MPG | | 0.229562 | 0.29762 | 0.253839 | 0.35668 | 0.264962 | 1.79E-06 |
| SMG9 | | 0.362894 | 0.164086 | 0.127594 | 0.207244 | 0.144178 | 1.81E-06 |
| DNTTIP1 | | 0.241407 | 0.172391 | 0.145829 | 0.236508 | 0.165941 | 1.81E-06 |
| SCYL1 | | 0.239663 | 0.366065 | 0.310037 | 0.397457 | 0.303591 | 1.89E-06 |
| VPS54 | | 0.279657 | 0.1773 | 0.146057 | 0.229312 | 0.161045 | 1.9E-06 |
| ITPR2 | | 0.201604 | 0.628505 | 0.546537 | 0.58863 | 0.484766 | 1.93E-06 |
| CASP3 | | 0.338247 | 0.236444 | 0.187028 | 0.259055 | 0.188792 | 1.95E-06 |
| SUN1 | | 0.233964 | 0.28779 | 0.244707 | 0.340369 | 0.255169 | 1.95E-06 |
| MOCS2 | | 0.237648 | 0.202756 | 0.171963 | 0.24946 | 0.177911 | 1.98E-06 |
| COTL1 | | 0.194786 | 0.798832 | 0.697941 | 0.648357 | 0.563112 | 2E-06 |
| ZNF251 | | 0.469035 | 0.119163 | 0.086089 | 0.138163 | 0.087595 | 2.01E-06 |
| TRIAP1 | | 0.291529 | 0.139139 | 0.113682 | 0.194771 | 0.132753 | 2.02E-06 |
| NAA80 | | 0.280121 | 0.196675 | 0.161966 | 0.236268 | 0.167029 | 2.05E-06 |
| EML2 | | 0.28891 | 0.203489 | 0.166559 | 0.259055 | 0.18716 | 2.06E-06 |
| GPANK1 | | 0.332641 | 0.144668 | 0.114878 | 0.192852 | 0.132209 | 2.1E-06 |
| TMEM267 | | 0.358855 | 0.128426 | 0.100145 | 0.157592 | 0.102285 | 2.14E-06 |
| CANX | | 0.332582 | 0.297046 | 0.235889 | 0.318302 | 0.244831 | 2.15E-06 |
| TMEM273 | | 1.113416 | 0.045906 | 0.021217 | 0.055169 | 0.024483 | 2.16E-06 |
| GTPBP2 | | 0.231296 | 0.212559 | 0.181072 | 0.266731 | 0.191513 | 2.17E-06 |
| GLRX3 | | 0.20985 | 0.42009 | 0.363221 | 0.451667 | 0.348749 | 2.18E-06 |
| SLC39A6 | | 0.279262 | 0.205082 | 0.16899 | 0.261454 | 0.188792 | 2.2E-06 |
| SMIM26 | | 0.291477 | 0.156187 | 0.127615 | 0.218038 | 0.151795 | 2.23E-06 |
| BRMS1 | | 0.203517 | 0.277789 | 0.24124 | 0.336771 | 0.24864 | 2.27E-06 |
| TIMMDC1 | | 0.218817 | 0.304253 | 0.261436 | 0.358599 | 0.269314 | 2.27E-06 |
| TMEM70 | | 0.356261 | 0.112962 | 0.088245 | 0.169105 | 0.112622 | 2.29E-06 |
| PSMC1 | | 0.285126 | 0.160662 | 0.131851 | 0.23291 | 0.164309 | 2.31E-06 |
| FERMT3 | | 0.210718 | 0.64902 | 0.560823 | 0.596306 | 0.489663 | 2.32E-06 |
| EMC6 | | 0.297272 | 0.160712 | 0.130785 | 0.222355 | 0.157236 | 2.37E-06 |
| ABCB10 | | 0.321234 | 0.149589 | 0.119729 | 0.202207 | 0.14037 | 2.39E-06 |
| ZDHHC16 | | 0.248594 | 0.193759 | 0.16309 | 0.247541 | 0.175734 | 2.41E-06 |
| RNF181 | | 0.247626 | 0.199321 | 0.167885 | 0.265052 | 0.190968 | 2.43E-06 |
| ANAPC2 | | 0.23413 | 0.238882 | 0.203097 | 0.30391 | 0.223069 | 2.47E-06 |
| KIAA1143 | | 0.275726 | 0.16339 | 0.134966 | 0.209882 | 0.144723 | 2.51E-06 |
| DHRS7 | | 0.23717 | 0.236123 | 0.200329 | 0.293116 | 0.214908 | 2.51E-06 |
| CD80 | | 1.226956 | 0.037955 | 0.016215 | 0.048693 | 0.020131 | 2.52E-06 |
| TMCO3 | | 0.262819 | 0.16221 | 0.135195 | 0.206045 | 0.142002 | 2.52E-06 |
| PECR | | 0.328828 | 0.132502 | 0.105496 | 0.172463 | 0.115343 | 2.53E-06 |
| ASPSCR1 | | 0.251642 | 0.255697 | 0.21477 | 0.30319 | 0.224157 | 2.53E-06 |
| PGD | | 0.210684 | 0.231057 | 0.199663 | 0.296234 | 0.214363 | 2.54E-06 |
| GIT1 | | 0.332688 | 0.17098 | 0.135768 | 0.21444 | 0.150707 | 2.56E-06 |
| MMP24OS | | 0.224496 | 0.325428 | 0.278532 | 0.366275 | 0.279108 | 2.57E-06 |
| OSBPL3 | | 0.235465 | 0.159913 | 0.135832 | 0.221636 | 0.153428 | 2.57E-06 |
| TMEM219 | | 0.197368 | 0.305338 | 0.266298 | 0.362197 | 0.26877 | 2.6E-06 |
| PLIN3 | | 0.245946 | 0.206388 | 0.17404 | 0.26697 | 0.193145 | 2.6E-06 |
| PDIA5 | | 0.460504 | 0.123529 | 0.089773 | 0.149916 | 0.098477 | 2.6E-06 |
| ATP5PF | | 0.344004 | 0.129738 | 0.102215 | 0.19573 | 0.135473 | 2.6E-06 |
| SEPTIN11 | | 0.255601 | 0.165286 | 0.13845 | 0.219957 | 0.153428 | 2.62E-06 |
| TMEM256 | | 0.224316 | 0.19061 | 0.163162 | 0.257376 | 0.182263 | 2.69E-06 |
| COG1 | | 0.278444 | 0.152789 | 0.125971 | 0.202207 | 0.139282 | 2.7E-06 |
| ZBTB7B | | 0.197392 | 0.471489 | 0.411198 | 0.486687 | 0.381393 | 2.71E-06 |
| UQCR11 | | 0.232812 | 0.345893 | 0.294346 | 0.408251 | 0.31284 | 2.72E-06 |
| YEATS4 | | 0.31174 | 0.154734 | 0.124665 | 0.201967 | 0.139282 | 2.72E-06 |
| GFOD1 | | 0.324659 | 0.223835 | 0.178729 | 0.243943 | 0.177367 | 2.76E-06 |
| SEC61G | | 0.339314 | 0.195631 | 0.15463 | 0.234109 | 0.168118 | 2.79E-06 |
| OGDH | | 0.226149 | 0.436374 | 0.373062 | 0.457184 | 0.35691 | 2.8E-06 |
| IQGAP2 | | 0.245859 | 0.295806 | 0.249457 | 0.336052 | 0.251904 | 2.81E-06 |
| THYN1 | | 0.256739 | 0.171887 | 0.143865 | 0.228112 | 0.160501 | 2.81E-06 |
| ARF6 | | 0.141326 | 1.154962 | 1.047187 | 0.829456 | 0.749184 | 2.83E-06 |
| MDH1 | | 0.279494 | 0.098254 | 0.08095 | 0.155433 | 0.100653 | 2.89E-06 |
| AAK1 | | 0.323507 | 0.106887 | 0.085416 | 0.154234 | 0.100653 | 2.89E-06 |
| LPCAT3 | | 0.375231 | 0.102434 | 0.078975 | 0.151595 | 0.099021 | 2.94E-06 |
| GCDH | | 0.370686 | 0.109301 | 0.084535 | 0.159751 | 0.10555 | 2.94E-06 |
| MLX | | 0.230025 | 0.291608 | 0.248631 | 0.343967 | 0.256257 | 2.94E-06 |
| NDUFB1 | | 0.215718 | 0.214439 | 0.184657 | 0.290477 | 0.212187 | 2.96E-06 |
| ALDH18A1 | | 0.267141 | 0.201271 | 0.167249 | 0.262413 | 0.189336 | 2.97E-06 |
| HERPUD1 | | 0.202705 | 0.809219 | 0.703147 | 0.632766 | 0.531012 | 3E-06 |
| GMIP | | 0.201424 | 0.641299 | 0.557733 | 0.58911 | 0.493471 | 3E-06 |
| ABHD14A | | 0.296379 | 0.089185 | 0.072623 | 0.14296 | 0.091404 | 3.01E-06 |
| RAB3GAP2 | | 0.216512 | 0.241681 | 0.208001 | 0.297913 | 0.219804 | 3.02E-06 |
| C11orf24 | | 0.172645 | 0.410766 | 0.364437 | 0.441833 | 0.332427 | 3.03E-06 |
| POLR2F | | 0.265769 | 0.207044 | 0.17221 | 0.269609 | 0.195865 | 3.03E-06 |
| RALGDS | | 0.336929 | 0.165138 | 0.130744 | 0.21396 | 0.151251 | 3.06E-06 |
| CHMP6 | | 0.251096 | 0.175883 | 0.147787 | 0.226193 | 0.159412 | 3.06E-06 |
| ANAPC5 | | 0.198524 | 0.582915 | 0.507977 | 0.563924 | 0.446681 | 3.06E-06 |
| OPA1 | | 0.192636 | 0.344624 | 0.301548 | 0.399376 | 0.302503 | 3.06E-06 |
| UFC1 | | 0.178781 | 0.870556 | 0.769092 | 0.688175 | 0.59086 | 3.07E-06 |
| ARL1 | | 0.247456 | 0.172325 | 0.145163 | 0.236987 | 0.167573 | 3.12E-06 |
| YJU2B | | 0.282294 | 0.22476 | 0.184816 | 0.26745 | 0.195865 | 3.12E-06 |
| ESRRA | | 0.312241 | 0.158784 | 0.127883 | 0.206764 | 0.144723 | 3.18E-06 |
| NDUFC2 | | 0.240132 | 0.430614 | 0.364587 | 0.437515 | 0.34494 | 3.19E-06 |
| NAA60 | | 0.221828 | 0.293116 | 0.251341 | 0.351163 | 0.262786 | 3.19E-06 |
| DLAT | | 0.253509 | 0.1691 | 0.14185 | 0.230511 | 0.162677 | 3.27E-06 |
| TULP4 | | 0.204023 | 0.354068 | 0.307376 | 0.386663 | 0.294886 | 3.27E-06 |
| TOR1A | | 0.180898 | 0.193286 | 0.170508 | 0.254018 | 0.180631 | 3.38E-06 |
| LMNB1 | | 0.269245 | 0.28793 | 0.238911 | 0.30271 | 0.227421 | 3.41E-06 |
| TRIM65 | | 0.247407 | 0.182723 | 0.153928 | 0.224035 | 0.156692 | 3.41E-06 |
| LPIN1 | | 0.246367 | 0.174677 | 0.147255 | 0.233149 | 0.164853 | 3.44E-06 |
| MAN1A2 | | 0.245941 | 0.282759 | 0.238441 | 0.333173 | 0.247552 | 3.45E-06 |
| ATP6V0E1 | | 0.201691 | 0.52741 | 0.4586 | 0.543775 | 0.437432 | 3.45E-06 |
| SPPL3 | | 0.245001 | 0.235128 | 0.198405 | 0.288079 | 0.211643 | 3.45E-06 |
| NGLY1 | | 0.191868 | 0.670887 | 0.587342 | 0.633965 | 0.522307 | 3.46E-06 |
| ZFAND2A | | 0.221601 | 0.327381 | 0.280766 | 0.377069 | 0.285092 | 3.49E-06 |
| SUPT4H1 | | 0.227 | 0.287785 | 0.245886 | 0.359319 | 0.272035 | 3.53E-06 |
| CHMP2A | | 0.233148 | 0.360552 | 0.306749 | 0.404893 | 0.311208 | 3.56E-06 |
| SYS1 | | 0.242107 | 0.224393 | 0.189726 | 0.26721 | 0.193145 | 3.56E-06 |
| NSMCE4A | | 0.221966 | 0.354304 | 0.303778 | 0.387383 | 0.299238 | 3.57E-06 |
| RASSF1 | | 0.202657 | 0.382172 | 0.332088 | 0.408251 | 0.313384 | 3.57E-06 |
| LACTB | | 0.282962 | 0.151518 | 0.124533 | 0.191413 | 0.131121 | 3.57E-06 |
| CC2D1A | | 0.308479 | 0.108898 | 0.087934 | 0.152794 | 0.099565 | 3.58E-06 |
| PHF10 | | 0.190352 | 0.628722 | 0.551007 | 0.597745 | 0.487486 | 3.58E-06 |
| COQ8A | | 0.259497 | 0.261933 | 0.218813 | 0.30439 | 0.227965 | 3.6E-06 |
| EEF2K | | 0.391982 | 0.098282 | 0.074899 | 0.14344 | 0.093036 | 3.61E-06 |
| GET3 | | 0.246417 | 0.279686 | 0.235772 | 0.336052 | 0.253536 | 3.62E-06 |
| CLTA | | 0.22405 | 0.305317 | 0.261399 | 0.359079 | 0.272035 | 3.64E-06 |
| SHLD1 | | 0.242664 | 0.18552 | 0.156798 | 0.229551 | 0.162133 | 3.69E-06 |
| ZMPSTE24 | | 0.29831 | 0.152609 | 0.124102 | 0.212041 | 0.148531 | 3.73E-06 |
| SLC25A19 | | 0.281794 | 0.193723 | 0.15935 | 0.238906 | 0.170294 | 3.74E-06 |
| BTBD7 | | 0.244054 | 0.303421 | 0.256199 | 0.345407 | 0.263874 | 3.74E-06 |
| ADAM28 | | 0.265036 | 0.352152 | 0.293053 | 0.331974 | 0.251904 | 3.77E-06 |
| CERK | | 0.191467 | 0.404331 | 0.354079 | 0.42936 | 0.334603 | 3.77E-06 |
| COPS9 | | 0.253812 | 0.164964 | 0.138351 | 0.236987 | 0.168118 | 3.82E-06 |
| GSTP1 | | 0.2463 | 0.561913 | 0.473724 | 0.475654 | 0.387922 | 3.87E-06 |
| SLC30A6 | | 0.249348 | 0.158926 | 0.133701 | 0.213001 | 0.148531 | 3.88E-06 |
| PARN | | 0.254548 | 0.227755 | 0.190916 | 0.282322 | 0.208379 | 3.91E-06 |
| TRIM11 | | 0.352221 | 0.123792 | 0.096976 | 0.164068 | 0.109358 | 3.94E-06 |
| RNF168 | | 0.220907 | 0.323101 | 0.277229 | 0.372991 | 0.285092 | 3.94E-06 |
| ALAD | | 0.306208 | 0.173331 | 0.140184 | 0.221876 | 0.158324 | 3.94E-06 |
| CALU | | 0.334982 | 0.216205 | 0.171406 | 0.246102 | 0.179543 | 3.96E-06 |
| ORC2 | | 0.303272 | 0.174935 | 0.141769 | 0.220197 | 0.155604 | 3.99E-06 |
| CCNC | | 0.248863 | 0.358372 | 0.301591 | 0.370832 | 0.286181 | 4.07E-06 |
| FAM217B | | 0.367401 | 0.108103 | 0.083799 | 0.150636 | 0.099021 | 4.08E-06 |
| MANF | | 0.406531 | 0.392549 | 0.296153 | 0.2852 | 0.221436 | 4.11E-06 |
| GPR65 | | 0.292674 | 0.20078 | 0.163914 | 0.229312 | 0.162133 | 4.14E-06 |
| LRRK1 | | 0.275338 | 0.20971 | 0.173274 | 0.257856 | 0.188248 | 4.14E-06 |
| MCM9 | | 0.385588 | 0.108837 | 0.083312 | 0.144639 | 0.09358 | 4.15E-06 |
| UBALD1 | | 0.336477 | 0.13983 | 0.110741 | 0.189494 | 0.130577 | 4.16E-06 |
| CHERP | | 0.195953 | 0.329297 | 0.287475 | 0.385464 | 0.287813 | 4.17E-06 |
| STX4 | | 0.238079 | 0.30253 | 0.256507 | 0.353322 | 0.268226 | 4.18E-06 |
| NUCKS1 | | 0.223221 | 0.420713 | 0.360404 | 0.456704 | 0.355822 | 4.2E-06 |
| NTMT1 | | 0.242897 | 0.255838 | 0.216195 | 0.297194 | 0.220348 | 4.2E-06 |
| ARHGAP25 | | 0.197218 | 0.623723 | 0.544031 | 0.564404 | 0.471164 | 4.23E-06 |
| PEMT | | 0.291587 | 0.094559 | 0.077255 | 0.139842 | 0.089227 | 4.26E-06 |
| ATP13A1 | | 0.218216 | 0.505676 | 0.434693 | 0.490525 | 0.39445 | 4.3E-06 |
| ASL | | 0.356025 | 0.109091 | 0.085234 | 0.164548 | 0.110446 | 4.31E-06 |
| NOC2L | | 0.197938 | 0.334319 | 0.291458 | 0.387623 | 0.292165 | 4.32E-06 |
| C6orf120 | | 0.218075 | 0.25133 | 0.216072 | 0.292636 | 0.214363 | 4.35E-06 |
| PQBP1 | | 0.212569 | 0.372507 | 0.321473 | 0.413528 | 0.318825 | 4.35E-06 |
| GRINA | | 0.187467 | 0.489592 | 0.429933 | 0.495083 | 0.394995 | 4.36E-06 |
| SLC25A4 | | 0.32795 | 0.144603 | 0.115201 | 0.185896 | 0.127856 | 4.38E-06 |
| PAGR1 | | 0.184493 | 0.312505 | 0.274991 | 0.366994 | 0.275299 | 4.4E-06 |
| ELP1 | | 0.242345 | 0.156056 | 0.131925 | 0.213001 | 0.147987 | 4.42E-06 |
| FHOD1 | | 0.341458 | 0.105248 | 0.083066 | 0.152555 | 0.100653 | 4.44E-06 |
| RNF121 | | 0.29406 | 0.143984 | 0.117434 | 0.190933 | 0.131121 | 4.52E-06 |
| BPNT2 | | 0.222432 | 0.338478 | 0.290116 | 0.382826 | 0.291621 | 4.56E-06 |
| SFMBT2 | | 0.250921 | 0.22684 | 0.190627 | 0.278484 | 0.205658 | 4.77E-06 |
| WIPI2 | | 0.196078 | 0.451316 | 0.393963 | 0.494123 | 0.388466 | 4.77E-06 |
| HAUS1 | | 0.334384 | 0.149512 | 0.118582 | 0.203646 | 0.143634 | 4.87E-06 |
| BFAR | | 0.24199 | 0.196761 | 0.166377 | 0.262893 | 0.191513 | 4.88E-06 |
| ZMYM6 | | 0.254323 | 0.280571 | 0.235225 | 0.324298 | 0.244831 | 4.91E-06 |
| TBL2 | | 0.321351 | 0.158934 | 0.127198 | 0.206045 | 0.145267 | 5.02E-06 |
| SSR1 | | 0.37723 | 0.157521 | 0.121277 | 0.206045 | 0.146355 | 5.06E-06 |
| SRPRA | | 0.208709 | 0.714801 | 0.618526 | 0.591029 | 0.506529 | 5.08E-06 |
| SLC39A9 | | 0.249856 | 0.247982 | 0.208548 | 0.30415 | 0.227965 | 5.11E-06 |
| TAP1 | | 0.183995 | 0.832357 | 0.732693 | 0.679779 | 0.577802 | 5.11E-06 |
| ZNF330 | | 0.22783 | 0.179148 | 0.152977 | 0.238906 | 0.170838 | 5.17E-06 |
| HMGCR | | 0.227989 | 0.168837 | 0.144157 | 0.227153 | 0.160501 | 5.17E-06 |
| PLEKHG2 | | 0.319328 | 0.170471 | 0.136623 | 0.216119 | 0.153428 | 5.2E-06 |
| RHOQ | | 0.175391 | 0.804304 | 0.712233 | 0.645958 | 0.538085 | 5.2E-06 |
| PBXIP1 | | 0.188825 | 0.608513 | 0.53386 | 0.564404 | 0.451034 | 5.24E-06 |
| IL4R | | 0.293911 | 0.533548 | 0.435209 | 0.402495 | 0.332971 | 5.25E-06 |
| MCEE | | 0.214923 | 0.141102 | 0.121572 | 0.19693 | 0.135473 | 5.26E-06 |
| TSNAX | | 0.217404 | 0.319306 | 0.274639 | 0.371792 | 0.284548 | 5.31E-06 |
| COX16 | | 0.222837 | 0.433371 | 0.371346 | 0.4651 | 0.366703 | 5.33E-06 |
| MLEC | | 0.268035 | 0.384423 | 0.319244 | 0.366035 | 0.286181 | 5.35E-06 |
| RRAGA | | 0.224647 | 0.277759 | 0.237707 | 0.327896 | 0.249184 | 5.37E-06 |
| PSMC2 | | 0.24461 | 0.243466 | 0.205496 | 0.295515 | 0.218716 | 5.45E-06 |
| FURIN | | 0.223896 | 0.349241 | 0.299037 | 0.388822 | 0.29815 | 5.5E-06 |
| LZTS2 | | 0.295696 | 0.170539 | 0.138935 | 0.201487 | 0.140914 | 5.56E-06 |
| RAP2B | | 0.281475 | 0.217677 | 0.179093 | 0.273447 | 0.202394 | 5.58E-06 |
| PHF6 | | 0.258931 | 0.20516 | 0.171453 | 0.262653 | 0.192057 | 5.59E-06 |
| NAAA | | 0.215456 | 0.244213 | 0.210334 | 0.287359 | 0.212731 | 5.61E-06 |
| TIPARP | | 0.212515 | 0.513692 | 0.443332 | 0.493164 | 0.399891 | 5.74E-06 |
| AZIN1 | | 0.210069 | 0.264892 | 0.228998 | 0.333653 | 0.249728 | 5.74E-06 |
| RRBP1 | | 0.301961 | 0.162228 | 0.131591 | 0.185656 | 0.127312 | 5.77E-06 |
| ERAL1 | | 0.208009 | 0.207306 | 0.179471 | 0.273207 | 0.198585 | 5.77E-06 |
| H4C16 | | 0.430993 | 0.132726 | 0.09845 | 0.148477 | 0.098477 | 5.82E-06 |
| AKT1S1 | | 0.294579 | 0.116982 | 0.095376 | 0.16095 | 0.107182 | 5.94E-06 |
| RAD17 | | 0.282784 | 0.168435 | 0.138454 | 0.228592 | 0.163765 | 5.95E-06 |
| NCR3 | | 0.368358 | 0.193663 | 0.150024 | 0.195491 | 0.136561 | 6.06E-06 |
| BCAS4 | | 0.253342 | 0.420664 | 0.352917 | 0.39242 | 0.311208 | 6.06E-06 |
| DNAJB14 | | 0.242054 | 0.174602 | 0.147633 | 0.227153 | 0.161045 | 6.15E-06 |
| KAT2A | | 0.293409 | 0.216216 | 0.176426 | 0.254258 | 0.18716 | 6.15E-06 |
| PRKAR1B | | 0.373691 | 0.120979 | 0.093372 | 0.164308 | 0.111534 | 6.17E-06 |
| IGFLR1 | | 0.192239 | 0.50897 | 0.445474 | 0.497242 | 0.397715 | 6.19E-06 |
| PTRHD1 | | 0.212905 | 0.276828 | 0.238846 | 0.323339 | 0.243199 | 6.23E-06 |
| CCDC102A | | 0.318963 | 0.15415 | 0.123574 | 0.189974 | 0.132753 | 6.26E-06 |
| FKBP3 | | 0.197419 | 0.15712 | 0.137026 | 0.225714 | 0.159412 | 6.28E-06 |
| JAGN1 | | 0.270979 | 0.129665 | 0.107461 | 0.182778 | 0.124592 | 6.31E-06 |
| PGAP2 | | 0.235837 | 0.236684 | 0.200991 | 0.277764 | 0.205658 | 6.31E-06 |
| ATP2B4 | | 0.321189 | 0.110281 | 0.08827 | 0.16095 | 0.107726 | 6.38E-06 |
| RNF126 | | 0.190318 | 0.365887 | 0.320668 | 0.414008 | 0.314472 | 6.46E-06 |
| AIFM1 | | 0.267647 | 0.170524 | 0.14165 | 0.229312 | 0.165397 | 6.53E-06 |
| VRK3 | | 0.226409 | 0.189969 | 0.162378 | 0.244183 | 0.17519 | 6.54E-06 |
| PSMA3 | | 0.277776 | 0.14251 | 0.117551 | 0.204605 | 0.143634 | 6.56E-06 |
| CCNL2 | | 0.195693 | 0.56207 | 0.490773 | 0.554569 | 0.45049 | 6.58E-06 |
| ZNF706 | | 0.207561 | 0.391249 | 0.338822 | 0.440873 | 0.341676 | 6.66E-06 |
| RRAGD | | 1.901106 | 0.016322 | 0.00437 | 0.031422 | 0.009793 | 6.69E-06 |
| MBOAT7 | | 0.290675 | 0.141024 | 0.11529 | 0.19693 | 0.138194 | 6.69E-06 |
| ATG101 | | 0.197144 | 0.270554 | 0.235997 | 0.325018 | 0.241567 | 6.69E-06 |
| PCID2 | | 0.212714 | 0.238743 | 0.206014 | 0.295275 | 0.218172 | 6.74E-06 |
| R3HCC1 | | 0.27659 | 0.145963 | 0.120498 | 0.207004 | 0.146355 | 6.78E-06 |
| ARL16 | | 0.24308 | 0.149187 | 0.126054 | 0.193811 | 0.133297 | 6.85E-06 |
| RSAD1 | | 0.195499 | 0.310587 | 0.271227 | 0.352842 | 0.268226 | 6.89E-06 |
| SEC24C | | 0.228589 | 0.375526 | 0.3205 | 0.420485 | 0.32481 | 6.91E-06 |
| PPP2R1B | | 0.201548 | 0.25722 | 0.223683 | 0.298153 | 0.220348 | 6.91E-06 |
| RFNG | | 0.248269 | 0.153857 | 0.129533 | 0.19669 | 0.136561 | 6.95E-06 |
| TRAP1 | | 0.280851 | 0.183469 | 0.151015 | 0.235788 | 0.170294 | 7.13E-06 |
| GLRX5 | | 0.253505 | 0.20954 | 0.175774 | 0.265531 | 0.193145 | 7.31E-06 |
| RARA | | 0.21079 | 0.432561 | 0.37376 | 0.42792 | 0.336235 | 7.32E-06 |
| TRIM8 | | 0.202717 | 0.384807 | 0.334364 | 0.425042 | 0.331338 | 7.34E-06 |
| GARS1 | | 0.260991 | 0.165128 | 0.137802 | 0.218997 | 0.155604 | 7.41E-06 |
| PIGL | | 0.26509 | 0.234046 | 0.194761 | 0.270329 | 0.201306 | 7.44E-06 |
| SAT1 | | 0.161225 | 0.905427 | 0.809692 | 0.701607 | 0.584331 | 7.44E-06 |
| LSS | | 0.283477 | 0.166657 | 0.136927 | 0.21468 | 0.152339 | 7.45E-06 |
| RCC1L | | 0.192302 | 0.352371 | 0.308398 | 0.393859 | 0.301959 | 7.58E-06 |
| SRPRB | | 0.320205 | 0.335991 | 0.269114 | 0.313984 | 0.244831 | 7.6E-06 |
| WDR83OS | | 0.202016 | 0.613545 | 0.533376 | 0.574718 | 0.472252 | 7.61E-06 |
| DPP9 | | 0.213612 | 0.312543 | 0.26953 | 0.369153 | 0.282372 | 7.71E-06 |
| UBXN11 | | 0.354634 | 0.155827 | 0.121867 | 0.185416 | 0.128945 | 7.73E-06 |
| BANF1 | | 0.197157 | 0.232084 | 0.202439 | 0.30271 | 0.222524 | 7.74E-06 |
| SACM1L | | 0.212499 | 0.30012 | 0.259016 | 0.354521 | 0.270403 | 7.81E-06 |
| TSFM | | 0.253822 | 0.156355 | 0.131131 | 0.208443 | 0.146355 | 7.84E-06 |
| GPD2 | | 0.306092 | 0.124832 | 0.100968 | 0.163588 | 0.109902 | 7.85E-06 |
| TMEM131 | | 0.184581 | 0.4623 | 0.40678 | 0.494843 | 0.392818 | 7.85E-06 |
| NME2 | | 0.189099 | 0.706546 | 0.619749 | 0.605901 | 0.516866 | 7.92E-06 |
| PMM2 | | 0.225921 | 0.276968 | 0.236821 | 0.320461 | 0.241023 | 8.02E-06 |
| NCF2 | | 0.343172 | 0.138813 | 0.109427 | 0.162869 | 0.108814 | 8.07E-06 |
| SBF1 | | 0.217419 | 0.288667 | 0.248283 | 0.340849 | 0.258977 | 8.15E-06 |
| ADO | | 0.290561 | 0.112391 | 0.091889 | 0.153754 | 0.101741 | 8.2E-06 |
| EXOSC3 | | 0.259079 | 0.141653 | 0.118368 | 0.189254 | 0.131121 | 8.23E-06 |
| TMEM258 | | 0.261871 | 0.368003 | 0.306916 | 0.37515 | 0.294886 | 8.23E-06 |
| NDFIP1 | | 0.170525 | 0.279032 | 0.247925 | 0.348045 | 0.257345 | 8.23E-06 |
| MICOS13 | | 0.263118 | 0.118676 | 0.098891 | 0.170544 | 0.115887 | 8.24E-06 |
| SCML1 | | 1.295952 | 0.030416 | 0.012387 | 0.03646 | 0.013058 | 8.43E-06 |
| NOP9 | | 0.240934 | 0.210519 | 0.178141 | 0.269129 | 0.197497 | 8.43E-06 |
| DIAPH2 | | 0.349526 | 0.169708 | 0.133194 | 0.195011 | 0.136561 | 8.44E-06 |
| CSNK1E | | 0.263931 | 0.235385 | 0.196032 | 0.289038 | 0.217084 | 8.44E-06 |
| KTN1 | | 0.203066 | 0.3635 | 0.315773 | 0.422643 | 0.32753 | 8.56E-06 |
| RRM1 | | 0.301033 | 0.140554 | 0.114083 | 0.176541 | 0.120783 | 8.56E-06 |
| BAG1 | | 0.206164 | 0.399427 | 0.346239 | 0.421684 | 0.329162 | 8.56E-06 |
| THAP7 | | 0.273311 | 0.117962 | 0.097604 | 0.169105 | 0.114799 | 8.58E-06 |
| SYTL3 | | 1.19685 | 0.012033 | 0.005249 | 0.029503 | 0.008705 | 8.59E-06 |
| TM9SF1 | | 0.237154 | 0.221816 | 0.188192 | 0.277525 | 0.20457 | 8.65E-06 |
| C5orf15 | | 0.236446 | 0.16212 | 0.137613 | 0.212041 | 0.150707 | 8.79E-06 |
| CDC16 | | 0.188224 | 0.485946 | 0.426508 | 0.513073 | 0.40642 | 8.79E-06 |
| TST | | 1.494821 | 0.013167 | 0.004672 | 0.03622 | 0.013058 | 8.81E-06 |
| GBA2 | | 0.20062 | 0.242541 | 0.211054 | 0.300072 | 0.223613 | 8.83E-06 |
| SLFN11 | | 0.213731 | 0.188024 | 0.162134 | 0.239866 | 0.17247 | 8.91E-06 |
| PIGO | | 0.295132 | 0.154827 | 0.126184 | 0.205805 | 0.146355 | 8.92E-06 |
| MED17 | | 0.159126 | 0.328356 | 0.294065 | 0.378508 | 0.286181 | 9E-06 |
| HIBCH | | 0.265373 | 0.117163 | 0.097478 | 0.177261 | 0.121328 | 9.11E-06 |
| SNX11 | | 0.310259 | 0.13732 | 0.110748 | 0.180619 | 0.12568 | 9.16E-06 |
| STRADA | | 0.280422 | 0.195382 | 0.160868 | 0.237947 | 0.173558 | 9.22E-06 |
| TRIM47 | | 1.248345 | 0.030118 | 0.012678 | 0.051811 | 0.023939 | 9.22E-06 |
| PSMB2 | | 0.2365 | 0.280367 | 0.237976 | 0.332454 | 0.251904 | 9.25E-06 |
| CHSY1 | | 0.22788 | 0.165376 | 0.141213 | 0.218997 | 0.156148 | 9.26E-06 |
| PIP4K2A | | 0.205509 | 0.314986 | 0.273166 | 0.369153 | 0.284004 | 9.27E-06 |
| IMP4 | | 0.204928 | 0.419694 | 0.364119 | 0.432958 | 0.341132 | 9.27E-06 |
| LMBR1 | | 0.17878 | 0.22212 | 0.196232 | 0.282082 | 0.206746 | 9.31E-06 |
| PES1 | | 0.224574 | 0.227292 | 0.194527 | 0.284001 | 0.208379 | 9.33E-06 |
| SIDT1 | | 0.251532 | 0.195157 | 0.163933 | 0.246342 | 0.181719 | 9.43E-06 |
| ARFGAP2 | | 0.203529 | 0.34002 | 0.295281 | 0.391221 | 0.303047 | 9.55E-06 |
| ORAI2 | | 0.144687 | 1.23888 | 1.120661 | 0.846006 | 0.758433 | 9.59E-06 |
| GZF1 | | 0.279886 | 0.188917 | 0.155602 | 0.226433 | 0.163765 | 9.79E-06 |
| TMBIM6 | | 0.224341 | 0.258878 | 0.221596 | 0.331254 | 0.250272 | 9.87E-06 |
| ELP6 | | 0.233023 | 0.148376 | 0.126246 | 0.207004 | 0.145811 | 9.87E-06 |
| ARPC3 | | 0.187851 | 0.486595 | 0.427188 | 0.524346 | 0.418934 | 9.89E-06 |
| FDFT1 | | 0.206656 | 0.32109 | 0.278238 | 0.366994 | 0.28346 | 1.01E-05 |
| CBX6 | | 0.234843 | 0.258726 | 0.219859 | 0.315183 | 0.237758 | 1.01E-05 |
| NSF | | 0.20941 | 0.293208 | 0.253593 | 0.350204 | 0.266594 | 1.01E-05 |
| UBE2N | | 0.203689 | 0.190307 | 0.165248 | 0.259535 | 0.188792 | 1.01E-05 |
| CKLF | | 0.198779 | 0.200483 | 0.174678 | 0.256656 | 0.186616 | 1.01E-05 |
| HID1 | | 1.06675 | 0.044217 | 0.021109 | 0.062605 | 0.0321 | 1.01E-05 |
| CLUH | | 0.198983 | 0.165761 | 0.144405 | 0.220197 | 0.156148 | 1.04E-05 |
| ITPR3 | | 0.194105 | 0.287383 | 0.251206 | 0.331494 | 0.250272 | 1.04E-05 |
| DGLUCY | | 0.206939 | 0.198948 | 0.172363 | 0.253778 | 0.184984 | 1.04E-05 |
| TXLNA | | 0.208904 | 0.432755 | 0.374417 | 0.450947 | 0.354189 | 1.04E-05 |
| PTS | | 0.170654 | 0.284745 | 0.252979 | 0.335332 | 0.25136 | 1.05E-05 |
| SH3BP5L | | 0.188875 | 0.208747 | 0.183132 | 0.258575 | 0.187704 | 1.07E-05 |
| BASP1 | | 0.199415 | 0.353502 | 0.307867 | 0.387383 | 0.298694 | 1.08E-05 |
| SMIM15 | | 0.207427 | 0.319129 | 0.276391 | 0.362677 | 0.275299 | 1.09E-05 |
| CINP | | 0.254701 | 0.132838 | 0.11134 | 0.191653 | 0.133841 | 1.1E-05 |
| YY1AP1 | | 0.181938 | 0.297118 | 0.261915 | 0.363876 | 0.275843 | 1.11E-05 |
| MYG1 | | 0.248272 | 0.144529 | 0.121679 | 0.19717 | 0.138738 | 1.11E-05 |
| SLX9 | | 0.281383 | 0.130622 | 0.107476 | 0.182058 | 0.126224 | 1.11E-05 |
| UROS | | 0.244298 | 0.163218 | 0.137793 | 0.217558 | 0.155604 | 1.12E-05 |
| AGPS | | 0.203234 | 0.262564 | 0.228063 | 0.326937 | 0.245375 | 1.12E-05 |
| METTL3 | | 0.17089 | 0.395534 | 0.351351 | 0.439434 | 0.342764 | 1.13E-05 |
| PHF19 | | 0.286255 | 0.17772 | 0.145736 | 0.188055 | 0.131121 | 1.13E-05 |
| RAPGEF2 | | 0.305571 | 0.150128 | 0.121472 | 0.204605 | 0.146355 | 1.13E-05 |
| KPNA2 | | 0.220304 | 0.173639 | 0.149049 | 0.227153 | 0.162133 | 1.13E-05 |
| SPTLC2 | | 0.190098 | 0.510914 | 0.44784 | 0.509954 | 0.414037 | 1.15E-05 |
| OXCT1 | | 0.21474 | 0.234082 | 0.201709 | 0.28544 | 0.212731 | 1.17E-05 |
| REXO4 | | 0.190599 | 0.237636 | 0.208226 | 0.290717 | 0.215996 | 1.18E-05 |
| FOXK2 | | 0.215142 | 0.232464 | 0.200259 | 0.298633 | 0.223069 | 1.19E-05 |
| GADD45GIP1 | | 0.213352 | 0.334466 | 0.288487 | 0.376349 | 0.289989 | 1.19E-05 |
| ACSL4 | | 0.199604 | 0.207194 | 0.180422 | 0.272967 | 0.200762 | 1.2E-05 |
| CCDC82 | | 0.225093 | 0.304604 | 0.2606 | 0.348045 | 0.269859 | 1.2E-05 |
| LSM1 | | 0.19926 | 0.301833 | 0.262896 | 0.350444 | 0.265506 | 1.2E-05 |
| PGM3 | | 0.264646 | 0.127836 | 0.106411 | 0.17798 | 0.12296 | 1.22E-05 |
| PGAP6 | | 0.176047 | 0.260079 | 0.230202 | 0.324538 | 0.242655 | 1.22E-05 |
| PPP1R21 | | 0.194964 | 0.370632 | 0.323782 | 0.408251 | 0.316104 | 1.22E-05 |
| SNX8 | | 0.242585 | 0.29209 | 0.246883 | 0.3219 | 0.246464 | 1.23E-05 |
| RASGRP1 | | 0.192368 | 0.476566 | 0.417075 | 0.472535 | 0.376496 | 1.23E-05 |
| MCCC2 | | 0.243984 | 0.148792 | 0.125642 | 0.210122 | 0.149075 | 1.25E-05 |
| RASA2 | | 0.178065 | 0.40808 | 0.360697 | 0.450468 | 0.350381 | 1.25E-05 |
| SLC35D1 | | 0.28167 | 0.157245 | 0.129356 | 0.205325 | 0.145811 | 1.25E-05 |
| GGA3 | | 0.19557 | 0.299765 | 0.261763 | 0.355001 | 0.269314 | 1.25E-05 |
| PEG10 | | 0.360414 | 0.226055 | 0.176084 | 0.189734 | 0.134385 | 1.25E-05 |
| DEAF1 | | 0.20215 | 0.262293 | 0.227999 | 0.307268 | 0.229053 | 1.25E-05 |
| RAP2A | | 0.272081 | 0.118453 | 0.098094 | 0.173183 | 0.119695 | 1.27E-05 |
| NAA40 | | 0.260577 | 0.173169 | 0.144554 | 0.212041 | 0.151795 | 1.28E-05 |
| CIAO3 | | 0.264099 | 0.122566 | 0.102063 | 0.169825 | 0.115887 | 1.29E-05 |
| COA3 | | 0.22149 | 0.183432 | 0.157326 | 0.243224 | 0.176823 | 1.29E-05 |
| USP5 | | 0.240417 | 0.238884 | 0.202215 | 0.301031 | 0.226877 | 1.29E-05 |
| PRPSAP1 | | 0.265044 | 0.15122 | 0.125842 | 0.206284 | 0.146899 | 1.32E-05 |
| ESYT2 | | 0.200076 | 0.50571 | 0.440223 | 0.49964 | 0.40642 | 1.32E-05 |
| ATPAF1 | | 0.242025 | 0.159702 | 0.135038 | 0.218038 | 0.156692 | 1.34E-05 |
| RUFY3 | | 0.179494 | 0.416152 | 0.367467 | 0.447349 | 0.353101 | 1.35E-05 |
| SLC12A9 | | 0.194543 | 0.328234 | 0.286827 | 0.35764 | 0.274755 | 1.37E-05 |
| ELMO1 | | 0.174728 | 0.488648 | 0.432911 | 0.491725 | 0.393362 | 1.38E-05 |
| PPT1 | | 0.269804 | 0.187502 | 0.15552 | 0.245383 | 0.180631 | 1.39E-05 |
| EXOC2 | | 0.197911 | 0.366129 | 0.319195 | 0.405373 | 0.319369 | 1.39E-05 |
| UTP3 | | 0.223564 | 0.248712 | 0.213009 | 0.30271 | 0.226877 | 1.4E-05 |
| PDE4B | | 0.163304 | 0.939634 | 0.839072 | 0.719117 | 0.626224 | 1.4E-05 |
| ASS1 | | 1.9458 | 0.015238 | 0.003955 | 0.021348 | 0.004353 | 1.4E-05 |
| SNAP23 | | 0.197363 | 0.257686 | 0.224739 | 0.305829 | 0.230686 | 1.4E-05 |
| TUBA1C | | 0.280898 | 0.1065 | 0.087658 | 0.155673 | 0.104461 | 1.41E-05 |
| SUPT5H | | 0.198818 | 0.598792 | 0.521706 | 0.576877 | 0.477149 | 1.43E-05 |
| NLRC3 | | 0.208853 | 0.389496 | 0.337002 | 0.401535 | 0.318825 | 1.45E-05 |
| EBP | | 0.277122 | 0.20949 | 0.172879 | 0.247541 | 0.183351 | 1.46E-05 |
| ACSL5 | | 0.19972 | 0.360156 | 0.313595 | 0.404653 | 0.311752 | 1.46E-05 |
| MEF2D | | 0.202766 | 0.528167 | 0.458916 | 0.5349 | 0.433079 | 1.47E-05 |
| UROD | | 0.204559 | 0.190138 | 0.165003 | 0.247541 | 0.180631 | 1.47E-05 |
| TNIP2 | | 0.22266 | 0.284086 | 0.243457 | 0.324059 | 0.245375 | 1.47E-05 |
| PGM2 | | 0.2335 | 0.231508 | 0.196913 | 0.281842 | 0.211099 | 1.48E-05 |
| APOL6 | | 0.162071 | 0.106954 | 0.095589 | 0.157832 | 0.10555 | 1.49E-05 |
| PHIP | | 0.182726 | 0.329748 | 0.29052 | 0.383545 | 0.294886 | 1.49E-05 |
| DCAF8 | | 0.177939 | 0.576035 | 0.509195 | 0.570401 | 0.462459 | 1.5E-05 |
| MARK2 | | 0.179538 | 0.533691 | 0.471242 | 0.547613 | 0.443417 | 1.52E-05 |
| TIMM44 | | 0.240217 | 0.23291 | 0.197185 | 0.280883 | 0.210011 | 1.53E-05 |
| ATP6V1A | | 0.219981 | 0.187114 | 0.160652 | 0.244423 | 0.178999 | 1.53E-05 |
| KTI12 | | 0.249916 | 0.128576 | 0.108126 | 0.181818 | 0.126224 | 1.55E-05 |
| UBA1 | | 0.206548 | 0.339805 | 0.294478 | 0.400576 | 0.309576 | 1.55E-05 |
| ZNF460 | | 0.225156 | 0.191669 | 0.163973 | 0.246582 | 0.179543 | 1.58E-05 |
| ATP6V1F | | 0.197617 | 0.454244 | 0.396096 | 0.48165 | 0.384657 | 1.58E-05 |
| BTBD6 | | 0.323339 | 0.14749 | 0.117876 | 0.183257 | 0.1284 | 1.58E-05 |
| APOL3 | | 0.240192 | 0.138722 | 0.117447 | 0.194771 | 0.13765 | 1.6E-05 |
| BPNT1 | | 0.161464 | 0.170041 | 0.152037 | 0.230031 | 0.165397 | 1.6E-05 |
| GGCT | | 0.273375 | 0.140683 | 0.116398 | 0.187815 | 0.132209 | 1.61E-05 |
| TMED3 | | 0.262008 | 0.199242 | 0.166153 | 0.257616 | 0.190968 | 1.64E-05 |
| TMEM138 | | 0.253676 | 0.181021 | 0.151832 | 0.227393 | 0.165397 | 1.67E-05 |
| GDE1 | | 0.218656 | 0.144839 | 0.12447 | 0.204126 | 0.144723 | 1.67E-05 |
| BABAM2 | | 0.260367 | 0.160366 | 0.133885 | 0.204366 | 0.146355 | 1.68E-05 |
| GLB1 | | 0.216698 | 0.237416 | 0.204304 | 0.290237 | 0.217084 | 1.68E-05 |
| SEC16A | | 0.199429 | 0.280887 | 0.244623 | 0.343488 | 0.261153 | 1.68E-05 |
| DGKG | | 1.092017 | 0.035339 | 0.016578 | 0.043416 | 0.018498 | 1.69E-05 |
| RAB2B | | 0.266977 | 0.147878 | 0.122896 | 0.194531 | 0.13765 | 1.69E-05 |
| TTC3 | | 0.188975 | 0.517429 | 0.453904 | 0.53562 | 0.431447 | 1.7E-05 |
| G6PD | | 0.210015 | 0.269078 | 0.232626 | 0.330535 | 0.25136 | 1.71E-05 |
| ARAP1 | | 0.191392 | 0.511879 | 0.448283 | 0.516671 | 0.41839 | 1.71E-05 |
| ZMYND11 | | 0.220492 | 0.248963 | 0.213678 | 0.30343 | 0.229053 | 1.71E-05 |
| NT5C2 | | 0.184545 | 0.400264 | 0.352203 | 0.448069 | 0.351469 | 1.72E-05 |
| LONP1 | | 0.212514 | 0.265869 | 0.229454 | 0.318302 | 0.242111 | 1.75E-05 |
| CCPG1 | | 0.247848 | 0.176486 | 0.148628 | 0.230031 | 0.166485 | 1.77E-05 |
| MYDGF | | 0.311054 | 0.615181 | 0.495868 | 0.431039 | 0.357998 | 1.77E-05 |
| KXD1 | | 0.167835 | 0.479441 | 0.426787 | 0.480451 | 0.381393 | 1.78E-05 |
| AP2A1 | | 0.195755 | 0.261433 | 0.228261 | 0.320221 | 0.243199 | 1.79E-05 |
| ATG2B | | 0.228143 | 0.19832 | 0.169312 | 0.253778 | 0.186616 | 1.81E-05 |
| UBE2G1 | | 0.208913 | 0.603973 | 0.522551 | 0.55289 | 0.458107 | 1.81E-05 |
| MBIP | | 0.212078 | 0.130914 | 0.113017 | 0.189254 | 0.132209 | 1.81E-05 |
| SPPL2A | | 0.183256 | 0.314005 | 0.276549 | 0.369153 | 0.281284 | 1.82E-05 |
| TNS3 | | 0.279868 | 0.271783 | 0.223858 | 0.275366 | 0.207291 | 1.82E-05 |
| OSBPL8 | | 0.175511 | 0.491338 | 0.435057 | 0.506596 | 0.408052 | 1.82E-05 |
| MRGBP | | 0.287943 | 0.15345 | 0.125686 | 0.201487 | 0.144178 | 1.83E-05 |
| ARFGAP1 | | 0.236183 | 0.121492 | 0.103145 | 0.186855 | 0.130577 | 1.84E-05 |
| APH1A | | 0.193857 | 0.448759 | 0.392335 | 0.485248 | 0.385201 | 1.84E-05 |
| SLC20A1 | | 0.25331 | 0.312787 | 0.262419 | 0.333413 | 0.258433 | 1.84E-05 |
| ZDHHC18 | | 0.222419 | 0.150782 | 0.12924 | 0.210602 | 0.150707 | 1.84E-05 |
| SEC13 | | 0.229821 | 0.542628 | 0.462721 | 0.478292 | 0.39173 | 1.85E-05 |
| MTHFD1 | | 0.223795 | 0.292213 | 0.250225 | 0.331254 | 0.254625 | 1.88E-05 |
| TPM4 | | 0.224195 | 0.326127 | 0.279189 | 0.384025 | 0.298694 | 1.88E-05 |
| TEPSIN | | 0.315542 | 0.148225 | 0.119106 | 0.181578 | 0.127856 | 1.89E-05 |
| PPP6R2 | | 0.186037 | 0.507163 | 0.445805 | 0.519789 | 0.415125 | 1.93E-05 |
| CSNK1A1 | | 0.205247 | 0.236779 | 0.20538 | 0.311346 | 0.234494 | 1.95E-05 |
| ESCO1 | | 0.201086 | 0.199375 | 0.173436 | 0.25066 | 0.182263 | 1.97E-05 |
| ECE1 | | 0.179717 | 0.565167 | 0.498972 | 0.533941 | 0.433624 | 1.97E-05 |
| TRIM41 | | 0.18637 | 0.217253 | 0.190925 | 0.26745 | 0.198585 | 1.97E-05 |
| GALT | | 0.24477 | 0.161775 | 0.13653 | 0.212041 | 0.151795 | 1.97E-05 |
| PA2G4 | | 0.182223 | 0.343171 | 0.302452 | 0.377788 | 0.291077 | 1.97E-05 |
| RHEX | | 1.484163 | 0.025359 | 0.009065 | 0.038139 | 0.015234 | 1.98E-05 |
| ATP6V0D1 | | 0.189866 | 0.450206 | 0.39469 | 0.48165 | 0.381937 | 2E-05 |
| HTATIP2 | | 0.246999 | 0.196308 | 0.165418 | 0.233869 | 0.171382 | 2E-05 |
| DUSP2 | | 0.275832 | 0.374555 | 0.309373 | 0.297194 | 0.232862 | 2E-05 |
| CCND2 | | 0.213477 | 0.414783 | 0.357733 | 0.387143 | 0.306311 | 2E-05 |
| WDR55 | | 0.237106 | 0.179779 | 0.152533 | 0.238187 | 0.174102 | 2.01E-05 |
| RRP1 | | 0.226641 | 0.210221 | 0.17966 | 0.26697 | 0.198041 | 2.02E-05 |
| GSPT1 | | 0.217474 | 0.195139 | 0.167833 | 0.259775 | 0.190968 | 2.02E-05 |
| DHDDS | | 0.174053 | 0.148507 | 0.131629 | 0.205805 | 0.145811 | 2.02E-05 |
| FEZ2 | | 0.319456 | 0.127099 | 0.101853 | 0.168146 | 0.116431 | 2.03E-05 |
| EIF2B4 | | 0.245238 | 0.126864 | 0.107033 | 0.17894 | 0.125136 | 2.03E-05 |
| STAU2 | | 0.169977 | 0.471818 | 0.419379 | 0.472296 | 0.374864 | 2.03E-05 |
| COPB1 | | 0.204535 | 0.443362 | 0.384758 | 0.472056 | 0.37704 | 2.04E-05 |
| HSPBP1 | | 0.213976 | 0.203829 | 0.175733 | 0.263133 | 0.194233 | 2.04E-05 |
| PSMD11 | | 0.260808 | 0.129484 | 0.10807 | 0.188774 | 0.132753 | 2.06E-05 |
| WASHC3 | | 0.181741 | 0.322933 | 0.28471 | 0.382106 | 0.293798 | 2.06E-05 |
| C1GALT1C1 | | 0.250176 | 0.117139 | 0.09849 | 0.173423 | 0.120783 | 2.06E-05 |
| AHR | | 0.297298 | 0.136204 | 0.110839 | 0.166707 | 0.114799 | 2.07E-05 |
| STT3A | | 0.278424 | 0.436352 | 0.359768 | 0.365076 | 0.294886 | 2.07E-05 |
| VCP | | 0.226078 | 0.330502 | 0.282565 | 0.376349 | 0.293798 | 2.07E-05 |
| ZNF44 | | 0.288568 | 0.128343 | 0.105076 | 0.16143 | 0.110446 | 2.08E-05 |
| ZNF451 | | 0.276532 | 0.141278 | 0.116636 | 0.186136 | 0.130577 | 2.1E-05 |
| NDUFA8 | | 0.173586 | 0.235254 | 0.208584 | 0.289998 | 0.215452 | 2.1E-05 |
| ZBTB17 | | 0.207579 | 0.20476 | 0.17732 | 0.260494 | 0.193145 | 2.11E-05 |
| TRIM26 | | 0.187544 | 0.472767 | 0.415136 | 0.496522 | 0.397171 | 2.11E-05 |
| ATP6V1C2 | | 1.269734 | 0.020216 | 0.008384 | 0.027345 | 0.008161 | 2.11E-05 |
| DPP3 | | 0.237397 | 0.116482 | 0.098809 | 0.171024 | 0.117519 | 2.13E-05 |
| PSMA7 | | 0.198579 | 0.418211 | 0.364432 | 0.459343 | 0.36235 | 2.13E-05 |
| FBXW5 | | 0.19004 | 0.437398 | 0.383415 | 0.459822 | 0.365615 | 2.14E-05 |
| CR1 | | 0.218005 | 0.550724 | 0.473487 | 0.452866 | 0.37432 | 2.14E-05 |
| AASDHPPT | | 0.184119 | 0.252831 | 0.222538 | 0.314704 | 0.237214 | 2.14E-05 |
| AGPAT4 | | 0.311075 | 0.161547 | 0.130213 | 0.179899 | 0.126224 | 2.17E-05 |
| BTAF1 | | 0.170059 | 0.624458 | 0.555022 | 0.600624 | 0.492927 | 2.17E-05 |
| ANKRD28 | | 0.29484 | 0.17893 | 0.145857 | 0.231231 | 0.171926 | 2.18E-05 |
| TACC1 | | 0.186795 | 0.54298 | 0.477038 | 0.525786 | 0.427639 | 2.2E-05 |
| ARL2 | | 0.264891 | 0.141689 | 0.117922 | 0.183018 | 0.1284 | 2.21E-05 |
| BIN2 | | 0.188187 | 0.43849 | 0.384866 | 0.466299 | 0.368335 | 2.23E-05 |
| GSTO1 | | 0.327653 | 0.13036 | 0.103875 | 0.189254 | 0.135473 | 2.23E-05 |
| ELL | | 0.225224 | 0.216168 | 0.184923 | 0.270089 | 0.201306 | 2.23E-05 |
| BCAT2 | | 0.267542 | 0.166653 | 0.138445 | 0.21492 | 0.155604 | 2.23E-05 |
| NDUFS6 | | 0.216319 | 0.221209 | 0.190408 | 0.28592 | 0.214363 | 2.26E-05 |
| TXNRD2 | | 0.182447 | 0.176236 | 0.1553 | 0.224274 | 0.162133 | 2.28E-05 |
| AKR1A1 | | 0.20398 | 0.464207 | 0.403003 | 0.462461 | 0.374864 | 2.28E-05 |
| BCKDHB | | 0.333604 | 0.123808 | 0.098248 | 0.169105 | 0.118063 | 2.29E-05 |
| GNB2 | | 0.177684 | 0.560088 | 0.495186 | 0.565363 | 0.458651 | 2.29E-05 |
| PPP1R7 | | 0.198095 | 0.456344 | 0.397795 | 0.470137 | 0.375952 | 2.3E-05 |
| UBR7 | | 0.195852 | 0.224307 | 0.195833 | 0.279444 | 0.208379 | 2.34E-05 |
| PLEKHB2 | | 0.171096 | 0.402833 | 0.357783 | 0.443032 | 0.34494 | 2.36E-05 |
| FXR2 | | 0.196583 | 0.165143 | 0.144107 | 0.222835 | 0.161045 | 2.37E-05 |
| WBP1 | | 0.212496 | 0.24516 | 0.211583 | 0.299832 | 0.227421 | 2.37E-05 |
| DHX38 | | 0.174561 | 0.309179 | 0.273944 | 0.366515 | 0.28074 | 2.4E-05 |
| TPCN1 | | 0.294039 | 0.157549 | 0.128499 | 0.191893 | 0.136561 | 2.44E-05 |
| ATP5F1C | | 0.281518 | 0.103412 | 0.085079 | 0.164308 | 0.113711 | 2.46E-05 |
| BCL2L13 | | 0.161521 | 0.41254 | 0.368844 | 0.42888 | 0.336235 | 2.47E-05 |
| HDDC3 | | 0.205814 | 0.16168 | 0.140185 | 0.208683 | 0.149619 | 2.5E-05 |
| HDLBP | | 0.266416 | 0.305421 | 0.253922 | 0.323339 | 0.251904 | 2.5E-05 |
| HSPA14 | | 0.262439 | 0.126567 | 0.105516 | 0.170065 | 0.118063 | 2.5E-05 |
| SLC16A5 | | 2.976968 | 0.011517 | 0.001463 | 0.015351 | 0.001632 | 2.53E-05 |
| PLD3 | | 0.21492 | 0.410035 | 0.353284 | 0.426001 | 0.340044 | 2.53E-05 |
| SURF4 | | 0.255185 | 0.279821 | 0.234457 | 0.307268 | 0.237758 | 2.54E-05 |
| PSMG3 | | 0.218634 | 0.233022 | 0.200254 | 0.276325 | 0.207835 | 2.55E-05 |
| PIN1 | | 0.183614 | 0.295024 | 0.259767 | 0.360038 | 0.276387 | 2.56E-05 |
| TUT1 | | 0.226467 | 0.112724 | 0.096348 | 0.159511 | 0.108814 | 2.56E-05 |
| UQCR10 | | 0.191836 | 0.44102 | 0.386109 | 0.462701 | 0.368335 | 2.56E-05 |
| SNRPA1 | | 0.185809 | 0.265034 | 0.233007 | 0.322859 | 0.243199 | 2.56E-05 |
| GTF2E2 | | 0.203378 | 0.290564 | 0.252359 | 0.346606 | 0.267138 | 2.57E-05 |
| BDH1 | | 0.30262 | 0.128121 | 0.103878 | 0.163349 | 0.112078 | 2.57E-05 |
| MROH1 | | 0.195245 | 0.301746 | 0.263552 | 0.336292 | 0.258433 | 2.57E-05 |
| HIRA | | 0.205869 | 0.273583 | 0.237201 | 0.329096 | 0.252992 | 2.59E-05 |
| NRBP1 | | 0.186471 | 0.528439 | 0.464367 | 0.528184 | 0.429271 | 2.59E-05 |
| SMYD2 | | 0.247961 | 0.218279 | 0.183809 | 0.261454 | 0.196409 | 2.6E-05 |
| BAK1 | | 0.204191 | 0.349071 | 0.303002 | 0.360518 | 0.276387 | 2.6E-05 |
| SIL1 | | 0.261835 | 0.191769 | 0.15994 | 0.239386 | 0.176279 | 2.61E-05 |
| FAM78A | | 0.208552 | 0.381799 | 0.330411 | 0.413289 | 0.332971 | 2.62E-05 |
| COPE | | 0.213902 | 0.462834 | 0.399057 | 0.461262 | 0.372144 | 2.66E-05 |
| TMEM106C | | 0.301817 | 0.166295 | 0.134903 | 0.195251 | 0.14037 | 2.67E-05 |
| EIF2B3 | | 0.25327 | 0.15693 | 0.131663 | 0.207724 | 0.150163 | 2.69E-05 |
| TRANK1 | | 0.195557 | 0.433546 | 0.378588 | 0.453586 | 0.363983 | 2.69E-05 |
| NDUFA7 | | 0.245123 | 0.263603 | 0.222413 | 0.310866 | 0.238847 | 2.7E-05 |
| TXNL4A | | 0.186783 | 0.647592 | 0.56895 | 0.59007 | 0.495103 | 2.7E-05 |
| SF3B5 | | 0.176328 | 0.824948 | 0.73004 | 0.658431 | 0.557127 | 2.7E-05 |
| GOLPH3L | | 0.2175 | 0.174372 | 0.149969 | 0.233149 | 0.170838 | 2.71E-05 |
| USP48 | | 0.196003 | 0.511142 | 0.44621 | 0.512593 | 0.415669 | 2.72E-05 |
| PITRM1 | | 0.200991 | 0.243299 | 0.211659 | 0.305349 | 0.230141 | 2.74E-05 |
| UNC13D | | 0.186726 | 0.354834 | 0.311756 | 0.400336 | 0.313384 | 2.74E-05 |
| EIF4EBP1 | | 0.316358 | 0.143652 | 0.115366 | 0.191653 | 0.138194 | 2.74E-05 |
| MIF | | 0.165874 | 0.865378 | 0.771388 | 0.685776 | 0.5963 | 2.77E-05 |
| SYNGR2 | | 0.120516 | 1.396966 | 1.285011 | 0.85752 | 0.792709 | 2.78E-05 |
| PDZD11 | | 0.269136 | 0.130396 | 0.108204 | 0.182778 | 0.130033 | 2.79E-05 |
| VDAC3 | | 0.23043 | 0.208895 | 0.178058 | 0.273447 | 0.20457 | 2.82E-05 |
| INO80B | | 0.175007 | 0.285081 | 0.252514 | 0.331734 | 0.255169 | 2.82E-05 |
| CYB561D2 | | 0.213797 | 0.259338 | 0.223618 | 0.312785 | 0.238847 | 2.89E-05 |
| TP53INP2 | | 0.221592 | 0.260781 | 0.22365 | 0.289278 | 0.21926 | 2.89E-05 |
| MTX2 | | 0.183359 | 0.118926 | 0.104732 | 0.170065 | 0.117519 | 2.89E-05 |
| HMG20B | | 0.22269 | 0.272769 | 0.233754 | 0.324778 | 0.249184 | 2.91E-05 |
| SPI1 | | 0.12644 | 1.215525 | 1.113529 | 0.828256 | 0.737214 | 2.93E-05 |
| TBXA2R | | 0.321539 | 0.234902 | 0.187973 | 0.215879 | 0.158868 | 2.94E-05 |
| MIB2 | | 0.298179 | 0.140938 | 0.114622 | 0.173663 | 0.121872 | 2.95E-05 |
| LDAH | | 0.222096 | 0.16735 | 0.143472 | 0.218757 | 0.158868 | 2.95E-05 |
| RAD50 | | 0.194284 | 0.245188 | 0.214296 | 0.30343 | 0.230686 | 2.98E-05 |
| COX8A | | 0.231622 | 0.364733 | 0.310635 | 0.398177 | 0.316649 | 3.01E-05 |
| ADPRS | | 0.193864 | 0.223758 | 0.195623 | 0.265291 | 0.198041 | 3.01E-05 |
| APEH | | 0.189893 | 0.353898 | 0.310252 | 0.389782 | 0.301415 | 3.01E-05 |
| IL17RA | | 0.255965 | 0.203058 | 0.170046 | 0.245143 | 0.183351 | 3.02E-05 |
| PRDX5 | | 0.221487 | 0.254697 | 0.218449 | 0.314224 | 0.239391 | 3.03E-05 |
| NIPSNAP3A | | 0.218289 | 0.139523 | 0.119932 | 0.187095 | 0.132209 | 3.04E-05 |
| UBA5 | | 0.269977 | 0.246205 | 0.204186 | 0.28496 | 0.218172 | 3.04E-05 |
| NAA25 | | 0.217887 | 0.211754 | 0.182071 | 0.259055 | 0.192601 | 3.06E-05 |
| PPFIBP2 | | 0.197877 | 0.173792 | 0.151517 | 0.224035 | 0.162133 | 3.06E-05 |
| ISOC2 | | 0.289467 | 0.177536 | 0.145261 | 0.2142 | 0.157236 | 3.07E-05 |
| CCDC167 | | 0.274369 | 0.161171 | 0.133258 | 0.201487 | 0.144723 | 3.08E-05 |
| MLLT10 | | 0.202681 | 0.231194 | 0.200893 | 0.278244 | 0.208923 | 3.09E-05 |
| LITAF | | 0.145975 | 1.201564 | 1.085936 | 0.790357 | 0.707835 | 3.11E-05 |
| SFT2D1 | | 0.18133 | 0.367845 | 0.324398 | 0.416167 | 0.324266 | 3.12E-05 |
| CHDH | | 2.940742 | 0.014673 | 0.001911 | 0.021108 | 0.004897 | 3.16E-05 |
| CCDC141 | | 0.236376 | 0.41703 | 0.354007 | 0.383305 | 0.306855 | 3.17E-05 |
| PRKAG1 | | 0.193688 | 0.224023 | 0.195879 | 0.283041 | 0.212187 | 3.17E-05 |
| LYSMD3 | | 0.198333 | 0.194027 | 0.169105 | 0.25042 | 0.186072 | 3.18E-05 |
| TES | | 0.181362 | 0.56053 | 0.494315 | 0.55289 | 0.449946 | 3.18E-05 |
| PCSK7 | | 0.186642 | 0.616975 | 0.542104 | 0.583353 | 0.490207 | 3.19E-05 |
| CNPY2 | | 0.235783 | 0.259186 | 0.220107 | 0.295754 | 0.224701 | 3.19E-05 |
| HEXD | | 0.221809 | 0.284353 | 0.24383 | 0.323099 | 0.24864 | 3.19E-05 |
| PIGT | | 0.216611 | 0.374816 | 0.322561 | 0.399856 | 0.315016 | 3.19E-05 |
| RHOG | | 0.167853 | 0.586355 | 0.521953 | 0.561526 | 0.464635 | 3.21E-05 |
| EIF4ENIF1 | | 0.223914 | 0.182135 | 0.155951 | 0.235548 | 0.173014 | 3.21E-05 |
| PNP | | 0.190893 | 0.316126 | 0.276947 | 0.352842 | 0.274211 | 3.22E-05 |
| ISG20 | | 0.104612 | 1.555621 | 1.446813 | 0.895898 | 0.843852 | 3.22E-05 |
| TMEM175 | | 0.175658 | 0.512952 | 0.454149 | 0.503238 | 0.410229 | 3.24E-05 |
| MRE11 | | 0.3086 | 0.161874 | 0.130701 | 0.202447 | 0.147443 | 3.31E-05 |
| RPAP1 | | 0.214282 | 0.146425 | 0.126215 | 0.195491 | 0.139282 | 3.31E-05 |
| MOB1B | | 0.299249 | 0.175512 | 0.142635 | 0.197409 | 0.141458 | 3.31E-05 |
| MORF4L1 | | 0.196539 | 0.257269 | 0.224504 | 0.331254 | 0.254625 | 3.32E-05 |
| SUDS3 | | 0.172507 | 0.212789 | 0.188808 | 0.272967 | 0.203482 | 3.32E-05 |
| SEC23A | | 0.208622 | 0.158742 | 0.137369 | 0.225234 | 0.164309 | 3.36E-05 |
| RNF185 | | 0.209001 | 0.136286 | 0.117906 | 0.185176 | 0.131121 | 3.37E-05 |
| CLHC1 | | 1.140812 | 0.03275 | 0.014852 | 0.040537 | 0.01741 | 3.38E-05 |
| HK1 | | 0.20834 | 0.233485 | 0.202088 | 0.283521 | 0.213819 | 3.41E-05 |
| NKAP | | 0.222129 | 0.170849 | 0.146468 | 0.218757 | 0.159412 | 3.43E-05 |
| PSMB6 | | 0.220736 | 0.243045 | 0.208564 | 0.304869 | 0.229053 | 3.43E-05 |
| CLPP | | 0.218327 | 0.378468 | 0.325317 | 0.397218 | 0.314472 | 3.44E-05 |
| TARS2 | | 0.265914 | 0.128464 | 0.10684 | 0.181818 | 0.128945 | 3.46E-05 |
| MAPK7 | | 0.273291 | 0.149836 | 0.123979 | 0.176781 | 0.125136 | 3.48E-05 |
| APBB3 | | 0.229806 | 0.136267 | 0.116202 | 0.167666 | 0.115887 | 3.49E-05 |
| RUVBL2 | | 0.279663 | 0.14434 | 0.118905 | 0.199089 | 0.144178 | 3.55E-05 |
| CHP1 | | 0.206629 | 0.257096 | 0.222789 | 0.318302 | 0.244831 | 3.57E-05 |
| EVI2A | | 0.211558 | 0.313558 | 0.27079 | 0.340369 | 0.264962 | 3.62E-05 |
| CNNM3 | | 0.204981 | 0.214512 | 0.1861 | 0.260014 | 0.194233 | 3.62E-05 |
| HCCS | | 0.184515 | 0.147853 | 0.130102 | 0.202447 | 0.145267 | 3.62E-05 |
| ATP6AP1 | | 0.178451 | 0.385736 | 0.340856 | 0.427201 | 0.334603 | 3.63E-05 |
| SUCLG1 | | 0.199682 | 0.246085 | 0.214277 | 0.30367 | 0.230686 | 3.64E-05 |
| LAMC1 | | 0.21038 | 0.389207 | 0.336395 | 0.399376 | 0.317737 | 3.65E-05 |
| ATOX1 | | 0.234596 | 0.154236 | 0.131089 | 0.21396 | 0.155604 | 3.68E-05 |
| INIP | | 0.221906 | 0.146596 | 0.125696 | 0.186136 | 0.131665 | 3.69E-05 |
| MPDU1 | | 0.203666 | 0.204769 | 0.17781 | 0.26793 | 0.200762 | 3.69E-05 |
| ASAH1 | | 0.202061 | 0.163765 | 0.142362 | 0.221396 | 0.160501 | 3.69E-05 |
| SNAP29 | | 0.198363 | 0.143533 | 0.125094 | 0.207964 | 0.150163 | 3.73E-05 |
| CPSF4 | | 0.245884 | 0.151034 | 0.127367 | 0.204845 | 0.149075 | 3.74E-05 |
| STK17B | | 0.103994 | 1.582838 | 1.472758 | 0.913169 | 0.861262 | 3.85E-05 |
| PRKAB1 | | 0.199613 | 0.302367 | 0.263296 | 0.334133 | 0.261153 | 3.89E-05 |
| ATP5MC1 | | 0.233123 | 0.168645 | 0.143482 | 0.225474 | 0.164309 | 3.9E-05 |
| CLDND1 | | 0.187073 | 0.309546 | 0.271901 | 0.351403 | 0.267138 | 3.93E-05 |
| LRRC58 | | 0.222873 | 0.156299 | 0.133926 | 0.213241 | 0.154516 | 3.99E-05 |
| TJAP1 | | 0.19642 | 0.203446 | 0.17755 | 0.260014 | 0.194233 | 4.03E-05 |
| PMS2 | | 0.256185 | 0.141557 | 0.118525 | 0.184217 | 0.131121 | 4.03E-05 |
| PDCD5 | | 0.249638 | 0.173268 | 0.145737 | 0.23147 | 0.171382 | 4.03E-05 |
| MED22 | | 0.214871 | 0.297961 | 0.25673 | 0.334613 | 0.259521 | 4.03E-05 |
| GYS1 | | 0.148973 | 0.201684 | 0.181898 | 0.262653 | 0.194233 | 4.04E-05 |
| ATG4B | | 0.152574 | 0.283798 | 0.255317 | 0.348285 | 0.266594 | 4.05E-05 |
| ATRN | | 0.29232 | 0.131898 | 0.107707 | 0.181578 | 0.130033 | 4.06E-05 |
| TMEM160 | | 0.158235 | 0.229471 | 0.205634 | 0.300312 | 0.224701 | 4.07E-05 |
| ATP5MK | | 0.226466 | 0.176512 | 0.15087 | 0.244663 | 0.181175 | 4.1E-05 |
| ASF1A | | 0.204069 | 0.130787 | 0.113536 | 0.181818 | 0.1284 | 4.11E-05 |
| TM9SF4 | | 0.15723 | 0.258394 | 0.231714 | 0.32214 | 0.244287 | 4.12E-05 |
| ATG5 | | 0.167599 | 0.282745 | 0.251734 | 0.345646 | 0.265506 | 4.23E-05 |
| ASB7 | | 0.223311 | 0.142959 | 0.122459 | 0.186855 | 0.132753 | 4.25E-05 |
| CKS1B | | 0.227748 | 0.188778 | 0.16121 | 0.226433 | 0.165397 | 4.29E-05 |
| RLF | | 0.178084 | 0.232834 | 0.205796 | 0.284241 | 0.213275 | 4.3E-05 |
| GATAD1 | | 0.250657 | 0.192276 | 0.161611 | 0.230271 | 0.170294 | 4.31E-05 |
| SDR39U1 | | 0.16648 | 0.422715 | 0.376645 | 0.455985 | 0.361262 | 4.33E-05 |
| JMJD6 | | 0.158469 | 0.268154 | 0.240259 | 0.326457 | 0.248096 | 4.38E-05 |
| C6orf62 | | 0.177372 | 0.372096 | 0.329049 | 0.430799 | 0.341676 | 4.43E-05 |
| IGSF8 | | 0.192409 | 0.261904 | 0.229204 | 0.318781 | 0.244287 | 4.43E-05 |
| PDXK | | 0.224357 | 0.390232 | 0.334029 | 0.385224 | 0.308487 | 4.47E-05 |
| DIAPH1 | | 0.163598 | 0.744312 | 0.664519 | 0.651715 | 0.543526 | 4.49E-05 |
| EGLN1 | | 0.172242 | 0.34244 | 0.303903 | 0.3929 | 0.307943 | 4.5E-05 |
| BORCS6 | | 0.186399 | 0.164432 | 0.144502 | 0.218997 | 0.159412 | 4.5E-05 |
| MED12 | | 0.214438 | 0.272391 | 0.234769 | 0.318062 | 0.247552 | 4.51E-05 |
| RANBP3 | | 0.160262 | 0.324064 | 0.289993 | 0.379947 | 0.296518 | 4.51E-05 |
| AP5Z1 | | 0.225526 | 0.218418 | 0.186809 | 0.269369 | 0.204026 | 4.51E-05 |
| IKBKG | | 0.174517 | 0.461491 | 0.40891 | 0.484049 | 0.390642 | 4.51E-05 |
| ARFGEF1 | | 0.278742 | 0.143416 | 0.118219 | 0.187335 | 0.133841 | 4.53E-05 |
| NPEPL1 | | 0.271341 | 0.172135 | 0.142622 | 0.207484 | 0.152339 | 4.53E-05 |
| TBC1D17 | | 0.173883 | 0.285576 | 0.25315 | 0.33917 | 0.261697 | 4.53E-05 |
| CCDC47 | | 0.180127 | 0.253421 | 0.223675 | 0.307748 | 0.234494 | 4.54E-05 |
| NDUFS3 | | 0.195671 | 0.364191 | 0.317999 | 0.405613 | 0.322089 | 4.54E-05 |
| SENP5 | | 0.211388 | 0.165317 | 0.142785 | 0.216599 | 0.158324 | 4.55E-05 |
| CRTC2 | | 0.173616 | 0.376704 | 0.333992 | 0.420964 | 0.33025 | 4.57E-05 |
| GEMIN7 | | 0.191688 | 0.386171 | 0.338124 | 0.416887 | 0.332971 | 4.58E-05 |
| HS2ST1 | | 0.147175 | 0.225013 | 0.20319 | 0.278724 | 0.208379 | 4.58E-05 |
| YWHAG | | 0.236113 | 0.212666 | 0.180559 | 0.278244 | 0.210011 | 4.59E-05 |
| PTPA | | 0.181096 | 0.396768 | 0.349963 | 0.422883 | 0.333515 | 4.6E-05 |
| TOB1 | | 0.186402 | 0.316546 | 0.278179 | 0.349244 | 0.272035 | 4.64E-05 |
| ATG9A | | 0.223995 | 0.215436 | 0.184455 | 0.262413 | 0.198041 | 4.66E-05 |
| BPGM | | 0.219064 | 0.167243 | 0.143683 | 0.216599 | 0.158868 | 4.66E-05 |
| MT1F | | 1.161207 | 0.01671 | 0.007472 | 0.030463 | 0.010881 | 4.69E-05 |
| CAPN3 | | 0.240524 | 0.247137 | 0.209186 | 0.273207 | 0.209467 | 4.69E-05 |
| C14orf119 | | 0.180237 | 0.239428 | 0.211309 | 0.298393 | 0.226877 | 4.69E-05 |
| DAPK2 | | 1.113812 | 0.036987 | 0.017091 | 0.043895 | 0.020131 | 4.69E-05 |
| LEO1 | | 0.136584 | 0.148194 | 0.134808 | 0.202686 | 0.145267 | 4.78E-05 |
| SPOCK2 | | 0.229033 | 0.353679 | 0.301761 | 0.33989 | 0.264418 | 4.78E-05 |
| NXPE3 | | 0.243825 | 0.211515 | 0.178625 | 0.25066 | 0.186616 | 4.79E-05 |
| RDH14 | | 0.206102 | 0.237482 | 0.205868 | 0.283521 | 0.215996 | 4.82E-05 |
| MTERF4 | | 0.153153 | 0.48088 | 0.432447 | 0.468458 | 0.375952 | 4.83E-05 |
| ENTPD6 | | 0.200579 | 0.154018 | 0.134026 | 0.207484 | 0.150163 | 4.87E-05 |
| OTULIN | | 0.18527 | 0.310569 | 0.273141 | 0.363636 | 0.284004 | 4.89E-05 |
| SERTAD3 | | 0.16609 | 0.23005 | 0.205033 | 0.263852 | 0.195865 | 4.89E-05 |
| KLF10 | | 0.157985 | 0.285933 | 0.256275 | 0.310146 | 0.23667 | 4.91E-05 |
| CD63 | | 0.231873 | 0.118793 | 0.101156 | 0.17894 | 0.127312 | 4.91E-05 |
| IFI6 | | 0.19942 | 0.259953 | 0.226393 | 0.281602 | 0.214908 | 4.92E-05 |
| NSMAF | | 0.167893 | 0.228553 | 0.203445 | 0.278484 | 0.208923 | 4.93E-05 |
| GOLIM4 | | 0.200552 | 0.13833 | 0.120377 | 0.191173 | 0.136561 | 4.93E-05 |
| HMOX2 | | 0.169972 | 0.372254 | 0.330882 | 0.412809 | 0.321001 | 4.96E-05 |
| DCTD | | 0.167321 | 0.325061 | 0.289465 | 0.370832 | 0.289445 | 4.99E-05 |
| PPP1CC | | 0.190437 | 0.269104 | 0.235827 | 0.333413 | 0.257345 | 5.08E-05 |
| MPLKIP | | 0.161968 | 0.233604 | 0.208796 | 0.286639 | 0.215996 | 5.09E-05 |
| NOC3L | | 0.153602 | 0.278622 | 0.250482 | 0.3219 | 0.245919 | 5.1E-05 |
| UQCC1 | | 0.158522 | 0.172542 | 0.154587 | 0.23171 | 0.170294 | 5.15E-05 |
| PSME3IP1 | | 0.239425 | 0.15662 | 0.13267 | 0.217798 | 0.159956 | 5.17E-05 |
| GMCL1 | | 0.27245 | 0.161238 | 0.133491 | 0.205325 | 0.149619 | 5.18E-05 |
| STAMBP | | 0.267151 | 0.135947 | 0.112966 | 0.184697 | 0.133297 | 5.18E-05 |
| MORC2 | | 0.16664 | 0.252304 | 0.224782 | 0.307748 | 0.235582 | 5.24E-05 |
| OGT | | 0.130078 | 1.165288 | 1.064819 | 0.815064 | 0.742111 | 5.25E-05 |
| VPS53 | | 0.156963 | 0.359707 | 0.322625 | 0.405613 | 0.319369 | 5.28E-05 |
| FAM177A1 | | 0.222832 | 0.169776 | 0.145478 | 0.227153 | 0.168662 | 5.29E-05 |
| RNFT1 | | 0.224726 | 0.251992 | 0.215644 | 0.281602 | 0.214363 | 5.3E-05 |
| HSCB | | 0.203245 | 0.123364 | 0.107153 | 0.177021 | 0.12568 | 5.31E-05 |
| TIMM8B | | 0.210564 | 0.292807 | 0.253044 | 0.323579 | 0.250272 | 5.32E-05 |
| ATRAID | | 0.177756 | 0.271254 | 0.23981 | 0.332694 | 0.252992 | 5.34E-05 |
| KPNA6 | | 0.148029 | 0.416743 | 0.376104 | 0.451907 | 0.359086 | 5.34E-05 |
| MFSD6 | | 0.172988 | 0.192515 | 0.170761 | 0.242504 | 0.181175 | 5.37E-05 |
| FLOT2 | | 0.151184 | 0.897269 | 0.808001 | 0.739746 | 0.633841 | 5.41E-05 |
| ASNSD1 | | 0.179562 | 0.221953 | 0.195978 | 0.271528 | 0.20457 | 5.44E-05 |
| SLBP | | 0.186873 | 0.432373 | 0.379843 | 0.431278 | 0.347116 | 5.45E-05 |
| ZBTB2 | | 0.1873 | 0.191585 | 0.168259 | 0.234109 | 0.174646 | 5.53E-05 |
| TAF1C | | 0.223147 | 0.173709 | 0.148816 | 0.215639 | 0.15778 | 5.57E-05 |
| FBXW8 | | 0.202026 | 0.20069 | 0.174466 | 0.248981 | 0.186072 | 5.59E-05 |
| ZNRD2 | | 0.181832 | 0.257934 | 0.22739 | 0.30319 | 0.23123 | 5.6E-05 |
| DPH2 | | 0.170624 | 0.206921 | 0.183841 | 0.256656 | 0.190968 | 5.6E-05 |
| MAZ | | 0.199537 | 0.250145 | 0.217834 | 0.310866 | 0.239935 | 5.63E-05 |
| LTN1 | | 0.144123 | 0.27936 | 0.252802 | 0.337971 | 0.259521 | 5.63E-05 |
| PRDX6 | | 0.188282 | 0.24097 | 0.211487 | 0.310626 | 0.23667 | 5.64E-05 |
| KRTCAP2 | | 0.193096 | 0.438363 | 0.383448 | 0.448789 | 0.365615 | 5.64E-05 |
| MED29 | | 0.155075 | 0.320214 | 0.28758 | 0.377309 | 0.293254 | 5.64E-05 |
| TMEM250 | | 0.222064 | 0.23556 | 0.201954 | 0.275846 | 0.211643 | 5.64E-05 |
| EXOSC7 | | 0.167287 | 0.226577 | 0.201771 | 0.280883 | 0.213275 | 5.76E-05 |
| DRG2 | | 0.230696 | 0.227378 | 0.193777 | 0.280163 | 0.214908 | 5.78E-05 |
| CD46 | | 0.170644 | 0.324952 | 0.288702 | 0.378508 | 0.296518 | 5.87E-05 |
| UBE2Q2 | | 0.166652 | 0.285408 | 0.254272 | 0.340849 | 0.263874 | 5.91E-05 |
| ABHD14B | | 0.163594 | 0.236286 | 0.210956 | 0.289518 | 0.21926 | 5.91E-05 |
| TFG | | 0.203108 | 0.218043 | 0.189409 | 0.278964 | 0.212187 | 5.92E-05 |
| LIG3 | | 0.242098 | 0.134877 | 0.114041 | 0.189254 | 0.136561 | 5.93E-05 |
| TUBB6 | | 0.194621 | 0.401053 | 0.350441 | 0.389542 | 0.306855 | 5.96E-05 |
| SNW1 | | 0.230604 | 0.139121 | 0.11857 | 0.194771 | 0.14037 | 5.97E-05 |
| NDUFS8 | | 0.249074 | 0.164991 | 0.13883 | 0.234109 | 0.17519 | 5.99E-05 |
| C2orf68 | | 0.125526 | 0.297797 | 0.272981 | 0.351403 | 0.26877 | 6.03E-05 |
| SOS1 | | 0.179495 | 0.389112 | 0.343591 | 0.432238 | 0.344396 | 6.1E-05 |
| NDUFS5 | | 0.246672 | 0.147895 | 0.124652 | 0.211562 | 0.154516 | 6.12E-05 |
| WRAP73 | | 0.196159 | 0.158982 | 0.138771 | 0.207724 | 0.150163 | 6.14E-05 |
| ATF6B | | 0.181547 | 0.389045 | 0.343043 | 0.437755 | 0.348749 | 6.14E-05 |
| ATP6V1C1 | | 0.159191 | 0.187108 | 0.16756 | 0.247781 | 0.183896 | 6.14E-05 |
| HMBOX1 | | 0.169177 | 0.497524 | 0.442472 | 0.508995 | 0.411861 | 6.14E-05 |
| CRACR2A | | 0.152726 | 0.238419 | 0.214469 | 0.28592 | 0.214908 | 6.2E-05 |
| HADHB | | 0.1444 | 0.279359 | 0.252751 | 0.345646 | 0.26605 | 6.29E-05 |
| EXOSC8 | | 0.175964 | 0.245676 | 0.217467 | 0.293116 | 0.22198 | 6.3E-05 |
| METTL26 | | 0.155399 | 0.39045 | 0.350579 | 0.417366 | 0.32753 | 6.34E-05 |
| FOLR2 | | 1.157205 | 0.025942 | 0.011632 | 0.033341 | 0.013058 | 6.34E-05 |
| CALCOCO2 | | 0.149917 | 0.426724 | 0.384607 | 0.461262 | 0.367247 | 6.34E-05 |
| TATDN3 | | 0.24255 | 0.134458 | 0.11365 | 0.17822 | 0.127312 | 6.37E-05 |
| POU2F1 | | 0.191993 | 0.189263 | 0.16568 | 0.245862 | 0.182807 | 6.37E-05 |
| ELOA | | 0.180619 | 0.279497 | 0.246607 | 0.328616 | 0.256801 | 6.38E-05 |
| USP45 | | 0.257268 | 0.150463 | 0.125888 | 0.188295 | 0.136017 | 6.4E-05 |
| FGFR1OP2 | | 0.183149 | 0.398528 | 0.351015 | 0.434157 | 0.344396 | 6.48E-05 |
| RAB1B | | 0.184772 | 0.567486 | 0.499267 | 0.561046 | 0.4679 | 6.49E-05 |
| ADAM17 | | 0.177172 | 0.305154 | 0.269889 | 0.363157 | 0.284004 | 6.5E-05 |
| SPIDR | | 0.283178 | 0.201322 | 0.165442 | 0.23219 | 0.17519 | 6.55E-05 |
| TSC22D4 | | 0.153815 | 0.801415 | 0.720368 | 0.680019 | 0.572905 | 6.55E-05 |
| RAB37 | | 0.263626 | 0.224 | 0.18659 | 0.240585 | 0.182807 | 6.56E-05 |
| ZNF708 | | 0.198168 | 0.186333 | 0.162419 | 0.224514 | 0.165397 | 6.59E-05 |
| EPB41L5 | | 0.313808 | 0.167876 | 0.135058 | 0.173183 | 0.12296 | 6.61E-05 |
| KIAA2013 | | 0.170578 | 0.389456 | 0.346027 | 0.432238 | 0.344396 | 6.62E-05 |
| CAPRIN1 | | 0.195492 | 0.158278 | 0.13822 | 0.220676 | 0.162133 | 6.63E-05 |
| TMEM199 | | 0.19603 | 0.24263 | 0.211804 | 0.289758 | 0.223069 | 6.63E-05 |
| IL16 | | 0.131066 | 1.050523 | 0.959292 | 0.770209 | 0.695865 | 6.63E-05 |
| SMG5 | | 0.180294 | 0.368865 | 0.325532 | 0.417366 | 0.330794 | 6.65E-05 |
| REL | | 0.127555 | 1.213973 | 1.111248 | 0.806189 | 0.733406 | 6.66E-05 |
| SNRNP27 | | 0.147597 | 0.204957 | 0.185026 | 0.264092 | 0.196953 | 6.68E-05 |
| MED11 | | 0.16524 | 0.208429 | 0.185873 | 0.256177 | 0.191513 | 6.71E-05 |
| CLASP2 | | 0.260482 | 0.179903 | 0.150185 | 0.226673 | 0.168662 | 6.72E-05 |
| METTL17 | | 0.166671 | 0.305527 | 0.272193 | 0.35644 | 0.280196 | 6.72E-05 |
| POP4 | | 0.168749 | 0.147455 | 0.131178 | 0.210842 | 0.153428 | 6.72E-05 |
| ABCC1 | | 0.174524 | 0.516175 | 0.457362 | 0.509475 | 0.414581 | 6.74E-05 |
| SSR4 | | 0.243561 | 0.465424 | 0.393124 | 0.422643 | 0.352013 | 6.79E-05 |
| CDIP1 | | 0.191894 | 0.223588 | 0.195742 | 0.279683 | 0.213275 | 6.85E-05 |
| FAM50A | | 0.136668 | 0.254655 | 0.231639 | 0.313984 | 0.237214 | 6.85E-05 |
| FASTKD5 | | 0.239379 | 0.160078 | 0.135603 | 0.210842 | 0.154516 | 6.87E-05 |
| KARS1 | | 0.157673 | 0.209542 | 0.187848 | 0.283281 | 0.212187 | 6.89E-05 |
| RBM42 | | 0.175267 | 0.484668 | 0.429224 | 0.502279 | 0.399891 | 6.92E-05 |
| FBXO34 | | 0.123601 | 0.195109 | 0.17909 | 0.247781 | 0.182807 | 6.96E-05 |
| STRN3 | | 0.138781 | 0.218209 | 0.198196 | 0.276565 | 0.206202 | 7.03E-05 |
| C5orf22 | | 0.174055 | 0.152959 | 0.135575 | 0.204126 | 0.147987 | 7.06E-05 |
| MTG1 | | 0.159339 | 0.42235 | 0.378187 | 0.454785 | 0.365615 | 7.06E-05 |
| ZBTB40 | | 0.268702 | 0.184929 | 0.153503 | 0.227633 | 0.171382 | 7.08E-05 |
| STX5 | | 0.171157 | 0.65117 | 0.578324 | 0.612377 | 0.506529 | 7.12E-05 |
| SRSF11 | | 0.192895 | 0.22044 | 0.192852 | 0.287599 | 0.219804 | 7.12E-05 |
| BSCL2 | | 0.204317 | 0.149615 | 0.129858 | 0.204366 | 0.148531 | 7.13E-05 |
| HOXC4 | | 1.446289 | 0.017239 | 0.006326 | 0.024706 | 0.007617 | 7.14E-05 |
| NDUFB3 | | 0.223527 | 0.215479 | 0.184551 | 0.282562 | 0.214908 | 7.15E-05 |
| CYP51A1 | | 0.204943 | 0.158375 | 0.137402 | 0.215639 | 0.158324 | 7.17E-05 |
| RETREG2 | | 0.17182 | 0.245558 | 0.217988 | 0.306069 | 0.23395 | 7.17E-05 |
| CLN3 | | 0.211518 | 0.148698 | 0.12842 | 0.203886 | 0.148531 | 7.17E-05 |
| GOLGA5 | | 0.155193 | 0.238885 | 0.214522 | 0.297194 | 0.226877 | 7.2E-05 |
| RAF1 | | 0.176474 | 0.356107 | 0.315106 | 0.414728 | 0.326442 | 7.2E-05 |
| ARF3 | | 0.162481 | 0.215851 | 0.19286 | 0.293356 | 0.22198 | 7.24E-05 |
| TOMM70 | | 0.154339 | 0.220782 | 0.198382 | 0.286879 | 0.215996 | 7.3E-05 |
| PWWP3A | | 0.175002 | 0.178161 | 0.157809 | 0.224754 | 0.164853 | 7.32E-05 |
| DIS3 | | 0.144242 | 0.299359 | 0.270877 | 0.355481 | 0.275299 | 7.33E-05 |
| TPST2 | | 0.238406 | 0.192719 | 0.163364 | 0.242264 | 0.182263 | 7.39E-05 |
| GRHPR | | 0.178178 | 0.404897 | 0.357856 | 0.419285 | 0.337867 | 7.42E-05 |
| HNRNPLL | | 1.306748 | 0.010959 | 0.00443 | 0.030463 | 0.011425 | 7.44E-05 |
| DNAJC2 | | 0.150796 | 0.235878 | 0.212468 | 0.296954 | 0.224157 | 7.48E-05 |
| C19orf53 | | 0.183372 | 0.337483 | 0.297202 | 0.398897 | 0.315016 | 7.5E-05 |
| DROSHA | | 0.20913 | 0.208956 | 0.180759 | 0.261693 | 0.196953 | 7.59E-05 |
| ABCF1 | | 0.155988 | 0.328566 | 0.294894 | 0.387143 | 0.300326 | 7.61E-05 |
| COG2 | | 0.195101 | 0.155238 | 0.135602 | 0.2142 | 0.157236 | 7.69E-05 |
| SLC37A1 | | 0.189207 | 0.301985 | 0.264868 | 0.346366 | 0.270947 | 7.69E-05 |
| PSME2 | | 0.223439 | 0.221595 | 0.189801 | 0.271528 | 0.207291 | 7.71E-05 |
| MCM2 | | 0.256333 | 0.206405 | 0.172805 | 0.215639 | 0.159956 | 7.76E-05 |
| DIS3L | | 0.210699 | 0.191748 | 0.165693 | 0.237467 | 0.177911 | 7.8E-05 |
| WRN | | 0.205343 | 0.133786 | 0.116037 | 0.183737 | 0.131121 | 7.83E-05 |
| NBAS | | 0.166324 | 0.211188 | 0.188192 | 0.276085 | 0.209467 | 7.83E-05 |
| TUSC2 | | 0.140329 | 0.165005 | 0.149711 | 0.225234 | 0.165397 | 7.83E-05 |
| KLRK1 | | 0.292718 | 0.314326 | 0.256604 | 0.261454 | 0.20457 | 7.86E-05 |
| SAP30BP | | 0.141835 | 0.286628 | 0.25979 | 0.347325 | 0.265506 | 7.93E-05 |
| TYMP | | 0.11237 | 0.217833 | 0.201511 | 0.279683 | 0.208379 | 7.95E-05 |
| CTNNBL1 | | 0.154289 | 0.265944 | 0.238971 | 0.323819 | 0.249184 | 7.97E-05 |
| TMF1 | | 0.16491 | 0.546543 | 0.487508 | 0.546894 | 0.454298 | 7.98E-05 |
| DERL2 | | 0.209948 | 0.302129 | 0.261211 | 0.337971 | 0.265506 | 8.05E-05 |
| WDR37 | | 0.114172 | 0.244612 | 0.226 | 0.298633 | 0.223613 | 8.05E-05 |
| NBN | | 0.16674 | 0.267181 | 0.238019 | 0.325258 | 0.250816 | 8.14E-05 |
| FASTK | | 0.186082 | 0.245263 | 0.215584 | 0.308227 | 0.237758 | 8.16E-05 |
| GUK1 | | 0.178265 | 0.330591 | 0.292165 | 0.37587 | 0.295974 | 8.2E-05 |
| TMX1 | | 0.201934 | 0.253135 | 0.220071 | 0.310866 | 0.239935 | 8.23E-05 |
| SDHB | | 0.179624 | 0.447085 | 0.394746 | 0.462701 | 0.375408 | 8.23E-05 |
| CCDC124 | | 0.163853 | 0.288876 | 0.257862 | 0.340609 | 0.260609 | 8.25E-05 |
| MESD | | 0.201981 | 0.234109 | 0.203524 | 0.286879 | 0.219804 | 8.29E-05 |
| NCBP2AS2 | | 0.164653 | 0.317176 | 0.282967 | 0.363636 | 0.285092 | 8.41E-05 |
| CNPPD1 | | 0.151965 | 0.734255 | 0.660847 | 0.648837 | 0.564744 | 8.44E-05 |
| TMEM179B | | 0.192978 | 0.371363 | 0.324868 | 0.399376 | 0.316649 | 8.45E-05 |
| LAGE3 | | 0.168321 | 0.197434 | 0.175692 | 0.247541 | 0.18444 | 8.45E-05 |
| TIMM50 | | 0.14097 | 0.209291 | 0.189808 | 0.265531 | 0.198585 | 8.58E-05 |
| SERBP1 | | 0.164618 | 0.647413 | 0.577599 | 0.626769 | 0.518498 | 8.58E-05 |
| TCF20 | | 0.218278 | 0.204291 | 0.175606 | 0.247781 | 0.18716 | 8.59E-05 |
| TARBP1 | | 0.191231 | 0.246983 | 0.216322 | 0.281362 | 0.215452 | 8.68E-05 |
| EIF4G1 | | 0.196857 | 0.319442 | 0.278697 | 0.369873 | 0.291621 | 8.97E-05 |
| SART1 | | 0.16569 | 0.425889 | 0.379681 | 0.465819 | 0.3716 | 8.98E-05 |
| SMC4 | | 0.152802 | 0.218875 | 0.196878 | 0.259535 | 0.195321 | 9.05E-05 |
| NDUFB8 | | 0.181478 | 0.557606 | 0.491697 | 0.53634 | 0.442873 | 9.07E-05 |
| ORMDL1 | | 0.146169 | 0.44987 | 0.406524 | 0.48261 | 0.387378 | 9.09E-05 |
| RANGAP1 | | 0.161964 | 0.186012 | 0.166259 | 0.240345 | 0.178455 | 9.22E-05 |
| ATF7 | | 0.150194 | 0.156408 | 0.140944 | 0.205325 | 0.148531 | 9.25E-05 |
| SLC35A1 | | 0.219638 | 0.173109 | 0.148662 | 0.223555 | 0.167573 | 9.25E-05 |
| HIF1A | | 0.301055 | 0.174428 | 0.141576 | 0.205565 | 0.153428 | 9.28E-05 |
| NDUFAF6 | | 0.226577 | 0.175335 | 0.149852 | 0.212041 | 0.157236 | 9.32E-05 |
| CDK2AP2 | | 0.221904 | 0.165239 | 0.141681 | 0.217558 | 0.161589 | 9.34E-05 |
| PLEKHG1 | | 0.182679 | 0.654594 | 0.57674 | 0.548093 | 0.466812 | 9.35E-05 |
| ZFAND2B | | 0.163113 | 0.40861 | 0.364928 | 0.429839 | 0.343308 | 9.43E-05 |
| TANGO2 | | 0.181959 | 0.209181 | 0.184394 | 0.254737 | 0.192601 | 9.46E-05 |
| RPP21 | | 0.190143 | 0.170451 | 0.149404 | 0.221156 | 0.163765 | 9.46E-05 |
| TYK2 | | 0.133417 | 0.52434 | 0.478025 | 0.541857 | 0.435256 | 9.49E-05 |
| ASCC3 | | 0.14234 | 0.282152 | 0.255643 | 0.341809 | 0.264418 | 9.5E-05 |
| UFL1 | | 0.171195 | 0.257001 | 0.228245 | 0.319021 | 0.247552 | 9.62E-05 |
| SELENOT | | 0.145339 | 0.288677 | 0.261012 | 0.3574 | 0.274755 | 9.62E-05 |
| TTN | | 0.228858 | 0.300989 | 0.256837 | 0.297673 | 0.23123 | 9.63E-05 |
| C17orf49 | | 0.186373 | 0.269858 | 0.237154 | 0.323579 | 0.250816 | 9.67E-05 |
| CCDC115 | | 0.176594 | 0.20586 | 0.182143 | 0.248021 | 0.186072 | 9.68E-05 |
| TBC1D20 | | 0.117641 | 0.249498 | 0.229961 | 0.312785 | 0.237214 | 9.71E-05 |
| IDS | | 0.158009 | 0.68891 | 0.617443 | 0.615735 | 0.515778 | 9.87E-05 |
| STARD3NL | | 0.210722 | 0.187329 | 0.161872 | 0.239626 | 0.181175 | 9.93E-05 |
| ST6GAL1 | | 0.111118 | 1.296455 | 1.200349 | 0.840249 | 0.784004 | 0.000101 |
| SEC23IP | | 0.135681 | 0.273778 | 0.249203 | 0.335092 | 0.257345 | 0.000101 |
| SPCS1 | | 0.214168 | 0.690102 | 0.594898 | 0.545455 | 0.478781 | 0.000101 |
| SHMT2 | | 0.156265 | 0.251505 | 0.225687 | 0.317582 | 0.243743 | 0.000101 |
| METTL4 | | 0.221749 | 0.160787 | 0.137879 | 0.191893 | 0.139282 | 0.000101 |
| SNRNP35 | | 0.143787 | 0.307408 | 0.278247 | 0.346846 | 0.270947 | 0.000101 |
| PDCL3 | | 0.173205 | 0.240056 | 0.212899 | 0.296474 | 0.226333 | 0.000103 |
| REXO2 | | 0.190429 | 0.408169 | 0.357697 | 0.382106 | 0.303591 | 0.000104 |
| TRAFD1 | | 0.138093 | 0.270884 | 0.246158 | 0.32142 | 0.245919 | 0.000104 |
| SLC30A5 | | 0.167239 | 0.191567 | 0.170599 | 0.253058 | 0.18988 | 0.000105 |
| ZNF292 | | 0.151907 | 0.40548 | 0.364957 | 0.441113 | 0.353101 | 0.000105 |
| SCYL3 | | 0.169836 | 0.160839 | 0.142977 | 0.208203 | 0.152339 | 0.000106 |
| RARS1 | | 0.113612 | 0.189783 | 0.175411 | 0.259055 | 0.193145 | 0.000106 |
| GMPR2 | | 0.170635 | 0.151785 | 0.134854 | 0.210602 | 0.154516 | 0.000107 |
| CDK9 | | 0.151341 | 0.508192 | 0.457582 | 0.524586 | 0.420566 | 0.000108 |
| GLYCTK | | 0.194606 | 0.312699 | 0.27324 | 0.346366 | 0.274211 | 0.000108 |
| SRP72 | | 0.174805 | 0.604244 | 0.535292 | 0.563684 | 0.475517 | 0.000108 |
| MICOS10 | | 0.173492 | 0.555624 | 0.492668 | 0.514512 | 0.430903 | 0.000109 |
| ATF6 | | 0.137519 | 0.275821 | 0.250744 | 0.335572 | 0.258977 | 0.000109 |
| MAN1B1 | | 0.190459 | 0.18509 | 0.1622 | 0.240345 | 0.180631 | 0.000109 |
| EIF4E2 | | 0.172781 | 0.486213 | 0.431335 | 0.496282 | 0.40642 | 0.000109 |
| TMEM35B | | 0.188913 | 0.147361 | 0.129275 | 0.183737 | 0.132209 | 0.00011 |
| DNAAF2 | | 0.174714 | 0.260416 | 0.230714 | 0.289038 | 0.223613 | 0.00011 |
| ZNF76 | | 0.14592 | 0.221753 | 0.200421 | 0.269609 | 0.204026 | 0.00011 |
| MED18 | | 0.186901 | 0.17718 | 0.155651 | 0.227153 | 0.16975 | 0.00011 |
| PSENEN | | 0.184506 | 0.296169 | 0.260614 | 0.33893 | 0.264418 | 0.000111 |
| AMPD2 | | 0.156849 | 0.19547 | 0.175333 | 0.244663 | 0.183351 | 0.000111 |
| SLC25A17 | | 0.199361 | 0.134715 | 0.117328 | 0.183018 | 0.132753 | 0.000111 |
| ZNF516 | | 0.257058 | 0.236712 | 0.198079 | 0.244903 | 0.186072 | 0.000111 |
| PSMD12 | | 0.155472 | 0.250702 | 0.22509 | 0.307508 | 0.235582 | 0.000112 |
| PNO1 | | 0.218783 | 0.181136 | 0.155648 | 0.228352 | 0.171382 | 0.000112 |
| GLE1 | | 0.157183 | 0.194104 | 0.174067 | 0.254977 | 0.192601 | 0.000113 |
| NFYC | | 0.175448 | 0.339525 | 0.300647 | 0.391701 | 0.311752 | 0.000114 |
| NFATC2IP | | 0.140466 | 0.384439 | 0.348773 | 0.42864 | 0.339499 | 0.000114 |
| USP38 | | 0.167001 | 0.244792 | 0.218035 | 0.305589 | 0.233406 | 0.000114 |
| MAD2L2 | | 0.217993 | 0.145164 | 0.124806 | 0.200768 | 0.146899 | 0.000115 |
| CLASRP | | 0.152301 | 0.626326 | 0.563577 | 0.601823 | 0.498912 | 0.000115 |
| ARIH1 | | 0.156792 | 0.501916 | 0.450228 | 0.524586 | 0.428183 | 0.000115 |
| ZFP36L2 | | 0.111376 | 1.580254 | 1.462848 | 0.878388 | 0.819913 | 0.000116 |
| MED26 | | 0.191593 | 0.216251 | 0.189358 | 0.263373 | 0.201306 | 0.000117 |
| SLC25A22 | | 0.160906 | 0.130576 | 0.116795 | 0.187575 | 0.136017 | 0.000117 |
| DNASE2 | | 0.178001 | 0.134568 | 0.118948 | 0.188295 | 0.136561 | 0.000118 |
| AKAP1 | | 0.203369 | 0.214918 | 0.18666 | 0.247781 | 0.187704 | 0.000118 |
| SNX15 | | 0.208901 | 0.219637 | 0.190029 | 0.257856 | 0.196409 | 0.000119 |
| RO60 | | 0.146828 | 0.276597 | 0.249832 | 0.338211 | 0.262786 | 0.00012 |
| CSTF3 | | 0.139681 | 0.341785 | 0.310246 | 0.388582 | 0.305223 | 0.000123 |
| FTSJ1 | | 0.148162 | 0.138625 | 0.125095 | 0.19717 | 0.14309 | 0.000125 |
| WIZ | | 0.144286 | 0.300929 | 0.272289 | 0.349484 | 0.273123 | 0.000125 |
| SCRIB | | 0.184907 | 0.254791 | 0.224141 | 0.308227 | 0.239391 | 0.000126 |
| ODR4 | | 0.119437 | 0.193009 | 0.177674 | 0.251859 | 0.187704 | 0.000126 |
| FOXJ3 | | 0.116506 | 0.244157 | 0.225215 | 0.30319 | 0.230686 | 0.000127 |
| MARCHF3 | | 0.193798 | 0.410576 | 0.358967 | 0.389542 | 0.315016 | 0.000128 |
| DDX39A | | 0.172734 | 0.442947 | 0.392965 | 0.457184 | 0.366703 | 0.000128 |
| SIVA1 | | 0.1726 | 0.449134 | 0.398491 | 0.4651 | 0.373776 | 0.000128 |
| ZNF513 | | 0.155635 | 0.168642 | 0.151396 | 0.21444 | 0.15778 | 0.000128 |
| MEN1 | | 0.16966 | 0.259179 | 0.230424 | 0.308467 | 0.237214 | 0.000128 |
| SEL1L | | 0.196984 | 0.358319 | 0.312587 | 0.366035 | 0.291077 | 0.000129 |
| ZSCAN29 | | 0.186033 | 0.16169 | 0.142129 | 0.208923 | 0.153972 | 0.000129 |
| UBQLN1 | | 0.168736 | 0.187459 | 0.166768 | 0.253058 | 0.190424 | 0.000129 |
| ACO2 | | 0.142442 | 0.32795 | 0.297118 | 0.378508 | 0.293254 | 0.000129 |
| LMAN1 | | 0.349338 | 0.35238 | 0.276599 | 0.269369 | 0.218172 | 0.00013 |
| VKORC1 | | 0.168645 | 0.272844 | 0.242744 | 0.323099 | 0.25136 | 0.00013 |
| PLEKHJ1 | | 0.13758 | 0.320337 | 0.2912 | 0.377309 | 0.293254 | 0.000131 |
| RGCC | | 0.197736 | 0.451914 | 0.394032 | 0.384505 | 0.31556 | 0.000131 |
| MYCBP | | 0.166784 | 0.139609 | 0.124368 | 0.197409 | 0.144178 | 0.000132 |
| LDB1 | | 0.147517 | 0.609511 | 0.550269 | 0.580235 | 0.477693 | 0.000133 |
| TOP1 | | 0.175672 | 0.518786 | 0.45931 | 0.506836 | 0.420566 | 0.000134 |
| GCA | | 0.181877 | 0.279428 | 0.246331 | 0.293835 | 0.225789 | 0.000135 |
| QSER1 | | 0.187807 | 0.148895 | 0.130721 | 0.200528 | 0.147443 | 0.000135 |
| ABHD3 | | 0.19012 | 0.188719 | 0.165419 | 0.247302 | 0.187704 | 0.000135 |
| FBH1 | | 0.175955 | 0.43909 | 0.388674 | 0.444951 | 0.360174 | 0.000137 |
| TOR1AIP2 | | 0.116969 | 0.216116 | 0.199285 | 0.281123 | 0.212731 | 0.000137 |
| TSR1 | | 0.189264 | 0.190016 | 0.166654 | 0.238426 | 0.178999 | 0.000137 |
| PRMT7 | | 0.255088 | 0.151322 | 0.126798 | 0.194771 | 0.143634 | 0.000137 |
| STARD3 | | 0.163954 | 0.369874 | 0.33014 | 0.419525 | 0.334603 | 0.000138 |
| ERCC3 | | 0.150728 | 0.323382 | 0.291301 | 0.383545 | 0.302503 | 0.000139 |
| RIT1 | | 0.162612 | 0.210998 | 0.188507 | 0.256656 | 0.195321 | 0.000139 |
| TMEM87B | | 0.205491 | 0.178501 | 0.154803 | 0.23291 | 0.175734 | 0.000139 |
| ROCK2 | | 0.262893 | 0.153846 | 0.128218 | 0.200048 | 0.148531 | 0.00014 |
| PGRMC2 | | 0.17284 | 0.395248 | 0.350622 | 0.416167 | 0.332427 | 0.00014 |
| RNASET2 | | 0.159694 | 0.588429 | 0.526771 | 0.565363 | 0.470076 | 0.000142 |
| MBD1 | | 0.212344 | 0.181258 | 0.15645 | 0.247062 | 0.188792 | 0.000142 |
| NAA38 | | 0.160476 | 0.352008 | 0.314952 | 0.396258 | 0.310664 | 0.000142 |
| CENPM | | 0.203343 | 0.483307 | 0.419769 | 0.406332 | 0.335147 | 0.000143 |
| BCL3 | | 0.189858 | 0.285124 | 0.249966 | 0.32166 | 0.252992 | 0.000143 |
| COMMD1 | | 0.150908 | 0.281992 | 0.253985 | 0.337971 | 0.261697 | 0.000143 |
| TBL1XR1 | | 0.160126 | 0.549227 | 0.491529 | 0.549532 | 0.455386 | 0.000144 |
| TASL | | 0.18953 | 0.29206 | 0.256105 | 0.315663 | 0.248096 | 0.000145 |
| IRAK4 | | 0.160943 | 0.375971 | 0.336284 | 0.418326 | 0.331882 | 0.000145 |
| SLIRP | | 0.179293 | 0.128341 | 0.113343 | 0.189974 | 0.138194 | 0.000145 |
| NKTR | | 0.130742 | 0.467835 | 0.427302 | 0.496522 | 0.397715 | 0.000145 |
| PPP3CB | | 0.155106 | 0.381794 | 0.342877 | 0.42816 | 0.34222 | 0.000146 |
| PRDX2 | | 0.198324 | 0.356123 | 0.310384 | 0.349244 | 0.276931 | 0.000146 |
| MOB3B | | 0.195551 | 0.401439 | 0.350552 | 0.369633 | 0.29815 | 0.000147 |
| SYNGR3 | | 1.032957 | 0.033014 | 0.016134 | 0.041257 | 0.019587 | 0.000149 |
| EPS15 | | 0.148938 | 0.390538 | 0.352232 | 0.439194 | 0.348205 | 0.000149 |
| NELFB | | 0.137002 | 0.371579 | 0.337917 | 0.419525 | 0.328074 | 0.000149 |
| PURB | | 0.144103 | 0.401161 | 0.363027 | 0.42864 | 0.34222 | 0.00015 |
| IFNGR1 | | 0.215133 | 0.257094 | 0.221478 | 0.28544 | 0.22198 | 0.00015 |
| DPYSL2 | | 0.219867 | 0.24639 | 0.211561 | 0.272967 | 0.213819 | 0.000151 |
| AKR7A2 | | 0.236166 | 0.163705 | 0.138985 | 0.219717 | 0.164309 | 0.000152 |
| CISD2 | | 0.194056 | 0.223434 | 0.195314 | 0.277285 | 0.213275 | 0.000152 |
| MFSD14A | | 0.137527 | 0.302675 | 0.275155 | 0.368913 | 0.286725 | 0.000153 |
| NOP2 | | 0.133593 | 0.258439 | 0.235582 | 0.302471 | 0.231774 | 0.000153 |
| ZBTB1 | | 0.141986 | 0.753436 | 0.682818 | 0.66107 | 0.564744 | 0.000154 |
| CASP4 | | 0.144325 | 0.562398 | 0.508859 | 0.556488 | 0.458651 | 0.000156 |
| TPP2 | | 0.204385 | 0.361646 | 0.313875 | 0.395059 | 0.317193 | 0.000157 |
| DMAP1 | | 0.139719 | 0.287016 | 0.260523 | 0.33869 | 0.26333 | 0.000157 |
| MAN2A1 | | 0.175825 | 0.450784 | 0.399062 | 0.469177 | 0.384657 | 0.00016 |
| BORCS5 | | 0.165934 | 0.223746 | 0.199436 | 0.278484 | 0.213275 | 0.00016 |
| NF2 | | 0.15922 | 0.26144 | 0.234122 | 0.317582 | 0.247008 | 0.000161 |
| FBXL20 | | 0.223999 | 0.200695 | 0.171833 | 0.234349 | 0.176823 | 0.000162 |
| ABL1 | | 0.18468 | 0.190303 | 0.167437 | 0.243224 | 0.185528 | 0.000162 |
| TRIP11 | | 0.164067 | 0.215899 | 0.192691 | 0.269609 | 0.206202 | 0.000162 |
| ARPC5 | | 0.142532 | 0.312305 | 0.282926 | 0.382826 | 0.299782 | 0.000162 |
| GALNT2 | | 0.19571 | 0.250746 | 0.218937 | 0.291437 | 0.225789 | 0.000162 |
| MBD3 | | 0.151164 | 0.185579 | 0.167118 | 0.245622 | 0.184984 | 0.000162 |
| CTNNB1 | | 0.159769 | 0.279831 | 0.250496 | 0.342768 | 0.268226 | 0.000162 |
| NOL7 | | 0.165347 | 0.189026 | 0.168557 | 0.260254 | 0.196953 | 0.000163 |
| SNRPB | | 0.1646 | 0.538381 | 0.480331 | 0.538498 | 0.440696 | 0.000163 |
| NUDC | | 0.160541 | 0.537772 | 0.481139 | 0.532982 | 0.437432 | 0.000163 |
| SZRD1 | | 0.161556 | 0.614561 | 0.549455 | 0.610938 | 0.502176 | 0.000164 |
| ACD | | 0.182888 | 0.20965 | 0.184688 | 0.250899 | 0.191513 | 0.000164 |
| SLC52A2 | | 0.184986 | 0.171476 | 0.15084 | 0.228112 | 0.171382 | 0.000165 |
| KPNA1 | | 0.16292 | 0.313398 | 0.279931 | 0.366035 | 0.289445 | 0.000165 |
| MCRS1 | | 0.145634 | 0.495598 | 0.448012 | 0.493164 | 0.399891 | 0.000166 |
| METTL5 | | 0.138124 | 0.234645 | 0.213222 | 0.289278 | 0.221436 | 0.000166 |
| GPR18 | | 0.188891 | 0.663803 | 0.582341 | 0.523147 | 0.454842 | 0.000166 |
| ACOX1 | | 0.204128 | 0.14355 | 0.12461 | 0.203166 | 0.150707 | 0.000167 |
| RILP | | 2.104031 | 0.012398 | 0.002884 | 0.018949 | 0.004897 | 0.000167 |
| MRS2 | | 0.137377 | 0.205557 | 0.186887 | 0.260734 | 0.198585 | 0.000167 |
| FAR1 | | 0.167947 | 0.303943 | 0.270542 | 0.359319 | 0.282372 | 0.000168 |
| TESK1 | | 0.223122 | 0.212476 | 0.182031 | 0.244663 | 0.18716 | 0.000168 |
| IKZF5 | | 0.148414 | 0.233863 | 0.211001 | 0.288558 | 0.22198 | 0.000169 |
| SLC10A7 | | 0.185971 | 0.131175 | 0.11531 | 0.186376 | 0.136017 | 0.000169 |
| CLINT1 | | 0.171913 | 0.492724 | 0.437374 | 0.492924 | 0.402612 | 0.000169 |
| PEF1 | | 0.147711 | 0.206689 | 0.186574 | 0.271288 | 0.205658 | 0.00017 |
| LIX1L | | 0.147463 | 0.340609 | 0.307514 | 0.380427 | 0.302503 | 0.00017 |
| RIC1 | | 0.150676 | 0.481477 | 0.433728 | 0.505877 | 0.409684 | 0.000172 |
| KPNA3 | | 0.146608 | 0.236068 | 0.213257 | 0.300792 | 0.230141 | 0.000172 |
| PPP2R5E | | 0.16593 | 0.334766 | 0.298395 | 0.384745 | 0.305767 | 0.000174 |
| ZMIZ2 | | 0.13651 | 0.602446 | 0.548056 | 0.564404 | 0.46518 | 0.000174 |
| TMEM208 | | 0.260459 | 0.192052 | 0.160329 | 0.230271 | 0.176279 | 0.000174 |
| SMARCC1 | | 0.149316 | 0.463751 | 0.418154 | 0.501319 | 0.407508 | 0.000175 |
| SF3B2 | | 0.166848 | 0.346824 | 0.308946 | 0.413289 | 0.326986 | 0.000178 |
| GOLGA2 | | 0.205717 | 0.137618 | 0.119329 | 0.190933 | 0.140914 | 0.000179 |
| DELE1 | | 0.145153 | 0.365676 | 0.330675 | 0.404174 | 0.32481 | 0.000179 |
| HTT | | 0.161057 | 0.420237 | 0.375847 | 0.449268 | 0.362894 | 0.000181 |
| DPY30 | | 0.16132 | 0.25204 | 0.225376 | 0.311586 | 0.244287 | 0.000181 |
| NUDT16L1 | | 0.165189 | 0.255509 | 0.227866 | 0.30439 | 0.236126 | 0.000183 |
| HDHD3 | | 0.151724 | 0.155589 | 0.140057 | 0.200768 | 0.147443 | 0.000183 |
| FUT8 | | 0.162403 | 0.382229 | 0.341535 | 0.398657 | 0.317737 | 0.000184 |
| FEM1C | | 0.180241 | 0.188181 | 0.16608 | 0.245862 | 0.18716 | 0.000185 |
| UBE4A | | 0.165706 | 0.404668 | 0.360759 | 0.444951 | 0.355277 | 0.000185 |
| EIF4E | | 0.10747 | 0.182187 | 0.169109 | 0.24946 | 0.18716 | 0.000186 |
| DENR | | 0.149834 | 0.267675 | 0.24127 | 0.327657 | 0.256257 | 0.000188 |
| UBE2M | | 0.151401 | 0.145892 | 0.131358 | 0.210122 | 0.155604 | 0.000188 |
| CHL1 | | 1.614299 | 0.023081 | 0.007539 | 0.023267 | 0.007617 | 0.000189 |
| SSNA1 | | 0.140667 | 0.292091 | 0.264956 | 0.351403 | 0.273123 | 0.000189 |
| SEMA4D | | 0.150934 | 0.29176 | 0.262779 | 0.353802 | 0.277476 | 0.000189 |
| ROMO1 | | 0.178513 | 0.350455 | 0.309667 | 0.380907 | 0.302503 | 0.000189 |
| ACOT9 | | 0.194659 | 0.204767 | 0.178921 | 0.251619 | 0.192601 | 0.00019 |
| CELSR1 | | 0.225224 | 0.225846 | 0.193203 | 0.240345 | 0.182807 | 0.000192 |
| NAA10 | | 0.150607 | 0.340883 | 0.307092 | 0.385704 | 0.304135 | 0.000192 |
| ABHD2 | | 0.211173 | 0.142543 | 0.123133 | 0.194051 | 0.143634 | 0.000193 |
| YTHDF2 | | 0.155829 | 0.460003 | 0.412906 | 0.491964 | 0.398259 | 0.000194 |
| ZNRF1 | | 1.083863 | 0.009348 | 0.00441 | 0.028304 | 0.010881 | 0.000195 |
| SS18 | | 0.152495 | 0.394552 | 0.354976 | 0.439434 | 0.352013 | 0.000195 |
| ACAP3 | | 0.172751 | 0.247041 | 0.219163 | 0.292636 | 0.228509 | 0.000196 |
| WSB2 | | 0.119967 | 0.135527 | 0.124713 | 0.186136 | 0.134929 | 0.000196 |
| SHKBP1 | | 0.142326 | 0.537115 | 0.486657 | 0.551451 | 0.446681 | 0.000197 |
| TMCO1 | | 0.164788 | 0.485738 | 0.433307 | 0.461502 | 0.378672 | 0.000197 |
| SOCS4 | | 0.176528 | 0.219425 | 0.194154 | 0.272727 | 0.209467 | 0.000197 |
| EFTUD2 | | 0.155808 | 0.390134 | 0.350196 | 0.436556 | 0.347116 | 0.000198 |
| U2AF2 | | 0.134288 | 0.236768 | 0.215724 | 0.305109 | 0.235038 | 0.000199 |
| MRNIP | | 0.173683 | 0.324608 | 0.28779 | 0.331734 | 0.261697 | 0.0002 |
| SPECC1L | | 0.163213 | 0.398374 | 0.355761 | 0.437755 | 0.353645 | 0.0002 |
| AHCYL1 | | 0.161272 | 0.228198 | 0.204063 | 0.283761 | 0.218716 | 0.0002 |
| USP6NL | | 0.14416 | 0.724247 | 0.655376 | 0.644519 | 0.559848 | 0.0002 |
| CLPTM1L | | 0.203232 | 0.48016 | 0.417068 | 0.437515 | 0.36235 | 0.0002 |
| WWOX | | 0.170282 | 0.226198 | 0.201016 | 0.270568 | 0.208923 | 0.0002 |
| NXF1 | | 0.138712 | 0.53789 | 0.488581 | 0.552171 | 0.45049 | 0.0002 |
| MSRA | | 0.197769 | 0.221759 | 0.193351 | 0.248981 | 0.192057 | 0.0002 |
| SEC11C | | 0.321692 | 0.502021 | 0.401683 | 0.324778 | 0.266594 | 0.0002 |
| TMEM167A | | 0.157898 | 0.371668 | 0.333137 | 0.418326 | 0.33025 | 0.0002 |
| EPOP | | 2.027875 | 0.009999 | 0.002452 | 0.01871 | 0.004897 | 0.000201 |
| SEC24A | | 0.224508 | 0.280996 | 0.2405 | 0.2852 | 0.223613 | 0.000201 |
| SLC5A3 | | 0.133832 | 0.200204 | 0.182467 | 0.253778 | 0.193145 | 0.000202 |
| CCRL2 | | 1.581769 | 0.014759 | 0.004931 | 0.022308 | 0.007073 | 0.000202 |
| STK17A | | 0.138922 | 0.773154 | 0.702176 | 0.638043 | 0.543526 | 0.000203 |
| SELENOO | | 0.178152 | 0.214055 | 0.189189 | 0.259535 | 0.198041 | 0.000205 |
| MTRR | | 0.113878 | 0.153368 | 0.141728 | 0.21468 | 0.158324 | 0.000206 |
| SCARB2 | | 0.216263 | 0.182052 | 0.156709 | 0.224274 | 0.16975 | 0.000207 |
| LSM2 | | 0.177291 | 0.333583 | 0.295008 | 0.376349 | 0.301415 | 0.000208 |
| SEM1 | | 0.158974 | 0.15875 | 0.142186 | 0.226673 | 0.169206 | 0.000209 |
| GDF7 | | 1.753839 | 0.014444 | 0.004283 | 0.01775 | 0.004353 | 0.000209 |
| SLC66A2 | | 0.231633 | 0.270405 | 0.230296 | 0.297433 | 0.235582 | 0.000211 |
| CUL5 | | 0.165966 | 0.28669 | 0.255536 | 0.340609 | 0.268226 | 0.000211 |
| PHF23 | | 0.150611 | 0.206202 | 0.185761 | 0.258815 | 0.199129 | 0.000211 |
| PARL | | 0.153091 | 0.193032 | 0.173598 | 0.259295 | 0.197497 | 0.000212 |
| BLOC1S6 | | 0.153902 | 0.198771 | 0.178658 | 0.257856 | 0.196409 | 0.000213 |
| GLRX | | 0.157793 | 0.327158 | 0.293263 | 0.359559 | 0.282916 | 0.000214 |
| FBXO46 | | 0.133541 | 0.322291 | 0.293798 | 0.379467 | 0.299238 | 0.000214 |
| SF3B4 | | 0.157343 | 0.267364 | 0.239739 | 0.334133 | 0.262242 | 0.000214 |
| ELK4 | | 0.146696 | 0.598584 | 0.540711 | 0.580715 | 0.480413 | 0.000215 |
| MIB1 | | 0.199856 | 0.17755 | 0.154582 | 0.224035 | 0.169206 | 0.000215 |
| ILRUN | | 0.145273 | 0.784263 | 0.709137 | 0.686256 | 0.589227 | 0.000216 |
| CLIP1 | | 0.149664 | 0.271753 | 0.244974 | 0.327657 | 0.256801 | 0.000216 |
| CHAC1 | | 1.011399 | 0.005972 | 0.002963 | 0.016791 | 0.003808 | 0.000218 |
| RICTOR | | 0.181304 | 0.255569 | 0.225388 | 0.294315 | 0.229053 | 0.000219 |
| NDUFB7 | | 0.154051 | 0.652815 | 0.5867 | 0.573279 | 0.484766 | 0.000222 |
| MAP4K1 | | 0.143939 | 0.740206 | 0.66992 | 0.645239 | 0.54679 | 0.000222 |
| DAPP1 | | 0.128364 | 0.942296 | 0.862076 | 0.746942 | 0.657236 | 0.000222 |
| RHOBTB2 | | 0.184617 | 0.17214 | 0.151463 | 0.198609 | 0.147443 | 0.000225 |
| SNIP1 | | 0.151317 | 0.239912 | 0.216023 | 0.30271 | 0.233406 | 0.000227 |
| NOL8 | | 0.174808 | 0.167434 | 0.148327 | 0.21444 | 0.160501 | 0.000227 |
| DAPK3 | | 0.148532 | 0.242 | 0.218325 | 0.299592 | 0.232318 | 0.000227 |
| KLC2 | | 0.143159 | 0.182127 | 0.164922 | 0.235068 | 0.176823 | 0.000229 |
| TMEM134 | | 0.13808 | 0.486582 | 0.442171 | 0.493164 | 0.399347 | 0.000229 |
| SDE2 | | 0.163039 | 0.327951 | 0.292907 | 0.380187 | 0.302503 | 0.000229 |
| TUFM | | 0.162217 | 0.664689 | 0.594 | 0.593428 | 0.5 | 0.000229 |
| UQCRQ | | 0.206679 | 0.252278 | 0.218607 | 0.299112 | 0.233406 | 0.00023 |
| ARAF | | 0.155572 | 0.389546 | 0.349725 | 0.435116 | 0.348749 | 0.00023 |
| VAT1 | | 0.162263 | 0.23469 | 0.209724 | 0.280643 | 0.217084 | 0.000232 |
| SLC35C1 | | 0.157978 | 0.13782 | 0.123525 | 0.194051 | 0.14309 | 0.000234 |
| COL19A1 | | 0.181889 | 0.469857 | 0.414201 | 0.419045 | 0.346572 | 0.000237 |
| HSPD1 | | 0.154737 | 0.334749 | 0.300704 | 0.380907 | 0.300871 | 0.000238 |
| RILPL2 | | 0.182094 | 0.380575 | 0.335447 | 0.35788 | 0.286181 | 0.000238 |
| RAB8B | | 0.150703 | 0.434232 | 0.391161 | 0.454785 | 0.370511 | 0.000238 |
| ZW10 | | 0.120528 | 0.130818 | 0.120333 | 0.180139 | 0.130033 | 0.00024 |
| AKAP17A | | 0.134238 | 0.93394 | 0.85096 | 0.749101 | 0.658868 | 0.00024 |
| NIT1 | | 0.1488 | 0.196058 | 0.176844 | 0.247302 | 0.188792 | 0.000241 |
| RNF44 | | 0.123857 | 0.940297 | 0.86294 | 0.718638 | 0.645811 | 0.000241 |
| SS18L2 | | 0.146849 | 0.273359 | 0.246903 | 0.332694 | 0.261697 | 0.000242 |
| PDHX | | 0.163737 | 0.14327 | 0.127899 | 0.197649 | 0.146355 | 0.000242 |
| SGTA | | 0.144224 | 0.253528 | 0.229409 | 0.307988 | 0.238847 | 0.000245 |
| OSBPL9 | | 0.143539 | 0.389682 | 0.352778 | 0.432718 | 0.343308 | 0.000245 |
| SRP68 | | 0.190435 | 0.363337 | 0.318407 | 0.376589 | 0.303591 | 0.000246 |
| AKR1B1 | | 0.163228 | 0.369846 | 0.330282 | 0.390981 | 0.315016 | 0.000246 |
| PDK2 | | 0.167237 | 0.190464 | 0.169617 | 0.242504 | 0.186072 | 0.000246 |
| SAMD4B | | 0.148187 | 0.218939 | 0.197567 | 0.277285 | 0.214363 | 0.000247 |
| SNX20 | | 0.145616 | 0.315859 | 0.285535 | 0.355721 | 0.281284 | 0.000247 |
| SH3YL1 | | 0.202625 | 0.199566 | 0.173416 | 0.219237 | 0.165397 | 0.000247 |
| RALBP1 | | 0.14334 | 0.318511 | 0.288387 | 0.37491 | 0.297606 | 0.000247 |
| WWP2 | | 0.116286 | 0.345257 | 0.31852 | 0.387863 | 0.307399 | 0.000249 |
| ABR | | 0.192647 | 0.251243 | 0.219838 | 0.273927 | 0.212731 | 0.000251 |
| SECISBP2L | | 0.153067 | 0.565169 | 0.508277 | 0.557448 | 0.46518 | 0.000251 |
| COPB2 | | 0.191169 | 0.419224 | 0.367197 | 0.413528 | 0.335691 | 0.000252 |
| DERL3 | | 0.313065 | 0.278499 | 0.224172 | 0.238187 | 0.187704 | 0.000253 |
| SFI1 | | 0.150785 | 0.216304 | 0.194838 | 0.264092 | 0.20185 | 0.000253 |
| STOML2 | | 0.187647 | 0.379508 | 0.333222 | 0.395778 | 0.321545 | 0.000254 |
| IFT27 | | 0.207251 | 0.143909 | 0.124652 | 0.19597 | 0.145811 | 0.000254 |
| CNOT9 | | 0.151815 | 0.318307 | 0.286513 | 0.372991 | 0.29543 | 0.000255 |
| WDR46 | | 0.136542 | 0.244632 | 0.222541 | 0.300552 | 0.231774 | 0.000255 |
| PPM1G | | 0.168747 | 0.441459 | 0.392729 | 0.462461 | 0.37432 | 0.000255 |
| GRSF1 | | 0.117232 | 0.274107 | 0.252715 | 0.345407 | 0.267138 | 0.000255 |
| OSBPL2 | | 0.155148 | 0.197751 | 0.177589 | 0.248741 | 0.189336 | 0.000256 |
| URM1 | | 0.122293 | 0.219582 | 0.201736 | 0.288798 | 0.220892 | 0.000256 |
| RAB7A | | 0.122617 | 0.284246 | 0.261085 | 0.359079 | 0.280196 | 0.000257 |
| MDH2 | | 0.155354 | 0.340337 | 0.305592 | 0.399856 | 0.318281 | 0.000257 |
| SMC3 | | 0.134478 | 0.331064 | 0.301599 | 0.37467 | 0.293798 | 0.000258 |
| TBCD | | 0.128367 | 0.236404 | 0.216278 | 0.294075 | 0.227965 | 0.000261 |
| SSRP1 | | 0.163036 | 0.394593 | 0.352429 | 0.418805 | 0.335147 | 0.000262 |
| CPNE1 | | 0.145927 | 0.786909 | 0.711207 | 0.669465 | 0.571817 | 0.000262 |
| CBLL1 | | 0.172854 | 0.169827 | 0.150651 | 0.216839 | 0.163221 | 0.000265 |
| COX7B | | 0.16115 | 0.226203 | 0.202296 | 0.291197 | 0.224157 | 0.000265 |
| XRN2 | | 0.162091 | 0.362665 | 0.324124 | 0.406093 | 0.32481 | 0.000265 |
| TRPM7 | | 0.135468 | 0.344506 | 0.31363 | 0.396018 | 0.317193 | 0.000265 |
| SLC66A3 | | 0.156814 | 0.153548 | 0.137733 | 0.202447 | 0.150707 | 0.000267 |
| RAB6A | | 0.160321 | 0.315055 | 0.281919 | 0.37419 | 0.29543 | 0.000267 |
| TOP3B | | 0.153467 | 0.214463 | 0.192821 | 0.259055 | 0.196409 | 0.000268 |
| ARHGDIA | | 0.151263 | 0.647581 | 0.583122 | 0.606141 | 0.507617 | 0.00027 |
| DCTN2 | | 0.192732 | 0.354187 | 0.309895 | 0.389302 | 0.31556 | 0.000271 |
| HRAS | | 0.150824 | 0.183376 | 0.165173 | 0.230271 | 0.173558 | 0.000272 |
| PDIA6 | | 0.246785 | 0.35514 | 0.299302 | 0.337731 | 0.275843 | 0.000272 |
| ELF4 | | 0.16547 | 0.257453 | 0.229555 | 0.283521 | 0.218716 | 0.000274 |
| AUP1 | | 0.172944 | 0.456063 | 0.404542 | 0.467498 | 0.381937 | 0.000274 |
| PBDC1 | | 0.136524 | 0.180596 | 0.16429 | 0.237467 | 0.179543 | 0.000275 |
| WDR75 | | 0.185404 | 0.164318 | 0.144502 | 0.217798 | 0.163765 | 0.000275 |
| LRRC59 | | 0.197657 | 0.476732 | 0.415694 | 0.435836 | 0.363983 | 0.000277 |
| EXOSC10 | | 0.156423 | 0.290289 | 0.260461 | 0.341569 | 0.269314 | 0.000277 |
| ERLEC1 | | 0.222186 | 0.363296 | 0.311441 | 0.34013 | 0.277476 | 0.000277 |
| MED13L | | 0.16395 | 0.624534 | 0.557445 | 0.591269 | 0.49728 | 0.000278 |
| RBCK1 | | 0.168502 | 0.260551 | 0.23183 | 0.30415 | 0.238847 | 0.000278 |
| INSIG1 | | 0.140584 | 0.435604 | 0.395159 | 0.444471 | 0.361262 | 0.000279 |
| ZDHHC4 | | 0.143135 | 0.216316 | 0.195885 | 0.275846 | 0.212731 | 0.000281 |
| THOC2 | | 0.224953 | 0.188078 | 0.160924 | 0.239386 | 0.184984 | 0.000281 |
| CDK5RAP3 | | 0.13664 | 0.934119 | 0.849708 | 0.746462 | 0.655604 | 0.000282 |
| AP1G1 | | 0.142107 | 0.281363 | 0.254969 | 0.343008 | 0.269314 | 0.000282 |
| PAFAH1B3 | | 0.165731 | 0.160791 | 0.143342 | 0.207244 | 0.15506 | 0.000284 |
| SMG1 | | 0.259278 | 0.181991 | 0.152055 | 0.218757 | 0.167573 | 0.000285 |
| ZNFX1 | | 0.181031 | 0.234194 | 0.206576 | 0.283521 | 0.220348 | 0.000287 |
| FDPS | | 0.146331 | 0.145483 | 0.13145 | 0.209643 | 0.156692 | 0.000287 |
| RMDN1 | | 0.15354 | 0.176033 | 0.158261 | 0.234589 | 0.178455 | 0.000288 |
| CAP1 | | 0.141415 | 0.308777 | 0.279946 | 0.37515 | 0.297062 | 0.000289 |
| KRT10 | | 0.174228 | 0.295064 | 0.261498 | 0.366994 | 0.292709 | 0.00029 |
| RNASE6 | | 0.155542 | 0.539796 | 0.484626 | 0.496042 | 0.412949 | 0.000291 |
| SIRT7 | | 0.147626 | 0.250768 | 0.226377 | 0.298633 | 0.233406 | 0.000291 |
| TDP1 | | 0.139338 | 0.204098 | 0.185308 | 0.259055 | 0.199129 | 0.000292 |
| DDX23 | | 0.134149 | 0.435181 | 0.39654 | 0.474934 | 0.381937 | 0.000293 |
| SMAP1 | | 0.138296 | 0.188179 | 0.170978 | 0.247781 | 0.189336 | 0.000295 |
| COPG1 | | 0.18221 | 0.503216 | 0.443511 | 0.469417 | 0.392274 | 0.000296 |
| MAP3K14 | | 0.176045 | 0.358989 | 0.317751 | 0.382826 | 0.309032 | 0.000298 |
| FAM76B | | 0.157125 | 0.241983 | 0.217013 | 0.2852 | 0.221436 | 0.000299 |
| SPAG7 | | 0.151764 | 0.368315 | 0.331539 | 0.405613 | 0.32753 | 0.000302 |
| HSPA5 | | 0.240399 | 0.373506 | 0.316177 | 0.346606 | 0.28346 | 0.000303 |
| RNF145 | | 0.160874 | 0.311529 | 0.278657 | 0.361478 | 0.288357 | 0.000303 |
| GOLGA3 | | 0.157253 | 0.343389 | 0.307927 | 0.388582 | 0.312296 | 0.000304 |
| PROSER1 | | 0.156842 | 0.199033 | 0.17853 | 0.266011 | 0.20457 | 0.000304 |
| DDHD2 | | 0.122863 | 0.322346 | 0.296031 | 0.362197 | 0.285637 | 0.000305 |
| TIMP2 | | 0.190628 | 0.264428 | 0.231698 | 0.281123 | 0.220892 | 0.000306 |
| NECAP2 | | 0.149759 | 0.489656 | 0.441376 | 0.492444 | 0.411317 | 0.000308 |
| G3BP2 | | 0.147017 | 0.579909 | 0.523725 | 0.565124 | 0.468444 | 0.00031 |
| HDHD5 | | 0.144573 | 0.21234 | 0.192093 | 0.261454 | 0.20185 | 0.000316 |
| RNPC3 | | 0.187123 | 0.174732 | 0.153477 | 0.203646 | 0.151795 | 0.000316 |
| SNX4 | | 0.117499 | 0.248169 | 0.228758 | 0.307028 | 0.238847 | 0.000317 |
| BRD1 | | 0.136168 | 0.595575 | 0.541934 | 0.580715 | 0.480413 | 0.000318 |
| ENTPD4 | | 0.134932 | 0.364925 | 0.332342 | 0.417846 | 0.331338 | 0.00032 |
| ACTR1A | | 0.159343 | 0.358503 | 0.321015 | 0.408731 | 0.328074 | 0.00032 |
| RNF214 | | 0.192702 | 0.167785 | 0.146806 | 0.209163 | 0.157236 | 0.000324 |
| ZDHHC3 | | 0.161958 | 0.220784 | 0.197339 | 0.28568 | 0.224157 | 0.000325 |
| PSMD1 | | 0.163107 | 0.202716 | 0.181046 | 0.261214 | 0.200218 | 0.000326 |
| TMEM129 | | 0.140396 | 0.19498 | 0.176899 | 0.245143 | 0.18716 | 0.000326 |
| EI24 | | 0.1339 | 0.172267 | 0.156998 | 0.233149 | 0.176279 | 0.000327 |
| SMARCD1 | | 0.141363 | 0.321048 | 0.291082 | 0.372272 | 0.29815 | 0.000327 |
| VAMP7 | | 0.128371 | 0.225761 | 0.206541 | 0.28568 | 0.221436 | 0.000328 |
| FIS1 | | 0.148823 | 0.638575 | 0.575986 | 0.588391 | 0.499456 | 0.000329 |
| RIOX2 | | 0.111957 | 0.26743 | 0.247461 | 0.319021 | 0.249728 | 0.00033 |
| RMND5B | | 0.149448 | 0.18225 | 0.164316 | 0.234589 | 0.177911 | 0.000333 |
| DLL1 | | 1.49516 | 0.025774 | 0.009143 | 0.027345 | 0.010881 | 0.000333 |
| MARCHF1 | | 0.141452 | 0.900051 | 0.815992 | 0.655073 | 0.587595 | 0.000334 |
| SPAG9 | | 0.125763 | 0.207569 | 0.190241 | 0.26721 | 0.205114 | 0.000334 |
| AKT2 | | 0.151974 | 0.459415 | 0.413482 | 0.496282 | 0.402067 | 0.000335 |
| SLC15A3 | | 0.223549 | 0.26793 | 0.22947 | 0.278484 | 0.218716 | 0.000335 |
| FCGRT | | 0.172867 | 0.165416 | 0.146737 | 0.193811 | 0.143634 | 0.000336 |
| PI4K2B | | 0.113143 | 0.358389 | 0.331357 | 0.403694 | 0.317193 | 0.000343 |
| PPP2R2A | | 0.118645 | 0.280527 | 0.25838 | 0.343008 | 0.269314 | 0.000343 |
| NUP133 | | 0.146452 | 0.256223 | 0.23149 | 0.307988 | 0.241023 | 0.000343 |
| PIEZO2 | | 4.484629 | 0.003273 | 0.000146 | 0.009834 | 0.000544 | 0.000344 |
| WDR45B | | 0.183451 | 0.207584 | 0.182797 | 0.257376 | 0.198585 | 0.000344 |
| SF3B6 | | 0.132171 | 0.180141 | 0.164371 | 0.24922 | 0.18988 | 0.000347 |
| CCNYL1 | | 0.185482 | 0.281607 | 0.247633 | 0.311346 | 0.246464 | 0.000349 |
| TTC39C | | 0.215243 | 0.30909 | 0.26625 | 0.302231 | 0.240479 | 0.000351 |
| TAF11 | | 0.151272 | 0.363689 | 0.327486 | 0.405853 | 0.328074 | 0.000352 |
| GPX4 | | 0.149144 | 0.561319 | 0.506189 | 0.556728 | 0.460283 | 0.000353 |
| UBAP1 | | 0.139044 | 0.248554 | 0.225717 | 0.308467 | 0.243199 | 0.000355 |
| SLC25A1 | | 0.134438 | 0.349825 | 0.318699 | 0.389782 | 0.313928 | 0.000357 |
| TOX4 | | 0.133259 | 0.304946 | 0.27804 | 0.365076 | 0.287269 | 0.00036 |
| HERC4 | | 0.127463 | 0.463859 | 0.424635 | 0.473495 | 0.386834 | 0.000362 |
| ECHS1 | | 0.152545 | 0.378076 | 0.340141 | 0.416887 | 0.331882 | 0.000362 |
| LTBP3 | | 0.112928 | 0.195202 | 0.180505 | 0.23243 | 0.176279 | 0.000363 |
| PIAS2 | | 0.151274 | 0.210286 | 0.189353 | 0.252579 | 0.194233 | 0.000364 |
| EEF1A2 | | 1.568699 | 0.011768 | 0.003967 | 0.015112 | 0.003264 | 0.000366 |
| CEBPZ | | 0.132487 | 0.436605 | 0.398296 | 0.46366 | 0.377584 | 0.000366 |
| RIPK2 | | 0.20074 | 0.167977 | 0.146157 | 0.216839 | 0.165397 | 0.000366 |
| C1orf52 | | 0.110112 | 0.301474 | 0.279321 | 0.358599 | 0.281284 | 0.000366 |
| ANKZF1 | | 0.156056 | 0.305229 | 0.273935 | 0.336052 | 0.26877 | 0.000366 |
| FAM117A | | 0.13317 | 0.309559 | 0.282264 | 0.353322 | 0.279652 | 0.000368 |
| IL18R1 | | 4.172576 | 0.008519 | 0.000472 | 0.011993 | 0.001632 | 0.000368 |
| HIPK1 | | 0.137058 | 0.428851 | 0.389985 | 0.455985 | 0.373232 | 0.00037 |
| BAP1 | | 0.134556 | 0.26045 | 0.237257 | 0.32142 | 0.252992 | 0.000373 |
| GPR174 | | 0.238789 | 0.222627 | 0.188667 | 0.237707 | 0.184984 | 0.000375 |
| MAGEF1 | | 0.19125 | 0.211455 | 0.185202 | 0.258335 | 0.20185 | 0.000378 |
| MED10 | | 0.121381 | 0.46609 | 0.42848 | 0.498441 | 0.4037 | 0.000379 |
| DNAJC7 | | 0.163727 | 0.479979 | 0.428485 | 0.493404 | 0.408052 | 0.000379 |
| STUB1 | | 0.13104 | 0.407986 | 0.372562 | 0.449029 | 0.358542 | 0.000381 |
| COPA | | 0.177917 | 0.47642 | 0.421145 | 0.473735 | 0.397171 | 0.000381 |
| TPD52L2 | | 0.119449 | 0.283083 | 0.260589 | 0.346606 | 0.273123 | 0.000382 |
| DPH7 | | 0.182158 | 0.190189 | 0.167629 | 0.242504 | 0.186072 | 0.000383 |
| EIF3A | | 0.156944 | 0.660902 | 0.592779 | 0.591988 | 0.516866 | 0.000383 |
| NUP210 | | 0.151881 | 0.712169 | 0.641006 | 0.637563 | 0.539173 | 0.000383 |
| KIDINS220 | | 0.135296 | 0.25532 | 0.232465 | 0.308947 | 0.241023 | 0.000394 |
| AURKAIP1 | | 0.148711 | 0.310384 | 0.279983 | 0.348285 | 0.274211 | 0.000395 |
| CENPT | | 0.133136 | 0.204907 | 0.186844 | 0.257136 | 0.198585 | 0.000396 |
| REPIN1 | | 0.13653 | 0.305033 | 0.27749 | 0.359319 | 0.286181 | 0.000396 |
| IDH3A | | 0.125214 | 0.218435 | 0.200276 | 0.282562 | 0.217628 | 0.000396 |
| FBXW2 | | 0.127681 | 0.392569 | 0.359319 | 0.441593 | 0.355277 | 0.000403 |
| TRMT1L | | 0.165197 | 0.244227 | 0.217803 | 0.28544 | 0.226333 | 0.000405 |
| SHLD2 | | 0.130316 | 0.230022 | 0.210155 | 0.287839 | 0.224157 | 0.000406 |
| MAP3K3 | | 0.145435 | 0.326057 | 0.294791 | 0.362677 | 0.291077 | 0.000407 |
| PSMD9 | | 0.139671 | 0.345516 | 0.313634 | 0.389542 | 0.31012 | 0.000409 |
| U2AF1L4 | | 0.153715 | 0.280471 | 0.252124 | 0.32166 | 0.255169 | 0.00041 |
| MTF1 | | 0.155721 | 0.156288 | 0.140297 | 0.204366 | 0.153972 | 0.000416 |
| BAG5 | | 0.117024 | 0.280999 | 0.259106 | 0.33917 | 0.26605 | 0.000416 |
| PEX19 | | 0.125009 | 0.187721 | 0.17214 | 0.240585 | 0.183896 | 0.000419 |
| NUP42 | | 0.125108 | 0.454523 | 0.416768 | 0.478532 | 0.389554 | 0.00042 |
| ZNF580 | | 0.158056 | 0.383552 | 0.343752 | 0.407052 | 0.331338 | 0.000422 |
| CHCHD10 | | 0.156971 | 0.278812 | 0.250068 | 0.341089 | 0.270947 | 0.000423 |
| ZCRB1 | | 0.17151 | 0.265616 | 0.235844 | 0.327657 | 0.259521 | 0.000425 |
| ZFP36 | | 0.108738 | 1.62211 | 1.504343 | 0.894939 | 0.835147 | 0.000428 |
| GPS1 | | 0.15165 | 0.281655 | 0.253551 | 0.33941 | 0.269859 | 0.00043 |
| WDR33 | | 0.13418 | 0.609551 | 0.555415 | 0.598705 | 0.49728 | 0.00043 |
| TMEM109 | | 0.149806 | 0.480356 | 0.432979 | 0.487647 | 0.404244 | 0.00043 |
| RNASEH1 | | 0.145891 | 0.311971 | 0.281966 | 0.350684 | 0.27802 | 0.00043 |
| COX17 | | 0.133568 | 0.222119 | 0.202478 | 0.283761 | 0.219804 | 0.000431 |
| UPF3A | | 0.139006 | 0.251253 | 0.228174 | 0.312545 | 0.247008 | 0.000435 |
| IFRD2 | | 0.157997 | 0.229151 | 0.205381 | 0.271288 | 0.211099 | 0.000435 |
| TBC1D13 | | 0.17457 | 0.187222 | 0.165885 | 0.230511 | 0.176279 | 0.000437 |
| FAM136A | | 0.147125 | 0.227264 | 0.20523 | 0.281362 | 0.218716 | 0.00044 |
| COPS7A | | 0.143473 | 0.224872 | 0.203585 | 0.270329 | 0.211099 | 0.000441 |
| SDF4 | | 0.166296 | 0.520524 | 0.463853 | 0.487407 | 0.408596 | 0.000442 |
| SLC46A1 | | 1.105586 | 0.007088 | 0.003294 | 0.019429 | 0.005985 | 0.000445 |
| VAPA | | 0.153979 | 0.34995 | 0.314524 | 0.41113 | 0.33025 | 0.000446 |
| ELAC2 | | 0.152032 | 0.34001 | 0.306002 | 0.378988 | 0.308487 | 0.000446 |
| ATG13 | | 0.153489 | 0.282937 | 0.254381 | 0.345646 | 0.273123 | 0.000447 |
| WASHC5 | | 0.102679 | 0.246342 | 0.229418 | 0.30343 | 0.235038 | 0.000451 |
| LAMTOR5 | | 0.12513 | 0.207527 | 0.190286 | 0.272008 | 0.210555 | 0.000451 |
| GTPBP1 | | 0.153064 | 0.546334 | 0.491339 | 0.555529 | 0.457563 | 0.000454 |
| FOXP4 | | 0.177253 | 0.205675 | 0.181896 | 0.237947 | 0.183896 | 0.000456 |
| GAR1 | | 0.116614 | 0.235509 | 0.217222 | 0.284481 | 0.220892 | 0.000456 |
| PPP1CA | | 0.114037 | 1.066055 | 0.985034 | 0.779563 | 0.713819 | 0.000456 |
| KCTD9 | | 0.161362 | 0.175783 | 0.157182 | 0.217798 | 0.164853 | 0.000457 |
| NUDCD2 | | 0.136742 | 0.306325 | 0.278625 | 0.350444 | 0.27802 | 0.000458 |
| SDF2 | | 0.111026 | 0.14822 | 0.137242 | 0.208443 | 0.155604 | 0.000458 |
| SNAPIN | | 0.152997 | 0.255851 | 0.230107 | 0.307508 | 0.243743 | 0.00046 |
| FBXW7 | | 0.140179 | 0.696343 | 0.631867 | 0.608779 | 0.523395 | 0.000462 |
| ZMYM5 | | 0.144769 | 0.171258 | 0.154907 | 0.217798 | 0.164309 | 0.000463 |
| RELCH | | 0.142887 | 0.461692 | 0.418157 | 0.478292 | 0.396083 | 0.000465 |
| PRORP | | 0.106056 | 0.1578 | 0.146616 | 0.216599 | 0.163765 | 0.000465 |
| CZIB | | 0.133118 | 0.184967 | 0.168663 | 0.237947 | 0.182263 | 0.000472 |
| SH3BP1 | | 0.136324 | 0.339291 | 0.308699 | 0.378508 | 0.301415 | 0.000475 |
| LAMTOR4 | | 0.136145 | 0.843176 | 0.767246 | 0.710242 | 0.614799 | 0.000475 |
| IL27RA | | 0.150843 | 0.32747 | 0.29496 | 0.355481 | 0.287269 | 0.000478 |
| PURA | | 0.124486 | 0.176672 | 0.162067 | 0.223075 | 0.168662 | 0.000478 |
| SNF8 | | 0.14747 | 0.240669 | 0.217283 | 0.301271 | 0.236126 | 0.000483 |
| CDK17 | | 0.112354 | 0.191677 | 0.177316 | 0.257616 | 0.198041 | 0.000484 |
| FNBP1 | | 0.116549 | 0.91405 | 0.843112 | 0.743104 | 0.65778 | 0.000485 |
| MZT1 | | 0.144846 | 0.207334 | 0.187528 | 0.257136 | 0.199129 | 0.000486 |
| FAM168A | | 0.169803 | 0.172116 | 0.153005 | 0.210602 | 0.158868 | 0.000486 |
| ZBTB7A | | 0.12179 | 0.614177 | 0.564458 | 0.591269 | 0.492927 | 0.000487 |
| YWHAE | | 0.172638 | 0.36112 | 0.320393 | 0.404653 | 0.322089 | 0.000488 |
| C12orf4 | | 0.116648 | 0.146753 | 0.135355 | 0.201487 | 0.150707 | 0.000488 |
| NAA30 | | 0.113051 | 0.235675 | 0.217912 | 0.295275 | 0.229597 | 0.000489 |
| COMMD7 | | 0.141856 | 0.211915 | 0.192069 | 0.268889 | 0.208923 | 0.000489 |
| DHX16 | | 0.111403 | 0.231276 | 0.214089 | 0.281362 | 0.21926 | 0.000489 |
| IPO9 | | 0.145629 | 0.256167 | 0.231571 | 0.306308 | 0.242655 | 0.000493 |
| MYO18A | | 0.142035 | 0.352809 | 0.31973 | 0.408012 | 0.33025 | 0.000493 |
| RAB4A | | 0.134013 | 0.310184 | 0.282668 | 0.348525 | 0.279652 | 0.000496 |
| CKAP5 | | 0.144515 | 0.162849 | 0.147327 | 0.210842 | 0.158324 | 0.000496 |
| STRN4 | | 0.120668 | 0.396691 | 0.364861 | 0.442552 | 0.355822 | 0.000498 |
| TOB2 | | 0.177359 | 0.296587 | 0.262278 | 0.331254 | 0.26605 | 0.000499 |
| PSMB5 | | 0.152131 | 0.148677 | 0.133797 | 0.207004 | 0.156148 | 0.000501 |
| UBE2J2 | | 0.128809 | 0.31246 | 0.285772 | 0.367954 | 0.291621 | 0.000505 |
| EIF3J | | 0.151131 | 0.369728 | 0.332957 | 0.403694 | 0.323177 | 0.000505 |
| SEH1L | | 0.141137 | 0.312403 | 0.283289 | 0.359559 | 0.285092 | 0.000507 |
| RPA1 | | 0.152781 | 0.343334 | 0.308834 | 0.387143 | 0.311752 | 0.000508 |
| FMNL1 | | 0.105579 | 1.231537 | 1.14463 | 0.842408 | 0.776387 | 0.000511 |
| EDRF1 | | 0.127074 | 0.229369 | 0.21003 | 0.291917 | 0.229597 | 0.000511 |
| AGAP1 | | 2.528652 | 0.006244 | 0.001082 | 0.014632 | 0.003264 | 0.000512 |
| RNF187 | | 0.132644 | 0.38409 | 0.350351 | 0.431758 | 0.348749 | 0.000513 |
| PPP4R2 | | 0.124578 | 0.22627 | 0.207551 | 0.2852 | 0.22198 | 0.000514 |
| SLC1A5 | | 0.196792 | 0.174437 | 0.152194 | 0.216359 | 0.165397 | 0.000517 |
| NDUFS7 | | 0.153764 | 0.432711 | 0.388965 | 0.44615 | 0.366703 | 0.000518 |
| TRAPPC5 | | 0.144731 | 0.521422 | 0.471651 | 0.511873 | 0.426007 | 0.000518 |
| VAMP8 | | 0.139702 | 0.642603 | 0.583295 | 0.597026 | 0.503808 | 0.000519 |
| ATP6V0A2 | | 0.136947 | 0.319024 | 0.290134 | 0.359079 | 0.286725 | 0.000519 |
| AP5M1 | | 0.161809 | 0.212055 | 0.189557 | 0.271048 | 0.212187 | 0.000529 |
| PTPN23 | | 0.160631 | 0.260808 | 0.233327 | 0.317102 | 0.252448 | 0.000529 |
| RAB1A | | 0.152259 | 0.265302 | 0.238729 | 0.337731 | 0.267138 | 0.00053 |
| SSR3 | | 0.239298 | 0.333695 | 0.282692 | 0.300072 | 0.245375 | 0.00053 |
| MANEAL | | 1.761773 | 0.016371 | 0.004828 | 0.020868 | 0.007073 | 0.000531 |
| OXSR1 | | 0.147781 | 0.298521 | 0.269456 | 0.349005 | 0.279108 | 0.000536 |
| NOC4L | | 0.181113 | 0.163086 | 0.143846 | 0.21492 | 0.164853 | 0.000538 |
| YY1 | | 0.130046 | 0.427533 | 0.39068 | 0.471096 | 0.382481 | 0.000538 |
| ITFG1 | | 0.127104 | 0.370702 | 0.33944 | 0.413768 | 0.331882 | 0.00054 |
| LYG1 | | 1.36726 | 0.015951 | 0.006183 | 0.024226 | 0.009249 | 0.000542 |
| GART | | 0.116763 | 0.198247 | 0.182834 | 0.255457 | 0.196953 | 0.000543 |
| TADA2B | | 0.10133 | 0.315067 | 0.293697 | 0.366275 | 0.288901 | 0.000544 |
| ZSWIM8 | | 0.130706 | 0.308629 | 0.281897 | 0.350204 | 0.277476 | 0.000547 |
| HSH2D | | 0.137304 | 0.698111 | 0.634734 | 0.623651 | 0.537541 | 0.000548 |
| TRMT112 | | 0.162054 | 0.41015 | 0.366572 | 0.458383 | 0.374864 | 0.000549 |
| POP5 | | 0.111183 | 0.229806 | 0.212761 | 0.281842 | 0.218172 | 0.000553 |
| SLC35F5 | | 0.109829 | 0.380585 | 0.352688 | 0.422403 | 0.341676 | 0.000554 |
| C5orf24 | | 0.144067 | 0.197426 | 0.178663 | 0.244663 | 0.189336 | 0.000554 |
| ATP5MC3 | | 0.174169 | 0.399032 | 0.353653 | 0.421924 | 0.343852 | 0.000556 |
| PXK | | 0.126278 | 0.807785 | 0.740087 | 0.666347 | 0.59086 | 0.000556 |
| TRAPPC6B | | 0.091436 | 0.192825 | 0.180983 | 0.247062 | 0.188792 | 0.000556 |
| EIF5B | | 0.161609 | 0.320745 | 0.286755 | 0.37443 | 0.300326 | 0.000558 |
| DNPEP | | 0.116895 | 0.242071 | 0.22323 | 0.299352 | 0.234494 | 0.000558 |
| POLG | | 0.148163 | 0.26094 | 0.235472 | 0.309187 | 0.243743 | 0.000562 |
| ANKRD11 | | 0.140578 | 0.584306 | 0.530057 | 0.573999 | 0.478237 | 0.000564 |
| MAP3K11 | | 0.112725 | 0.264121 | 0.24427 | 0.323099 | 0.252992 | 0.00057 |
| STXBP2 | | 0.139998 | 0.651745 | 0.591471 | 0.608299 | 0.519042 | 0.000578 |
| MPHOSPH8 | | 0.137949 | 0.406279 | 0.36923 | 0.445191 | 0.365615 | 0.000587 |
| TMEM50B | | 0.130824 | 0.231009 | 0.210983 | 0.284721 | 0.223069 | 0.000587 |
| AP3B1 | | 0.127758 | 0.39633 | 0.362743 | 0.443032 | 0.356366 | 0.000594 |
| C1orf43 | | 0.125471 | 0.262113 | 0.24028 | 0.332934 | 0.260609 | 0.000594 |
| NHERF1 | | 0.140287 | 0.439886 | 0.399125 | 0.416407 | 0.337323 | 0.0006 |
| USP14 | | 0.157696 | 0.30843 | 0.276493 | 0.368913 | 0.29543 | 0.000608 |
| NRIP1 | | 0.126725 | 0.266719 | 0.24429 | 0.295515 | 0.229053 | 0.000611 |
| CCDC43 | | 0.084921 | 0.151144 | 0.142504 | 0.211562 | 0.159956 | 0.000612 |
| MED13 | | 0.138607 | 0.271958 | 0.247046 | 0.329575 | 0.262786 | 0.000613 |
| GIPR | | 1.149412 | 0.007657 | 0.003452 | 0.019909 | 0.006529 | 0.000614 |
| DYNC1LI1 | | 0.126133 | 0.252739 | 0.231581 | 0.311106 | 0.245375 | 0.000615 |
| PYM1 | | 0.130268 | 0.288936 | 0.26399 | 0.324298 | 0.257889 | 0.000616 |
| PRPF38A | | 0.12041 | 0.297412 | 0.273597 | 0.347325 | 0.273667 | 0.000616 |
| PHF20 | | 0.153477 | 0.197441 | 0.177516 | 0.238906 | 0.184984 | 0.000617 |
| NRBF2 | | 0.127693 | 0.2482 | 0.227176 | 0.306788 | 0.242111 | 0.000617 |
| ATP13A3 | | 0.121046 | 0.147473 | 0.135605 | 0.210602 | 0.158868 | 0.000618 |
| ITPA | | 0.127168 | 0.349521 | 0.320031 | 0.399376 | 0.319913 | 0.000619 |
| UBXN4 | | 0.159963 | 0.577859 | 0.517211 | 0.549292 | 0.461371 | 0.000621 |
| SLC35A4 | | 0.155818 | 0.353343 | 0.317169 | 0.39362 | 0.318825 | 0.000622 |
| FBXO25 | | 0.135122 | 0.197752 | 0.180072 | 0.25042 | 0.194233 | 0.000627 |
| TMEM163 | | 1.053371 | 0.023447 | 0.011298 | 0.031902 | 0.01469 | 0.000634 |
| RAPGEF1 | | 0.128126 | 0.624129 | 0.57109 | 0.603022 | 0.507617 | 0.000636 |
| DCUN1D1 | | 0.097339 | 0.229958 | 0.214955 | 0.293116 | 0.230141 | 0.000637 |
| NAA20 | | 0.141865 | 0.26169 | 0.237181 | 0.323819 | 0.254625 | 0.000637 |
| CRIPT | | 0.126811 | 0.281191 | 0.25753 | 0.341569 | 0.270947 | 0.000637 |
| MED8 | | 0.134108 | 0.168812 | 0.153827 | 0.23219 | 0.178455 | 0.000638 |
| REEP5 | | 0.147567 | 0.835417 | 0.754191 | 0.680739 | 0.595756 | 0.000649 |
| COPZ1 | | 0.155385 | 0.19122 | 0.171695 | 0.260974 | 0.203482 | 0.00065 |
| RNF5 | | 0.126389 | 0.356144 | 0.326271 | 0.3929 | 0.315016 | 0.000652 |
| PET100 | | 0.135596 | 0.326835 | 0.297516 | 0.394099 | 0.314472 | 0.000655 |
| TIMM10B | | 0.120501 | 0.256886 | 0.236301 | 0.300792 | 0.235582 | 0.000657 |
| COPS6 | | 0.151379 | 0.308783 | 0.278025 | 0.361238 | 0.290533 | 0.000664 |
| RER1 | | 0.152786 | 0.551378 | 0.49597 | 0.528424 | 0.437976 | 0.000665 |
| DHRSX | | 0.173885 | 0.219336 | 0.194431 | 0.25018 | 0.196953 | 0.000666 |
| CRTAP | | 0.129796 | 0.193233 | 0.176607 | 0.238906 | 0.185528 | 0.000672 |
| DBI | | 0.131997 | 0.295002 | 0.269209 | 0.358359 | 0.284548 | 0.000673 |
| RASAL3 | | 0.1164 | 0.36118 | 0.333184 | 0.398177 | 0.322089 | 0.000673 |
| ARRDC3 | | 0.126816 | 0.675366 | 0.618534 | 0.599664 | 0.502176 | 0.000674 |
| NOB1 | | 0.13269 | 0.266513 | 0.243094 | 0.319021 | 0.249728 | 0.000677 |
| MUS81 | | 0.097583 | 0.259074 | 0.24213 | 0.314464 | 0.248096 | 0.00068 |
| GFM1 | | 0.116846 | 0.157134 | 0.144909 | 0.218997 | 0.167029 | 0.00068 |
| THOC5 | | 0.098925 | 0.185664 | 0.17336 | 0.244903 | 0.187704 | 0.00068 |
| IRF3 | | 0.133594 | 0.743914 | 0.678121 | 0.66155 | 0.562024 | 0.000682 |
| HCK | | 0.188193 | 0.522433 | 0.458542 | 0.422164 | 0.362894 | 0.000686 |
| SRP54 | | 0.1776 | 0.379465 | 0.335512 | 0.3929 | 0.322633 | 0.000688 |
| SAP130 | | 0.158467 | 0.169008 | 0.151428 | 0.220916 | 0.16975 | 0.000689 |
| CCDC28A | | 0.127168 | 0.210389 | 0.192638 | 0.266011 | 0.208923 | 0.000696 |
| ADRB2 | | 0.189251 | 0.251251 | 0.220362 | 0.255937 | 0.204026 | 0.000697 |
| NDUFAB1 | | 0.152741 | 0.411854 | 0.370479 | 0.421204 | 0.347116 | 0.000704 |
| SCNM1 | | 0.118754 | 0.22282 | 0.205213 | 0.282802 | 0.221436 | 0.000704 |
| EHMT1 | | 0.124781 | 0.704945 | 0.646536 | 0.634205 | 0.538629 | 0.000707 |
| MON2 | | 0.128759 | 0.308665 | 0.282311 | 0.371072 | 0.297062 | 0.000707 |
| PEPD | | 0.072239 | 0.194099 | 0.18462 | 0.253058 | 0.193145 | 0.000708 |
| HCFC1 | | 0.148473 | 0.413516 | 0.373076 | 0.448549 | 0.366703 | 0.000712 |
| SIGLEC10 | | 0.191723 | 0.478068 | 0.418577 | 0.409451 | 0.347116 | 0.000714 |
| NFX1 | | 0.127007 | 0.553686 | 0.507027 | 0.554809 | 0.467356 | 0.000715 |
| MTDH | | 0.148595 | 0.742215 | 0.669573 | 0.616695 | 0.542982 | 0.000715 |
| HDAC6 | | 0.138225 | 0.166717 | 0.151485 | 0.218278 | 0.167029 | 0.000719 |
| ECD | | 0.157756 | 0.191075 | 0.171283 | 0.244903 | 0.188248 | 0.000721 |
| INO80D | | 0.128706 | 0.283112 | 0.258949 | 0.334852 | 0.26605 | 0.000722 |
| ATG12 | | 0.147729 | 0.231474 | 0.208945 | 0.282322 | 0.223613 | 0.000722 |
| LSM6 | | 0.121836 | 0.418669 | 0.384764 | 0.447589 | 0.365615 | 0.000722 |
| THOP1 | | 0.137949 | 0.186539 | 0.169529 | 0.228832 | 0.175734 | 0.000727 |
| SIN3B | | 0.142159 | 0.392503 | 0.355672 | 0.419525 | 0.346028 | 0.000728 |
| SDSL | | 65535 | 0.002052 | 0 | 0.007676 | 0 | 0.000728 |
| RPAIN | | 0.116521 | 0.590872 | 0.545026 | 0.562725 | 0.470076 | 0.000729 |
| YBX1 | | 0.145924 | 0.642725 | 0.580895 | 0.591509 | 0.512514 | 0.00073 |
| TMEM9B | | 0.123918 | 0.432899 | 0.397268 | 0.467258 | 0.378128 | 0.000732 |
| TEX14 | | 2.557669 | 0.004617 | 0.000784 | 0.013193 | 0.00272 | 0.000733 |
| MAP4 | | 0.122923 | 0.600468 | 0.551425 | 0.569441 | 0.47062 | 0.000736 |
| CAST | | 0.127856 | 0.544458 | 0.498283 | 0.524586 | 0.440152 | 0.000737 |
| GMPPB | | 0.231409 | 0.351528 | 0.299433 | 0.316143 | 0.261697 | 0.000737 |
| ISCU | | 0.146971 | 0.645216 | 0.582723 | 0.581195 | 0.500544 | 0.000738 |
| UBXN7 | | 0.130238 | 0.311274 | 0.284405 | 0.3574 | 0.288901 | 0.00074 |
| COX14 | | 0.122435 | 0.366044 | 0.336261 | 0.409451 | 0.332427 | 0.000741 |
| NPAT | | 0.156683 | 0.207402 | 0.186057 | 0.248981 | 0.193689 | 0.000741 |
| ZNF493 | | 0.158878 | 0.247375 | 0.221579 | 0.271048 | 0.213275 | 0.000742 |
| CIZ1 | | 0.054099 | 0.224997 | 0.216716 | 0.295754 | 0.226877 | 0.000742 |
| UBL5 | | 0.125433 | 0.321992 | 0.29518 | 0.380907 | 0.306311 | 0.000743 |
| DARS1 | | 0.134852 | 0.453918 | 0.413412 | 0.474214 | 0.391186 | 0.000748 |
| CCM2 | | 0.136918 | 0.605774 | 0.550927 | 0.595586 | 0.494559 | 0.00075 |
| EIF3I | | 0.091655 | 0.158432 | 0.14868 | 0.228352 | 0.174102 | 0.00075 |
| ZNF687 | | 0.150055 | 0.257675 | 0.232221 | 0.309667 | 0.247008 | 0.000752 |
| NUDT5 | | 0.140085 | 0.27549 | 0.249998 | 0.325018 | 0.256801 | 0.000756 |
| NOP14 | | 0.117917 | 0.318518 | 0.29352 | 0.364596 | 0.288901 | 0.000756 |
| UNC45A | | 0.141337 | 0.178098 | 0.161478 | 0.239866 | 0.184984 | 0.000757 |
| SMPD4 | | 0.154517 | 0.309384 | 0.277961 | 0.364116 | 0.29543 | 0.000757 |
| KRIT1 | | 0.138657 | 0.218635 | 0.1986 | 0.265052 | 0.207835 | 0.000764 |
| SEC23B | | 0.17232 | 0.330365 | 0.293171 | 0.351403 | 0.286181 | 0.000774 |
| VPS26C | | 0.111689 | 0.261779 | 0.242278 | 0.314224 | 0.249184 | 0.000774 |
| PI4K2A | | 0.156154 | 0.201947 | 0.18123 | 0.251379 | 0.197497 | 0.000775 |
| PCGF3 | | 0.118909 | 0.233345 | 0.214883 | 0.279444 | 0.21926 | 0.00078 |
| DNAJB2 | | 0.115461 | 0.209962 | 0.193814 | 0.265771 | 0.206746 | 0.000781 |
| NOSIP | | 0.109308 | 0.263478 | 0.244253 | 0.334613 | 0.263874 | 0.000787 |
| ZNF30 | | 1.017341 | 0.023408 | 0.011564 | 0.029264 | 0.013058 | 0.000792 |
| SP140 | | 0.111986 | 1.060167 | 0.980986 | 0.773807 | 0.712731 | 0.000798 |
| MAPK3 | | 0.102621 | 0.221897 | 0.206661 | 0.26865 | 0.210555 | 0.000811 |
| TMED2 | | 0.18656 | 0.2706 | 0.237776 | 0.296474 | 0.23667 | 0.000812 |
| CYRIB | | 0.122521 | 0.556858 | 0.51152 | 0.559607 | 0.459739 | 0.000812 |
| SOS2 | | 0.14967 | 0.316623 | 0.285422 | 0.346606 | 0.28074 | 0.000813 |
| ST3GAL2 | | 0.113566 | 0.351422 | 0.324819 | 0.394819 | 0.318281 | 0.000814 |
| GUSB | | 0.117438 | 0.294869 | 0.271817 | 0.344447 | 0.273123 | 0.000816 |
| HIPK2 | | 0.16727 | 0.287135 | 0.255702 | 0.310626 | 0.248096 | 0.000818 |
| ECHDC1 | | 0.117391 | 0.400693 | 0.36938 | 0.435116 | 0.355277 | 0.000822 |
| PEX26 | | 0.06254 | 0.163364 | 0.156434 | 0.219237 | 0.164853 | 0.000825 |
| TOMM6 | | 0.111358 | 0.243641 | 0.225543 | 0.318062 | 0.250816 | 0.000828 |
| NDUFAF8 | | 0.129191 | 0.451814 | 0.413113 | 0.459103 | 0.375952 | 0.000833 |
| BAZ1A | | 0.115406 | 0.321922 | 0.297174 | 0.367714 | 0.294886 | 0.000837 |
| MED24 | | 0.105994 | 0.37346 | 0.347005 | 0.423363 | 0.337323 | 0.000841 |
| RECQL | | 0.13218 | 0.353576 | 0.322621 | 0.397697 | 0.320457 | 0.000844 |
| RGS13 | | 1.115413 | 0.021709 | 0.01002 | 0.025186 | 0.010337 | 0.000845 |
| PSMG2 | | 0.119516 | 0.396199 | 0.3647 | 0.444951 | 0.358542 | 0.000846 |
| SNRPN | | 0.103456 | 0.289223 | 0.269209 | 0.345167 | 0.272035 | 0.000846 |
| PRPS2 | | 0.158675 | 0.205616 | 0.1842 | 0.24922 | 0.194233 | 0.000849 |
| CPNE3 | | 0.136057 | 0.282142 | 0.25675 | 0.331494 | 0.266594 | 0.000853 |
| STAT2 | | 0.120645 | 0.47844 | 0.440058 | 0.498681 | 0.405876 | 0.000854 |
| AAAS | | 0.113008 | 0.224251 | 0.207356 | 0.276565 | 0.217084 | 0.000854 |
| ALOX5AP | | 0.162731 | 0.55581 | 0.496523 | 0.48285 | 0.412405 | 0.000857 |
| SLC6A9 | | 1.684836 | 0.008757 | 0.002724 | 0.021828 | 0.008161 | 0.00086 |
| HYCC2 | | 0.084282 | 0.210175 | 0.198248 | 0.264572 | 0.206202 | 0.000861 |
| NMRAL1 | | 0.128209 | 0.187545 | 0.171597 | 0.243943 | 0.189336 | 0.000862 |
| DDX43 | | 2.275835 | 0.007708 | 0.001592 | 0.011993 | 0.002176 | 0.000871 |
| CHORDC1 | | 0.070541 | 0.195132 | 0.185821 | 0.258095 | 0.198041 | 0.00088 |
| GRWD1 | | 0.115416 | 0.252571 | 0.233153 | 0.309906 | 0.245375 | 0.000883 |
| SLC25A36 | | 0.112768 | 0.411801 | 0.380838 | 0.443991 | 0.362894 | 0.000889 |
| MAGED1 | | 0.142607 | 0.501668 | 0.454451 | 0.478532 | 0.398803 | 0.000899 |
| PRELID3B | | 0.148342 | 0.157474 | 0.142086 | 0.221156 | 0.170294 | 0.000901 |
| KCTD13 | | 0.176362 | 0.253176 | 0.224044 | 0.277045 | 0.223069 | 0.000902 |
| CBLB | | 0.165251 | 0.560767 | 0.500078 | 0.48189 | 0.423286 | 0.000907 |
| STX6 | | 0.147713 | 0.2247 | 0.202832 | 0.266011 | 0.210011 | 0.000911 |
| NCLN | | 0.159219 | 0.389066 | 0.348412 | 0.404653 | 0.334603 | 0.000913 |
| NACC1 | | 0.113931 | 0.35987 | 0.332544 | 0.396978 | 0.319913 | 0.000914 |
| POLR2I | | 0.105695 | 0.258536 | 0.240272 | 0.312065 | 0.244287 | 0.000921 |
| RFXANK | | 0.127156 | 0.409836 | 0.37526 | 0.435356 | 0.356366 | 0.000923 |
| TRABD | | 0.141621 | 0.537471 | 0.487218 | 0.505877 | 0.422198 | 0.000923 |
| SLC35F6 | | 0.090744 | 0.14977 | 0.14064 | 0.211801 | 0.161045 | 0.000929 |
| PFKFB3 | | 0.181633 | 0.247945 | 0.218614 | 0.258335 | 0.205658 | 0.000929 |
| HDAC10 | | 0.132326 | 0.181641 | 0.165722 | 0.230991 | 0.178999 | 0.000931 |
| USP15 | | 0.121575 | 0.575034 | 0.528562 | 0.581434 | 0.477693 | 0.000934 |
| UQCC6 | | 1.06056 | 0.013123 | 0.006292 | 0.020149 | 0.007073 | 0.000935 |
| DDX56 | | 0.087367 | 0.18891 | 0.177809 | 0.24922 | 0.193689 | 0.000935 |
| MDN1 | | 0.1435 | 0.214698 | 0.19437 | 0.26745 | 0.212187 | 0.000936 |
| TMX2 | | 0.118696 | 0.363157 | 0.334475 | 0.41065 | 0.332427 | 0.000936 |
| SAMD1 | | 0.142004 | 0.208174 | 0.18866 | 0.261454 | 0.20457 | 0.000938 |
| LRRC8A | | 0.129477 | 0.253736 | 0.231956 | 0.297673 | 0.23667 | 0.000939 |
| HPS1 | | 0.109567 | 0.414097 | 0.383813 | 0.450228 | 0.365615 | 0.000952 |
| TXNL1 | | 0.128526 | 0.364917 | 0.333814 | 0.413049 | 0.33025 | 0.000952 |
| PPP2R5D | | 0.169302 | 0.178446 | 0.158687 | 0.233629 | 0.182807 | 0.000953 |
| XPC | | 0.116514 | 0.252732 | 0.233123 | 0.299112 | 0.234494 | 0.000953 |
| CUL4A | | 0.11727 | 0.288915 | 0.26636 | 0.341329 | 0.273667 | 0.000961 |
| DNAJB5 | | 1.005995 | 0.011037 | 0.005496 | 0.022547 | 0.008705 | 0.00097 |
| IFI27 | | 1.585487 | 0.007272 | 0.002423 | 0.019189 | 0.006529 | 0.000971 |
| ADHFE1 | | 1.053351 | 0.01703 | 0.008206 | 0.024226 | 0.009793 | 0.000971 |
| HOOK3 | | 0.123276 | 0.322547 | 0.29613 | 0.369153 | 0.297606 | 0.000972 |
| SHFL | | 0.111593 | 0.270007 | 0.249909 | 0.307748 | 0.245919 | 0.000975 |
| HSPA4 | | 0.133726 | 0.2141 | 0.195146 | 0.276805 | 0.218716 | 0.000979 |
| PNPLA7 | | 0.186386 | 0.321876 | 0.282866 | 0.309187 | 0.249184 | 0.00099 |
| ZNF490 | | 0.132124 | 0.181361 | 0.165489 | 0.219957 | 0.169206 | 0.000992 |
| STAU1 | | 0.127317 | 0.470907 | 0.431131 | 0.5006 | 0.408052 | 0.000995 |
| CNOT6L | | 0.131687 | 0.676197 | 0.617208 | 0.635164 | 0.544614 | 0.001001 |
| BLNK | | 0.119687 | 0.75736 | 0.697065 | 0.671624 | 0.577258 | 0.001004 |
| GTF3C3 | | 0.092346 | 0.251382 | 0.235796 | 0.305109 | 0.241023 | 0.001004 |
| MOAP1 | | 0.122938 | 0.5184 | 0.476054 | 0.506356 | 0.422198 | 0.001005 |
| NOM1 | | 0.146977 | 0.171045 | 0.154478 | 0.216599 | 0.166485 | 0.001007 |
| GAPVD1 | | 0.087365 | 0.193225 | 0.181871 | 0.254977 | 0.196953 | 0.001007 |
| CERS2 | | 0.124642 | 0.411266 | 0.377226 | 0.454545 | 0.365615 | 0.001012 |
| SRF | | 0.11641 | 0.326547 | 0.301233 | 0.379228 | 0.304679 | 0.001013 |
| DNMT1 | | 0.111618 | 0.226644 | 0.20977 | 0.272248 | 0.211643 | 0.001014 |
| PRPF4 | | 0.140621 | 0.184054 | 0.166961 | 0.23291 | 0.181175 | 0.001016 |
| TPP1 | | 0.121583 | 0.776532 | 0.713772 | 0.661789 | 0.576714 | 0.001017 |
| TMEM259 | | 0.13097 | 0.552635 | 0.504676 | 0.558167 | 0.463003 | 0.001028 |
| SYNPO | | 0.201774 | 0.342192 | 0.29753 | 0.314704 | 0.258977 | 0.001032 |
| ARNT | | 0.125175 | 0.369988 | 0.339239 | 0.403694 | 0.33025 | 0.001033 |
| MTR | | 0.11416 | 0.257546 | 0.237952 | 0.30295 | 0.239391 | 0.001037 |
| SLC25A32 | | 0.101334 | 0.314284 | 0.292966 | 0.372751 | 0.297062 | 0.001043 |
| RWDD1 | | 0.120115 | 0.221271 | 0.203594 | 0.280163 | 0.220892 | 0.001044 |
| USP16 | | 0.129247 | 0.363642 | 0.332481 | 0.418086 | 0.339499 | 0.001054 |
| ZFP92 | | 1.367139 | 0.009201 | 0.003567 | 0.015591 | 0.004353 | 0.001068 |
| NMT1 | | 0.120652 | 0.515317 | 0.473974 | 0.528904 | 0.432535 | 0.001069 |
| ENO1 | | 0.149725 | 0.508227 | 0.458127 | 0.509715 | 0.425462 | 0.001071 |
| BECN1 | | 0.101958 | 0.265861 | 0.247721 | 0.330295 | 0.262242 | 0.001072 |
| SLC5A6 | | 0.131194 | 0.458626 | 0.41876 | 0.450947 | 0.373776 | 0.001073 |
| EWSR1 | | 0.126649 | 0.470735 | 0.431173 | 0.501079 | 0.409684 | 0.001074 |
| POMP | | 0.141441 | 0.214643 | 0.194598 | 0.284721 | 0.223613 | 0.001074 |
| ZNF263 | | 0.082043 | 0.188216 | 0.177811 | 0.228832 | 0.174646 | 0.001078 |
| RASGRP4 | | 2.019715 | 0.006863 | 0.001692 | 0.020628 | 0.007617 | 0.00108 |
| CEP104 | | 0.114624 | 0.210563 | 0.194481 | 0.26769 | 0.210011 | 0.001086 |
| CCT6A | | 0.119964 | 0.317476 | 0.292145 | 0.371552 | 0.29815 | 0.00109 |
| ANXA1 | | 2.915327 | 0.00373 | 0.000494 | 0.009595 | 0.001088 | 0.001091 |
| ZNF830 | | 0.111396 | 0.298573 | 0.276387 | 0.345886 | 0.276931 | 0.001093 |
| MARK3 | | 0.123284 | 0.473356 | 0.434586 | 0.508275 | 0.420566 | 0.001093 |
| EAF1 | | 0.142093 | 0.236631 | 0.214436 | 0.290717 | 0.232862 | 0.0011 |
| HEATR5B | | 0.125671 | 0.515908 | 0.47287 | 0.520269 | 0.430903 | 0.001107 |
| PLEKHA3 | | 0.130479 | 0.214972 | 0.196383 | 0.266251 | 0.210011 | 0.001112 |
| M6PR | | 0.123442 | 0.596549 | 0.547629 | 0.577836 | 0.486398 | 0.001113 |
| SEPTIN2 | | 0.084377 | 0.215217 | 0.202991 | 0.2864 | 0.223069 | 0.001117 |
| PSMC3 | | 0.139657 | 0.360792 | 0.327503 | 0.39362 | 0.319913 | 0.00113 |
| XPO7 | | 0.111459 | 0.166296 | 0.153932 | 0.228592 | 0.177367 | 0.001133 |
| LRRC36 | | 1.165031 | 0.014951 | 0.006667 | 0.019909 | 0.007073 | 0.001137 |
| PACC1 | | 0.086227 | 0.214572 | 0.202123 | 0.260974 | 0.203482 | 0.001137 |
| RETREG3 | | 0.131311 | 0.574639 | 0.524646 | 0.57064 | 0.478781 | 0.001137 |
| EXOSC1 | | 0.04983 | 0.184934 | 0.178656 | 0.248261 | 0.192057 | 0.001137 |
| DENND6A | | 0.13011 | 0.314612 | 0.287481 | 0.346606 | 0.280196 | 0.001146 |
| RCOR1 | | 0.12951 | 0.233792 | 0.213719 | 0.283281 | 0.224701 | 0.001152 |
| MBP | | 0.142398 | 0.302731 | 0.274278 | 0.327177 | 0.26605 | 0.001154 |
| NARF | | 0.127377 | 0.323399 | 0.29607 | 0.37587 | 0.304135 | 0.001155 |
| HPS4 | | 0.116887 | 0.212331 | 0.195807 | 0.259295 | 0.202938 | 0.001157 |
| OS9 | | 0.115044 | 0.944181 | 0.871814 | 0.736388 | 0.664853 | 0.001161 |
| DDRGK1 | | 0.102754 | 0.267891 | 0.249475 | 0.331734 | 0.263874 | 0.001162 |
| BABAM1 | | 0.14294 | 0.35472 | 0.32126 | 0.401055 | 0.325354 | 0.001171 |
| IL10RB | | 0.136863 | 0.477344 | 0.434142 | 0.487167 | 0.403156 | 0.001174 |
| GNPAT | | 0.098048 | 0.23031 | 0.215178 | 0.290717 | 0.229597 | 0.001174 |
| PPP2R5B | | 0.11302 | 0.208419 | 0.192714 | 0.246822 | 0.191513 | 0.001176 |
| SMNDC1 | | 0.107148 | 0.351103 | 0.325972 | 0.402255 | 0.324266 | 0.001177 |
| GSE1 | | 0.111117 | 0.444131 | 0.411208 | 0.465339 | 0.379217 | 0.001177 |
| TRPC4AP | | 0.128021 | 0.406139 | 0.371652 | 0.454545 | 0.3716 | 0.001182 |
| EBNA1BP2 | | 0.135231 | 0.25634 | 0.233404 | 0.296954 | 0.23667 | 0.001183 |
| NAP1L4 | | 0.139028 | 0.426656 | 0.387459 | 0.473495 | 0.385201 | 0.001186 |
| NAXE | | 0.085544 | 0.257457 | 0.242635 | 0.314944 | 0.248096 | 0.001189 |
| MYL12A | | 0.109196 | 0.319867 | 0.29655 | 0.39266 | 0.314472 | 0.001193 |
| SCPEP1 | | 0.136011 | 0.34836 | 0.317019 | 0.365315 | 0.29815 | 0.001201 |
| KIAA0930 | | 0.11882 | 0.392693 | 0.361647 | 0.426961 | 0.348749 | 0.001201 |
| POLG2 | | 0.133412 | 0.291243 | 0.265518 | 0.330295 | 0.266594 | 0.001209 |
| POLR1A | | 0.113871 | 0.20027 | 0.185071 | 0.253058 | 0.198585 | 0.001209 |
| LY75 | | 0.099787 | 0.387445 | 0.361552 | 0.427201 | 0.346028 | 0.001209 |
| CCNL1 | | 0.126668 | 0.734224 | 0.672508 | 0.662749 | 0.571273 | 0.001213 |
| SCAMP3 | | 0.121428 | 0.284758 | 0.261771 | 0.341569 | 0.274755 | 0.001215 |
| EMC8 | | 0.11496 | 0.283333 | 0.261632 | 0.334133 | 0.267138 | 0.001216 |
| AP1G2 | | 0.115328 | 0.620521 | 0.572848 | 0.598225 | 0.499456 | 0.001222 |
| CARS2 | | 0.125717 | 0.400963 | 0.367502 | 0.435356 | 0.359086 | 0.001238 |
| EPRS1 | | 0.133381 | 0.426482 | 0.38882 | 0.431518 | 0.353101 | 0.001243 |
| LINC02694 | | 1.220372 | 0.011798 | 0.005063 | 0.021348 | 0.008161 | 0.00125 |
| TMUB2 | | 0.117281 | 0.640125 | 0.590147 | 0.581195 | 0.492927 | 0.001255 |
| LCORL | | 0.119618 | 0.198138 | 0.182373 | 0.254977 | 0.200762 | 0.001255 |
| CCDC59 | | 0.128048 | 0.360111 | 0.329526 | 0.40993 | 0.333515 | 0.00126 |
| AATF | | 0.131997 | 0.489822 | 0.446996 | 0.51835 | 0.427639 | 0.001266 |
| PCM1 | | 0.120662 | 0.634198 | 0.583313 | 0.616215 | 0.522307 | 0.001277 |
| PRR14L | | 0.084494 | 0.307517 | 0.290024 | 0.363876 | 0.290533 | 0.001277 |
| DLD | | 0.106741 | 0.308869 | 0.286842 | 0.361478 | 0.291621 | 0.00128 |
| NUCB1 | | 0.150531 | 0.605733 | 0.545716 | 0.532022 | 0.459739 | 0.001284 |
| DUSP11 | | 0.132059 | 0.320214 | 0.292204 | 0.364596 | 0.294886 | 0.001284 |
| USP21 | | 0.112249 | 0.173977 | 0.160954 | 0.222595 | 0.17247 | 0.001284 |
| AGTPBP1 | | 0.105048 | 0.34933 | 0.324798 | 0.383545 | 0.312296 | 0.001284 |
| FAM91A1 | | 0.120562 | 0.436516 | 0.401521 | 0.477812 | 0.39173 | 0.001292 |
| NUP93 | | 0.109294 | 0.234806 | 0.217675 | 0.28616 | 0.226333 | 0.001292 |
| FAM98B | | 0.14427 | 0.270491 | 0.24475 | 0.322379 | 0.261153 | 0.001296 |
| EZH1 | | 0.131968 | 0.322094 | 0.293939 | 0.35716 | 0.291077 | 0.001301 |
| PPP1R16B | | 0.135997 | 0.570761 | 0.519415 | 0.541137 | 0.457018 | 0.001304 |
| TOMM22 | | 0.131546 | 0.384307 | 0.350816 | 0.424322 | 0.347116 | 0.001304 |
| DGAT1 | | 0.081011 | 0.182807 | 0.172825 | 0.235788 | 0.182807 | 0.001315 |
| MTMR14 | | 0.119047 | 0.566438 | 0.521574 | 0.55385 | 0.469532 | 0.001316 |
| PWP1 | | 0.124258 | 0.505559 | 0.463838 | 0.519309 | 0.433624 | 0.001316 |
| RTN2 | | 1.438781 | 0.008755 | 0.003229 | 0.01703 | 0.005441 | 0.001321 |
| VTI1B | | 0.131216 | 0.323576 | 0.295445 | 0.383545 | 0.311208 | 0.001321 |
| EXOC8 | | 0.158793 | 0.22067 | 0.197671 | 0.261454 | 0.207291 | 0.001325 |
| NMI | | 0.101886 | 0.410773 | 0.382765 | 0.447589 | 0.36235 | 0.001329 |
| AAGAB | | 0.136952 | 0.260993 | 0.237357 | 0.319501 | 0.257345 | 0.001332 |
| SMIM6 | | 65535 | 0.005587 | 0 | 0.006956 | 0 | 0.001336 |
| TAOK2 | | 0.092599 | 0.199714 | 0.187298 | 0.253298 | 0.196953 | 0.001343 |
| SAR1B | | 0.169143 | 0.277009 | 0.246364 | 0.305349 | 0.248096 | 0.001344 |
| SENP1 | | 0.112979 | 0.188393 | 0.174203 | 0.241785 | 0.188792 | 0.001347 |
| RBL2 | | 0.123523 | 0.632663 | 0.580749 | 0.60638 | 0.519042 | 0.00135 |
| RBM41 | | 0.087277 | 0.170943 | 0.160908 | 0.217318 | 0.167029 | 0.001351 |
| PDE6D | | 0.107921 | 0.232425 | 0.215673 | 0.280643 | 0.221436 | 0.001357 |
| DTNBP1 | | 0.093052 | 0.305404 | 0.286328 | 0.348525 | 0.280196 | 0.001362 |
| ABCG1 | | 0.203297 | 0.34203 | 0.297074 | 0.308947 | 0.254625 | 0.001362 |
| KLHL15 | | 0.112111 | 0.176154 | 0.162984 | 0.229072 | 0.177911 | 0.001373 |
| PPRC1 | | 0.084238 | 0.186906 | 0.176305 | 0.242744 | 0.188792 | 0.001374 |
| AVL9 | | 0.08835 | 0.277298 | 0.260826 | 0.329336 | 0.263874 | 0.001378 |
| XRCC5 | | 0.155063 | 0.207775 | 0.186601 | 0.270568 | 0.214908 | 0.001385 |
| AKAP8 | | 0.095373 | 0.361261 | 0.338151 | 0.415927 | 0.336235 | 0.001388 |
| TRMU | | 0.069814 | 0.224898 | 0.214274 | 0.279204 | 0.218716 | 0.001388 |
| GTF3C1 | | 0.109077 | 0.236705 | 0.219468 | 0.298153 | 0.237214 | 0.00139 |
| NCBP1 | | 0.110698 | 0.186577 | 0.172797 | 0.241545 | 0.188792 | 0.001393 |
| MAP4K5 | | 0.112141 | 0.284722 | 0.263429 | 0.331254 | 0.264962 | 0.001404 |
| FYTTD1 | | 0.124129 | 0.287673 | 0.263956 | 0.345167 | 0.279108 | 0.001406 |
| EHMT2 | | 0.10236 | 0.25234 | 0.235057 | 0.302231 | 0.241023 | 0.001409 |
| SLC25A44 | | 0.097714 | 0.170096 | 0.158957 | 0.220916 | 0.170838 | 0.001409 |
| CCDC186 | | 0.0885 | 0.234976 | 0.220995 | 0.298633 | 0.235582 | 0.001413 |
| CNN2 | | 0.086461 | 1.599718 | 1.506663 | 0.901415 | 0.845484 | 0.001419 |
| SIPA1L1 | | 0.150002 | 0.28962 | 0.26102 | 0.311346 | 0.252448 | 0.001422 |
| POLK | | 0.124975 | 0.268561 | 0.246276 | 0.313744 | 0.252448 | 0.001423 |
| ZNF335 | | 0.101649 | 0.175749 | 0.163792 | 0.228352 | 0.177367 | 0.001424 |
| THAP11 | | 0.110106 | 0.279069 | 0.258563 | 0.335572 | 0.269859 | 0.001428 |
| DENND1A | | 0.152857 | 0.27246 | 0.245069 | 0.32118 | 0.261153 | 0.00143 |
| OXR1 | | 0.109028 | 0.316033 | 0.29303 | 0.369153 | 0.295974 | 0.001431 |
| MINK1 | | 0.104364 | 0.525216 | 0.488564 | 0.526985 | 0.4358 | 0.001434 |
| CSTF2T | | 0.129599 | 0.292081 | 0.266987 | 0.341329 | 0.276931 | 0.001435 |
| RHBDF2 | | 0.122654 | 0.546136 | 0.501624 | 0.530103 | 0.443961 | 0.00146 |
| CEP170 | | 0.152104 | 0.22611 | 0.203485 | 0.257136 | 0.20457 | 0.001462 |
| IREB2 | | 0.092836 | 0.287744 | 0.269811 | 0.348285 | 0.277476 | 0.001466 |
| TRIM33 | | 0.095578 | 0.324174 | 0.303393 | 0.37443 | 0.300326 | 0.001467 |
| EDEM3 | | 0.159611 | 0.287302 | 0.257212 | 0.320461 | 0.258433 | 0.001477 |
| POLD2 | | 0.125463 | 0.27777 | 0.254635 | 0.309906 | 0.248096 | 0.001477 |
| ZRANB1 | | 0.110667 | 0.252948 | 0.23427 | 0.310386 | 0.249184 | 0.001484 |
| POLR2H | | 0.101347 | 0.322852 | 0.30095 | 0.358119 | 0.287269 | 0.001485 |
| OAS1 | | 0.12046 | 0.702937 | 0.646628 | 0.552171 | 0.465724 | 0.001485 |
| RAB10 | | 0.127751 | 0.582685 | 0.533306 | 0.566323 | 0.470076 | 0.001489 |
| QRICH1 | | 0.134999 | 0.647047 | 0.589247 | 0.617174 | 0.522307 | 0.001495 |
| EZH2 | | 0.138193 | 0.189149 | 0.171872 | 0.242024 | 0.18988 | 0.001498 |
| HEXA | | 0.122064 | 0.532506 | 0.489305 | 0.537299 | 0.448313 | 0.001509 |
| PEBP1 | | 0.162541 | 0.323283 | 0.288837 | 0.355721 | 0.290533 | 0.001511 |
| TIMM9 | | 0.078876 | 0.235217 | 0.222702 | 0.297673 | 0.23395 | 0.001512 |
| CHID1 | | 0.08088 | 0.237084 | 0.224158 | 0.287359 | 0.227965 | 0.001515 |
| RAD23B | | 0.130821 | 0.28575 | 0.260979 | 0.353322 | 0.286181 | 0.001521 |
| RNMT | | 0.120803 | 0.339037 | 0.311804 | 0.383065 | 0.310664 | 0.001521 |
| HARS1 | | 0.104821 | 0.283544 | 0.263673 | 0.340609 | 0.272035 | 0.001529 |
| SAE1 | | 0.086434 | 0.208304 | 0.196191 | 0.263612 | 0.205114 | 0.001538 |
| PHC3 | | 0.118738 | 0.578243 | 0.532558 | 0.583353 | 0.477693 | 0.001543 |
| THOC6 | | 0.105648 | 0.237731 | 0.220944 | 0.289278 | 0.230141 | 0.001543 |
| RNF111 | | 0.121585 | 0.291559 | 0.267994 | 0.33989 | 0.275843 | 0.001546 |
| USPL1 | | 0.104363 | 0.319598 | 0.297295 | 0.364116 | 0.294342 | 0.001546 |
| VPS26A | | 0.097792 | 0.2349 | 0.219505 | 0.295754 | 0.234494 | 0.001549 |
| NELFA | | 0.118263 | 0.31668 | 0.291756 | 0.369153 | 0.29815 | 0.00155 |
| LONRF1 | | 0.138637 | 0.203242 | 0.18462 | 0.238426 | 0.18716 | 0.001553 |
| RAD23A | | 0.13058 | 0.491614 | 0.449072 | 0.504198 | 0.422198 | 0.001555 |
| MCPH1 | | 0.091966 | 0.372032 | 0.349056 | 0.417606 | 0.335691 | 0.001558 |
| DMAC2L | | 0.059365 | 0.246121 | 0.236199 | 0.289758 | 0.226333 | 0.001563 |
| ARFRP1 | | 0.101185 | 0.24057 | 0.224276 | 0.295035 | 0.235582 | 0.001576 |
| SMARCA2 | | 0.118041 | 0.76999 | 0.709498 | 0.67858 | 0.573449 | 0.001582 |
| VTI1A | | 0.09754 | 0.302759 | 0.282966 | 0.359559 | 0.290533 | 0.001582 |
| ATP6V0C | | 0.137018 | 0.373769 | 0.339905 | 0.431518 | 0.357454 | 0.001589 |
| ARHGAP27 | | 0.136944 | 0.443725 | 0.403543 | 0.456225 | 0.386289 | 0.001598 |
| RUFY1 | | 0.125162 | 0.556016 | 0.509811 | 0.532502 | 0.45593 | 0.001606 |
| SHARPIN | | 0.110031 | 0.310701 | 0.287886 | 0.352123 | 0.285637 | 0.001624 |
| RELA | | 0.100284 | 0.276157 | 0.257613 | 0.335572 | 0.270403 | 0.001624 |
| TENT2 | | 0.10576 | 0.42113 | 0.391362 | 0.462221 | 0.374864 | 0.001626 |
| MAP3K4 | | 0.105231 | 0.240929 | 0.223981 | 0.2864 | 0.229597 | 0.001632 |
| TIMM17A | | 0.140043 | 0.315854 | 0.286635 | 0.351403 | 0.285637 | 0.001643 |
| COLGALT1 | | 0.139453 | 0.423585 | 0.384558 | 0.425762 | 0.354733 | 0.001645 |
| PTPRA | | 0.124971 | 0.360079 | 0.3302 | 0.411849 | 0.337867 | 0.001653 |
| DDA1 | | 0.102084 | 0.214216 | 0.199582 | 0.278244 | 0.220892 | 0.001669 |
| POLR1H | | 0.064602 | 0.165984 | 0.158715 | 0.226673 | 0.174646 | 0.001669 |
| WDR70 | | 0.093149 | 0.203376 | 0.19066 | 0.255697 | 0.200762 | 0.001675 |
| NEDD8 | | 0.091514 | 0.357816 | 0.335823 | 0.42888 | 0.34494 | 0.001676 |
| DPP8 | | 0.090289 | 0.248989 | 0.233884 | 0.299112 | 0.238303 | 0.00168 |
| NME3 | | 0.132617 | 0.268808 | 0.2452 | 0.318062 | 0.256801 | 0.001682 |
| FAM210A | | 0.097341 | 0.215504 | 0.201443 | 0.257856 | 0.203482 | 0.001682 |
| SRPK1 | | 0.085594 | 0.221531 | 0.20877 | 0.2864 | 0.225245 | 0.001686 |
| MALSU1 | | 0.115001 | 0.218815 | 0.20205 | 0.263612 | 0.208923 | 0.00169 |
| CCT8 | | 0.092877 | 0.215044 | 0.201636 | 0.288079 | 0.227421 | 0.001695 |
| NDUFC1 | | 0.159973 | 0.191347 | 0.171264 | 0.246342 | 0.194777 | 0.001703 |
| RAB33B | | 0.102213 | 0.216934 | 0.202096 | 0.263852 | 0.208379 | 0.001704 |
| CDK12 | | 0.106236 | 0.349208 | 0.324418 | 0.402974 | 0.325898 | 0.001705 |
| AGGF1 | | 0.089048 | 0.254962 | 0.2397 | 0.305349 | 0.243743 | 0.001705 |
| MAD2L1BP | | 0.060322 | 0.162802 | 0.156136 | 0.219717 | 0.169206 | 0.001705 |
| STRIP1 | | 0.131652 | 0.271364 | 0.247697 | 0.314224 | 0.255713 | 0.00171 |
| TRIM28 | | 0.152184 | 0.187318 | 0.168565 | 0.245383 | 0.194777 | 0.001721 |
| PSMD10 | | 0.124809 | 0.198201 | 0.181775 | 0.259295 | 0.205114 | 0.001722 |
| RFX3 | | 0.1201 | 0.364981 | 0.335827 | 0.377788 | 0.31012 | 0.001728 |
| ZP3 | | 1.639745 | 0.0092 | 0.002952 | 0.014872 | 0.004353 | 0.001739 |
| HERC2 | | 0.124063 | 0.204581 | 0.187723 | 0.264092 | 0.210555 | 0.001744 |
| NDUFB6 | | 0.121231 | 0.306647 | 0.281932 | 0.33965 | 0.273123 | 0.001744 |
| ANKRD52 | | 0.098595 | 0.269781 | 0.25196 | 0.327896 | 0.263874 | 0.001753 |
| FTSJ3 | | 0.113805 | 0.231378 | 0.213828 | 0.284001 | 0.227421 | 0.001753 |
| RABIF | | 0.049696 | 0.190369 | 0.183923 | 0.246582 | 0.191513 | 0.001775 |
| PNMA3 | | 1.148865 | 0.015999 | 0.007215 | 0.019189 | 0.007073 | 0.001782 |
| STK25 | | 0.131258 | 0.379073 | 0.346107 | 0.426481 | 0.355277 | 0.001788 |
| NDUFB4 | | 0.11685 | 0.158641 | 0.146298 | 0.227633 | 0.177367 | 0.00179 |
| RNF31 | | 0.082134 | 0.260093 | 0.2457 | 0.32118 | 0.256257 | 0.0018 |
| RANBP10 | | 0.1063 | 0.243482 | 0.226187 | 0.289278 | 0.231774 | 0.001806 |
| COG4 | | 0.104662 | 0.303817 | 0.282557 | 0.351643 | 0.284548 | 0.001818 |
| EMC2 | | 0.065493 | 0.167189 | 0.159769 | 0.230511 | 0.178999 | 0.001825 |
| MAIP1 | | 0.103667 | 0.184214 | 0.171442 | 0.245383 | 0.193145 | 0.001848 |
| TIPRL | | 0.081661 | 0.23555 | 0.222588 | 0.299112 | 0.236126 | 0.001854 |
| SNRPG | | 0.091286 | 0.176483 | 0.165662 | 0.240106 | 0.18716 | 0.001865 |
| TBCB | | 0.130115 | 0.597027 | 0.545539 | 0.55409 | 0.47062 | 0.001871 |
| SBNO2 | | 0.075822 | 0.317114 | 0.300878 | 0.366515 | 0.294342 | 0.001871 |
| DIMT1 | | 0.066247 | 0.240147 | 0.229369 | 0.282802 | 0.224701 | 0.001872 |
| CASS4 | | 1.482922 | 0.010582 | 0.003786 | 0.016551 | 0.005441 | 0.001884 |
| GTF3C6 | | 0.142667 | 0.268673 | 0.243376 | 0.328136 | 0.266594 | 0.001884 |
| TAPBPL | | 0.109548 | 0.210598 | 0.195198 | 0.270089 | 0.215452 | 0.001885 |
| TMEM115 | | 0.092634 | 0.246065 | 0.230762 | 0.297673 | 0.238303 | 0.001902 |
| UBXN2B | | 0.147639 | 0.235672 | 0.212748 | 0.277285 | 0.224157 | 0.001904 |
| ILF2 | | 0.118498 | 0.300606 | 0.276902 | 0.360518 | 0.289445 | 0.001929 |
| PNN | | 0.124839 | 0.625567 | 0.573711 | 0.600624 | 0.498912 | 0.001929 |
| CD55 | | 0.097051 | 1.382017 | 1.292105 | 0.886544 | 0.818281 | 0.001931 |
| IFNAR2 | | 0.140194 | 0.586256 | 0.531967 | 0.537539 | 0.453754 | 0.001933 |
| NABP2 | | 0.085764 | 0.187091 | 0.176293 | 0.236987 | 0.185528 | 0.001965 |
| PAN2 | | 0.108615 | 0.246525 | 0.228647 | 0.291437 | 0.234494 | 0.001966 |
| PSKH1 | | 0.095269 | 0.185067 | 0.173241 | 0.23147 | 0.181175 | 0.001971 |
| ZCCHC3 | | 0.110593 | 0.554271 | 0.51337 | 0.548333 | 0.45593 | 0.001975 |
| PELP1 | | 0.098815 | 0.207662 | 0.193915 | 0.262413 | 0.207291 | 0.001979 |
| TM9SF3 | | 0.154559 | 0.525469 | 0.472085 | 0.510914 | 0.43852 | 0.001983 |
| GOLGA7 | | 0.103969 | 0.286675 | 0.266742 | 0.344447 | 0.278564 | 0.001993 |
| AMBRA1 | | 0.111419 | 0.228322 | 0.211352 | 0.286639 | 0.230141 | 0.001997 |
| PHAX | | 0.092698 | 0.212951 | 0.199698 | 0.271048 | 0.214908 | 0.001997 |
| HEXIM1 | | 0.11816 | 0.501385 | 0.461957 | 0.502758 | 0.415125 | 0.001997 |
| RUNDC1 | | 0.11014 | 0.19847 | 0.183882 | 0.242024 | 0.190424 | 0.002003 |
| CTPS1 | | 0.112823 | 0.194453 | 0.179826 | 0.243464 | 0.191513 | 0.002011 |
| PRDX1 | | 0.154384 | 0.278932 | 0.250625 | 0.322859 | 0.262786 | 0.002029 |
| TRADD | | 0.089213 | 0.260814 | 0.245174 | 0.310866 | 0.247008 | 0.002035 |
| METTL23 | | 0.104513 | 0.404116 | 0.375876 | 0.427201 | 0.348205 | 0.002042 |
| PFKP | | 0.083738 | 0.454834 | 0.429186 | 0.455745 | 0.375408 | 0.002052 |
| AP2A2 | | 0.111098 | 0.282839 | 0.261876 | 0.329336 | 0.265506 | 0.002057 |
| CHD8 | | 0.115304 | 0.332258 | 0.306737 | 0.388343 | 0.316104 | 0.002078 |
| ABHD10 | | 0.128838 | 0.299307 | 0.273737 | 0.336052 | 0.275299 | 0.00208 |
| MRFAP1L1 | | 0.066568 | 0.308266 | 0.294365 | 0.368674 | 0.297062 | 0.00209 |
| NT5DC1 | | 0.10197 | 0.208357 | 0.194138 | 0.263612 | 0.209467 | 0.002093 |
| SCAMP4 | | 0.10121 | 0.39421 | 0.367503 | 0.422164 | 0.344396 | 0.002093 |
| RNF115 | | 0.080237 | 0.322405 | 0.304963 | 0.376109 | 0.301415 | 0.002116 |
| SUGT1 | | 0.112523 | 0.263625 | 0.243844 | 0.324778 | 0.261153 | 0.00212 |
| TNPO3 | | 0.110959 | 0.25853 | 0.239392 | 0.313504 | 0.252992 | 0.002122 |
| ANKRD13C | | 0.11217 | 0.191266 | 0.176959 | 0.238187 | 0.186616 | 0.002137 |
| MTMR3 | | 0.090704 | 0.315429 | 0.296208 | 0.362917 | 0.294886 | 0.002153 |
| TPGS1 | | 0.094176 | 0.230484 | 0.215919 | 0.280883 | 0.222524 | 0.002156 |
| MMS19 | | 0.062527 | 0.206054 | 0.197315 | 0.264572 | 0.207291 | 0.002161 |
| FIBP | | 0.111189 | 0.345576 | 0.319942 | 0.389062 | 0.317193 | 0.002165 |
| LRRC47 | | 0.100408 | 0.389427 | 0.363245 | 0.440633 | 0.35691 | 0.002165 |
| REV1 | | 0.099268 | 0.256818 | 0.239742 | 0.30415 | 0.244287 | 0.002166 |
| SLC39A3 | | 0.129074 | 0.257562 | 0.235519 | 0.310626 | 0.251904 | 0.00218 |
| TTC7B | | 2.437157 | 0.008304 | 0.001533 | 0.010794 | 0.002176 | 0.002235 |
| CDHR1 | | 2.018282 | 0.008159 | 0.002014 | 0.010794 | 0.002176 | 0.002255 |
| AP2M1 | | 0.099914 | 0.265555 | 0.247786 | 0.340369 | 0.272035 | 0.002265 |
| SUCO | | 0.120833 | 0.240161 | 0.220865 | 0.301751 | 0.243199 | 0.00227 |
| COPS4 | | 0.061052 | 0.195113 | 0.187029 | 0.259295 | 0.203482 | 0.002277 |
| SRSF1 | | 0.084768 | 0.238714 | 0.225092 | 0.304629 | 0.240479 | 0.002311 |
| APRT | | 0.11775 | 0.649617 | 0.598703 | 0.605181 | 0.508705 | 0.002331 |
| NUP98 | | 0.101931 | 0.361597 | 0.336931 | 0.417126 | 0.338411 | 0.002331 |
| TMEM185B | | 0.122443 | 0.276757 | 0.254237 | 0.298633 | 0.242111 | 0.002334 |
| TMEM205 | | 0.085684 | 0.18769 | 0.176867 | 0.245622 | 0.193689 | 0.002334 |
| COMMD9 | | 0.079426 | 0.201374 | 0.190587 | 0.259775 | 0.205114 | 0.002334 |
| TRAM1 | | 0.143542 | 0.870978 | 0.788491 | 0.666347 | 0.597933 | 0.002334 |
| VPS13B | | 0.117195 | 0.619672 | 0.571324 | 0.601583 | 0.510881 | 0.002334 |
| ZNF639 | | 0.103023 | 0.303215 | 0.282317 | 0.351643 | 0.287269 | 0.002335 |
| HNRNPR | | 0.099988 | 0.34086 | 0.318036 | 0.402974 | 0.32753 | 0.002335 |
| FAM89B | | 0.094712 | 0.420961 | 0.394212 | 0.441353 | 0.361262 | 0.002335 |
| STK11 | | 0.112516 | 0.407265 | 0.376709 | 0.444951 | 0.366159 | 0.002343 |
| PHF12 | | 0.088418 | 0.379576 | 0.357011 | 0.426001 | 0.347661 | 0.002345 |
| RAB27A | | 0.071489 | 0.255202 | 0.242865 | 0.293356 | 0.234494 | 0.002359 |
| INPP5A | | 0.158428 | 0.241706 | 0.216569 | 0.256896 | 0.204026 | 0.00236 |
| TMED7 | | 0.097387 | 0.356158 | 0.332909 | 0.399376 | 0.319369 | 0.00237 |
| KLHL21 | | 0.08996 | 0.200577 | 0.188452 | 0.247302 | 0.195865 | 0.002374 |
| FAM204A | | 0.102536 | 0.343007 | 0.319475 | 0.391941 | 0.322089 | 0.00241 |
| ZBP1 | | 0.12462 | 0.483937 | 0.443889 | 0.443272 | 0.373232 | 0.002417 |
| GMDS | | 0.094298 | 0.195244 | 0.18289 | 0.260734 | 0.206202 | 0.002419 |
| NDUFB5 | | 0.102065 | 0.458064 | 0.426777 | 0.48141 | 0.396627 | 0.00242 |
| CHD9 | | 0.10717 | 0.41803 | 0.388103 | 0.450708 | 0.37432 | 0.002427 |
| GLUD1 | | 0.105023 | 0.325772 | 0.3029 | 0.37491 | 0.305767 | 0.002428 |
| SRSF8 | | 0.104143 | 0.470323 | 0.437568 | 0.483569 | 0.403156 | 0.002428 |
| GTF3C2 | | 0.0756 | 0.221039 | 0.209754 | 0.282802 | 0.225789 | 0.00245 |
| ZC3H12D | | 0.143559 | 0.597295 | 0.540721 | 0.487167 | 0.433624 | 0.002453 |
| SSU72 | | 0.108112 | 0.317899 | 0.294947 | 0.385944 | 0.312296 | 0.002461 |
| PPIP5K2 | | 0.096852 | 0.283348 | 0.264951 | 0.345646 | 0.279652 | 0.002461 |
| USO1 | | 0.160592 | 0.328284 | 0.293702 | 0.355961 | 0.293254 | 0.002475 |
| BLOC1S2 | | 0.107649 | 0.515486 | 0.478423 | 0.503238 | 0.425462 | 0.00249 |
| UNK | | 0.092053 | 0.221382 | 0.207698 | 0.261693 | 0.208923 | 0.002491 |
| ZC3H18 | | 0.092414 | 0.467685 | 0.438666 | 0.492924 | 0.402612 | 0.002501 |
| AFG3L2 | | 0.128475 | 0.233627 | 0.213721 | 0.290237 | 0.235038 | 0.002502 |
| CD28 | | 3.972951 | 0.00185 | 0.000118 | 0.007436 | 0.000544 | 0.00252 |
| TMC6 | | 0.12764 | 0.686544 | 0.628413 | 0.632766 | 0.532644 | 0.002532 |
| EPSTI1 | | 0.108437 | 0.490467 | 0.454954 | 0.46366 | 0.389554 | 0.002536 |
| CCDC97 | | 0.116908 | 0.420139 | 0.387436 | 0.456225 | 0.37432 | 0.002564 |
| PRKACA | | 0.116413 | 0.53274 | 0.491441 | 0.518829 | 0.428727 | 0.002569 |
| CRK | | 0.083036 | 0.269244 | 0.254185 | 0.3207 | 0.258433 | 0.002581 |
| SIKE1 | | 0.07516 | 0.273466 | 0.259584 | 0.334613 | 0.269314 | 0.002592 |
| EXOC7 | | 0.112718 | 0.505886 | 0.467866 | 0.514752 | 0.434168 | 0.002609 |
| RNF7 | | 0.120056 | 0.546809 | 0.503147 | 0.541137 | 0.45593 | 0.002624 |
| CDC23 | | 0.119232 | 0.204296 | 0.188091 | 0.251619 | 0.200762 | 0.002636 |
| PSMD6 | | 0.111884 | 0.365656 | 0.33837 | 0.412089 | 0.334059 | 0.002636 |
| CERCAM | | 1.970845 | 0.008352 | 0.002131 | 0.01847 | 0.007073 | 0.002637 |
| SERP1 | | 0.142019 | 0.683848 | 0.619737 | 0.58959 | 0.520675 | 0.002641 |
| ENPP7 | | 1.029569 | 0.008592 | 0.004209 | 0.016071 | 0.005441 | 0.002661 |
| CTR9 | | 0.075515 | 0.286261 | 0.271663 | 0.353562 | 0.284004 | 0.002692 |
| NMD3 | | 0.095832 | 0.252823 | 0.236575 | 0.311825 | 0.252992 | 0.002699 |
| NPTN | | 0.07437 | 0.258465 | 0.245479 | 0.318302 | 0.254081 | 0.0027 |
| DFFA | | 0.064942 | 0.209168 | 0.199961 | 0.265291 | 0.211099 | 0.00274 |
| SSH2 | | 0.117094 | 0.386949 | 0.356784 | 0.412089 | 0.344396 | 0.002756 |
| TNKS2 | | 0.102134 | 0.334171 | 0.311332 | 0.387863 | 0.31556 | 0.002766 |
| MAT2A | | 0.115015 | 0.623653 | 0.575864 | 0.574238 | 0.492383 | 0.00277 |
| CRCP | | 0.129117 | 0.225868 | 0.206532 | 0.281602 | 0.227421 | 0.002771 |
| SLC25A28 | | 0.107695 | 0.360686 | 0.334742 | 0.402495 | 0.331882 | 0.002771 |
| PPM1K | | 0.107514 | 0.548423 | 0.509038 | 0.525066 | 0.443417 | 0.002795 |
| STARD8 | | 1.411094 | 0.005359 | 0.002015 | 0.015112 | 0.004897 | 0.002796 |
| RHOBTB3 | | 1.267461 | 0.010612 | 0.004408 | 0.016791 | 0.005985 | 0.002812 |
| PPP1CB | | 0.134935 | 0.644661 | 0.5871 | 0.632046 | 0.536453 | 0.002832 |
| USP28 | | 0.112268 | 0.399213 | 0.369325 | 0.42816 | 0.353101 | 0.002836 |
| PRCC | | 0.117398 | 0.37155 | 0.342513 | 0.419765 | 0.34494 | 0.002852 |
| RARG | | 1.169814 | 0.013026 | 0.00579 | 0.01847 | 0.007073 | 0.002863 |
| ELP2 | | 0.092152 | 0.402041 | 0.377163 | 0.450228 | 0.370511 | 0.002865 |
| NSMCE2 | | 0.050674 | 0.183582 | 0.177245 | 0.251139 | 0.196409 | 0.002865 |
| CAPZB | | 0.117849 | 0.928359 | 0.855539 | 0.739746 | 0.664309 | 0.002883 |
| SRSF2 | | 0.130547 | 0.651052 | 0.594726 | 0.60638 | 0.522307 | 0.002897 |
| SSTR3 | | 1.479192 | 0.011247 | 0.004034 | 0.019189 | 0.007617 | 0.002909 |
| GZMB | | 2.39985 | 0.002113 | 0.0004 | 0.008395 | 0.001088 | 0.002919 |
| STK38L | | 0.105995 | 0.21836 | 0.202892 | 0.272967 | 0.218716 | 0.002949 |
| CYB5B | | 0.101422 | 0.447416 | 0.417043 | 0.473015 | 0.39173 | 0.002953 |
| RBM27 | | 0.108096 | 0.342775 | 0.318031 | 0.397697 | 0.32481 | 0.002968 |
| FCRL4 | | 65535 | 0.00488 | 0 | 0.005997 | 0 | 0.002968 |
| LARP7 | | 0.064801 | 0.320067 | 0.306009 | 0.37587 | 0.300326 | 0.002968 |
| PHF5A | | 0.073251 | 0.287472 | 0.27324 | 0.33965 | 0.272579 | 0.002986 |
| PDLIM1 | | 0.12554 | 0.785554 | 0.720086 | 0.665627 | 0.577802 | 0.002996 |
| FARSA | | 0.115133 | 0.341269 | 0.315092 | 0.382826 | 0.316104 | 0.003004 |
| TIMM23 | | 0.087995 | 0.317163 | 0.298396 | 0.367234 | 0.296518 | 0.003004 |
| PAM | | 1.483197 | 0.009379 | 0.003355 | 0.01751 | 0.006529 | 0.003011 |
| CCDC6 | | 0.116367 | 0.497736 | 0.459165 | 0.5006 | 0.421654 | 0.003011 |
| PRPSAP2 | | 0.079074 | 0.282016 | 0.266975 | 0.346606 | 0.278564 | 0.003011 |
| RTRAF | | 0.093818 | 0.269944 | 0.252948 | 0.33917 | 0.272579 | 0.003058 |
| BAZ2A | | 0.122263 | 0.484917 | 0.445515 | 0.508515 | 0.431447 | 0.003064 |
| MTREX | | 0.107771 | 0.258476 | 0.239871 | 0.323339 | 0.263874 | 0.003087 |
| ABCB9 | | 1.109644 | 0.008242 | 0.003819 | 0.015831 | 0.005441 | 0.003095 |
| AKAP13 | | 0.090174 | 0.982815 | 0.923266 | 0.769489 | 0.701306 | 0.003099 |
| ATR | | 0.089733 | 0.249204 | 0.234176 | 0.30271 | 0.244287 | 0.003117 |
| L3MBTL2 | | 0.077413 | 0.209583 | 0.198633 | 0.266731 | 0.211099 | 0.003117 |
| APOBEC3F | | 0.095501 | 0.218799 | 0.204785 | 0.273207 | 0.219804 | 0.00313 |
| FAM168B | | 0.083749 | 0.342086 | 0.322793 | 0.398897 | 0.323721 | 0.003145 |
| MAP2K3 | | 0.113339 | 0.516684 | 0.477647 | 0.523147 | 0.439064 | 0.003153 |
| ACSF3 | | 0.080189 | 0.414534 | 0.392121 | 0.443991 | 0.363439 | 0.003162 |
| NOL9 | | 0.090804 | 0.260499 | 0.244608 | 0.310386 | 0.250272 | 0.003172 |
| NCOA6 | | 0.07887 | 0.289711 | 0.274298 | 0.342048 | 0.276387 | 0.003203 |
| CHD1 | | 0.105869 | 0.483773 | 0.449544 | 0.498921 | 0.420022 | 0.003211 |
| SPG7 | | 0.095137 | 0.362533 | 0.339398 | 0.407052 | 0.336235 | 0.003217 |
| COA4 | | 0.073629 | 0.278385 | 0.264534 | 0.330535 | 0.265506 | 0.003219 |
| SNX25 | | 0.120218 | 0.210401 | 0.193579 | 0.265052 | 0.213819 | 0.003221 |
| NDUFV3 | | 0.104956 | 0.271379 | 0.252337 | 0.316863 | 0.257345 | 0.003228 |
| EMC1 | | 0.101017 | 0.19305 | 0.179995 | 0.247062 | 0.196409 | 0.003258 |
| EMC4 | | 0.138045 | 0.62751 | 0.570249 | 0.57136 | 0.48531 | 0.003262 |
| PRR12 | | 0.101354 | 0.374229 | 0.34884 | 0.419765 | 0.346028 | 0.003265 |
| RDH11 | | 0.104631 | 0.219804 | 0.204427 | 0.271288 | 0.218716 | 0.003268 |
| ARL6IP1 | | 0.100268 | 0.244038 | 0.227653 | 0.315663 | 0.255169 | 0.003283 |
| DYNLL2 | | 0.105596 | 0.588031 | 0.546528 | 0.568242 | 0.481502 | 0.003305 |
| KDM7A | | 0.132779 | 0.397526 | 0.362573 | 0.407292 | 0.335691 | 0.003306 |
| PGLS | | 0.113764 | 0.46395 | 0.42877 | 0.46486 | 0.39173 | 0.003312 |
| TPR | | 0.100978 | 0.426551 | 0.397717 | 0.470377 | 0.385745 | 0.003315 |
| ZZZ3 | | 0.060358 | 0.206247 | 0.197797 | 0.260494 | 0.206202 | 0.003335 |
| C21orf91 | | 0.049735 | 0.217533 | 0.210162 | 0.280163 | 0.22198 | 0.003345 |
| ATP5IF1 | | 0.118551 | 0.30148 | 0.277697 | 0.362197 | 0.294342 | 0.00335 |
| SEC11A | | 0.132735 | 0.565496 | 0.51579 | 0.533941 | 0.456474 | 0.00335 |
| LMBRD1 | | 0.116107 | 0.702482 | 0.648162 | 0.645958 | 0.563656 | 0.00335 |
| PRPF31 | | 0.094763 | 0.356402 | 0.333744 | 0.400096 | 0.326442 | 0.00335 |
| PPIE | | 0.08632 | 0.201475 | 0.189774 | 0.259055 | 0.206746 | 0.003353 |
| EIPR1 | | 0.082336 | 0.22137 | 0.20909 | 0.271288 | 0.217628 | 0.003386 |
| EXOC3 | | 0.082063 | 0.486408 | 0.459513 | 0.507076 | 0.41839 | 0.003392 |
| HSP90B1 | | 0.192789 | 0.395656 | 0.346164 | 0.329815 | 0.279108 | 0.003393 |
| ISY1 | | 0.110499 | 0.404832 | 0.374983 | 0.442072 | 0.365615 | 0.003404 |
| DEPDC5 | | 0.112661 | 0.232475 | 0.215012 | 0.270329 | 0.217084 | 0.00345 |
| ATXN7L3 | | 0.109816 | 0.252994 | 0.234451 | 0.307988 | 0.25136 | 0.003452 |
| DDX39B | | 0.10079 | 0.571675 | 0.533099 | 0.580475 | 0.483678 | 0.003461 |
| PRSS1 | | 1.7462 | 0.01198 | 0.003571 | 0.013912 | 0.004353 | 0.003473 |
| FH | | 0.103131 | 0.245441 | 0.228508 | 0.294555 | 0.237214 | 0.003475 |
| C11orf68 | | 0.094663 | 0.361 | 0.338074 | 0.396498 | 0.326442 | 0.003498 |
| MYSM1 | | 0.100198 | 0.397564 | 0.37089 | 0.42888 | 0.355277 | 0.003502 |
| PCK2 | | 0.073894 | 0.229042 | 0.217606 | 0.274406 | 0.218716 | 0.003521 |
| BBLN | | 0.114544 | 0.53011 | 0.489649 | 0.521228 | 0.4358 | 0.003524 |
| CNOT11 | | 0.099802 | 0.303994 | 0.283676 | 0.365076 | 0.298694 | 0.003563 |
| UPB1 | | 1.488174 | 0.014299 | 0.005097 | 0.015591 | 0.005441 | 0.003569 |
| SNX13 | | 0.11029 | 0.271014 | 0.251068 | 0.316863 | 0.259521 | 0.003586 |
| COQ4 | | 0.095952 | 0.233293 | 0.218281 | 0.283281 | 0.229597 | 0.003616 |
| UBE3C | | 0.08902 | 0.316838 | 0.297879 | 0.37587 | 0.305223 | 0.003637 |
| DNAJA1 | | 0.092239 | 0.375544 | 0.352285 | 0.420964 | 0.345484 | 0.003639 |
| SPTY2D1 | | 0.134326 | 0.282228 | 0.257137 | 0.329336 | 0.272579 | 0.003642 |
| GSDMD | | 0.089761 | 0.38077 | 0.357801 | 0.425522 | 0.346572 | 0.003644 |
| RTN4RL1 | | 65535 | 0.003672 | 0 | 0.005757 | 0 | 0.00365 |
| RFX7 | | 0.126877 | 0.263174 | 0.241018 | 0.305349 | 0.25136 | 0.003699 |
| ANAPC7 | | 0.085534 | 0.212165 | 0.199952 | 0.273687 | 0.21926 | 0.003731 |
| STK10 | | 0.104419 | 0.80355 | 0.747446 | 0.700168 | 0.604461 | 0.003748 |
| SCFD1 | | 0.102731 | 0.356178 | 0.331697 | 0.398897 | 0.326442 | 0.00377 |
| PSMD7 | | 0.109924 | 0.373448 | 0.346051 | 0.414488 | 0.337867 | 0.003772 |
| BCR | | 0.096746 | 0.307213 | 0.287287 | 0.351163 | 0.287813 | 0.003772 |
| SPSB3 | | 0.088722 | 0.401035 | 0.377116 | 0.434637 | 0.357454 | 0.003772 |
| SLC4A7 | | 0.122954 | 0.247376 | 0.227167 | 0.273207 | 0.219804 | 0.003774 |
| WDR81 | | 0.092271 | 0.243933 | 0.22882 | 0.295754 | 0.239391 | 0.003784 |
| TP53BP2 | | 0.073586 | 0.216935 | 0.206147 | 0.262413 | 0.210011 | 0.003792 |
| TTC1 | | 0.08195 | 0.333582 | 0.315161 | 0.390022 | 0.317193 | 0.003801 |
| GOPC | | 0.080311 | 0.331728 | 0.313766 | 0.385464 | 0.315016 | 0.003803 |
| KLF4 | | 0.137812 | 0.573389 | 0.521151 | 0.430319 | 0.371055 | 0.003815 |
| DENND4A | | 0.089241 | 0.931513 | 0.875638 | 0.726313 | 0.638194 | 0.00382 |
| TKTL2 | | 2.02221 | 0.007495 | 0.001845 | 0.009115 | 0.001632 | 0.003844 |
| EDC4 | | 0.055092 | 0.26466 | 0.254744 | 0.323099 | 0.260609 | 0.003857 |
| TM2D3 | | 0.115604 | 0.211504 | 0.195217 | 0.262653 | 0.212187 | 0.003868 |
| LIPN | | 1.585892 | 0.008098 | 0.002698 | 0.011993 | 0.003264 | 0.003878 |
| ZRANB2 | | 0.100522 | 0.479448 | 0.447179 | 0.498201 | 0.412949 | 0.003879 |
| CNDP2 | | 0.101937 | 0.643007 | 0.599142 | 0.597266 | 0.505985 | 0.003886 |
| PEX13 | | 0.096607 | 0.216832 | 0.202788 | 0.269609 | 0.218172 | 0.003902 |
| GLS | | 0.07352 | 0.301632 | 0.286646 | 0.372991 | 0.300871 | 0.003903 |
| RAB24 | | 0.079402 | 0.223152 | 0.211203 | 0.274646 | 0.22198 | 0.003907 |
| AP3D1 | | 0.111651 | 0.417316 | 0.386238 | 0.44687 | 0.367247 | 0.003913 |
| CD274 | | 1.585215 | 0.004748 | 0.001582 | 0.011034 | 0.00272 | 0.00393 |
| TMEM59 | | 0.109828 | 0.290219 | 0.268945 | 0.351643 | 0.285637 | 0.003935 |
| RSU1 | | 0.083549 | 0.396089 | 0.373802 | 0.437035 | 0.356366 | 0.00395 |
| JCHAIN | | 0.260371 | 1.390935 | 1.161254 | 0.649316 | 0.590316 | 0.003954 |
| RBM19 | | 0.078501 | 0.35849 | 0.339505 | 0.401055 | 0.328618 | 0.003974 |
| NDUFA2 | | 0.122181 | 0.724811 | 0.665955 | 0.610938 | 0.52938 | 0.003987 |
| PPTC7 | | 0.08305 | 0.424121 | 0.400395 | 0.465819 | 0.386834 | 0.003987 |
| HELZ | | 0.090771 | 0.426258 | 0.400265 | 0.46462 | 0.385745 | 0.003991 |
| LTV1 | | 0.064367 | 0.191661 | 0.183298 | 0.24946 | 0.198041 | 0.004024 |
| RPN2 | | 0.161979 | 0.333556 | 0.298132 | 0.336532 | 0.282916 | 0.004028 |
| GPATCH8 | | 0.10083 | 0.363586 | 0.339042 | 0.402974 | 0.334603 | 0.00403 |
| EFR3A | | 0.088606 | 0.412785 | 0.388196 | 0.451187 | 0.376496 | 0.004037 |
| USF1 | | 0.106209 | 0.520687 | 0.483732 | 0.521948 | 0.44124 | 0.004059 |
| SETDB2 | | 0.08906 | 0.339529 | 0.319203 | 0.384505 | 0.314472 | 0.004066 |
| LIMD1 | | 0.112306 | 0.734913 | 0.679874 | 0.645958 | 0.54951 | 0.004078 |
| SLF2 | | 0.069152 | 0.213508 | 0.203516 | 0.256177 | 0.205114 | 0.004094 |
| ASB3 | | 0.059418 | 0.193834 | 0.186013 | 0.254018 | 0.20185 | 0.004105 |
| NAA50 | | 0.066809 | 0.178511 | 0.170433 | 0.243464 | 0.192601 | 0.004107 |
| GTF2F1 | | 0.072513 | 0.318898 | 0.303266 | 0.377069 | 0.304135 | 0.004109 |
| CUL2 | | 0.077181 | 0.222217 | 0.210641 | 0.278004 | 0.225245 | 0.004156 |
| NCBP2 | | 0.109121 | 0.513156 | 0.475774 | 0.510674 | 0.428183 | 0.004158 |
| TUBB4B | | 0.077659 | 0.218947 | 0.207473 | 0.273447 | 0.218172 | 0.00417 |
| CHAMP1 | | 0.066867 | 0.240849 | 0.229941 | 0.291197 | 0.233406 | 0.00417 |
| SLC25A38 | | 0.080127 | 0.381085 | 0.360496 | 0.411609 | 0.339499 | 0.004202 |
| CST7 | | 3.725451 | 0.002388 | 0.000181 | 0.007916 | 0.001088 | 0.004254 |
| GLG1 | | 0.09499 | 0.315339 | 0.295245 | 0.384025 | 0.314472 | 0.004279 |
| GNPTAB | | 0.070045 | 0.291312 | 0.277506 | 0.33893 | 0.275843 | 0.004282 |
| SNRNP70 | | 0.091485 | 0.489492 | 0.459416 | 0.519069 | 0.433079 | 0.0043 |
| PHTF2 | | 0.104939 | 0.62998 | 0.585783 | 0.587911 | 0.503808 | 0.004305 |
| LANCL1 | | 0.111862 | 0.23309 | 0.215699 | 0.274406 | 0.224157 | 0.004324 |
| STMP1 | | 0.099357 | 0.469551 | 0.438302 | 0.494123 | 0.412405 | 0.004338 |
| LNPEP | | 0.109445 | 0.634629 | 0.588266 | 0.591509 | 0.514146 | 0.004347 |
| PRPF18 | | 0.042316 | 0.214651 | 0.208446 | 0.275606 | 0.219804 | 0.004347 |
| PDP1 | | 0.110561 | 0.245263 | 0.22717 | 0.290717 | 0.238303 | 0.004367 |
| E4F1 | | 0.0461 | 0.208877 | 0.202308 | 0.263612 | 0.210011 | 0.004403 |
| DENND1C | | 0.102289 | 0.525257 | 0.489305 | 0.51811 | 0.439064 | 0.004409 |
| PPP1R10 | | 0.08252 | 0.292195 | 0.275951 | 0.334852 | 0.272579 | 0.004432 |
| NDUFA13 | | 0.121612 | 0.279361 | 0.256777 | 0.346126 | 0.281828 | 0.004462 |
| DDHD1 | | 0.085169 | 0.371263 | 0.34998 | 0.417366 | 0.344396 | 0.004488 |
| DCAF15 | | 0.104888 | 0.289477 | 0.269178 | 0.328856 | 0.269859 | 0.00454 |
| CRNKL1 | | 0.078769 | 0.191656 | 0.181472 | 0.251859 | 0.201306 | 0.00456 |
| UHMK1 | | 0.111072 | 0.492575 | 0.456075 | 0.51787 | 0.436888 | 0.004563 |
| EID1 | | 0.115135 | 0.693534 | 0.640337 | 0.62557 | 0.542437 | 0.004585 |
| TRAPPC12 | | 0.070758 | 0.311072 | 0.296183 | 0.363396 | 0.29815 | 0.004609 |
| FBXO33 | | 0.112358 | 0.278682 | 0.257802 | 0.328856 | 0.270947 | 0.004653 |
| MARF1 | | 0.093248 | 0.414354 | 0.38842 | 0.434876 | 0.35963 | 0.004665 |
| MIOS | | 0.08843 | 0.355434 | 0.334302 | 0.391701 | 0.322633 | 0.004685 |
| AKAP8L | | 0.104281 | 0.401945 | 0.373917 | 0.440873 | 0.367791 | 0.004688 |
| LMF2 | | 0.05706 | 0.214344 | 0.206032 | 0.282322 | 0.227421 | 0.004688 |
| CRMP1 | | 1.952356 | 0.011709 | 0.003026 | 0.010794 | 0.00272 | 0.004693 |
| CDC25B | | 0.101714 | 0.668199 | 0.622711 | 0.573519 | 0.482046 | 0.004715 |
| CCT3 | | 0.05108 | 0.173086 | 0.167065 | 0.243943 | 0.190968 | 0.004741 |
| MFSD10 | | 0.086943 | 0.280628 | 0.264215 | 0.32214 | 0.261697 | 0.004763 |
| USP19 | | 0.062481 | 0.253917 | 0.243155 | 0.310866 | 0.25136 | 0.004763 |
| FBXO42 | | 0.076291 | 0.269246 | 0.255378 | 0.324059 | 0.265506 | 0.004766 |
| PDHA1 | | 0.121688 | 0.48585 | 0.44655 | 0.483329 | 0.404244 | 0.004805 |
| ZMYM4 | | 0.095018 | 0.257624 | 0.241203 | 0.308227 | 0.252448 | 0.004805 |
| TAFAZZIN | | 0.099926 | 0.308023 | 0.28741 | 0.347565 | 0.287269 | 0.004807 |
| EDEM2 | | 0.135762 | 0.387769 | 0.352943 | 0.401295 | 0.336235 | 0.004814 |
| VPS45 | | 0.10645 | 0.260905 | 0.242347 | 0.292876 | 0.239391 | 0.00484 |
| CIAO2A | | 0.122968 | 0.514295 | 0.472276 | 0.525306 | 0.451034 | 0.004848 |
| UBE2L6 | | 0.105739 | 0.676955 | 0.629114 | 0.604941 | 0.51469 | 0.004848 |
| SUMF2 | | 0.116458 | 0.565868 | 0.521984 | 0.548333 | 0.466812 | 0.004853 |
| SREBF2 | | 0.098916 | 0.583556 | 0.544886 | 0.546414 | 0.457018 | 0.004871 |
| DCPS | | 0.122032 | 0.322267 | 0.296129 | 0.320461 | 0.262242 | 0.004884 |
| DCP1A | | 0.115239 | 0.247007 | 0.228045 | 0.301991 | 0.249184 | 0.004886 |
| ANKRD12 | | 0.086385 | 0.359627 | 0.338725 | 0.407532 | 0.336235 | 0.004896 |
| POLR2C | | 0.074942 | 0.228882 | 0.217296 | 0.293596 | 0.237214 | 0.004896 |
| PUS7L | | 0.040482 | 0.193679 | 0.188319 | 0.24994 | 0.199674 | 0.004905 |
| SMCR8 | | 0.063325 | 0.253842 | 0.242941 | 0.305349 | 0.247552 | 0.004928 |
| MARCKS | | 0.153775 | 0.382001 | 0.343379 | 0.334133 | 0.282372 | 0.004931 |
| RABL6 | | 0.101237 | 0.380183 | 0.354419 | 0.419285 | 0.344396 | 0.004931 |
| TBRG1 | | 0.095441 | 0.392878 | 0.367728 | 0.441593 | 0.368879 | 0.004933 |
| CAND1 | | 0.077394 | 0.35409 | 0.335595 | 0.41065 | 0.334603 | 0.004934 |
| TAF13 | | 0.079078 | 0.241155 | 0.228292 | 0.297673 | 0.240479 | 0.004946 |
| ASCC2 | | 0.076925 | 0.310528 | 0.294404 | 0.364116 | 0.296518 | 0.004991 |
| PCCB | | 0.084201 | 0.217839 | 0.205489 | 0.26721 | 0.215996 | 0.005033 |
| PATL1 | | 0.073712 | 0.214174 | 0.203506 | 0.263852 | 0.211643 | 0.005063 |
| LAS1L | | 0.104279 | 0.233202 | 0.21694 | 0.283761 | 0.230686 | 0.00507 |
| RRP36 | | 0.09228 | 0.342404 | 0.321188 | 0.383065 | 0.313384 | 0.005077 |
| KCNG1 | | 1.117085 | 0.019885 | 0.009167 | 0.019189 | 0.008161 | 0.00508 |
| MED4 | | 0.07047 | 0.395964 | 0.377088 | 0.445431 | 0.363983 | 0.00508 |
| CKS2 | | 0.077364 | 0.214287 | 0.203098 | 0.254977 | 0.204026 | 0.005083 |
| ATXN1L | | 0.088319 | 0.326023 | 0.306663 | 0.383305 | 0.316104 | 0.005103 |
| SRSF7 | | 0.094311 | 0.526615 | 0.493291 | 0.534421 | 0.443417 | 0.005113 |
| PGAM5 | | 0.061343 | 0.248731 | 0.238377 | 0.298873 | 0.241023 | 0.005141 |
| C12orf60 | | 1.154631 | 0.019768 | 0.00888 | 0.025905 | 0.013058 | 0.005149 |
| PGS1 | | 0.098277 | 0.227674 | 0.212681 | 0.279444 | 0.227965 | 0.005149 |
| NUBP2 | | 0.064254 | 0.239163 | 0.228745 | 0.299832 | 0.242111 | 0.005181 |
| PKN2 | | 0.114938 | 0.288248 | 0.266174 | 0.333173 | 0.276931 | 0.005221 |
| ZNF664 | | 0.089146 | 0.203727 | 0.19152 | 0.264812 | 0.212731 | 0.005222 |
| LHPP | | 0.088133 | 0.250137 | 0.235313 | 0.294555 | 0.239391 | 0.005228 |
| TIAL1 | | 0.104698 | 0.645761 | 0.600558 | 0.616215 | 0.520131 | 0.005239 |
| MLH1 | | 0.093079 | 0.216585 | 0.203053 | 0.265771 | 0.215452 | 0.005292 |
| CAPZA1 | | 0.080553 | 0.327085 | 0.309322 | 0.39338 | 0.319913 | 0.00532 |
| CYTH4 | | 0.093325 | 0.611985 | 0.57365 | 0.573039 | 0.477693 | 0.005341 |
| CARD11 | | 0.116774 | 0.721519 | 0.665419 | 0.640681 | 0.541349 | 0.005351 |
| UPF1 | | 0.070805 | 0.362285 | 0.344934 | 0.413528 | 0.337867 | 0.005394 |
| LMBR1L | | 0.083656 | 0.281884 | 0.266003 | 0.32118 | 0.260609 | 0.005396 |
| ABTB1 | | 0.132731 | 0.269581 | 0.245886 | 0.293356 | 0.242111 | 0.0054 |
| SMC6 | | 0.106261 | 0.787998 | 0.732044 | 0.645718 | 0.57617 | 0.005402 |
| CABIN1 | | 0.114043 | 0.247322 | 0.228524 | 0.295994 | 0.242655 | 0.00541 |
| HUWE1 | | 0.096966 | 0.490319 | 0.458446 | 0.514752 | 0.425462 | 0.005425 |
| RTN4 | | 0.082211 | 0.28256 | 0.266909 | 0.347565 | 0.286725 | 0.005457 |
| THAP12 | | 0.075427 | 0.270845 | 0.257049 | 0.318062 | 0.260065 | 0.00553 |
| ZNHIT1 | | 0.090108 | 0.29653 | 0.278576 | 0.355721 | 0.291621 | 0.005566 |
| AEN | | 0.066543 | 0.341173 | 0.325794 | 0.370592 | 0.299238 | 0.005569 |
| YME1L1 | | 0.103947 | 0.228041 | 0.212189 | 0.298873 | 0.243743 | 0.005571 |
| TCF4 | | 0.104422 | 0.85266 | 0.793125 | 0.705205 | 0.612078 | 0.005574 |
| NDUFB2 | | 0.054161 | 0.189236 | 0.182263 | 0.263852 | 0.211099 | 0.005625 |
| TGFB1 | | 0.093434 | 0.303713 | 0.284667 | 0.352603 | 0.289445 | 0.005643 |
| SLC12A8 | | 3.167131 | 0.003395 | 0.000378 | 0.006476 | 0.000544 | 0.005663 |
| DPH5 | | 0.087914 | 0.284559 | 0.267736 | 0.306548 | 0.250816 | 0.005675 |
| SPEN | | 0.084385 | 0.535025 | 0.504628 | 0.55337 | 0.458651 | 0.005697 |
| ZZEF1 | | 0.098563 | 0.442626 | 0.413396 | 0.476373 | 0.399891 | 0.005703 |
| CDS2 | | 0.087948 | 0.294608 | 0.277185 | 0.354761 | 0.294342 | 0.005724 |
| GPC4 | | 1.945377 | 0.010069 | 0.002614 | 0.009595 | 0.002176 | 0.005735 |
| HEATR1 | | 0.07178 | 0.272017 | 0.258814 | 0.325258 | 0.267138 | 0.005789 |
| TMED5 | | 0.11846 | 0.406703 | 0.374643 | 0.415687 | 0.350925 | 0.005808 |
| DCTN4 | | 0.065665 | 0.265031 | 0.253239 | 0.325258 | 0.264418 | 0.00588 |
| DPH3 | | 0.054437 | 0.242593 | 0.23361 | 0.292636 | 0.23667 | 0.005881 |
| CUL1 | | 0.082949 | 0.370459 | 0.34976 | 0.424562 | 0.348749 | 0.005882 |
| CASC3 | | 0.091235 | 0.474152 | 0.445096 | 0.502758 | 0.420566 | 0.005882 |
| C19orf12 | | 0.041867 | 0.234762 | 0.228047 | 0.28592 | 0.231774 | 0.005908 |
| C9orf72 | | 0.129047 | 0.280584 | 0.256576 | 0.289998 | 0.238847 | 0.00595 |
| YTHDF3 | | 0.074143 | 0.413559 | 0.392842 | 0.455985 | 0.374864 | 0.00596 |
| PRNP | | 0.099555 | 0.319733 | 0.298413 | 0.351403 | 0.292165 | 0.006018 |
| TDP2 | | 0.084864 | 0.295423 | 0.278546 | 0.344447 | 0.285092 | 0.006059 |
| AP2S1 | | 0.093744 | 0.221538 | 0.207601 | 0.296474 | 0.241023 | 0.006065 |
| NUDCD3 | | 0.080651 | 0.31196 | 0.294999 | 0.360278 | 0.296518 | 0.006079 |
| TUT7 | | 0.1032 | 0.953305 | 0.887495 | 0.726073 | 0.648531 | 0.006094 |
| TMEM265 | | 1.257735 | 0.012906 | 0.005397 | 0.01727 | 0.007073 | 0.006109 |
| HSD17B4 | | 0.074455 | 0.415405 | 0.39451 | 0.439914 | 0.361262 | 0.006133 |
| OCIAD2 | | 0.080276 | 0.326131 | 0.308479 | 0.381866 | 0.314472 | 0.006177 |
| TLCD3A | | 1.216562 | 0.012055 | 0.005187 | 0.01727 | 0.007073 | 0.006186 |
| GORASP1 | | 0.081905 | 0.246197 | 0.232609 | 0.28544 | 0.232318 | 0.006207 |
| GBF1 | | 0.094332 | 0.203659 | 0.190769 | 0.266731 | 0.215452 | 0.006272 |
| RHOF | | 0.116558 | 0.301123 | 0.277752 | 0.343488 | 0.286181 | 0.006277 |
| FARSB | | 0.048337 | 0.224692 | 0.217289 | 0.278964 | 0.224701 | 0.006325 |
| STK24 | | 0.097054 | 0.717446 | 0.670769 | 0.645479 | 0.557127 | 0.006357 |
| DECR1 | | 0.110966 | 0.493757 | 0.457203 | 0.503958 | 0.428183 | 0.006378 |
| SH2B1 | | 0.086393 | 0.376133 | 0.35427 | 0.414248 | 0.345484 | 0.006378 |
| PHKG2 | | 0.033995 | 0.201118 | 0.196434 | 0.259055 | 0.206746 | 0.006378 |
| IK | | 0.094 | 0.535379 | 0.501608 | 0.539698 | 0.447225 | 0.006389 |
| FASTKD2 | | 0.082733 | 0.220636 | 0.208339 | 0.274886 | 0.223069 | 0.006426 |
| IQGAP1 | | 0.109185 | 0.34505 | 0.3199 | 0.398177 | 0.332427 | 0.006429 |
| DNAJC11 | | 0.052991 | 0.220376 | 0.212428 | 0.272727 | 0.219804 | 0.006447 |
| MTHFR | | 0.059263 | 0.237275 | 0.227725 | 0.273927 | 0.219804 | 0.006513 |
| SENP3 | | 0.064918 | 0.200605 | 0.191778 | 0.26721 | 0.214908 | 0.006546 |
| MARCHF5 | | 0.097891 | 0.332603 | 0.310784 | 0.369153 | 0.309576 | 0.006568 |
| PDIA3 | | 0.133741 | 0.37307 | 0.340041 | 0.396498 | 0.335691 | 0.006601 |
| KCTD5 | | 0.062067 | 0.208529 | 0.199748 | 0.26721 | 0.215996 | 0.006614 |
| DCAF11 | | 0.080536 | 0.390231 | 0.369044 | 0.434157 | 0.360174 | 0.006623 |
| TMEM127 | | 0.056044 | 0.400182 | 0.384934 | 0.443272 | 0.366159 | 0.006636 |
| ZNF791 | | 0.058354 | 0.313765 | 0.301327 | 0.363157 | 0.293798 | 0.006664 |
| GABARAP | | 0.116026 | 0.647996 | 0.597923 | 0.627968 | 0.53482 | 0.006683 |
| PEDS1 | | 0.057001 | 0.300484 | 0.288843 | 0.354282 | 0.288357 | 0.006691 |
| MTMR4 | | 0.080581 | 0.306879 | 0.290208 | 0.351163 | 0.289989 | 0.006692 |
| ERICH1 | | 0.090105 | 0.461846 | 0.433883 | 0.46462 | 0.390098 | 0.006737 |
| CAB39 | | 0.080106 | 0.375855 | 0.355554 | 0.425282 | 0.350925 | 0.006742 |
| TCN2 | | 65535 | 0.001404 | 0 | 0.005037 | 0 | 0.006756 |
| ITPRIP | | 0.082986 | 0.228928 | 0.216131 | 0.263852 | 0.213819 | 0.00676 |
| ICAM4 | | 1.051722 | 0.013215 | 0.006375 | 0.023987 | 0.01197 | 0.006792 |
| DNAJC4 | | 0.105623 | 0.503147 | 0.467626 | 0.507796 | 0.428727 | 0.006798 |
| ZXDC | | 0.104691 | 0.324785 | 0.302052 | 0.369153 | 0.31012 | 0.006842 |
| MAP7D1 | | 0.078843 | 0.391685 | 0.370854 | 0.433437 | 0.360174 | 0.006849 |
| RRP12 | | 0.065189 | 0.37882 | 0.362084 | 0.400816 | 0.331882 | 0.00685 |
| PRRC1 | | 0.077729 | 0.211522 | 0.200427 | 0.276085 | 0.223069 | 0.006852 |
| SLC7A6 | | 0.090791 | 0.560064 | 0.525904 | 0.550732 | 0.461915 | 0.006853 |
| BMPR1A | | 1.700522 | 0.008215 | 0.002528 | 0.012953 | 0.004353 | 0.006871 |
| SCIN | | 2.515969 | 0.004986 | 0.000872 | 0.009355 | 0.002176 | 0.006877 |
| RNF157 | | 1.300655 | 0.006343 | 0.002575 | 0.019429 | 0.008705 | 0.006906 |
| TSR3 | | 0.102122 | 0.418047 | 0.389478 | 0.449029 | 0.37432 | 0.006933 |
| PHF2 | | 0.085859 | 0.257517 | 0.242638 | 0.297194 | 0.243743 | 0.006937 |
| PIH1D1 | | 0.096118 | 0.442186 | 0.413686 | 0.466059 | 0.390642 | 0.006947 |
| RHBDD1 | | 0.063126 | 0.261741 | 0.250536 | 0.314944 | 0.257345 | 0.006963 |
| GYPC | | 0.084291 | 0.482859 | 0.455456 | 0.493404 | 0.408596 | 0.006964 |
| PAK2 | | 0.099944 | 0.494129 | 0.461057 | 0.519069 | 0.439608 | 0.006965 |
| CALML6 | | 1.514764 | 0.006455 | 0.002259 | 0.009355 | 0.002176 | 0.006967 |
| ARL14EP | | 0.043984 | 0.199014 | 0.193038 | 0.254018 | 0.202938 | 0.006974 |
| CCL25 | | 1.079366 | 0.005025 | 0.002378 | 0.012953 | 0.004353 | 0.007002 |
| MITD1 | | 0.048095 | 0.330704 | 0.319861 | 0.371792 | 0.305223 | 0.007061 |
| STOX1 | | 1.161474 | 0.011048 | 0.004939 | 0.014632 | 0.005441 | 0.007112 |
| CLASP1 | | 0.024149 | 0.227673 | 0.223894 | 0.290477 | 0.23395 | 0.007135 |
| UBA6 | | 0.091494 | 0.365172 | 0.342732 | 0.418566 | 0.343308 | 0.007138 |
| SF3B1 | | 0.101659 | 0.609113 | 0.567669 | 0.614296 | 0.521219 | 0.007263 |
| RNF220 | | 0.051392 | 0.284947 | 0.274975 | 0.342768 | 0.28074 | 0.007313 |
| QTRT1 | | 0.058568 | 0.229748 | 0.220608 | 0.281842 | 0.229597 | 0.00733 |
| ZNF672 | | 0.077889 | 0.236669 | 0.224231 | 0.275126 | 0.224157 | 0.007338 |
| GTF2A1 | | 0.070475 | 0.319732 | 0.304489 | 0.359799 | 0.293798 | 0.007342 |
| ARL6IP4 | | 0.107565 | 0.518697 | 0.481431 | 0.528184 | 0.446681 | 0.007431 |
| AKAP5 | | 1.120038 | 0.009423 | 0.004336 | 0.011993 | 0.003808 | 0.007433 |
| SUSD3 | | 0.087394 | 0.296296 | 0.27888 | 0.333413 | 0.276931 | 0.007559 |
| PIP5K1A | | 0.108868 | 0.387293 | 0.359143 | 0.431518 | 0.364527 | 0.007573 |
| BMS1 | | 0.057965 | 0.223253 | 0.214461 | 0.283281 | 0.229053 | 0.007573 |
| ZMYM3 | | 0.082834 | 0.239997 | 0.226606 | 0.286639 | 0.23395 | 0.007603 |
| SCAF11 | | 0.099798 | 0.523566 | 0.488572 | 0.537779 | 0.458651 | 0.007625 |
| NFU1 | | 0.067365 | 0.225703 | 0.215406 | 0.276805 | 0.225789 | 0.007634 |
| ZNF592 | | 0.084336 | 0.524161 | 0.494398 | 0.541137 | 0.453754 | 0.007655 |
| KCNA2 | | 1.349624 | 0.005453 | 0.00214 | 0.01847 | 0.008161 | 0.007693 |
| NUP50 | | 0.054461 | 0.250531 | 0.24125 | 0.313744 | 0.254081 | 0.007755 |
| STEEP1 | | 0.078398 | 0.223351 | 0.211537 | 0.279204 | 0.228509 | 0.007757 |
| SMIM35 | | 2.745682 | 0.005546 | 0.000827 | 0.007196 | 0.001088 | 0.007807 |
| COPS7B | | 0.028653 | 0.219531 | 0.215214 | 0.276325 | 0.223069 | 0.007823 |
| USP4 | | 0.087755 | 0.399673 | 0.376087 | 0.44711 | 0.373776 | 0.007826 |
| ERCC1 | | 0.074157 | 0.283246 | 0.269055 | 0.328616 | 0.270947 | 0.007889 |
| HDAC1 | | 0.107923 | 0.683071 | 0.633837 | 0.637563 | 0.553863 | 0.007928 |
| ORAI1 | | 0.104681 | 0.530118 | 0.493016 | 0.511633 | 0.436888 | 0.007977 |
| RAP1A | | 0.104773 | 0.539003 | 0.501246 | 0.541857 | 0.454298 | 0.008024 |
| KDM3A | | 0.072073 | 0.26799 | 0.254931 | 0.309667 | 0.255169 | 0.008092 |
| TUBGCP2 | | 0.080771 | 0.331479 | 0.313431 | 0.386903 | 0.321001 | 0.008097 |
| GPR132 | | 0.103879 | 0.394295 | 0.366902 | 0.379707 | 0.321001 | 0.008102 |
| ARMC9 | | 1.75077 | 0.005208 | 0.001548 | 0.010074 | 0.00272 | 0.008156 |
| SYCP2 | | 1.864719 | 0.009532 | 0.002617 | 0.010074 | 0.00272 | 0.008158 |
| FAM32A | | 0.085047 | 0.493388 | 0.465144 | 0.506117 | 0.424918 | 0.008204 |
| FCER1G | | 1.074377 | 0.006583 | 0.003126 | 0.019909 | 0.009249 | 0.008233 |
| CDC73 | | 0.091565 | 0.494309 | 0.463911 | 0.522427 | 0.437432 | 0.008328 |
| NARS1 | | 0.10234 | 0.390508 | 0.363766 | 0.432478 | 0.35963 | 0.00834 |
| NFKBIE | | 0.088655 | 0.335655 | 0.31565 | 0.350923 | 0.293798 | 0.008357 |
| PEX2 | | 0.054681 | 0.359698 | 0.34632 | 0.397697 | 0.326442 | 0.008401 |
| PPIB | | 0.184716 | 0.571329 | 0.502668 | 0.450708 | 0.398803 | 0.008406 |
| ZC3H15 | | 0.104387 | 0.613587 | 0.570759 | 0.599904 | 0.509249 | 0.008439 |
| RBM22 | | 0.04427 | 0.318947 | 0.309309 | 0.377309 | 0.306855 | 0.00845 |
| NSMCE3 | | 0.094888 | 0.726039 | 0.679823 | 0.621252 | 0.533732 | 0.008473 |
| SLC9A5 | | 3.4236 | 0.003663 | 0.000341 | 0.005997 | 0.000544 | 0.008483 |
| CHD3 | | 0.135206 | 0.477865 | 0.435115 | 0.421444 | 0.366703 | 0.008503 |
| SNX17 | | 0.104931 | 0.820825 | 0.763244 | 0.700168 | 0.606638 | 0.008507 |
| NONO | | 0.085275 | 0.365097 | 0.344142 | 0.424083 | 0.354733 | 0.00851 |
| CLTB | | 0.032558 | 0.218663 | 0.213784 | 0.283281 | 0.229053 | 0.008517 |
| ANAPC4 | | 0.057351 | 0.213 | 0.204699 | 0.26745 | 0.217084 | 0.008531 |
| HAAO | | 1.07165 | 0.019901 | 0.009468 | 0.020628 | 0.009793 | 0.008565 |
| GGNBP2 | | 0.10259 | 0.699726 | 0.651697 | 0.639962 | 0.554407 | 0.008595 |
| TAX1BP3 | | 0.064999 | 0.268891 | 0.257046 | 0.300552 | 0.246464 | 0.008617 |
| TP73 | | 1.050632 | 0.012627 | 0.006096 | 0.026385 | 0.014146 | 0.008795 |
| B4GALT1 | | 0.084684 | 1.345266 | 1.268573 | 0.836412 | 0.776931 | 0.008795 |
| TRIM27 | | 0.085864 | 0.462573 | 0.435846 | 0.478292 | 0.395539 | 0.00881 |
| UBE2G2 | | 0.065291 | 0.358633 | 0.342765 | 0.409691 | 0.338955 | 0.008832 |
| HECTD1 | | 0.074364 | 0.356229 | 0.338332 | 0.411849 | 0.340044 | 0.008899 |
| GAREM1 | | 1.130969 | 0.005478 | 0.002501 | 0.015112 | 0.005985 | 0.008911 |
| GPATCH4 | | 0.01739 | 0.236329 | 0.233498 | 0.280643 | 0.226333 | 0.008921 |
| PIK3CA | | 0.105223 | 0.390874 | 0.36338 | 0.404893 | 0.340588 | 0.008942 |
| H4C3 | | 0.05562 | 0.38162 | 0.367188 | 0.396738 | 0.329162 | 0.009001 |
| TCF25 | | 0.109784 | 0.769174 | 0.712815 | 0.673543 | 0.585419 | 0.00906 |
| SF3A3 | | 0.096667 | 0.239059 | 0.223566 | 0.294315 | 0.243743 | 0.009104 |
| ZMYND8 | | 0.093387 | 0.507791 | 0.475962 | 0.516671 | 0.440696 | 0.009112 |
| XPO6 | | 0.085287 | 0.430551 | 0.405836 | 0.469417 | 0.390642 | 0.009137 |
| DHX8 | | 0.075129 | 0.330307 | 0.313546 | 0.383785 | 0.317737 | 0.009159 |
| UBR5 | | 0.1045 | 0.484363 | 0.450519 | 0.504438 | 0.42111 | 0.009235 |
| C3orf38 | | 0.074616 | 0.354872 | 0.336985 | 0.400336 | 0.334059 | 0.0093 |
| CAMTA1 | | 0.02836 | 0.19553 | 0.191723 | 0.266731 | 0.215452 | 0.0093 |
| NUP153 | | 0.088681 | 0.396134 | 0.372517 | 0.436076 | 0.366159 | 0.009307 |
| PICALM | | 0.102193 | 0.585258 | 0.545236 | 0.582154 | 0.496736 | 0.00934 |
| CUTA | | 0.111538 | 0.625742 | 0.579187 | 0.559127 | 0.495103 | 0.009361 |
| TMEM147 | | 0.100684 | 0.320517 | 0.298911 | 0.349724 | 0.291621 | 0.009395 |
| TBC1D15 | | 0.050976 | 0.336068 | 0.324401 | 0.382586 | 0.316104 | 0.009434 |
| FAM114A1 | | 1.233896 | 0.005155 | 0.002192 | 0.010794 | 0.003264 | 0.009524 |
| RASGRF1 | | 1.919209 | 0.008624 | 0.00228 | 0.012473 | 0.004353 | 0.009572 |
| ZDHHC19 | | 1.317401 | 0.009432 | 0.003784 | 0.010794 | 0.003264 | 0.009584 |
| WDR11 | | 0.081585 | 0.436639 | 0.412632 | 0.461502 | 0.388466 | 0.009586 |
| CYTH2 | | 0.037887 | 0.307839 | 0.29986 | 0.347805 | 0.285092 | 0.009663 |
| PPP2CA | | 0.085662 | 0.322973 | 0.304354 | 0.396018 | 0.32753 | 0.009695 |
| INTS10 | | 0.082486 | 0.320225 | 0.30243 | 0.363157 | 0.303047 | 0.009709 |
| KLF3 | | 0.093901 | 0.760893 | 0.712946 | 0.620053 | 0.539717 | 0.00975 |
| ZNF146 | | 0.077732 | 0.52587 | 0.498286 | 0.527225 | 0.448313 | 0.009778 |
| SLC25A46 | | 0.063009 | 0.280996 | 0.268988 | 0.342528 | 0.281284 | 0.00978 |
| RNF32 | | 1.819828 | 0.005802 | 0.001643 | 0.009834 | 0.00272 | 0.009808 |
| ZNF783 | | 0.106727 | 0.279643 | 0.259702 | 0.301751 | 0.250816 | 0.009817 |
| TBC1D10C | | 0.071891 | 1.232493 | 1.172582 | 0.830415 | 0.779652 | 0.009821 |
| RTF1 | | 0.092837 | 0.424986 | 0.3985 | 0.463181 | 0.389554 | 0.009845 |
| BZW2 | | 0.068295 | 0.305053 | 0.290949 | 0.345167 | 0.287269 | 0.009883 |
| EMC3 | | 0.076017 | 0.292269 | 0.277268 | 0.343488 | 0.28346 | 0.009891 |
| PIM3 | | 0.083038 | 0.445242 | 0.420339 | 0.431518 | 0.366703 | 0.009892 |
| ARMC8 | | 0.042283 | 0.347415 | 0.33738 | 0.398417 | 0.32753 | 0.009916 |
| ARIH2 | | 0.082985 | 0.358859 | 0.3388 | 0.403694 | 0.338955 | 0.009932 |
| CENPC | | 0.059023 | 0.225301 | 0.21627 | 0.279444 | 0.227421 | 0.009943 |
| TMEM165 | | 0.073404 | 0.226173 | 0.214953 | 0.289998 | 0.238847 | 0.009943 |
| **Plasma B cells** | | |  |  |  |  |  |
| SOCS3 | | 3.1554 | 1.188401 | 0.133381 | 0.654762 | 0.124088 | 2.74E-23 |
| KDM5D | | 1.616449 | 1.29156 | 0.421226 | 0.777778 | 0.233577 | 1.56E-17 |
| MT-ND2 | | 0.913549 | 3.410879 | 1.810759 | 0.968254 | 0.693431 | 3.93E-16 |
| POU2F2 | | 1.246304 | 1.077107 | 0.45403 | 0.803571 | 0.430657 | 1.68E-15 |
| EIF1AY | | 1.684215 | 0.957901 | 0.298072 | 0.678571 | 0.240876 | 6.33E-15 |
| STAT3 | | 0.845044 | 1.209833 | 0.673507 | 0.875 | 0.649635 | 1.11E-14 |
| JUNB | | 0.846604 | 1.52738 | 0.849364 | 0.892857 | 0.656934 | 3.7E-12 |
| IGHM | | 1.133034 | 4.754519 | 2.16785 | 0.789683 | 0.437956 | 9.82E-12 |
| DDX3Y | | 1.603679 | 0.641095 | 0.210944 | 0.599206 | 0.20438 | 1.78E-11 |
| BCL3 | | 0.894538 | 1.13948 | 0.612948 | 0.81746 | 0.554745 | 6.06E-11 |
| SERPINB9 | | 1.68538 | 0.548918 | 0.17067 | 0.521825 | 0.167883 | 1.96E-09 |
| SBNO2 | | 0.904183 | 0.978077 | 0.522621 | 0.77381 | 0.510949 | 3.72E-09 |
| ZFP36 | | 0.469794 | 2.058304 | 1.486235 | 0.96627 | 0.868613 | 6.68E-09 |
| ATP5PO | | 1.356317 | 0.55802 | 0.21795 | 0.585317 | 0.226277 | 6.85E-09 |
| PHACTR1 | | 2.100543 | 0.453027 | 0.105633 | 0.422619 | 0.10219 | 1.26E-08 |
| GLDC | | 0.633443 | 1.61653 | 1.042074 | 0.876984 | 0.693431 | 1.26E-08 |
| TMEM184B | | 0.506506 | 1.706374 | 1.20116 | 0.920635 | 0.80292 | 1.26E-08 |
| STK17B | | 0.370452 | 2.054813 | 1.58948 | 0.968254 | 0.861314 | 2.17E-08 |
| SGK1 | | 1.053735 | 1.140228 | 0.54927 | 0.714286 | 0.445255 | 3.25E-08 |
| JAK3 | | 0.966801 | 0.693531 | 0.354838 | 0.672619 | 0.423358 | 9.55E-08 |
| CARD11 | | 0.7296 | 1.221165 | 0.736449 | 0.829365 | 0.547445 | 3.69E-07 |
| CCR10 | | 1.017998 | 0.858151 | 0.423756 | 0.640873 | 0.379562 | 5.6E-07 |
| MVP | | 0.75832 | 0.813655 | 0.48102 | 0.746032 | 0.474453 | 5.6E-07 |
| THEMIS2 | | 0.535868 | 1.448293 | 0.998951 | 0.90873 | 0.751825 | 5.6E-07 |
| CCR9 | | 1.615791 | 0.894326 | 0.291806 | 0.410714 | 0.153285 | 5.19E-06 |
| NFKBIA | | 0.50737 | 1.570182 | 1.104629 | 0.880952 | 0.744526 | 5.21E-06 |
| UBALD2 | | 0.325073 | 1.895912 | 1.513427 | 0.958333 | 0.883212 | 1.8E-05 |
| PIM1 | | 0.439962 | 1.871887 | 1.379869 | 0.926587 | 0.788321 | 3.4E-05 |
| CCL3 | | 2.092405 | 0.382505 | 0.089693 | 0.269841 | 0.051095 | 4.21E-05 |
| FCRL5 | | 0.529132 | 1.388022 | 0.96186 | 0.85119 | 0.664234 | 4.21E-05 |
| PPP1R16B | | 0.931524 | 0.605937 | 0.317695 | 0.571429 | 0.321168 | 4.89E-05 |
| HEG1 | | 2.375817 | 0.23281 | 0.044855 | 0.265873 | 0.058394 | 4.91E-05 |
| LDLR | | 0.57279 | 1.28066 | 0.861007 | 0.827381 | 0.627737 | 5.42E-05 |
| NDUFV3 | | 0.513126 | 1.143973 | 0.801585 | 0.839286 | 0.671533 | 5.42E-05 |
| WARS1 | | 0.321669 | 1.882444 | 1.506225 | 0.934524 | 0.875912 | 5.42E-05 |
| UTY | | 1.584669 | 0.283439 | 0.094499 | 0.343254 | 0.116788 | 7.04E-05 |
| P2RX5 | | 0.402023 | 1.738475 | 1.315672 | 0.922619 | 0.781022 | 7.9E-05 |
| RGS3 | | 1.127554 | 0.447414 | 0.204778 | 0.460317 | 0.20438 | 9.24E-05 |
| EOLA1 | | 0.763276 | 0.584711 | 0.344486 | 0.615079 | 0.379562 | 0.000127 |
| DDIT4 | | 1.605188 | 0.27622 | 0.090792 | 0.329365 | 0.109489 | 0.000151 |
| XRRA1 | | 1.090905 | 0.391273 | 0.183689 | 0.456349 | 0.218978 | 0.00023 |
| SLC3A2 | | 0.45943 | 1.466548 | 1.066581 | 0.880952 | 0.715328 | 0.00023 |
| CRACR2B | | 1.563826 | 0.253265 | 0.085668 | 0.291667 | 0.087591 | 0.000273 |
| SLC37A1 | | 0.557453 | 1.006282 | 0.683769 | 0.78373 | 0.576642 | 0.000382 |
| PARP9 | | 2.128795 | 0.167014 | 0.038188 | 0.222222 | 0.043796 | 0.000408 |
| CHST11 | | 0.468906 | 1.129395 | 0.816002 | 0.853175 | 0.686131 | 0.000511 |
| SELENOI | | 0.608477 | 0.786359 | 0.515764 | 0.700397 | 0.489051 | 0.000525 |
| NRIP1 | | 2.219785 | 0.170995 | 0.036708 | 0.22619 | 0.051095 | 0.000639 |
| TLR7 | | 0.797745 | 0.634626 | 0.365067 | 0.573413 | 0.328467 | 0.00074 |
| SLC2A5 | | 0.74753 | 0.789903 | 0.470484 | 0.625 | 0.430657 | 0.000925 |
| DUSP5 | | 0.397756 | 1.691346 | 1.283796 | 0.882937 | 0.759124 | 0.000929 |
| MAP3K8 | | 0.845199 | 0.503282 | 0.280144 | 0.537698 | 0.306569 | 0.000968 |
| SERPINB1 | | 1.366485 | 0.277237 | 0.107522 | 0.333333 | 0.138686 | 0.001085 |
| CD69 | | 0.798483 | 0.93146 | 0.535546 | 0.603175 | 0.40146 | 0.001221 |
| APOL6 | | 0.873094 | 0.483984 | 0.264243 | 0.505952 | 0.291971 | 0.001383 |
| MAN2A1 | | 0.290732 | 1.552243 | 1.268938 | 0.926587 | 0.832117 | 0.001541 |
| USP48 | | 0.309119 | 1.525754 | 1.231489 | 0.928571 | 0.824818 | 0.001623 |
| NANS | | 0.349165 | 1.455516 | 1.142636 | 0.882937 | 0.766423 | 0.001742 |
| SYNE2 | | 0.416207 | 1.278696 | 0.958245 | 0.853175 | 0.708029 | 0.001789 |
| ETV6 | | 0.557761 | 0.795766 | 0.540608 | 0.722222 | 0.540146 | 0.001873 |
| ASB7 | | 1.020116 | 0.337439 | 0.166383 | 0.406746 | 0.218978 | 0.002555 |
| ZFAND2A | | 0.596575 | 0.699819 | 0.462806 | 0.674603 | 0.481752 | 0.002555 |
| ZFP36L1 | | 1.310383 | 0.299411 | 0.120727 | 0.331349 | 0.145985 | 0.002653 |
| LILRB4 | | 0.565257 | 0.859852 | 0.581118 | 0.704365 | 0.532847 | 0.002675 |
| LGMN | | 0.797666 | 0.631668 | 0.363385 | 0.541667 | 0.364964 | 0.003382 |
| ZNF587B | | 0.809847 | 0.458813 | 0.261726 | 0.501984 | 0.313869 | 0.00361 |
| MAPK6 | | 0.483069 | 0.948446 | 0.67857 | 0.78373 | 0.605839 | 0.004033 |
| TNFRSF1B | | 0.849109 | 0.53809 | 0.298708 | 0.513889 | 0.335766 | 0.004188 |
| TARS3 | | 1.222125 | 0.28868 | 0.123743 | 0.349206 | 0.175182 | 0.004423 |
| NAPSA | | 1.82608 | 0.185318 | 0.052265 | 0.200397 | 0.051095 | 0.004769 |
| NAMPT | | 1.050564 | 0.310383 | 0.149847 | 0.376984 | 0.19708 | 0.004769 |
| ENGASE | | 0.963532 | 0.371255 | 0.19038 | 0.424603 | 0.233577 | 0.004769 |
| CLTC | | 0.692304 | 0.504169 | 0.312013 | 0.55754 | 0.394161 | 0.004769 |
| CTU2 | | 0.686812 | 0.541883 | 0.336631 | 0.537698 | 0.321168 | 0.004769 |
| YIPF6 | | 0.643545 | 0.652386 | 0.417617 | 0.626984 | 0.423358 | 0.004915 |
| ELK4 | | 0.651995 | 0.589058 | 0.374876 | 0.638889 | 0.445255 | 0.004982 |
| HDAC9 | | 0.524189 | 0.790097 | 0.549394 | 0.688492 | 0.525547 | 0.005107 |
| SEMA4D | | 0.5241 | 0.622044 | 0.432565 | 0.656746 | 0.459854 | 0.005107 |
| RB1 | | 0.374411 | 1.146705 | 0.884591 | 0.871032 | 0.693431 | 0.005376 |
| ARID5B | | 0.658032 | 0.643809 | 0.408009 | 0.599206 | 0.416058 | 0.005411 |
| CYTH4 | | 0.512199 | 0.923467 | 0.647492 | 0.755952 | 0.525547 | 0.005411 |
| KIAA0586 | | 1.254992 | 0.238989 | 0.100136 | 0.319444 | 0.145985 | 0.005476 |
| PLEKHH3 | | 1.084683 | 0.32279 | 0.152194 | 0.339286 | 0.167883 | 0.005878 |
| LZTFL1 | | 0.463727 | 1.07524 | 0.779668 | 0.763889 | 0.569343 | 0.005878 |
| NOP58 | | 0.342265 | 1.24854 | 0.98485 | 0.861111 | 0.715328 | 0.005878 |
| ACAP1 | | 0.342081 | 1.124562 | 0.887169 | 0.859127 | 0.729927 | 0.005878 |
| IL10RA | | 0.846445 | 0.357401 | 0.19877 | 0.400794 | 0.218978 | 0.006106 |
| DGLUCY | | 0.720298 | 0.520361 | 0.315845 | 0.555556 | 0.357664 | 0.006106 |
| ANKRD28 | | 0.401884 | 1.075516 | 0.814025 | 0.809524 | 0.70073 | 0.006106 |
| CREB3L2 | | 0.311073 | 1.368413 | 1.102999 | 0.89881 | 0.766423 | 0.006106 |
| DTX3L | | 1.183533 | 0.296043 | 0.130339 | 0.335317 | 0.160584 | 0.00704 |
| CDK14 | | 0.676863 | 0.518601 | 0.324397 | 0.535714 | 0.335766 | 0.00704 |
| SDAD1 | | 0.885836 | 0.355531 | 0.192404 | 0.428571 | 0.255474 | 0.007064 |
| CPEB4 | | 0.411771 | 1.220809 | 0.917682 | 0.815476 | 0.686131 | 0.00754 |
| REL | | 1.026842 | 0.330937 | 0.162418 | 0.373016 | 0.20438 | 0.007753 |
| MAP3K11 | | 0.679842 | 0.480127 | 0.299711 | 0.549603 | 0.357664 | 0.007945 |
| SPTY2D1 | | 0.322242 | 1.219934 | 0.975735 | 0.871032 | 0.759124 | 0.008103 |
| RGS16 | | 1.080629 | 0.369347 | 0.174636 | 0.353175 | 0.175182 | 0.008172 |
| SLC7A5 | | 0.389102 | 1.524477 | 1.164098 | 0.845238 | 0.715328 | 0.008281 |
| TMED9 | | 0.232026 | 1.793042 | 1.526664 | 0.948413 | 0.861314 | 0.008281 |
| SAMSN1 | | 0.696744 | 0.525547 | 0.324243 | 0.525794 | 0.350365 | 0.0084 |
| SPNS1 | | 0.507837 | 0.659701 | 0.463952 | 0.662698 | 0.467153 | 0.0084 |
| FCER2 | | 1.128918 | 0.357178 | 0.163323 | 0.375 | 0.218978 | 0.008756 |
| ATP2B1 | | 0.552015 | 0.680671 | 0.464263 | 0.656746 | 0.467153 | 0.008763 |
| MGST2 | | 0.939531 | 0.280019 | 0.146002 | 0.34127 | 0.167883 | 0.008894 |
| RNASET2 | | 0.75851 | 0.415219 | 0.245439 | 0.460317 | 0.262774 | 0.008996 |
| TNRC6A | | 0.51836 | 0.668866 | 0.466979 | 0.690476 | 0.467153 | 0.009132 |

^1^log2FC - log fold-change of the average expression between the two groups (FF compared to HMF infants).

^2^FF – formula-fed.

^3^HMF - human milk fed.

^4^pct_FF, percent of cells with detected expression of the gene in FF infants.

^5^pct_HMF, percent of cells with detected expression the gene in HMF infants.

^6^p_val_adj, adjusted p-value based on Benjamini and Hochberg method using all genes in the dataset.

**Supplemental Table 6.** The list of differentially expressed genes in T cell subsets of FF (n=3) and HMF (n=6) infants.

| **Gene** | **log2FC^1^** | **avg_FF^2^** | **avg_HMF^3^** | **pct_FF^4^** | **pct_HMF^5^** | **p_val_adj^6^** | |
| --- | --- | --- | --- | --- | --- | --- | --- |
| **CD4+ T cells** | |  |  |  |  |  |  |
| SOCS3 | 1.523003 | 1.342717 | 0.467213 | 0.733874 | 0.413218 | 0 |  |
| MT-ND2 | 1.263635 | 1.475368 | 0.614481 | 0.873817 | 0.38871 | 0 |  |
| SBNO2 | 1.158326 | 0.600947 | 0.269244 | 0.571938 | 0.303736 | 3.7E-220 |  |
| GPRIN3 | 1.097478 | 0.625741 | 0.292429 | 0.581152 | 0.320812 | 2.5E-211 |  |
| ZFP36 | 0.771419 | 1.080832 | 0.633196 | 0.755867 | 0.56127 | 1.5E-205 |  |
| DDIT4 | 0.983592 | 0.834662 | 0.422104 | 0.628947 | 0.383688 | 1.9E-200 |  |
| MYC | 0.700894 | 1.186973 | 0.730215 | 0.787197 | 0.570912 | 6.4E-198 |  |
| DDX3Y | 1.535678 | 0.34965 | 0.1206 | 0.369579 | 0.135597 | 6.1E-183 |  |
| NFKBIZ | 0.774451 | 0.875699 | 0.511944 | 0.696892 | 0.485135 | 2.3E-177 |  |
| IRF1 | 0.57172 | 1.229883 | 0.827483 | 0.816562 | 0.644034 | 3.7E-174 |  |
| CD69 | 0.390964 | 1.847286 | 1.408777 | 0.91166 | 0.824227 | 5.6E-168 |  |
| ARID5B | 0.841287 | 0.727172 | 0.405868 | 0.635582 | 0.405986 | 1.2E-167 |  |
| PIM2 | 0.421041 | 1.400243 | 1.045822 | 0.882664 | 0.757533 | 1.6E-149 |  |
| BATF | 1.885289 | 0.230738 | 0.062458 | 0.262809 | 0.07734 | 1.4E-148 |  |
| BTG2 | 0.385225 | 1.524115 | 1.166953 | 0.897776 | 0.799518 | 5E-143 |  |
| TP53INP1 | 1.205321 | 0.40878 | 0.177277 | 0.415653 | 0.205504 | 2.5E-141 |  |
| UBALD2 | 0.560162 | 0.857661 | 0.581688 | 0.744072 | 0.570711 | 2.1E-124 |  |
| SOCS1 | 1.133094 | 0.386669 | 0.176297 | 0.403735 | 0.207513 | 2.3E-124 |  |
| PLAC8 | 0.349245 | 1.370163 | 1.075571 | 0.875046 | 0.795299 | 3.7E-116 |  |
| KDM5D | 1.456486 | 0.221601 | 0.080747 | 0.265635 | 0.098433 | 3.3E-115 |  |
| CCR7 | 0.275802 | 1.597976 | 1.319914 | 0.923701 | 0.857573 | 9.3E-114 |  |
| LDLR | 1.364959 | 0.237227 | 0.092102 | 0.267478 | 0.105665 | 4.7E-105 |  |
| BCL3 | 1.441055 | 0.209313 | 0.077089 | 0.248433 | 0.096826 | 4.3E-100 |  |
| EIF1AY | 1.659532 | 0.175149 | 0.055442 | 0.210591 | 0.070711 | 6.9E-100 |  |
| HDGFL3 | 1.68009 | 0.183557 | 0.057281 | 0.204202 | 0.065086 | 1.3E-99 |  |
| RASGRF2 | 0.940601 | 0.332614 | 0.173297 | 0.378302 | 0.193049 | 2E-99 |  |
| ATP10A | 0.779956 | 0.445994 | 0.25974 | 0.484458 | 0.296706 | 9.67E-98 |  |
| CFL2 | 1.540832 | 0.189996 | 0.065299 | 0.224475 | 0.081358 | 2.36E-96 |  |
| NAMPT | 0.998751 | 0.249376 | 0.124796 | 0.334808 | 0.156689 | 4.14E-96 |  |
| UTY | 1.34731 | 0.210108 | 0.082578 | 0.249785 | 0.104661 | 1.37E-91 |  |
| FAM107B | 0.299328 | 1.241902 | 1.009208 | 0.88721 | 0.790679 | 3.09E-91 |  |
| THEMIS2 | 0.852761 | 0.332056 | 0.183867 | 0.392554 | 0.211531 | 9.17E-91 |  |
| ID2 | 0.653589 | 0.634694 | 0.403473 | 0.567392 | 0.386099 | 1.66E-90 |  |
| MAF | 1.277696 | 0.354506 | 0.146217 | 0.260474 | 0.114303 | 2.83E-88 |  |
| FOS | 0.289601 | 2.05396 | 1.680403 | 0.933038 | 0.842306 | 3.6E-88 |  |
| SLC2A3 | 0.59615 | 0.692725 | 0.458249 | 0.61064 | 0.44536 | 2.68E-87 |  |
| UGCG | 0.740518 | 0.469747 | 0.281155 | 0.488144 | 0.313178 | 9.37E-85 |  |
| PIM1 | 0.392243 | 1.16953 | 0.891116 | 0.800098 | 0.659904 | 2.58E-83 |  |
| RAPGEF6 | 0.417234 | 0.880746 | 0.659555 | 0.766187 | 0.621937 | 3.26E-83 |  |
| USP53 | 1.12227 | 0.223771 | 0.102794 | 0.269812 | 0.122539 | 7.69E-83 |  |
| SFMBT2 | 0.628547 | 0.555071 | 0.359035 | 0.552033 | 0.381077 | 1.89E-80 |  |
| ARID5A | 0.678388 | 0.451214 | 0.281947 | 0.489986 | 0.32282 | 2.95E-76 |  |
| JMJD1C | 0.404348 | 0.870354 | 0.65762 | 0.755744 | 0.621736 | 1.11E-75 |  |
| NFKBIA | 0.228485 | 1.765379 | 1.506806 | 0.927755 | 0.865207 | 9.74E-75 |  |
| JAK3 | 0.307367 | 1.116527 | 0.902283 | 0.858214 | 0.746485 | 1.06E-74 |  |
| C20orf204 | 2.489787 | 0.094352 | 0.016798 | 0.11193 | 0.023503 | 3.37E-73 |  |
| MTHFD2 | 1.218273 | 0.165695 | 0.071215 | 0.215506 | 0.089594 | 4.94E-73 |  |
| ANKRD55 | 0.805443 | 0.327851 | 0.187592 | 0.363312 | 0.206107 | 8.48E-73 |  |
| PIK3IP1 | 0.26389 | 1.279672 | 1.065762 | 0.90994 | 0.812575 | 4.21E-71 |  |
| TENT5C | 1.223032 | 0.239249 | 0.10249 | 0.238727 | 0.112696 | 4.87E-71 |  |
| KLHL15 | 0.939729 | 0.270801 | 0.141177 | 0.313921 | 0.17276 | 1.61E-70 |  |
| ATP5PO | 1.355816 | 0.115245 | 0.045028 | 0.166728 | 0.061671 | 1.02E-65 |  |
| SMAP2 | 0.277829 | 1.137507 | 0.938252 | 0.861899 | 0.769586 | 2.45E-65 |  |
| RNASET2 | 0.547141 | 0.469816 | 0.32153 | 0.537658 | 0.366613 | 3.58E-65 |  |
| NOP58 | 0.571236 | 0.463988 | 0.312283 | 0.516894 | 0.358176 | 2.78E-64 |  |
| TAF4B | 0.961681 | 0.21978 | 0.112848 | 0.271655 | 0.140619 | 9.31E-64 |  |
| USP9Y | 1.496063 | 0.123334 | 0.043724 | 0.159356 | 0.059663 | 4.93E-63 |  |
| DUSP2 | 0.771582 | 0.356982 | 0.209111 | 0.367613 | 0.225191 | 1.8E-62 |  |
| PRDM1 | 1.979402 | 0.107626 | 0.027293 | 0.119794 | 0.033949 | 1.81E-62 |  |
| FMN1 | 1.234021 | 0.14547 | 0.061844 | 0.173731 | 0.0681 | 1.86E-62 |  |
| YWHAB | 0.579261 | 0.394249 | 0.263873 | 0.482369 | 0.319004 | 3.47E-62 |  |
| SERPINB9 | 0.440125 | 0.699513 | 0.515591 | 0.647131 | 0.488951 | 5.84E-61 |  |
| TNFSF8 | 1.112817 | 0.152089 | 0.070325 | 0.20027 | 0.089393 | 2.11E-59 |  |
| NTPCR | 1.027502 | 0.162461 | 0.079697 | 0.217594 | 0.103656 | 2.92E-58 |  |
| ZNRF1 | 0.820746 | 0.241423 | 0.136682 | 0.286399 | 0.156489 | 1.05E-57 |  |
| DDX21 | 0.470998 | 0.565076 | 0.407683 | 0.585698 | 0.442748 | 1.49E-57 |  |
| MATK | 1.008659 | 0.167403 | 0.0832 | 0.209608 | 0.096826 | 1.66E-57 |  |
| CLEC2B | 0.594924 | 0.457126 | 0.302653 | 0.477823 | 0.336481 | 3.87E-57 |  |
| SGK1 | 0.775933 | 0.340685 | 0.198964 | 0.338985 | 0.206107 | 4.5E-57 |  |
| KLRG1 | 0.659982 | 0.330467 | 0.209148 | 0.343531 | 0.203094 | 6.14E-56 |  |
| PCED1B | 0.533628 | 0.491905 | 0.339816 | 0.502027 | 0.351547 | 6.14E-56 |  |
| FURIN | 0.718156 | 0.279102 | 0.169659 | 0.339845 | 0.207714 | 1.29E-53 |  |
| STAT3 | 0.442728 | 0.559608 | 0.411727 | 0.593685 | 0.44094 | 1.97E-53 |  |
| GBP2 | 0.496625 | 0.562623 | 0.398766 | 0.542204 | 0.399357 | 3.56E-53 |  |
| TIMP1 | 0.728657 | 0.264292 | 0.159491 | 0.302248 | 0.175573 | 3.94E-52 |  |
| NPDC1 | 1.199489 | 0.165718 | 0.072159 | 0.174223 | 0.078144 | 5.91E-52 |  |
| CALHM6 | 1.098331 | 0.135204 | 0.063148 | 0.168325 | 0.072519 | 1.01E-50 |  |
| MAD1L1 | 0.460368 | 0.555252 | 0.403557 | 0.570217 | 0.433909 | 1.34E-50 |  |
| FOSL2 | 1.054031 | 0.133937 | 0.064507 | 0.179629 | 0.081961 | 2.49E-50 |  |
| DTX1 | 0.995631 | 0.205018 | 0.10282 | 0.220789 | 0.115508 | 3.63E-50 |  |
| KDSR | 0.629476 | 0.30799 | 0.199088 | 0.36233 | 0.227802 | 4.57E-50 |  |
| NOTCH1 | 0.475754 | 0.497676 | 0.357874 | 0.532989 | 0.390317 | 1.17E-49 |  |
| BCL2L11 | 0.596521 | 0.353062 | 0.233496 | 0.382971 | 0.245279 | 1.26E-49 |  |
| CSTF2T | 0.593444 | 0.317448 | 0.210391 | 0.381988 | 0.245078 | 6.45E-49 |  |
| NR4A2 | 0.519833 | 0.47226 | 0.329379 | 0.454356 | 0.314986 | 2.44E-48 |  |
| CNP | 0.666343 | 0.274428 | 0.172918 | 0.334685 | 0.207111 | 5.03E-48 |  |
| ICOS | 0.366673 | 0.828746 | 0.642749 | 0.690503 | 0.573524 | 6.18E-48 |  |
| PIM3 | 0.623438 | 0.334999 | 0.217455 | 0.378793 | 0.251908 | 1.43E-47 |  |
| CSRNP1 | 0.43087 | 0.635369 | 0.471326 | 0.587787 | 0.456408 | 2.68E-47 |  |
| RGS1 | 0.28618 | 1.469333 | 1.204956 | 0.812139 | 0.719365 | 3.74E-47 |  |
| SYNE2 | 0.359024 | 0.781033 | 0.608965 | 0.684236 | 0.543994 | 5.8E-47 |  |
| CMTM8 | 0.866889 | 0.181757 | 0.099663 | 0.230004 | 0.124749 | 2.7E-46 |  |
| GPR132 | 0.605331 | 0.369021 | 0.242565 | 0.391449 | 0.264765 | 4.09E-46 |  |
| MT2A | 0.98172 | 0.14526 | 0.073556 | 0.193144 | 0.096424 | 6.1E-46 |  |
| LINS1 | 0.403553 | 0.57041 | 0.431226 | 0.590736 | 0.443351 | 1.46E-45 |  |
| GPR171 | 0.630729 | 0.331755 | 0.214264 | 0.370193 | 0.244877 | 1.73E-45 |  |
| IFI16 | 0.415415 | 0.560965 | 0.420614 | 0.581644 | 0.44094 | 2.11E-45 |  |
| YBX3 | 0.713026 | 0.248313 | 0.151481 | 0.290208 | 0.176778 | 6.3E-45 |  |
| SLA | 0.508799 | 0.382299 | 0.268682 | 0.433591 | 0.299518 | 1.73E-44 |  |
| SRGN | 0.28073 | 1.060315 | 0.872825 | 0.780931 | 0.671957 | 4.65E-44 |  |
| PDK1 | 0.409609 | 0.603487 | 0.454321 | 0.588279 | 0.452391 | 1.16E-43 |  |
| GPR183 | 0.338761 | 0.861745 | 0.681399 | 0.683991 | 0.549819 | 5.02E-43 |  |
| XBP1 | 0.56379 | 0.331076 | 0.22398 | 0.37523 | 0.251507 | 1.73E-42 |  |
| MAP3K5 | 1.181727 | 0.100535 | 0.044318 | 0.140926 | 0.06147 | 2.19E-42 |  |
| STOM | 1.453034 | 0.079338 | 0.028978 | 0.104558 | 0.036963 | 8.61E-42 |  |
| SERPINB1 | 0.419697 | 0.482924 | 0.361025 | 0.522054 | 0.383086 | 1.32E-40 |  |
| DENND4A | 0.375399 | 0.564547 | 0.435205 | 0.592456 | 0.459421 | 1.76E-39 |  |
| B4GALT1 | 0.350423 | 0.640193 | 0.502138 | 0.627104 | 0.492567 | 5.08E-39 |  |
| MAN2A2 | 0.561936 | 0.245108 | 0.166034 | 0.315641 | 0.200884 | 2.01E-38 |  |
| ADGRE5 | 0.217858 | 1.282977 | 1.103156 | 0.864725 | 0.780233 | 2.1E-38 |  |
| SSH2 | 0.465794 | 0.38306 | 0.277363 | 0.452513 | 0.323825 | 3.85E-38 |  |
| PGAP6 | 0.389079 | 0.515156 | 0.393382 | 0.54503 | 0.407593 | 9.65E-38 |  |
| CH25H | 2.980717 | 0.043891 | 0.00556 | 0.049638 | 0.007031 | 1.29E-37 |  |
| S100A4 | 0.43752 | 0.514395 | 0.379831 | 0.419585 | 0.293491 | 5.1E-37 |  |
| ZNF460 | 0.669586 | 0.187028 | 0.117582 | 0.249539 | 0.148051 | 6.17E-37 |  |
| P2RX5 | 0.908238 | 0.122256 | 0.065142 | 0.164394 | 0.082965 | 6.89E-37 |  |
| GPCPD1 | 0.313016 | 0.671048 | 0.540165 | 0.667772 | 0.540579 | 9.52E-37 |  |
| GIMAP4 | 0.254599 | 1.045748 | 0.876567 | 0.783143 | 0.689233 | 1.24E-36 |  |
| RNF44 | 0.257574 | 0.912099 | 0.762964 | 0.793955 | 0.674769 | 1.91E-36 |  |
| TAGLN2 | 0.399875 | 0.471072 | 0.357037 | 0.514928 | 0.389715 | 3.99E-36 |  |
| STX11 | 1.138023 | 0.072463 | 0.032926 | 0.107138 | 0.042387 | 7.16E-36 |  |
| CEBPB | 0.745181 | 0.134078 | 0.07999 | 0.186755 | 0.099638 | 1.67E-35 |  |
| FBXL16 | 0.800421 | 0.17173 | 0.098604 | 0.204079 | 0.116312 | 3.1E-35 |  |
| CD55 | 0.279646 | 0.813941 | 0.670519 | 0.727854 | 0.61591 | 1.49E-34 |  |
| GBP5 | 1.498741 | 0.074547 | 0.026379 | 0.085268 | 0.030133 | 8.42E-34 |  |
| BCL6 | 1.284965 | 0.060038 | 0.024638 | 0.086989 | 0.031338 | 5.87E-33 |  |
| S100B | 1.764342 | 0.091735 | 0.027003 | 0.072859 | 0.022901 | 6.25E-33 |  |
| SETBP1 | 2.260856 | 0.036721 | 0.007662 | 0.050129 | 0.009642 | 1.19E-32 |  |
| IDI1 | 0.604502 | 0.203236 | 0.133668 | 0.268215 | 0.169948 | 1.91E-32 |  |
| PRDX2 | 0.315872 | 0.6273 | 0.503951 | 0.62735 | 0.504018 | 2.03E-32 |  |
| ST6GALNAC1 | 0.974301 | 0.096012 | 0.048869 | 0.134292 | 0.065287 | 3.73E-32 |  |
| HNRNPF | 0.261679 | 0.812754 | 0.677931 | 0.741123 | 0.625552 | 3.97E-32 |  |
| SAT1 | 0.376945 | 0.429142 | 0.330468 | 0.482492 | 0.351145 | 5.39E-32 |  |
| FAS | 1.026052 | 0.092214 | 0.045282 | 0.127534 | 0.061069 | 6.75E-32 |  |
| RUNX3 | 0.451028 | 0.320224 | 0.234251 | 0.387517 | 0.274207 | 9.48E-32 |  |
| PPP1R15B | 0.245212 | 0.86222 | 0.727448 | 0.769013 | 0.66814 | 1.02E-31 |  |
| PELI1 | 0.491868 | 0.316148 | 0.224814 | 0.371667 | 0.262957 | 1.56E-31 |  |
| LMNB1 | 0.520728 | 0.245298 | 0.170978 | 0.307532 | 0.204098 | 2.6E-31 |  |
| ZNF141 | 0.554374 | 0.217833 | 0.148334 | 0.276201 | 0.177581 | 3.11E-31 |  |
| R3HDM4 | 0.192827 | 1.099816 | 0.962217 | 0.856248 | 0.77963 | 4.51E-31 |  |
| NKG7 | 0.899968 | 0.088818 | 0.047597 | 0.124585 | 0.058055 | 7.65E-31 |  |
| MYO1F | 0.810603 | 0.136557 | 0.077857 | 0.157882 | 0.084572 | 1.39E-30 |  |
| LPIN2 | 0.412 | 0.373998 | 0.28109 | 0.433714 | 0.315589 | 1.5E-30 |  |
| PMAIP1 | 0.747308 | 0.166622 | 0.099259 | 0.190195 | 0.110687 | 2.16E-30 |  |
| FLOT1 | 0.374166 | 0.430681 | 0.332293 | 0.475243 | 0.354761 | 3.07E-30 |  |
| SLC7A8 | 2.383441 | 0.029956 | 0.005741 | 0.043249 | 0.007232 | 3.2E-30 |  |
| CCNL1 | 0.319498 | 0.570001 | 0.456769 | 0.607814 | 0.484934 | 3.66E-30 |  |
| PRSS21 | 2.63733 | 0.030175 | 0.00485 | 0.041528 | 0.006428 | 3.66E-30 |  |
| SLAMF6 | 0.477982 | 0.328654 | 0.235967 | 0.360855 | 0.255524 | 4.02E-30 |  |
| TMEM140 | 0.61352 | 0.180317 | 0.117855 | 0.221649 | 0.134793 | 4.26E-30 |  |
| ARL4C | 0.180628 | 1.292676 | 1.140552 | 0.888684 | 0.81599 | 6.05E-30 |  |
| HS3ST3B1 | 0.574012 | 0.22642 | 0.152097 | 0.276324 | 0.181197 | 6.58E-30 |  |
| PSMB9 | 0.329192 | 0.547471 | 0.435777 | 0.569235 | 0.449377 | 7.84E-30 |  |
| FAM177A1 | 0.547613 | 0.200529 | 0.137192 | 0.266372 | 0.171354 | 1.39E-29 |  |
| APBA2 | 0.414348 | 0.382578 | 0.28707 | 0.429045 | 0.319807 | 1.72E-29 |  |
| NCK2 | 0.283706 | 0.702416 | 0.577019 | 0.684359 | 0.57272 | 2.05E-29 |  |
| ZC3H12D | 0.624786 | 0.236841 | 0.153595 | 0.252734 | 0.165729 | 2.06E-29 |  |
| NANS | 0.705376 | 0.149556 | 0.09172 | 0.19511 | 0.115307 | 2.82E-29 |  |
| IL2RB | 0.55467 | 0.218407 | 0.148695 | 0.257157 | 0.166131 | 4.26E-29 |  |
| PREX1 | 0.540906 | 0.232712 | 0.159952 | 0.272638 | 0.180394 | 5.19E-29 |  |
| SLC7A5 | 0.797759 | 0.103299 | 0.059422 | 0.141664 | 0.073122 | 5.95E-29 |  |
| ENGASE | 0.583214 | 0.199213 | 0.13297 | 0.254822 | 0.165327 | 1.19E-28 |  |
| DCXR | 0.464939 | 0.270758 | 0.196165 | 0.334316 | 0.231217 | 1.36E-28 |  |
| CENPM | 0.842842 | 0.11507 | 0.064156 | 0.142278 | 0.075131 | 1.39E-28 |  |
| SARDH | 0.47922 | 0.263939 | 0.189341 | 0.315272 | 0.215348 | 2.5E-28 |  |
| RNF157 | 0.547824 | 0.281641 | 0.192657 | 0.297088 | 0.205705 | 4.14E-28 |  |
| IL4R | 0.556493 | 0.215761 | 0.146707 | 0.275464 | 0.183407 | 4.86E-28 |  |
| PDCL | 0.514645 | 0.243467 | 0.170419 | 0.297702 | 0.203495 | 5.58E-28 |  |
| FKBP5 | 0.309754 | 0.608053 | 0.490565 | 0.602777 | 0.491161 | 7.2E-28 |  |
| NSMCE3 | 0.283035 | 0.735116 | 0.604162 | 0.665561 | 0.559863 | 7.6E-28 |  |
| TBC1D15 | 0.3271 | 0.498954 | 0.397735 | 0.551542 | 0.430695 | 7.98E-28 |  |
| ZNF101 | 0.37558 | 0.382057 | 0.294488 | 0.422042 | 0.309763 | 1.67E-27 |  |
| IRF4 | 0.777995 | 0.150601 | 0.087827 | 0.165745 | 0.095018 | 2.29E-27 |  |
| ACAP1 | 0.181874 | 1.132488 | 0.998353 | 0.867551 | 0.783849 | 2.3E-27 |  |
| EOLA1 | 0.668638 | 0.141109 | 0.088772 | 0.19511 | 0.11812 | 4E-27 |  |
| SAMD10 | 0.670821 | 0.147716 | 0.092787 | 0.191424 | 0.115106 | 5.57E-27 |  |
| CPSF1 | 0.573295 | 0.159689 | 0.107324 | 0.220666 | 0.137204 | 6.22E-27 |  |
| PIEZO1 | 0.49695 | 0.219001 | 0.155185 | 0.281361 | 0.189032 | 6.97E-27 |  |
| FRMD4B | 1.333857 | 0.047574 | 0.018873 | 0.069542 | 0.024709 | 7.68E-27 |  |
| KLF6 | 0.154726 | 1.738481 | 1.561682 | 0.927018 | 0.8636 | 8.37E-27 |  |
| NARF | 0.407685 | 0.287414 | 0.216662 | 0.362575 | 0.253515 | 1.32E-26 |  |
| TRAF1 | 0.490624 | 0.226496 | 0.161201 | 0.287996 | 0.195058 | 1.65E-26 |  |
| ARPC1B | 0.310103 | 0.550446 | 0.443981 | 0.568866 | 0.453797 | 2.01E-26 |  |
| LRRC37A2 | 2.229802 | 0.029267 | 0.006239 | 0.04374 | 0.009642 | 2.81E-26 |  |
| IFI27L2 | 0.790041 | 0.102362 | 0.059199 | 0.136503 | 0.072519 | 2.92E-26 |  |
| ATP8A1 | 0.370567 | 0.354346 | 0.274079 | 0.421796 | 0.308759 | 4.1E-26 |  |
| SMDT1 | 0.284583 | 0.611368 | 0.501921 | 0.626367 | 0.508437 | 4.57E-26 |  |
| FAM184A | 0.868728 | 0.093668 | 0.051295 | 0.119548 | 0.060064 | 6.64E-26 |  |
| B3GNT2 | 0.463312 | 0.272314 | 0.197515 | 0.333825 | 0.238851 | 1.43E-25 |  |
| GNB5 | 0.401665 | 0.287624 | 0.217727 | 0.357661 | 0.252712 | 1.47E-25 |  |
| SAYSD1 | 0.608725 | 0.147184 | 0.09652 | 0.193513 | 0.116914 | 1.53E-25 |  |
| NFIL3 | 1.269859 | 0.044728 | 0.018549 | 0.067576 | 0.024307 | 1.62E-25 |  |
| DUSP5 | 0.914754 | 0.085883 | 0.045555 | 0.120039 | 0.061671 | 1.68E-25 |  |
| ZNF331 | 0.468572 | 0.303003 | 0.218974 | 0.330262 | 0.236842 | 1.77E-25 |  |
| HELB | 0.280986 | 0.634313 | 0.522058 | 0.641479 | 0.524508 | 2.58E-25 |  |
| LPIN1 | 0.437765 | 0.246747 | 0.182168 | 0.323627 | 0.226396 | 2.78E-25 |  |
| EZR | 0.258366 | 0.741559 | 0.619969 | 0.686202 | 0.584773 | 3.05E-25 |  |
| KLRK1 | 0.823917 | 0.097789 | 0.055242 | 0.107384 | 0.051828 | 4.3E-25 |  |
| DEF6 | 0.170487 | 1.111584 | 0.98769 | 0.849736 | 0.77963 | 5.81E-25 |  |
| CTLA4 | 0.953478 | 0.090626 | 0.046798 | 0.106647 | 0.05223 | 7.09E-25 |  |
| KLF9 | 0.321705 | 0.520499 | 0.416464 | 0.522423 | 0.414423 | 7.09E-25 |  |
| GIMAP6 | 0.310541 | 0.533918 | 0.430519 | 0.539378 | 0.428887 | 8.43E-25 |  |
| GADD45B | 0.525683 | 0.241051 | 0.167441 | 0.279887 | 0.194054 | 1.25E-24 |  |
| NOP16 | 0.420817 | 0.291443 | 0.217708 | 0.32719 | 0.23182 | 1.76E-24 |  |
| FOSB | 0.219293 | 1.080061 | 0.927758 | 0.775525 | 0.669546 | 1.96E-24 |  |
| H3-3B | 0.447668 | 0.244354 | 0.179167 | 0.322275 | 0.227802 | 2.15E-24 |  |
| GOLGA8B | 0.498208 | 0.188708 | 0.133603 | 0.254331 | 0.170149 | 3.94E-24 |  |
| TRIB2 | 0.238679 | 0.833677 | 0.706559 | 0.707335 | 0.613499 | 6.22E-24 |  |
| MAN1A1 | 0.713506 | 0.0857 | 0.052263 | 0.123602 | 0.064685 | 6.96E-24 |  |
| PARP9 | 0.482826 | 0.19714 | 0.141068 | 0.253471 | 0.169345 | 8.49E-24 |  |
| LTBP3 | 0.246181 | 0.758064 | 0.639143 | 0.678707 | 0.58176 | 1.02E-23 |  |
| FAM43A | 1.604424 | 0.035007 | 0.011513 | 0.04976 | 0.014665 | 1.46E-23 |  |
| JAG2 | 2.051474 | 0.027077 | 0.006532 | 0.039563 | 0.008839 | 2.04E-23 |  |
| ALG13 | 0.280633 | 0.52109 | 0.428977 | 0.568866 | 0.447971 | 2.32E-23 |  |
| CR1 | 0.804137 | 0.105885 | 0.060641 | 0.127411 | 0.069506 | 2.55E-23 |  |
| CLASRP | 0.365917 | 0.286371 | 0.222217 | 0.36405 | 0.260145 | 2.67E-23 |  |
| UBASH3B | 0.404274 | 0.285954 | 0.216071 | 0.34648 | 0.251105 | 3.47E-23 |  |
| TBXA2R | 0.783853 | 0.109321 | 0.063495 | 0.127165 | 0.069305 | 3.97E-23 |  |
| NME1 | 0.520936 | 0.172016 | 0.119881 | 0.228161 | 0.149458 | 4.11E-23 |  |
| DEAF1 | 0.317601 | 0.423491 | 0.33981 | 0.482123 | 0.373644 | 6.07E-23 |  |
| NABP1 | 0.780096 | 0.085686 | 0.049897 | 0.120162 | 0.064082 | 6.75E-23 |  |
| HAPLN3 | 0.372035 | 0.335414 | 0.259172 | 0.377196 | 0.279831 | 7.76E-23 |  |
| TRIM22 | 0.283536 | 0.58031 | 0.476768 | 0.581275 | 0.477099 | 7.92E-23 |  |
| LRIG1 | 0.464454 | 0.207202 | 0.150168 | 0.257157 | 0.174568 | 1.34E-22 |  |
| THAP2 | 0.759797 | 0.101235 | 0.059787 | 0.132572 | 0.074126 | 1.52E-22 |  |
| ZFY | 1.364807 | 0.048924 | 0.018996 | 0.069419 | 0.028526 | 2.14E-22 |  |
| KLHL36 | 0.329401 | 0.413911 | 0.329418 | 0.47254 | 0.367216 | 2.35E-22 |  |
| GIMAP5 | 0.243039 | 0.689025 | 0.582201 | 0.672441 | 0.571314 | 2.74E-22 |  |
| TRIB1 | 1.680974 | 0.027835 | 0.008681 | 0.043986 | 0.012053 | 3.46E-22 |  |
| ADTRP | 0.396146 | 0.344886 | 0.262074 | 0.345128 | 0.253515 | 3.58E-22 |  |
| REL | 0.333639 | 0.345408 | 0.274092 | 0.416022 | 0.310366 | 3.92E-22 |  |
| EIF4E3 | 0.259733 | 0.611796 | 0.510998 | 0.593439 | 0.480514 | 4.79E-22 |  |
| TSPAN14 | 0.183381 | 1.023529 | 0.901357 | 0.841258 | 0.748694 | 7.74E-22 |  |
| HIF1A | 0.534392 | 0.157854 | 0.10899 | 0.215383 | 0.140217 | 8.65E-22 |  |
| SRA1 | 0.42392 | 0.195284 | 0.145564 | 0.257525 | 0.173765 | 9.05E-22 |  |
| SESN1 | 0.438139 | 0.246539 | 0.181967 | 0.290453 | 0.204701 | 1.08E-21 |  |
| DTNBP1 | 0.529164 | 0.155432 | 0.107707 | 0.207151 | 0.134391 | 1.16E-21 |  |
| CBLB | 0.315766 | 0.4323 | 0.347321 | 0.469222 | 0.365207 | 1.31E-21 |  |
| INAFM2 | 1.25809 | 0.044756 | 0.018712 | 0.062907 | 0.024307 | 1.37E-21 |  |
| TRAF4 | 0.454727 | 0.228237 | 0.166533 | 0.276938 | 0.194857 | 1.42E-21 |  |
| TIPARP | 0.381245 | 0.354235 | 0.271973 | 0.37437 | 0.283447 | 1.57E-21 |  |
| MCM3 | 0.486333 | 0.182373 | 0.130185 | 0.242044 | 0.163921 | 2.13E-21 |  |
| NXT1 | 0.358757 | 0.319785 | 0.24938 | 0.378179 | 0.283246 | 2.19E-21 |  |
| HSPD1 | 0.383967 | 0.247301 | 0.189513 | 0.32891 | 0.235637 | 2.35E-21 |  |
| LGALS9 | 0.536266 | 0.157742 | 0.108771 | 0.211328 | 0.139012 | 2.46E-21 |  |
| APOBEC3C | 0.597037 | 0.13205 | 0.0873 | 0.176066 | 0.110687 | 2.74E-21 |  |
| MYD88 | 0.352636 | 0.295406 | 0.231348 | 0.367858 | 0.271796 | 4.27E-21 |  |
| FBXO32 | 0.449818 | 0.224942 | 0.164688 | 0.267846 | 0.187826 | 5.44E-21 |  |
| HAUS3 | 0.359297 | 0.342296 | 0.266835 | 0.402138 | 0.308759 | 5.76E-21 |  |
| CHST11 | 0.345036 | 0.353674 | 0.278443 | 0.403121 | 0.306951 | 6.33E-21 |  |
| AFF3 | 0.497392 | 0.163094 | 0.115534 | 0.206414 | 0.133789 | 6.5E-21 |  |
| ZNF394 | 0.269836 | 0.527075 | 0.437164 | 0.569112 | 0.458417 | 1.13E-20 |  |
| CLIC1 | 0.524706 | 0.139675 | 0.097088 | 0.197567 | 0.128164 | 1.42E-20 |  |
| CCL5 | 1.661091 | 0.035331 | 0.011172 | 0.040546 | 0.011049 | 1.44E-20 |  |
| TXK | 0.345204 | 0.342799 | 0.26985 | 0.406438 | 0.310165 | 1.69E-20 |  |
| ZNF595 | 0.779776 | 0.081324 | 0.047368 | 0.10677 | 0.056649 | 2.52E-20 |  |
| STK17A | 0.200832 | 0.859448 | 0.747762 | 0.751321 | 0.656489 | 3.11E-20 |  |
| KLRD1 | 2.006674 | 0.02688 | 0.006689 | 0.036245 | 0.008839 | 3.5E-20 |  |
| ENO1 | 0.274786 | 0.519834 | 0.429681 | 0.572552 | 0.465247 | 4.1E-20 |  |
| CPT1A | 0.628344 | 0.09315 | 0.060261 | 0.135397 | 0.078947 | 4.19E-20 |  |
| CREM | 0.402455 | 0.246791 | 0.186715 | 0.297948 | 0.214142 | 4.64E-20 |  |
| GLRX | 0.313936 | 0.392169 | 0.315478 | 0.442806 | 0.345319 | 5.49E-20 |  |
| ATP2B1 | 0.320178 | 0.366471 | 0.293533 | 0.429045 | 0.327642 | 5.5E-20 |  |
| FUT8 | 0.363813 | 0.27557 | 0.214147 | 0.341811 | 0.25231 | 5.8E-20 |  |
| DNASE1L1 | 0.666739 | 0.09908 | 0.062413 | 0.134415 | 0.078546 | 5.88E-20 |  |
| HIPK1 | 0.217945 | 0.76831 | 0.660585 | 0.711267 | 0.617718 | 5.9E-20 |  |
| REXO2 | 0.258948 | 0.608745 | 0.508727 | 0.579064 | 0.474086 | 9.64E-20 |  |
| MGAT4A | 0.157165 | 1.076852 | 0.965705 | 0.839169 | 0.773403 | 9.83E-20 |  |
| STING1 | 0.251675 | 0.607683 | 0.510405 | 0.606094 | 0.506027 | 1.03E-19 |  |
| FKBP11 | 0.505307 | 0.165106 | 0.116319 | 0.211942 | 0.142025 | 1.05E-19 |  |
| SREBF2 | 0.32904 | 0.309141 | 0.246096 | 0.367244 | 0.273403 | 1.3E-19 |  |
| PDE3B | 0.266454 | 0.568709 | 0.472802 | 0.550436 | 0.445962 | 1.37E-19 |  |
| RNF149 | 0.236553 | 0.632884 | 0.537174 | 0.643937 | 0.535356 | 1.45E-19 |  |
| MAP2K3 | 0.418784 | 0.200533 | 0.15001 | 0.268829 | 0.189233 | 1.75E-19 |  |
| MLKL | 0.540328 | 0.12967 | 0.089163 | 0.175083 | 0.111691 | 2.1E-19 |  |
| CDK5RAP3 | 0.263293 | 0.501458 | 0.417807 | 0.546382 | 0.43632 | 2.22E-19 |  |
| RALGDS | 0.353271 | 0.261564 | 0.204754 | 0.334193 | 0.244877 | 2.27E-19 |  |
| STAT1 | 0.222045 | 0.638196 | 0.547157 | 0.589262 | 0.482925 | 2.38E-19 |  |
| DTX3L | 0.319043 | 0.338045 | 0.270977 | 0.399435 | 0.304138 | 2.72E-19 |  |
| PTTG1IP | 0.317554 | 0.324958 | 0.260756 | 0.399066 | 0.301326 | 3.38E-19 |  |
| CRTC3 | 0.230049 | 0.643726 | 0.548845 | 0.658558 | 0.551025 | 4.68E-19 |  |
| ZAP70 | 0.170376 | 0.991771 | 0.881298 | 0.827252 | 0.738851 | 4.68E-19 |  |
| PTPRE | 0.458065 | 0.132602 | 0.096529 | 0.187492 | 0.120932 | 4.84E-19 |  |
| PRPF6 | 0.265089 | 0.522419 | 0.434729 | 0.557439 | 0.455806 | 7.56E-19 |  |
| PCSK5 | 0.400195 | 0.266206 | 0.201719 | 0.30188 | 0.222981 | 9.4E-19 |  |
| LPAR2 | 0.406168 | 0.219585 | 0.165704 | 0.282099 | 0.202692 | 9.41E-19 |  |
| HSP90AA1 | 0.236282 | 0.584312 | 0.49604 | 0.625998 | 0.52511 | 9.52E-19 |  |
| XRRA1 | 0.661438 | 0.096979 | 0.061315 | 0.132203 | 0.078746 | 1.41E-18 |  |
| LSM7 | 0.279289 | 0.393307 | 0.324084 | 0.473645 | 0.370229 | 1.7E-18 |  |
| ITPK1 | 0.439137 | 0.177392 | 0.13084 | 0.228161 | 0.157091 | 1.86E-18 |  |
| SLC16A1 | 0.535778 | 0.125489 | 0.08656 | 0.167588 | 0.106468 | 1.93E-18 |  |
| CTSA | 0.320775 | 0.28161 | 0.225469 | 0.338739 | 0.249096 | 2.16E-18 |  |
| CD83 | 0.538942 | 0.097029 | 0.066782 | 0.140312 | 0.08417 | 2.7E-18 |  |
| IKZF3 | 0.31182 | 0.412641 | 0.332434 | 0.438752 | 0.351145 | 3.16E-18 |  |
| ZNRF2 | 0.350984 | 0.24682 | 0.193519 | 0.313552 | 0.22961 | 4.24E-18 |  |
| MRFAP1 | 0.525266 | 0.109631 | 0.076175 | 0.161691 | 0.101446 | 4.4E-18 |  |
| ABCA7 | 0.356666 | 0.253322 | 0.197836 | 0.317607 | 0.233829 | 5.14E-18 |  |
| PDE4B | 0.329503 | 0.401258 | 0.319325 | 0.416144 | 0.328244 | 5.42E-18 |  |
| SMAD3 | 0.303939 | 0.384137 | 0.311165 | 0.413319 | 0.323624 | 1.24E-17 |  |
| CFLAR | 0.178674 | 0.893707 | 0.789604 | 0.778351 | 0.68863 | 1.5E-17 |  |
| GUK1 | 0.308359 | 0.309018 | 0.249551 | 0.388131 | 0.296505 | 1.73E-17 |  |
| RGS16 | 1.348075 | 0.050193 | 0.019717 | 0.056518 | 0.023704 | 1.86E-17 |  |
| NGRN | 0.435466 | 0.179151 | 0.132474 | 0.243396 | 0.171957 | 2.02E-17 |  |
| ARHGEF3 | 0.24967 | 0.504988 | 0.42474 | 0.546259 | 0.447368 | 2.68E-17 |  |
| VDAC1 | 0.444541 | 0.143949 | 0.105776 | 0.21096 | 0.144034 | 2.96E-17 |  |
| TIFA | 0.588813 | 0.099175 | 0.065941 | 0.136503 | 0.083568 | 3.26E-17 |  |
| DARS1 | 0.286214 | 0.35365 | 0.290011 | 0.425851 | 0.330655 | 3.29E-17 |  |
| WHAMM | 0.306462 | 0.294008 | 0.237741 | 0.363067 | 0.274809 | 3.45E-17 |  |
| SH3YL1 | 0.326897 | 0.293657 | 0.234118 | 0.344145 | 0.260145 | 3.95E-17 |  |
| KCNA3 | 0.29597 | 0.332843 | 0.271109 | 0.397838 | 0.308558 | 4.11E-17 |  |
| OGT | 0.14374 | 1.127243 | 1.020346 | 0.87394 | 0.800924 | 5.33E-17 |  |
| SACM1L | 0.285244 | 0.335114 | 0.274996 | 0.405701 | 0.311973 | 5.57E-17 |  |
| S100PBP | 0.273074 | 0.404151 | 0.334457 | 0.454356 | 0.357573 | 5.81E-17 |  |
| CYCS | 0.423218 | 0.170385 | 0.127066 | 0.236761 | 0.166734 | 5.91E-17 |  |
| GPR15 | 2.064754 | 0.029436 | 0.007036 | 0.028996 | 0.006629 | 5.99E-17 |  |
| ADD2 | 1.231818 | 0.038224 | 0.016275 | 0.050866 | 0.020088 | 6.31E-17 |  |
| MCM6 | 0.45825 | 0.147693 | 0.107501 | 0.198304 | 0.134793 | 7.3E-17 |  |
| CEMIP2 | 0.288664 | 0.394758 | 0.323173 | 0.438752 | 0.346525 | 7.84E-17 |  |
| PPIF | 0.539536 | 0.1147 | 0.078913 | 0.158619 | 0.10225 | 7.9E-17 |  |
| EFHD2 | 0.278247 | 0.314882 | 0.25965 | 0.390466 | 0.296304 | 9.92E-17 |  |
| SREBF1 | 0.262914 | 0.421709 | 0.351454 | 0.459024 | 0.360787 | 1.01E-16 |  |
| SDCBP | 0.314712 | 0.285225 | 0.229324 | 0.366261 | 0.279831 | 1.09E-16 |  |
| PRKCB | 0.209311 | 0.619126 | 0.535513 | 0.619978 | 0.521896 | 1.21E-16 |  |
| GMIP | 0.258659 | 0.427745 | 0.357537 | 0.491461 | 0.392929 | 1.38E-16 |  |
| ATF6 | 0.322897 | 0.267638 | 0.213966 | 0.334439 | 0.251507 | 1.65E-16 |  |
| RUNDC3B | 6.037254 | 0.011186 | 0.00017 | 0.01585 | 0.000402 | 1.83E-16 |  |
| WDR75 | 0.405094 | 0.161786 | 0.122179 | 0.222878 | 0.15448 | 1.86E-16 |  |
| FGFR1 | 1.003834 | 0.043736 | 0.02181 | 0.06303 | 0.028927 | 2.07E-16 |  |
| PHF11 | 0.279134 | 0.319268 | 0.263104 | 0.392554 | 0.301326 | 2.15E-16 |  |
| CD2 | 0.13167 | 1.194708 | 1.090499 | 0.872343 | 0.8182 | 2.31E-16 |  |
| RBCK1 | 0.361955 | 0.229748 | 0.178769 | 0.294999 | 0.219968 | 2.42E-16 |  |
| JUN | 0.136983 | 1.738411 | 1.580944 | 0.868043 | 0.814986 | 2.48E-16 |  |
| MAT2A | 0.271786 | 0.421066 | 0.348766 | 0.473768 | 0.385898 | 2.81E-16 |  |
| ELMO2 | 0.35333 | 0.218027 | 0.170666 | 0.287259 | 0.212133 | 3.13E-16 |  |
| TTC39B | 0.334297 | 0.282506 | 0.224075 | 0.346111 | 0.266975 | 3.16E-16 |  |
| TNIP2 | 0.300509 | 0.299391 | 0.243096 | 0.361224 | 0.275412 | 3.59E-16 |  |
| USP36 | 0.286853 | 0.33695 | 0.276194 | 0.415776 | 0.325834 | 3.59E-16 |  |
| BICDL1 | 0.241303 | 0.486901 | 0.411909 | 0.52832 | 0.430695 | 4.52E-16 |  |
| IKBKB | 0.212482 | 0.634542 | 0.547643 | 0.62563 | 0.52732 | 5.2E-16 |  |
| ANKDD1A | 1.381982 | 0.024967 | 0.00958 | 0.03858 | 0.012857 | 5.29E-16 |  |
| FAN1 | 0.46702 | 0.15992 | 0.115695 | 0.198919 | 0.137605 | 6.05E-16 |  |
| YARS1 | 0.390826 | 0.183397 | 0.139876 | 0.251628 | 0.182001 | 6.58E-16 |  |
| SLC12A9 | 0.331774 | 0.24062 | 0.191186 | 0.301511 | 0.224387 | 6.93E-16 |  |
| NOB1 | 0.321235 | 0.256677 | 0.20544 | 0.326944 | 0.247087 | 8.02E-16 |  |
| TINF2 | 0.274332 | 0.324147 | 0.268015 | 0.396609 | 0.306147 | 9.6E-16 |  |
| IL21R | 0.242773 | 0.518107 | 0.437863 | 0.530286 | 0.436119 | 1.02E-15 |  |
| GTPBP1 | 0.315117 | 0.251887 | 0.202463 | 0.326207 | 0.246083 | 1.03E-15 |  |
| KBTBD2 | 0.233915 | 0.577099 | 0.490721 | 0.599214 | 0.502009 | 1.04E-15 |  |
| POLR3E | 0.33243 | 0.236162 | 0.187559 | 0.30532 | 0.227601 | 1.18E-15 |  |
| ZNF267 | 0.438838 | 0.12428 | 0.091685 | 0.175943 | 0.117316 | 1.36E-15 |  |
| GNB2 | 0.273904 | 0.377946 | 0.312591 | 0.442806 | 0.354761 | 1.4E-15 |  |
| RELT | 0.475123 | 0.106105 | 0.076332 | 0.151001 | 0.097429 | 1.63E-15 |  |
| PNPLA8 | 0.387287 | 0.150492 | 0.115061 | 0.210222 | 0.145038 | 1.68E-15 |  |
| OXLD1 | 0.288774 | 0.26803 | 0.219408 | 0.334071 | 0.251708 | 1.83E-15 |  |
| XRN2 | 0.25059 | 0.433321 | 0.364229 | 0.494655 | 0.40217 | 2.11E-15 |  |
| TPM4 | 0.344639 | 0.208043 | 0.163835 | 0.277798 | 0.203696 | 2.18E-15 |  |
| NELL2 | 0.280914 | 0.513792 | 0.422886 | 0.410984 | 0.331057 | 2.31E-15 |  |
| MVP | 0.255705 | 0.398067 | 0.333412 | 0.477209 | 0.38188 | 2.41E-15 |  |
| ZBTB10 | 0.250703 | 0.508261 | 0.427187 | 0.529672 | 0.436722 | 2.66E-15 |  |
| ELOVL5 | 0.257328 | 0.423585 | 0.354387 | 0.474505 | 0.38188 | 3.36E-15 |  |
| MGAT2 | 0.364417 | 0.213239 | 0.16564 | 0.270918 | 0.200884 | 3.45E-15 |  |
| APOL6 | 0.22448 | 0.540656 | 0.46275 | 0.570095 | 0.467658 | 3.47E-15 |  |
| FLNB | 0.293242 | 0.291052 | 0.237518 | 0.347709 | 0.264162 | 3.59E-15 |  |
| LPCAT1 | 0.320029 | 0.211884 | 0.169731 | 0.274849 | 0.201286 | 3.91E-15 |  |
| KLHL2 | 0.399088 | 0.152949 | 0.115987 | 0.207274 | 0.144235 | 3.99E-15 |  |
| RAB5C | 0.214955 | 0.587355 | 0.50605 | 0.613589 | 0.511852 | 3.99E-15 |  |
| ABCA1 | 0.53087 | 0.128262 | 0.088775 | 0.150756 | 0.098835 | 4.03E-15 |  |
| TP53INP2 | 0.535733 | 0.10653 | 0.073485 | 0.149036 | 0.097429 | 4.24E-15 |  |
| VPS28 | 0.259241 | 0.357104 | 0.29837 | 0.4288 | 0.340096 | 5.54E-15 |  |
| RAB27A | 0.32022 | 0.224095 | 0.179488 | 0.276201 | 0.203696 | 6.72E-15 |  |
| WDR74 | 0.36684 | 0.188445 | 0.146135 | 0.247573 | 0.179992 | 7.06E-15 |  |
| IRF7 | 0.271274 | 0.340885 | 0.282453 | 0.385182 | 0.301326 | 7.16E-15 |  |
| E2F3 | 0.397344 | 0.137175 | 0.104151 | 0.183069 | 0.124347 | 8.4E-15 |  |
| ARID4B | 0.211114 | 0.595564 | 0.51449 | 0.633002 | 0.530333 | 8.74E-15 |  |
| REC8 | 0.400552 | 0.1443 | 0.109317 | 0.191915 | 0.132382 | 9.31E-15 |  |
| KLF16 | 0.305811 | 0.286876 | 0.232079 | 0.348077 | 0.269184 | 1.04E-14 |  |
| RORA | 0.259188 | 0.438776 | 0.366623 | 0.457304 | 0.37284 | 1.04E-14 |  |
| SH3GLB2 | 0.312326 | 0.243203 | 0.195862 | 0.3079 | 0.232624 | 1.17E-14 |  |
| PALM2AKAP2 | 0.354923 | 0.227242 | 0.177683 | 0.254454 | 0.187425 | 1.18E-14 |  |
| CCNL2 | 0.23395 | 0.459524 | 0.390735 | 0.523529 | 0.425472 | 1.18E-14 |  |
| PTGER4 | 0.174493 | 0.952539 | 0.844025 | 0.739771 | 0.652069 | 1.21E-14 |  |
| IRF2BPL | 0.298917 | 0.307941 | 0.250314 | 0.364418 | 0.28405 | 1.24E-14 |  |
| SRSF11 | 0.343621 | 0.203378 | 0.160275 | 0.273989 | 0.203294 | 1.45E-14 |  |
| GPR155 | 0.358604 | 0.184553 | 0.143937 | 0.237253 | 0.171555 | 1.49E-14 |  |
| UPB1 | 2.114087 | 0.018159 | 0.004195 | 0.024327 | 0.005424 | 1.6E-14 |  |
| ZNF689 | 0.451598 | 0.119868 | 0.087651 | 0.160462 | 0.107071 | 1.79E-14 |  |
| CANT1 | 0.300863 | 0.208042 | 0.168882 | 0.281361 | 0.207111 | 1.8E-14 |  |
| CCDC167 | 0.327839 | 0.214924 | 0.171236 | 0.273252 | 0.204299 | 1.83E-14 |  |
| MED16 | 0.277811 | 0.276038 | 0.227687 | 0.342548 | 0.259944 | 1.83E-14 |  |
| BST2 | 0.344488 | 0.169576 | 0.133556 | 0.217103 | 0.154279 | 2.03E-14 |  |
| NOCT | 0.492908 | 0.105975 | 0.075305 | 0.144981 | 0.094616 | 2.28E-14 |  |
| KAT6A | 0.200092 | 0.65927 | 0.573891 | 0.679936 | 0.582764 | 2.38E-14 |  |
| PPP2R1B | 0.298726 | 0.219441 | 0.178399 | 0.279641 | 0.207111 | 2.47E-14 |  |
| SFXN1 | 0.255471 | 0.370465 | 0.310344 | 0.432608 | 0.344516 | 2.76E-14 |  |
| ST6GALNAC3 | 1.158644 | 0.029707 | 0.013307 | 0.042757 | 0.016874 | 2.79E-14 |  |
| PTPRJ | 0.388817 | 0.163141 | 0.1246 | 0.21096 | 0.150462 | 2.79E-14 |  |
| CHMP1B | 0.178304 | 0.786507 | 0.695069 | 0.710038 | 0.617517 | 3.15E-14 |  |
| ZNF831 | 0.430671 | 0.124335 | 0.092246 | 0.174223 | 0.119124 | 3.28E-14 |  |
| RELB | 0.38267 | 0.156303 | 0.119887 | 0.199287 | 0.139614 | 3.45E-14 |  |
| KRAS | 0.268218 | 0.353846 | 0.293814 | 0.424008 | 0.338891 | 3.69E-14 |  |
| HEATR3 | 0.36697 | 0.162428 | 0.125948 | 0.217349 | 0.154881 | 5.63E-14 |  |
| PTPN1 | 0.221535 | 0.510755 | 0.43805 | 0.561986 | 0.467859 | 5.75E-14 |  |
| TCIRG1 | 0.265867 | 0.280206 | 0.233047 | 0.358275 | 0.27722 | 6.73E-14 |  |
| CDK17 | 0.328215 | 0.201397 | 0.160417 | 0.273129 | 0.203897 | 7.17E-14 |  |
| ARL16 | 0.438093 | 0.121831 | 0.089924 | 0.167465 | 0.113901 | 7.32E-14 |  |
| BAZ1A | 0.286709 | 0.261087 | 0.214031 | 0.330507 | 0.253917 | 7.32E-14 |  |
| RRP7A | 0.392323 | 0.148406 | 0.113071 | 0.203096 | 0.143833 | 7.99E-14 |  |
| SRSF2 | 0.207143 | 0.539898 | 0.467687 | 0.589507 | 0.497188 | 8.06E-14 |  |
| ZNF217 | 0.219236 | 0.512704 | 0.440423 | 0.552771 | 0.459221 | 8.82E-14 |  |
| SEPHS2 | 0.33746 | 0.212531 | 0.168204 | 0.278167 | 0.209924 | 9.02E-14 |  |
| SPTAN1 | 0.166857 | 0.841921 | 0.749967 | 0.750461 | 0.668341 | 9.02E-14 |  |
| CUBN | 0.436074 | 0.148803 | 0.109987 | 0.193021 | 0.136802 | 9.22E-14 |  |
| NFKBIB | 0.424069 | 0.110393 | 0.082278 | 0.159602 | 0.107473 | 1.11E-13 |  |
| NAP1L4 | 0.221061 | 0.536776 | 0.460519 | 0.58447 | 0.493371 | 1.18E-13 |  |
| USP24 | 0.237028 | 0.337653 | 0.286495 | 0.417496 | 0.32965 | 1.33E-13 |  |
| NR1H2 | 0.267141 | 0.301061 | 0.250171 | 0.372896 | 0.29329 | 1.44E-13 |  |
| MFSD14A | 0.248749 | 0.342688 | 0.288415 | 0.423394 | 0.33869 | 1.49E-13 |  |
| CYBC1 | 0.23781 | 0.393836 | 0.333986 | 0.453127 | 0.362194 | 1.54E-13 |  |
| PPTC7 | 0.242685 | 0.35786 | 0.302453 | 0.433591 | 0.343913 | 1.61E-13 |  |
| TNFRSF13C | 1.300796 | 0.025349 | 0.010289 | 0.038088 | 0.014464 | 1.73E-13 |  |
| GADD45A | 0.465435 | 0.166571 | 0.12064 | 0.19683 | 0.141623 | 1.89E-13 |  |
| PPP1R11 | 0.254766 | 0.282004 | 0.236354 | 0.359504 | 0.277421 | 2.16E-13 |  |
| XRCC6 | 0.329587 | 0.179026 | 0.142462 | 0.250768 | 0.185617 | 2.27E-13 |  |
| BACH1 | 0.281405 | 0.248454 | 0.204425 | 0.316255 | 0.242266 | 2.29E-13 |  |
| ATP5F1B | 0.285838 | 0.242349 | 0.19879 | 0.325593 | 0.2499 | 2.46E-13 |  |
| CORO7 | 0.231082 | 0.47695 | 0.406359 | 0.506082 | 0.421053 | 2.63E-13 |  |
| CD99 | 0.197425 | 0.639443 | 0.557662 | 0.616415 | 0.532945 | 2.99E-13 |  |
| NDUFV2 | 0.212519 | 0.508814 | 0.439121 | 0.557808 | 0.462836 | 3.28E-13 |  |
| TATDN2 | 0.210654 | 0.483542 | 0.417851 | 0.551419 | 0.451989 | 3.47E-13 |  |
| HEATR1 | 0.30277 | 0.225573 | 0.182871 | 0.294016 | 0.223182 | 3.51E-13 |  |
| OGDH | 0.258577 | 0.354832 | 0.296608 | 0.408035 | 0.325231 | 3.51E-13 |  |
| NAB1 | 0.313688 | 0.198663 | 0.159841 | 0.257034 | 0.191643 | 3.64E-13 |  |
| CERS2 | 0.240613 | 0.367983 | 0.311456 | 0.440226 | 0.356167 | 3.88E-13 |  |
| B3GALT6 | 0.431014 | 0.116799 | 0.086635 | 0.158865 | 0.108276 | 4.92E-13 |  |
| HIVEP2 | 0.197239 | 0.560715 | 0.489066 | 0.579678 | 0.491161 | 5.03E-13 |  |
| GRK5 | 0.2941 | 0.240617 | 0.196243 | 0.305934 | 0.234632 | 5.32E-13 |  |
| TBC1D4 | 0.253594 | 0.302831 | 0.254016 | 0.381619 | 0.298714 | 5.32E-13 |  |
| PPRC1 | 0.31549 | 0.193669 | 0.155628 | 0.251874 | 0.187224 | 5.37E-13 |  |
| SCAND1 | 0.279771 | 0.265444 | 0.218652 | 0.340828 | 0.264363 | 6.39E-13 |  |
| SSBP4 | 0.427581 | 0.11026 | 0.081979 | 0.163165 | 0.112093 | 6.44E-13 |  |
| GNL3 | 0.221544 | 0.465874 | 0.399556 | 0.516157 | 0.424669 | 6.44E-13 |  |
| TFRC | 0.308408 | 0.220864 | 0.178355 | 0.286767 | 0.218763 | 6.87E-13 |  |
| TNKS | 0.291442 | 0.215034 | 0.175701 | 0.285293 | 0.215548 | 7.55E-13 |  |
| INPP5A | 0.309946 | 0.202012 | 0.162957 | 0.26244 | 0.196464 | 8.12E-13 |  |
| IGFBP4 | 1.317704 | 0.027121 | 0.01088 | 0.038948 | 0.015669 | 8.5E-13 |  |
| OXR1 | 0.294456 | 0.238356 | 0.194351 | 0.301266 | 0.230615 | 8.54E-13 |  |
| EDA | 2.121834 | 0.022084 | 0.005074 | 0.023467 | 0.006027 | 1.03E-12 |  |
| PCGF3 | 0.347473 | 0.153756 | 0.120846 | 0.206905 | 0.149458 | 1.03E-12 |  |
| HERPUD1 | 0.310929 | 0.197583 | 0.159276 | 0.259246 | 0.193652 | 1.07E-12 |  |
| IRAK1 | 0.234346 | 0.306374 | 0.260439 | 0.375845 | 0.294496 | 1.09E-12 |  |
| GCH1 | 0.416715 | 0.115734 | 0.0867 | 0.164025 | 0.113299 | 1.1E-12 |  |
| CD81 | 0.312607 | 0.153178 | 0.123337 | 0.220543 | 0.159703 | 1.12E-12 |  |
| STX10 | 0.288637 | 0.240385 | 0.196797 | 0.304951 | 0.234231 | 1.13E-12 |  |
| TMEM259 | 0.216611 | 0.432436 | 0.372148 | 0.504116 | 0.410808 | 1.19E-12 |  |
| SMG1 | 0.381731 | 0.159113 | 0.122122 | 0.219192 | 0.16131 | 1.25E-12 |  |
| BTBD1 | 0.253711 | 0.272773 | 0.228784 | 0.345497 | 0.268984 | 1.27E-12 |  |
| ARAF | 0.243306 | 0.32985 | 0.278659 | 0.40607 | 0.325231 | 1.27E-12 |  |
| TM9SF3 | 0.186316 | 0.630682 | 0.554273 | 0.655609 | 0.56127 | 1.52E-12 |  |
| GZMK | 1.389787 | 0.041903 | 0.015991 | 0.038211 | 0.015468 | 1.79E-12 |  |
| SNAPC1 | 0.431077 | 0.125074 | 0.092768 | 0.169677 | 0.118923 | 1.79E-12 |  |
| BBC3 | 0.364972 | 0.175113 | 0.135972 | 0.211328 | 0.15448 | 2.1E-12 |  |
| ELP1 | 0.352177 | 0.159399 | 0.124873 | 0.216611 | 0.157895 | 2.19E-12 |  |
| USP32 | 0.267375 | 0.24319 | 0.202049 | 0.305934 | 0.234632 | 2.19E-12 |  |
| COL5A3 | 2.883584 | 0.019179 | 0.002599 | 0.01757 | 0.003013 | 2.45E-12 |  |
| TRAF7 | 0.27956 | 0.22644 | 0.186551 | 0.293525 | 0.225191 | 2.59E-12 |  |
| CERS6 | 0.227537 | 0.380427 | 0.324919 | 0.434574 | 0.352551 | 2.69E-12 |  |
| TRIP12 | 0.189322 | 0.601952 | 0.527923 | 0.63079 | 0.535757 | 2.71E-12 |  |
| TULP4 | 0.265051 | 0.225389 | 0.187562 | 0.287013 | 0.217356 | 2.75E-12 |  |
| DYNC2I1 | 0.323465 | 0.162572 | 0.129919 | 0.216366 | 0.157895 | 2.99E-12 |  |
| NCDN | 0.341238 | 0.161085 | 0.127154 | 0.214154 | 0.156689 | 3.77E-12 |  |
| IL18R1 | 1.045738 | 0.026178 | 0.01268 | 0.041528 | 0.017879 | 3.87E-12 |  |
| EEFSEC | 0.296077 | 0.20471 | 0.166729 | 0.265266 | 0.201085 | 4.32E-12 |  |
| PAXX | 0.252364 | 0.267761 | 0.22479 | 0.33321 | 0.258538 | 4.4E-12 |  |
| MBP | 0.230848 | 0.336443 | 0.286695 | 0.399926 | 0.319807 | 4.45E-12 |  |
| RRAS2 | 0.371723 | 0.15187 | 0.117374 | 0.20113 | 0.146645 | 4.71E-12 |  |
| DNAJC2 | 0.294343 | 0.198024 | 0.161478 | 0.264897 | 0.200482 | 5.08E-12 |  |
| TMEM238 | 0.315054 | 0.180529 | 0.145113 | 0.240201 | 0.180394 | 6.24E-12 |  |
| MIF | 0.158924 | 0.753583 | 0.674979 | 0.732277 | 0.644034 | 6.39E-12 |  |
| N4BP2 | 0.214981 | 0.413391 | 0.35616 | 0.470205 | 0.383688 | 6.45E-12 |  |
| GBP4 | 0.285501 | 0.213142 | 0.174874 | 0.260474 | 0.197268 | 6.75E-12 |  |
| AK5 | 0.330663 | 0.226883 | 0.180411 | 0.260106 | 0.199478 | 6.91E-12 |  |
| VSIR | 0.272658 | 0.275556 | 0.228103 | 0.34562 | 0.274407 | 6.96E-12 |  |
| TRDMT1 | 0.304946 | 0.202272 | 0.163734 | 0.259 | 0.196464 | 8.14E-12 |  |
| LPAR6 | 0.232608 | 0.315473 | 0.268497 | 0.365033 | 0.289474 | 9.24E-12 |  |
| DCAF8 | 0.200303 | 0.436674 | 0.380067 | 0.499816 | 0.405785 | 9.58E-12 |  |
| TACC3 | 0.217067 | 0.38343 | 0.32987 | 0.46185 | 0.376055 | 1.01E-11 |  |
| BAG3 | 0.26814 | 0.259414 | 0.215414 | 0.318712 | 0.249498 | 1.04E-11 |  |
| SESN3 | 0.245009 | 0.420777 | 0.355056 | 0.427694 | 0.353355 | 1.04E-11 |  |
| WIZ | 0.24841 | 0.285384 | 0.240243 | 0.350289 | 0.278626 | 1.18E-11 |  |
| CD28 | 0.148313 | 0.859724 | 0.775734 | 0.751444 | 0.680193 | 1.28E-11 |  |
| TPST2 | 0.329413 | 0.14969 | 0.119132 | 0.205676 | 0.150462 | 1.31E-11 |  |
| PCNX4 | 0.373385 | 0.131286 | 0.101349 | 0.176926 | 0.126356 | 1.39E-11 |  |
| ERICH1 | 0.240489 | 0.309162 | 0.261692 | 0.369333 | 0.293893 | 1.47E-11 |  |
| JMJD6 | 0.263533 | 0.20407 | 0.17 | 0.268829 | 0.203294 | 1.48E-11 |  |
| RYBP | 0.203643 | 0.501687 | 0.435643 | 0.551911 | 0.469667 | 1.51E-11 |  |
| RB1 | 0.288312 | 0.192604 | 0.157716 | 0.263054 | 0.198272 | 1.57E-11 |  |
| B3GAT3 | 0.296809 | 0.181719 | 0.147928 | 0.246836 | 0.185014 | 1.66E-11 |  |
| GNA13 | 0.178354 | 0.597583 | 0.52809 | 0.625384 | 0.532744 | 1.69E-11 |  |
| MPV17 | 0.208449 | 0.446853 | 0.386737 | 0.479174 | 0.394335 | 1.92E-11 |  |
| DUS3L | 0.276034 | 0.227324 | 0.187737 | 0.291436 | 0.224387 | 1.97E-11 |  |
| UPF3A | 0.288129 | 0.183802 | 0.150527 | 0.249294 | 0.187826 | 1.99E-11 |  |
| DDIT3 | 0.335734 | 0.148857 | 0.117951 | 0.193636 | 0.139815 | 2.13E-11 |  |
| AKT2 | 0.237403 | 0.301859 | 0.256058 | 0.382971 | 0.30675 | 2.33E-11 |  |
| TTLL3 | 0.340264 | 0.151884 | 0.119973 | 0.201499 | 0.14765 | 2.37E-11 |  |
| CSNK1D | 0.22043 | 0.370638 | 0.318122 | 0.446369 | 0.363801 | 2.43E-11 |  |
| NONO | 0.221638 | 0.34784 | 0.298305 | 0.435926 | 0.350944 | 2.51E-11 |  |
| CCDC88C | 0.1504 | 0.812831 | 0.732362 | 0.762133 | 0.678988 | 2.85E-11 |  |
| PLK3 | 0.217441 | 0.408737 | 0.35155 | 0.468362 | 0.389514 | 3.07E-11 |  |
| MAP7 | 1.53801 | 0.01915 | 0.006595 | 0.02875 | 0.010245 | 3.24E-11 |  |
| MTHFD1L | 0.300131 | 0.227057 | 0.184411 | 0.27915 | 0.216553 | 3.4E-11 |  |
| ABCA2 | 0.374419 | 0.148277 | 0.114383 | 0.191178 | 0.14082 | 3.49E-11 |  |
| UBA6 | 0.279743 | 0.214473 | 0.17667 | 0.285539 | 0.220972 | 3.83E-11 |  |
| DUSP16 | 0.33351 | 0.178451 | 0.141619 | 0.207765 | 0.154078 | 3.97E-11 |  |
| HMG20B | 0.24109 | 0.269538 | 0.228058 | 0.332105 | 0.26135 | 4.15E-11 |  |
| RNPS1 | 0.207252 | 0.410624 | 0.355677 | 0.49527 | 0.407393 | 4.54E-11 |  |
| CNOT6L | 0.119051 | 1.040269 | 0.957873 | 0.84949 | 0.787666 | 5.02E-11 |  |
| CYLD | 0.118471 | 1.114369 | 1.026516 | 0.861408 | 0.797509 | 5.29E-11 |  |
| LRRC8B | 0.26134 | 0.220356 | 0.183846 | 0.283204 | 0.217155 | 5.59E-11 |  |
| RBM11 | 1.052679 | 0.033671 | 0.016232 | 0.04374 | 0.020691 | 5.61E-11 |  |
| CDV3 | 0.223963 | 0.337349 | 0.288842 | 0.424868 | 0.346324 | 6.04E-11 |  |
| CYB5R3 | 0.201303 | 0.358292 | 0.31163 | 0.421551 | 0.340297 | 6.12E-11 |  |
| EXOSC7 | 0.246171 | 0.255511 | 0.215429 | 0.320064 | 0.251306 | 6.2E-11 |  |
| ULBP2 | 2.783019 | 0.007563 | 0.001099 | 0.013392 | 0.001607 | 6.23E-11 |  |
| LIMK2 | 0.300616 | 0.134804 | 0.109448 | 0.182455 | 0.130976 | 6.34E-11 |  |
| RUFY1 | 0.229102 | 0.260532 | 0.222277 | 0.322767 | 0.251708 | 6.57E-11 |  |
| ETV6 | 0.237507 | 0.289201 | 0.245303 | 0.350289 | 0.278425 | 6.82E-11 |  |
| AGMAT | 0.257175 | 0.25848 | 0.216276 | 0.315518 | 0.248694 | 6.91E-11 |  |
| TBC1D9B | 0.185624 | 0.534438 | 0.469914 | 0.566286 | 0.474488 | 6.95E-11 |  |
| TIMM10B | 0.26862 | 0.222679 | 0.184848 | 0.278044 | 0.214745 | 7.33E-11 |  |
| NIPAL3 | 0.246467 | 0.294225 | 0.248019 | 0.356432 | 0.28626 | 7.75E-11 |  |
| NCBP2AS2 | 0.208684 | 0.340127 | 0.294321 | 0.405209 | 0.325231 | 8.01E-11 |  |
| EIF3A | 0.17248 | 0.601444 | 0.533671 | 0.618749 | 0.529932 | 8.05E-11 |  |
| HES4 | 1.083254 | 0.028003 | 0.013216 | 0.037965 | 0.016673 | 8.78E-11 |  |
| FLOT2 | 0.187958 | 0.468672 | 0.411423 | 0.530409 | 0.439132 | 9.08E-11 |  |
| ARHGAP27 | 0.271043 | 0.180071 | 0.149228 | 0.244748 | 0.184612 | 9.38E-11 |  |
| ERCC1 | 0.277168 | 0.181835 | 0.150052 | 0.239833 | 0.1818 | 1E-10 |  |
| RFX5 | 0.278784 | 0.218414 | 0.180036 | 0.28001 | 0.21816 | 1E-10 |  |
| MSI2 | 0.200673 | 0.36306 | 0.315914 | 0.429291 | 0.348935 | 1.08E-10 |  |
| NME2 | 0.186558 | 0.508246 | 0.446595 | 0.5626 | 0.477903 | 1.17E-10 |  |
| UBXN11 | 0.24439 | 0.180447 | 0.152328 | 0.23713 | 0.177782 | 1.23E-10 |  |
| GRPEL1 | 0.255731 | 0.239111 | 0.200271 | 0.302863 | 0.237244 | 1.24E-10 |  |
| CLDND1 | 0.285104 | 0.219782 | 0.180371 | 0.264037 | 0.204299 | 1.31E-10 |  |
| MIF4GD | 0.273309 | 0.157342 | 0.130188 | 0.21268 | 0.157091 | 1.32E-10 |  |
| PSMB2 | 0.254851 | 0.19771 | 0.165695 | 0.272515 | 0.209321 | 1.33E-10 |  |
| ACADVL | 0.25159 | 0.245232 | 0.205987 | 0.311586 | 0.246284 | 1.41E-10 |  |
| RAB5IF | 0.272268 | 0.171693 | 0.142165 | 0.24573 | 0.185818 | 1.43E-10 |  |
| NOL9 | 0.228287 | 0.302861 | 0.258537 | 0.368841 | 0.297509 | 1.46E-10 |  |
| RETREG2 | 0.260684 | 0.219739 | 0.183414 | 0.293156 | 0.226798 | 1.48E-10 |  |
| FASN | 0.266808 | 0.18723 | 0.155617 | 0.245485 | 0.18642 | 1.49E-10 |  |
| SNRPA1 | 0.263064 | 0.214675 | 0.178892 | 0.289593 | 0.225191 | 1.55E-10 |  |
| SCML4 | 0.199443 | 0.392325 | 0.34167 | 0.439857 | 0.358578 | 1.58E-10 |  |
| VAMP5 | 0.276595 | 0.185937 | 0.153498 | 0.243765 | 0.185215 | 1.61E-10 |  |
| ZNF410 | 0.222139 | 0.309641 | 0.265453 | 0.377196 | 0.301728 | 1.63E-10 |  |
| PEF1 | 0.29997 | 0.175718 | 0.14273 | 0.240324 | 0.182804 | 1.66E-10 |  |
| RAB11FIP2 | 0.214419 | 0.313534 | 0.270233 | 0.379162 | 0.303937 | 1.8E-10 |  |
| TRAFD1 | 0.277774 | 0.185415 | 0.152942 | 0.242905 | 0.185014 | 1.81E-10 |  |
| CTPS1 | 0.338173 | 0.137525 | 0.108788 | 0.189212 | 0.139012 | 1.82E-10 |  |
| HNRNPK | 0.178473 | 0.580393 | 0.512857 | 0.62477 | 0.53857 | 1.85E-10 |  |
| PRKAB1 | 0.28074 | 0.16985 | 0.139816 | 0.21526 | 0.160908 | 2.08E-10 |  |
| IVNS1ABP | 0.167137 | 0.666491 | 0.593582 | 0.652783 | 0.568702 | 2.2E-10 |  |
| CAMK2D | 0.274079 | 0.163472 | 0.135188 | 0.21268 | 0.157895 | 2.24E-10 |  |
| MSH6 | 0.238532 | 0.246846 | 0.209229 | 0.31134 | 0.243873 | 2.24E-10 |  |
| RNMT | 0.226325 | 0.251194 | 0.214723 | 0.331859 | 0.259743 | 2.35E-10 |  |
| SMCO4 | 1.110097 | 0.017991 | 0.008335 | 0.031208 | 0.012455 | 2.36E-10 |  |
| UBE2L6 | 0.163806 | 0.662971 | 0.591812 | 0.635827 | 0.549217 | 2.36E-10 |  |
| SEC14L1 | 0.297852 | 0.148647 | 0.120919 | 0.206168 | 0.152471 | 2.37E-10 |  |
| AHRR | 1.972677 | 0.01344 | 0.003424 | 0.017447 | 0.004018 | 2.48E-10 |  |
| ECE1 | 0.186253 | 0.465263 | 0.408913 | 0.515665 | 0.42728 | 2.52E-10 |  |
| ARPP19 | 0.2652 | 0.210971 | 0.175546 | 0.284802 | 0.222378 | 2.7E-10 |  |
| MEF2A | 0.2246 | 0.298964 | 0.255863 | 0.372773 | 0.300321 | 2.97E-10 |  |
| STX4 | 0.242869 | 0.218694 | 0.18481 | 0.280256 | 0.216352 | 2.97E-10 |  |
| RIPK1 | 0.22382 | 0.250456 | 0.214464 | 0.317361 | 0.249699 | 3E-10 |  |
| PRKCH | 0.165228 | 0.614822 | 0.548291 | 0.645902 | 0.557654 | 3.11E-10 |  |
| MTRR | 0.286647 | 0.130164 | 0.106709 | 0.183438 | 0.132985 | 3.11E-10 |  |
| ZNF493 | 0.248271 | 0.24743 | 0.208313 | 0.30446 | 0.239454 | 3.14E-10 |  |
| LAMTOR2 | 0.235852 | 0.253355 | 0.215145 | 0.313183 | 0.248292 | 3.61E-10 |  |
| TRMT10C | 0.23506 | 0.22775 | 0.193508 | 0.295614 | 0.229409 | 3.67E-10 |  |
| DNPH1 | 0.252277 | 0.217492 | 0.182599 | 0.277307 | 0.215348 | 3.7E-10 |  |
| PSMC4 | 0.285844 | 0.148741 | 0.122007 | 0.211574 | 0.157895 | 3.73E-10 |  |
| TNFSF10 | 0.229652 | 0.299428 | 0.255364 | 0.350166 | 0.283045 | 3.82E-10 |  |
| CTR9 | 0.257416 | 0.176158 | 0.147371 | 0.238481 | 0.180996 | 3.9E-10 |  |
| LENG9 | 1.021605 | 0.024871 | 0.012251 | 0.035631 | 0.015669 | 3.96E-10 |  |
| CHD7 | 0.180558 | 0.501363 | 0.442384 | 0.533604 | 0.455002 | 4.09E-10 |  |
| LIMD1 | 0.280896 | 0.163473 | 0.134551 | 0.21268 | 0.159904 | 4.34E-10 |  |
| CD38 | 0.222699 | 0.353125 | 0.302615 | 0.379039 | 0.308357 | 4.51E-10 |  |
| NUP98 | 0.207417 | 0.344727 | 0.298563 | 0.425605 | 0.348534 | 4.57E-10 |  |
| STXBP2 | 0.179258 | 0.458994 | 0.405364 | 0.514682 | 0.426276 | 4.73E-10 |  |
| CDKN2D | 0.22611 | 0.328339 | 0.280709 | 0.368964 | 0.29992 | 4.77E-10 |  |
| NPEPL1 | 0.237858 | 0.240562 | 0.203997 | 0.306303 | 0.242467 | 4.8E-10 |  |
| DNAJB9 | 0.216483 | 0.346776 | 0.298457 | 0.409755 | 0.335677 | 4.86E-10 |  |
| LPCAT4 | 0.308346 | 0.148834 | 0.120193 | 0.20113 | 0.150864 | 4.88E-10 |  |
| GBP1 | 0.272294 | 0.180276 | 0.149269 | 0.231478 | 0.176175 | 5.11E-10 |  |
| PPP1R15A | 0.118213 | 1.117427 | 1.029517 | 0.805504 | 0.749297 | 5.42E-10 |  |
| SPOCK2 | 0.140917 | 0.838294 | 0.760285 | 0.752181 | 0.675573 | 5.52E-10 |  |
| PDCD1 | 1.093729 | 0.02447 | 0.011466 | 0.037351 | 0.017075 | 5.54E-10 |  |
| RCL1 | 0.239655 | 0.244638 | 0.207196 | 0.305689 | 0.241462 | 5.64E-10 |  |
| TYMS | 1.668917 | 0.015556 | 0.004892 | 0.02187 | 0.00683 | 5.66E-10 |  |
| LAG3 | 1.290808 | 0.032187 | 0.013156 | 0.034648 | 0.015267 | 5.85E-10 |  |
| FAR1 | 0.282493 | 0.193055 | 0.158724 | 0.251014 | 0.193853 | 5.88E-10 |  |
| GRK6 | 0.154571 | 0.738077 | 0.663088 | 0.704386 | 0.621133 | 6.49E-10 |  |
| NEMP2 | 0.243442 | 0.225954 | 0.190869 | 0.27657 | 0.216352 | 6.74E-10 |  |
| YWHAE | 0.235219 | 0.214627 | 0.182338 | 0.292419 | 0.228405 | 6.78E-10 |  |
| DHX36 | 0.187656 | 0.408586 | 0.358751 | 0.481877 | 0.399759 | 6.8E-10 |  |
| GTPBP6 | 0.188203 | 0.437941 | 0.38438 | 0.493918 | 0.408397 | 7.25E-10 |  |
| DNAJC5 | 0.201377 | 0.35722 | 0.310682 | 0.430888 | 0.353957 | 7.47E-10 |  |
| FOXN2 | 0.218468 | 0.301899 | 0.259475 | 0.373879 | 0.302531 | 7.71E-10 |  |
| CARS2 | 0.221326 | 0.22063 | 0.189252 | 0.290331 | 0.226396 | 8E-10 |  |
| IBTK | 0.226503 | 0.261978 | 0.223913 | 0.330507 | 0.264564 | 8.14E-10 |  |
| GGNBP2 | 0.200613 | 0.412149 | 0.358644 | 0.48286 | 0.403978 | 8.61E-10 |  |
| ACO2 | 0.254838 | 0.167358 | 0.14026 | 0.229266 | 0.173363 | 8.62E-10 |  |
| TPGS2 | 0.232186 | 0.209458 | 0.178321 | 0.266495 | 0.206107 | 8.67E-10 |  |
| ISG15 | 0.172786 | 0.483394 | 0.428832 | 0.485072 | 0.40458 | 8.84E-10 |  |
| PRMT1 | 0.215136 | 0.319113 | 0.274904 | 0.393046 | 0.319204 | 9.66E-10 |  |
| MCM3AP | 0.218339 | 0.245698 | 0.211191 | 0.321293 | 0.253315 | 1.04E-09 |  |
| SNRPB | 0.19048 | 0.422804 | 0.370509 | 0.492935 | 0.411812 | 1.05E-09 |  |
| SLC5A6 | 0.215155 | 0.285064 | 0.24557 | 0.34304 | 0.275814 | 1.06E-09 |  |
| PPP3CA | 0.290396 | 0.157686 | 0.128936 | 0.206659 | 0.155283 | 1.17E-09 |  |
| AP3M2 | 0.259867 | 0.21722 | 0.181414 | 0.273252 | 0.21595 | 1.18E-09 |  |
| LAP3 | 0.258994 | 0.193227 | 0.161474 | 0.257894 | 0.20008 | 1.25E-09 |  |
| RABGAP1L | 0.163254 | 0.588502 | 0.525537 | 0.604865 | 0.516874 | 1.25E-09 |  |
| RNH1 | 0.179251 | 0.452621 | 0.399737 | 0.510136 | 0.42507 | 1.31E-09 |  |
| SLC44A5 | 1.484775 | 0.014486 | 0.005176 | 0.021133 | 0.006629 | 1.32E-09 |  |
| TUFM | 0.171054 | 0.537607 | 0.477499 | 0.580415 | 0.502611 | 1.34E-09 |  |
| AHNAK | 0.158489 | 0.618639 | 0.554277 | 0.486178 | 0.407995 | 1.37E-09 |  |
| DIS3 | 0.255507 | 0.203378 | 0.170369 | 0.269198 | 0.210526 | 1.4E-09 |  |
| PML | 0.218814 | 0.263815 | 0.226688 | 0.340091 | 0.273403 | 1.41E-09 |  |
| SFT2D1 | 0.268747 | 0.146953 | 0.121977 | 0.199042 | 0.148654 | 1.45E-09 |  |
| JOSD1 | 0.192334 | 0.370891 | 0.3246 | 0.438875 | 0.359783 | 1.5E-09 |  |
| CYRIB | 0.17759 | 0.473445 | 0.41861 | 0.52746 | 0.443953 | 1.52E-09 |  |
| NSUN2 | 0.221477 | 0.247159 | 0.211985 | 0.323258 | 0.256529 | 1.58E-09 |  |
| CAPRIN1 | 0.254941 | 0.140475 | 0.117721 | 0.202728 | 0.150864 | 1.59E-09 |  |
| GPR19 | 2.069752 | 0.009866 | 0.00235 | 0.01585 | 0.003616 | 1.6E-09 |  |
| SPAG9 | 0.265193 | 0.164516 | 0.136891 | 0.222263 | 0.169546 | 1.67E-09 |  |
| INPPL1 | 0.269748 | 0.141765 | 0.117589 | 0.199902 | 0.149056 | 1.67E-09 |  |
| SERINC5 | 0.15145 | 0.735308 | 0.662031 | 0.698489 | 0.607473 | 1.67E-09 |  |
| LRRC8C | 0.167366 | 0.51719 | 0.46054 | 0.5626 | 0.479309 | 1.79E-09 |  |
| CCNDBP1 | 0.220089 | 0.315165 | 0.270573 | 0.371544 | 0.303335 | 1.79E-09 |  |
| FKBP8 | 0.204355 | 0.273097 | 0.237029 | 0.360732 | 0.290679 | 1.93E-09 |  |
| CLTA | 0.243226 | 0.202715 | 0.171264 | 0.266863 | 0.208517 | 1.94E-09 |  |
| NFKB2 | 0.192552 | 0.433289 | 0.379152 | 0.483966 | 0.404379 | 1.97E-09 |  |
| P2RY11 | 0.215792 | 0.234301 | 0.20175 | 0.296719 | 0.233427 | 2.05E-09 |  |
| NT5E | 1.465268 | 0.018765 | 0.006796 | 0.024696 | 0.00904 | 2.05E-09 |  |
| UBA3 | 0.208628 | 0.266309 | 0.230453 | 0.338862 | 0.270189 | 2.06E-09 |  |
| PMF1 | 0.219148 | 0.264388 | 0.227129 | 0.336528 | 0.271796 | 2.07E-09 |  |
| NPRL3 | 0.221327 | 0.228261 | 0.195797 | 0.289593 | 0.226999 | 2.08E-09 |  |
| SFI1 | 0.188934 | 0.376268 | 0.330083 | 0.4417 | 0.362997 | 2.11E-09 |  |
| PACSIN2 | 0.204696 | 0.260521 | 0.22606 | 0.329647 | 0.263761 | 2.16E-09 |  |
| MAN2B2 | 0.23146 | 0.232491 | 0.19803 | 0.29844 | 0.237244 | 2.32E-09 |  |
| SHISAL2A | 0.265114 | 0.183405 | 0.152617 | 0.236516 | 0.182603 | 2.38E-09 |  |
| KANSL2 | 0.217537 | 0.270856 | 0.232945 | 0.347709 | 0.278626 | 2.42E-09 |  |
| RHOT2 | 0.218349 | 0.281567 | 0.242021 | 0.348077 | 0.281438 | 2.43E-09 |  |
| ARL6IP5 | 0.164939 | 0.587024 | 0.523605 | 0.624892 | 0.53636 | 2.43E-09 |  |
| SEH1L | 0.2776 | 0.183416 | 0.151311 | 0.238973 | 0.185818 | 2.48E-09 |  |
| DDX56 | 0.234806 | 0.179687 | 0.152698 | 0.244256 | 0.187425 | 2.71E-09 |  |
| EHMT1 | 0.157939 | 0.530834 | 0.475789 | 0.566163 | 0.481117 | 2.75E-09 |  |
| TNFRSF1B | 0.27214 | 0.208833 | 0.172932 | 0.251014 | 0.196464 | 2.8E-09 |  |
| PLEKHB2 | 0.202663 | 0.342772 | 0.29785 | 0.408527 | 0.337485 | 2.88E-09 |  |
| MAVS | 0.19559 | 0.275693 | 0.24074 | 0.34906 | 0.278626 | 3.15E-09 |  |
| DPP4 | 0.213571 | 0.342443 | 0.295323 | 0.374002 | 0.306951 | 3.18E-09 |  |
| HDAC10 | 0.243407 | 0.154286 | 0.130333 | 0.209239 | 0.157694 | 3.22E-09 |  |
| PPP2R5D | 0.214167 | 0.247722 | 0.213547 | 0.315518 | 0.251105 | 3.25E-09 |  |
| MAPK8IP3 | 0.265724 | 0.150338 | 0.125048 | 0.212065 | 0.159904 | 3.27E-09 |  |
| MT1E | 2.918447 | 0.005598 | 0.00074 | 0.011672 | 0.001607 | 3.28E-09 |  |
| RELA | 0.226645 | 0.233128 | 0.199236 | 0.30446 | 0.242467 | 3.32E-09 |  |
| INPP5D | 0.17237 | 0.513366 | 0.455553 | 0.561617 | 0.475291 | 3.38E-09 |  |
| GZMB | 1.067331 | 0.018487 | 0.008822 | 0.02703 | 0.010647 | 3.4E-09 |  |
| TXLNA | 0.192868 | 0.32938 | 0.288163 | 0.396363 | 0.324628 | 3.41E-09 |  |
| ABHD3 | 0.24024 | 0.194376 | 0.16456 | 0.252365 | 0.196464 | 3.43E-09 |  |
| IFIH1 | 0.243377 | 0.196198 | 0.165741 | 0.247205 | 0.192045 | 3.58E-09 |  |
| XPC | 0.214901 | 0.22726 | 0.195808 | 0.296351 | 0.234632 | 3.73E-09 |  |
| SULT1B1 | 0.195337 | 0.302634 | 0.264311 | 0.351272 | 0.282644 | 3.81E-09 |  |
| CIR1 | 0.230968 | 0.171027 | 0.145726 | 0.233075 | 0.177983 | 3.87E-09 |  |
| SCAMP4 | 0.258031 | 0.161877 | 0.135366 | 0.21096 | 0.159904 | 3.98E-09 |  |
| ZNF655 | 0.188378 | 0.385875 | 0.338641 | 0.456936 | 0.378666 | 4.05E-09 |  |
| CHP1 | 0.252882 | 0.147819 | 0.124052 | 0.209608 | 0.158497 | 4.15E-09 |  |
| MCMBP | 0.176351 | 0.366218 | 0.32408 | 0.435803 | 0.357573 | 4.16E-09 |  |
| DNAJB14 | 0.253724 | 0.168256 | 0.141121 | 0.229881 | 0.177581 | 4.18E-09 |  |
| CARM1 | 0.214535 | 0.200254 | 0.172584 | 0.261457 | 0.202692 | 4.24E-09 |  |
| PER1 | 0.179272 | 0.48075 | 0.424573 | 0.517877 | 0.444355 | 4.37E-09 |  |
| DIP2A | 0.169909 | 0.509901 | 0.45325 | 0.556334 | 0.474086 | 4.68E-09 |  |
| SRM | 0.200997 | 0.358731 | 0.312078 | 0.419708 | 0.350743 | 4.69E-09 |  |
| MAFK | 0.245068 | 0.182358 | 0.153869 | 0.24057 | 0.186219 | 4.8E-09 |  |
| SRF | 0.196356 | 0.293242 | 0.255927 | 0.359381 | 0.291683 | 4.86E-09 |  |
| ADK | 0.213266 | 0.206093 | 0.177772 | 0.268092 | 0.209723 | 4.97E-09 |  |
| PIH1D1 | 0.193648 | 0.312947 | 0.273639 | 0.379531 | 0.309763 | 5.03E-09 |  |
| SLC3A2 | 0.179663 | 0.41695 | 0.368129 | 0.473154 | 0.393732 | 5.23E-09 |  |
| CDC16 | 0.200176 | 0.309892 | 0.269744 | 0.382848 | 0.314182 | 5.35E-09 |  |
| AKR1B1 | 0.179788 | 0.333799 | 0.294689 | 0.395012 | 0.321012 | 5.38E-09 |  |
| ANXA2R | 0.213923 | 0.300798 | 0.259345 | 0.358152 | 0.291884 | 5.63E-09 |  |
| NKTR | 0.190615 | 0.372998 | 0.326833 | 0.452267 | 0.373644 | 5.92E-09 |  |
| NFE2L2 | 0.177834 | 0.453536 | 0.400939 | 0.495638 | 0.420852 | 6.01E-09 |  |
| TBX21 | 1.049141 | 0.015836 | 0.007653 | 0.026907 | 0.010848 | 6.44E-09 |  |
| RREB1 | 0.20875 | 0.262192 | 0.226872 | 0.328664 | 0.265569 | 6.45E-09 |  |
| PCBP1 | 0.119332 | 0.926784 | 0.85321 | 0.819142 | 0.745078 | 6.63E-09 |  |
| CSNK1G2 | 0.169797 | 0.456124 | 0.405479 | 0.522177 | 0.439936 | 6.92E-09 |  |
| FOXO1 | 0.11964 | 0.993874 | 0.914779 | 0.824917 | 0.756127 | 7.37E-09 |  |
| ETF1 | 0.235572 | 0.172908 | 0.146859 | 0.242044 | 0.186621 | 7.5E-09 |  |
| NLRC5 | 0.14877 | 0.731877 | 0.660167 | 0.704632 | 0.608076 | 7.59E-09 |  |
| YWHAQ | 0.216815 | 0.242131 | 0.208345 | 0.321293 | 0.258738 | 7.94E-09 |  |
| TMC6 | 0.156242 | 0.657288 | 0.589823 | 0.656346 | 0.573323 | 8.1E-09 |  |
| EPC2 | 0.163629 | 0.564859 | 0.504293 | 0.607814 | 0.522499 | 8.31E-09 |  |
| THADA | 0.221573 | 0.234254 | 0.200904 | 0.304091 | 0.242266 | 8.88E-09 |  |
| ACAA2 | 0.220499 | 0.207272 | 0.177895 | 0.265143 | 0.206308 | 9.2E-09 |  |
| DNTTIP2 | 0.173293 | 0.428074 | 0.379623 | 0.494532 | 0.414825 | 9.57E-09 |  |
| NPIPB15 | 1.065033 | 0.017542 | 0.008384 | 0.026416 | 0.010647 | 9.84E-09 |  |
| PPP1R35 | 0.183035 | 0.337602 | 0.297376 | 0.400663 | 0.330454 | 1.02E-08 |  |
| SMARCC1 | 0.184224 | 0.348469 | 0.306695 | 0.430151 | 0.356971 | 1.05E-08 |  |
| FTL | 0.121697 | 0.935905 | 0.860196 | 0.815948 | 0.735034 | 1.12E-08 |  |
| NOLC1 | 0.257295 | 0.171778 | 0.143719 | 0.236393 | 0.183608 | 1.16E-08 |  |
| GIPC1 | 0.207206 | 0.291353 | 0.252374 | 0.356923 | 0.291483 | 1.18E-08 |  |
| UBE2R2 | 0.145872 | 0.662952 | 0.599198 | 0.661015 | 0.573925 | 1.18E-08 |  |
| NR4A1 | 0.21146 | 0.194967 | 0.168385 | 0.234304 | 0.181398 | 1.2E-08 |  |
| SRP54 | 0.200259 | 0.20294 | 0.176638 | 0.273252 | 0.213138 | 1.22E-08 |  |
| RAB35 | 0.18796 | 0.310442 | 0.272521 | 0.384445 | 0.31358 | 1.23E-08 |  |
| WDR86 | 1.05206 | 0.024766 | 0.011944 | 0.033911 | 0.016071 | 1.35E-08 |  |
| KLF7 | 0.198626 | 0.291916 | 0.25437 | 0.358275 | 0.29309 | 1.37E-08 |  |
| SUSD6 | 0.198854 | 0.276356 | 0.240773 | 0.345743 | 0.281639 | 1.4E-08 |  |
| ZDHHC3 | 0.199666 | 0.257654 | 0.224352 | 0.330262 | 0.267778 | 1.43E-08 |  |
| SKIL | 0.192224 | 0.363278 | 0.317961 | 0.410861 | 0.341503 | 1.48E-08 |  |
| SCML1 | 0.274477 | 0.185171 | 0.15309 | 0.231847 | 0.181197 | 1.49E-08 |  |
| INTS4 | 0.178737 | 0.403631 | 0.356598 | 0.455461 | 0.38188 | 1.56E-08 |  |
| UVRAG | 0.213697 | 0.168837 | 0.145592 | 0.223983 | 0.171957 | 1.69E-08 |  |
| MON1B | 0.197268 | 0.286648 | 0.250015 | 0.352255 | 0.287465 | 1.69E-08 |  |
| CTNNBL1 | 0.234747 | 0.162037 | 0.137704 | 0.224106 | 0.172157 | 1.88E-08 |  |
| CZIB | 0.233328 | 0.186173 | 0.158372 | 0.243887 | 0.191844 | 1.88E-08 |  |
| RCC2 | 0.193436 | 0.280063 | 0.244921 | 0.357906 | 0.290076 | 1.9E-08 |  |
| LPP | 0.176446 | 0.28641 | 0.253438 | 0.362207 | 0.293491 | 1.91E-08 |  |
| PHF19 | 0.182011 | 0.386273 | 0.340489 | 0.419708 | 0.349538 | 1.98E-08 |  |
| SPTY2D1 | 0.193389 | 0.266825 | 0.233352 | 0.340828 | 0.275211 | 2.13E-08 |  |
| DENND1A | 0.249101 | 0.181983 | 0.153124 | 0.239096 | 0.187425 | 2.4E-08 |  |
| VMP1 | 0.201469 | 0.220438 | 0.191707 | 0.289225 | 0.230213 | 2.64E-08 |  |
| SIGMAR1 | 0.206704 | 0.219191 | 0.189932 | 0.28259 | 0.224588 | 2.69E-08 |  |
| HSP90AB1 | 0.149229 | 0.6339 | 0.571608 | 0.652783 | 0.576135 | 2.7E-08 |  |
| DCAF7 | 0.166616 | 0.363897 | 0.324207 | 0.433837 | 0.358377 | 2.71E-08 |  |
| HPS1 | 0.215223 | 0.209502 | 0.180468 | 0.276938 | 0.219767 | 2.79E-08 |  |
| CRKL | 0.171361 | 0.328764 | 0.291944 | 0.409878 | 0.336481 | 2.9E-08 |  |
| KLHL5 | 0.162181 | 0.211884 | 0.189355 | 0.273252 | 0.213138 | 3.01E-08 |  |
| LIMS2 | 0.206922 | 0.245793 | 0.212951 | 0.305566 | 0.246685 | 3.1E-08 |  |
| CRYBG1 | 0.235171 | 0.170536 | 0.144885 | 0.227546 | 0.17718 | 3.14E-08 |  |
| TRAPPC10 | 0.151191 | 0.454856 | 0.409601 | 0.52316 | 0.436119 | 3.18E-08 |  |
| TLE4 | 0.254523 | 0.195005 | 0.163466 | 0.247451 | 0.196264 | 3.2E-08 |  |
| SDE2 | 0.228827 | 0.241168 | 0.205796 | 0.314166 | 0.255524 | 3.29E-08 |  |
| ZNF414 | 0.20728 | 0.204244 | 0.17691 | 0.266986 | 0.211732 | 3.43E-08 |  |
| TMF1 | 0.158295 | 0.42174 | 0.377914 | 0.487652 | 0.407794 | 3.49E-08 |  |
| NF2 | 0.195485 | 0.23813 | 0.207954 | 0.310849 | 0.249297 | 3.57E-08 |  |
| JMY | 0.18747 | 0.362725 | 0.318525 | 0.434697 | 0.360988 | 3.61E-08 |  |
| TPR | 0.169769 | 0.416442 | 0.37021 | 0.492812 | 0.414423 | 3.61E-08 |  |
| NETO2 | 1.241093 | 0.011747 | 0.004969 | 0.020396 | 0.007232 | 3.62E-08 |  |
| GIMAP8 | 0.18518 | 0.31131 | 0.273809 | 0.360241 | 0.297308 | 3.62E-08 |  |
| RAP2B | 0.201898 | 0.258153 | 0.22444 | 0.325593 | 0.264363 | 3.68E-08 |  |
| NDUFAF8 | 0.165494 | 0.428403 | 0.381974 | 0.482615 | 0.404178 | 3.69E-08 |  |
| FAM98B | 0.218541 | 0.198012 | 0.170178 | 0.258386 | 0.205102 | 3.81E-08 |  |
| PPP2CA | 0.18096 | 0.230057 | 0.202937 | 0.315272 | 0.251908 | 4.02E-08 |  |
| DPP9 | 0.193271 | 0.232895 | 0.203694 | 0.30446 | 0.244476 | 4.09E-08 |  |
| NAA25 | 0.222757 | 0.185342 | 0.158824 | 0.246836 | 0.194054 | 4.28E-08 |  |
| SEC16A | 0.217433 | 0.24187 | 0.208031 | 0.304829 | 0.248292 | 4.34E-08 |  |
| MCF2L2 | 1.289287 | 0.015024 | 0.006147 | 0.023344 | 0.009241 | 4.47E-08 |  |
| BIRC2 | 0.156827 | 0.487217 | 0.437032 | 0.531761 | 0.453596 | 4.51E-08 |  |
| MIOS | 0.235001 | 0.177247 | 0.150604 | 0.234427 | 0.184211 | 4.59E-08 |  |
| ZC3H7B | 0.198357 | 0.284501 | 0.247955 | 0.348569 | 0.286862 | 4.64E-08 |  |
| RNF126 | 0.173042 | 0.354151 | 0.314121 | 0.429168 | 0.357172 | 4.64E-08 |  |
| ZDHHC8 | 0.165009 | 0.250508 | 0.223434 | 0.318221 | 0.255725 | 4.77E-08 |  |
| USP15 | 0.150791 | 0.580069 | 0.5225 | 0.621698 | 0.542788 | 4.77E-08 |  |
| LUC7L | 0.16595 | 0.411438 | 0.366732 | 0.475488 | 0.399156 | 5.09E-08 |  |
| MTERF4 | 0.145375 | 0.671022 | 0.6067 | 0.637548 | 0.55223 | 5.31E-08 |  |
| HAGH | 0.21361 | 0.210815 | 0.181802 | 0.268952 | 0.214544 | 5.34E-08 |  |
| INTS10 | 0.192754 | 0.301959 | 0.264194 | 0.366753 | 0.302933 | 5.44E-08 |  |
| MAP3K3 | 0.18376 | 0.328282 | 0.289021 | 0.402384 | 0.333869 | 5.57E-08 |  |
| NUP50 | 0.172886 | 0.305357 | 0.270872 | 0.38936 | 0.321816 | 5.65E-08 |  |
| TMEM243 | 0.143419 | 0.682892 | 0.61827 | 0.663595 | 0.587585 | 5.83E-08 |  |
| ATP6V1F | 0.19725 | 0.263841 | 0.230125 | 0.342794 | 0.279028 | 5.88E-08 |  |
| DKC1 | 0.220677 | 0.200599 | 0.172147 | 0.258386 | 0.205504 | 6.02E-08 |  |
| SNX20 | 0.158153 | 0.459717 | 0.411985 | 0.500184 | 0.423865 | 6.02E-08 |  |
| PLPBP | 0.181751 | 0.219723 | 0.193715 | 0.270181 | 0.213941 | 6.1E-08 |  |
| SPSB3 | 0.187684 | 0.317453 | 0.278728 | 0.387025 | 0.321615 | 6.56E-08 |  |
| RASA1 | 0.173481 | 0.272749 | 0.241847 | 0.34992 | 0.282644 | 6.59E-08 |  |
| MKI67 | 1.415822 | 0.016232 | 0.006084 | 0.019413 | 0.00683 | 6.72E-08 |  |
| WWC3 | 0.219728 | 0.193506 | 0.166169 | 0.247696 | 0.195862 | 6.83E-08 |  |
| RBM26 | 0.168446 | 0.402237 | 0.357911 | 0.477454 | 0.398955 | 6.83E-08 |  |
| MX1 | 0.183738 | 0.253035 | 0.222777 | 0.294385 | 0.237445 | 6.92E-08 |  |
| ASCC3 | 0.213327 | 0.21703 | 0.187198 | 0.283942 | 0.227802 | 7.16E-08 |  |
| PRKCA | 0.162251 | 0.403882 | 0.360921 | 0.454847 | 0.382483 | 7.2E-08 |  |
| USP10 | 0.178766 | 0.218424 | 0.192968 | 0.289225 | 0.230816 | 7.33E-08 |  |
| DIMT1 | 0.214247 | 0.203765 | 0.175645 | 0.257525 | 0.204299 | 7.35E-08 |  |
| PHC2 | 0.192058 | 0.273609 | 0.239506 | 0.339845 | 0.278224 | 7.56E-08 |  |
| PPP1R9B | 0.161185 | 0.428679 | 0.383363 | 0.474014 | 0.400763 | 7.61E-08 |  |
| CERT1 | 0.181461 | 0.289401 | 0.255197 | 0.359012 | 0.294094 | 7.82E-08 |  |
| AASDHPPT | 0.223288 | 0.175169 | 0.150052 | 0.231847 | 0.1818 | 7.9E-08 |  |
| EIF2AK1 | 0.157308 | 0.418393 | 0.375172 | 0.484703 | 0.405986 | 8.01E-08 |  |
| EXOC8 | 0.23196 | 0.18672 | 0.158988 | 0.247451 | 0.196464 | 8.23E-08 |  |
| PRRC2C | 0.143334 | 0.623879 | 0.564875 | 0.655486 | 0.572519 | 8.73E-08 |  |
| POLR2F | 0.228418 | 0.171938 | 0.146762 | 0.235533 | 0.185014 | 9.38E-08 |  |
| SNU13 | 0.124746 | 0.827321 | 0.75879 | 0.748372 | 0.676979 | 9.4E-08 |  |
| SS18 | 0.175443 | 0.280897 | 0.248733 | 0.357292 | 0.291081 | 9.56E-08 |  |
| APH1A | 0.180386 | 0.307462 | 0.271325 | 0.389483 | 0.32262 | 9.61E-08 |  |
| FBXW7 | 0.137221 | 0.60735 | 0.552244 | 0.622927 | 0.540779 | 9.79E-08 |  |
| SCAMP3 | 0.196619 | 0.226322 | 0.197487 | 0.296597 | 0.239654 | 9.98E-08 |  |
| NEMF | 0.174666 | 0.309481 | 0.274192 | 0.382971 | 0.316191 | 1.04E-07 |  |
| ATR | 0.180553 | 0.193986 | 0.171167 | 0.261211 | 0.205906 | 1.05E-07 |  |
| PIGL | 0.212854 | 0.213416 | 0.184141 | 0.27571 | 0.220571 | 1.1E-07 |  |
| PARVG | 0.16021 | 0.3819 | 0.34176 | 0.448827 | 0.374448 | 1.13E-07 |  |
| ING3 | 0.207776 | 0.202597 | 0.175423 | 0.263792 | 0.210526 | 1.13E-07 |  |
| LGALS8 | 0.144855 | 0.449514 | 0.406572 | 0.507065 | 0.42949 | 1.14E-07 |  |
| UBR4 | 0.152983 | 0.465873 | 0.419001 | 0.530532 | 0.451989 | 1.15E-07 |  |
| RAB4B | 0.196352 | 0.220548 | 0.192484 | 0.282836 | 0.226798 | 1.16E-07 |  |
| MYLIP | 0.157883 | 0.532452 | 0.477258 | 0.552279 | 0.479711 | 1.16E-07 |  |
| CREBRF | 0.141358 | 0.6302 | 0.57138 | 0.65438 | 0.567899 | 1.16E-07 |  |
| TOP2A | 1.568578 | 0.010507 | 0.003542 | 0.017201 | 0.005625 | 1.16E-07 |  |
| FAM91A1 | 0.20232 | 0.182608 | 0.158714 | 0.250154 | 0.197469 | 1.17E-07 |  |
| PTBP1 | 0.172259 | 0.33836 | 0.300279 | 0.429783 | 0.354761 | 1.18E-07 |  |
| TNRC6A | 0.172731 | 0.301595 | 0.267564 | 0.381128 | 0.315789 | 1.21E-07 |  |
| RAB1B | 0.16482 | 0.38706 | 0.345273 | 0.459024 | 0.385697 | 1.26E-07 |  |
| ARIH1 | 0.162797 | 0.369098 | 0.329712 | 0.449318 | 0.374247 | 1.26E-07 |  |
| LRP10 | 0.157462 | 0.428868 | 0.384523 | 0.493058 | 0.417236 | 1.28E-07 |  |
| THAP4 | 0.207839 | 0.20889 | 0.180864 | 0.267969 | 0.216151 | 1.29E-07 |  |
| TYK2 | 0.169278 | 0.36672 | 0.326119 | 0.442806 | 0.37043 | 1.3E-07 |  |
| APOL3 | 0.20076 | 0.235441 | 0.204855 | 0.305443 | 0.248694 | 1.32E-07 |  |
| EIF6 | 0.184903 | 0.290855 | 0.255867 | 0.357784 | 0.296103 | 1.38E-07 |  |
| MCUB | 0.139419 | 0.623022 | 0.565633 | 0.643814 | 0.557051 | 1.46E-07 |  |
| FAM89A | 1.045927 | 0.018288 | 0.008857 | 0.025679 | 0.011249 | 1.47E-07 |  |
| NACC1 | 0.165295 | 0.281465 | 0.250995 | 0.348569 | 0.283648 | 1.47E-07 |  |
| ARHGAP25 | 0.165461 | 0.308713 | 0.275262 | 0.380759 | 0.313781 | 1.48E-07 |  |
| YTHDC2 | 0.187712 | 0.273744 | 0.240347 | 0.350412 | 0.28867 | 1.5E-07 |  |
| LRRC58 | 0.189652 | 0.19497 | 0.170953 | 0.257894 | 0.2045 | 1.58E-07 |  |
| ARF6 | 0.129057 | 0.718764 | 0.657259 | 0.712864 | 0.631981 | 1.58E-07 |  |
| COMT | 0.172008 | 0.249122 | 0.221122 | 0.30876 | 0.248694 | 1.61E-07 |  |
| PPA1 | 0.180429 | 0.248903 | 0.219642 | 0.332105 | 0.270189 | 1.62E-07 |  |
| TLK2 | 0.16972 | 0.196011 | 0.174257 | 0.268338 | 0.211933 | 1.7E-07 |  |
| AP2A2 | 0.16276 | 0.363496 | 0.324716 | 0.430643 | 0.35918 | 1.71E-07 |  |
| SELPLG | 0.098925 | 1.082883 | 1.011118 | 0.84433 | 0.792688 | 1.76E-07 |  |
| BIRC3 | 0.180939 | 0.314516 | 0.277444 | 0.369456 | 0.305544 | 1.84E-07 |  |
| BECN1 | 0.209611 | 0.200954 | 0.173779 | 0.268092 | 0.216553 | 1.92E-07 |  |
| VTI1A | 0.19748 | 0.223369 | 0.194794 | 0.292542 | 0.237244 | 1.93E-07 |  |
| TBCD | 0.167072 | 0.268055 | 0.238743 | 0.343285 | 0.280434 | 1.99E-07 |  |
| CRISPLD2 | 1.146708 | 0.01402 | 0.006332 | 0.020396 | 0.007834 | 2.04E-07 |  |
| PHF21A | 0.189363 | 0.191901 | 0.168296 | 0.259737 | 0.206509 | 2.08E-07 |  |
| BTAF1 | 0.164198 | 0.384897 | 0.343491 | 0.457427 | 0.386902 | 2.11E-07 |  |
| ATP5MF | 0.193631 | 0.191121 | 0.167117 | 0.270795 | 0.215749 | 2.14E-07 |  |
| FST | 2.269235 | 0.013008 | 0.002698 | 0.013147 | 0.003415 | 2.2E-07 |  |
| ARL8B | 0.166166 | 0.234809 | 0.209264 | 0.313552 | 0.253515 | 2.22E-07 |  |
| SGMS1 | 0.205051 | 0.2039 | 0.176885 | 0.262932 | 0.211129 | 2.24E-07 |  |
| FOXK2 | 0.21157 | 0.202415 | 0.174805 | 0.272638 | 0.219767 | 2.25E-07 |  |
| CCR5 | 1.383836 | 0.0183 | 0.007013 | 0.022976 | 0.009642 | 2.25E-07 |  |
| CTDP1 | 0.189773 | 0.265915 | 0.23314 | 0.336036 | 0.275814 | 2.33E-07 |  |
| DNAJA1 | 0.175786 | 0.315665 | 0.279454 | 0.387885 | 0.323222 | 2.36E-07 |  |
| SYNJ2 | 0.214799 | 0.226017 | 0.194751 | 0.276938 | 0.224588 | 2.44E-07 |  |
| PGD | 0.186706 | 0.189264 | 0.166289 | 0.251505 | 0.199478 | 2.48E-07 |  |
| PDE4D | 0.142491 | 0.686174 | 0.621642 | 0.643445 | 0.577943 | 2.49E-07 |  |
| CUL4A | 0.181666 | 0.272954 | 0.24066 | 0.3439 | 0.284452 | 2.53E-07 |  |
| HIC1 | 1.442569 | 0.008038 | 0.002957 | 0.014498 | 0.004219 | 2.59E-07 |  |
| WWP1 | 0.162634 | 0.310156 | 0.277091 | 0.387394 | 0.320209 | 2.71E-07 |  |
| SQSTM1 | 0.169068 | 0.350126 | 0.311408 | 0.415284 | 0.348935 | 2.79E-07 |  |
| COTL1 | 0.130567 | 0.747995 | 0.683273 | 0.693083 | 0.617718 | 2.84E-07 |  |
| PRPS1 | 0.182512 | 0.320325 | 0.28226 | 0.398452 | 0.331458 | 3.17E-07 |  |
| MPRIP | 0.143224 | 0.517973 | 0.469021 | 0.557194 | 0.482322 | 3.34E-07 |  |
| ITM2B | 0.12675 | 0.70551 | 0.646171 | 0.711635 | 0.635396 | 3.34E-07 |  |
| PPP6R3 | 0.152733 | 0.428629 | 0.385571 | 0.499816 | 0.424869 | 3.42E-07 |  |
| DDX42 | 0.1526 | 0.366498 | 0.329712 | 0.446738 | 0.375251 | 3.51E-07 |  |
| CAPZB | 0.10549 | 0.986485 | 0.916927 | 0.816439 | 0.755123 | 3.59E-07 |  |
| MFSD6 | 0.167624 | 0.249482 | 0.222115 | 0.316747 | 0.257734 | 3.65E-07 |  |
| MARCHF6 | 0.1564 | 0.437228 | 0.392308 | 0.516771 | 0.439534 | 3.93E-07 |  |
| MAP4 | 0.15874 | 0.344061 | 0.308212 | 0.406438 | 0.339695 | 3.97E-07 |  |
| PRKRA | 0.198774 | 0.21669 | 0.1888 | 0.281116 | 0.228807 | 4.02E-07 |  |
| RAB11FIP4 | 0.153531 | 0.307753 | 0.276685 | 0.376213 | 0.309763 | 4.07E-07 |  |
| ZFP36L1 | 0.09791 | 1.276365 | 1.192617 | 0.874432 | 0.814785 | 4.1E-07 |  |
| PURB | 0.148855 | 0.4209 | 0.379637 | 0.473031 | 0.399759 | 4.1E-07 |  |
| KCNAB2 | 0.132505 | 0.656229 | 0.598643 | 0.651677 | 0.574126 | 4.16E-07 |  |
| PICALM | 0.148712 | 0.411386 | 0.371093 | 0.485932 | 0.407794 | 4.24E-07 |  |
| PQBP1 | 0.151679 | 0.338836 | 0.305021 | 0.401892 | 0.335878 | 4.33E-07 |  |
| AKAP1 | 0.1696 | 0.227903 | 0.202626 | 0.285662 | 0.230414 | 4.5E-07 |  |
| PITPNM1 | 0.148562 | 0.368397 | 0.332349 | 0.424131 | 0.35918 | 4.55E-07 |  |
| ITK | 0.092946 | 1.194338 | 1.119818 | 0.874677 | 0.822218 | 4.56E-07 |  |
| ATP6V1E1 | 0.173784 | 0.216715 | 0.192121 | 0.286153 | 0.232222 | 4.8E-07 |  |
| ATP6V0E1 | 0.156955 | 0.381638 | 0.342297 | 0.466028 | 0.391724 | 4.82E-07 |  |
| YBX1 | 0.149947 | 0.523488 | 0.471811 | 0.578941 | 0.501406 | 4.92E-07 |  |
| NFATC1 | 0.167908 | 0.221492 | 0.197157 | 0.287505 | 0.231418 | 4.94E-07 |  |
| IDS | 0.149467 | 0.45732 | 0.412312 | 0.499201 | 0.421053 | 4.98E-07 |  |
| RBM5 | 0.164169 | 0.284584 | 0.253975 | 0.366384 | 0.301928 | 5.67E-07 |  |
| ARPC5L | 0.192096 | 0.246069 | 0.215392 | 0.306917 | 0.252913 | 5.77E-07 |  |
| ABCE1 | 0.182887 | 0.226294 | 0.199351 | 0.295491 | 0.241462 | 5.77E-07 |  |
| RAB21 | 0.139009 | 0.562118 | 0.510484 | 0.607077 | 0.52732 | 5.82E-07 |  |
| TRIM27 | 0.154989 | 0.31297 | 0.281091 | 0.377933 | 0.316593 | 5.86E-07 |  |
| MROH1 | 0.171098 | 0.259109 | 0.230132 | 0.316624 | 0.258337 | 5.93E-07 |  |
| ATXN7 | 0.140781 | 0.507409 | 0.460235 | 0.563828 | 0.486943 | 6.02E-07 |  |
| TRADD | 0.179757 | 0.272017 | 0.240151 | 0.321661 | 0.266975 | 6.09E-07 |  |
| DDX39A | 0.180762 | 0.254752 | 0.224752 | 0.324487 | 0.268783 | 6.29E-07 |  |
| INO80D | 0.165156 | 0.224999 | 0.200661 | 0.297457 | 0.239654 | 6.48E-07 |  |
| PAQR7 | 1.052849 | 0.01573 | 0.007582 | 0.02273 | 0.009843 | 6.54E-07 |  |
| ELK3 | 0.168702 | 0.238749 | 0.212402 | 0.309375 | 0.251507 | 6.63E-07 |  |
| ADPRM | 0.167281 | 0.232336 | 0.2069 | 0.286276 | 0.231418 | 6.63E-07 |  |
| KRI1 | 0.1547 | 0.355761 | 0.319587 | 0.414056 | 0.348333 | 6.73E-07 |  |
| PPIA | 0.137836 | 0.540313 | 0.49108 | 0.591473 | 0.509642 | 6.8E-07 |  |
| NAA15 | 0.147887 | 0.427103 | 0.385491 | 0.501413 | 0.428084 | 6.85E-07 |  |
| MGAT4B | 0.182393 | 0.200184 | 0.17641 | 0.258754 | 0.208116 | 6.99E-07 |  |
| SLC8B1 | 0.189582 | 0.216477 | 0.18982 | 0.271164 | 0.219968 | 7.11E-07 |  |
| NELFCD | 0.171581 | 0.30557 | 0.271306 | 0.37695 | 0.313781 | 7.15E-07 |  |
| CMTM7 | 0.155347 | 0.415868 | 0.373414 | 0.470328 | 0.399558 | 7.32E-07 |  |
| RNF213 | 0.089712 | 1.130416 | 1.062264 | 0.872343 | 0.820209 | 7.33E-07 |  |
| ANGPT1 | 4.642792 | 0.004346 | 0.000174 | 0.006266 | 0.000201 | 7.37E-07 |  |
| C2CD5 | 0.196015 | 0.194757 | 0.170015 | 0.260597 | 0.209723 | 7.39E-07 |  |
| LRFN2 | 2.741504 | 0.006649 | 0.000994 | 0.008478 | 0.001205 | 7.54E-07 |  |
| EPSTI1 | 0.161419 | 0.292049 | 0.261134 | 0.338985 | 0.27963 | 7.81E-07 |  |
| GPI | 0.177043 | 0.18931 | 0.167447 | 0.268583 | 0.215548 | 7.93E-07 |  |
| PAF1 | 0.130936 | 0.217065 | 0.198232 | 0.28431 | 0.227601 | 8E-07 |  |
| NHP2 | 0.144517 | 0.518807 | 0.469355 | 0.545399 | 0.476497 | 8.26E-07 |  |
| VPS13B | 0.150934 | 0.470274 | 0.423561 | 0.536675 | 0.462435 | 8.46E-07 |  |
| ACTR1A | 0.16537 | 0.272391 | 0.242891 | 0.344637 | 0.286661 | 8.5E-07 |  |
| NSMAF | 0.189603 | 0.217463 | 0.190682 | 0.283819 | 0.232423 | 8.6E-07 |  |
| AKT1 | 0.149718 | 0.290982 | 0.262299 | 0.366998 | 0.30233 | 8.6E-07 |  |
| HBEGF | 1.337465 | 0.010275 | 0.004066 | 0.01585 | 0.005424 | 8.62E-07 |  |
| PITRM1 | 0.188436 | 0.22198 | 0.1948 | 0.289593 | 0.235838 | 8.66E-07 |  |
| MAPK1IP1L | 0.129976 | 0.581437 | 0.531344 | 0.620346 | 0.539775 | 8.67E-07 |  |
| CRYGN | 2.350809 | 0.006741 | 0.001321 | 0.009583 | 0.001808 | 9.13E-07 |  |
| TNIK | 0.136375 | 0.604142 | 0.54965 | 0.618995 | 0.549819 | 9.13E-07 |  |
| APRT | 0.120168 | 0.781147 | 0.718718 | 0.731785 | 0.656689 | 9.31E-07 |  |
| CCDC85B | 0.167052 | 0.362966 | 0.32328 | 0.420936 | 0.359582 | 9.31E-07 |  |
| PCSK7 | 0.138692 | 0.53096 | 0.482293 | 0.577712 | 0.508437 | 9.41E-07 |  |
| RSF1 | 0.169406 | 0.222799 | 0.198115 | 0.292296 | 0.236641 | 9.43E-07 |  |
| EIF2S1 | 0.164816 | 0.25936 | 0.23136 | 0.327559 | 0.269184 | 9.8E-07 |  |
| NRARP | 1.375248 | 0.010189 | 0.003928 | 0.015112 | 0.005022 | 1.01E-06 |  |
| DENND4B | 0.145184 | 0.413489 | 0.373904 | 0.489741 | 0.411812 | 1.04E-06 |  |
| GNG10 | 0.160101 | 0.298837 | 0.267448 | 0.360855 | 0.300121 | 1.05E-06 |  |
| NPEPPS | 0.145681 | 0.28895 | 0.261197 | 0.369701 | 0.306951 | 1.08E-06 |  |
| LSG1 | 0.161767 | 0.236649 | 0.211548 | 0.30618 | 0.249297 | 1.08E-06 |  |
| CAND1 | 0.141168 | 0.45646 | 0.413911 | 0.52574 | 0.449377 | 1.1E-06 |  |
| PGLS | 0.135636 | 0.398108 | 0.362385 | 0.459393 | 0.386701 | 1.11E-06 |  |
| STAT2 | 0.176628 | 0.226608 | 0.200495 | 0.296228 | 0.242266 | 1.11E-06 |  |
| SOD1 | 0.153346 | 0.399899 | 0.359575 | 0.481386 | 0.413218 | 1.12E-06 |  |
| DYNC1LI2 | 0.164128 | 0.193533 | 0.172722 | 0.256174 | 0.205705 | 1.12E-06 |  |
| IRF9 | 0.161198 | 0.301009 | 0.269186 | 0.371176 | 0.310767 | 1.13E-06 |  |
| MORF4L1 | 0.191441 | 0.205636 | 0.180082 | 0.280256 | 0.227802 | 1.15E-06 |  |
| ITPRIP | 0.143907 | 0.376004 | 0.340308 | 0.425114 | 0.357372 | 1.16E-06 |  |
| ZBTB1 | 0.129269 | 0.644374 | 0.589148 | 0.649466 | 0.569908 | 1.18E-06 |  |
| ZDHHC18 | 0.185361 | 0.217372 | 0.191163 | 0.281361 | 0.230816 | 1.19E-06 |  |
| CXCR6 | 1.897274 | 0.009215 | 0.002474 | 0.010935 | 0.002611 | 1.22E-06 |  |
| SNRNP70 | 0.159479 | 0.285504 | 0.255625 | 0.370807 | 0.308959 | 1.26E-06 |  |
| CDC42SE2 | 0.09785 | 1.048964 | 0.980178 | 0.844821 | 0.785657 | 1.26E-06 |  |
| RTCA | 0.183923 | 0.206196 | 0.181516 | 0.272392 | 0.221575 | 1.32E-06 |  |
| LRRC41 | 0.164203 | 0.230555 | 0.205752 | 0.294139 | 0.240056 | 1.35E-06 |  |
| GIMAP1 | 0.124477 | 0.602427 | 0.552628 | 0.606217 | 0.533347 | 1.35E-06 |  |
| TPX2 | 1.078467 | 0.012584 | 0.005959 | 0.01929 | 0.007834 | 1.37E-06 |  |
| SRPK1 | 0.171967 | 0.212713 | 0.188811 | 0.283696 | 0.230816 | 1.39E-06 |  |
| DAPK2 | 1.526557 | 0.008951 | 0.003107 | 0.014252 | 0.00462 | 1.45E-06 |  |
| HNRNPL | 0.131216 | 0.582931 | 0.532252 | 0.636933 | 0.555243 | 1.47E-06 |  |
| UBE2Z | 0.158832 | 0.249273 | 0.223286 | 0.322521 | 0.264363 | 1.48E-06 |  |
| SRRM2 | 0.138523 | 0.468899 | 0.425971 | 0.535324 | 0.455404 | 1.52E-06 |  |
| IDH2 | 0.136509 | 0.519571 | 0.472663 | 0.548102 | 0.480916 | 1.53E-06 |  |
| GADD45GIP1 | 0.156281 | 0.257599 | 0.231152 | 0.326699 | 0.269787 | 1.53E-06 |  |
| CCDC163 | 1.338898 | 0.009888 | 0.003909 | 0.014252 | 0.00462 | 1.53E-06 |  |
| EXOC4 | 0.155972 | 0.303778 | 0.27265 | 0.385428 | 0.320008 | 1.53E-06 |  |
| ECH1 | 0.136192 | 0.517707 | 0.471071 | 0.553508 | 0.484733 | 1.56E-06 |  |
| ANKRD18A | 2.232613 | 0.006615 | 0.001408 | 0.010075 | 0.00221 | 1.56E-06 |  |
| USP14 | 0.158855 | 0.248304 | 0.222415 | 0.331613 | 0.2728 | 1.59E-06 |  |
| ARPC3 | 0.178223 | 0.212224 | 0.187561 | 0.293034 | 0.240056 | 1.6E-06 |  |
| MIDN | 0.156131 | 0.375918 | 0.337359 | 0.43826 | 0.371033 | 1.65E-06 |  |
| RRM2 | 1.393963 | 0.008299 | 0.003158 | 0.014498 | 0.004821 | 1.74E-06 |  |
| SZRD1 | 0.145631 | 0.462521 | 0.418111 | 0.536798 | 0.461029 | 1.75E-06 |  |
| NFE2L1 | 0.178977 | 0.252282 | 0.222848 | 0.319572 | 0.265769 | 1.81E-06 |  |
| CEBPZ | 0.150378 | 0.363528 | 0.327544 | 0.426711 | 0.364403 | 1.83E-06 |  |
| NDUFB8 | 0.129674 | 0.502093 | 0.458932 | 0.563951 | 0.485938 | 1.83E-06 |  |
| DDX24 | 0.140757 | 0.399943 | 0.362765 | 0.471065 | 0.396947 | 1.84E-06 |  |
| RUVBL1 | 0.160934 | 0.294085 | 0.263043 | 0.356309 | 0.29771 | 1.86E-06 |  |
| EXOSC6 | 0.150347 | 0.336409 | 0.303116 | 0.410001 | 0.345319 | 1.86E-06 |  |
| NT5DC2 | 1.083261 | 0.015031 | 0.007094 | 0.021747 | 0.009642 | 1.88E-06 |  |
| ERGIC2 | 0.17268 | 0.216122 | 0.191742 | 0.281853 | 0.228606 | 1.88E-06 |  |
| AKAP8 | 0.147745 | 0.268918 | 0.242741 | 0.346971 | 0.285858 | 1.99E-06 |  |
| ANAPC5 | 0.136791 | 0.442237 | 0.402232 | 0.505959 | 0.429289 | 2.02E-06 |  |
| ATP6V0A2 | 0.156592 | 0.2479 | 0.222402 | 0.309129 | 0.254118 | 2.08E-06 |  |
| UXS1 | 0.149696 | 0.286092 | 0.257895 | 0.349674 | 0.291884 | 2.12E-06 |  |
| CDK12 | 0.152787 | 0.278692 | 0.250687 | 0.356801 | 0.297107 | 2.16E-06 |  |
| LMBRD1 | 0.15897 | 0.232944 | 0.208639 | 0.303969 | 0.249096 | 2.17E-06 |  |
| SELENOK | 0.148157 | 0.289233 | 0.261004 | 0.359258 | 0.299719 | 2.27E-06 |  |
| MAPK3 | 0.171808 | 0.242256 | 0.215058 | 0.311463 | 0.257332 | 2.29E-06 |  |
| ETNK1 | 0.138707 | 0.379749 | 0.344939 | 0.453987 | 0.380675 | 2.29E-06 |  |
| CELF1 | 0.131635 | 0.48077 | 0.438845 | 0.536921 | 0.462234 | 2.32E-06 |  |
| GRIPAP1 | 0.167927 | 0.255351 | 0.227294 | 0.328173 | 0.271394 | 2.36E-06 |  |
| CAPN1 | 0.15246 | 0.302592 | 0.272247 | 0.374125 | 0.313178 | 2.37E-06 |  |
| MARK3 | 0.151568 | 0.391865 | 0.352785 | 0.472294 | 0.399357 | 2.43E-06 |  |
| GNAI3 | 0.137528 | 0.368808 | 0.335274 | 0.446124 | 0.378666 | 2.46E-06 |  |
| MCPH1 | 0.156905 | 0.250104 | 0.22433 | 0.317607 | 0.263359 | 2.48E-06 |  |
| PHF8 | 0.167249 | 0.233858 | 0.20826 | 0.301143 | 0.248092 | 2.58E-06 |  |
| GLS | 0.165706 | 0.210593 | 0.187742 | 0.286645 | 0.233427 | 2.62E-06 |  |
| SLC25A38 | 0.143318 | 0.320394 | 0.290095 | 0.387025 | 0.325834 | 2.79E-06 |  |
| GALT | 0.150327 | 0.345365 | 0.31119 | 0.416267 | 0.353355 | 2.79E-06 |  |
| ATP6V0C | 0.159028 | 0.261569 | 0.234268 | 0.347709 | 0.288268 | 2.86E-06 |  |
| TRPC4AP | 0.140566 | 0.335209 | 0.304089 | 0.41983 | 0.349739 | 2.89E-06 |  |
| COX14 | 0.148787 | 0.350176 | 0.315862 | 0.420445 | 0.355364 | 2.96E-06 |  |
| WDR83OS | 0.135127 | 0.466211 | 0.424527 | 0.52832 | 0.456609 | 2.98E-06 |  |
| TBCB | 0.142332 | 0.462721 | 0.41925 | 0.497727 | 0.431298 | 3E-06 |  |
| RRP1B | 0.136609 | 0.440414 | 0.400625 | 0.502027 | 0.428084 | 3E-06 |  |
| ACTR3 | 0.133719 | 0.472403 | 0.430585 | 0.541344 | 0.465649 | 3.01E-06 |  |
| USP48 | 0.150302 | 0.340653 | 0.306949 | 0.41209 | 0.350141 | 3.02E-06 |  |
| CMTM6 | 0.141458 | 0.287821 | 0.26094 | 0.366261 | 0.304741 | 3.05E-06 |  |
| TIMM13 | 0.126037 | 0.410096 | 0.375789 | 0.467134 | 0.393732 | 3.15E-06 |  |
| CALCOCO2 | 0.130274 | 0.366675 | 0.335016 | 0.438875 | 0.369225 | 3.22E-06 |  |
| CASP8 | 0.145519 | 0.419405 | 0.379164 | 0.459516 | 0.398152 | 3.27E-06 |  |
| NUP153 | 0.153894 | 0.324474 | 0.291644 | 0.401278 | 0.338891 | 3.39E-06 |  |
| WIPI2 | 0.15912 | 0.365337 | 0.327185 | 0.43654 | 0.373443 | 3.45E-06 |  |
| TIMM10 | 0.153033 | 0.251945 | 0.226588 | 0.298194 | 0.246685 | 3.55E-06 |  |
| BIRC5 | 1.516474 | 0.00618 | 0.00216 | 0.009706 | 0.00221 | 3.58E-06 |  |
| FNBP4 | 0.138377 | 0.467883 | 0.425091 | 0.54159 | 0.464042 | 3.61E-06 |  |
| PCLAF | 1.000419 | 0.009049 | 0.004523 | 0.016955 | 0.006629 | 3.67E-06 |  |
| RNF19A | 0.189537 | 0.309626 | 0.271507 | 0.362084 | 0.309361 | 3.72E-06 |  |
| POLD2 | 0.165285 | 0.221878 | 0.197861 | 0.277798 | 0.227601 | 3.74E-06 |  |
| HNRNPH2 | 0.137896 | 0.349238 | 0.317403 | 0.426588 | 0.359984 | 3.96E-06 |  |
| ZBTB32 | 1.590284 | 0.007401 | 0.002458 | 0.012409 | 0.003817 | 4.05E-06 |  |
| NARS1 | 0.132508 | 0.34544 | 0.315126 | 0.423762 | 0.355163 | 4.05E-06 |  |
| LAMTOR4 | 0.119138 | 0.724905 | 0.667447 | 0.696277 | 0.614102 | 4.06E-06 |  |
| NAPA | 0.12755 | 0.51915 | 0.475222 | 0.569357 | 0.494777 | 4.08E-06 |  |
| TAB2 | 0.111909 | 0.845327 | 0.782234 | 0.754884 | 0.687626 | 4.12E-06 |  |
| HERC4 | 0.1322 | 0.369434 | 0.337086 | 0.439735 | 0.37043 | 4.13E-06 |  |
| PPP6R1 | 0.129634 | 0.56398 | 0.515513 | 0.607323 | 0.532141 | 4.14E-06 |  |
| IRF2 | 0.150524 | 0.394526 | 0.355438 | 0.449318 | 0.385898 | 4.3E-06 |  |
| VPS4A | 0.139653 | 0.305662 | 0.277461 | 0.383708 | 0.319606 | 4.42E-06 |  |
| EDC4 | 0.151609 | 0.291448 | 0.262375 | 0.364173 | 0.30454 | 4.42E-06 |  |
| MEPCE | 0.129293 | 0.427591 | 0.390937 | 0.476594 | 0.408799 | 4.55E-06 |  |
| ITGA6 | 0.113712 | 0.878299 | 0.81173 | 0.735963 | 0.685014 | 4.55E-06 |  |
| POLR2L | 0.134825 | 0.511984 | 0.466305 | 0.566409 | 0.488751 | 4.74E-06 |  |
| PHYKPL | 0.13669 | 0.356563 | 0.324331 | 0.426219 | 0.360587 | 4.86E-06 |  |
| TRA2B | 0.131526 | 0.473726 | 0.432448 | 0.547487 | 0.471073 | 4.86E-06 |  |
| ANKRD17 | 0.129004 | 0.443494 | 0.405559 | 0.517631 | 0.435717 | 4.98E-06 |  |
| YTHDF3 | 0.146172 | 0.316636 | 0.286127 | 0.387025 | 0.326235 | 5.07E-06 |  |
| SMPD4 | 0.147941 | 0.231313 | 0.208769 | 0.30532 | 0.250301 | 5.13E-06 |  |
| CASC3 | 0.134496 | 0.405934 | 0.369801 | 0.478069 | 0.409401 | 5.2E-06 |  |
| BBLN | 0.118457 | 0.648443 | 0.597328 | 0.63165 | 0.560466 | 5.44E-06 |  |
| BRI3 | 0.126889 | 0.391444 | 0.358486 | 0.466028 | 0.392125 | 5.51E-06 |  |
| TCF20 | 0.154789 | 0.258335 | 0.232053 | 0.338494 | 0.281639 | 5.53E-06 |  |
| CCND3 | 0.095661 | 0.992695 | 0.929007 | 0.819511 | 0.763359 | 5.64E-06 |  |
| SUPT5H | 0.135502 | 0.493632 | 0.449379 | 0.552525 | 0.478505 | 5.7E-06 |  |
| TK1 | 1.135377 | 0.007807 | 0.003554 | 0.013884 | 0.004821 | 5.72E-06 |  |
| PLCL1 | 0.140822 | 0.447649 | 0.406018 | 0.458164 | 0.398152 | 5.73E-06 |  |
| PPAN | 0.153373 | 0.336686 | 0.30273 | 0.404227 | 0.345922 | 5.91E-06 |  |
| MPPED2 | 1.190205 | 0.010439 | 0.004575 | 0.012901 | 0.004219 | 6.01E-06 |  |
| AURKAIP1 | 0.149155 | 0.242961 | 0.219097 | 0.313552 | 0.259341 | 6.28E-06 |  |
| PEBP1 | 0.149629 | 0.281248 | 0.25354 | 0.370316 | 0.308156 | 6.53E-06 |  |
| ERO1A | 0.122537 | 0.416167 | 0.382279 | 0.476226 | 0.404982 | 6.67E-06 |  |
| ATP13A1 | 0.137164 | 0.282064 | 0.256482 | 0.351149 | 0.29309 | 6.85E-06 |  |
| RASGRP4 | 1.004895 | 0.011473 | 0.005717 | 0.019536 | 0.008638 | 6.96E-06 |  |
| SCAF11 | 0.141093 | 0.336001 | 0.304696 | 0.423885 | 0.360185 | 6.97E-06 |  |
| RUNX1 | 0.124805 | 0.574759 | 0.527128 | 0.604374 | 0.53174 | 6.98E-06 |  |
| CSK | 0.103246 | 0.960278 | 0.893957 | 0.798993 | 0.731418 | 7.51E-06 |  |
| ID1 | 1.07904 | 0.010011 | 0.004739 | 0.01499 | 0.005625 | 7.57E-06 |  |
| PCNT | 0.141144 | 0.316086 | 0.286627 | 0.386903 | 0.327039 | 7.79E-06 |  |
| PRG4 | 1.40315 | 0.008548 | 0.003232 | 0.012409 | 0.004018 | 7.83E-06 |  |
| TRAPPC6A | 0.130123 | 0.357356 | 0.326535 | 0.415039 | 0.353355 | 8.26E-06 |  |
| DYM | 0.126978 | 0.434629 | 0.398011 | 0.493058 | 0.421053 | 8.35E-06 |  |
| EFTUD2 | 0.158947 | 0.25932 | 0.232267 | 0.334808 | 0.281237 | 8.8E-06 |  |
| FRAT2 | 0.150969 | 0.266144 | 0.239701 | 0.328542 | 0.273805 | 9.34E-06 |  |
| TMEM131L | 0.104307 | 0.802805 | 0.746811 | 0.749109 | 0.67718 | 9.38E-06 |  |
| AUTS2 | 0.133153 | 0.369615 | 0.337028 | 0.400786 | 0.344114 | 9.4E-06 |  |
| PAG1 | 0.107623 | 0.858385 | 0.796681 | 0.767662 | 0.699679 | 9.62E-06 |  |
| CD226 | 0.132845 | 0.297667 | 0.271482 | 0.371422 | 0.31137 | 9.69E-06 |  |
| DCTD | 0.137963 | 0.280542 | 0.254957 | 0.340214 | 0.287063 | 9.81E-06 |  |
| RICTOR | 0.143302 | 0.240926 | 0.218145 | 0.317361 | 0.259743 | 9.99E-06 |  |
| TRIM8 | 0.143836 | 0.316418 | 0.286393 | 0.386043 | 0.326838 | 1.04E-05 |  |
| RNF166 | 0.141341 | 0.215808 | 0.195668 | 0.287505 | 0.236039 | 1.05E-05 |  |
| RO60 | 0.144378 | 0.244098 | 0.220852 | 0.315272 | 0.262957 | 1.06E-05 |  |
| SBF1 | 0.146491 | 0.25366 | 0.229168 | 0.329279 | 0.273805 | 1.09E-05 |  |
| WDR6 | 0.132775 | 0.363319 | 0.331374 | 0.444895 | 0.379068 | 1.09E-05 |  |
| ATP2B4 | 0.13382 | 0.411708 | 0.375237 | 0.454847 | 0.392326 | 1.09E-05 |  |
| MAGED1 | 0.133282 | 0.224616 | 0.204795 | 0.285907 | 0.235235 | 1.1E-05 |  |
| ARF5 | 0.130682 | 0.272115 | 0.248549 | 0.352992 | 0.29309 | 1.12E-05 |  |
| IL11RA | 0.133435 | 0.408212 | 0.37215 | 0.469591 | 0.402772 | 1.12E-05 |  |
| ERCC3 | 0.138063 | 0.23419 | 0.212818 | 0.307409 | 0.253917 | 1.14E-05 |  |
| CWF19L1 | 0.151855 | 0.240697 | 0.21665 | 0.30876 | 0.257131 | 1.15E-05 |  |
| TBC1D2B | 0.142 | 0.340653 | 0.308721 | 0.410247 | 0.352551 | 1.17E-05 |  |
| CCDC141 | 0.138129 | 0.236979 | 0.215342 | 0.299791 | 0.248092 | 1.17E-05 |  |
| TSPO | 0.136581 | 0.22955 | 0.208815 | 0.286645 | 0.235436 | 1.17E-05 |  |
| KDM3B | 0.117909 | 0.354408 | 0.326595 | 0.43224 | 0.364403 | 1.17E-05 |  |
| NFATC2 | 0.129729 | 0.473304 | 0.432601 | 0.528812 | 0.455605 | 1.21E-05 |  |
| CLK4 | 0.152926 | 0.231538 | 0.208251 | 0.294999 | 0.244476 | 1.22E-05 |  |
| CHD1 | 0.136272 | 0.408253 | 0.371456 | 0.481386 | 0.414022 | 1.22E-05 |  |
| MAZ | 0.121427 | 0.259889 | 0.238911 | 0.345251 | 0.285858 | 1.22E-05 |  |
| RBM10 | 0.131925 | 0.323901 | 0.295597 | 0.398329 | 0.337083 | 1.25E-05 |  |
| GOLGA6L6 | 4.305414 | 0.0031 | 0.000157 | 0.005037 | 0.000201 | 1.32E-05 |  |
| PCBP2 | 0.139079 | 0.327081 | 0.297021 | 0.410738 | 0.349337 | 1.36E-05 |  |
| RAB10 | 0.127061 | 0.422181 | 0.386589 | 0.479666 | 0.414825 | 1.36E-05 |  |
| NELFA | 0.128783 | 0.240939 | 0.220364 | 0.312201 | 0.256328 | 1.43E-05 |  |
| PSMB10 | 0.117678 | 0.602376 | 0.555191 | 0.600565 | 0.535757 | 1.43E-05 |  |
| METTL27 | 1.053516 | 0.011992 | 0.005777 | 0.017324 | 0.007433 | 1.44E-05 |  |
| BCL2L13 | 0.117087 | 0.317282 | 0.292549 | 0.362452 | 0.304339 | 1.46E-05 |  |
| SLC25A39 | 0.124385 | 0.441413 | 0.40495 | 0.49785 | 0.431097 | 1.54E-05 |  |
| INTS8 | 0.123996 | 0.350103 | 0.321269 | 0.429905 | 0.362595 | 1.6E-05 |  |
| RXRB | 0.134444 | 0.245218 | 0.223399 | 0.320064 | 0.264966 | 1.61E-05 |  |
| HBP1 | 0.116821 | 0.619813 | 0.571602 | 0.649343 | 0.571113 | 1.71E-05 |  |
| LRCH3 | 0.121781 | 0.257308 | 0.23648 | 0.334685 | 0.277019 | 1.73E-05 |  |
| IP6K1 | 0.132408 | 0.260452 | 0.237612 | 0.323381 | 0.271796 | 1.75E-05 |  |
| DELE1 | 0.119638 | 0.233031 | 0.214486 | 0.300283 | 0.248092 | 1.8E-05 |  |
| GZMM | 0.11071 | 0.614555 | 0.569159 | 0.619732 | 0.545199 | 1.82E-05 |  |
| TPP2 | 0.119713 | 0.474705 | 0.436905 | 0.528812 | 0.462435 | 1.83E-05 |  |
| HM13 | 0.125696 | 0.384624 | 0.352531 | 0.459762 | 0.392326 | 1.83E-05 |  |
| GSTK1 | 0.09997 | 0.924865 | 0.862948 | 0.775648 | 0.711732 | 1.87E-05 |  |
| METTL26 | 0.11924 | 0.412702 | 0.379963 | 0.464431 | 0.394737 | 1.89E-05 |  |
| DEPP1 | 1.495116 | 0.008504 | 0.003017 | 0.011304 | 0.003616 | 1.89E-05 |  |
| WDR43 | 0.160658 | 0.288968 | 0.258516 | 0.361347 | 0.30916 | 1.92E-05 |  |
| MGAT5B | 1.301886 | 0.011238 | 0.004558 | 0.014744 | 0.005826 | 1.93E-05 |  |
| RAB22A | 0.121676 | 0.265805 | 0.244307 | 0.340582 | 0.28405 | 1.94E-05 |  |
| NUCB1 | 0.112289 | 0.637339 | 0.589614 | 0.637548 | 0.567095 | 1.98E-05 |  |
| BIRC6 | 0.115956 | 0.636436 | 0.587284 | 0.662735 | 0.585577 | 2.06E-05 |  |
| NT5C2 | 0.12019 | 0.287392 | 0.26442 | 0.363558 | 0.302732 | 2.06E-05 |  |
| MAP4K1 | 0.130017 | 0.328191 | 0.299908 | 0.388623 | 0.330454 | 2.08E-05 |  |
| PSMG2 | 0.124699 | 0.314286 | 0.288262 | 0.390957 | 0.33186 | 2.12E-05 |  |
| NRN1 | 2.306542 | 0.006855 | 0.001386 | 0.008109 | 0.001808 | 2.16E-05 |  |
| CRTC2 | 0.138584 | 0.259678 | 0.235894 | 0.338002 | 0.283447 | 2.16E-05 |  |
| PHF14 | 0.123649 | 0.32229 | 0.295818 | 0.397223 | 0.334271 | 2.22E-05 |  |
| MALT1 | 0.116965 | 0.556117 | 0.512809 | 0.594668 | 0.517276 | 2.26E-05 |  |
| TMEM134 | 0.144612 | 0.272406 | 0.246425 | 0.331245 | 0.280032 | 2.37E-05 |  |
| TRPM7 | 0.116053 | 0.292384 | 0.269785 | 0.375722 | 0.315187 | 2.37E-05 |  |
| HERC1 | 0.120191 | 0.519591 | 0.478058 | 0.582013 | 0.510446 | 2.39E-05 |  |
| BAG1 | 0.124646 | 0.244321 | 0.224098 | 0.306303 | 0.254922 | 2.4E-05 |  |
| NMI | 0.112176 | 0.351407 | 0.325118 | 0.417373 | 0.354158 | 2.52E-05 |  |
| CYBA | 0.097497 | 0.950392 | 0.888287 | 0.788058 | 0.72037 | 2.63E-05 |  |
| NDUFA9 | 0.140049 | 0.281296 | 0.255273 | 0.35078 | 0.29771 | 2.68E-05 |  |
| NUP160 | 0.124064 | 0.314847 | 0.288903 | 0.385182 | 0.324829 | 2.68E-05 |  |
| SECISBP2 | 0.140006 | 0.318799 | 0.289315 | 0.392554 | 0.335878 | 2.73E-05 |  |
| MTCH1 | 0.125685 | 0.371124 | 0.34016 | 0.443666 | 0.381679 | 2.76E-05 |  |
| CHMP4B | 0.132377 | 0.362543 | 0.330758 | 0.442929 | 0.380474 | 2.84E-05 |  |
| BPNT2 | 0.123525 | 0.255061 | 0.234131 | 0.327682 | 0.274608 | 2.88E-05 |  |
| FYTTD1 | 0.133153 | 0.261662 | 0.238593 | 0.340091 | 0.285456 | 2.92E-05 |  |
| NDUFS1 | 0.139011 | 0.266094 | 0.241651 | 0.338125 | 0.284452 | 2.93E-05 |  |
| ARID1A | 0.126422 | 0.346778 | 0.317684 | 0.431257 | 0.36601 | 2.94E-05 |  |
| GTPBP4 | 0.11885 | 0.233507 | 0.215042 | 0.304337 | 0.253515 | 2.96E-05 |  |
| GOLT1B | 0.118239 | 0.298077 | 0.274621 | 0.364418 | 0.307754 | 2.96E-05 |  |
| STUB1 | 0.121383 | 0.34371 | 0.315975 | 0.41725 | 0.355163 | 2.97E-05 |  |
| DOT1L | 0.129696 | 0.254194 | 0.23234 | 0.32461 | 0.272599 | 2.98E-05 |  |
| AJAP1 | 2.830126 | 0.004753 | 0.000668 | 0.00602 | 0.000804 | 3E-05 |  |
| SERBP1 | 0.123363 | 0.551266 | 0.506087 | 0.613835 | 0.542989 | 3.05E-05 |  |
| ITGA4 | 0.113274 | 0.847396 | 0.783407 | 0.684974 | 0.632985 | 3.08E-05 |  |
| DENND2D | 0.112555 | 0.689078 | 0.637362 | 0.644182 | 0.582563 | 3.09E-05 |  |
| GSPT1 | 0.140764 | 0.223142 | 0.202399 | 0.304829 | 0.253515 | 3.13E-05 |  |
| BZW2 | 0.115676 | 0.356631 | 0.329152 | 0.41037 | 0.349337 | 3.21E-05 |  |
| TOP1 | 0.126795 | 0.3349 | 0.306722 | 0.410001 | 0.348534 | 3.23E-05 |  |
| SRSF9 | 0.123169 | 0.260739 | 0.239402 | 0.342302 | 0.28626 | 3.36E-05 |  |
| VPS4B | 0.123139 | 0.282521 | 0.259407 | 0.357538 | 0.302129 | 3.38E-05 |  |
| TPP1 | 0.120334 | 0.39523 | 0.363602 | 0.453004 | 0.391523 | 3.47E-05 |  |
| METTL23 | 0.107305 | 0.318015 | 0.29522 | 0.373265 | 0.316995 | 3.51E-05 |  |
| CBFB | 0.116528 | 0.476873 | 0.43987 | 0.532252 | 0.461631 | 3.53E-05 |  |
| BLOC1S1 | 0.119305 | 0.298582 | 0.274884 | 0.369824 | 0.311973 | 3.55E-05 |  |
| FAM89B | 0.119163 | 0.470485 | 0.433186 | 0.505959 | 0.443351 | 3.57E-05 |  |
| ZNF24 | 0.122177 | 0.391367 | 0.359588 | 0.45669 | 0.39313 | 3.62E-05 |  |
| MLF2 | 0.124827 | 0.35994 | 0.330106 | 0.435926 | 0.374046 | 3.64E-05 |  |
| NINJ1 | 0.115438 | 0.272712 | 0.251741 | 0.330507 | 0.278023 | 3.71E-05 |  |
| PKP2 | 1.02835 | 0.016537 | 0.008107 | 0.02359 | 0.012455 | 3.79E-05 |  |
| CRBN | 0.130328 | 0.297168 | 0.2715 | 0.373756 | 0.317597 | 3.94E-05 |  |
| TERF2 | 0.124067 | 0.292892 | 0.268757 | 0.360732 | 0.303937 | 3.98E-05 |  |
| NPLOC4 | 0.116492 | 0.556367 | 0.513208 | 0.590736 | 0.52049 | 4.05E-05 |  |
| PSMD13 | 0.122036 | 0.375385 | 0.344938 | 0.455338 | 0.392125 | 4.05E-05 |  |
| ABCF1 | 0.135342 | 0.270959 | 0.246696 | 0.34906 | 0.294697 | 4.07E-05 |  |
| MYDGF | 0.117152 | 0.360378 | 0.33227 | 0.418356 | 0.35898 | 4.16E-05 |  |
| KIAA0319L | 0.128399 | 0.322272 | 0.294829 | 0.389606 | 0.335074 | 4.21E-05 |  |
| MMP24OS | 0.132341 | 0.297142 | 0.271097 | 0.354589 | 0.302933 | 4.24E-05 |  |
| CFAP97 | 0.122346 | 0.28383 | 0.260752 | 0.34992 | 0.298313 | 4.27E-05 |  |
| MARCHF5 | 0.121153 | 0.29112 | 0.267671 | 0.356186 | 0.300522 | 4.38E-05 |  |
| ATIC | 0.119458 | 0.321276 | 0.295745 | 0.390097 | 0.330052 | 4.45E-05 |  |
| KCNK5 | 1.262938 | 0.007492 | 0.003122 | 0.011795 | 0.004219 | 4.57E-05 |  |
| TP53I3 | 1.252745 | 0.007468 | 0.003134 | 0.012409 | 0.00462 | 4.58E-05 |  |
| PRKDC | 0.127089 | 0.319139 | 0.292228 | 0.3971 | 0.339695 | 4.63E-05 |  |
| PRRG2 | 1.078092 | 0.00862 | 0.004083 | 0.013024 | 0.005022 | 4.67E-05 |  |
| PPP1CB | 0.112939 | 0.630336 | 0.582873 | 0.666667 | 0.59321 | 4.7E-05 |  |
| GPX4 | 0.110221 | 0.577241 | 0.534783 | 0.611132 | 0.537967 | 4.71E-05 |  |
| PIP5K1A | 0.128792 | 0.269899 | 0.246848 | 0.348569 | 0.29329 | 4.73E-05 |  |
| IKZF5 | 0.116191 | 0.23028 | 0.212461 | 0.306917 | 0.253917 | 4.75E-05 |  |
| RGS14 | 0.11684 | 0.374414 | 0.345286 | 0.435189 | 0.373041 | 5.09E-05 |  |
| HCFC1 | 0.121957 | 0.33437 | 0.307266 | 0.406192 | 0.347529 | 5.23E-05 |  |
| MED13L | 0.106671 | 0.436054 | 0.404976 | 0.509276 | 0.43853 | 5.31E-05 |  |
| DDX27 | 0.107385 | 0.331787 | 0.307988 | 0.407175 | 0.346926 | 5.38E-05 |  |
| EIF4A3 | 0.121495 | 0.317641 | 0.291986 | 0.386903 | 0.328244 | 5.43E-05 |  |
| TBC1D14 | 0.124927 | 0.27 | 0.247604 | 0.342057 | 0.288067 | 5.45E-05 |  |
| NOP14 | 0.131493 | 0.310113 | 0.283098 | 0.379776 | 0.324227 | 5.5E-05 |  |
| SF3B3 | 0.12082 | 0.290655 | 0.267305 | 0.367736 | 0.311571 | 5.61E-05 |  |
| ZZZ3 | 0.123857 | 0.272288 | 0.249887 | 0.338002 | 0.285657 | 5.9E-05 |  |
| DHX38 | 0.134333 | 0.24378 | 0.222105 | 0.318835 | 0.268783 | 5.92E-05 |  |
| GNL1 | 0.109914 | 0.335268 | 0.310674 | 0.395503 | 0.336882 | 6.15E-05 |  |
| AIM2 | 1.429071 | 0.004758 | 0.001767 | 0.009338 | 0.002812 | 6.38E-05 |  |
| YIPF4 | 0.104099 | 0.315345 | 0.293393 | 0.390097 | 0.32965 | 6.39E-05 |  |
| PEDS1 | 0.102643 | 0.325825 | 0.30345 | 0.3971 | 0.338489 | 6.49E-05 |  |
| RBM27 | 0.126051 | 0.309067 | 0.283209 | 0.389606 | 0.331258 | 6.51E-05 |  |
| SDF4 | 0.12606 | 0.325983 | 0.298708 | 0.399558 | 0.341101 | 6.71E-05 |  |
| GLUD1 | 0.121072 | 0.286252 | 0.26321 | 0.366138 | 0.309562 | 6.79E-05 |  |
| IFNAR2 | 0.114186 | 0.392916 | 0.363017 | 0.450055 | 0.38871 | 7.23E-05 |  |
| SUPT6H | 0.109118 | 0.423555 | 0.392702 | 0.500061 | 0.426677 | 7.28E-05 |  |
| MAPK14 | 0.11987 | 0.289717 | 0.266618 | 0.357415 | 0.30454 | 7.58E-05 |  |
| NAA60 | 0.12958 | 0.286217 | 0.261631 | 0.358644 | 0.305544 | 7.66E-05 |  |
| ST8SIA4 | 0.120503 | 0.31744 | 0.292003 | 0.377688 | 0.323624 | 7.72E-05 |  |
| USP33 | 0.127003 | 0.288837 | 0.264497 | 0.368104 | 0.311973 | 7.74E-05 |  |
| IRAK4 | 0.122525 | 0.276688 | 0.254159 | 0.350534 | 0.294496 | 7.76E-05 |  |
| TCOF1 | 0.132526 | 0.314009 | 0.286449 | 0.379776 | 0.324427 | 7.79E-05 |  |
| ZZEF1 | 0.123431 | 0.396096 | 0.363617 | 0.475857 | 0.412616 | 7.94E-05 |  |
| TAPBP | 0.08602 | 1.150068 | 1.0835 | 0.873695 | 0.805946 | 7.96E-05 |  |
| SSRP1 | 0.122061 | 0.321645 | 0.295551 | 0.396855 | 0.341101 | 7.99E-05 |  |
| PIP4P1 | 0.115508 | 0.280409 | 0.258833 | 0.339108 | 0.28646 | 8.07E-05 |  |
| CDC14A | 0.106942 | 0.571163 | 0.530356 | 0.591473 | 0.520088 | 8.08E-05 |  |
| COPB1 | 0.114112 | 0.282476 | 0.260994 | 0.363558 | 0.306951 | 8.11E-05 |  |
| GRINA | 0.117817 | 0.290088 | 0.26734 | 0.360118 | 0.304942 | 8.15E-05 |  |
| PXN | 0.109592 | 0.453001 | 0.419864 | 0.510628 | 0.444757 | 8.2E-05 |  |
| STXBP3 | 0.1049 | 0.293431 | 0.272853 | 0.362452 | 0.307151 | 8.84E-05 |  |
| UBE2I | 0.110702 | 0.443316 | 0.410571 | 0.500799 | 0.436922 | 8.94E-05 |  |
| TMEM87A | 0.110582 | 0.253597 | 0.234885 | 0.329279 | 0.276014 | 9.03E-05 |  |
| DYNLRB1 | 0.109611 | 0.595146 | 0.551604 | 0.600934 | 0.539373 | 9.06E-05 |  |
| TRMT1 | 0.116821 | 0.326607 | 0.301203 | 0.392063 | 0.334673 | 9.1E-05 |  |
| AGAP2 | 0.136437 | 0.278771 | 0.253616 | 0.347586 | 0.297308 | 9.15E-05 |  |
| RALBP1 | 0.108894 | 0.272506 | 0.252694 | 0.348446 | 0.295098 | 9.72E-05 |  |
| STX5 | 0.111396 | 0.284911 | 0.26374 | 0.358766 | 0.303536 | 0.000103 |  |
| CLIP1 | 0.119282 | 0.278357 | 0.256269 | 0.355695 | 0.302129 | 0.000104 |  |
| GTF2F1 | 0.114887 | 0.271976 | 0.251158 | 0.3439 | 0.291483 | 0.000105 |  |
| FOXK1 | 0.110843 | 0.388606 | 0.359867 | 0.459516 | 0.395339 | 0.000105 |  |
| HTR6 | 3.013407 | 0.003494 | 0.000433 | 0.005037 | 0.000603 | 0.000107 |  |
| TIA1 | 0.109362 | 0.468491 | 0.43429 | 0.521071 | 0.455605 | 0.000107 |  |
| ANKRD11 | 0.105269 | 0.541343 | 0.50325 | 0.580784 | 0.507433 | 0.00011 |  |
| ARFGAP2 | 0.113163 | 0.247599 | 0.22892 | 0.326576 | 0.276416 | 0.000113 |  |
| PPP3R1 | 0.103843 | 0.486633 | 0.452837 | 0.552156 | 0.483126 | 0.000113 |  |
| ZRANB2 | 0.115014 | 0.339773 | 0.313737 | 0.410001 | 0.353154 | 0.000115 |  |
| SRSF4 | 0.108914 | 0.547577 | 0.50776 | 0.593193 | 0.527119 | 0.000123 |  |
| EIF4G1 | 0.111933 | 0.242098 | 0.224025 | 0.327927 | 0.274608 | 0.000124 |  |
| SNX17 | 0.106166 | 0.54962 | 0.510626 | 0.582627 | 0.515066 | 0.000124 |  |
| DCTN1 | 0.109318 | 0.382943 | 0.354998 | 0.460499 | 0.399156 | 0.000127 |  |
| LY6E | 0.09247 | 0.810038 | 0.759747 | 0.70242 | 0.632784 | 0.000128 |  |
| PCGF5 | 0.102807 | 0.379621 | 0.35351 | 0.427448 | 0.369225 | 0.000128 |  |
| THRAP3 | 0.109703 | 0.487608 | 0.451904 | 0.549945 | 0.485135 | 0.000131 |  |
| DTHD1 | 1.721959 | 0.003669 | 0.001112 | 0.006143 | 0.001205 | 0.000134 |  |
| TMEM106B | 0.115844 | 0.388118 | 0.358172 | 0.448581 | 0.38871 | 0.00014 |  |
| PSMB4 | 0.11713 | 0.280731 | 0.25884 | 0.364418 | 0.312575 | 0.000142 |  |
| ORMDL1 | 0.10204 | 0.336278 | 0.313315 | 0.411476 | 0.353957 | 0.000149 |  |
| CCNB2 | 1.461288 | 0.010183 | 0.003698 | 0.013515 | 0.005826 | 0.00015 |  |
| ZMAT2 | 0.104979 | 0.490511 | 0.456087 | 0.536798 | 0.464644 | 0.00015 |  |
| ELMO1 | 0.102538 | 0.474017 | 0.441497 | 0.514191 | 0.447971 | 0.000151 |  |
| STAT6 | 0.101932 | 0.700459 | 0.652677 | 0.691854 | 0.623343 | 0.000154 |  |
| DDX54 | 0.101108 | 0.312429 | 0.291283 | 0.385674 | 0.325633 | 0.000154 |  |
| KIF26B | 1.621029 | 0.005812 | 0.00189 | 0.007863 | 0.00221 | 0.000158 |  |
| CSNK1A1 | 0.129128 | 0.245955 | 0.224897 | 0.331613 | 0.281237 | 0.000159 |  |
| HELZ | 0.103762 | 0.39527 | 0.367839 | 0.467134 | 0.400161 | 0.00016 |  |
| DOCK8 | 0.110788 | 0.511062 | 0.473286 | 0.567023 | 0.499598 | 0.00016 |  |
| IL1R1 | 1.147567 | 0.00685 | 0.003092 | 0.009829 | 0.003415 | 0.000166 |  |
| KIF2C | 1.48471 | 0.006686 | 0.002389 | 0.010444 | 0.003817 | 0.000167 |  |
| MORC3 | 0.096501 | 0.7115 | 0.665465 | 0.706229 | 0.63399 | 0.000172 |  |
| ANKRD12 | 0.115503 | 0.292217 | 0.269734 | 0.368596 | 0.314383 | 0.000173 |  |
| POLDIP2 | 0.111408 | 0.316118 | 0.292625 | 0.383954 | 0.330454 | 0.000175 |  |
| SYNGR2 | 0.093209 | 0.438941 | 0.411479 | 0.492935 | 0.427079 | 0.000177 |  |
| HCLS1 | 0.08376 | 1.027492 | 0.969537 | 0.825163 | 0.764966 | 0.00018 |  |
| TBL1XR1 | 0.099062 | 0.542242 | 0.506259 | 0.583733 | 0.51808 | 0.000182 |  |
| YTHDF2 | 0.114898 | 0.384383 | 0.354958 | 0.460253 | 0.39996 | 0.000183 |  |
| CELSR1 | 1.291453 | 0.007321 | 0.002991 | 0.010075 | 0.003616 | 0.000188 |  |
| PTPN6 | 0.109181 | 0.430689 | 0.399298 | 0.474628 | 0.420048 | 0.000191 |  |
| RAB5A | 0.092361 | 0.350543 | 0.328804 | 0.424499 | 0.363399 | 0.000191 |  |
| AP1G2 | 0.120137 | 0.371269 | 0.341605 | 0.446001 | 0.386902 | 0.000198 |  |
| ARHGDIA | 0.113607 | 0.462629 | 0.427596 | 0.535324 | 0.469466 | 0.000201 |  |
| EXOC7 | 0.101597 | 0.468435 | 0.436581 | 0.525863 | 0.454198 | 0.000204 |  |
| SNRNP40 | 0.110347 | 0.293424 | 0.271818 | 0.351026 | 0.300522 | 0.000206 |  |
| ITPR2 | 0.098001 | 0.428324 | 0.400195 | 0.488144 | 0.427883 | 0.000231 |  |
| PHF20L1 | 0.089641 | 0.494561 | 0.464767 | 0.551542 | 0.481117 | 0.000231 |  |
| EMC10 | 0.096768 | 0.643818 | 0.60205 | 0.651063 | 0.569305 | 0.000231 |  |
| AURKB | 1.078901 | 0.005347 | 0.002531 | 0.009952 | 0.003616 | 0.000239 |  |
| MTA3 | 0.1032 | 0.310267 | 0.288848 | 0.372527 | 0.319606 | 0.000244 |  |
| SP110 | 0.096392 | 0.683037 | 0.638892 | 0.672564 | 0.606067 | 0.000245 |  |
| TNFRSF25 | 0.095956 | 0.573957 | 0.537024 | 0.608551 | 0.534351 | 0.000246 |  |
| RGS19 | 0.10327 | 0.348485 | 0.324412 | 0.399926 | 0.346525 | 0.00025 |  |
| SIPA1L2 | 1.07441 | 0.00926 | 0.004397 | 0.013515 | 0.006027 | 0.000252 |  |
| UBE2O | 0.097761 | 0.281629 | 0.263178 | 0.348077 | 0.295098 | 0.000253 |  |
| STRAP | 0.103915 | 0.512198 | 0.476603 | 0.574886 | 0.50904 | 0.000254 |  |
| PRPF40A | 0.113411 | 0.281991 | 0.260672 | 0.366507 | 0.315187 | 0.000259 |  |
| PNN | 0.108032 | 0.461948 | 0.428619 | 0.530041 | 0.464644 | 0.000259 |  |
| STAT5A | 0.106902 | 0.40779 | 0.378665 | 0.46357 | 0.403375 | 0.000261 |  |
| ARIH2 | 0.090809 | 0.287301 | 0.269774 | 0.362084 | 0.309361 | 0.000264 |  |
| TAF1D | 0.091682 | 0.447416 | 0.419868 | 0.509399 | 0.438931 | 0.000272 |  |
| METTL3 | 0.103034 | 0.291876 | 0.271758 | 0.364173 | 0.310366 | 0.000276 |  |
| DNMT1 | 0.109389 | 0.380226 | 0.352462 | 0.448458 | 0.392728 | 0.000277 |  |
| AFF4 | 0.106828 | 0.289422 | 0.268765 | 0.363558 | 0.312174 | 0.000277 |  |
| GOLGA7 | 0.104963 | 0.46713 | 0.43435 | 0.521809 | 0.457413 | 0.000279 |  |
| PRKAR1A | 0.101148 | 0.466969 | 0.435351 | 0.528689 | 0.456609 | 0.00028 |  |
| CCNE2 | 1.252781 | 0.006346 | 0.002663 | 0.009829 | 0.003616 | 0.000292 |  |
| AGPS | 0.108565 | 0.277878 | 0.257734 | 0.355449 | 0.303937 | 0.000296 |  |
| TXNDC11 | 0.095078 | 0.37325 | 0.349445 | 0.43654 | 0.375854 | 0.000297 |  |
| AEBP2 | 0.100768 | 0.567146 | 0.528885 | 0.604988 | 0.535356 | 0.000298 |  |
| ANKRD10 | 0.096133 | 0.483987 | 0.452788 | 0.534341 | 0.467658 | 0.000308 |  |
| ZFC3H1 | 0.114985 | 0.403268 | 0.372375 | 0.476594 | 0.416633 | 0.000309 |  |
| PTGES2 | 0.112326 | 0.297168 | 0.274909 | 0.363804 | 0.312575 | 0.000312 |  |
| CSF1 | 1.100892 | 0.008581 | 0.004001 | 0.012778 | 0.005625 | 0.000323 |  |
| NDUFB11 | 0.105038 | 0.472656 | 0.439467 | 0.530901 | 0.467256 | 0.000324 |  |
| IFNG | 1.226513 | 0.007545 | 0.003224 | 0.010689 | 0.004219 | 0.000325 |  |
| RNF187 | 0.094771 | 0.32516 | 0.304486 | 0.401155 | 0.343712 | 0.000326 |  |
| RB1CC1 | 0.103019 | 0.404467 | 0.376592 | 0.477577 | 0.415026 | 0.000331 |  |
| MDM2 | 0.108711 | 0.326363 | 0.302674 | 0.398329 | 0.344717 | 0.00034 |  |
| SEMA4D | 0.097891 | 0.690052 | 0.644783 | 0.69984 | 0.619928 | 0.000344 |  |
| CHIC2 | 0.090551 | 0.430277 | 0.404101 | 0.498096 | 0.430695 | 0.000347 |  |
| WWC1 | 2.490396 | 0.003203 | 0.00057 | 0.004915 | 0.000804 | 0.000356 |  |
| OTUD5 | 0.099367 | 0.369077 | 0.344512 | 0.444649 | 0.385496 | 0.000359 |  |
| LONP2 | 0.112748 | 0.305262 | 0.282314 | 0.384814 | 0.33186 | 0.000369 |  |
| AKIRIN2 | 0.100885 | 0.403791 | 0.376519 | 0.473154 | 0.410607 | 0.000373 |  |
| ARRDC2 | 0.092534 | 0.525583 | 0.49293 | 0.553631 | 0.492969 | 0.000382 |  |
| MAGEH1 | 0.089036 | 0.337367 | 0.317176 | 0.377196 | 0.325633 | 0.000384 |  |
| ANXA7 | 0.113818 | 0.266437 | 0.246225 | 0.34648 | 0.295701 | 0.000391 |  |
| ACSF3 | 0.103096 | 0.347216 | 0.323269 | 0.410984 | 0.356167 | 0.0004 |  |
| GAK | 0.096132 | 0.281959 | 0.263783 | 0.366261 | 0.312977 | 0.000401 |  |
| DUS1L | 0.101306 | 0.510278 | 0.475675 | 0.562231 | 0.495179 | 0.000406 |  |
| FABP2 | 65535 | 0.002023 | 0 | 0.003072 | 0 | 0.000408 |  |
| ZXDC | 0.103944 | 0.298133 | 0.277409 | 0.374247 | 0.322017 | 0.00041 |  |
| RASAL1 | 1.845743 | 0.005107 | 0.001421 | 0.007372 | 0.00221 | 0.000412 |  |
| PTPN20 | 1.662009 | 0.005056 | 0.001598 | 0.007372 | 0.00221 | 0.000415 |  |
| MYBBP1A | 0.094166 | 0.351967 | 0.329728 | 0.412704 | 0.357975 | 0.000423 |  |
| USP3 | 0.101035 | 0.609226 | 0.56802 | 0.640742 | 0.566091 | 0.000423 |  |
| GHITM | 0.095716 | 0.425574 | 0.398255 | 0.498096 | 0.436922 | 0.00043 |  |
| IFRD1 | 0.097095 | 0.300149 | 0.280613 | 0.365647 | 0.312174 | 0.00044 |  |
| SYMPK | 0.105366 | 0.348679 | 0.324121 | 0.423394 | 0.364805 | 0.000444 |  |
| RBM19 | 0.092308 | 0.284411 | 0.266783 | 0.355203 | 0.304339 | 0.000451 |  |
| ASXL1 | 0.077205 | 0.954548 | 0.904809 | 0.822951 | 0.757935 | 0.000458 |  |
| STK11 | 0.093929 | 0.330576 | 0.309739 | 0.402998 | 0.347328 | 0.000461 |  |
| CCDC122 | 1.796994 | 0.004401 | 0.001266 | 0.006635 | 0.001808 | 0.000462 |  |
| MOCS1 | 2.72599 | 0.003461 | 0.000523 | 0.004792 | 0.000804 | 0.000463 |  |
| IWS1 | 0.093979 | 0.292597 | 0.274144 | 0.367613 | 0.314785 | 0.000487 |  |
| TRABD | 0.094387 | 0.38604 | 0.361593 | 0.454601 | 0.396143 | 0.000504 |  |
| CLNS1A | 0.105249 | 0.271236 | 0.252153 | 0.351395 | 0.300723 | 0.000525 |  |
| MYO9B | 0.107794 | 0.396528 | 0.36798 | 0.475857 | 0.416031 | 0.000528 |  |
| LGALS4 | 1.11977 | 0.01096 | 0.005044 | 0.014129 | 0.00683 | 0.000553 |  |
| UBTF | 0.095723 | 0.382381 | 0.357833 | 0.449441 | 0.390518 | 0.000555 |  |
| DHX30 | 0.093052 | 0.311628 | 0.292163 | 0.383708 | 0.331659 | 0.000557 |  |
| CEP55 | 1.892891 | 0.005037 | 0.001356 | 0.00688 | 0.002009 | 0.000557 |  |
| SLMAP | 0.104362 | 0.323183 | 0.30063 | 0.398943 | 0.34552 | 0.000558 |  |
| GNB1 | 0.103782 | 0.569672 | 0.530132 | 0.614818 | 0.544797 | 0.000562 |  |
| NCBP2 | 0.090312 | 0.390347 | 0.36666 | 0.449318 | 0.392527 | 0.000565 |  |
| SCAF8 | 0.090462 | 0.425499 | 0.399638 | 0.503256 | 0.437525 | 0.000574 |  |
| CCNT1 | 0.103403 | 0.336801 | 0.313506 | 0.410616 | 0.356971 | 0.000577 |  |
| ARHGAP8 | 1.86269 | 0.004112 | 0.001131 | 0.006512 | 0.001808 | 0.000588 |  |
| ILDR2 | 1.065321 | 0.00615 | 0.002939 | 0.010321 | 0.004219 | 0.000614 |  |
| SNX1 | 0.091124 | 0.615829 | 0.578135 | 0.630544 | 0.563078 | 0.000619 |  |
| CTSS | 0.068495 | 0.34752 | 0.331406 | 0.455461 | 0.377461 | 0.000621 |  |
| NLRC3 | 0.098709 | 0.396275 | 0.370068 | 0.461482 | 0.402571 | 0.000621 |  |
| PPHLN1 | 0.097915 | 0.426287 | 0.398315 | 0.494041 | 0.43411 | 0.000622 |  |
| UBXN4 | 0.092887 | 0.426242 | 0.399663 | 0.489372 | 0.427481 | 0.000648 |  |
| RABGGTB | 0.089432 | 0.387194 | 0.363921 | 0.451161 | 0.391121 | 0.000649 |  |
| MCRS1 | 0.085756 | 0.345883 | 0.325922 | 0.396732 | 0.342909 | 0.000654 |  |
| RER1 | 0.10094 | 0.414628 | 0.38661 | 0.475243 | 0.416432 | 0.000659 |  |
| ICA1 | 1.243115 | 0.00446 | 0.001884 | 0.008109 | 0.002812 | 0.000666 |  |
| ZC3H15 | 0.09441 | 0.433506 | 0.406046 | 0.508785 | 0.439132 | 0.00067 |  |
| SH3BGRL3 | 0.081096 | 1.037964 | 0.981228 | 0.814351 | 0.750703 | 0.000698 |  |
| ZER1 | 0.096317 | 0.344153 | 0.321927 | 0.408035 | 0.35456 | 0.000727 |  |
| STARD3 | 0.093522 | 0.352456 | 0.330333 | 0.422533 | 0.366814 | 0.000757 |  |
| VPS26B | 0.085652 | 0.41032 | 0.386669 | 0.472048 | 0.412816 | 0.000772 |  |
| TOMM20 | 0.097011 | 0.623729 | 0.583167 | 0.658558 | 0.587786 | 0.000776 |  |
| ARAP1 | 0.085363 | 0.278832 | 0.262812 | 0.359872 | 0.308357 | 0.000791 |  |
| PCED1A | 0.082495 | 0.396497 | 0.374461 | 0.450547 | 0.392728 | 0.000794 |  |
| MAT2B | 0.092721 | 0.586655 | 0.550137 | 0.618258 | 0.550824 | 0.00081 |  |
| AGFG1 | 0.088434 | 0.366041 | 0.344277 | 0.443298 | 0.382885 | 0.000811 |  |
| UBE2H | 0.097402 | 0.321766 | 0.300759 | 0.400049 | 0.34773 | 0.000823 |  |
| UBAP2L | 0.092819 | 0.482243 | 0.452194 | 0.54761 | 0.478907 | 0.000825 |  |
| VPS39 | 0.089485 | 0.354159 | 0.332859 | 0.427079 | 0.371033 | 0.000828 |  |
| MXD4 | 0.104726 | 0.485035 | 0.451074 | 0.507433 | 0.453797 | 0.000852 |  |
| SFPQ | 0.094418 | 0.286018 | 0.267899 | 0.368719 | 0.315589 | 0.000863 |  |
| TIAL1 | 0.094912 | 0.615699 | 0.576497 | 0.6475 | 0.576336 | 0.000877 |  |
| UBE2G2 | 0.09284 | 0.361752 | 0.339206 | 0.438014 | 0.384693 | 0.000918 |  |
| PA2G4 | 0.080317 | 0.347763 | 0.328932 | 0.425359 | 0.366814 | 0.000939 |  |
| PLPP2 | 1.365491 | 0.005947 | 0.002308 | 0.008846 | 0.003415 | 0.000945 |  |
| CPM | 1.235261 | 0.004894 | 0.002079 | 0.008846 | 0.003415 | 0.000948 |  |
| TP53 | 0.092197 | 0.34097 | 0.319861 | 0.40865 | 0.354962 | 0.000972 |  |
| KMT2B | 0.093725 | 0.334441 | 0.313405 | 0.407175 | 0.35677 | 0.000973 |  |
| SKA1 | 2.675775 | 0.002763 | 0.000432 | 0.004423 | 0.000804 | 0.001044 |  |
| CTDNEP1 | 0.092613 | 0.393452 | 0.368988 | 0.475857 | 0.411611 | 0.001054 |  |
| SH3D21 | 1.856178 | 0.003408 | 0.000941 | 0.005529 | 0.001406 | 0.001057 |  |
| PPP2R5C | 0.096592 | 0.593985 | 0.555518 | 0.627473 | 0.567095 | 0.001067 |  |
| THUMPD1 | 0.084244 | 0.447765 | 0.422367 | 0.510136 | 0.441543 | 0.00107 |  |
| ATAD2B | 0.096348 | 0.319062 | 0.29845 | 0.398943 | 0.344315 | 0.001074 |  |
| SLCO4A1 | 1.615581 | 0.004006 | 0.001307 | 0.00688 | 0.00221 | 0.001076 |  |
| SMG5 | 0.074738 | 0.274935 | 0.261054 | 0.353606 | 0.300924 | 0.001085 |  |
| UBE2Q1 | 0.089235 | 0.388024 | 0.364751 | 0.462342 | 0.40237 | 0.001091 |  |
| KHSRP | 0.089181 | 0.290164 | 0.272771 | 0.386165 | 0.330856 | 0.001091 |  |
| NDUFB9 | 0.090492 | 0.639214 | 0.600351 | 0.639759 | 0.581157 | 0.001123 |  |
| GPR34 | 3.037745 | 0.001706 | 0.000208 | 0.003563 | 0.000402 | 0.00116 |  |
| EML4 | 0.090279 | 0.500582 | 0.470218 | 0.546504 | 0.485135 | 0.001166 |  |
| TUBGCP6 | 0.095745 | 0.464897 | 0.435045 | 0.522914 | 0.467256 | 0.001181 |  |
| ADAM12 | 1.169529 | 0.006475 | 0.002879 | 0.008723 | 0.003415 | 0.001193 |  |
| CDK1 | 1.07809 | 0.009779 | 0.004632 | 0.012778 | 0.006227 | 0.001204 |  |
| SELENOF | 0.083763 | 0.702639 | 0.663005 | 0.697752 | 0.628967 | 0.001239 |  |
| MTPN | 0.099906 | 0.284795 | 0.26574 | 0.377073 | 0.324227 | 0.00124 |  |
| RAB5B | 0.090185 | 0.311979 | 0.293074 | 0.389237 | 0.337083 | 0.001242 |  |
| NXF1 | 0.093842 | 0.44554 | 0.417482 | 0.518491 | 0.457413 | 0.001264 |  |
| SPRED3 | 1.301449 | 0.004538 | 0.001841 | 0.007126 | 0.002411 | 0.001264 |  |
| POLR2E | 0.079558 | 0.356973 | 0.33782 | 0.427079 | 0.368823 | 0.001279 |  |
| CRACR2B | 65535 | 0.001265 | 0 | 0.00258 | 0 | 0.001289 |  |
| PHC3 | 0.09194 | 0.494359 | 0.463838 | 0.551542 | 0.48634 | 0.001296 |  |
| RNF144A | 0.101331 | 0.424571 | 0.395773 | 0.465291 | 0.411812 | 0.0013 |  |
| MOAP1 | 0.091139 | 0.646683 | 0.607094 | 0.655977 | 0.589393 | 0.001369 |  |
| HLF | 1.077265 | 0.011129 | 0.005275 | 0.013761 | 0.007031 | 0.001424 |  |
| RAB7A | 0.094411 | 0.270978 | 0.253812 | 0.361838 | 0.310165 | 0.001437 |  |
| SAFB2 | 0.088275 | 0.485911 | 0.457071 | 0.544784 | 0.482523 | 0.001455 |  |
| DGUOK | 0.081363 | 0.345141 | 0.326215 | 0.407667 | 0.356167 | 0.001455 |  |
| PRSS27 | 1.012364 | 0.006169 | 0.003058 | 0.009215 | 0.003817 | 0.001456 |  |
| IRF3 | 0.088784 | 0.516669 | 0.485831 | 0.556702 | 0.494174 | 0.001458 |  |
| PHF10 | 0.08935 | 0.580749 | 0.545873 | 0.606708 | 0.541985 | 0.001465 |  |
| DOCK2 | 0.085326 | 0.747059 | 0.704157 | 0.732031 | 0.66131 | 0.001466 |  |
| PPP1R18 | 0.08472 | 0.709325 | 0.66887 | 0.654134 | 0.596223 | 0.001496 |  |
| GDI1 | 0.097011 | 0.357669 | 0.334409 | 0.429414 | 0.377059 | 0.001548 |  |
| NDUFAB1 | 0.076977 | 0.322668 | 0.305903 | 0.39366 | 0.341101 | 0.001553 |  |
| DENND6A | 0.084743 | 0.350023 | 0.330055 | 0.417865 | 0.362796 | 0.001564 |  |
| EAPP | 0.087765 | 0.355159 | 0.334197 | 0.427448 | 0.377059 | 0.001566 |  |
| ZCCHC18 | 1.613329 | 0.003411 | 0.001115 | 0.00602 | 0.001808 | 0.001568 |  |
| SIN3A | 0.086352 | 0.5637 | 0.53095 | 0.594913 | 0.532141 | 0.00158 |  |
| MMP9 | 1.189401 | 0.00409 | 0.001793 | 0.007003 | 0.002411 | 0.001592 |  |
| RSU1 | 0.080542 | 0.408358 | 0.386185 | 0.470574 | 0.411812 | 0.001642 |  |
| ADAR | 0.082923 | 0.810342 | 0.765079 | 0.759553 | 0.693451 | 0.00167 |  |
| AAK1 | 0.072448 | 1.012876 | 0.963269 | 0.838064 | 0.783246 | 0.001678 |  |
| RABEP1 | 0.084949 | 0.341385 | 0.321863 | 0.420322 | 0.364604 | 0.001693 |  |
| GREM2 | 65535 | 0.001771 | 0 | 0.002457 | 0 | 0.001716 |  |
| AKAP17A | 0.086395 | 0.645283 | 0.607775 | 0.652783 | 0.589192 | 0.001716 |  |
| ANKLE2 | 0.081285 | 0.344782 | 0.325893 | 0.41897 | 0.364202 | 0.001732 |  |
| SLC2A8 | 2.303071 | 0.002411 | 0.000489 | 0.004177 | 0.000804 | 0.001786 |  |
| CHMP4A | 0.089382 | 0.352075 | 0.330924 | 0.428554 | 0.372238 | 0.001809 |  |
| LACC1 | 1.341733 | 0.004921 | 0.001942 | 0.007863 | 0.003013 | 0.001823 |  |
| REPS1 | 0.090809 | 0.415824 | 0.390457 | 0.47856 | 0.420651 | 0.001825 |  |
| PRPF4B | 0.094151 | 0.434327 | 0.406888 | 0.511979 | 0.451185 | 0.001896 |  |
| NDUFS7 | 0.080911 | 0.341445 | 0.322823 | 0.413319 | 0.361993 | 0.001913 |  |
| PTK2B | 0.08754 | 0.60648 | 0.570775 | 0.628333 | 0.562676 | 0.001927 |  |
| UTRN | 0.081637 | 0.344214 | 0.325277 | 0.419462 | 0.367818 | 0.001931 |  |
| HPCAL1 | 0.077421 | 0.489325 | 0.463758 | 0.533481 | 0.472077 | 0.001933 |  |
| U2SURP | 0.081613 | 0.515994 | 0.487615 | 0.572798 | 0.506027 | 0.001971 |  |
| TCERG1 | 0.08952 | 0.573908 | 0.539379 | 0.616169 | 0.553033 | 0.001974 |  |
| PSMD3 | 0.07968 | 0.330617 | 0.312852 | 0.409878 | 0.356368 | 0.001975 |  |
| RHOH | 0.071302 | 0.95097 | 0.905113 | 0.794201 | 0.733427 | 0.002013 |  |
| WDFY2 | 0.08648 | 0.353164 | 0.332616 | 0.417987 | 0.365609 | 0.002035 |  |
| SMARCA2 | 0.078025 | 0.84584 | 0.80131 | 0.762993 | 0.697268 | 0.002051 |  |
| SAFB | 0.080891 | 0.506306 | 0.478699 | 0.562354 | 0.49779 | 0.002094 |  |
| ZNF292 | 0.093343 | 0.397116 | 0.372236 | 0.475366 | 0.418843 | 0.002116 |  |
| DYRK2 | 0.071813 | 0.892339 | 0.849008 | 0.786829 | 0.735637 | 0.002116 |  |
| TUT7 | 0.079224 | 0.508671 | 0.481491 | 0.523897 | 0.46364 | 0.00212 |  |
| IL7 | 1.469147 | 0.003091 | 0.001116 | 0.005529 | 0.001607 | 0.002139 |  |
| PLAAT4 | 0.073349 | 0.872479 | 0.829229 | 0.726256 | 0.671957 | 0.002197 |  |
| ARL6IP4 | 0.089529 | 0.504127 | 0.473794 | 0.564197 | 0.498795 | 0.002198 |  |
| SUSD3 | 0.07931 | 0.643066 | 0.608669 | 0.645411 | 0.582162 | 0.002219 |  |
| CDHR2 | 2.077354 | 0.002726 | 0.000646 | 0.003686 | 0.000603 | 0.002224 |  |
| SDSL | 3.416255 | 0.001872 | 0.000175 | 0.002826 | 0.000201 | 0.002265 |  |
| TOMM22 | 0.08753 | 0.37293 | 0.350976 | 0.450055 | 0.392125 | 0.002277 |  |
| IL10 | 65535 | 0.001465 | 0 | 0.002334 | 0 | 0.002279 |  |
| AIF1L | 1.023844 | 0.009854 | 0.004846 | 0.013147 | 0.00683 | 0.002314 |  |
| PLS3 | 1.705766 | 0.003471 | 0.001064 | 0.006143 | 0.002009 | 0.002316 |  |
| FBXO7 | 0.082652 | 0.436206 | 0.411918 | 0.503379 | 0.447168 | 0.002323 |  |
| GOLPH3 | 0.08513 | 0.538095 | 0.507262 | 0.578081 | 0.520892 | 0.002346 |  |
| DNM2 | 0.083331 | 0.690594 | 0.651835 | 0.687431 | 0.619727 | 0.00238 |  |
| MAML2 | 0.077937 | 0.471328 | 0.446541 | 0.487406 | 0.432101 | 0.00239 |  |
| LASP1 | 0.092299 | 0.529845 | 0.497009 | 0.560143 | 0.503013 | 0.002424 |  |
| RTF1 | 0.078293 | 0.335116 | 0.317414 | 0.404595 | 0.354359 | 0.002568 |  |
| RASA3 | 0.073695 | 0.884492 | 0.840446 | 0.769013 | 0.705906 | 0.002729 |  |
| RNPEPL1 | 0.076948 | 0.845702 | 0.801777 | 0.738543 | 0.677782 | 0.002746 |  |
| LNPEP | 0.079095 | 0.66032 | 0.625093 | 0.669001 | 0.60908 | 0.002759 |  |
| TOGARAM2 | 1.046004 | 0.004687 | 0.00227 | 0.008232 | 0.003415 | 0.002771 |  |
| RNF10 | 0.085113 | 0.544192 | 0.513015 | 0.582381 | 0.515267 | 0.002823 |  |
| SYNGAP1 | 0.091537 | 0.469555 | 0.440688 | 0.520703 | 0.460828 | 0.002886 |  |
| RC3H2 | 0.081606 | 0.415399 | 0.392555 | 0.480034 | 0.425271 | 0.002927 |  |
| SPCS1 | 0.084372 | 0.497355 | 0.469103 | 0.557562 | 0.495581 | 0.002952 |  |
| XRN1 | 0.07879 | 0.513899 | 0.486586 | 0.574149 | 0.501004 | 0.002972 |  |
| SRSF3 | 0.090748 | 0.495476 | 0.46527 | 0.5583 | 0.500804 | 0.002986 |  |
| PMPCB | 0.080327 | 0.493172 | 0.466463 | 0.545399 | 0.485737 | 0.003004 |  |
| CLK3 | 0.063282 | 0.29906 | 0.286225 | 0.376582 | 0.324628 | 0.003026 |  |
| FAM111B | 1.486146 | 0.002377 | 0.000848 | 0.003932 | 0.000804 | 0.003035 |  |
| SMOX | 1.313109 | 0.003831 | 0.001542 | 0.006635 | 0.002411 | 0.003096 |  |
| RBM15B | 0.07781 | 0.465648 | 0.441199 | 0.517631 | 0.460225 | 0.003096 |  |
| UNC13D | 0.080614 | 0.426169 | 0.403009 | 0.487161 | 0.432302 | 0.003155 |  |
| STAG1 | 0.087359 | 0.441988 | 0.416019 | 0.507188 | 0.449779 | 0.003184 |  |
| DLGAP5 | 1.467521 | 0.003745 | 0.001354 | 0.005652 | 0.001808 | 0.0032 |  |
| SELP | 1.065895 | 0.007627 | 0.003643 | 0.010444 | 0.005022 | 0.003214 |  |
| RRP36 | 0.062038 | 0.304338 | 0.291529 | 0.375476 | 0.325231 | 0.003272 |  |
| USP34 | 0.075938 | 0.654989 | 0.621405 | 0.669738 | 0.605665 | 0.00342 |  |
| MTMR3 | 0.069148 | 0.29052 | 0.276924 | 0.371176 | 0.321012 | 0.003482 |  |
| CMBL | 3.025145 | 0.002136 | 0.000262 | 0.003072 | 0.000402 | 0.003523 |  |
| ASB2 | 3.021131 | 0.001268 | 0.000156 | 0.003072 | 0.000402 | 0.003523 |  |
| EEF1A2 | 2.706276 | 0.001943 | 0.000298 | 0.003072 | 0.000402 | 0.003526 |  |
| HIGD2A | 0.087661 | 0.517004 | 0.486525 | 0.563706 | 0.507634 | 0.003692 |  |
| SF3B1 | 0.083266 | 0.551436 | 0.52051 | 0.61494 | 0.546806 | 0.00387 |  |
| RFFL | 0.067013 | 0.382291 | 0.36494 | 0.446492 | 0.390317 | 0.003956 |  |
| ITSN2 | 0.083254 | 0.367366 | 0.346767 | 0.448335 | 0.394134 | 0.004012 |  |
| OCM2 | 1.016796 | 0.007594 | 0.003753 | 0.010566 | 0.005223 | 0.004043 |  |
| CMTM3 | 0.080968 | 0.610607 | 0.577282 | 0.583118 | 0.52491 | 0.004072 |  |
| SF3A1 | 0.072934 | 0.327481 | 0.311337 | 0.425359 | 0.368622 | 0.004152 |  |
| KIF5B | 0.086464 | 0.409273 | 0.385465 | 0.47598 | 0.423463 | 0.004183 |  |
| CRACDL | 1.074473 | 0.005136 | 0.002439 | 0.007372 | 0.003013 | 0.004292 |  |
| MCM7 | 0.072578 | 0.44815 | 0.426162 | 0.49527 | 0.437726 | 0.004362 |  |
| TFCP2 | 0.076189 | 0.356338 | 0.338008 | 0.426957 | 0.375452 | 0.004692 |  |
| FBXW2 | 0.074855 | 0.364194 | 0.345779 | 0.441823 | 0.390317 | 0.004727 |  |
| PPP4R3A | 0.080351 | 0.47214 | 0.446563 | 0.539133 | 0.477702 | 0.004773 |  |
| MKNK2 | 0.078275 | 0.506724 | 0.479964 | 0.550313 | 0.490157 | 0.004885 |  |
| NUMA1 | 0.071063 | 0.776434 | 0.739115 | 0.741123 | 0.67718 | 0.004915 |  |
| NUDC | 0.078685 | 0.387058 | 0.366513 | 0.452881 | 0.402772 | 0.004923 |  |
| ETV7 | 2.456226 | 0.001969 | 0.000359 | 0.003317 | 0.000603 | 0.005039 |  |
| PTPN4 | 0.066222 | 0.38889 | 0.371442 | 0.448089 | 0.393732 | 0.005202 |  |
| RASA2 | 0.083916 | 0.459474 | 0.433511 | 0.523774 | 0.462636 | 0.005203 |  |
| PI4KA | 0.070572 | 0.48433 | 0.461209 | 0.537658 | 0.475894 | 0.005295 |  |
| SRP72 | 0.070711 | 0.396753 | 0.377776 | 0.477577 | 0.419446 | 0.005379 |  |
| CHTOP | 0.077551 | 0.483285 | 0.457993 | 0.538395 | 0.480916 | 0.005389 |  |
| ELF1 | 0.069496 | 0.746647 | 0.711533 | 0.699595 | 0.645641 | 0.005534 |  |
| HSPH1 | 0.078881 | 0.39898 | 0.377751 | 0.454601 | 0.404379 | 0.005553 |  |
| MYCBP2 | 0.078954 | 0.421366 | 0.398925 | 0.490478 | 0.433909 | 0.005572 |  |
| SRSF8 | 0.075266 | 0.370028 | 0.351218 | 0.436294 | 0.385697 | 0.005841 |  |
| LRBA | 0.064723 | 0.373838 | 0.357438 | 0.459885 | 0.40237 | 0.005841 |  |
| NDUFV1 | 0.074129 | 0.552277 | 0.524616 | 0.592087 | 0.525914 | 0.005965 |  |
| TSHZ3 | 1.469953 | 0.002916 | 0.001053 | 0.004669 | 0.001406 | 0.005999 |  |
| COPS2 | 0.068042 | 0.401972 | 0.383453 | 0.479174 | 0.422258 | 0.006081 |  |
| ISY1 | 0.066358 | 0.370496 | 0.353841 | 0.440718 | 0.385697 | 0.006306 |  |
| EGLN2 | 0.072441 | 0.500835 | 0.476308 | 0.553754 | 0.494978 | 0.00651 |  |
| XPO6 | 0.076178 | 0.45959 | 0.435952 | 0.538027 | 0.475492 | 0.006534 |  |
| GPT2 | 2.720759 | 0.00157 | 0.000238 | 0.003194 | 0.000603 | 0.006535 |  |
| RBM39 | 0.081145 | 0.455305 | 0.430404 | 0.534218 | 0.474287 | 0.006562 |  |
| MADCAM1 | 1.408517 | 0.002388 | 0.0009 | 0.003563 | 0.000804 | 0.006698 |  |
| CPNE1 | 0.073134 | 0.610756 | 0.580567 | 0.62735 | 0.561671 | 0.006818 |  |
| SYTL1 | 0.071823 | 0.69407 | 0.660363 | 0.659418 | 0.599638 | 0.00685 |  |
| CCND1 | 1.427398 | 0.005827 | 0.002167 | 0.007372 | 0.003214 | 0.006917 |  |
| SIVA1 | 0.079172 | 0.415641 | 0.393446 | 0.478314 | 0.423865 | 0.006981 |  |
| HMCES | 0.061131 | 0.36041 | 0.345457 | 0.441332 | 0.384291 | 0.007031 |  |
| LGALS9C | 3.335219 | 0.001358 | 0.000135 | 0.002334 | 0.000201 | 0.007097 |  |
| IL1R2 | 3.136846 | 0.001236 | 0.000141 | 0.002334 | 0.000201 | 0.007099 |  |
| RAB2A | 0.059177 | 0.332087 | 0.318741 | 0.413073 | 0.357975 | 0.007202 |  |
| PITPNC1 | 0.06566 | 0.92961 | 0.88825 | 0.797518 | 0.737043 | 0.007232 |  |
| PNMA8A | 65535 | 0.001248 | 0 | 0.001843 | 0 | 0.007266 |  |
| RBM6 | 0.075044 | 0.500407 | 0.475043 | 0.554614 | 0.49096 | 0.007341 |  |
| ACTR2 | 0.068757 | 0.297168 | 0.283338 | 0.386288 | 0.335878 | 0.007522 |  |
| HUWE1 | 0.075025 | 0.454158 | 0.431144 | 0.522669 | 0.469064 | 0.007569 |  |
| PAK2 | 0.066547 | 0.361793 | 0.345484 | 0.444772 | 0.389916 | 0.007627 |  |
| PGRMC2 | 0.068542 | 0.377909 | 0.360374 | 0.448458 | 0.395942 | 0.007977 |  |
| BUD23 | 0.058424 | 0.366755 | 0.352199 | 0.434083 | 0.381278 | 0.008061 |  |
| SOX18 | 2.697071 | 0.001716 | 0.000265 | 0.002703 | 0.000402 | 0.008102 |  |
| SELENON | 0.063866 | 0.439209 | 0.42019 | 0.479543 | 0.42507 | 0.008167 |  |
| SERF2 | 0.078167 | 0.540277 | 0.511783 | 0.597248 | 0.53415 | 0.008432 |  |
| TNFRSF8 | 2.307789 | 0.001832 | 0.00037 | 0.003072 | 0.000603 | 0.008527 |  |
| CFAP73 | 1.938796 | 0.002185 | 0.00057 | 0.00344 | 0.000804 | 0.008628 |  |
| STAT5B | 0.075589 | 0.728763 | 0.691563 | 0.710038 | 0.649859 | 0.009011 |  |
| STK39 | 0.077717 | 0.38225 | 0.362203 | 0.45583 | 0.403375 | 0.009112 |  |
| CNTRL | 0.064853 | 0.601072 | 0.574651 | 0.60118 | 0.538771 | 0.009134 |  |
| PDCD6 | 0.068686 | 0.410588 | 0.391498 | 0.486301 | 0.427481 | 0.00917 |  |
| BAX | 0.073785 | 0.430897 | 0.409413 | 0.484458 | 0.431699 | 0.009333 |  |
| TGFBR2 | 0.059836 | 1.217199 | 1.167748 | 0.86534 | 0.815187 | 0.009444 |  |
| PTH2 | 65535 | 0.001012 | 0 | 0.00172 | 0 | 0.00968 |  |
| DDX23 | 0.053416 | 0.353868 | 0.341005 | 0.426588 | 0.375653 | 0.009692 |  |
| NBR1 | 0.067077 | 0.391113 | 0.373345 | 0.467134 | 0.413218 | 0.009844 |  |
| **Regulatory T cells** | |  |  |  |  |  |  |
| MT-ND2 | 1.547541 | 1.622624 | 0.555088 | 0.884211 | 0.377358 | 9.62E-15 |  |
| SOCS3 | 2.107913 | 1.234158 | 0.286303 | 0.731579 | 0.320755 | 2.25E-13 |  |
| DDX3Y | 2.392174 | 0.38951 | 0.0742 | 0.415789 | 0.066038 | 7.44E-06 |  |
| ARID5B | 0.91373 | 0.855709 | 0.454219 | 0.736842 | 0.433962 | 0.001056 |  |
| UTY | 3.328649 | 0.226187 | 0.022514 | 0.247368 | 0.028302 | 0.002663 |  |
| BCL3 | 2.783447 | 0.223871 | 0.032516 | 0.278947 | 0.04717 | 0.002663 |  |
| SBNO2 | 1.078625 | 0.603422 | 0.285708 | 0.594737 | 0.330189 | 0.005251 |  |
| CD55 | 0.761523 | 0.732729 | 0.432217 | 0.736842 | 0.462264 | 0.007182 |  |
| **TH1 T cells** | |  |  |  |  |  |  |
| MT-ND2 | 1.564921 | 3.266885 | 1.104195 | 0.959292 | 0.526316 | 4.27E-20 |  |
| EIF1AY | 2.108785 | 0.930907 | 0.215823 | 0.59823 | 0.157895 | 2.77E-08 |  |
| MT2A | 0.8243 | 1.434782 | 0.810302 | 0.849558 | 0.618421 | 5.02E-06 |  |
| NOP58 | 0.611967 | 1.380417 | 0.903212 | 0.863717 | 0.723684 | 1.34E-05 |  |
| DDX3Y | 1.44588 | 0.749421 | 0.275089 | 0.553982 | 0.197368 | 4.27E-05 |  |
| ATP5PO | 1.375413 | 0.543016 | 0.209301 | 0.561062 | 0.223684 | 0.000154 |  |
| TAGLN2 | 0.458413 | 1.687777 | 1.228341 | 0.907965 | 0.802632 | 0.000168 |  |
| RRP7A | 1.087313 | 0.708564 | 0.333477 | 0.628319 | 0.407895 | 0.000544 |  |
| GIMAP6 | 0.437064 | 1.641402 | 1.212399 | 0.909735 | 0.842105 | 0.000557 |  |
| ITGA4 | 0.36842 | 2.479286 | 1.92053 | 0.916814 | 0.855263 | 0.000747 |  |
| THEMIS2 | 0.815577 | 0.874634 | 0.496951 | 0.732743 | 0.5 | 0.00115 |  |
| GIMAP1 | 0.498046 | 1.536226 | 1.087748 | 0.902655 | 0.776316 | 0.003285 |  |
| CCDC167 | 0.454825 | 1.377117 | 1.004743 | 0.851327 | 0.736842 | 0.003408 |  |
| ACTN4 | 0.413288 | 1.628041 | 1.222513 | 0.876106 | 0.776316 | 0.003874 |  |
| PMAIP1 | 0.562854 | 1.482624 | 1.00368 | 0.821239 | 0.723684 | 0.004089 |  |
| KDM5D | 1.467837 | 0.413652 | 0.149545 | 0.39115 | 0.144737 | 0.006292 |  |
| **TH2 T cells** | |  |  |  |  |  |  |
| FMN1 | 1.877267 | 0.827372 | 0.22521 | 0.720395 | 0.245902 | 4.99E-15 |  |
| SOCS3 | 0.971538 | 1.481981 | 0.755754 | 0.845395 | 0.557377 | 2.57E-08 |  |
| UBALD2 | 1.096747 | 0.693769 | 0.324385 | 0.657895 | 0.377049 | 2.83E-05 |  |
| SOCS1 | 1.320558 | 0.536461 | 0.214788 | 0.536184 | 0.245902 | 5.02E-05 |  |
| DDX3Y | 1.264356 | 0.572746 | 0.238426 | 0.526316 | 0.213115 | 6.39E-05 |  |
| BTG1 | 0.361308 | 1.81841 | 1.415557 | 0.973684 | 0.852459 | 8.29E-05 |  |
| SLC2A3 | 0.82221 | 0.945472 | 0.534735 | 0.733553 | 0.5 | 8.79E-05 |  |
| SERPINB9 | 0.981408 | 0.660464 | 0.334515 | 0.654605 | 0.360656 | 0.000131 |  |
| SMAP2 | 0.740418 | 0.711089 | 0.425634 | 0.707237 | 0.442623 | 0.000992 |  |
| DDIT4 | 0.444453 | 1.412528 | 1.038015 | 0.901316 | 0.770492 | 0.000992 |  |
| CD69 | 0.306919 | 2.12912 | 1.72111 | 0.960526 | 0.885246 | 0.000992 |  |
| DUSP2 | 1.215434 | 0.531653 | 0.228953 | 0.496711 | 0.262295 | 0.001099 |  |
| BCL3 | 1.088914 | 0.406199 | 0.19096 | 0.480263 | 0.221311 | 0.003804 |  |
| PIK3IP1 | 0.703536 | 0.702126 | 0.431151 | 0.694079 | 0.45082 | 0.004148 |  |
| FBXL16 | 1.987078 | 0.206095 | 0.051987 | 0.253289 | 0.057377 | 0.004183 |  |
| PIM2 | 0.465616 | 1.334211 | 0.966185 | 0.881579 | 0.721311 | 0.004183 |  |
| ZFP36 | 0.398805 | 1.71704 | 1.302352 | 0.921053 | 0.819672 | 0.004183 |  |
| SFMBT2 | 0.933623 | 0.496482 | 0.259929 | 0.509868 | 0.245902 | 0.004738 |  |
| CCNL1 | 0.690308 | 0.611447 | 0.378927 | 0.634868 | 0.442623 | 0.004738 |  |
| PLAC8 | 0.411632 | 1.38044 | 1.037777 | 0.914474 | 0.770492 | 0.00931 |  |
| **CD8+ T cells** | |  |  |  |  |  |  |
| MT-ND2 | 1.268339 | 1.554825 | 0.645466 | 0.85261 | 0.389157 | 2.07E-78 |  |
| C20orf204 | 2.777252 | 0.240523 | 0.035085 | 0.281474 | 0.046988 | 7.2E-36 |  |
| S100B | 1.578917 | 0.818408 | 0.273948 | 0.460594 | 0.187952 | 6.56E-34 |  |
| SOCS3 | 1.201383 | 0.781575 | 0.339874 | 0.587513 | 0.318072 | 1.67E-31 |  |
| DDX3Y | 1.654167 | 0.361756 | 0.114938 | 0.340839 | 0.124096 | 6.89E-25 |  |
| FAM107B | 0.322216 | 1.487422 | 1.1897 | 0.95087 | 0.874699 | 1.61E-24 |  |
| NPDC1 | 1.498181 | 0.370457 | 0.131142 | 0.366428 | 0.146988 | 4.9E-24 |  |
| ZFP36 | 0.578348 | 1.27677 | 0.855092 | 0.812692 | 0.666265 | 3.77E-21 |  |
| DDIT4 | 0.836667 | 0.664708 | 0.372194 | 0.55783 | 0.350602 | 2.12E-17 |  |
| ANXA1 | 0.85058 | 0.644555 | 0.357446 | 0.528147 | 0.314458 | 6.33E-17 |  |
| BTG2 | 0.330923 | 1.517763 | 1.206663 | 0.899693 | 0.815663 | 8.12E-15 |  |
| MT2A | 1.160336 | 0.26478 | 0.118465 | 0.323439 | 0.145783 | 1.2E-14 |  |
| THEMIS2 | 0.975701 | 0.280737 | 0.142752 | 0.341863 | 0.16506 | 1.56E-13 |  |
| TIMP1 | 1.296185 | 0.214529 | 0.087357 | 0.256909 | 0.107229 | 3.35E-13 |  |
| KLF6 | 0.279052 | 1.735787 | 1.430518 | 0.908905 | 0.848193 | 7.87E-13 |  |
| FMN1 | 1.817221 | 0.11888 | 0.033734 | 0.151484 | 0.037349 | 8.06E-13 |  |
| SLC7A8 | 2.015757 | 0.112687 | 0.027866 | 0.139202 | 0.03253 | 2.96E-12 |  |
| KLRG1 | 1.528295 | 0.172859 | 0.059928 | 0.19652 | 0.06988 | 2.96E-12 |  |
| ATP5PO | 1.40124 | 0.140085 | 0.053037 | 0.201638 | 0.072289 | 3.22E-12 |  |
| GIMAP4 | 0.440657 | 0.980522 | 0.722447 | 0.763562 | 0.615663 | 3.22E-12 |  |
| DTX1 | 1.743082 | 0.134071 | 0.040051 | 0.154555 | 0.048193 | 8.34E-11 |  |
| NFKBIZ | 0.534941 | 0.738338 | 0.509591 | 0.643808 | 0.480723 | 1.05E-10 |  |
| PDE6G | 1.024365 | 0.219836 | 0.108077 | 0.257932 | 0.125301 | 1.05E-09 |  |
| USP53 | 0.970036 | 0.24279 | 0.123942 | 0.288639 | 0.148193 | 1.36E-09 |  |
| CALHM6 | 1.526849 | 0.111312 | 0.038629 | 0.148414 | 0.048193 | 1.4E-09 |  |
| SOCS1 | 0.820155 | 0.364468 | 0.206428 | 0.390993 | 0.248193 | 1.5E-09 |  |
| GPRIN3 | 0.667185 | 0.434216 | 0.273441 | 0.474923 | 0.310843 | 1.57E-09 |  |
| CD55 | 0.309263 | 1.082652 | 0.873759 | 0.848516 | 0.751807 | 1.9E-09 |  |
| SERPINB9 | 0.36222 | 0.918802 | 0.714797 | 0.770727 | 0.627711 | 3.17E-09 |  |
| UTY | 1.21729 | 0.171749 | 0.073868 | 0.206755 | 0.093976 | 3.18E-09 |  |
| TIPARP | 0.703425 | 0.380059 | 0.233399 | 0.421699 | 0.271084 | 4.01E-09 |  |
| CR1 | 1.058225 | 0.216691 | 0.10406 | 0.242579 | 0.121687 | 1.03E-08 |  |
| PRPF6 | 0.458096 | 0.637655 | 0.464179 | 0.650972 | 0.50241 | 1.03E-08 |  |
| IL4R | 1.158542 | 0.145594 | 0.065221 | 0.205732 | 0.093976 | 1.52E-08 |  |
| PHACTR2 | 1.444666 | 0.105046 | 0.038591 | 0.149437 | 0.054217 | 1.6E-08 |  |
| GBP5 | 1.510527 | 0.154934 | 0.054379 | 0.16479 | 0.066265 | 1.61E-08 |  |
| YWHAB | 0.542617 | 0.436775 | 0.299856 | 0.5261 | 0.366265 | 1.61E-08 |  |
| PLXNA4 | 1.055699 | 0.189194 | 0.091015 | 0.209826 | 0.096386 | 1.76E-08 |  |
| NFKBIA | 0.186009 | 2.009171 | 1.76613 | 0.953941 | 0.903614 | 1.76E-08 |  |
| TRAF1 | 0.790309 | 0.245916 | 0.142194 | 0.308086 | 0.171084 | 1.83E-08 |  |
| SYNE2 | 0.418446 | 0.770145 | 0.576246 | 0.692938 | 0.540964 | 1.83E-08 |  |
| MYC | 0.295155 | 1.308591 | 1.066482 | 0.847492 | 0.755422 | 2.92E-08 |  |
| NR4A2 | 0.542168 | 0.689588 | 0.473567 | 0.548618 | 0.398795 | 2.97E-08 |  |
| SAMD10 | 0.921958 | 0.187217 | 0.098811 | 0.24565 | 0.126506 | 4.09E-08 |  |
| IDS | 0.514467 | 0.551082 | 0.385786 | 0.567042 | 0.424096 | 4.09E-08 |  |
| DEF6 | 0.23021 | 1.228624 | 1.047415 | 0.891505 | 0.816867 | 5.01E-08 |  |
| NAMPT | 0.715447 | 0.243729 | 0.148435 | 0.330604 | 0.187952 | 5.56E-08 |  |
| PRDX2 | 0.442819 | 0.636939 | 0.468593 | 0.617195 | 0.457831 | 6.46E-08 |  |
| EIF1AY | 1.233451 | 0.155781 | 0.066253 | 0.178096 | 0.079518 | 8.25E-08 |  |
| ATP10A | 0.688221 | 0.327629 | 0.203333 | 0.385875 | 0.248193 | 8.25E-08 |  |
| SERPINB6 | 1.384926 | 0.135033 | 0.051705 | 0.163767 | 0.06988 | 8.52E-08 |  |
| ZNF66 | 1.428127 | 0.095674 | 0.035554 | 0.135107 | 0.048193 | 9.51E-08 |  |
| USP9Y | 1.754068 | 0.106842 | 0.031675 | 0.127943 | 0.046988 | 1.51E-07 |  |
| GPR183 | 0.994777 | 0.219137 | 0.109966 | 0.222108 | 0.113253 | 1.98E-07 |  |
| NTPCR | 0.902264 | 0.188099 | 0.100642 | 0.240532 | 0.126506 | 2.12E-07 |  |
| MGAT2 | 0.697382 | 0.241588 | 0.148985 | 0.308086 | 0.180723 | 4.18E-07 |  |
| TFEB | 1.193112 | 0.11238 | 0.04915 | 0.154555 | 0.063855 | 4.21E-07 |  |
| AVEN | 1.046751 | 0.121361 | 0.058746 | 0.16479 | 0.073494 | 1.09E-06 |  |
| SBNO2 | 0.602709 | 0.340053 | 0.223931 | 0.398158 | 0.26506 | 1.53E-06 |  |
| B4GALT1 | 0.344084 | 0.831089 | 0.654738 | 0.726714 | 0.609639 | 1.64E-06 |  |
| HDGFL3 | 1.100859 | 0.14304 | 0.066691 | 0.171955 | 0.081928 | 1.79E-06 |  |
| PTGER2 | 0.948873 | 0.183003 | 0.094802 | 0.187308 | 0.091566 | 2.01E-06 |  |
| CSRNP1 | 0.368839 | 0.823008 | 0.637342 | 0.684749 | 0.566265 | 2.04E-06 |  |
| NOP58 | 0.453666 | 0.534488 | 0.390275 | 0.566018 | 0.443373 | 2.18E-06 |  |
| DSC1 | 1.006678 | 0.186863 | 0.093 | 0.200614 | 0.103614 | 2.22E-06 |  |
| CLIC3 | 0.834906 | 0.188266 | 0.105545 | 0.220061 | 0.116867 | 2.29E-06 |  |
| TNFRSF13C | 1.288337 | 0.083086 | 0.034017 | 0.117707 | 0.043373 | 2.42E-06 |  |
| EZR | 0.315311 | 0.894053 | 0.718531 | 0.785056 | 0.668675 | 2.99E-06 |  |
| CD226 | 0.592253 | 0.284208 | 0.188517 | 0.356192 | 0.23253 | 4.48E-06 |  |
| ITM2A | 0.300965 | 0.936601 | 0.760248 | 0.761515 | 0.636145 | 4.81E-06 |  |
| GPR132 | 0.661538 | 0.354373 | 0.224036 | 0.368475 | 0.250602 | 4.81E-06 |  |
| PIK3IP1 | 0.189551 | 1.341663 | 1.176476 | 0.925281 | 0.856627 | 5.42E-06 |  |
| PIM2 | 0.246197 | 1.195118 | 1.007623 | 0.847492 | 0.774699 | 6.2E-06 |  |
| FAN1 | 0.826164 | 0.190313 | 0.107342 | 0.238485 | 0.137349 | 8.06E-06 |  |
| MTSS2 | 0.840013 | 0.19835 | 0.110806 | 0.237462 | 0.139759 | 8.73E-06 |  |
| KDM5D | 1.046304 | 0.156083 | 0.075576 | 0.187308 | 0.101205 | 9.07E-06 |  |
| ARL4C | 0.252887 | 1.09728 | 0.920855 | 0.83521 | 0.737349 | 1.01E-05 |  |
| GIMAP5 | 0.352315 | 0.67667 | 0.530053 | 0.661208 | 0.533735 | 1.02E-05 |  |
| NR4A3 | 0.752219 | 0.179649 | 0.106656 | 0.221085 | 0.121687 | 1.05E-05 |  |
| BCL2L11 | 0.458478 | 0.362585 | 0.263873 | 0.423746 | 0.287952 | 1.51E-05 |  |
| GIMAP6 | 0.424784 | 0.579954 | 0.432037 | 0.570113 | 0.444578 | 1.51E-05 |  |
| RUNDC3B | 4.660545 | 0.023855 | 0.000943 | 0.035824 | 0.001205 | 1.59E-05 |  |
| NSMCE3 | 0.275609 | 0.968812 | 0.800337 | 0.779939 | 0.668675 | 1.89E-05 |  |
| LRRC37A2 | 3.061951 | 0.027773 | 0.003326 | 0.044012 | 0.004819 | 1.91E-05 |  |
| PIEZO1 | 0.717633 | 0.171542 | 0.104314 | 0.226203 | 0.127711 | 2.22E-05 |  |
| IDI1 | 0.665524 | 0.230629 | 0.145403 | 0.296827 | 0.187952 | 2.23E-05 |  |
| NANS | 0.674344 | 0.208858 | 0.130874 | 0.266121 | 0.166265 | 3.07E-05 |  |
| PECR | 0.640056 | 0.222458 | 0.142748 | 0.287615 | 0.181928 | 3.17E-05 |  |
| XBP1 | 0.636289 | 0.261244 | 0.168076 | 0.30911 | 0.203614 | 3.63E-05 |  |
| PGAP6 | 0.4378 | 0.400589 | 0.295739 | 0.450358 | 0.325301 | 3.72E-05 |  |
| SLAMF6 | 0.468588 | 0.357384 | 0.258271 | 0.415558 | 0.289157 | 3.77E-05 |  |
| DNAJC24 | 0.84355 | 0.139997 | 0.078016 | 0.190379 | 0.10241 | 3.94E-05 |  |
| NCK2 | 0.29491 | 0.764416 | 0.623093 | 0.729785 | 0.616867 | 4.41E-05 |  |
| FLOT1 | 0.468469 | 0.396975 | 0.286906 | 0.451382 | 0.331325 | 5.12E-05 |  |
| USP36 | 0.395418 | 0.422221 | 0.321002 | 0.499488 | 0.366265 | 5.82E-05 |  |
| LINS1 | 0.358879 | 0.553108 | 0.431297 | 0.597748 | 0.454217 | 6.21E-05 |  |
| DDX24 | 0.374473 | 0.499851 | 0.385579 | 0.573183 | 0.427711 | 6.42E-05 |  |
| PPP1R15B | 0.212285 | 1.048865 | 0.905348 | 0.827021 | 0.763855 | 6.42E-05 |  |
| STX10 | 0.523332 | 0.285199 | 0.198431 | 0.357216 | 0.243373 | 6.76E-05 |  |
| NT5E | 0.420443 | 0.408366 | 0.305129 | 0.446264 | 0.319277 | 7.55E-05 |  |
| EOLA1 | 0.732088 | 0.186537 | 0.112301 | 0.240532 | 0.146988 | 7.66E-05 |  |
| GBP4 | 0.479013 | 0.259045 | 0.185857 | 0.30911 | 0.195181 | 7.7E-05 |  |
| ABCA1 | 1.195187 | 0.091845 | 0.040112 | 0.110542 | 0.046988 | 7.82E-05 |  |
| NKG7 | 0.591835 | 0.281703 | 0.186909 | 0.322416 | 0.213253 | 7.9E-05 |  |
| MYO1F | 1.137426 | 0.072472 | 0.032944 | 0.102354 | 0.040964 | 9.05E-05 |  |
| DENND4A | 0.331325 | 0.679854 | 0.540352 | 0.662231 | 0.559036 | 9.45E-05 |  |
| CDK5RAP3 | 0.308534 | 0.658115 | 0.531403 | 0.672467 | 0.540964 | 9.53E-05 |  |
| POLR3E | 0.486937 | 0.301669 | 0.215252 | 0.384852 | 0.262651 | 9.6E-05 |  |
| ACOT9 | 1.009292 | 0.08555 | 0.0425 | 0.122825 | 0.055422 | 9.97E-05 |  |
| GNB5 | 0.455908 | 0.394643 | 0.287715 | 0.455476 | 0.333735 | 9.97E-05 |  |
| IRF1 | 0.235533 | 1.22792 | 1.04296 | 0.845445 | 0.742169 | 9.97E-05 |  |
| SMAP2 | 0.178469 | 1.205986 | 1.065658 | 0.899693 | 0.83494 | 0.000104 |  |
| RNF44 | 0.228644 | 0.981791 | 0.837897 | 0.834186 | 0.731325 | 0.000106 |  |
| UBALD2 | 0.300451 | 0.7678 | 0.623452 | 0.713408 | 0.612048 | 0.000109 |  |
| MCM6 | 0.493428 | 0.293364 | 0.208387 | 0.34391 | 0.23012 | 0.000119 |  |
| IVNS1ABP | 0.272075 | 0.839612 | 0.695305 | 0.757421 | 0.645783 | 0.000122 |  |
| JMJD1C | 0.238867 | 0.932172 | 0.789932 | 0.787103 | 0.712048 | 0.000128 |  |
| MTFR1 | 1.375159 | 0.063622 | 0.024527 | 0.090072 | 0.03494 | 0.00017 |  |
| ADGRE5 | 0.203412 | 1.315261 | 1.142297 | 0.879222 | 0.803614 | 0.000208 |  |
| HNRNPF | 0.238343 | 0.902216 | 0.764825 | 0.794268 | 0.685542 | 0.000232 |  |
| ZNF141 | 0.612699 | 0.223066 | 0.145879 | 0.282497 | 0.186747 | 0.000234 |  |
| PRKCA | 0.428915 | 0.369813 | 0.274704 | 0.428864 | 0.312048 | 0.000241 |  |
| LTBP3 | 0.277919 | 0.803556 | 0.662756 | 0.714432 | 0.607229 | 0.000241 |  |
| MATK | 0.405726 | 0.444369 | 0.335435 | 0.468782 | 0.351807 | 0.000274 |  |
| SRGN | 0.208629 | 1.145761 | 0.991494 | 0.831116 | 0.753012 | 0.000293 |  |
| CEBPB | 0.7405 | 0.122185 | 0.073131 | 0.168884 | 0.092771 | 0.000295 |  |
| ACSF3 | 0.387004 | 0.381995 | 0.292118 | 0.451382 | 0.325301 | 0.000303 |  |
| TENT5C | 0.852804 | 0.139555 | 0.077273 | 0.168884 | 0.095181 | 0.000319 |  |
| PRR5 | 0.415684 | 0.353237 | 0.264809 | 0.415558 | 0.304819 | 0.000321 |  |
| DUSP2 | 0.468624 | 0.51164 | 0.369739 | 0.458547 | 0.359036 | 0.000338 |  |
| WWC3 | 0.547909 | 0.196018 | 0.134079 | 0.261003 | 0.166265 | 0.000351 |  |
| GRK5 | 0.721472 | 0.181306 | 0.109958 | 0.223132 | 0.139759 | 0.000363 |  |
| ATP2B1 | 0.449114 | 0.350625 | 0.25683 | 0.414534 | 0.309639 | 0.000363 |  |
| SFMBT2 | 0.405859 | 0.452905 | 0.341847 | 0.496418 | 0.387952 | 0.000386 |  |
| TNFRSF10B | 1.350303 | 0.056766 | 0.022264 | 0.079836 | 0.03012 | 0.000399 |  |
| DNAJC5 | 0.337167 | 0.418335 | 0.331151 | 0.505629 | 0.36747 | 0.000409 |  |
| DPP4 | 0.464704 | 0.276201 | 0.200141 | 0.319345 | 0.216867 | 0.000434 |  |
| RALGDS | 0.396728 | 0.339626 | 0.257973 | 0.418628 | 0.309639 | 0.000468 |  |
| CYBRD1 | 5.118224 | 0.018156 | 0.000523 | 0.026612 | 0.001205 | 0.000541 |  |
| EIF4E3 | 0.317441 | 0.579033 | 0.464669 | 0.599795 | 0.474699 | 0.000579 |  |
| JAK3 | 0.213865 | 0.994803 | 0.857744 | 0.825998 | 0.749398 | 0.000591 |  |
| CHMP1B | 0.235639 | 0.906391 | 0.769805 | 0.76868 | 0.66506 | 0.000605 |  |
| TRANK1 | 0.434287 | 0.290944 | 0.215316 | 0.367451 | 0.255422 | 0.000642 |  |
| DNPEP | 0.467381 | 0.245265 | 0.177395 | 0.314227 | 0.210843 | 0.000647 |  |
| TAF4B | 0.506943 | 0.210129 | 0.14787 | 0.284545 | 0.186747 | 0.00068 |  |
| ITM2B | 0.235172 | 0.795583 | 0.675914 | 0.757421 | 0.657831 | 0.000697 |  |
| ZC3H12D | 0.679363 | 0.24706 | 0.154275 | 0.254862 | 0.172289 | 0.00074 |  |
| RRP7A | 0.588967 | 0.1777 | 0.118138 | 0.243603 | 0.155422 | 0.000771 |  |
| NINJ1 | 0.358314 | 0.404362 | 0.315433 | 0.457523 | 0.342169 | 0.000771 |  |
| CRYGN | 65535 | 0.013375 | 0 | 0.022518 | 0 | 0.000772 |  |
| WDR86 | 3.34696 | 0.021083 | 0.002072 | 0.028659 | 0.00241 | 0.000772 |  |
| VPS28 | 0.352844 | 0.425596 | 0.333258 | 0.498465 | 0.378313 | 0.000772 |  |
| APOBEC3C | 0.521074 | 0.197147 | 0.137382 | 0.255885 | 0.166265 | 0.000829 |  |
| PCNT | 0.304682 | 0.586074 | 0.474497 | 0.622313 | 0.509639 | 0.00083 |  |
| MVB12B | 0.444282 | 0.277338 | 0.203829 | 0.335722 | 0.231325 | 0.000883 |  |
| FBXO32 | 0.545265 | 0.261633 | 0.179288 | 0.298874 | 0.210843 | 0.000886 |  |
| SQSTM1 | 0.382208 | 0.420749 | 0.322825 | 0.475947 | 0.36988 | 0.000914 |  |
| CYCS | 0.565867 | 0.205754 | 0.138997 | 0.274309 | 0.185542 | 0.000976 |  |
| DOCK8 | 0.288344 | 0.629935 | 0.515817 | 0.659161 | 0.53494 | 0.000997 |  |
| MX1 | 0.526783 | 0.245393 | 0.170327 | 0.281474 | 0.190361 | 0.001 |  |
| TARS3 | 0.814497 | 0.088325 | 0.050222 | 0.12999 | 0.06747 | 0.001015 |  |
| GOLGA8B | 0.558701 | 0.201513 | 0.13681 | 0.264074 | 0.178313 | 0.001016 |  |
| TMEM204 | 0.347236 | 0.464056 | 0.364789 | 0.504606 | 0.389157 | 0.001057 |  |
| PRSS21 | 1.735021 | 0.039101 | 0.011746 | 0.053224 | 0.015663 | 0.001073 |  |
| ELL2 | 0.927625 | 0.093993 | 0.049414 | 0.128966 | 0.06747 | 0.001116 |  |
| EGR3 | 0.795999 | 0.096399 | 0.055521 | 0.121801 | 0.061446 | 0.001131 |  |
| FOSL2 | 0.715021 | 0.174591 | 0.10636 | 0.206755 | 0.13253 | 0.001199 |  |
| STX4 | 0.445197 | 0.278401 | 0.204481 | 0.339816 | 0.240964 | 0.001269 |  |
| CBLB | 0.302443 | 0.597045 | 0.484131 | 0.590583 | 0.477108 | 0.001271 |  |
| DCXR | 0.432768 | 0.270768 | 0.200596 | 0.335722 | 0.237349 | 0.001309 |  |
| TFRC | 0.430901 | 0.309201 | 0.229365 | 0.383828 | 0.284337 | 0.001309 |  |
| RNF157 | 0.462575 | 0.291928 | 0.211849 | 0.324463 | 0.228916 | 0.001313 |  |
| S100A4 | 0.389176 | 0.374333 | 0.285828 | 0.387922 | 0.284337 | 0.001313 |  |
| SDE2 | 0.386915 | 0.353588 | 0.270411 | 0.428864 | 0.319277 | 0.001313 |  |
| XRRA1 | 0.875918 | 0.093675 | 0.051044 | 0.123849 | 0.06506 | 0.001335 |  |
| PRR7 | 0.442096 | 0.221594 | 0.163107 | 0.282497 | 0.187952 | 0.001335 |  |
| APOBEC3G | 0.598988 | 0.1266 | 0.083583 | 0.174002 | 0.101205 | 0.001373 |  |
| STX2 | 0.620325 | 0.159788 | 0.103946 | 0.205732 | 0.131325 | 0.00148 |  |
| BST2 | 0.476315 | 0.277617 | 0.199554 | 0.315251 | 0.220482 | 0.001577 |  |
| GPCPD1 | 0.276939 | 0.655079 | 0.540663 | 0.664278 | 0.543373 | 0.001611 |  |
| PMAIP1 | 0.651246 | 0.150803 | 0.096021 | 0.180143 | 0.108434 | 0.001647 |  |
| TRMT1 | 0.327413 | 0.443231 | 0.35324 | 0.505629 | 0.389157 | 0.001647 |  |
| AHNAK | 0.500621 | 0.326006 | 0.230422 | 0.319345 | 0.228916 | 0.001758 |  |
| TOMM40L | 0.797941 | 0.085646 | 0.049261 | 0.123849 | 0.06506 | 0.00176 |  |
| CSNK1D | 0.304945 | 0.456941 | 0.369882 | 0.532242 | 0.412048 | 0.001773 |  |
| MCPH1 | 0.403989 | 0.315925 | 0.238766 | 0.382805 | 0.283133 | 0.001856 |  |
| SPSB3 | 0.380869 | 0.359044 | 0.275736 | 0.425793 | 0.321687 | 0.001856 |  |
| PRR3 | 0.858339 | 0.093776 | 0.051726 | 0.12999 | 0.06988 | 0.001865 |  |
| LPIN2 | 0.385334 | 0.32624 | 0.24977 | 0.394063 | 0.293976 | 0.001947 |  |
| EXOC7 | 0.293065 | 0.538987 | 0.439903 | 0.586489 | 0.46506 | 0.001956 |  |
| PLK3 | 0.395653 | 0.362141 | 0.27528 | 0.42477 | 0.321687 | 0.001969 |  |
| CH25H | 1.825615 | 0.032233 | 0.009094 | 0.042989 | 0.010843 | 0.00197 |  |
| KLHL12 | 0.528797 | 0.176987 | 0.122675 | 0.229273 | 0.149398 | 0.002372 |  |
| GIMAP8 | 0.374689 | 0.3276 | 0.252668 | 0.376663 | 0.274699 | 0.002372 |  |
| TSPAN14 | 0.175242 | 0.995678 | 0.881791 | 0.839304 | 0.762651 | 0.00244 |  |
| C16orf74 | 1.154227 | 0.049287 | 0.022145 | 0.071648 | 0.028916 | 0.002594 |  |
| SHISAL2A | 0.578718 | 0.173667 | 0.11628 | 0.215967 | 0.139759 | 0.002639 |  |
| WDR36 | 0.529848 | 0.171435 | 0.118741 | 0.232344 | 0.154217 | 0.002639 |  |
| INSL3 | 1.06184 | 0.063746 | 0.030536 | 0.087001 | 0.039759 | 0.002699 |  |
| STAT3 | 0.289741 | 0.56814 | 0.464766 | 0.607984 | 0.486747 | 0.002875 |  |
| DTX3L | 0.419871 | 0.300371 | 0.224525 | 0.356192 | 0.261446 | 0.002883 |  |
| LTB4R | 0.508222 | 0.222677 | 0.156561 | 0.274309 | 0.192771 | 0.002979 |  |
| IRF2 | 0.300954 | 0.473699 | 0.384509 | 0.534289 | 0.413253 | 0.002998 |  |
| SLC9A8 | 0.601814 | 0.133538 | 0.087992 | 0.184237 | 0.113253 | 0.003067 |  |
| TTC21B | 0.485182 | 0.199518 | 0.142537 | 0.257932 | 0.175904 | 0.003072 |  |
| TIMM10 | 0.391221 | 0.32339 | 0.24658 | 0.368475 | 0.275904 | 0.003076 |  |
| SORBS3 | 0.817781 | 0.102528 | 0.058165 | 0.12999 | 0.072289 | 0.003228 |  |
| TMEM30A | 0.618042 | 0.123792 | 0.080657 | 0.177073 | 0.108434 | 0.003254 |  |
| ARL14EP | 0.477429 | 0.194952 | 0.140025 | 0.25998 | 0.177108 | 0.003277 |  |
| ZNF410 | 0.351231 | 0.375372 | 0.29426 | 0.441146 | 0.340964 | 0.003277 |  |
| NT5DC3 | 1.101403 | 0.054017 | 0.025175 | 0.073695 | 0.031325 | 0.003344 |  |
| RNH1 | 0.294285 | 0.530238 | 0.432396 | 0.576254 | 0.463855 | 0.003426 |  |
| ZFP91 | 0.308696 | 0.477762 | 0.385731 | 0.544524 | 0.436145 | 0.003587 |  |
| ACADVL | 0.372046 | 0.314158 | 0.242746 | 0.394063 | 0.293976 | 0.003677 |  |
| ZNF331 | 0.34365 | 0.454393 | 0.358082 | 0.455476 | 0.359036 | 0.003713 |  |
| ABCA7 | 0.436915 | 0.240979 | 0.178015 | 0.313204 | 0.224096 | 0.003728 |  |
| SLC4A7 | 0.433918 | 0.254797 | 0.188613 | 0.327533 | 0.239759 | 0.00384 |  |
| SLC7A5 | 0.702165 | 0.094056 | 0.057812 | 0.136131 | 0.077108 | 0.00403 |  |
| KLF3 | 0.227093 | 0.871475 | 0.744548 | 0.761515 | 0.650602 | 0.004045 |  |
| SSBP4 | 0.553276 | 0.143304 | 0.097657 | 0.207779 | 0.13494 | 0.004047 |  |
| ARHGAP26 | 0.480357 | 0.215837 | 0.154712 | 0.28045 | 0.196386 | 0.004047 |  |
| MAFF | 1.094133 | 0.062575 | 0.029311 | 0.082907 | 0.038554 | 0.004053 |  |
| LPAR5 | 0.603155 | 0.124719 | 0.082104 | 0.174002 | 0.106024 | 0.004274 |  |
| SCML4 | 0.320885 | 0.41218 | 0.329982 | 0.482088 | 0.372289 | 0.004474 |  |
| RAB24 | 0.664671 | 0.11377 | 0.07177 | 0.158649 | 0.096386 | 0.004552 |  |
| DTNBP1 | 0.515423 | 0.187348 | 0.131066 | 0.241556 | 0.163855 | 0.004552 |  |
| CUL4A | 0.338037 | 0.292786 | 0.231628 | 0.371546 | 0.275904 | 0.004924 |  |
| HEXD | 0.427706 | 0.177361 | 0.131858 | 0.239509 | 0.159036 | 0.004957 |  |
| SCRN1 | 0.748635 | 0.105506 | 0.062794 | 0.137155 | 0.079518 | 0.005019 |  |
| ARRDC2 | 0.303377 | 0.524655 | 0.425156 | 0.553736 | 0.449398 | 0.00529 |  |
| NRN1 | 4.345784 | 0.01906 | 0.000937 | 0.020471 | 0.001205 | 0.005444 |  |
| SNN | 0.770241 | 0.091718 | 0.053776 | 0.125896 | 0.071084 | 0.005532 |  |
| GNLY | 1.273051 | 0.043637 | 0.018056 | 0.059365 | 0.022892 | 0.005576 |  |
| LDLR | 0.672319 | 0.130856 | 0.082112 | 0.155578 | 0.093976 | 0.00567 |  |
| SSH2 | 0.336934 | 0.377422 | 0.298813 | 0.458547 | 0.356627 | 0.00567 |  |
| LPP | 0.34585 | 0.270891 | 0.213149 | 0.353122 | 0.256627 | 0.005758 |  |
| NOCT | 0.466274 | 0.214009 | 0.154907 | 0.264074 | 0.184337 | 0.005772 |  |
| IARS1 | 0.565936 | 0.131684 | 0.088955 | 0.188332 | 0.120482 | 0.0058 |  |
| R3HDM2 | 0.354516 | 0.339886 | 0.265836 | 0.417605 | 0.325301 | 0.00582 |  |
| KLHL15 | 0.442741 | 0.239083 | 0.175902 | 0.298874 | 0.218072 | 0.005865 |  |
| CREG1 | 0.687265 | 0.090808 | 0.056395 | 0.122825 | 0.068675 | 0.005897 |  |
| ATP6V1F | 0.314058 | 0.340211 | 0.273658 | 0.431934 | 0.325301 | 0.005978 |  |
| FAS | 1.314195 | 0.045525 | 0.018308 | 0.065507 | 0.027711 | 0.006131 |  |
| TP53INP2 | 0.623876 | 0.150147 | 0.097434 | 0.194473 | 0.128916 | 0.006151 |  |
| ARFGAP3 | 0.467547 | 0.196145 | 0.141851 | 0.262027 | 0.181928 | 0.006178 |  |
| TAGLN2 | 0.303684 | 0.482933 | 0.391263 | 0.539406 | 0.443373 | 0.006178 |  |
| IFFO2 | 0.389976 | 0.303297 | 0.231459 | 0.351075 | 0.262651 | 0.006246 |  |
| DNAJA1 | 0.316515 | 0.496513 | 0.398704 | 0.549642 | 0.451807 | 0.006314 |  |
| PCNX4 | 0.42004 | 0.196063 | 0.146539 | 0.265097 | 0.181928 | 0.006368 |  |
| GATD1 | 0.398731 | 0.242329 | 0.183812 | 0.30911 | 0.221687 | 0.006378 |  |
| PPP1R16B | 0.441999 | 0.214738 | 0.158071 | 0.278403 | 0.19759 | 0.006756 |  |
| LY6E | 0.192819 | 1.00717 | 0.881168 | 0.804504 | 0.722892 | 0.006812 |  |
| GIMAP1 | 0.253839 | 0.644477 | 0.540499 | 0.643808 | 0.531325 | 0.007009 |  |
| FAM120B | 0.486025 | 0.182285 | 0.13015 | 0.235415 | 0.162651 | 0.0073 |  |
| FAM177A1 | 0.383649 | 0.273693 | 0.209785 | 0.346981 | 0.254217 | 0.0073 |  |
| FOXN2 | 0.311007 | 0.335691 | 0.270593 | 0.409417 | 0.313253 | 0.0073 |  |
| RAB10 | 0.289361 | 0.503056 | 0.411632 | 0.555783 | 0.449398 | 0.007419 |  |
| RASGRF2 | 0.291661 | 0.429205 | 0.350644 | 0.501535 | 0.387952 | 0.007555 |  |
| CRB3 | 4.440446 | 0.013974 | 0.000644 | 0.019447 | 0.001205 | 0.007601 |  |
| CRTC3 | 0.232322 | 0.624107 | 0.53128 | 0.665302 | 0.550602 | 0.007725 |  |
| TCEANC | 1.341424 | 0.039922 | 0.015755 | 0.054248 | 0.020482 | 0.0079 |  |
| PSMA1 | 0.519894 | 0.178355 | 0.124389 | 0.241556 | 0.171084 | 0.008003 |  |
| HNRNPK | 0.234892 | 0.673061 | 0.571932 | 0.69089 | 0.584337 | 0.008003 |  |
| TBC1D1 | 0.299621 | 0.384517 | 0.312407 | 0.467758 | 0.354217 | 0.008127 |  |
| TMEM88 | 0.646639 | 0.105711 | 0.067525 | 0.14739 | 0.089157 | 0.008149 |  |
| COMT | 0.342007 | 0.343324 | 0.270863 | 0.399181 | 0.303614 | 0.008149 |  |
| AKT2 | 0.303284 | 0.382539 | 0.310012 | 0.467758 | 0.36988 | 0.008149 |  |
| RNF144A | 0.283046 | 0.420946 | 0.345956 | 0.487206 | 0.374699 | 0.008331 |  |
| CSTF2T | 0.331986 | 0.306201 | 0.243259 | 0.374616 | 0.279518 | 0.008407 |  |
| MAGEF1 | 0.521234 | 0.156048 | 0.10873 | 0.207779 | 0.137349 | 0.008539 |  |
| SIDT1 | 0.442458 | 0.195019 | 0.14351 | 0.257932 | 0.180723 | 0.008579 |  |
| VPS37B | 0.406916 | 0.232316 | 0.17522 | 0.295803 | 0.212048 | 0.008579 |  |
| KPNA3 | 0.319081 | 0.284339 | 0.22792 | 0.374616 | 0.273494 | 0.00867 |  |
| SGMS1 | 0.452345 | 0.215133 | 0.157231 | 0.274309 | 0.19759 | 0.00922 |  |
| NGRN | 0.462264 | 0.188694 | 0.136962 | 0.252815 | 0.179518 | 0.00928 |  |
| MIB1 | 0.429196 | 0.231599 | 0.172003 | 0.291709 | 0.213253 | 0.009323 |  |
| JMJD6 | 0.37269 | 0.356516 | 0.275352 | 0.400205 | 0.313253 | 0.009474 |  |
| TSPYL4 | 0.340662 | 0.344157 | 0.271774 | 0.413511 | 0.322892 | 0.009513 |  |
| RORA | 0.29376 | 0.525768 | 0.428907 | 0.535312 | 0.446988 | 0.009513 |  |
| SEMA4D | 0.188616 | 0.83422 | 0.731984 | 0.784033 | 0.689157 | 0.009513 |  |
| YBX3 | 0.287134 | 0.512335 | 0.419873 | 0.520983 | 0.433735 | 0.009642 |  |
| INPP5A | 0.440085 | 0.172871 | 0.127422 | 0.230297 | 0.155422 | 0.009679 |  |
| IL2RB | 0.31014 | 0.431686 | 0.348182 | 0.469806 | 0.372289 | 0.00979 |  |
| RNF17 | 2.477512 | 0.01906 | 0.003422 | 0.026612 | 0.004819 | 0.009812 |  |
| **Gamma-delta T cells** | | |  |  |  |  |  |
| DDX3Y | 2.093034 | 0.487632 | 0.114295 | 0.481203 | 0.118705 | 5.42E-16 |  |
| UTY | 1.999433 | 0.265365 | 0.066367 | 0.31203 | 0.093525 | 2.06E-07 |  |
| KDM5D | 1.850223 | 0.243191 | 0.067449 | 0.289474 | 0.089928 | 1.8E-06 |  |
| MT-ND2 | 0.629147 | 1.178508 | 0.761975 | 0.793233 | 0.492806 | 3.88E-06 |  |
| PTGDR | 1.123667 | 0.413607 | 0.189815 | 0.443609 | 0.219424 | 1.75E-05 |  |
| MVB12B | 1.033205 | 0.438088 | 0.21406 | 0.462406 | 0.258993 | 5.85E-05 |  |
| USP9Y | 1.763779 | 0.193089 | 0.05686 | 0.244361 | 0.079137 | 6.02E-05 |  |
| KLRD1 | 0.87864 | 0.663257 | 0.360732 | 0.503759 | 0.273381 | 7.33E-05 |  |
| DUSP2 | 0.482957 | 1.452985 | 1.039625 | 0.804511 | 0.676259 | 7.33E-05 |  |
| LITAF | 0.363346 | 1.64692 | 1.280249 | 0.838346 | 0.769784 | 0.000151 |  |
| EIF1AY | 2.064452 | 0.162933 | 0.038953 | 0.199248 | 0.057554 | 0.000176 |  |
| B4GALT1 | 0.534665 | 0.912358 | 0.629817 | 0.710526 | 0.546763 | 0.001211 |  |
| NKG7 | 0.501902 | 1.249807 | 0.882583 | 0.759398 | 0.564748 | 0.00122 |  |
| SFMBT2 | 0.981303 | 0.374706 | 0.189797 | 0.406015 | 0.241007 | 0.001868 |  |
| CD84 | 0.786783 | 0.443937 | 0.257322 | 0.488722 | 0.309353 | 0.001992 |  |
| GNLY | 1.172977 | 0.422988 | 0.187598 | 0.270677 | 0.115108 | 0.002455 |  |
| ID2 | 0.635154 | 0.597098 | 0.384455 | 0.586466 | 0.388489 | 0.00258 |  |
| ITM2A | 0.603792 | 0.719149 | 0.473216 | 0.616541 | 0.442446 | 0.002605 |  |
| CDKN1B | 0.37533 | 1.151759 | 0.887924 | 0.845865 | 0.71223 | 0.002948 |  |
| RUNX3 | 0.472413 | 0.8745 | 0.630303 | 0.729323 | 0.564748 | 0.004595 |  |
| ABCA7 | 0.918464 | 0.334065 | 0.176744 | 0.387218 | 0.233813 | 0.004958 |  |
| FBXO32 | 1.240362 | 0.227657 | 0.096359 | 0.270677 | 0.125899 | 0.005143 |  |
| OXNAD1 | 0.689603 | 0.481026 | 0.298248 | 0.522556 | 0.356115 | 0.005143 |  |
| GPRIN3 | 0.618746 | 0.528377 | 0.344098 | 0.556391 | 0.384892 | 0.006134 |  |
| MRFAP1 | 1.73255 | 0.107967 | 0.032489 | 0.154135 | 0.046763 | 0.007888 |  |
| USP53 | 1.241089 | 0.197384 | 0.083504 | 0.236842 | 0.104317 | 0.007888 |  |
| SH3GLB2 | 0.860008 | 0.317607 | 0.174986 | 0.387218 | 0.230216 | 0.00902 |  |
| LRRC37A2 | 2.510236 | 0.080984 | 0.014215 | 0.112782 | 0.02518 | 0.009508 |  |
| TEC | 1.534051 | 0.124465 | 0.042979 | 0.169173 | 0.057554 | 0.009964 |  |

^1^log2FC - log fold-change of the average expression between the two groups (FF compared to HMF infants).

^2^FF – formula-fed.

^3^HMF - human milk fed.

^4^pct_FF, percent of cells with detected expression of the gene in FF infants.

^5^pct_HMF, percent of cells with detected expression the gene in HMF infants.

^6^p_val_adj, adjusted p-value based on Benjamini and Hochberg method using all genes in the dataset.

**Supplementa1 Figure 1.** Cell type UMAPs showing donor variation (Human milk (n=6) and formula fed (n=3) infants) for (A) all PBMCs, (B) monocytes, (C) NK (natural killer cells) cells, (D) B cells, (E) T cells and (F) Stacked bar plot depicting donor distribution by cell type.

**Supplemental Figure 2. An overview of the top 10 up-regulated pathways across PBMC subtypes. (A) A representative ComplexUpSet plot depicting the pathways that cells have in common. (B) A dot plot showing the pathways identified by the ComplexUpSet plot. The circle color indicates the number of pathways shared among the immune cell subtypes.**
